# Site‐Selective Late‐Stage Aromatic [^18^F]Fluorination via Aryl Sulfonium Salts

**DOI:** 10.1002/anie.201912567

**Published:** 2019-12-12

**Authors:** Peng Xu, Da Zhao, Florian Berger, Aboubakr Hamad, Jens Rickmeier, Roland Petzold, Mykhailo Kondratiuk, Kostiantyn Bohdan, Tobias Ritter

**Affiliations:** ^1^ Max-Planck-Institut für Kohlenforschung Kaiser-Wilhelm-Platz 1 45470 Mülheim an der Ruhr Germany

**Keywords:** ^18^F labeling, C−H functionalization, fluorination, late-stage functionalization, radiochemistry

## Abstract

Site‐selective functionalization of C−H bonds in small complex molecules is a long‐standing challenge in organic chemistry. Herein, we report a broadly applicable and site‐selective aromatic C−H dibenzothiophenylation reaction. The conceptual advantage of this transformation is further demonstrated through the two‐step C−H [^18^F]fluorination of a series of marketed small‐molecule drugs.

## Graphical abstract

**A broadly applicable** and site‐selective late‐stage aromatic [^18^F]fluorination reaction is reported. A collection of three electronically different dibenzothiophenes enables ^18^F labeling of a series of small complex molecules.

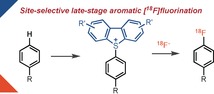

Over the past ten years, several promising new methods for the introduction of the ^18^F nucleus into small molecules have been disclosed, with the aim of improving clinical care and drug discovery through ^18^F positron‐emission tomography (PET).^1^, ^2^ However, implementation and translation to hospital settings is challenging if the fluorination reactions are operationally complex.^3^, ^4^ Unfortunately, the introduction of conventional leaving groups that can directly provide aryl fluorides upon reaction with fluoride cannot currently be accomplished at a late stage, so the requirement for de novo syntheses slows down the development of PET tracers (Scheme 1 a). Direct C−H fluorination in this regard is promising but selectivity becomes a substantial concern in every direct C−H functionalization reaction, especially for PET tracer synthesis because even small quantities of constitutional isomers must be separated (Scheme 1 b).^5^ There are few highly regioselective arene C−H functionalization reactions, and all of them either require suitable substitution patterns6a, 6d or directing groups,6b, 6c or afford functional groups that are not suitable for ^18^F fluorination.6e Herein, we report a highly selective and late‐stage C−H functionalization to afford aryl dibenzothiophenium salts, which can be converted into [^18^F]Ar‐F in a straightforward nucleophilic aromatic substitution reaction simply by adding fluoride (Scheme 1). The C−H dibenzothiophenylation reaction developed herein proceeds in high selectivity for both small complex molecules and simple monosubstituted arenes to afford aryl dibenzothiophenium salts. Given its high selectivity and the operational simplicity, this two‐step method to quickly access [^18^F]Ar‐F by arene C−H functionalization holds promise for the acceleration of ^18^F PET development.

**Scheme 1:**
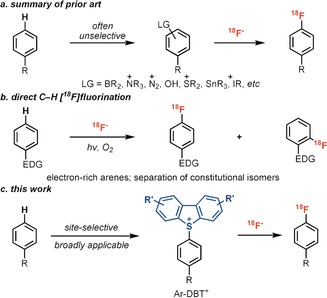
Strategies for aromatic C−^18^F bond formation.

Some of the modern ^18^F fluorination reactions are starting to have an impact on the synthesis of structurally complex [^18^F]labeled small molecules that cannot be made by conventional nucleophilic aromatic substitution.^7^ Most notably, practical [^18^F]fluorodemetallation reactions mediated by copper that proceed on a large variety of small molecules have been developed by the groups of Gouverneur,7c Sanford and Scott,7f, 7h and Neumaier.7d Our group has reported deoxyfluorination reactions7g, 7i that in addition can also functionalize small peptides.7l Sanford, Scott, and co‐workers have described a Cu‐mediated two‐step [^18^F]fluorination of electron‐rich arenes using hypervalent iodine compounds.7j Nicewicz, Li, and co‐workers have developed a promising C−H to C‐^18^F fluorination reaction that does not require coordination directing groups and is enabled by laser‐photoredox catalysis.5d None of the useful functional groups for F^18^ introduction can currently be introduced selectively at a late stage in a general sense. Direct [^18^F]fluorination is desirable, but control of regioselectivity is challenging.

Aryl sulfonium salts are good precursors for ^18^F labeling of arenes.^8^ Årstad and co‐workers have demonstrated that dibenzothiophene sulfonium salts can be efficiently converted into ^18^F‐labeled arenes for electron‐poor and electron‐neutral substrates, including several valuable PET tracers.8d However, reported methods for the preparation of aryl sulfonium salts often require aryl Grignard reagents, multistep syntheses from aryl halides, or strong acids as co‐solvents, none of which are suitable for site‐selective late‐stage incorporation.^8^, ^9^ Our previous approach through selective C−H thianthrenation^10^ can also provide access to aryl fluorides but requires an iridium‐catalyzed photoredox method,^11^ which adds additional challenges for implementation at good manufacturing practice (GMP) production facilities in radiopharmacies. Herein, we report a second highly selective arene C−H functionalization reaction, which differs conceptually from thianthrenation in that it can provide aryl sulfonium salts that can engage directly in C−F bond formation, simply through the addition of fluoride. Furthermore, we show how a group of three electronically different dibenzothiophenes designed for [^18^F]fluorination show a markedly expanded substrate scope compared to previous reported [^18^F]fluorinations of arylsulfonium compounds.^8^

Essential for the success of a site‐selective late‐stage ^18^F labeling method is the availability of a practical and general method to produce the precursors from readily available starting materials. We found that in the presence of acid anhydrides as activators,^12^ the reaction of bench‐stable dibenzothiophene *S*‐oxide with ethylbenzene afforded the corresponding dibenzothiophenium salt with high positional selectivity (*p*/*o*=50:1; *p*/*m*=100:1; Scheme 2). In contrast to previously reported arylthianthrenium salts, aryl dibenzothiophenium salts can provide aryl fluoride directly. For example, treatment of a biphenyl‐derived dibenzothiophenium salt with fluoride afforded the aryl fluoride in 84 % yield of isolated product, yet the corresponding thianthrenium salt gave the desired product in only 7 % yield with the wrong C−S cleavage product as the major side product (Scheme 2). The site‐selective C−H functionalization reaction developed herein exhibits broad substrate scope (Table 1). Both electron‐poor (**2**, **21**–**23**) and electron‐rich (**16**, **24**, **27**) arenes proceed efficiently with high regioselectivity. Various functional groups are well tolerated, including halides (**2**, **21**–**24**), nitriles (**3**), ethers (**4**–**5**, **20**), esters (**4**, **10**, **15**), ketones (**5**), aldehydes (**7**), amides (**5**, **8**, **25**), and sulfonamides (**9**, **14**). Heterocycles such as quinolines (**12**), imidazoles (**19**), and pyridines (**20**) are also compatible. Arenes that are more electron‐deficient than 1,2‐dichlorobenzene are too electron‐poor to react. The utility of our method for site‐selective late‐stage aromatic C−H functionalization is further demonstrated with the reactions of small complex substrates, drugs, agrochemicals, and natural products. For example, the dibenzothiophenium salt of fenofibrate (**10**) was obtained in 89 % yield of isolated product on a gram scale. For all the substrates shown in Table 1, analytically pure compounds can be obtained through simple chromatography on silica gel.

**Scheme 2:**
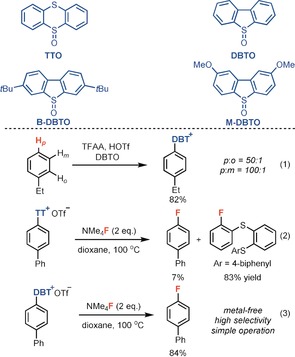
Synthesis and fluorination of aryl sulfonium salts. TTO=thianthrene *S*‐oxide; DBTO=dibenzothiophene *S*‐oxide; B‐DBTO=3,7‐di‐*tert*‐butyldibenzothiophene *S*‐oxide; M‐DBTO=2,8‐dimethoxydibenzothiophene *S*‐oxide.

**Table 1:** Site‐selective aromatic C−H dibenzothiophenylation. 
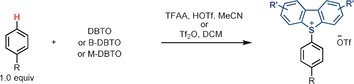

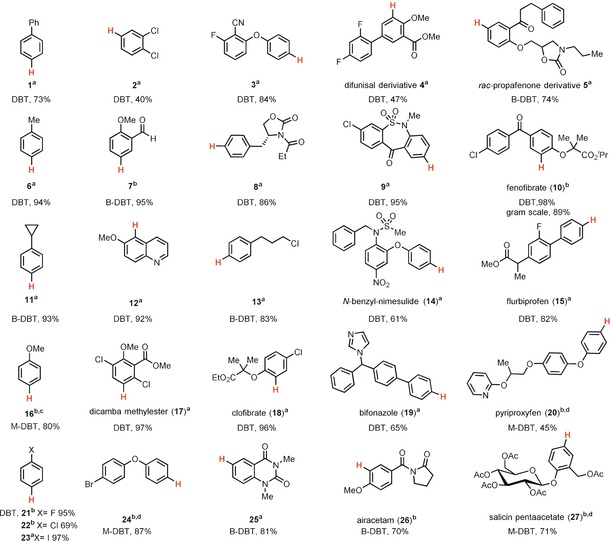

[a] Reaction condition A: 0.500 mmol arene, 2.00 to 4.00 equiv triflic acid (HOTf), 3.00 equiv trifluoroacetic anhydride (TFAA) and 1.50 to 2.00 equiv DBTO or B‐DBTO in MeCN (2.0 mL, *c*=0.25 m), −40 °C to 25 °C. [b] Reaction condition B: 0.500 mmol arene, 1.50 to 2.00 equiv DBTO, B‐DBTO or M‐DBTO in DCM (2.0 mL, *c*=0.25 m), 1.50 to 2.00 equiv triflic anhydride (Tf_2_O), −40 °C to 25 °C. [c] 3.00 equiv methanesulfonic anhydride instead of Tf_2_O, and reaction performed at 0 °C to 25 °C. [d] 2.00 equiv K_2_CO_3_ was added, and reaction performed at −78 °C to 25 °C.

As depicted in Scheme 1, three differently substituted dibenzothiophene *S*‐oxides display similar high site‐selectivity under otherwise identical reaction conditions. For unsymmetrical triaryl sulfonium salts, the S_N_Ar reaction occurs preferentially at the most electron‐deficient arene.^8^, ^13^ As such, an electron‐rich dibenzothiophen such as M‐DBT should provide high selectivity for the desired arene fluorination. However, M‐DBT is less reactive than more electron‐deficient dibenzothiophenes such as DBT, and cannot be used to efficiently C−H functionalize less electron‐rich arenes. Thus the dibenzothiophene *S*‐oxide selected for each arene should be electron‐deficient enough to functionalize the arene, and electron‐rich enough to provide high selectivity in fluorination. For example, both DBT and B‐DBT salts of compound **25** could be synthesized in good yields. However, fluorination of the B‐DBT salt affords 94 % yield of desired fluorination product while DBT salt gives only 55 % yield of desired product with 42 % yield of the undesired side‐product (see the Supporting Information). By use of electron‐rich M‐DBT, [^18^F]fluorination with aryl sulfoniums can now proceed on otherwise challenging electron‐rich complex substrates (**36**, **38**; Table 2).^8^ Moreover, a range of small‐molecule drugs were successfully ^18^F‐labeled. Halides (**30**–**32**, **34**, **35**, **37**), amides (**28**), sulfonamides (**31**), heterocycles (**33**, **38**) were well tolerated. Substrates bearing *ortho*‐substituents proceeded efficiently to afford the desired ^18^F‐labeled products (**29**, **30**, **32**, **37**).

**Table 2:** Aromatic [^18^F]fluorination. 
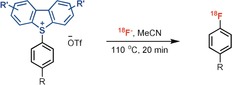

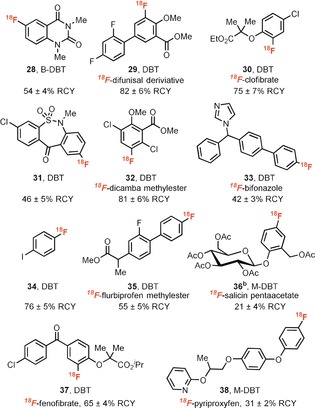

[a] Reaction conditions: aryl dibenzothiophenium precursor (9.0 μmol) in 500 μL MeCN at 110 °C for 20 min. RCY=decay‐corrected radiochemical yield. [b] Kryptofix 222 and K_2_CO_3_ were added, and reaction performed in 500 μL DMSO.

Elution of the [^18^F]fluoride from the anion exchange cartridge is commonly achieved with an aqueous solution of a base.^14^ Aryldibenzothiophenium salts can be used directly for elution of [^18^F]fluoride, which avoids the addition of bases or kryptofix.7g, 7i, 7l No special care is required to exclude air or moisture at any stage of the radio‐synthesis, and the radiolabeled product can be readily separated from the starting material due to the pronounced polarity difference owing to the cationic sulfonium salt. No carrier‐added [^18^F]fluorination enabled the automated synthesis of ^18^F‐labeled compound **32** in high specific activity (1.4 Ci μmol^−1^). A Hammett analysis of the [^19^F]fluorination with aryl dimethoxyldibenzothiophenium salts (Hammett‐slope *ρ*=+3.4) is consistent with a mechanism proceeding via C−F bond reductive elimination from hypervalent sulfurane as previously suggested.8d, 15

In conclusion, we developed a site‐selective late‐stage aromatic [^18^F]fluorination, enabled by a selective C−H dibenzothiophenylation reaction. We show for the first time how a collection of three electronically different dibenzothiophenes appropriately matched to the electronic requirements of the arene can expand the substrate scope compared to prior art. Beyond the immediate practicality of our method, this new procedure may inspire the development of diverse site‐selective reactions for carbon–heteroatom bond formation.

## Conflict of interest

The authors declare no conflict of interest.

## Supporting information

As a service to our authors and readers, this journal provides supporting information supplied by the authors. Such materials are peer reviewed and may be re‐organized for online delivery, but are not copy‐edited or typeset. Technical support issues arising from supporting information (other than missing files) should be addressed to the authors.

Supplementary
