# Supplementary material for: Site‐Selective Late‐Stage Aromatic [18F]Fluorination via Aryl Sulfonium Salts
Source: Angew Chem Int Ed Engl. 2019 Dec 12;59(5):1956–60. doi: 10.1002/anie.201912567 (PMC7004179; doi:10.1002/anie.201912567)

Angewandte Chemie
Eine Zeitschrift der Gesellschaft Deutscher Chemiker

Supporting Information

Site-Selective Late-Stage Aromatic [^{18}F]Fluorination via Aryl Sulfonium Salts

*Peng Xu[+], Da Zhao[+], Florian Berger, Aboubakr Hamad, Jens Rickmeier, Roland Petzold, Mykhailo Kondratiuk, Kostiantyn Bohdan, and Tobias Ritter**

anie_201912567_sm_miscellaneous_information.pdf

TABLE OF CONTENTS

TABLE OF CONTENTS 1

MATERIALS AND METHODS 10

EXPERIMENTAL DATA 11

Preparation of dibenthiophene *S*-oxide 11

Dibenzothiophene *S*-oxide (**DBTO**) 11

3,7-Di-*tert*-butyldibenzothiophene *S*-oxide (**B-DBTO**) 11

2,8-Dimethoxydibenzothiophene *S*-oxide (**M-DBTO**) 12

General procedure for dibenzothiophenylation of arenes 13

Reaction condition A for dibenzothiophenylation of arenes 13

Reaction condition B for dibenzothiophenylation of arenes 13

General guide for the selection of dibenzothiophene *S*-oxides 14

Dibenzothiophenylation of arenes 14

Biphenyl-derived dibenzothiophenium salt **S1** 14

1,2-Dichlorobenzene-derived dibenzothiophenium salt **S2** 15

2-Fluoro-6-phenoxybenzonitrile-derived dibenzothiophenium salt **S3** 16

Diflunisal derivative-derived dibenzothiophenium salt **S4** 17

rac-Propafenone derivative-derived 3,7-di-*tert*-butyldibenzothiophenium salt **S5** 18

Toluene-derived dibenzothiophenium salt **S6** 19

o-Anisaldehyde-derived 3,7-di-*tert*-butyldibenzothiophenium salt **S7** 20

(*R*)-(−)-4-Benzyl-3-propionyl-2-oxazolidinone-derived dibenzothiophenium salt **S8** 21

3-Chloro-6-methyldibenzo[*c*,*f*][1,2]thiazepin-11(6*H*)-one 5,5-dioxide-derived dibenzothiophenium salt **S9** 22

Fenofibrate-derived dibenzothiophenium salt **S10** 23

Cyclopropylbenzene-derived 3,7-di-*tert*-butyldibenzothiophenium salt **S11** 23

6-Methoxyquinoline-derived dibenzothiophenium salt **S12** 24

1-Chloro-3-phenylpropane-derived 3,7-di-*tert*-butyldibenzothiophenium salt **S13** 25

N-Benzyl-nimesulide-derived dibenzothiophenium salt **S14** 26

Flurbiprofen methylester-derived dibenzothiophenium salt **S15** 27

Anisole-derived 2,8-dimethoxydibenzothiophenium salt **S16** 28

Dicamba methylester (**17**) 29

Dicamba methylester-derived dibenzothiophenium salt **S17** 30

Clofibrate-derived dibenzothiophenium salt **S18** 31

Bifonazole-derived dibenzothiophenium salt **S19** 32

Pyriproxyfen-derived 2,8-dimethoxydibenzothiophenium salt **S20** 33
Fluorobenzene-derived dibenzothiophenium salt **S21** 34
Chlorobenzene-derived dibenzothiophenium salt **S22** 34
Iodobenzene-derived dibenzothiophenium salt **S23** 35
4-Bromodiphenylether-derived 2,8-dimethoxydibenzothiophenium salt **S24** 36
1,3-Dimethylquinazoline-2,4(1*H*,3*H*)-dione-derived 3,7-di-*tert*-butyldibenzothiophenium salt **S25** 37
1,3-Dimethylquinazoline-2,4(1*H*,3*H*)-dione-derived dibenzothiophenium salt **S25-DBT** 38
Airacetam-derived 3,7-di-*tert*-butyldibenzothiophenium salt **S26** 39
Salicin pentaacetate-derived 2,8-dimethoxydibenzothiophenium salt **S27** 40
Biphenyl-derived thianthrenium salt **1-TT** 41

Gram scale synthesis of fenofibrate-derived dibenzothiophenium salt **S10** 42

Comparison of fluorination of aryl thianthrenium salt and aryl dibenzothiophenium salt 42

Fluorination of biphenyl-derived thianthrenium salt **1-TT** 42
Fluorination of biphenyl-derived dibenzothiophenium salt **S1** 43

Fluorination of aryl dibenzothiophenium salts 44

6-Fluoro-1,3-dimethylquinazoline-2,4(1*H*,3*H*)-dione (**S28**) 44
Fluorodiflunisal derivative **S29** 45
Fluoroclofibrate (**S30**) 45
3-Chloro-9-fluoro-6-methyldibenzo[*c*,*f*][1,2]thiazepin-11(6*H*)-one 5,5-dioxide (**S31**) 46
Fluorodicamba methylester (**S32**) 47
Fluorobifonazole (**S33**) 48
Fluorofenofibrate (**S37**) 49

Comparison of fluorination of aryl dibenzothiophenium salts derived from DBTO and B-DBTO 49

Radiochemistry 51

General information 51
General procedure for pre-conditioning of QMA cartridges 52
General procedure for ^{18}F-fluorination 52
Reaction optimization 53
6-[^{18}F]Fluoro-1,3-dimethylquinazoline-2,4(1*H*,3*H*)-dione (**28**) 53
[^{18}F]Fluorodiflunisal derivative **29** 55
[^{18}F]Fluoroclofibrate (**30**) 57
3-Chloro-9-[^{18}F]fluoro-6-methyldibenzo[*c*,*f*][1,2]thiazepin-11(6*H*)-one 5,5-dioxide (**31**) 58
[^{18}F]Fluorodicamba methylester (**32**) 60
[^{18}F]Fluorobifonazole (**33**) 62
4-[^{18}F]Fluoroiodobenzene (**34**) 64
[^{18}F]Fluoroflurbiprofen methylester (**35**) 66

[^{18}F]Fluorosalicin pentaacetate (**36**) 68
[^{18}F]Fluorofenofibrate (**37**) 70
[^{18}F]Fluoropyriproxyfen (**38**) 72
Automated synthesis and determination of specific activity of [^{18}F]fluorodicamba methylester **32** 74
Hammett Analysis 75
General procedure for competition experiments: 76
General procedure for *in situ* preparation of aryl Grignard reagents 77
Anisole-derived 2,8-dimethoxydibenzothiophenium salt **S39** 78
Toluene-derived 2,8-dimethoxydibenzothiophenium salt **S40** 79
Benzene-derived 2,8-dimethoxydibenzothiophenium salt **S41** 80
Fluorobenzene-derived 2,8-dimethoxydibenzothiophenium salt **S42** 81
Chlorobenzene-derived 2,8-dimethoxydibenzothiophenium salt **S43** 82
Benzotrifluoride-derived 2,8-dimethoxydibenzothiophenium salt **S44** 83
Determination of Selectivity 83
Ethylbenzene-derived *para*-dibenzothiophenium salt **S45** 84
Ethylbenzene-derived *ortho*-dibenzothiophenium salt **S46** 85
Ethylbenzene-derived *meta*-dibenzothiophenium salt **S47** 85
Selectivity of dibenzothiophenylation of ethylbenzene 87
Example of dibenzothiophenylation of arene with moderate selectivity 89
REFERENCES 91
SPECTROSCOPIC DATA 93
^{1}H NMR of dibenzothiophene *S*-oxide (**DBTO**) 93
^{13}C NMR of dibenzothiophene *S*-oxide (**DBTO**) 94
^{1}H NMR of 3,7-di-*tert*-butyldibenzothiophene *S*-oxide (**B-DBTO**) 95
^{13}C NMR of 3,7-di-*tert*-butyldibenzothiophene *S*-oxide (**B-DBTO**) 96
^{1}H NMR of 2,8-dimethoxydibenzothiophene *S*-oxide (**M-DBTO**) 97
^{13}C NMR of 2,8-dimethoxydibenzothiophene *S*-oxide (**M-DBTO**) 98
^{1}H NMR of biphenyl-derived dibenzothiophenium salt **S1** 99
^{13}C NMR of biphenyl-derived dibenzothiophenium salt **S1** 100
^{19}F NMR of biphenyl-derived dibenzothiophenium salt **S1** 101
^{1}H NMR of 1,2-dichlorobenzene-derived dibenzothiophenium salt **S2** 102
^{13}C NMR of 1,2-dichlorobenzene-derived dibenzothiophenium salt **S2** 103

^{19}F NMR of 1,2-dichlorobenzene-derived dibenzothiophenium salt **S2** 104

^{1}H NMR of 2-fluoro-6-phenoxybenzonitrile-derived dibenzothiophenium salt **S3** 105

^{13}C NMR of 2-fluoro-6-phenoxybenzonitrile-derived dibenzothiophenium salt **S3** 106

^{19}F NMR of 2-fluoro-6-phenoxybenzonitrile-derived dibenzothiophenium salt **S3** 107

^{1}H NMR of diflunisal derivative-derived dibenzothiophenium salt **S4** 108

^{13}C NMR of diflunisal derivative-derived dibenzothiophenium salt **S4** 109

^{19}F NMR of diflunisal derivative-derived dibenzothiophenium salt **S4** 110

^{1}H NMR of *rac*-propafenone derivative-derived 3,7-di-*tert*-butyldibenzothiophenium salt **S5** 111

^{13}C NMR of *rac*-propafenone derivative-derived 3,7-di-*tert*-butyldibenzothiophenium salt **S5** 112

^{19}F NMR of *rac*-propafenone derivative-derived 3,7-di-*tert*-butyldibenzothiophenium salt **S5** 113

^{1}H NMR of toluene-derived dibenzothiophenium salt **S6** 114

^{13}C NMR of toluene-derived dibenzothiophenium salt **S6** 115

^{19}F NMR of toluene-derived dibenzothiophenium salt **S6** 116

^{1}H NMR of *o*-anisaldehyde-derived 3,7-di-*tert*-butyldibenzothiophenium salt **S7** 117

^{13}C NMR of *o*-anisaldehyde-derived 3,7-di-*tert*-butyldibenzothiophenium salt **S7** 118

^{19}F NMR of *o*-anisaldehyde-derived 3,7-di-*tert*-butyldibenzothiophenium salt **S7** 119

^{1}H NMR of (*R*)-(−)-4-benzyl-3-propionyl-2-oxazolidinone-derived dibenzothiophenium salt **S8** 120

^{13}C NMR of (*R*)-(−)-4-benzyl-3-propionyl-2-oxazolidinone-derived dibenzothiophenium salt **S8** 121

^{19}F NMR of (*R*)-(−)-4-benzyl-3-propionyl-2-oxazolidinone-derived dibenzothiophenium salt **S8** 122

^{1}H NMR of 3-chloro-6-methyldibenzo[*c*,*f*][1,2]thiazepin-11(6*H*)-one 5,5-dioxide-derived dibenzothiophenium salt **S9** 123

^{13}C NMR of 3-chloro-6-methyldibenzo[*c*,*f*][1,2]thiazepin-11(6*H*)-one 5,5-dioxide-derived dibenzothiophenium salt **S9** 124

^{19}F NMR of 3-chloro-6-methyldibenzo[*c*,*f*][1,2]thiazepin-11(6*H*)-one 5,5-dioxide-derived dibenzothiophenium salt **S9** 125

^{1}H NMR of fenofibrate-derived dibenzothiophenium salt **S10** 126

^{13}C NMR of fenofibrate-derived dibenzothiophenium salt **S10** 127

^{19}F NMR of fenofibrate-derived dibenzothiophenium salt **S10** 128

^{1}H NMR of cyclopropylbenzene-derived 3,7-di-*tert*-butyldibenzothiophenium salt **S11** 129

^{13}C NMR of cyclopropylbenzene-derived 3,7-di-*tert*-butyldibenzothiophenium salt **S11** 130

^{19}F NMR of cyclopropylbenzene-derived 3,7-di-*tert*-butyldibenzothiophenium salt **S11** 131

^{1}H NMR of 6-methoxyquinoline-derived dibenzothiophenium salt **S12** 132

^{13}C NMR of 6-methoxyquinoline-derived dibenzothiophenium salt **S12** 133

^{19}F NMR of 6-methoxyquinoline-derived dibenzothiophenium salt **S12** 134

^{1}H NMR of 1-chloro-3-phenylpropane-derived 3,7-di-*tert*-butyldibenzothiophenium salt **S13** 135

^{13}C NMR of 1-chloro-3-phenylpropane-derived 3,7-di-*tert*-butyldibenzothiophenium salt **S13** 136

^{19}F NMR of 1-chloro-3-phenylpropane-derived 3,7-di-*tert*-butyldibenzothiophenium salt **S13** 137

^{1}H NMR of *N*-benzyl-nimesulide-derived dibenzothiophenium salt **S14** 138

^{13}C NMR of *N*-benzyl-nimesulide-derived dibenzothiophenium salt **S14** 139

^{19}F NMR of *N*-benzyl-nimesulide-derived dibenzothiophenium salt **S14** 140

^{1}H NMR of flurbiprofen methylester-derived dibenzothiophenium salt **S15** 141

^{13}C NMR of flurbiprofen methylester-derived dibenzothiophenium salt **S15** 142

^{19}F NMR of flurbiprofen methylester-derived dibenzothiophenium salt **S15** 143

^{1}H NMR of anisole-derived 2,8-dimethoxydibenzothiophenium salt **S16** 144

^{13}C NMR of anisole-derived 2,8-dimethoxydibenzothiophenium salt **S16** 145

^{19}F NMR of anisole-derived 2,8-dimethoxydibenzothiophenium salt **S16** 146

^{1}H NMR of dicamba methylester (**17**) 147

^{13}C NMR of dicamba methylester (**17**) 148

^{1}H NMR of dicamba methylester-derived dibenzothiophenium salt **S17** 149

^{13}C NMR of dicamba methylester-derived dibenzothiophenium salt **S17** 150

^{19}F NMR of dicamba methylester-derived dibenzothiophenium salt **S17** 151

^{1}H NMR of clofibrate-derived dibenzothiophenium salt **S18** 152

^{13}C NMR of clofibrate-derived dibenzothiophenium salt **S18** 153

^{19}F NMR of clofibrate-derived dibenzothiophenium salt **S18** 154

^{1}H NMR of bifonazole-derived dibenzothiophenium salt **S19** 155

^{13}C NMR of bifonazole-derived dibenzothiophenium salt **S19** 156

^{19}F NMR of bifonazole-derived dibenzothiophenium salt **S19** 157

^{1}H NMR of pyriproxyfen-derived 2,8-dimethoxydibenzothiophenium salt **S20** 158

^{13}C NMR of pyriproxyfen-derived 2,8-dimethoxydibenzothiophenium salt **S20** 159

^{19}F NMR of pyriproxyfen-derived 2,8-dimethoxydibenzothiophenium salt **S20** 160

^{1}H NMR of fluorobenzene-derived dibenzothiophenium salt **S21** 161

^{13}C NMR of fluorobenzene-derived dibenzothiophenium salt **S21** 162

^{19}F NMR of fluorobenzene-derived dibenzothiophenium salt **S21** 163

^{1}H NMR of chlorobenzene-derived dibenzothiophenium salt **S22** 164

^{13}C NMR of chlorobenzene-derived dibenzothiophenium salt **S22** 165

^{19}F NMR of chlorobenzene-derived dibenzothiophenium salt **S22** 166

^{1}H NMR of iodobenzene-derived dibenzothiophenium salt **S23** 167

^{13}C NMR of iodobenzene-derived dibenzothiophenium salt **S23** 168

^{19}F NMR of iodobenzene-derived dibenzothiophenium salt **S23** 169

^{1}H NMR of 4-bromodiphenylether-derived 2,8-dimethoxydibenzothiophenium salt **S24** 170

^{13}C NMR of 4-bromodiphenylether-derived 2,8-dimethoxydibenzothiophenium salt **S24** 171

^{19}F NMR of 4-bromodiphenylether-derived 2,8-dimethoxydibenzothiophenium salt **S24** 172

^{1}H NMR of 1,3-dimethylquinazoline-2,4(1*H*,3*H*)-dione-derived 3,7-di-*tert*-butyldibenzothiophenium salt **S25** 173

^{13}C NMR of 1,3-dimethylquinazoline-2,4(1*H*,3*H*)-dione-derived 3,7-di-*tert*-butyldibenzothiophenium salt **S25** 174

^{19}F NMR of 1,3-dimethylquinazoline-2,4(1*H*,3*H*)-dione-derived 3,7-di-*tert*-butyldibenzothiophenium salt **S25** 175

^{1}H NMR of 1,3-dimethylquinazoline-2,4(1*H*,3*H*)-dione-derived dibenzothiophenium salt **S25-DBT** 176

^{13}C NMR of 1,3-dimethylquinazoline-2,4(1*H*,3*H*)-dione-derived dibenzothiophenium salt **S25-DBT** 177

^{19}F NMR of 1,3-dimethylquinazoline-2,4(1*H*,3*H*)-dione-derived dibenzothiophenium salt **S25-DBT** 178

^{1}H NMR of airacetam-derived 3,7-di-*tert*-butyldibenzothiophenium salt **S26** 179

^{13}C NMR of airacetam-derived 3,7-di-*tert*-butyldibenzothiophenium salt **S26** 180

^{19}F NMR of airacetam-derived 3,7-di-*tert*-butyldibenzothiophenium salt **S26** 181

^{1}H NMR of salicin pentaacetate-derived 2,8-dimethoxydibenzothiophenium salt **S27** 182

^{13}C NMR of salicin pentaacetate-derived 2,8-dimethoxydibenzothiophenium salt **S27** 183

^{19}F NMR of salicin pentaacetate-derived 2,8-dimethoxydibenzothiophenium salt **S27** 184

^{1}H NMR of biphenyl-derived thianthrenium salt **1-TT** ... 185

^{13}C NMR of biphenyl-derived thianthrenium salt **1-TT** ... 186

^{19}F NMR of biphenyl-derived thianthrenium salt **1-TT** ... 187

^{1}H NMR of 4-fluorobiphenyl (**1a**) ... 188

^{13}C NMR of 4-fluorobiphenyl (**1a**) ... 189

^{19}F NMR of 4-fluorobiphenyl (**1a**) ... 190

^{1}H NMR of [1,1'-biphenyl]-4-yl(2-((2-fluorophenyl)thio)phenyl)sulfane (**1b**) ... 191

^{13}C NMR of [1,1'-biphenyl]-4-yl(2-((2-fluorophenyl)thio)phenyl)sulfane (**1b**) ... 192

^{19}F NMR of [1,1'-biphenyl]-4-yl(2-((2-fluorophenyl)thio)phenyl)sulfane (**1b**) ... 193

^{1}H NMR of 6-fluoro-1,3-dimethylquinazoline-2,4(1*H*,3*H*)-dione (**S28**) ... 194

^{13}C NMR of 6-fluoro-1,3-dimethylquinazoline-2,4(1*H*,3*H*)-dione (**S28**) ... 195

^{19}F NMR of 6-fluoro-1,3-dimethylquinazoline-2,4(1*H*,3*H*)-dione (**S28**) ... 196

^{1}H NMR of fluorodiflunsial derivative **S29** ... 197

^{13}C NMR of fluorodiflunsial derivative **S29** ... 198

^{19}F NMR of fluorodiflunsial derivative **S29** ... 199

^{1}H NMR of fluoroclofibrate (**S30**) ... 200

^{13}C NMR of fluoroclofibrate (**S30**) ... 201

^{19}F NMR of fluoroclofibrate (**S30**) ... 202

^{1}H NMR of 3-chloro-9-fluoro-6-methyldibenzo[*c*,*f*][1,2]thiazepin-11(6*H*)-one 5,5-dioxide (**S31**) ... 203

^{13}C NMR of 3-chloro-9-fluoro-6-methyldibenzo[*c*,*f*][1,2]thiazepin-11(6*H*)-one 5,5-dioxide (**S31**) ... 204

^{19}F NMR of 3-chloro-9-fluoro-6-methyldibenzo[*c*,*f*][1,2]thiazepin-11(6*H*)-one 5,5-dioxide (**S31**) ... 205

^{1}H NMR of fluorodicamba methylester (**S32**) ... 206

^{13}C NMR of fluorodicamba methylester (**S32**) ... 207

^{19}F NMR of fluorodicamba methylester (**S32**) ... 208

^{1}H NMR of fluorobifonazole (**S33**) ... 209

^{13}C NMR of fluorobifonazole (**S33**) ... 210

^{19}F NMR of fluorobifonazole (**S33**) ... 211

^{1}H NMR of fluorofenofibrate (**S37**) ... 212

^{13}C NMR of fluorofenofibrate (**S37**) ... 213

^{19}F NMR of fluorofenofibrate (**S37**)....214

^{1}H NMR of anisole-derived 2,8-dimethoxydibenzothiophenium salt **S39**....215

^{13}C NMR of anisole-derived 2,8-dimethoxydibenzothiophenium salt **S39**....216

^{19}F NMR of anisole-derived 2,8-dimethoxydibenzothiophenium salt **S39**....217

^{1}H NMR of toluene-derived 2,8-dimethoxydibenzothiophenium salt **S40**....218

^{13}C NMR of toluene-derived 2,8-dimethoxydibenzothiophenium salt **S40**....219

^{19}F NMR of toluene-derived 2,8-dimethoxydibenzothiophenium salt **S40**....220

^{1}H NMR of benzene-derived 2,8-dimethoxydibenzothiophenium salt **S41**....221

^{13}C NMR of benzene-derived 2,8-dimethoxydibenzothiophenium salt **S41**....222

^{19}F NMR of benzene-derived 2,8-dimethoxydibenzothiophenium salt **S41**....223

^{1}H NMR of fluorobenzene-derived 2,8-dimethoxydibenzothiophenium salt **S42**....224

^{13}C NMR of fluorobenzene-derived 2,8-dimethoxydibenzothiophenium salt **S42**....225

^{19}F NMR of fluorobenzene-derived 2,8-dimethoxydibenzothiophenium salt **S42**....226

^{1}H NMR of chlorobenzene-derived 2,8-dimethoxydibenzothiophenium salt **S43**....227

^{13}C NMR of chlorobenzene-derived 2,8-dimethoxydibenzothiophenium salt **S43**....228

^{19}F NMR of chlorobenzene-derived 2,8-dimethoxydibenzothiophenium salt **S43**....229

^{1}H NMR of benzotrifluoride-derived 2,8-dimethoxydibenzothiophenium salt **S44**....230

^{13}C NMR of benzotrifluoride-derived 2,8-dimethoxydibenzothiophenium salt **S44**....231

^{19}F NMR of benzotrifluoride-derived 2,8-dimethoxydibenzothiophenium salt **S44**....232

^{1}H NMR of ethylbenzene-derived *para*-dibenzothiophenium salt **S45**....233

^{13}C NMR of ethylbenzene-derived *para*-dibenzothiophenium salt **S45**....234

^{19}F NMR of ethylbenzene-derived *para*-dibenzothiophenium salt **S45**....235

^{1}H NMR of ethylbenzene-derived *ortho*-dibenzothiophenium salt **S46**....236

^{13}C NMR of ethylbenzene-derived *ortho*-dibenzothiophenium salt **S46**....237

^{19}F NMR of ethylbenzene-derived *ortho*-dibenzothiophenium salt **S46**....238

^{1}H NMR of ethylbenzene-derived *meta*-dibenzothiophenium salt **S47**....239

^{13}C NMR of ethylbenzene-derived *meta*-dibenzothiophenium salt **S47**....240

^{19}F NMR of ethylbenzene-derived *meta*-dibenzothiophenium salt **S47**....241

^{1}H NMR of ibuprofen methylester-derived dibenzothiophenium salts **S48a** and **S48b**....242

^{13}C NMR of ibuprofen methylester-derived dibenzothiophenium salts **S48a** and **S48b** 243

^{19}F NMR of ibuprofen methylester-derived dibenzothiophenium salts **S48a** and **S48b**........................... 244

MATERIALS AND METHODS

All air- and moisture-insensitive reactions were carried out under an ambient atmosphere and monitored by thin-layer chromatography (TLC). All air- and moisture-sensitive manipulations were performed using standard *Schlenk* and glove-box techniques under an atmosphere of nitrogen. Concentration under reduced pressure was performed by rotary evaporation at 25–40 °C at an appropriate pressure, unless otherwise stated. Purified compounds were further dried under high vacuum (0.008–0.5 Torr). Yields refer to purified and spectroscopically pure compounds, unless otherwise stated.

Solvents

Anhydrous DCM, MeCN, THF, and 1,4-dioxane were obtained from Phoenix Solvent Drying Systems. All deuterated solvents were purchased from Euriso-Top.

Chromatography

Thin layer chromatography (TLC) was performed using EMD TLC silica gel 60 F254 plates pre-coated with 250 μm thickness silica gel and visualized by fluorescence quenching under UV light, permanganate stain, cerium ammonium molybdate stain or phosphomolybdic acid stain. Preparative HPLC separation was executed on Shimadzu Prominence Preparative HPLC system.

Spectroscopy and Instruments

NMR spectra were recorded on a Bruker *Ascend*™ 500 spectrometer operating at 500 MHz, 471 MHz, and 126 MHz, for ^{1}H, ^{19}F, and ^{13}C acquisitions, respectively. Chemical shifts are reported in ppm with the solvent residual peak as the internal standard. For ^{1}H NMR: $CDCl_3$, δ 7.26; CD_3CN, δ 1.94; CD_2Cl_2, δ 5.32, acetone-d_{6}, δ 2.05. For ^{13}C NMR: $CDCl_3$, δ 77.16; CD_3CN, δ 1.32; CD_2Cl_2, δ 53.84.[1] Data is reported as follows: s = singlet, d = doublet, t = triplet, q = quartet, m = multiplet, br = broad; coupling constants in Hz; integration.

Starting materials

All substrates and materials were used as received from commercial suppliers, unless otherwise stated. CsF was purchased from TCI and stored in glovebox. Tetrabutylammonium difluorotriphenylsilicate (TBAT) and tetramethylammonium fluoride (TMAF) were purchased from Sigma Aldrich and stored in glovebox. Anhydrous $AlCl_3$ was purchased from TCI and stored in glovebox. Trifluoromethanesulfonic anhydride was purchased from Fluorochem and stored in glovebox. Methanesulfonic anhydride was purchased from Alfa Aesar and stored in glovebox. Trifluoroacetic anhydride was purchased from abcr. 2,8-dimethoxydibenzothiophene was synthesized according to the previous reported method.[2] Difunisal deriviative **4** and *rac*-propafenone derivative **5** were synthesized according to the previous reported literature.[3] *N*-benzyl-nimesulide (**14**) was made according to the reported literature.[4] 4-Fluoroiodobenzene (**S34**) was purchased form Sigma Aldrich. Fluoroflurbiprofen methylester (**S35**), fluorosalicin pentaacetate (**S36**), and fluoropyriproxyfen (**S38**) were synthesized according to the literature.[5]

All substrates and reagents were used as received from commercial suppliers unless otherwise stated.

EXPERIMENTAL DATA

Preparation of dibenthiophene *S*-oxide

Dibenzothiophene *S*-oxide (DBTO)

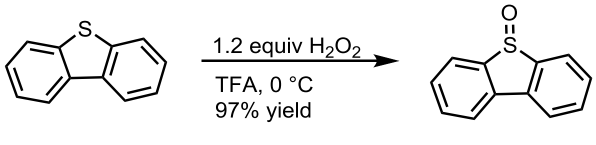

Dibenzothiophene (3.68 g, 20.0 mmol, 1.00 equiv.) suspended in trifluoroactetic acid (20 mL) was cooled to 0 °C (water/ice bath). Hydrogen peroxide solution (35 wt. % in H_2O, 2.05 mL, 2.33 g, 24.0 mmol, 1.20 equiv.) was added dropwise to the reaction mixture within 1 min. The reaction mixture was allowed to stir at 0 °C, and the completion of reaction was confirmed by TLC monitoring. No suspended solid was left at this point. The reaction mixture was poured onto a mixture of ice (100 g), saturated aqueous Na_2CO_3 solution (100 mL), and DCM (100 mL). The entire mixture was transferred into a separatory funnel, and the layers were separated. The DCM layer was collected, and the aqueous layer was further extracted with DCM (4 × ca. 60 mL). The combined organic layer was dried over Na_2SO_4, filtered, and the solvent was removed under reduced pressure. The residue was washed with hexanes (2 × 10 mL) and dried *in vacuo* to afford dibenzothiophene *S*-oxide (**DBTO**) (3.88 g, 19.4 mmol, 97%) as a white solid. The spectra corresponded to the data reported previously.[6]

R*f* = 0.52 (EtOAc).

NMR Spectroscopy:

^{1}H NMR (500 MHz, $CDCl_3$, 298 K, δ): 7.98 (d, *J* = 7.7 Hz, 2H), 7.79 (d, *J* = 7.7 Hz, 2H), 7.58 (tt, *J* = 7.6, 1.1 Hz, 2H), 7.49 (tt, *J* = 7.5, 1.1 Hz, 2H) ppm.

^{13}C NMR (126 MHz, $CDCl_3$, 298 K, δ): 144.9, 136.9, 132.4, 129.4, 127.3, 121.9 ppm.

3,7-Di-*tert*-butyldibenzothiophene *S*-oxide (B-DBTO)

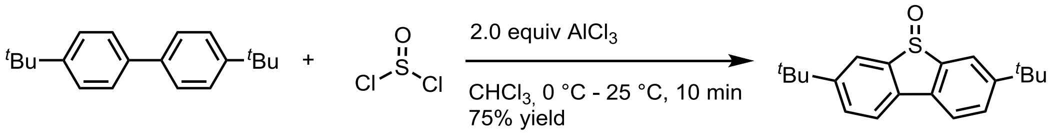

A flame-dried, 250 mL nitrogen-filled *Schlenk*-tube equipped with a magnetic stir bar was charged with 4,4´-di-*tert*-butylbiphenyl (2.66 g, 10.0 mmol, 1.00 equiv.) and $CHCl_3$ (50.0 mL, c = 0.20 M). After cooling to 0 °C (water/ice bath), thionyl chloride (1.09 mL, 1.78 g, 15.0 mmol, 1.50 equiv.) was added dropwise to the reaction mixture. Subsequently, anhydrous $AlCl_3$ (2.67 g, 20.0 mmol, 2.00 equiv.) was added in one portion to the stirred reaction mixture, and the *Schlenk*-tube was immediately taken out of the cold bath. After stirring vigorously at 25 °C for 10 min, the reaction mixture was poured onto an ice-water mixture (100 mL), and 10

wt. % aqueous NaOH (100 mL) was added to the mixture. The entire mixture was poured into a separatory funnel, and the layers were separated. The $CHCl_3$ layer was collected, and the aqueous layer was further extracted with DCM (3 × ca. 100 mL). The combined organic layers were dried over Na_2SO_4, filtered, and the solvent was removed under reduced pressure. The residue was purified by chromatography on silica gel eluting with hexanes/DCM/ethyl acetate (8:1:1, v/v/v). The product was collected and dried *in vacuo* to afford 3,7-di-*tert*-butyldibenzothiophene *S*-oxide (**B-DBTO**) (2.33 g, 7.46 mmol, 75%) as a white solid.

R*f* = 0.30 (DCM).

NMR Spectroscopy:

^{1}**H NMR** (500 MHz, $CDCl_3$, 298 K, δ): 7.95 (s, 2H), 7.62 (d, *J* = 8.1 Hz, 2H), 7.54 (d, *J* = 8.1 Hz, 2H), 1.33 (s, 18H) ppm.

^{13}**C NMR** (126 MHz, $CDCl_3$, 298 K, δ): 152.9, 145.1, 134.3, 129.7, 124.1, 121.2, 35.2, 31.2 ppm.

HRMS-EI (m/z) calculated for $C_{20}H_{24}OS$ $[M]^+$, 312.1542; found, 312.1542; deviation: 0.1 ppm.

2,8-Dimethoxydibenzothiophene *S*-oxide (M-DBTO)

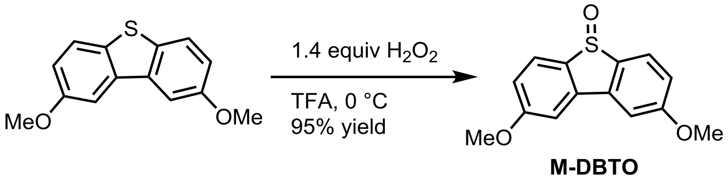

2,8-Dimethoxydibenzothiophene (4.89 g, 20.0 mmol, 1.00 equiv.) dissolved in trifluoroactetic acid (20 mL) was cooled to 0 °C (water/ice bath). Hydrogen peroxide solution (35 wt. % in H_2O, 2.05 mL, 2.33 g, 24.0 mmol, 1.20 equiv.) was added dropwise to the reaction mixture within 1 min. After stirring at 0 °C for 2 h, hydrogen peroxide solution (35 wt. % in H_2O, 171 µL, 194 mg, 2.00 mmol, 0.100 equiv.) was added to the reaction mixture. After 30 min, additional hydrogen peroxide solution (35 wt. % in H_2O, 171 µL, 194 mg, 2.00 mmol, 0.100 equiv.) was added. The reaction completion was confirmed by TLC, and the reaction mixture was poured onto a mixture of ice (100 g), saturated aqueous Na_2CO_3 solution (100 mL), and DCM (100 mL). The reaction mixture was transferred into a separatory funnel, and the layers were separated. The DCM layer was collected, and the aqueous layer was further extracted with DCM (4 × ca. 60 mL). The combined organic layer was dried over Na_2SO_4, filtered, and the solvent was removed under reduced pressure. The residue was washed with Et_2O (2 × 10 mL) and dried *in vacuo* to afford 2,8-dimethoxydibenzothiophene *S*-oxide (**M-DBTO**) (5.05 g, 19.0 mmol, 95%) as a pale orange solid.

R*f* = 0.43 (EtOAc).

NMR Spectroscopy:

^{1}**H NMR** (500 MHz, $CDCl_3$, 298 K, δ): 7.86 (d, *J* = 6.12 Hz, 2H), 7.23 (d, *J* = 5.99 Hz, 2H), 6.99 (dd, *J* = 6.24 Hz, 2H), 3.93 (s, 6H) ppm.

^{13}**C NMR** (126 MHz, $CDCl_3$, 298 K, δ): 163.3, 139.2, 137.9, 128.8, 115.1, 107.3, 55.9 ppm.

HRMS-ESI (m/z) calculated for $C_{14}H_{12}O_3SN_a^+$ [M+Na]$^+$, 283.0399; found, 283.0396; deviation: 1.1 ppm.

General procedure for dibenzothiophenylation of arenes

Reaction condition A for dibenzothiophenylation of arenes

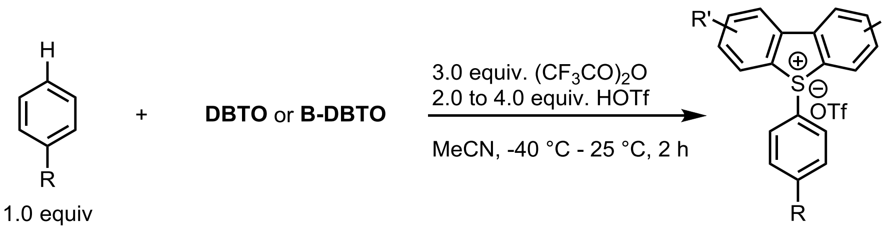

A flame-dried, 10 mL nitrogen-filled *Schlenk*-tube equipped with a magnetic stir bar was charged with arene (0.500 mmol, 1.00 equiv.) and dry MeCN (2.0 mL, c = 0.25 M) at 25 °C. After cooling to –40 °C (acetonitrile/dry ice bath), trifluoromethanesulfonic acid (1.00 to 2.00 mmol, 2.00 to 4.00 equiv.) and trifluoroacetic anhydride (209 µL, 315 mg, 1.50 mmol, 3.00 equiv.) were added to the stirred reaction mixture. Subsequently, (3,7-di-*tert*-butyl)dibenzothiophene *S*-oxide (**DBTO** or **B-DBTO**) (0.750 or 1.00 mmol, 1.50 or 2.00 equiv.) was added to the stirred reaction mixture in small portions over 1 min. After addition, the reaction mixture was stirred at –40 °C for 1 h. Subsequently, the *Schlenk*-tube was taken out of the cold bath and warmed to 25 °C in air. After stirring at 25 °C for another 1 h, the reaction mixture was diluted with DCM (10 mL) and poured onto saturated aqueous $NaHCO_3$ (10 mL). The mixture was concentrated under reduced pressure to remove most of the MeCN solvent, and the residue was diluted with 20 mL DCM and 10 mL water. The mixture was poured into a separatory funnel, and the layers were separated. The DCM layer was collected, and the aqueous layer was further extracted with DCM (4 × ca. 30 mL). The combined DCM layer was dried over Na_2SO_4, filtered, and the solvent was removed under reduced pressure. The residue was purified by chromatography on silica gel.

Reaction condition B for dibenzothiophenylation of arenes

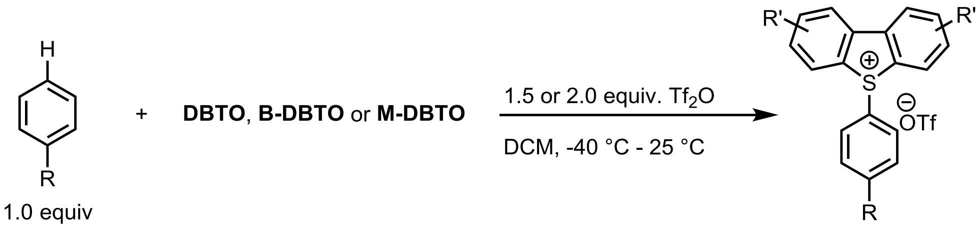

A flame-dried, 10 mL nitrogen-filled *Schlenk*-tube equipped with a magnetic stir bar was charged with arene (0.500 mmol, 1.00 equiv.), (3,7-di-*tert*-butyl or 2,8-dimethoxy)dibenzothiophene *S*-oxide (**DBTO**, **B-DBTO** or **M-DBTO**) (0.750 or 1.00 mmol, 1.50 or 2.00 equiv.), and dry DCM (2.0 mL, c = 0.25 M) at 25 °C. After cooling to –40 °C (acetonitrile/dry ice bath), trifluoromethanesulfonic anhydride (0.750 or 1.00 mmol, 1.50 or 2.00 equiv.) was added dropwise over 1 min. The *Schlenk*-tube was sealed, taken out of the cold bath and warmed to 25 °C in air. After stirring at 25 °C for a certain time, the reaction mixture was diluted with DCM (5.0 mL) and poured onto a mixture of DCM (20 mL) and saturated aqueous $NaHCO_3$ (5.0 or 10.0 mL). The

mixture was poured into a separatory funnel, and the layers were separated. The DCM layer was collected, and the aqueous layer was further extracted with DCM (4 × ca. 30 mL). The combined DCM layer was dried over Na_2SO_4, filtered, and the solvent was removed under reduced pressure. The residue was purified by chromatography on silica gel.

General guide for the selection of dibenzothiophene *S*-oxides

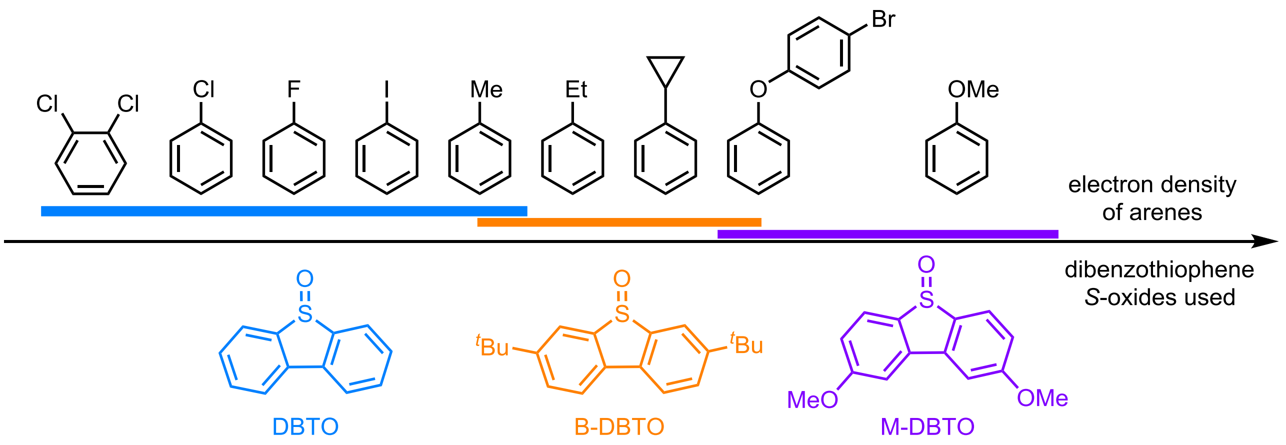

Dibenzothiophene *S*-oxides used should match the electron density of arenes. The selection of suitable dibenzothiophene *S*-oxide is determined by dibenzothiophenylation of arene and fluorination of aryl dibenzothiophenium salt. Generally, the selection of DBTO, B-DBTO, and M-DBTO could be predicted by evacuating the electron density of arenes. For arenes that are not easy to evacuate the electron density, B-DBTO will be used to test the arene density. If dibenzothiophenylation of arene does not work efficiently, more electron-poor DBTO will be tested. If dibenzothiophenylation of arene works efficiently, then fluorination of aryl dibenzothiophenium salt will be further tested. If the fluorination works well, then B-DBTO will be selected, otherwise M-DBTO will be further tested. Dibenzothiophenylation of arenes that are more electron-deficient than 1,2-dichlorobenzene cannot proceed efficiently and selectively.

Dibenzothiophenylation of arenes

Biphenyl-derived dibenzothiophenium salt S1

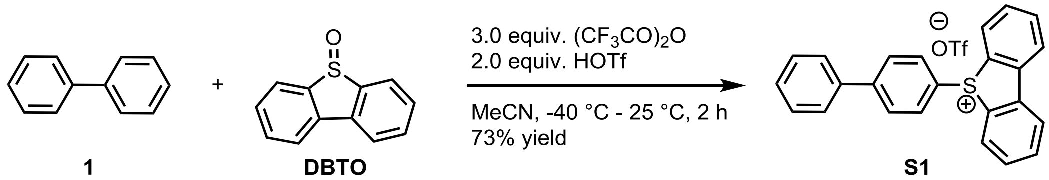

1 **DBTO** **S1**

A flame-dried, 10 mL nitrogen-filled *Schlenk*-tube equipped with a magnetic stir bar was charged with biphenyl (**1**) (77.1 mg, 0.500 mmol, 1.00 equiv.) and dry MeCN (2.0 mL, c = 0.25 M) at 25 °C. After cooling to –40 °C (acetonitrile/dry ice bath), trifluoromethanesulfonic acid (88.5 µL, 150 mg, 1.00 mmol, 2.00 equiv.) and trifluoroacetic anhydride (209 µL, 315 mg, 1.50 mmol, 3.00 equiv.) were added to the stirred reaction mixture. Subsequently, dibenzothiophene *S*-oxide (**DBTO**) (150 mg, 0.750 mmol, 1.50 equiv.) was added to the stirred

reaction mixture in small portions over 1 min. After addition, the reaction mixture was stirred at –40 °C for 1 h. Subsequently, the *Schlenk*-tube was taken out of the cold bath and warmed to 25 °C in air. After stirring at 25 °C for another 1 h, the reaction mixture was diluted with DCM (10 mL) and poured onto saturated aqueous $NaHCO_3$ (10 mL). The mixture was concentrated under reduced pressure to remove most of the MeCN solvent, and the residue was diluted with 20 mL DCM and 10 mL water. The mixture was poured into a separatory funnel, and the layers were separated. The DCM layer was collected, and the aqueous layer was further extracted with DCM (4 × ca. 30 mL). The combined DCM layer was dried over Na_2SO_4, filtered, and the solvent was removed under reduced pressure. The residue was purified by chromatography on silica gel eluting with DCM/MeOH (50:1 to 30:1, v/v). The product was collected and dried *in vacuo* to afford biphenyl-derived dibenzothiophenium salt **S1** (178 mg, 365 µmol, 73%) as a colorless solid.

R*f* = 0.48 (DCM/MeOH, 9:1, v/v).

NMR Spectroscopy:

^{1}H NMR (500 MHz, CD_3CN, 298 K, δ): 8.36 (d, *J* = 7.9 Hz, 2H), 8.15–8.09 (m, 2H), 7.95 (t, J = 7.7 Hz, 2H), 7.85–7.77 (m, 2H), 7.78–7.68 (m, 2H), 7.69–7.57 (m, 4H), 7.51–7.39 (m, 3H) ppm.

^{13}C NMR (126 MHz, CD_3CN, 298 K, δ): 148.5, 140.4, 138.9, 135.5, 133.1, 132.7, 132.1, 130.8, 130.2, 130.2, 128.9, 128.3, 126.0, 125.6, 122.2 (q, *J* = 321.2 Hz, CF_3) ppm.

^{19}F NMR (471 MHz, CD_3CN, 298 K, δ): –79.2 (s) ppm.

HRMS-ESI (m/z) calculated for $C_{24}H_{17}S^+$ $[M\text{-}OTf]^+$, 337.1045; found, 337.1041; deviation: 1.2 ppm.

1,2-Dichlorobenzene-derived dibenzothiophenium salt S2

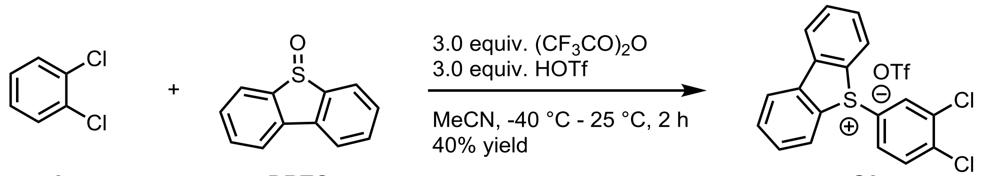

2 **DBTO** **S2**

A flame-dried, 10 mL nitrogen-filled *Schlenk*-tube equipped with a magnetic stir bar was charged with 1,2-dichlorobenzene (**2**) (73.5 mg, 0.500 mmol, 1.00 equiv.) and dry MeCN (2.0 mL, c = 0.25 M) at 25 °C. After cooling to –40 °C (acetonitrile/dry ice bath), trifluoromethanesulfonic acid (133 µL, 225 mg, 1.50 mmol, 3.00 equiv.) and trifluoroacetic anhydride (209 µL, 315 mg, 1.50 mmol, 3.00 equiv.) were added to the stirred reaction mixture. Subsequently, dibenzothiophene *S*-oxide (**DBTO**) (200 mg, 1.00 mmol, 2.00 equiv.) was added to the stirred reaction mixture in small portions over 1 min. After addition, the reaction mixture was stirred at –40 °C for 1 h. Subsequently, the *Schlenk*-tube was taken out of the cold bath and warmed to 25 °C in air. After stirring at 25 °C for another 1 h, the reaction mixture was diluted with DCM (10 mL) and poured onto saturated aqueous $NaHCO_3$ (10 mL). The mixture was concentrated under reduced pressure to remove most of the MeCN solvent, and the residue was diluted with 20 mL DCM and 10 mL water. The mixture was poured into a separatory funnel, and the layers were separated. The DCM layer was collected, and the aqueous layer was further extracted with DCM (4 × ca. 30 mL). The combined DCM layer was dried over

Na_2SO_4, filtered, and the solvent was removed under reduced pressure. The residue was purified by chromatography on silica gel eluting with DCM/MeOH (50:1 to 30:1, v/v) to afford a pale yellow solid. The solid was dissolved in 1.0 mL $CHCl_3$. A colorless solid precipitated after a few minutes, and the solvent was decanted. The solid was washed with 1.0 mL $CHCl_3$ and dried *in vacuo* to afford 1,2-dichlorobenzene-derived dibenzothiophenium salt **S2** (95.5 mg, 199 µmol, 40%) as a colorless solid.

R*f* = 0.43 (DCM/MeOH, 9:1, v/v).

NMR Spectroscopy:

^{1}H NMR (500 MHz, CD_3CN, 298 K, δ): 8.35 (d, *J* = 7.6 Hz, 2H), 8.12 (d, *J* = 8.1 Hz, 2H), 7.96 (t, *J* = 7.7 Hz, 2H), 7.85 (d, *J* = 2.4 Hz, 1H), 7.78–7.66 (m, 3H), 7.41 (dd, J = 8.9, 2.4 Hz, 1H) ppm.

^{13}C NMR (126 MHz, CD_3CN, 298 K, δ): 140.6, 140.6, 135.8, 135.8, 134.3, 133.4, 132.8, 132.2, 130.8, 129.2, 127.6, 125.8, 122.1 (q, *J* = 320.8 Hz, CF_3) ppm.

^{19}F NMR (471 MHz, CD_3CN, 298 K, δ): –79.2 (s) ppm.

HRMS-ESI (m/z) calculated for $C_{18}H_{11}SCl_2^+$ $[M-OTf]^+$, 328.9953; found, 328.9949; deviation: 1.4 ppm.

2-Fluoro-6-phenoxybenzonitrile-derived dibenzothiophenium salt S3

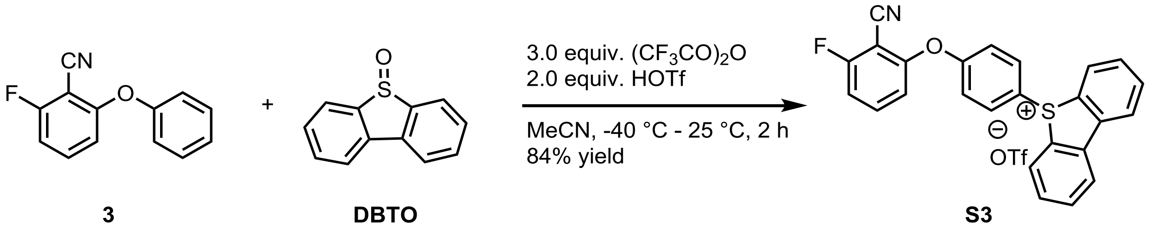

3 **DBTO** **S3**

A flame-dried, 10 mL nitrogen-filled *Schlenk*-tube equipped with a magnetic stir bar was charged with 2-fluoro-6-phenoxybenzonitrile (**3**) (102 mg, 0.500 mmol, 1.00 equiv.) and dry MeCN (2.0 mL, c = 0.25 M) at 25 °C. After cooling to –40 °C (acetonitrile/dry ice bath), trifluoromethanesulfonic acid (88.5 µL, 150 mg, 1.00 mmol, 2.00 equiv.) and trifluoroacetic anhydride (209 µL, 315 mg, 1.50 mmol, 3.00 equiv.) were added to the stirred reaction mixture. Subsequently, dibenzothiophene *S*-oxide (**DBTO**) (150 mg, 0.750 mmol, 1.50 equiv.) was added to the stirred reaction mixture in small portions over 1 min. After addition, the reaction mixture was stirred at –40 °C for 1 h. Subsequently, the *Schlenk*-tube was taken out of the cold bath and warmed to 25 °C in air. After stirring at 25 °C for another 1 h, the reaction mixture was diluted with DCM (10 mL) and poured onto saturated aqueous $NaHCO_3$ (10 mL). The mixture was concentrated under reduced pressure to remove most of the MeCN solvent, and the residue was diluted with 20 mL DCM and 10 mL water. The mixture was poured into a separatory funnel, and the layers were separated. The DCM layer was collected, and the aqueous layer was further extracted with DCM (4 × ca. 30 mL). The combined DCM layer was dried over Na_2SO_4, filtered, and the solvent was removed under reduced pressure. The residue was purified by chromatography on silica gel eluting with DCM/MeOH (50:1 to 30:1, v/v). The product was collected and dried *in vacuo* to afford 2-fluoro-6-phenoxybenzonitrile-derived dibenzothiophenium salt **S3** (230 mg, 422 µmol, 84%) as a colorless solid.

R*f* = 0.48 (DCM/MeOH, 9:1, v/v).

NMR Spectroscopy:

^{1}H NMR (500 MHz, CD_3CN, 298 K, δ): 8.34 (d, *J* = 7.9 Hz, 2H), 8.11 (d, *J* = 8.1 Hz, 2H), 7.94 (t, *J* = 7.7 Hz, 2H), 7.77–7.61 (m, 5H), 7.28–7.21 (m, 2H), 7.18 (t, J = 8.7 Hz, 1H), 6.93 (d, J = 8.5 Hz, 1H) ppm.

^{13}C NMR (126 MHz, CD_3CN, 298 K, δ): 164.8 (d, *J* = 257.6 Hz), 161.8, 158.4 (d, *J* = 3.6 Hz), 140.3, 137.4 (d, *J* = 10.3 Hz), 135.5, 134.4, 133.0, 132.6, 129.0, 125.6, 122.3, 122.1 (q, *J* = 321.3 Hz, CF_3), 122.1, 116.6 (d, *J* = 3.4 Hz), 113.4 (d, *J* = 19.5 Hz), 111.6, 96.1 (d, *J* = 18.4 Hz) ppm.

^{19}F NMR (471 MHz, CD_3CN, 298 K, δ): –79.2 (s), –106.6 (dd, *J* = 8.9, 6.6 Hz) ppm.

HRMS-ESI (m/z) calculated for $C_{25}H_{15}NOSF^+$ [M-OTf]$^+$, 396.0853; found, 396.0850; deviation: 0.7 ppm.

Diflunisal derivative-derived dibenzothiophenium salt S4

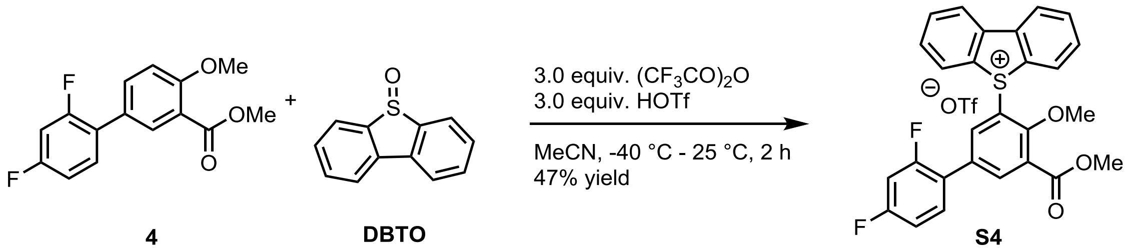

4 **DBTO** **S4**

A flame-dried, 10 mL nitrogen-filled *Schlenk*-tube equipped with a magnetic stir bar was charged with diflunisal derivative **4** (139 mg, 0.500 mmol, 1.00 equiv.) and dry MeCN (2.0 mL, c = 0.25 M) at 25 °C. After cooling to –40 °C (acetonitrile/dry ice bath), trifluoromethanesulfonic acid (133 µL, 225 mg, 1.50 mmol, 3.00 equiv.) and trifluoroacetic anhydride (209 µL, 315 mg, 1.50 mmol, 3.00 equiv.) were added to the stirred reaction mixture. Subsequently, dibenzothiophene *S*-oxide (**DBTO**) (200 mg, 1.00 mmol, 2.00 equiv.) was added to the stirred reaction mixture in small portions over 1 min. After addition, the reaction mixture was stirred at –40 °C for 1 h. Subsequently, the *Schlenk*-tube was taken out of the cold bath and warmed to 25 °C in air. After stirring at 25 °C for another 1 h, the reaction mixture was diluted with DCM (10 mL) and poured onto saturated aqueous $NaHCO_3$ (10 mL). The mixture was concentrated under reduced pressure to remove most of the MeCN solvent, and the residue was diluted with 20 mL DCM and 10 mL water. The mixture was poured into a separatory funnel, and the layers were separated. The DCM layer was collected, and the aqueous layer was further extracted with DCM (4 × ca. 30 mL). The combined DCM layer was dried over Na_2SO_4, filtered, and the solvent was removed under reduced pressure. The residue was purified by chromatography on silica gel eluting with DCM/MeOH (50:1 to 30:1, v/v). The product was collected and dried *in vacuo* to afford diflunisal derivative-derived dibenzothiophenium salt **S4** (143 mg, 234 µmol, 47%) as a colorless solid.

R*f* = 0.45 (DCM/MeOH, 9:1, v/v).

NMR Spectroscopy:

^{1}H NMR (500 MHz, CD_3CN, 298 K, δ): 8.33 (d, *J* = 7.9 Hz, 2H), 8.26–8.19 (m, 3H), 7.93 (t, *J* = 7.7 Hz,

2H), 7.79–7.71 (m, 2H), 7.44 (d, *J* = 2.3 Hz, 1H), 7.40 (d, *J* = 8.4 Hz, 1H), 7.05–6.98 (m, 2H), 3.96 (s, 3H), 3.93 (s, 3H) ppm.

^{13}C NMR (126 MHz, CD_3CN, 298 K, δ): 165.1, 164.0 (dd, *J* = 249.3, 12.3 Hz). 160.4 (dd, *J* = 250.1, 12.3 Hz). 159.8, 140.9, 139.6 (d, *J* = 3.1 Hz), 135.6, 134.2, 133.0, 132.7 (dd, *J* = 10.0, 4.2 Hz), 132.6, 130.4, 129.4, 126.7, 125.7, 123.2, 122.5 (dd, *J* = 13.1, 3.9 Hz), 122.1 (q, *J* = 321.4 Hz, CF_3), 113.2 (dd, *J* = 21.6, 3.7 Hz), 105.4 (t, *J* = 26.3 Hz), 64.7, 53.9 ppm.

^{19}F NMR (471 MHz, CD_3CN, 298 K, δ): –79.2 (s), –110.3 (d, *J* = 8.6 Hz), –115.2 (d, *J* = 8.0 Hz) ppm.

HRMS-ESI (m/z) calculated for $C_{27}H_{19}O_3SF_2^+$ [M-OTf]$^+$, 461.1017; found, 461.1011; deviation: –1.2 ppm.

rac-Propafenone derivative-derived 3,7-di-*tert*-butyldibenzothiophenium salt S5

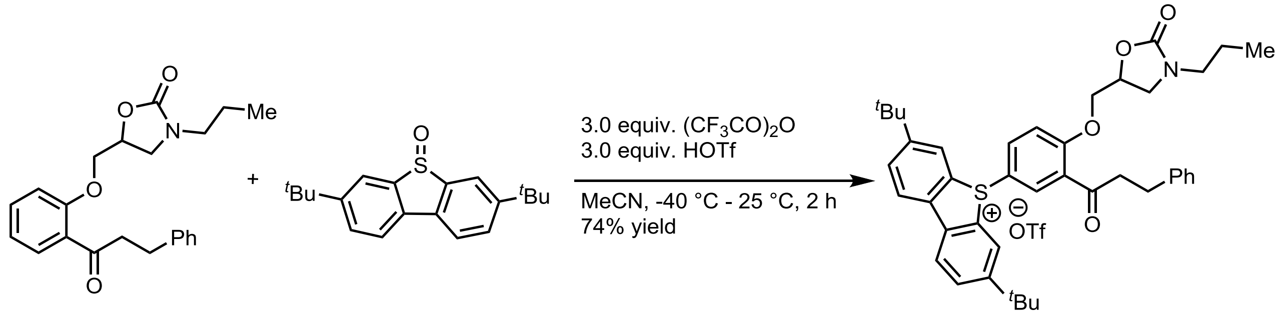

5 **B-DBTO** **S5**

A flame-dried, 10 mL nitrogen-filled *Schlenk*-tube equipped with a magnetic stir bar was charged with *rac*-propafenone derivative **5** (184 mg, 0.500 mmol, 1.00 equiv.), and dry MeCN (2.0 mL, c = 0.25 M) at 25 °C. After cooling to –40 °C (acetonitrile/dry ice bath), trifluoromethanesulfonic acid (133 µL, 225 mg, 1.50 mmol, 3.00 equiv.) and trifluoroacetic anhydride (209 µL, 315 mg, 1.50 mmol, 3.00 equiv.) were added to the stirred reaction mixture. Subsequently, 3,7-di-*tert*-butyldibenzothiophene *S*-oxide (**B-DBTO**) (234 mg, 0.750 mmol, 1.50 equiv.) was added to the stirred reaction mixture in small portions over 1 min. After addition, the reaction mixture was stirred at –40 °C for 1 h. Subsequently, the *Schlenk*-tube was taken out of the cold bath and warmed to 25 °C in air. After stirring at 25 °C for another 1 h, the reaction mixture was diluted with DCM (10 mL) and poured onto saturated aqueous $NaHCO_3$ (10 mL). The mixture was concentrated under reduced pressure to remove most of the MeCN solvent, and the residue was diluted with 20 mL DCM and 10 mL water. The mixture was poured into a separatory funnel, and the layers were separated. The DCM layer was collected, and the aqueous layer was further extracted with DCM (4 × ca. 30 mL). The combined DCM layer was dried over Na_2SO_4, filtered, and the solvent was removed under reduced pressure. The residue was purified by chromatography on silica gel eluting with DCM/MeOH (40:1 to 30:1, v/v) to afford an orange solid. The solid was dissolved in 1.0 mL DCM, and precipitated with 15.0 mL hexanes. The suspension was decanted, and the solid was dried *in vacuo* to afford *rac*-propafenone derivative-derived 3,7-di-*tert*-butyldibenzothiophenium salt **S5** (299 mg, 368 µmol, 74%) as a pale orange solid.

R*f* = 0.52 (DCM/MeOH, 9:1, v/v).

NMR Spectroscopy:

^{1}H NMR (500 MHz, CD_3CN, 298 K, δ): 8.22 (d, *J* = 8.3 Hz, 2H), 8.15 (s, 2H), 7.97 (dd, *J* = 8.3, 1.8 Hz, 2H), 7.81 (d, *J* = 2.7 Hz, 1H), 7.62 (dd, *J* = 9.1, 2.7 Hz, 1H), 7.26 (d, *J* = 9.1 Hz, 1H), 7.21–7.11 (m, 4H), 7.14–7.06 (m, 1H), 4.87–4.82 (m, 1H), 4.33–4.26 (m, 2H), 3.65 (t, *J* = 9.2 Hz, 1H), 3.37 (dd, *J* = 9.0, 6.5 Hz, 1H), 3.22 (t, *J* = 7.3 Hz, 2H), 3.17–3.10 (m, 1H), 3.08–2.99 (m, 1H), 2.92–2.84 (m, 2H), 1.54–1.43 (m, 2H), 1.32 (s, 18H), 0.85 (t, *J* = 7.4 Hz, 3H) ppm.

^{13}C NMR (126 MHz, CD_3CN, 298 K, δ): 199.7, 162.6, 158.1, 156.5, 142.0, 137.5, 136.4, 133.9, 133.5, 132.8, 131.6, 129.3, 129.2, 126.9, 125.8, 124.9, 122.1 (q, *J* = 321.2 Hz, CF_3), 118.6, 117.3, 71.4, 70.9, 46.4, 46.3, 45.5, 36.4, 31.3, 30.4, 21.2, 11.4 ppm.

^{19}F NMR (471 MHz, CD_3CN, 298 K, δ): –79.1 (s) ppm.

HRMS-ESI (m/z) calculated for $C_{42}H_{48}NO_4S^+$ $[M\text{-}OTf]^+$, 662.32986; found, 662.3294; deviation: 0.7 ppm.

Toluene-derived dibenzothiophenium salt S6

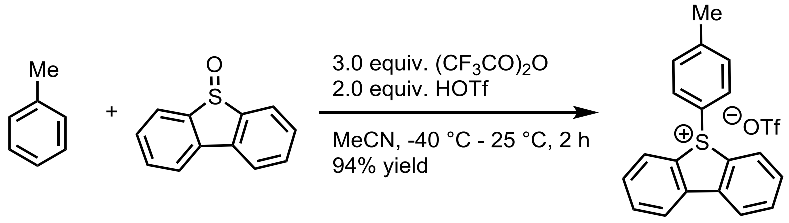

6 **DBTO** **S6**

A flame-dried, 10 mL nitrogen-filled *Schlenk*-tube equipped with a magnetic stir bar was charged with toluene (**6**) (46.1 mg, 0.500 mmol, 1.00 equiv.) and dry MeCN (2.0 mL, c = 0.25 M) at 25 °C. After cooling to –40 °C (acetonitrile/dry ice bath), trifluoromethanesulfonic acid (88.5 µL, 150 mg, 1.00 mmol, 2.00 equiv.) and trifluoroacetic anhydride (209 µL, 315 mg, 1.50 mmol, 3.00 equiv.) were added to the stirred reaction mixture. Subsequently, dibenzothiophene *S*-oxide (**DBTO**) (150 mg, 0.750 mmol, 1.50 equiv.) was added to the stirred reaction mixture in small portions over 1 min. After addition, the reaction mixture was stirred at –40 °C for 1 h. Subsequently, the *Schlenk*-tube was taken out of the cold bath and warmed to 25 °C in air. After stirring at 25 °C for another 1 h, the reaction mixture was diluted with DCM (10 mL) and poured onto saturated aqueous $NaHCO_3$ (10 mL). The mixture was concentrated under reduced pressure to remove most of the MeCN solvent, and the residue was diluted with 20 mL DCM and 10 mL water. The mixture was poured into a separatory funnel, and the layers were separated. The DCM layer was collected, and the aqueous layer was further extracted with DCM (4 × ca. 30 mL). The combined DCM layer was dried over Na_2SO_4, filtered, and the solvent was removed under reduced pressure. The residue was purified by chromatography on silica gel eluting with DCM/MeOH (50:1 to 30:1, v/v). The product was collected and dried *in vacuo* to afford toluene-derived dibenzothiophenium salt **S6** (200 mg, 471 µmol, 94%) as a colorless solid.

R*f* = 0.50 (DCM/MeOH, 9:1, v/v).

NMR Spectroscopy:

^{1}H NMR (500 MHz, CD_3CN, 298 K, δ): 8.41–8.31 (m, 2H), 8.14–8.03 (m, 2H), 7.96–7.92 (m, 2H), 7.73–

7.70 (m, 2H), 7.49–7.42 (m, 2H), 7.41–7.39 (m, 2H), 2.38 (s, 3H) ppm.

^{13}C NMR (126 MHz, CD_3CN, 298 K, δ): 148.1, 140.3, 135.4, 133.3, 133.2, 132.6, 131.5, 128.8, 125.5, 123.9, 122.2 (q, *J* = 320.8 Hz, CF_3), 21.6 ppm.

^{19}F NMR (471 MHz, CD_3CN, 298 K, δ): –79.3 (s) ppm.

HRMS-ESI (m/z) calculated for $C_{19}H_{15}S^+$ [M-OTf]$^+$, 275.0889; found, 275.0887; deviation: 0.9 ppm.

o-Anisaldehyde-derived 3,7-di-*tert*-butyldibenzothiophenium salt S7

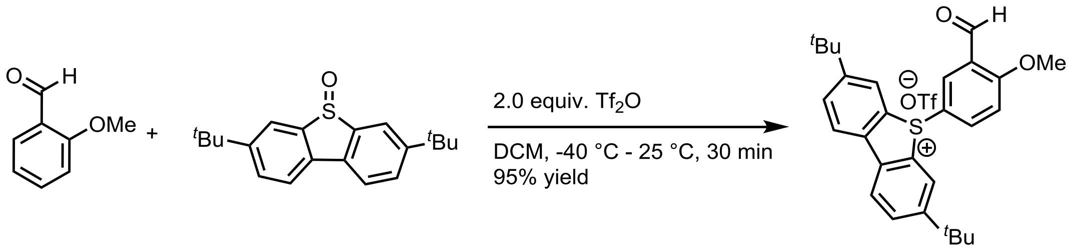

7 **B-DBTO** **S7**

A flame-dried, 10 mL nitrogen-filled *Schlenk*-tube equipped with a magnetic stir bar was charged with *o*-anisaldehyde (**7**) (68.1 mg, 0.500 mmol, 1.00 equiv.), 3,7-di-*tert*-butyldibenzothiophene *S*-oxide (**B-DBTO**) (234 mg, 0.750 mmol, 1.50 equiv.), and dry DCM (2.0 mL, c = 0.25 M) at 25 °C. After cooling to –40 °C (acetonitrile/dry ice bath), trifluoromethanesulfonic anhydride (168 µL, 282 mg, 1.00 mmol, 2.00 equiv.) was added dropwise over 1 min. The *Schlenk*-tube was sealed, taken out of the cold bath and warmed to 25 °C in air. After stirring at 25 °C for 30 min, the reaction mixture was diluted with DCM (5.0 mL) and poured onto a mixture of DCM (20 mL) and saturated aqueous $NaHCO_3$ (5.0 mL). The mixture was poured into a separatory funnel, and the layers were separated. The DCM layer was collected, and the aqueous layer was further extracted with DCM (4 × ca. 30 mL). The combined DCM layer was dried over Na_2SO_4, filtered, and the solvent was removed under reduced pressure. The residue was purified by chromatography on silica gel eluting with DCM/MeOH (30:1, v/v) to afford a pale orange solid. The solid was suspended in Et_2O (3.0 mL), and hexanes (17.0 mL) was added. The solvent was decanted, and the solid was dried *in vacuo* to afford *o*-anisaldehyde-derived 3,7-di-*tert*-butyldibenzothiophenium salt **S7** (277 mg, 477 µmol, 95 %) as a pale orange solid.

R*f* = 0.45 (DCM/MeOH, 10:1, v/v).

NMR Spectroscopy:

^{1}H NMR (500 MHz, CD_3CN, 298 K, δ): 10.25 (s, 1H), 8.23 (d, *J* = 8.3 Hz, 2H), 8.15 (s, 2H), 8.00–7.95 (m, 3H), 7.68–7.65 (m, 1H), 7.32 (d, J = 9.1 Hz, 1H), 3.96 (s, 3H), 1.32 (s, 18H) ppm.

^{13}C NMR (126 MHz, CD_3CN, 298 K, δ): 188.2, 166.9, 156.6, 138.5, 137.5, 133.5, 132.9, 132.6, 127.2, 125.9, 124.9, 122.1 (q, *J* = 321.0 Hz, CF_3), 118.4, 117.2, 57.9, 36.5, 31.2 ppm.

^{19}F NMR (471 MHz, CD_3CN, 298 K, δ): –79.0 (s) ppm.

HRMS-ESI (m/z) calculated for $C_{28}H_{31}O_2S^+$ [M-OTf]$^+$, 431.2039; found, 431.2040; deviation: –0.2 ppm.

(*R*)-(−)-4-Benzyl-3-propionyl-2-oxazolidinone-derived dibenzothiophenium salt S8

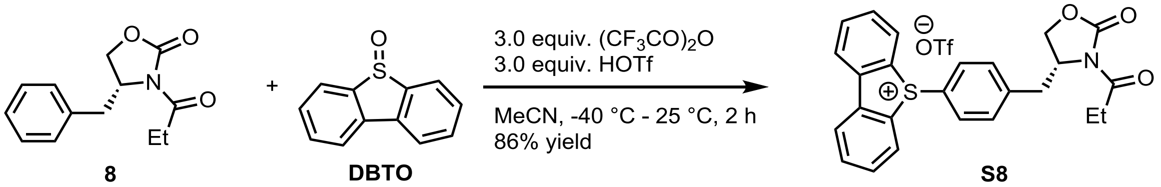

A flame-dried, 10 mL nitrogen-filled *Schlenk*-tube equipped with a magnetic stir bar was charged with (*R*)-(−)-4-benzyl-3-propionyl-2-oxazolidinone (**8**) (117 mg, 0.500 mmol, 1.00 equiv.) and dry MeCN (2.0 mL, c = 0.25 M) at 25 °C. After cooling to –40 °C (acetonitrile/dry ice bath), trifluoromethanesulfonic acid (133 µL, 225 mg, 1.50 mmol, 3.00 equiv.) and trifluoroacetic anhydride (209 µL, 315 mg, 1.50 mmol, 3.00 equiv.) were added to the stirred reaction mixture. Subsequently, dibenzothiophene *S*-oxide (**DBTO**) (150 mg, 0.750 mmol, 1.50 equiv.) was added to the stirred reaction mixture in small portions over 1 min. After addition, the reaction mixture was stirred at –40 °C for 1 h. Subsequently, the *Schlenk*-tube was taken out of the cold bath and warmed to 25 °C in air. After stirring at 25 °C for another 1 h, the reaction mixture was diluted with DCM (10 mL) and poured onto saturated aqueous $NaHCO_3$ (10 mL). The mixture was concentrated under reduced pressure to remove most of the MeCN solvent, and the residue was diluted with 20 mL DCM and 10 mL water. The mixture was poured into a separatory funnel, and the layers were separated. The DCM layer was collected, and the aqueous layer was further extracted with DCM (4 × ca. 30 mL). The combined DCM layer was dried over Na_2SO_4, filtered, and the solvent was removed under reduced pressure. The residue was purified by chromatography on silica gel eluting with DCM/MeOH (30:1, v/v). The product was collected and dried *in vacuo* to afford (*R*)-(−)-4-benzyl-3-propionyl-2-oxazolidinone-derived dibenzothiophenium salt **S8** (244 mg, 431 µmol, 86%) as a colorless solid.

R*f* = 0.50 (DCM/MeOH, 9:1, v/v).

NMR Spectroscopy:

^{1}H NMR (500 MHz, CD_3CN, 298 K, δ): 8.35 (d, *J* = 7.8 Hz, 2H), 8.06 (d, *J* = 8.1 Hz, 2H), 7.95 (*t*, J = 7.7 Hz, 2H), 7.79–7.69 (m, 2H), 7.51 (d, *J* = 8.5 Hz, 2H), 7.45–7.39 (m, 2H), 4.69–4.61 (m, 1H), 4.25 (t, *J* = 8.6 Hz, 1H), 4.07–4.05 (m, 1H), 3.13–3.10 (m, 1H), 3.06–3.01 (m, 1H), 2.85–2.71 (m, 2H), 1.03 (t, J = 7.4 Hz, 3H) ppm.

^{13}C NMR (126 MHz, CD_3CN, 298 K, δ): 174.7, 154.5, 145.6, 140.4, 140.4, 135.5, 133.7, 133.0, 133.0, 132.7, 131.6, 128.9, 126.1, 125.6, 122.1 (q, *J* = 321.5 Hz, CF_3), 67.4, 55.2, 38.3, 29.6, 8.7 ppm.

^{19}F NMR (471 MHz, CD_3CN, 298 K, δ): –79.3 (s) ppm.

HRMS-ESI (m/z) calculated for $C_{25}H_{22}NO_3S^+$ $[M\text{-}OTf]^+$, 416.1315; found, 416.1313; deviation: 0.4 ppm.

3-Chloro-6-methyldibenzo[*c*,*f*][1,2]thiazepin-11(6*H*)-one 5,5-dioxide-derived dibenzothiophenium salt S9

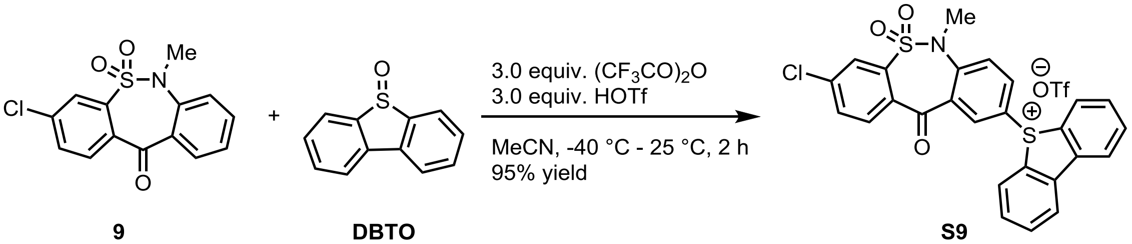

9 **DBTO** **S9**

A flame-dried, 10 mL nitrogen-filled *Schlenk*-tube equipped with a magnetic stir bar was charged with 6-methyldibenzo[*c*,*f*][1,2]thiazepin-11(6*H*)-one 5,5-dioxide (**9**) (154 mg, 0.500 mmol, 1.00 equiv.) and dry MeCN (2.0 mL, c = 0.25 M) at 25 °C. After cooling to –40 °C (acetonitrile/dry ice bath), trifluoromethanesulfonic acid (133 μL, 225 mg, 1.50 mmol, 3.00 equiv.) and trifluoroacetic anhydride (209 μL, 315 mg, 1.50 mmol, 3.00 equiv.) were added to the stirred reaction mixture. Subsequently, dibenzothiophene *S*-oxide (**DBTO**) (150 mg, 0.750 mmol, 1.50 equiv.) was added to the stirred reaction mixture in small portions over 1 min. After addition, the reaction mixture was stirred at –40 °C for 1 h. Subsequently, the *Schlenk*-tube was taken out of the cold bath and warmed to 25 °C in air. After stirring at 25 °C for another 1 h, the reaction mixture was diluted with DCM (10 mL) and poured onto saturated aqueous $NaHCO_3$ (10 mL). The mixture was concentrated under reduced pressure to remove most of the MeCN solvent, and the residue was diluted with 20 mL DCM and 10 mL water. The mixture was poured into a separatory funnel, and the layers were separated. The DCM layer was collected, and the aqueous layer was further extracted with DCM (4 × ca. 30 mL). The combined DCM layer was dried over Na_2SO_4, filtered, and the solvent was removed under reduced pressure. The residue was purified by chromatography on silica gel eluting with DCM/MeOH (30:1, v/v) to afford a colorless solid. The solid was washed with 5 mL Et_2O and dried *in vacuo* to afford 3-chloro-6-methyldibenzo[*c*,*f*][1,2]thiazepin-11(6*H*)-one 5,5-dioxide-derived dibenzothiophenium salt **S9** (305 mg, 477 μmol, 95%) as a colorless solid.

R*f* = 0.37 (DCM/MeOH, 9:1, v/v).

NMR Spectroscopy:

^{1}H NMR (500 MHz, CD_3CN, 298 K, δ): 8.62 (d, *J* = 2.4 Hz, 1H), 8.36 (d, *J* = 7.9 Hz, 2H), 8.17–8.11 (m, 2H), 7.95 (td, *J* = 7.6, 1.1 Hz, 2H), 7.88 (d, *J* = 1.8 Hz, 1H), 7.84–7.75 (m, 2H), 7.71 (td, *J* = 7.7, 1.2 Hz, 2H), 7.60–7.51 (m, 2H), 3.38 (s, 3H) ppm.

^{13}C NMR (126 MHz, CD_3CN, 298 K, δ): 189.7, 147.4, 140.5, 139.6, 138.6, 136.3, 135.7, 135.6, 135.3, 134.8, 133.8, 132.8, 132.6, 132.0, 129.1, 127.5, 125.7, 125.6, 123.5, 122.1 (q, *J* = 320.7 Hz, CF_3), 39.0 ppm.

^{19}F NMR (471 MHz, CD_3CN, 298 K, δ): –79.1 (s) ppm.

HRMS-ESI (m/z) calculated for $C_{26}H_{17}NO_3S_2Cl^+$ $[M-OTf]^+$, 490.0333; found, 490.0329; deviation: 0.8 ppm.

Fenofibrate-derived dibenzothiophenium salt S10

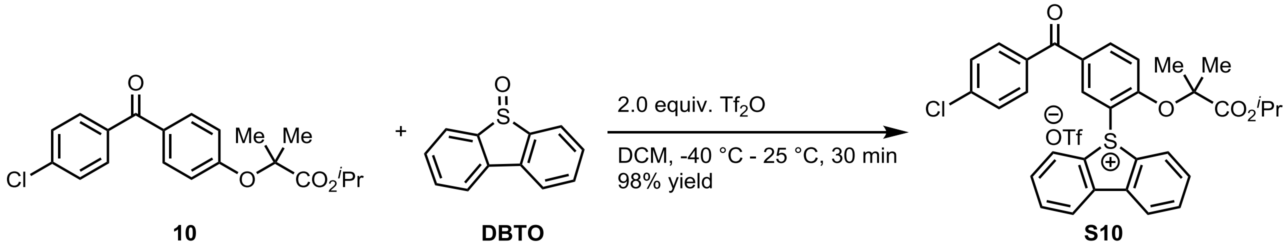

A flame-dried, 10 mL nitrogen-filled *Schlenk*-tube equipped with a magnetic stir bar was charged with fenofibrate (**10**) (180 mg, 0.500 mmol, 1.00 equiv.), dibenzothiophene *S*-oxide (**DBTO**) (150 mg, 0.750 mmol, 1.50 equiv.), and dry DCM (2.0 mL, c = 0.25 M) at 25 °C. After cooling to –40 °C (acetonitrile/dry ice bath), trifluoromethanesulfonic anhydride (168 µL, 282 mg, 1.00 mmol, 2.00 equiv.) was added dropwise over 1 min. The *Schlenk*-tube was sealed, taken out of the cold bath and warmed to 25 °C in air. After stirring at 25 °C for 30 min, the reaction mixture was diluted with DCM (5.0 mL) and poured onto a mixture of DCM (20 mL) and saturated aqueous $NaHCO_3$ (5.0 mL). The mixture was poured into a separatory funnel, and the layers were separated. The DCM layer was collected, and the aqueous layer was further extracted with DCM (4 × ca. 30 mL). The combined DCM layer was dried over Na_2SO_4, filtered, and the solvent was removed under reduced pressure. The residue was purified by chromatography on silica gel eluting with DCM/MeOH (40:1 to 30:1, v/v). The product was collected and dried *in vacuo* to afford fenofibrate-derived dibenzothiophenium salt **S10** (340 mg, 491 µmol, 98 %) as a colorless solid.

R*f* = 0.15 (DCM/MeOH, 20:1, v/v).

NMR Spectroscopy:

^{1}H NMR (500 MHz, CD_3CN, 298 K, δ): 8.31 (dd, *J* = 7.9, 1.1 Hz, 2H), 8.17 (d, *J* = 8.1 Hz, 2H), 8.06 (dd, *J* = 8.8, 2.2 Hz, 1H), 8.01–7.86 (m, 3H), 7.76 (td, *J* = 7.8, 1.2 Hz, 2H), 7.66 (d, *J* = 8.2 Hz, 2H), 7.58–7.48 (m, 2H), 6.97 (d, *J* = 8.8 Hz, 1H), 4.98–4.91 (m, 1H), 1.40 (brs, 6H), 1.09 (d, *J* = 6.3 Hz, 6H) ppm.

^{13}C NMR (126 MHz, CD_3CN, 298 K, δ): 192.5, 171.7, 159.3, 141.0, 139.6, 139.2, 136.1, 135.3, 132.4, 132.3, 132.1, 130.2, 129.8, 128.8, 125.4, 122.1 (q, *J* = 321.5 Hz, CF_3), 117.8, 83.7, 71.1, 25.0, 21.7 ppm.

^{19}F NMR (471 MHz, CD_3CN, 298 K, δ): –79.2 (s) ppm.

HRMS-ESI (m/z) calculated for $C_{32}H_{28}O_4SCl^+$ $[M\text{-}OTf]^+$, 543.1391; found, 543.1387; deviation: 0.7 ppm.

Cyclopropylbenzene-derived 3,7-di-*tert*-butyldibenzothiophenium salt S11

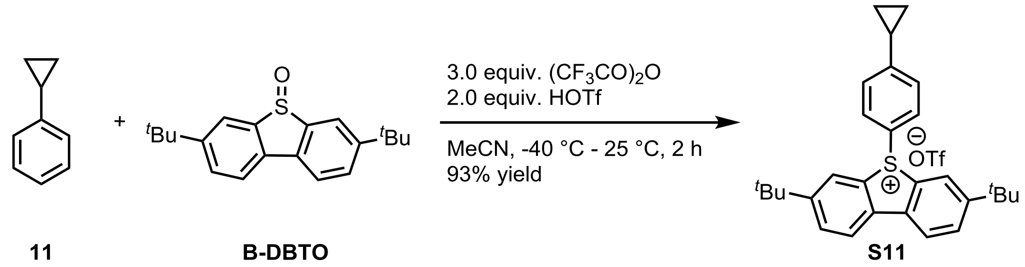

A flame-dried, 10 mL nitrogen-filled *Schlenk*-tube equipped with a magnetic stir bar was charged with cyclopropylbenzene (**11**) (59.0 mg, 0.500 mmol, 1.00 equiv.), and dry MeCN (2.0 mL, c = 0.25 M) at 25 °C. After cooling to –40 °C (acetonitrile/dry ice bath), trifluoromethanesulfonic acid (88.5 µL, 150 mg, 1.00 mmol, 2.00 equiv.) and trifluoroacetic anhydride (209 µL, 315 mg, 1.50 mmol, 3.00 equiv.) were added to the stirred reaction mixture. Subsequently, 3,7-di-*tert*-butyldibenzothiophene *S*-oxide (**B-DBTO**) (234 mg, 0.750 mmol, 1.50 equiv.) was added to the stirred reaction mixture in small portions over 1 min. After addition, the reaction mixture was stirred at –40 °C for 1 h. Subsequently, the *Schlenk*-tube was taken out of the cold bath and warmed to 25 °C in air. After stirring at 25 °C for another 1 h, the reaction mixture was diluted with DCM (10 mL) and poured onto saturated aqueous $NaHCO_3$ (10 mL). The mixture was concentrated under reduced pressure to remove most of the MeCN solvent, and the residue was diluted with 20 mL DCM and 10 mL water. The mixture was poured into a separatory funnel, and the layers were separated. The DCM layer was collected, and the aqueous layer was further extracted with DCM (4 × ca. 30 mL). The combined DCM layer was dried over Na_2SO_4, filtered, and the solvent was removed under reduced pressure. The residue was purified by chromatography on silica gel eluting with DCM/MeOH (30:1, v/v) to afford a solid. The solid was washed with 5 mL Et_2O and dried *in vacuo* to afford cyclopropylbenzene-derived 3,7-di-*tert*-butyldibenzothiophenium salt **S11** (260 mg, 463 µmol, 93%) as an pale orange solid.

R*f* = 0.50 (DCM/MeOH, 9:1, v/v).

NMR Spectroscopy:

^{1}H NMR (500 MHz, CD_3CN, 298 K, δ): 8.23–8.18 (m, 2H), 8.08–8.04 (m, 2H), 8.08–8.04 (m, 2H), 7.98–7.96 (m, 2H), 7.28–7.22 (m, 2H), 2.00–1.95 (m, 1H), 1.33 (s, 18H), 1.14–1.05 (m, 2H), 0.80–0.72 (m, 2H) ppm.

^{13}C NMR (126 MHz, CD_3CN, 298 K, δ): 156.6, 154.5, 137.6, 133.6, 132.8, 131.6, 129.2, 125.6, 124.8, 123.3, 122.2 (q, J = 321.0 Hz, CF_3), 36.5, 31.2, 16.3, 11.9 ppm.

^{19}F NMR (471 MHz, CD_3CN, 298 K, δ): –79.3 (s) ppm.

HRMS-ESI (m/z) calculated for $C_{29}H_{33}S^+$ [M-OTf]$^+$, 413.2297; found, 413.2296; deviation: 0.5 ppm.

6-Methoxyquinoline-derived dibenzothiophenium salt S12

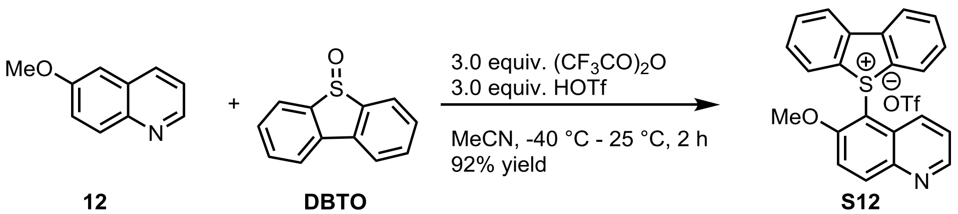

12 **DBTO** **S12**

A flame-dried, 10 mL nitrogen-filled *Schlenk*-tube equipped with a magnetic stir bar was charged with 6-methoxyquinoline (**12**) (79.6 mg, 0.500 mmol, 1.00 equiv.) and dry MeCN (2.0 mL, c = 0.25 M) at 25 °C. After cooling to –40 °C (acetonitrile/dry ice bath), trifluoromethanesulfonic acid (133 µL, 225 mg, 1.50 mmol, 3.00 equiv.) and trifluoroacetic anhydride (209 µL, 315 mg, 1.50 mmol, 3.00 equiv.) were added to the stirred

reaction mixture. Subsequently, dibenzothiophene *S*-oxide (**DBTO**) (150 mg, 0.750 mmol, 1.50 equiv.) was added to the stirred reaction mixture in small portions over 1 min. After addition, the reaction mixture was stirred at –40 °C for 1 h. Subsequently, the *Schlenk*-tube was taken out of the cold bath and warmed to 25 °C in air. After stirring at 25 °C for another 1 h, the reaction mixture was diluted with DCM (10 mL) and poured onto saturated aqueous $NaHCO_3$ (10 mL). The mixture was concentrated under reduced pressure to remove most of the MeCN solvent, and the residue was diluted with 20 mL DCM and 10 mL water. The mixture was poured into a separatory funnel, and the layers were separated. The DCM layer was collected, and the aqueous layer was further extracted with DCM (4 × ca. 30 mL). The combined DCM layer was dried over Na_2SO_4, filtered, and the solvent was removed under reduced pressure. The residue was purified by chromatography on silica gel eluting with DCM/MeOH (30:1 to 20:1, v/v) to afford a colorless solid. The solid was washed twice with 1.0 mL DCM/Et_2O (4:1, v/v) and dried *in vacuo* to afford 6-methoxyquinoline-derived dibenzothiophenium salt **S12** (226 mg, 460 µmol, 92%) as a colorless solid.

R*f* = 0.43 (DCM/MeOH, 9:1, v/v).

NMR Spectroscopy:

^{1}H NMR (500 MHz, CD_3CN, 298 K, δ): 9.17 (d, *J* = 8.7 Hz, 1H), 9.06–9.01 (m, 1H), 8.50 (d, *J* = 9.4 Hz, 1H), 8.37 (d, *J* = 7.9 Hz, 2H), 7.96–7.85 (m, 5H), 7.67 (t, *J* = 7.8 Hz, 2H), 7.55 (d, *J* = 9.4 Hz, 1H), 3.28 (s, 3H) ppm.

^{13}C NMR (126 MHz, CD_3CN, 298 K, δ): 161.2, 151.2, 144.7, 141.6, 141.2, 134.6, 132.1, 132.0, 131.9, 130.1, 127.8, 125.3, 124.8, 122.1 (q, *J* = 321.0 Hz, CF_3), 118.9, 101.9, 57.8 ppm.

^{19}F NMR (471 MHz, CD_3CN, 298 K, δ): –79.3 (s) ppm.

HRMS-ESI (m/z) calculated for $C_{22}H_{16}NOS^+$ $[M\text{-}OTf]^+$, 342.0947; found, 342.0945; deviation: 0.7 ppm.

1-Chloro-3-phenylpropane-derived 3,7-di-*tert*-butyldibenzothiophenium salt S13

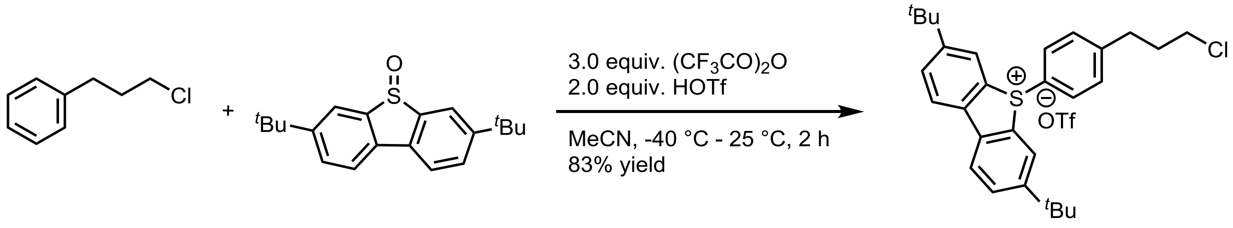

13 **B-DBTO** **S13**

A flame-dried, 10 mL nitrogen-filled *Schlenk*-tube equipped with a magnetic stir bar was charged with 1-chloro-3-phenylpropane (**13**) (77.3 mg, 0.500 mmol, 1.00 equiv.), and dry MeCN (2.0 mL, c = 0.25 M) at 25 °C. After cooling to –40 °C (acetonitrile/dry ice bath), trifluoromethanesulfonic acid (88.5 µL, 150 mg, 1.00 mmol, 2.00 equiv.) and trifluoroacetic anhydride (209 µL, 315 mg, 1.50 mmol, 3.00 equiv.) were added to the stirred reaction mixture. Subsequently, 3,7-di-*tert*-butyldibenzothiophene *S*-oxide (**B-DBTO**) (234 mg, 0.750 mmol, 1.50 equiv.) was added to the stirred reaction mixture in small portions over 1 min. After addition, the reaction mixture was stirred at –40 °C for 1 h. Subsequently, the *Schlenk*-tube was taken out of the cold bath

and warmed to 25 °C in air. After stirring at 25 °C for another 1 h, the reaction mixture was diluted with DCM (10 mL) and poured onto saturated aqueous $NaHCO_3$ (10 mL). The mixture was concentrated under reduced pressure to remove most of the MeCN solvent, and the residue was diluted with 20 mL DCM and 10 mL water. The mixture was poured into a separatory funnel, and the layers were separated. The DCM layer was collected, and the aqueous layer was further extracted with DCM (4 × ca. 30 mL). The combined DCM layer was dried over Na_2SO_4, filtered, and the solvent was removed under reduced pressure. The residue was purified by chromatography on silica gel eluting with DCM/MeOH (50:1 to 30:1, v/v) to afford an orange solid. The solid was washed with Et_2O (2 × 10 mL) and dried *in vacuo* to afford 1-chloro-3-phenylpropane-derived 3,7-di-*tert*-butyldibenzothiophenium salt **S13** (250 mg, 417 µmol, 83%) as an pale orange solid.

R*f* = 0.40 (DCM/MeOH, 9:1, v/v).

NMR Spectroscopy:

^{1}H NMR (500 MHz, CD_2Cl_2, 298 K, δ): 8.16 (d, *J* = 8.3 Hz, 2H), 8.04 (d, *J* = 1.7 Hz, 2H), 7.93 (dd, *J* = 8.4, 1.8 Hz, 2H), 7.66–7.55 (m, 2H), 7.48–7.39 (m, 2H), 3.53 (t, *J* = 6.4 Hz, 2H), 2.85 (dd, *J* = 8.6, 6.7 Hz, 2H), 2.13–2.04 (m, 2H), 1.35 (s, 18H) ppm.

^{13}C NMR (126 MHz, CD_2Cl_2, 298 K, δ): 156.3, 149.9, 136.8, 132.3, 132.3, 132.2, 131.1, 125.1, 124.1, 124.0, 121.4 (q, *J* = 321.7 Hz, CF_3), 44.4, 36.0, 33.5, 33.0, 31.1 ppm.

^{19}F NMR (471 MHz, CD_2Cl_2, 298 K, δ): –78.6 (s) ppm.

HRMS-ESI (m/z) calculated for $C_{29}H_{34}SCl^+$ $[M\text{-}OTf]^+$, 449.2064; found, 449.2062; deviation: 0.6 ppm.

N-Benzyl-nimesulide-derived dibenzothiophenium salt S14

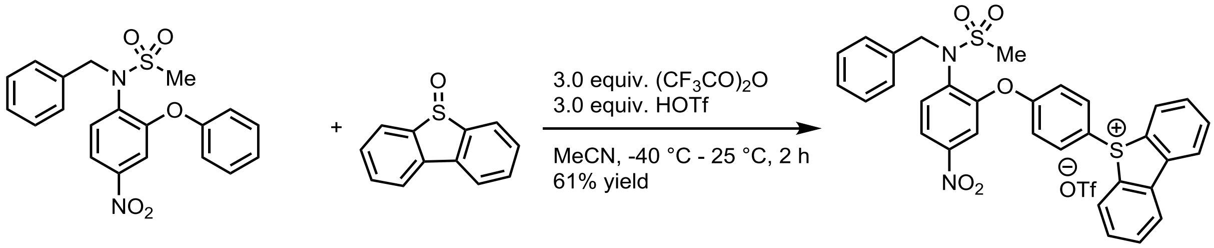

14 **DBTO** **S14**

A flame-dried, 10 mL nitrogen-filled *Schlenk*-tube equipped with a magnetic stir bar was charged with *N*-benzyl-nimesulide (**14**) (199 mg, 0.500 mmol, 1.00 equiv.) and dry MeCN (2.0 mL, c = 0.25 M) at 25 °C. After cooling to –40 °C (acetonitrile/dry ice bath), trifluoromethanesulfonic acid (133 µL, 225 mg, 1.50 mmol, 3.00 equiv.) and trifluoroacetic anhydride (209 µL, 315 mg, 1.50 mmol, 3.00 equiv.) were added to the stirred reaction mixture. Subsequently, dibenzothiophene *S*-oxide (**DBTO**) (150 mg, 0.750 mmol, 1.50 equiv.) was added to the stirred reaction mixture in small portions over 1 min. After addition, the reaction mixture was stirred at –40 °C for 1 h. Subsequently, the *Schlenk*-tube was taken out of the cold bath and warmed to 25 °C in air. After stirring at 25 °C for another 1 h, the reaction mixture was diluted with DCM (10 mL) and poured onto saturated aqueous $NaHCO_3$ (10 mL). The mixture was concentrated under reduced pressure to remove most of the MeCN solvent, and the residue was diluted with 20 mL DCM and 10 mL water. The mixture was

poured into a separatory funnel, and the layers were separated. The DCM layer was collected, and the aqueous layer was further extracted with DCM (4 × ca. 30 mL). The combined DCM layer was dried over Na_2SO_4, filtered, and the solvent was removed under reduced pressure. The residue was purified by chromatography on silica gel eluting with DCM/MeOH (40:1 to 30:1, v/v). The product was collected and dried *in vacuo* to afford *N*-benzyl-nimesulide-derived dibenzothiophenium salt **S14** (222 mg, 304 µmol, 61%) as a pale yellow solid.

R*f* = 0.18 (DCM/MeOH, 20:1, v/v).

NMR Spectroscopy:

^{1}H NMR (500 MHz, CD_3CN, 298 K, δ): 8.36 (d, *J* = 8.0, 2H), 8.12 (d, J = 8.1, 2H), 8.01–7.94 (m, 3H), 7.78–7.75 (m, 2H), 7.73 (d, *J* = 2.6 Hz, 1H), 7.65 (d, *J* = 8.7 Hz, 1H), 7.57–7.52 (m, 2H), 7.22–7.17 (m, 2H), 7.15–7.03 (m, 5H), 4.79 (s, 2H), 3.05 (s, 3H) ppm.

^{13}C NMR (126 MHz, CD_3CN, 298 K, δ): 162.1, 153.9, 148.9, 140.3, 137.8, 136.6, 135.5, 134.3, 133.8, 133.3, 132.7, 129.8, 129.5, 129.0, 129.0, 125.6, 122.1 (q, *J* = 321.5 Hz, CF_3), 121.8, 121.2, 121.0, 116.4, 55.0, 40.3 ppm.

^{19}F NMR (471 MHz, CD_3CN, 298 K, δ): –79.3 (s) ppm.

HRMS-ESI (m/z) calculated for $C_{32}H_{25}N_2O_5S_2^+$ [M-OTf]$^+$, 581.1199; found, 581.1195; deviation: 0.8 ppm.

Flurbiprofen methylester-derived dibenzothiophenium salt S15

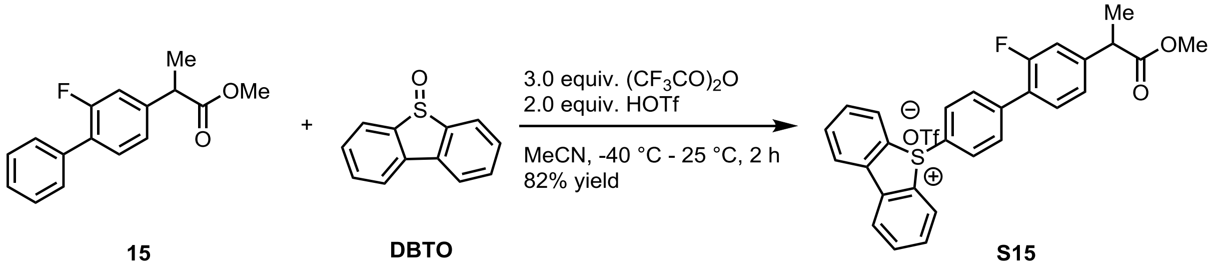

15 **DBTO** **S15**

A flame-dried, 10 mL nitrogen-filled *Schlenk*-tube equipped with a magnetic stir bar was charged with flurbiprofen methylester (**15**) (129 mg, 0.500 mmol, 1.00 equiv.) and dry MeCN (2.0 mL, c = 0.25 M) at 25 °C. After cooling to –40 °C (acetonitrile/dry ice bath), trifluoromethanesulfonic acid (88.5 µL, 150 mg, 1.00 mmol, 2.00 equiv.) and trifluoroacetic anhydride (209 µL, 315 mg, 1.50 mmol, 3.00 equiv.) were added to the stirred reaction mixture. Subsequently, dibenzothiophene *S*-oxide (**DBTO**) (150 mg, 0.750 mmol, 1.50 equiv.) was added to the stirred reaction mixture in small portions over 1 min. After addition, the reaction mixture was stirred at –40 °C for 1 h. Subsequently, the *Schlenk*-tube was taken out of the cold bath and warmed to 25 °C in air. After stirring at 25 °C for another 1 h, the reaction mixture was diluted with DCM (10 mL) and poured onto saturated aqueous $NaHCO_3$ (10 mL). The mixture was concentrated under reduced pressure to remove most of the MeCN solvent, and the residue was diluted with 20 mL DCM and 10 mL water. The mixture was poured into a separatory funnel, and the layers were separated. The DCM layer was collected, and the aqueous layer was further extracted with DCM (4 × ca. 30 mL). The combined DCM layer was dried over

Na_2SO_4, filtered, and the solvent was removed under reduced pressure. The residue was purified by chromatography on silica gel eluting with DCM/MeOH (50:1 to 30:1, v/v). The product was collected and dried *in vacuo* to afford flurbiprofen methylester-derived dibenzothiophenium salt **S15** (243 mg, 411 µmol, 82%) as a colorless solid.

R*f* = 0.52 (DCM/MeOH, 9:1, v/v).

NMR Spectroscopy:

^{1}H NMR (500 MHz, CD_3CN, 298 K, δ): 8.36 (d, *J* = 7.9 Hz, 2H), 8.13 (d, *J* = 8.1 Hz, 2H), 7.94 (t, *J* = 7.7 Hz, 2H), 7.77–7.63 (m, 6H), 7.41 (t, *J* = 8.1 Hz, 1H), 7.23–7.13 (m, 2H), 3.82 (q, J = 7.1 Hz, 1H), 3.61 (s, 3H), 1.44 (d, *J* = 7.2 Hz, 3H) ppm.

^{13}C NMR (126 MHz, CD_3CN, 298 K, δ): 174.9, 160.4 (d, *J* = 248.4 Hz), 145.7 (d, *J* = 8.2 Hz), 143.0, 140.4, 135.5, 132.9, 132.8, 132.7, 131.8 (d, *J* = 3.3 Hz), 131.6, 129.0, 126.8, 125.7, 125.6, 125.3 (d, *J* = 3.2 Hz), 122.1 (q, *J* = 320.7 Hz, CF_3), 116.4 (d, *J* = 23.3 Hz), 52.7, 45.5, 18.7 ppm.

^{19}F NMR (471 MHz, CD_3CN, 298 K, δ): –79.1 (s), –118.7 (m) ppm.

HRMS-ESI (m/z) calculated for $C_{28}H_{22}O_2SF^+$ $[M\text{-}OTf]^+$, 441.1319; found, 441.1313; deviation: 1.5 ppm.

Anisole-derived 2,8-dimethoxydibenzothiophenium salt S16

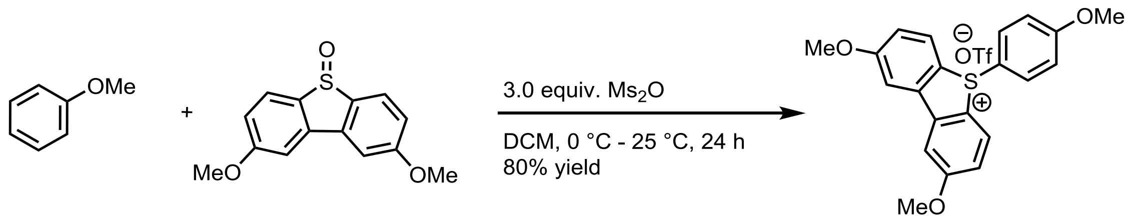

16 **M-DBTO** **S16**

A flame-dried, 10 mL nitrogen-filled *Schlenk*-tube equipped with a magnetic stir bar was charged with anisole (**16**) (54.7 mg, 0.500 mmol, 1.00 equiv.), 2,8-dimethoxydibenzothiophene *S*-oxide (**M-DBTO**) (195 mg, 0.750 mmol, 1.50 equiv.), and dry DCM (2.0 mL, c = 0.25 M) at 25 °C. After cooling to 0 °C (ice/water bath), methanesulfonic anhydride (261 mg, 1.50 mmol, 3.00 equiv.) were added dropwise over 1 min. The reaction mixture was allowed to warm to 25 °C in air. After stirring at 25 °C for 24 h, the reaction mixture was diluted with DCM (5.0 mL) and poured onto a mixture of DCM (20 mL) and saturated aqueous $NaHCO_3$ (10 mL). The mixture was poured into a separatory funnel, and the layers were separated. The DCM layer was collected, and the aqueous layer was further extracted with DCM (4 × ca. 30 mL). The combined DCM layer was washed with aqueous LiOTf solution (10% w/w, 2 × ca. 10 mL). The DCM layer was dried over Na_2SO_4, filtered, and the solvent was removed under reduced pressure. The residue was purified by chromatography on silica gel eluting with DCM/MeOH (50:1 to 30:1, v/v). The product was collected and dried *in vacuo* to afford anisole-derived 2,8-dimethoxydibenzothiophenium salt **S16** (200 mg, 400 µmol, 80 %) as an off white solid.

R*f* = 0.26 (CH_2Cl_2/MeOH, 20:1, v/v).

NMR Spectroscopy:

^{1}**H NMR** (500 MHz, CD_3CN, 298 K, δ): 7.86 (d, J = 8.9 Hz, 2H), 7.83 (d, J = 2.5 Hz, 2H), 7.49–7.45 (m, 2H), 7.20 (dd, J = 8.9, 2.6 Hz, 2H), 7.09–7.04 (m, 2H), 3.99 (s, 6H), 3.84 (s, 3H) ppm.

^{13}**C NMR** (126 MHz, CD_3CN, 298 K, δ): 165.8, 165.7, 142.2, 133.4, 129.8, 124.5, 119.3, 122.1 (q, J = 320.8 Hz, CF_3), 117.9, 117.7, 110.3, 57.3, 56.9 ppm.

^{19}**F NMR** (471 MHz, CD_3CN, 298 K, δ): –79.3 (s) ppm.

HRMS-ESI (m/z) calculated for $C_{21}H_{19}O_3S^+$ [M-OTf]$^+$, 351.1049; found, 351.1045; deviation: 1.4 ppm.

Dicamba methylester (17)

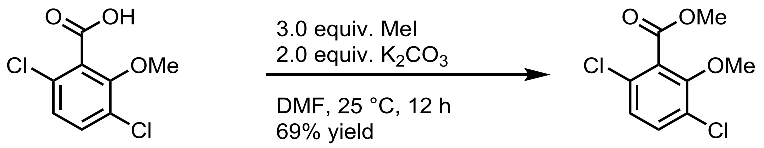

17

A 20 mL borosilicate vial with a magnetic stir bar was charged with dicamba (243 mg, 1.10 mmol, 1.00 equiv.), K_2CO_3 (304 mg, 2.20 mmol, 2.00 equiv.), and DMF (3 mL). Iodomethane (205 µL, 468 mg, 3.30 mmol, 3.00 equiv.) were added dropwise at 25 °C. After addition, the vial was sealed, and the reaction mixture was stirred at 25 °C for 12 h. The reaction mixture was diluted with ethyl acetate (10 mL) and poured into a separatory funnel. Ethyl acetate (20 mL) was added to the funnel, and the ethyl acetate layer was washed with water (2 × ca. 20 mL). The ethyl acetate layer was dried over Na_2SO_4, filtered, and the solvent was removed under reduced pressure. The residue was purified by chromatography on silica gel eluting with ethyl acetate/hexanes (50:1). The product was collected and dried *in vacuo* to afford dicamba methylester (**17**) (178 mg, 75.7 µmol, 69 %) as colorless oil.

R*f* = 0.37 (ethyl acetate/hexanes, 1:20, v/v).

NMR Spectroscopy:

^{1}**H NMR** (500 MHz, $CDCl_3$, 298 K, δ): 7.27 (d, J = 8.7 Hz, 1H), 7.03 (d, J = 8.6 Hz, 1H), 3.89 (s, 3H), 3.83 (s, 3H) ppm.

^{13}**C NMR** (126 MHz, CD_3CN, 298 K, δ): 165.0, 153.8, 131.8, 130.4, 129.6, 126.7, 125.8, 62.2, 52.9 ppm.

HRMS-EI (m/z) calculated for $C_9H_8O_3Cl_2^+$ [M]$^+$, 233.9845; found, 233.9843; deviation: 0.9 ppm.

Dicamba methylester-derived dibenzothiophenium salt S17

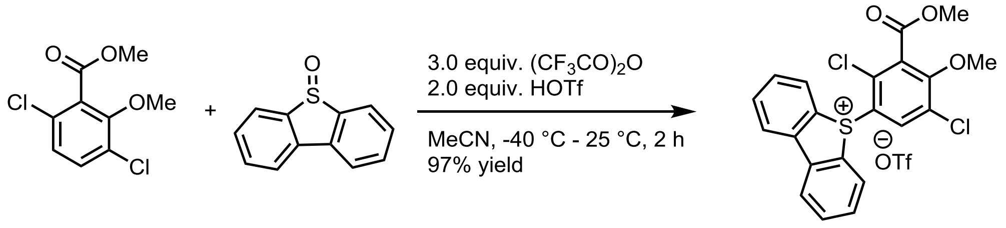

17 **DBTO** **S17**

A flame-dried, 10 mL nitrogen-filled *Schlenk*-tube equipped with a magnetic stir bar was charged with dicamba methylester (**17**) (118 mg, 0.500 mmol, 1.00 equiv.) and dry MeCN (2.0 mL, c = 0.25 M) at 25 °C. After cooling to –40 °C (acetonitrile/dry ice bath), trifluoromethanesulfonic acid (88.5 µL, 150 mg, 1.00 mmol, 2.00 equiv.) and trifluoroacetic anhydride (209 µL, 315 mg, 1.50 mmol, 3.00 equiv.) were added to the stirred reaction mixture. Subsequently, dibenzothiophene *S*-oxide (**DBTO**) (150 mg, 0.750 mmol, 1.50 equiv.) was added to the stirred reaction mixture in small portions over 1 min. After addition, the reaction mixture was stirred at –40 °C for 1 h. Subsequently, the *Schlenk*-tube was taken out of the cold bath and warmed to 25 °C in air. After stirring at 25 °C for another 1 h, the reaction mixture was diluted with DCM (10 mL) and poured onto saturated aqueous $NaHCO_3$ (10 mL). The mixture was concentrated under reduced pressure to remove most of the MeCN solvent, and the residue was diluted with 20 mL DCM and 10 mL water. The mixture was poured into a separatory funnel, and the layers were separated. The DCM layer was collected, and the aqueous layer was further extracted with DCM (4 × ca. 30 mL). The combined DCM layer was dried over Na_2SO_4, filtered, and the solvent was removed under reduced pressure. The residue was purified by chromatography on silica gel eluting with DCM/MeOH (50:1 to 30:1, v/v). The product was collected and dried *in vacuo* to afford dicamba methylester-derived dibenzothiophenium salt **S17** (275 mg, 485 µmol, 97%) as a colorless solid.

R*f* = 0.50 (DCM/MeOH, 9:1, v/v).

NMR Spectroscopy:

^{1}H NMR (500 MHz, CD_3CN, 298 K, δ): 8.35 (dd, *J* = 7.9, 1.2 Hz, 2H), 8.15 (d, *J* = 8.1 Hz, 2H), 8.00 (td, *J* = 7.7, 1.1 Hz, 2H), 7.79–7.76 (m, 2H), 7.03 (brs, 1H), 4.01 (s, 3H), 3.93 (s, 3H) ppm.

^{13}C NMR (126 MHz, CD_3CN, 298 K, δ): 164.1, 159.8, 141.0, 136.2, 134.2, 133.0, 130.8, 130.2, 129.4, 126.1, 122.2 (q, *J* = 320.9 Hz, CF_3), 63.5, 54.6 ppm.

^{19}F NMR (471 MHz, CD_3CN, 298 K, δ): –79.3 (s) ppm.

HRMS-ESI (m/z) calculated for $C_{21}H_{15}O_3SCl_2^+$ $[M-OTf]^+$, 417.0113; found, 417.0108; deviation: 1.4 ppm.

Clofibrate-derived dibenzothiophenium salt S18

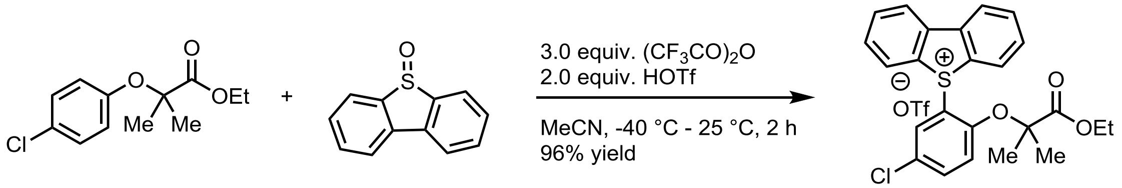

18 **DBTO** **S18**

A flame-dried, 10 mL nitrogen-filled *Schlenk*-tube equipped with a magnetic stir bar was charged with clofibrate (**18**) (121 mg, 0.500 mmol, 1.00 equiv.) and dry MeCN (2.0 mL, c = 0.25 M) at 25 °C. After cooling to –40 °C (acetonitrile/dry ice bath), trifluoromethanesulfonic acid (88.5 µL, 150 mg, 1.00 mmol, 2.00 equiv.) and trifluoroacetic anhydride (209 µL, 315 mg, 1.50 mmol, 3.00 equiv.) were added to the stirred reaction mixture. Subsequently, dibenzothiophene *S*-oxide (**DBTO**) (150 mg, 0.750 mmol, 1.50 equiv.) was added to the stirred reaction mixture in small portions over 1 min. After addition, the reaction mixture was stirred at –40 °C for 1 h. Subsequently, the *Schlenk*-tube was taken out of the cold bath and warmed to 25 °C in air. After stirring at 25 °C for another 1 h, the reaction mixture was diluted with DCM (10 mL) and poured onto saturated aqueous $NaHCO_3$ (10 mL). The mixture was concentrated under reduced pressure to remove most of the MeCN solvent, and the residue was diluted with 20 mL DCM and 10 mL water. The mixture was poured into a separatory funnel, and the layers were separated. The DCM layer was collected, and the aqueous layer was further extracted with DCM (4 × ca. 30 mL). The combined DCM layer was dried over Na_2SO_4, filtered, and the solvent was removed under reduced pressure. The residue was purified by chromatography on silica gel eluting with DCM/MeOH (50:1 to 30:1, v/v). The product was collected and dried *in vacuo* to afford clofibrate-derived dibenzothiophenium salt **S18** (276 mg, 480 µmol, 96%) as a colorless solid.

R*f* = 0.42 (DCM/MeOH, 9:1, v/v).

NMR Spectroscopy:

^{1}H NMR (500 MHz, CD_3CN, 298 K, δ): 8.33 (d, *J* = 7.8 Hz, 2H), 8.14 (d, *J* = 8.0 Hz, 2H), 7.95 (d, *J* = 7.7 Hz, 2H), 7.80–7.72 (m, 2H), 7.68–7.60 (m, 1H), 7.46 (brs, 1H), 6.91 (d, *J* = 9.1 Hz, 1H), 4.12 (q, *J* = 7.1 Hz, 2H), 1.42 (s, 6H), 1.12 (t, *J* = 7.1 Hz, 3H) ppm.

^{13}C NMR (126 MHz, CD_3CN, 298 K, δ): 172.5, 155.3, 141.0, 137.5, 135.4, 132.5, 130.2, 128.8, 125.5, 122.2 (q, *J* = 320.7 Hz, CF_3), 119.8, 118.3, 83.3, 63.1, 25.2, 14.2 ppm.

^{19}F NMR (471 MHz, CD_3CN, 298 K, δ): –79.3 (s) ppm.

HRMS-ESI (m/z) calculated for $C_{24}H_{22}O_3SCl^+$ [M-OTf]$^+$, 425.0973; found, 425.0967; deviation: 1.4 ppm.

Bifonazole-derived dibenzothiophenium salt S19

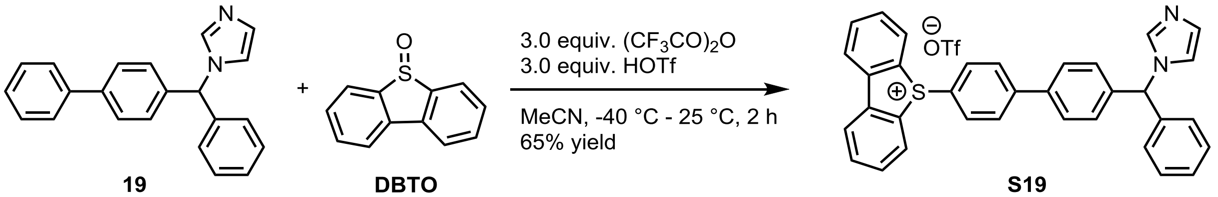

A flame-dried, 10 mL nitrogen-filled *Schlenk*-tube equipped with a magnetic stir bar was charged with bifonazole (**19**) (155 mg, 0.500 mmol, 1.00 equiv.) and dry MeCN (2.0 mL, c = 0.25 M) at 25 °C. After cooling to –40 °C (acetonitrile/dry ice bath), trifluoromethanesulfonic acid (133 µL, 225 mg, 1.50 mmol, 3.00 equiv.) and trifluoroacetic anhydride (209 µL, 315 mg, 1.50 mmol, 3.00 equiv.) were added to the stirred reaction mixture. Subsequently, dibenzothiophene *S*-oxide (**DBTO**) (150 mg, 0.750 mmol, 1.50 equiv.) was added to the stirred reaction mixture in small portions over 1 min. After addition, the reaction mixture was stirred at –40 °C for 1 h. Subsequently, the *Schlenk*-tube was taken out of the cold bath and warmed to 25 °C in air. After stirring at 25 °C for another 1 h, the reaction mixture was diluted with DCM (10 mL) and poured onto saturated aqueous $NaHCO_3$ (10 mL). The mixture was concentrated under reduced pressure to remove most of the MeCN solvent, and the residue was diluted with 20 mL DCM and 10 mL water. The mixture was poured into a separatory funnel, and the layers were separated. The DCM layer was collected, and the aqueous layer was further extracted with DCM (4 × ca. 30 mL). The combined DCM layer was dried over Na_2SO_4, filtered, and the solvent was removed under reduced pressure. The residue was purified by chromatography on silica gel eluting with DCM/MeOH (30:1 to 10:1, v/v) to afford a colorless solid. The solid was washed with 5.0 mL Et_2O and dried *in vacuo* to afford bifonazole-derived dibenzothiophenium salt **S19** (210 mg, 327 µmol, 65%) as a colorless solid.

R*f* = 0.40 (DCM/MeOH, 9:1, v/v).

NMR Spectroscopy:

^{1}H NMR (500 MHz, CD_3CN, 298 K, δ): 8.32 (d, *J* = 7.9 Hz, 2H), 8.11 (d, *J* = 8.1 Hz, 2H), 7.90 (t, *J* = 8.1 Hz, 2H), 7.72 (d, *J* = 8.8 Hz, 2H), 7.67 (d, *J* = 8.2 Hz, 2H), 7.61 (d, *J* = 8.8 Hz, 2H), 7.53 (d, *J* = 8.4 Hz, 2H), 7.47 (s, 1H), 7.35–7.29 (m, 3H), 7.21–7.08 (m, 4H), 6.95 (d, *J* = 11.7 Hz, 2H), 6.72 (s, 1H).

^{13}C NMR (126 MHz, CD_3CN, 298 K, δ): 147.3, 141.9, 140.3, 140.3, 138.5, 138.2 (brs), 135.4, 133.0, 132.6, 132.0, 130.6, 129.9, 129.8, 129.6, 129.3, 128.9, 128.6, 126.4, 125.6, 122.1 (q, *J* = 321.1 Hz, CF_3), 120.2 (brs), 64.7 ppm.

^{19}F NMR (471 MHz, CD_3CN, 298 K, δ): –79.0 (s) ppm.

HRMS-ESI (m/z) calculated for $C_{34}H_{25}N_2S^+$ $[M-OTf]^+$, 493.1733; found, 493.1732; deviation: 0.1 ppm.

Pyriproxyfen-derived 2,8-dimethoxydibenzothiophenium salt S20

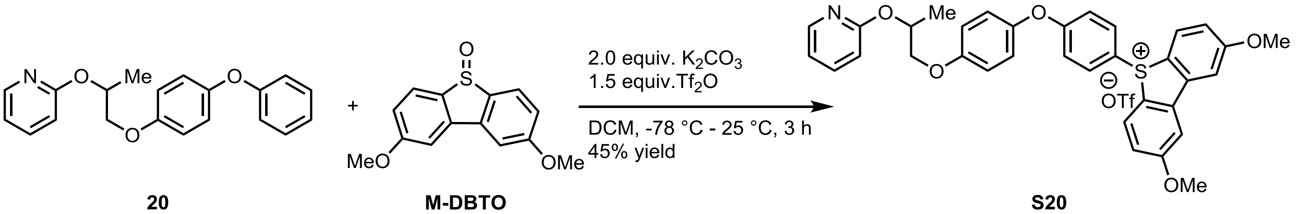

20 M-DBTO S20

A flame-dried, 10 mL nitrogen-filled *Schlenk*-tube equipped with a magnetic stir bar was charged with pyriproxyfen (**20**) (161 mg, 0.500 mmol, 1.00 equiv.), 2,8-dimethoxydibenzothiophene *S*-oxide (**M-DBTO**) (195 mg, 0.750 mmol, 1.50 equiv.), K_2CO_3 (138 mg, 1.00 mmol, 2.00 equiv.), and dry DCM (2.0 mL, c = 0.25 M) at 25 °C. After cooling to –78 °C (acetone/dry ice bath), trifluoromethanesulfonic anhydride (126 µL, 0.750 mmol, 1.50 equiv.) was added to the reaction mixture dropwise in 2 min. After addition, the reaction mixture was stirred at –78 °C for 15 min and then allowed to warm to –5 °C over 2 h. The *Schlenk*-tube was taken out of the cold bath, and the reaction mixture was allowed to warm to 25 °C in air. After stirring at 25 °C for 1 h, the reaction mixture was diluted with DCM (5.0 mL) and poured onto a mixture of DCM (20 mL) and saturated aqueous $NaHCO_3$ (10 mL). The mixture was poured into a separatory funnel, and the layers were separated. The DCM layer was collected, and the aqueous layer was further extracted with DCM (4 × ca. 30 mL). The combined DCM layer was dried over Na_2SO_4, filtered, and the solvent was removed under reduced pressure. The residue was purified by chromatography on silica gel eluting with DCM/MeOH (100:0 to 20:1, v/v). The product was collected and dried *in vacuo* to afford pyriproxyfen-derived 2,8-dimethoxydibenzothiophenium salt **S20** (160 mg, 224 µmol, 45 %) as an orange solid.

R*f* = 0.19 (CH_2Cl_2/MeOH, 20:1, v/v).

NMR Spectroscopy:

^{1}H NMR (500 MHz, CD_3CN, 298 K, δ): 8.12–8.08 (m, 1H), 7.87 (d, *J* = 9.0 Hz, 2H), 7.82–7.79 (m, 2H), 7.64–7.59 (m, 1H), 7.49–7.42 (m, 2H), 7.19–7.17 (m, 2H), 6.99–6.92 (m, 6H), 6.91–6.88 (m, 1H), 6.70–6.66 (m, 1H), 5.55–5.49 (m, 1H), 4.16–4.07 (m, 2H), 3.97 (s, 6H), 1.37 (d, *J* = 6.4 Hz, 3H) ppm.

^{13}C NMR (126 MHz, CD_3CN, 298 K, δ): 165.7, 165.1, 164.0, 157.4, 148.6, 147.8, 142.3, 140.1, 133.5, 129.9, 124.2, 122.9, 122.1 (q, *J* = 321.3 Hz, CF_3), 119.8, 119.8, 119.3, 117.9, 117.0, 112.2, 110.3, 71.9, 70.2, 57.3, 17.0 ppm.

^{19}F NMR (471 MHz, CD_3CN, 298 K, δ): –79.1 (s) ppm.

HRMS-ESI (m/z) calculated for $C_{34}H_{30}NO_5S^+$ [M-OTf]$^+$, 564.1839; found, 564.1837; deviation: 0.3 ppm.

Fluorobenzene-derived dibenzothiophenium salt S21

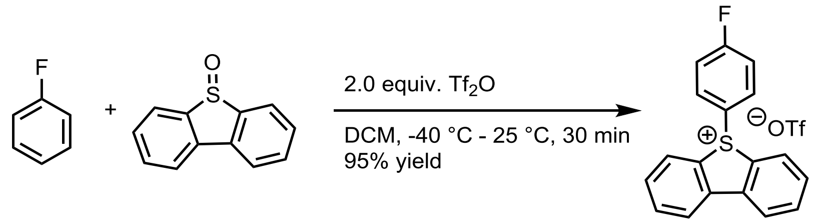

21 **DBTO** **S21**

A flame-dried, 10 mL nitrogen-filled *Schlenk*-tube equipped with a magnetic stir bar was charged with fluorobenzene (**21**) (48.1 mg, 0.500 mmol, 1.00 equiv.), dibenzothiophene *S*-oxide (**DBTO**) (200 mg, 1.00 mmol, 2.00 equiv.), and dry DCM (2.0 mL, c = 0.25 M) at 25 °C. After cooling to –40 °C (acetonitrile/dry ice bath), trifluoromethanesulfonic anhydride (168 µL, 282 mg, 1.00 mmol, 2.00 equiv.) was added dropwise over 1 min. The *Schlenk*-tube was sealed, taken out of the cold bath and warmed to 25 °C in air. After stirring at 25 °C for 30 min, the reaction mixture was diluted with DCM (5.0 mL) and poured onto a mixture of DCM (20 mL) and saturated aqueous $NaHCO_3$ (5.0 mL). The mixture was poured into a separatory funnel, and the layers were separated. The DCM layer was collected, and the aqueous layer was further extracted with DCM (4 × ca. 30 mL). The combined DCM layer was dried over Na_2SO_4, filtered, and the solvent was removed under reduced pressure. The residue was purified by chromatography on silica gel eluting with DCM/MeOH (50:1 to 30:1, v/v). The product was collected and dried *in vacuo* to afford fluorobenzene-derived dibenzothiophenium salt **S21** (204 mg, 477 µmol, 95 %) as an off white solid.

R*f* = 0.48 (DCM/MeOH, 9:1, v/v).

NMR Spectroscopy:

^{1}**H NMR** (500 MHz, CD_3CN, 298 K, δ): 8.34 (d, *J* = 7.9 Hz, 2H), 8.10 (d, *J* = 8.1 Hz, 2H), 7.93 (td, *J* = 7.7, 1.1 Hz, 2H), 7.75–7.62 (m, 4H), 7.40–7.28 (m, 2H) ppm.

^{13}**C NMR** (126 MHz, CD_3CN, 298 K, δ): 167.3 (d, *J* = 256.8 Hz), 140.3, 135.5, 134.8 (d, *J* = 10.2 Hz), 133.0, 132.7, 129.0, 125.6, 123.1 (d, *J* = 3.2 Hz), 122.1 (q, *J* = 321.3 Hz, CF_3), 119.9 (d, *J* = 23.8 Hz) ppm.

^{19}**F NMR** (471 MHz, CD_3CN, 298 K, δ): –79.1 (s), –102.9 (m) ppm.

HRMS-ESI (m/z) calculated for $C_{18}H_{12}SF^{+}$ $[M\text{-}OTf]^{+}$, 279.0638; found, 279.0636; deviation: 0.7 ppm.

Chlorobenzene-derived dibenzothiophenium salt S22

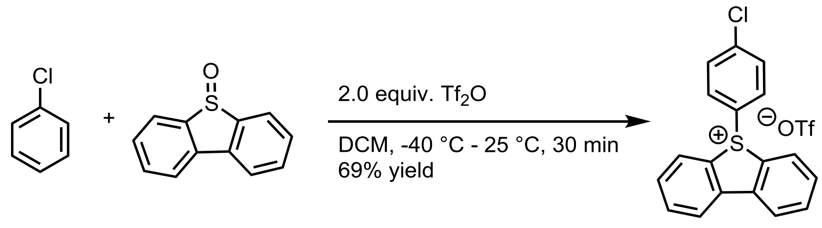

22 **DBTO** **S22**

A flame-dried, 10 mL nitrogen-filled *Schlenk*-tube equipped with a magnetic stir bar was charged with chlorobenzene (**22**) (48.1 mg, 0.500 mmol, 1.00 equiv.), dibenzothiophene *S*-oxide (**DBTO**) (200 mg, 1.00 mmol, 2.00 equiv.), and dry DCM (2.0 mL, c = 0.25 M) at 25 °C. After cooling to –40 °C (acetonitrile/dry ice bath), trifluoromethanesulfonic anhydride (168 µL, 282 mg, 1.00 mmol, 2.00 equiv.) was added dropwise over 1 min. The *Schlenk*-tube was sealed, taken out of the cold bath and warmed to 25 °C in air. After stirring at 25 °C for 30 min, the reaction mixture was diluted with DCM (5.0 mL) and poured onto a mixture of DCM (20 mL) and saturated aqueous $NaHCO_3$ (5.0 mL). The mixture was poured into a separatory funnel, and the layers were separated. The DCM layer was collected, and the aqueous layer was further extracted with DCM (4 × ca. 30 mL). The combined DCM layer was dried over Na_2SO_4, filtered, and the solvent was removed under reduced pressure. The residue was purified by chromatography on silica gel eluting with DCM/MeOH (50:1 to 30:1, v/v) to afford a slightly yellow solid. The solid was then dissolved in 2.0 mL $CHCl_3$, and a colorless solid precipitated after a few minutes. The solvent was decanted, and the solid was dried *in vacuo* to afford chlorobenzene-derived dibenzothiophenium salt **S22** (155 mg, 347 µmol, 69 %) as a colorless solid.

R*f* = 0.50 (DCM/MeOH, 9:1, v/v).

NMR Spectroscopy:

^{1}**H NMR** (500 MHz, CD_3CN, 298 K, δ): 8.34 (d, *J* = 8.0 Hz, 2H), 8.11 (d, *J* = 8.1 Hz, 2H), 7.93 (td, *J* = 7.7, 1.1 Hz, 2H), 7.71 (td, *J* = 7.6, 1.0 Hz, 2H), 7.61–7.53 (m, 4H) ppm.

^{13}**C NMR** (126 MHz, CD_3CN, 298 K, δ): 142.1, 140.4, 135.6, 133.1, 132.7, 132.7, 132.6, 129.1, 126.4, 125.6, 122.1 (q, *J* = 320.8 Hz, CF_3) ppm.

^{19}**F NMR** (471 MHz, CD_3CN, 298 K, δ): –79.1 (s) ppm.

HRMS-ESI (m/z) calculated for $C_{18}H_{12}SCl^{+}$ $[M\text{-}OTf]^{+}$, 295.0343; found, 295.0339; deviation: 1.4 ppm.

Iodobenzene-derived dibenzothiophenium salt S23

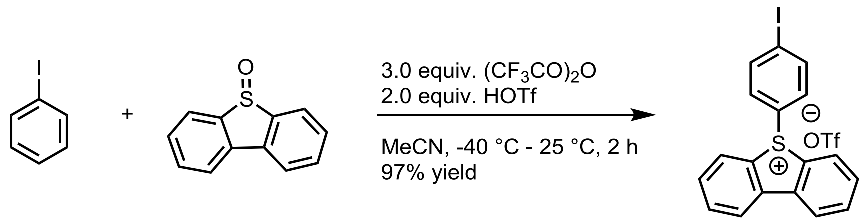

23 **DBTO** **S23**

A flame-dried, 10 mL nitrogen-filled *Schlenk*-tube equipped with a magnetic stir bar was charged with iodobenzene (**23**) (102 mg, 0.500 mmol, 1.00 equiv.) and dry MeCN (2.0 mL, c = 0.25 M) at 25 °C. After cooling to –40 °C (acetonitrile/dry ice bath), trifluoromethanesulfonic acid (88.5 µL, 150 mg, 1.00 mmol, 2.00 equiv.) and trifluoroacetic anhydride (209 µL, 315 mg, 1.50 mmol, 3.00 equiv.) were added to the stirred reaction mixture. Subsequently, dibenzothiophene *S*-oxide (**DBTO**) (150 mg, 0.750 mmol, 1.50 equiv.) was added to the stirred reaction mixture in small portions over 1 min. After addition, the reaction mixture was stirred at –40 °C for 1 h. Subsequently, the *Schlenk*-tube was taken out of the cold bath and warmed to 25 °C

in air. After stirring at 25 °C for another 1 h, the reaction mixture was diluted with DCM (10 mL) and poured onto saturated aqueous $NaHCO_3$ (10 mL). The mixture was concentrated under reduced pressure to remove most of the MeCN solvent, and the residue was diluted with 20 mL DCM and 10 mL water. The mixture was poured into a separatory funnel, and the layers were separated. The DCM layer was collected, and the aqueous layer was further extracted with DCM (4 × ca. 30 mL). The combined DCM layer was dried over Na_2SO_4, filtered, and the solvent was removed under reduced pressure. The residue was purified by chromatography on silica gel eluting with DCM/MeOH (50:1 to 30:1, v/v) to afford a solid. The solid was washed with 2 mL Et_2O and dried *in vacuo* to afford iodobenzene-derived dibenzothiophenium salt **S23** (260 mg, 485 µmol, 97%) as a colorless solid.

R*f* = 0.49 (DCM/MeOH, 9:1, v/v).

NMR Spectroscopy:

^{1}**H NMR** (500 MHz, DMSO-d_6, 298 K, δ): δ 8.51 (dd, *J* = 7.9, 1.1 Hz, 2H), 8.38 (dd, *J* = 8.0, 1.0 Hz, 2H), 8.00–7.91 (m, 4H), 7.76 (m, 2H), 7.42–7.32 (m, 2H) ppm.

^{13}**C NMR** (126 MHz, DMSO-d_6, 298 K, δ): 139.9, 139.2, 133.9, 133.0, 131.3, 131.2, 129.0, 128.3, 124.5, 120.7 (q, *J* = 322.8 Hz, CF_3), 102.7 ppm.

^{19}**F NMR** (471 MHz, DMSO-d_6, 298 K, δ): –77.7 (s) ppm.

HRMS-ESI (m/z) calculated for $C_{18}H_{12}SI^+$ [M-OTf]$^+$, 386.9699; found, 386.9693; deviation: 1.6 ppm.

4-Bromodiphenylether-derived 2,8-dimethoxydibenzothiophenium salt S24

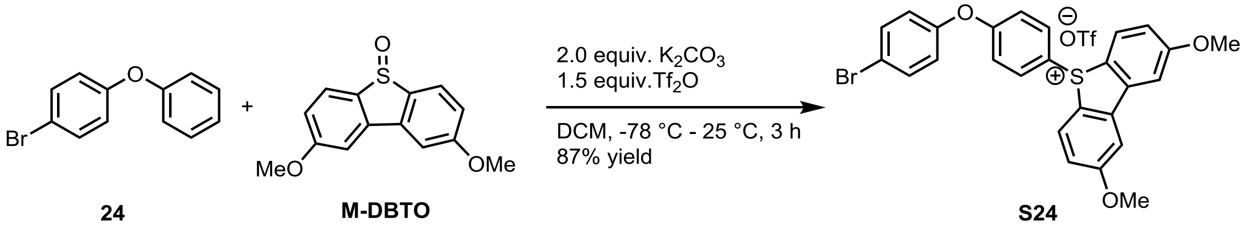

24 **M-DBTO** **S24**

A flame-dried, 10 mL nitrogen-filled *Schlenk*-tube equipped with a magnetic stir bar was charged with 4-bromodiphenylether (**24**) (125 mg, 0.500 mmol, 1.00 equiv.), 2,8-dimethoxydibenzothiophene *S*-oxide (**M-DBTO**) (195 mg, 0.750 mmol, 1.50 equiv.), K_2CO_3 (138 mg, 1.00 mmol, 2.00 equiv.), and dry DCM (2.0 mL, c = 0.25 M) at 25 °C. After cooling to –78 °C (acetone/dry ice bath), trifluoromethanesulfonic anhydride (126 µL, 0.750 mmol, 1.50 equiv.) was added to the reaction mixture dropwise in 2 min. After addition, the reaction mixture was stirred at –78 °C for 15 min and then allowed to warm to –5 °C over 2 h. The *Schlenk*-tube was taken out of the cold bath, and the reaction mixture was allowed to warm to 25 °C in air. After stirring at 25 °C for 1 h, the reaction mixture was diluted with DCM (5.0 mL) and poured onto a mixture of DCM (20 mL) and saturated aqueous $NaHCO_3$ (10 mL). The mixture was poured into a separatory funnel, and the layers were separated. The DCM layer was collected, and the aqueous layer was further extracted with DCM (4 × ca. 30 mL). The combined DCM layer was dried over Na_2SO_4, filtered, and the solvent was removed under reduced pressure. The residue was purified by chromatography on silica gel eluting with DCM/MeOH (50:1 to 30:1,

v/v) to afford an orange solid. The solid was washed with Et_2O (2 × 2 mL) and dried *in vacuo* to afford 4-bromodiphenylether-derived 2,8-dimethoxydibenzothiophenium salt **S24** (279 mg, 435 µmol, 87 %) as a pale orange solid.

R*f* = 0.20 (DCM/MeOH, 20:1, v/v).

NMR Spectroscopy:

^{1}**H NMR** (500 MHz, CD_3CN, 298 K, δ): 7.92 (d, *J* = 8.9 Hz, 2H), 7.86 (d, *J* = 2.5 Hz, 2H), 7.64–7.56 (m, 2H), 7.57–7.50 (m, 2H), 7.24 (dd, *J* = 9.0, 2.6 Hz, 2H), 7.14–7.06 (m, 2H), 7.10–6.99 (m, 2H), 4.02 (s, 6H) ppm.

^{13}**C NMR** (126 MHz, CD_3CN, 298 K, δ): 165.8, 163.9, 154.8, 142.4, 134.4, 133.6, 130.0, 124.1, 123.5, 122.1 (q, *J* = 321.5 Hz, CF_3), 121.1, 120.7, 119.3, 118.8, 110.4, 57.4 ppm.

^{19}**F NMR** (471 MHz, CD_3CN, 298 K, δ): −79.2 (s) ppm.

HRMS-ESI (m/z) calculated for $C_{26}H_{20}O_3SBr^+$ [M-OTf]$^+$, 491.0311; found, 491.0308; deviation: 0.7 ppm.

1,3-Dimethylquinazoline-2,4(1*H*,3*H*)-dione-derived 3,7-di-*tert*-butyldibenzothiophenium salt S25

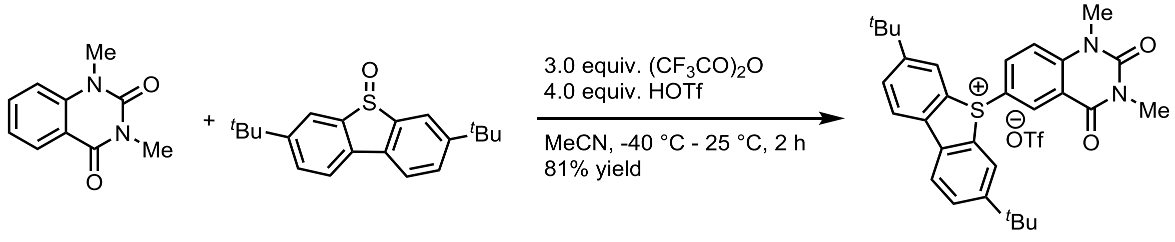

25 **B-DBTO** **S25**

A flame-dried, 10 mL nitrogen-filled *Schlenk*-tube equipped with a magnetic stir bar was charged with 1,3-dimethylquinazoline-2,4(1*H*,3*H*)-dione (**25**) (95.1 mg, 0.500 mmol, 1.00 equiv.), and dry MeCN (2.0 mL, c = 0.25 M) at 25 °C. After cooling to –40 °C (acetonitrile/dry ice bath), trifluoromethanesulfonic acid (177 µL, 300 mg, 2.00 mmol, 4.00 equiv.) and trifluoroacetic anhydride (209 µL, 315 mg, 1.50 mmol, 3.00 equiv.) were added to the stirred reaction mixture. Subsequently, 3,7-di-*tert*-butyldibenzothiophene *S*-oxide (**B-DBTO**) (234 mg, 0.750 mmol, 1.50 equiv.) was added to the stirred reaction mixture in small portions over 1 min. After addition, the reaction mixture was stirred at –40 °C for 1 h. Subsequently, the *Schlenk*-tube was taken out of the cold bath and warmed to 25 °C in air. After stirring at 25 °C for another 1 h, the reaction mixture was diluted with DCM (10 mL) and poured onto saturated aqueous $NaHCO_3$ (10 mL). The mixture was concentrated under reduced pressure to remove most of the MeCN solvent, and the residue was diluted with 20 mL DCM and 10 mL water. The mixture was poured into a separatory funnel, and the layers were separated. The DCM layer was collected, and the aqueous layer was further extracted with DCM (4 × ca. 30 mL). The combined DCM layer was dried over Na_2SO_4, filtered, and the solvent was removed under reduced pressure. The residue was purified by chromatography on silica gel eluting with DCM/MeOH (30:1, v/v) to afford a solid. The solid was dissolved in 2 mL DCM, and precipitated with 10 mL hexanes. The suspension was decanted, and the solid was again dissolved in 2 mL DCM, and precipitated with 10 mL hexanes. The

suspension was decanted, and the solid was dried *in vacuo* to afford 1,3-dimethylquinazoline-2,4(1*H*,3*H*)-dione-derived 3,7-di-*tert*-butyldibenzothiophenium salt **S25** (257 mg, 404 µmol, 81%) as an off white solid.

R*f* = 0.46 (DCM/MeOH, 9:1, v/v).

NMR Spectroscopy:

^{1}H NMR (500 MHz, CD_3CN, 298 K, δ): 8.51 (d, *J* = 2.5 Hz 1H), 8.25 (d, *J* = 8.3 Hz, 2H), 8.17 (s, 2H), 7.99 (d, *J* = 8.4 Hz, 2H), 7.54 (dd, J = 9.1, 2.5 Hz, 1H), 7.46–7.40 (m, 1H), 3.47 (s, 3H), 3.29 (s, 3H), 1.32 (s, 18H) ppm.

^{13}C NMR (126 MHz, CD_3CN, 298 K, δ: 161.0, 156.7, 151.5, 146.1, 137.7, 136.0, 133.6, 133.4, 133.0, 125.9, 125.0, 122.1 (q, *J* = 320.8 Hz, CF_3), 120.2, 119.0, 117.8, 36.5, 31.9, 31.2, 29.0 ppm.

^{19}F NMR (471 MHz, CD_3CN, 298 K, δ): –79.1 (s) ppm.

HRMS-ESI (m/z) calculated for $C_{30}H_{33}N_2O_2S^+$ $[M\text{-}OTf]^+$, 485.2257; found, 485.2254; deviation: 0.6 ppm.

1,3-Dimethylquinazoline-2,4(1*H*,3*H*)-dione-derived dibenzothiophenium salt S25-DBT

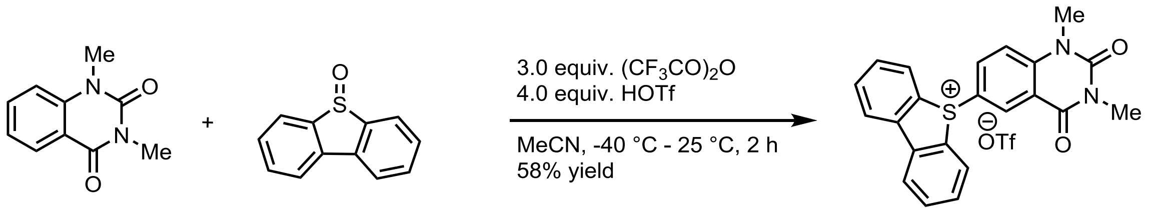

25 **DBTO** **S25-DBT**

A flame-dried, 10 mL nitrogen-filled *Schlenk*-tube equipped with a magnetic stir bar was charged with 1,3-dimethylquinazoline-2,4(1*H*,3*H*)-dione (**25**) (95.1 mg, 0.500 mmol, 1.00 equiv.), and dry MeCN (2.0 mL, c = 0.25 M) at 25 °C. After cooling to –40 °C (acetonitrile/dry ice bath), trifluoromethanesulfonic acid (177 µL, 300 mg, 2.00 mmol, 4.00 equiv.) and trifluoroacetic anhydride (209 µL, 315 mg, 1.50 mmol, 3.00 equiv.) were added to the stirred reaction mixture. Subsequently, dibenzothiophene *S*-oxide (**DBTO**) (150 mg, 0.750 mmol, 1.50 equiv.) was added to the stirred reaction mixture in small portions over 1 min. After addition, the reaction mixture was stirred at –40 °C for 1 h. Subsequently, the *Schlenk*-tube was taken out of the cold bath and warmed to 25 °C in air. After stirring at 25 °C for another 1 h, the reaction mixture was diluted with DCM (10 mL) and poured onto saturated aqueous $NaHCO_3$ (10 mL). The mixture was concentrated under reduced pressure to remove most of the MeCN solvent, and the residue was diluted with 20 mL DCM and 10 mL water. The mixture was poured into a separatory funnel, and the layers were separated. The DCM layer was collected, and the aqueous layer was further extracted with DCM (4 × ca. 30 mL). The combined DCM layer was dried over Na_2SO_4, filtered, and the solvent was removed under reduced pressure. The residue was purified by chromatography on silica gel eluting with DCM/MeOH (50:1 to 20:1, v/v). The product was collected and dried *in vacuo* to afford 1,3-dimethylquinazoline-2,4(1*H*,3*H*)-dione-derived dibenzothiophenium salt **S25-DBT** (152 mg, 292 µmol, 58%) as a white solid.

R*f* = 0.38 (DCM/MeOH, 9:1, v/v).

NMR Spectroscopy:

^{1}H NMR (500 MHz, CD_3CN, 298 K, δ): 8.55 (d, *J* = 2.5 Hz, 1H), 8.37 (dd, *J* = 8.0, 1.2 Hz, 2H), 8.14–8.10 (m, 2H), 7.95 (td, *J* = 7.7, 1.0 Hz, 2H), 7.72 (td, *J* = 7.8, 1.2 Hz, 2H), 7.49 (dd, *J* = 9.2, 2.5 Hz, 1H), 7.41 (d, *J* = 9.1 Hz, 1H), 3.47 (s, 3H), 3.30 (s, 3H) ppm.

^{13}C NMR (126 MHz, CD_3CN, 298 K, δ: 161.0, 151.6, 146.3, 140.4, 135.8, 135.6, 133.8, 133.1, 132.8, 129.0, 125.7, 122.1 (q, *J* = 320.8 Hz, CF_3), 119.7, 119.2, 118.3, 117.9, 31.9, 29.0 ppm.

^{19}F NMR (471 MHz, CD_3CN, 298 K, δ): –79.2 (s) ppm.

HRMS-ESI (m/z) calculated for $C_{22}H_{17}N_2O_2S^+$ $[M-OTf]^+$, 373.1005; found, 373.1003; deviation: 0.6 ppm.

Airacetam-derived 3,7-di-*tert*-butyldibenzothiophenium salt S26

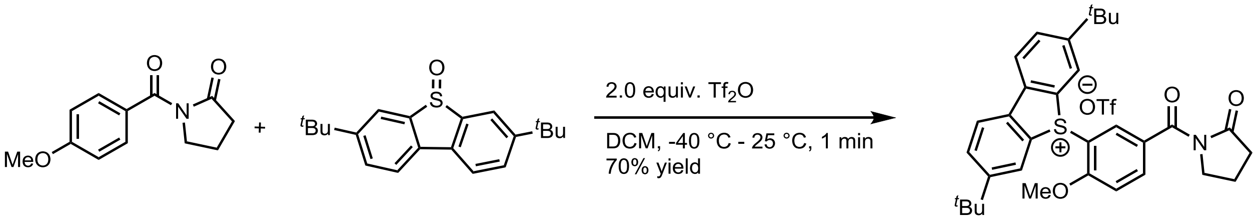

26 **B-DBTO** **S26**

A flame-dried, 10 mL nitrogen-filled *Schlenk*-tube equipped with a magnetic stir bar was charged with airacetam (**26**) (110 mg, 0.500 mmol, 1.00 equiv.), 3,7-di-*tert*-butyldibenzothiophene *S*-oxide (**B-DBTO**) (234 mg, 0.750 mmol, 1.50 equiv.), and dry DCM (2.0 mL, c = 0.25 M) at 25 °C. After cooling to –40 °C (acetonitrile/dry ice bath), trifluoromethanesulfonic anhydride (168 µL, 282 mg, 1.00 mmol, 2.00 equiv.) was added dropwise over 2 min. The *Schlenk*-tube was sealed and taken out of the cold bath. After stirring at 25 °C for 1 min (product decomposed as reaction proceeded), the reaction mixture was poured immediately onto a mixture of DCM (20 mL) and saturated aqueous $NaHCO_3$ (5.0 mL). The mixture was poured into a separatory funnel, and the layers were separated. The DCM layer was collected, and the aqueous layer was further extracted with DCM (4 × ca. 30 mL). The combined DCM layer was dried over Na_2SO_4, filtered, and the solvent was removed under reduced pressure. The residue was purified by chromatography on silica gel eluting with DCM/MeOH (50:1 to 30:1, v/v) to afford a slightly yellow thick oil. The oil was diluted with DCM (0.3 mL), and Et_2O (6 mL) was subsequently added to the mixture. The suspension was decanted, and the remained thick oil was re-diluted with DCM (0.3 mL). Et_2O (6 mL) was then added to the mixture, and the suspension was decanted. The thick oil was dried *in vacuo* to afford airacetam-derived 3,7-di-*tert*-butyldibenzothiophenium salt **S26** (233 mg, 351 µmol, 70%) as an off white solid.

R*f* = 0.40 (DCM/MeOH, 9:1, v/v).

NMR Spectroscopy:

^{1}H NMR (500 MHz, CD_3CN, 298 K, δ): 8.19 (d, *J* = 8.4 Hz, 2H), 8.15 (d, *J* = 1.8 Hz, 2H). 7.98 (dd, *J* = 8.2, 1.8 Hz, 2H), 7.91 (dd, *J* = 8.7, 2.1 Hz, 1H), 7.37 (d, *J* = 8.8 Hz, 1H), 7.19 (d, *J* = 2.1 Hz, 1H), 4.13 (s,

3H), 3.71 (t, *J* = 7.1 Hz, 2H), 2.41 (t, *J* = 8.0 Hz, 2H), 2.00–1.96 (m, 2H), 1.38 (s, 18H) ppm.

^{13}**C NMR** (126 MHz, CD_3CN, 298 K, δ): 175.9, 167.9, 162.2, 156.3, 138.5, 138.1, 133.0, 131.3, 130.5, 130.1, 125.9, 124.9, 122.2 (q, *J* = 321.4 Hz, CF_3), 114.7, 114.1, 58.5, 47.2, 36.4, 33.7, 31.3, 18.0 ppm.

^{19}**F NMR** (471 MHz, CD_3CN, 298 K, δ): –79.0 (s) ppm.

HRMS-ESI (m/z) calculated for $C_{32}H_{36}O_3S^+$ $[M\text{-}OTf]^+$, 514.2410; found, 514.2406; deviation: 0.9 ppm.

Salicin pentaacetate-derived 2,8-dimethoxydibenzothiophenium salt S27

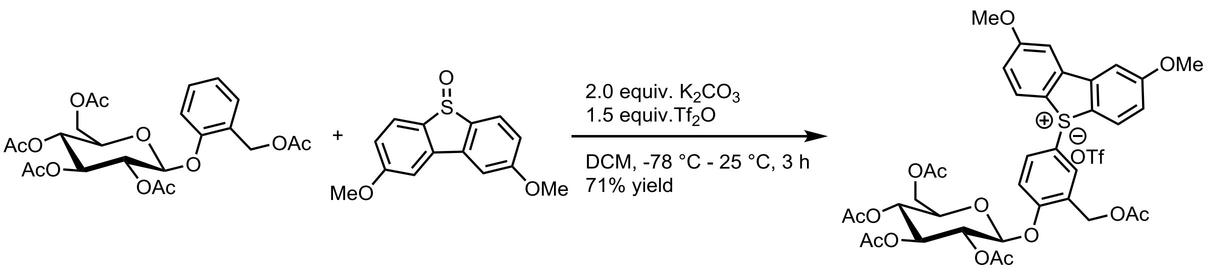

27 **M-DBTO** **S27**

A flame-dried, 10 mL nitrogen-filled *Schlenk*-tube equipped with a magnetic stir bar was charged with pyriproxyfen (**27**) (248 mg, 0.500 mmol, 1.00 equiv.), 2,8-dimethoxydibenzothiophene *S*-oxide (**M-DBTO**) (195 mg, 0.750 mmol, 1.50 equiv.), K_2CO_3 (138 mg, 1.00 mmol, 2.00 equiv.), and dry DCM (2.0 mL, c = 0.25 M) at 25 °C. After cooling to –78 °C (acetone/dry ice bath), trifluoromethanesulfonic anhydride (126 µL, 0.750 mmol, 1.50 equiv.) was added to the reaction mixture dropwise in 2 min. After addition, the reaction mixture was stirred at –78 °C for 15 min and then allowed to warm to –5 °C over 2 h. The *Schlenk*-tube was taken out of the cold bath, and the reaction mixture was allowed to warm to 25 °C in air. After stirring at 25 °C for 1 h, the reaction mixture was diluted with DCM (5.0 mL) and poured onto a mixture of DCM (20 mL) and saturated aqueous $NaHCO_3$ (10 mL). The mixture was poured into a separatory funnel, and the layers were separated. The DCM layer was collected, and the aqueous layer was further extracted with DCM (4 × ca. 30 mL). The combined DCM layer was dried over Na_2SO_4, filtered, and the solvent was removed under reduced pressure. The residue was purified by chromatography on silica gel eluting with DCM/MeOH (100:0 to 20:1, v/v). The product was collected and dried *in vacuo* to afford salicin pentaacetate-derived 2,8-dimethoxydibenzothiophenium salt **S27** (315 mg, 354 µmol, 71 %) as an orange solid.

R*f* = 0.22 (CH_2Cl_2/MeOH, 20:1, v/v).

NMR Spectroscopy:

^{1}**H NMR** (500 MHz, CD_3CN, 298 K, δ): 7.91 (dd, *J* = 8.9, 4.4 Hz, 2H), 7.81 (d, *J* = 2.6 Hz, 2H), 7.58 (dd, *J* = 9.1, 2.6 Hz, 1H), 7.47 (d, *J* = 2.6 Hz, 1H), 7.33 (d, *J* = 9.1 Hz, 1H), 7.16 (d, *J* = 9.0 Hz, 2H), 5.45 (d, *J* = 7.8 Hz, 1H), 5.37 (t, *J* = 9.5 Hz, 1H), 5.24–5.21 (m, 1H), 5.13 (t, *J* = 9.6 Hz, 1H), 4.90 (s, 2H), 4.24–4.21 (m, 1H), 4.14 –4.11 (m, 2H), 3.97 (s, 6H), 2.00 (s, 6H), 1.99 (s, 3H), 1.98 (s, 3H), 1.94 (s, 3H) ppm.

^{13}**C NMR** (126 MHz, CD_3CN, 298 K, δ): 171.2, 171.1, 170.8, 170.5, 170.4, 165.7, 159.3, 142.2, 132.9, 131.2, 130.2, 130.0 (d, *J* = 1.5 Hz), 123.8 (d, *J* = 4.8 Hz), 122.1 (q, *J* = 321.5 Hz, CF_3), 121.8, 119.3 (d, *J*

= 2.8 Hz), 117.8, 110.3 (d, *J* = 4.0 Hz), 98.4, 72.9, 72.7, 71.4, 68.9, 62.4, 60.7, 57.3, 20.9, 20.9, 20.8 ppm.

^{19}F NMR (471 MHz, CD_3CN, 298 K, δ): −78.9 (s) ppm.

HRMS-ESI (m/z) calculated for $C_{37}H_{39}O_{14}S^+$ [M-OTf], 739.2055; found, 793.2051; deviation: 0.5 ppm.

Biphenyl-derived thianthrenium salt 1-TT

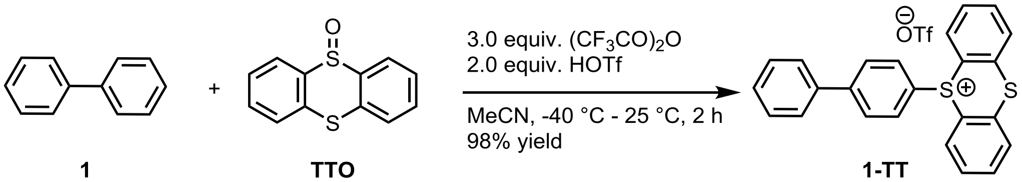

1 **TTO** **1-TT**

A flame-dried, 10 mL nitrogen-filled *Schlenk*-tube equipped with a magnetic stir bar was charged with biphenyl (**1**) (154 mg, 1.00 mmol, 1.00 equiv.) and dry MeCN (4.0 mL, c = 0.25 M) at 25 °C. After cooling to –40 °C (acetonitrile/dry ice bath), trifluoromethanesulfonic acid (177 µL, 300 mg, 2.00mmol, 2.00 equiv.) and trifluoroacetic anhydride (417 µL, 630 mg, 3.00 mmol, 3.00 equiv.) were added to the stirred reaction mixture. Subsequently, thianthrene-*S*-oxide (**TTO**) (232 mg, 1.00 mmol, 1.00 equiv.) was added to the stirred reaction mixture in small portions over 1 min. After addition, the reaction mixture was stirred at –40 °C for 1 h. Subsequently, the *Schlenk*-tube was taken out of the cold bath and warmed to 25 °C in air. After stirring at 25 °C for another 1 h, the reaction mixture was poured onto saturated aqueous $NaHCO_3$ (10 mL). The mixture was concentrated under reduced pressure to remove most of the MeCN solvent, and the residue was diluted with 20 mL DCM and 10 mL water. The mixture was poured into a separatory funnel, and the layers were separated. The DCM layer was collected, and the aqueous layer was further extracted with DCM (4 × ca. 30 mL). The combined DCM layer was dried over Na_2SO_4, filtered, and the solvent was removed under reduced pressure. The residue was purified by chromatography on silica gel eluting with DCM/MeOH (50:1 to 30:1, v/v). The product was collected and dried *in vacuo* to afford biphenyl-derived thianthrenium salt **1-TT** (510 mg, 984 µmol, 98 %) as a colorless solid.

R*f* = 0.37 (DCM/MeOH, 9:1, v/v).

NMR Spectroscopy:

^{1}H NMR (500 MHz, CD_3CN, 298 K, δ): 8.41 (d, *J* = 7.9 Hz, 2H), 7.94 (d, *J* = 7.9 Hz, 2H), 7.89 (t, *J* = 7.9 Hz, 2H), 7.83 (t, *J* = 7.6 Hz, 2H), 7.73–7.66 (m, 2H), 7.59–7.53 (m, 2H), 7.47–7.36 (m, 3H), 7.22–7.16 (m, 2H) ppm.

^{13}C NMR (126 MHz, CD_3CN, 298 K, δ): 146.4, 138.9, 137.5, 136.2, 136.1, 131.6, 131.0, 130.1, 129.9, 129.8, 129.6, 128.1, 123.3, 122.2 (q, *J* = 321.5 Hz, CF_3), 119.5 ppm.

^{19}F NMR (471 MHz, CD_3CN, 298 K, δ): –79.1 (s) ppm.

HRMS-ESI (m/z) calculated for $C_{24}H_{17}S_2^+$ [M-OTf]$^+$, 369.0766; found, 369.0761; deviation: 1.5 ppm.

Gram scale synthesis of fenofibrate-derived dibenzothiophenium salt S10

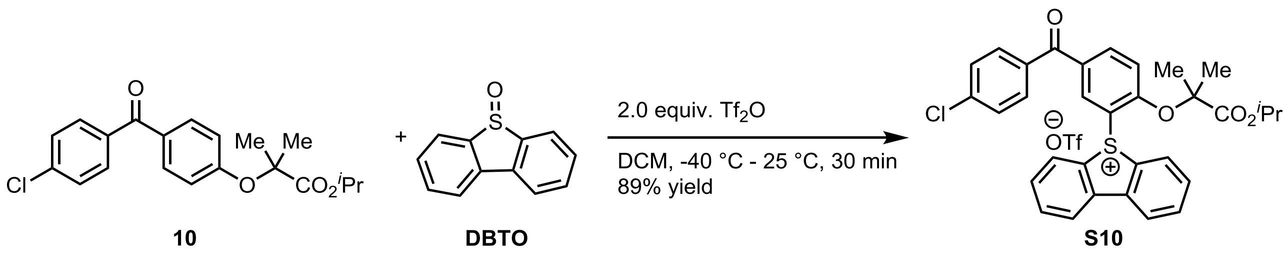

A flame-dried, 50 mL nitrogen-filled *Schlenk*-tube equipped with a magnetic stir bar was charged with fenofibrate (**10**) (1.08 g, 3.00 mmol, 1.00 equiv.), dibenzothiophene *S*-oxide (**DBTO**) (901 mg, 4.50 mmol, 1.50 equiv.), and dry DCM (12.0 mL, c = 0.25 M) at 25 °C. After cooling to –40 °C (acetonitrile/dry ice bath), trifluoromethanesulfonic anhydride (1.01 mL, 1.69 g, 6.00 mmol, 2.00 equiv.) was added dropwise over 5 min. The *Schlenk*-tube was sealed, taken out of the cold bath and warmed to 25 °C in air. After stirring at 25 °C for 30 min, the reaction mixture was diluted with DCM (30 mL) and poured onto a mixture of DCM (20 mL) and saturated aqueous $NaHCO_3$ (20 mL). The mixture was poured into a separatory funnel, and the layers were separated. The DCM layer was collected, and the aqueous layer was further extracted with DCM (4 × ca. 50 mL). The combined DCM layer was dried over Na_2SO_4, filtered, and the solvent was removed under reduced pressure. The residue was purified by chromatography on silica gel eluting with DCM/MeOH (40:1 to 20:1, v/v). The product was collected and dried *in vacuo* to afford fenofibrate-derived dibenzothiophenium salt **S10** (1.85 g, 2.67 mmol, 89 %) as a colorless solid.

Comparison of fluorination of aryl thianthrenium salt and aryl dibenzothiophenium salt

Fluorination of biphenyl-derived thianthrenium salt 1-TT

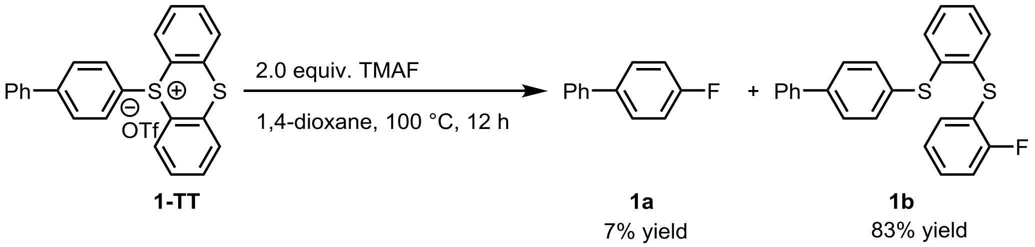

In a nitrogen-filled glovebox, a 20-mL borosilicate vial equipped with a magnetic stir bar was charged with biphenyl-derived thianthrenium salt **1-TT** (104 mg, 0.200 mmol, 1.00 equiv.) and TMAF (37.3 mg, 0.400 mmol, 2.00 equiv.). 1,4-Dioxane (4.0 mL, c = 0.05 M) was then added into the vial. The vial was sealed with a Teflon cap and taken out of the glovebox. The vial was placed in a preheated metal heating block (100 °C). After stirring at 100 °C for 12 h, the reaction mixture was allowed to cool to 25 °C in air. The reaction mixture was diluted with ethyl acetate (10 mL) and filtered through a short pad of Celite® using ethyl acetate (10 mL) as eluent. The filtrate was collected and concentrated by rotary evaporation. The residue was purified by chromatography on silica gel eluting with hexanes/ethyl acetate (100:0 to 40:1, v/v) to afford 4-fluorobiphenyl (**1a**) (2.3 mg, 13.4 μmol, 7%) as a colorless solid and [1,1'-biphenyl]-4-yl(2-((2-

fluorophenyl)thio)phenyl)sulfane (**1b**) (64.2 mg, 165 µmol, 83%) as a colorless solid.

4-Fluorobiphenyl (**1a**):

R*f* = 0.42 (hexanes).

NMR Spectroscopy:

^{1}**H NMR** (500 MHz, $CDCl_3$, 298 K, δ): 7.59–7.54 (m, 4H), 7.46 (t, *J* = 7.6 Hz, 2H), 7.38 (t, *J* = 7.4 Hz, 1H), 7.15 (*t*, *J* = 8.5 Hz, 2H) ppm.

^{13}**C NMR** (126 MHz, $CDCl_3$, 298 K, δ): 162.6 (d, *J* = 246.6 Hz), 140.4, 137.5 (d, *J* = 3.4 Hz), 128.9, 128.8 (d, *J* = 8.2 Hz), 127.4, 127.1, 115.7 (d, *J* = 21.4 Hz) ppm.

^{19}**F NMR** (471 MHz, $CDCl_3$, 298 K, δ): –115.9 (m).ppm.

HRMS-EI (m/z) calculated for $C_{12}H_9F^+$ $[M]^+$, 172.0683; found, 172.0683; deviation: –0.1 ppm.

[1,1'-Biphenyl]-4-yl(2-((2-fluorophenyl)thio)phenyl)sulfane (**1b**):

R*f* = 0.36 (hexanes/ethyl acetate, 40:1, v/v).

NMR Spectroscopy:

^{1}**H NMR** (500 MHz, $CDCl_3$, 298 K, δ): 7.59–7.52 (m, 4H), 7.45–7.36 (m, 4H), 7.35–7.22 (m, 4H), 7.18–7.03 (m, 5H) ppm.

^{13}**C NMR** (126 MHz, $CDCl_3$, 298 K, δ): 161.8 (d, *J* = 247.9 Hz), 140.4, 136.8, 134.3, 133.8, 132.4, 131.7, 130.9, 130.1 (d, *J* = 7.6 Hz), 129.0, 128.1, 128.1, 127.8, 127.6, 127.1, 125.0, 125.0, 121.5 (d, *J* = 17.7 Hz), 116.2 (d, *J* = 22.4 Hz) ppm.

^{19}**F NMR** (471 MHz, $CDCl_3$, 298 K, δ): –107.8 (m).ppm.

HRMS-EI (m/z) calculated for $C_{24}H_{17}S_2F^+$ $[M]^+$, 388.0750; found, 388.0757; deviation: –1.8 ppm.

Fluorination of biphenyl-derived dibenzothiophenium salt S1

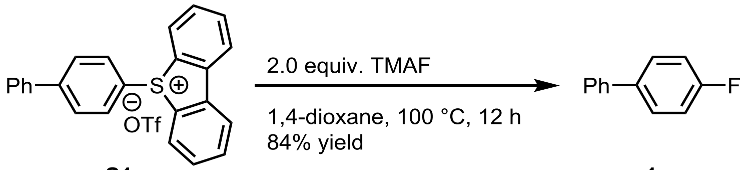

In a nitrogen-filled glovebox, a 20-mL borosilicate vial equipped with a magnetic stir bar was charged with biphenyl-derived dibenzothiophenium salt **S1** (104 mg, 0.200 mmol, 1.00 equiv.) and TMAF (37.3 mg, 0.400 mmol, 2.00 equiv.). 1,4-Dioxane (4.0 mL, c = 0.05 M) was then added into the vial. The vial was sealed with a Teflon cap and taken out of the glovebox. The vial was placed in a preheated metal heating block (100 °C). After stirring at 100 °C for 12 h, the reaction mixture was allowed to cool to 25 °C in air. The reaction mixture was evaporated to remove all violates. To separate 4-fluorobiphenyl (**1a**) from the byproduct dibenzothiophene, DCM (2.0 mL), TFA (1.0 mL), and hydrogen peroxide solution (35 wt. % in H_2O, 0.40 mL)

were added in sequence to the residue. After stirring at 25 °C for 20 min, the reaction mixture was diluted with DCM (20 mL) and H_2O (10 mL). The organic phase was separated and the aqueous phase was extracted with DCM (4 × 20 mL). The combined organic phase was washed with saturated brine (20 mL), dried over Na_2SO_4, filtered, and the solvent was removed under reduced pressure. The residue was purified by chromatography on silica gel eluting with hexanes to afford 4-fluorobiphenyl (**1a**) (29.0 mg, 168 µmol, 84%) as a colorless solid.

Fluorination of aryl dibenzothiophenium salts

6-Fluoro-1,3-dimethylquinazoline-2,4(1*H*,3*H*)-dione (S28)

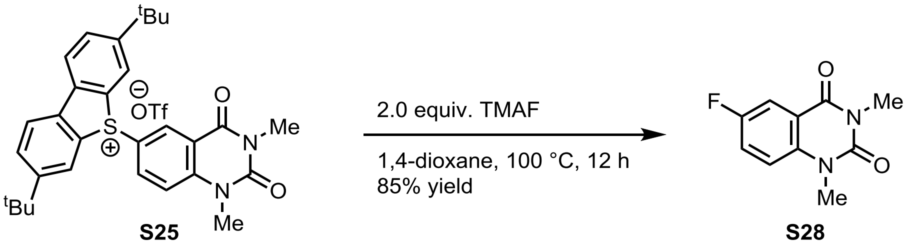

S25 **S28**

In a nitrogen-filled glovebox, a 4-mL borosilicate vial equipped with a magnetic stir bar was charged with 1,3-dimethylquinazoline-2,4(1*H*,3*H*)-dione-derived 3,7-di-*tert*-butyldibenzothiophenium salt **S25** (63.5 mg, 0.100 mmol, 1.00 equiv.) and TMAF (18.6 mg, 0.200 mmol, 2.00 equiv.). 1,4-Dioxane (2.0 mL, c = 0.05 M) was then added into the vial. The vial was sealed with a Teflon cap and taken out of the glovebox. The vial was placed in a preheated metal heating block (100 °C). After stirring at 100 °C for 12 h, the reaction mixture was allowed to cool to 25 °C in air. The reaction mixture was diluted with ethyl acetate (1.0 mL) and filtered through a short pad of Celite® using ethyl acetate (10 mL) as eluent. The filtrate was collected and concentrated by rotary evaporation. The residue was purified by chromatography on silica gel eluting with hexanes/ethyl acetate (2:1, v/v) to afford 6-fluoro-1,3-dimethylquinazoline-2,4(1*H*,3*H*)-dione (**S28**) (17.7 mg, 85.0 µmol, 85%) as a colorless solid.

R*f* = 0.26 (hexanes/ethyl acetate, 2:1, v/v).

NMR Spectroscopy:

^{1}**H NMR** (500 MHz, $CDCl_3$, 298 K, δ): 7.87 (dd, *J* = 8.2, 3.0 Hz, 1H), 7.43–7.35 (m, 1H), 7.17 (dd, *J* = 9.2, 3.9 Hz, 1H), 3.59 (s, 3H), 3.47 (s, 3H) ppm.

^{13}**C NMR** (126 MHz, $CDCl_3$, 298 K, δ): 161.2 (d, *J* = 2.9 Hz), 158.4 (d, *J* = 244.4 Hz), 151.0, 137.0, 122.8 (d, *J* = 23.8 Hz), 116.8 (d, *J* = 7.9 Hz), 115.5 (d, *J* = 7.6 Hz), 114.5 (d, *J* = 24.1 Hz), 31.2, 28.8 ppm.

^{19}**F NMR** (471 MHz, $CDCl_3$, 298 K, δ): –119.2 (m) ppm.

HRMS-EI (m/z) calculated for $C_{10}H_9N_2O_2F^+$ $[M]^+$, 208.0643; found, 208.0643; deviation: –0.4 ppm.

Fluorodiflunisal derivative S29

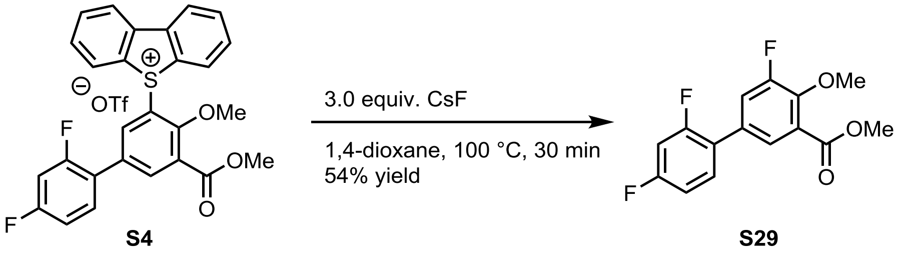

In a nitrogen-filled glovebox, a 20-mL borosilicate vial equipped with a magnetic stir bar was charged with diflunisal derivative-derived dibenzothiophenium salt **S4** (122 mg, 0.200 mmol, 1.00 equiv.) and CsF (91.1 mg, 0.600 mmol, 3.00 equiv.). 1,4-Dioxane (4.0 mL, c = 0.05 M) was then added into the vial. The vial was sealed with a Teflon cap and taken out of the glovebox. The vial was placed in a preheated metal heating block (100 °C). After stirring at 100 °C for 30 min, the reaction mixture was allowed to cool to 25 °C in air. The reaction mixture was diluted with ethyl acetate (10 mL) and filtered through a short pad of Celite® using ethyl acetate (10 mL) as eluent. The filtrate was collected and concentrated by rotary evaporation. The residue was purified by chromatography on silica gel eluting with hexanes/ethyl acetate (20:1, v/v) to afford fluorodiflunisal derivative **S29** (31.9 mg, 108 µmol, 54%) as a colorless solid.

R*f* = 0.17 (hexanes/ethyl acetate, 20:1, v/v).

NMR Spectroscopy:

^{1}**H NMR** (500 MHz, $CDCl_3$, 298 K, δ): 7.67 (d, *J* = 2.3 Hz, 1H), 7.43 (t, *J* = 1.9 Hz, 1H), 7.39 (dd, J = 8.6, 1.3 Hz, 1H), 6.96 (dt, *J* = 8.3, 2.0 Hz, 1H), 6.92 (t, *J* = 2.2 Hz, 1H), 4.03 (s, 3H), 3.93 (s, 3H) ppm.

^{13}**C NMR** (126 MHz, $CDCl_3$, 298 K, δ): 165.7 (d, *J* = 4.1 Hz), 162.8 (dd, *J* = 250.5, 12.0 Hz), 159.8 (dd, J = 251.1, 12.1 Hz), 156.0 (d, *J* = 248.1 Hz), 147.3 (d, *J* = 12.5 Hz), 131.3 (dd, *J* = 9.6, 4.5 Hz), 130.6 (d, *J* = 7.7 Hz), 126.7 (d, *J* = 2.9 Hz), 126.6 (t, *J* = 2.9 Hz), 123.1 (d, *J* = 13.6 Hz), 120.9 (dd, *J* = 20.7, 3.8 Hz), 112.0 (dd, *J* = 21.4, 3.9 Hz), 104.7 (t, *J* = 25.9 Hz), 62.4 (d, *J* = 5.2 Hz), 52.6 ppm.

^{19}**F NMR** (471 MHz, $CDCl_3$, 298 K, δ): –109.9 (m), –113.3 (m), –128.8 (d, *J* = 12.1 Hz) ppm.

HRMS-ESI (m/z) calculated for $C_{15}H_{11}O_3F_3Na^+$ $[M+Na]^+$, 319.0553; found, 319.0548; deviation: 1.4 ppm.

Fluoroclofibrate (S30)

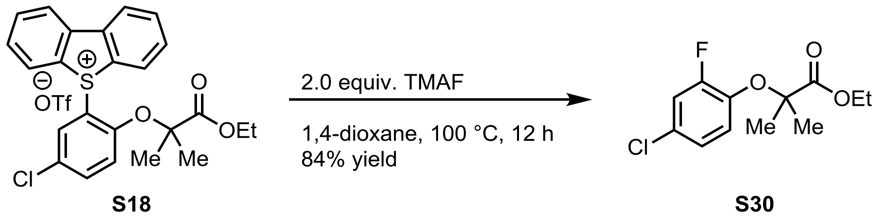

In a nitrogen-filled glovebox, a 20-mL borosilicate vial equipped with a magnetic stir bar was charged with clofibrate-derived dibenzothiophenium salt **S18** (115 mg, 0.200 mmol, 1.00 equiv.) and TMAF (37.3 mg, 0.400 mmol, 2.00 equiv.). 1,4-Dioxane (4.0 mL, c = 0.05 M) was then added into the vial. The vial was sealed with a

Teflon cap and taken out of the glovebox. The vial was placed in a preheated metal heating block (100 °C). After stirring at 100 °C for 12 h, the reaction mixture was allowed to cool to 25 °C in air. The reaction mixture was diluted with ethyl acetate (10 mL) and filtered through a short pad of Celite® using ethyl acetate (10 mL) as eluent. The filtrate was collected and concentrated by rotary evaporation. The residue was purified by chromatography on silica gel eluting with hexanes/ethyl acetate (50:1 to 25:1, v/v) to afford fluoroclofibrate (**S30**) (44.0 mg, 169 µmol, 84%) as a colorless oil.

R*f* = 0.35 (hexanes/ethyl acetate, 20:1, v/v).

NMR Spectroscopy:

^{1}**H NMR** (500 MHz, $CDCl_3$, 298 K, δ): 7.11–7.05 (m, 1H), 7.01–6.88 (m, 2H), 4.25–4.20 (m, 2H), 1.55 (s, 6H), 1.29–1.24 (m, 3H) ppm.

^{13}**C NMR** (126 MHz, $CDCl_3$, 298 K, δ): 173.5, 155.1 (d, *J* = 250.4 Hz), 142.0 (d, *J* = 11.3 Hz), 128.3 (d, *J* = 9.3 Hz), 124.3 (d, *J* = 3.7 Hz), 123.5 (d, *J* = 1.8 Hz), 117.3 (d, *J* = 22.9 Hz), 81.4, 61.7, 25.0, 14.2 ppm.

^{19}**F NMR** (471 MHz, $CDCl_3$, 298 K, δ): –126.1 (t, *J* = 9.4 Hz).ppm.

HRMS-ESI (m/z) calculated for $C_{12}H_{14}O_3ClFNa^+$ $[M+Na]^+$, 283.0508; found, 283.0505; deviation: 1.0 ppm.

3-Chloro-9-fluoro-6-methyldibenzo[*c*,*f*][1,2]thiazepin-11(6*H*)-one 5,5-dioxide (S31)

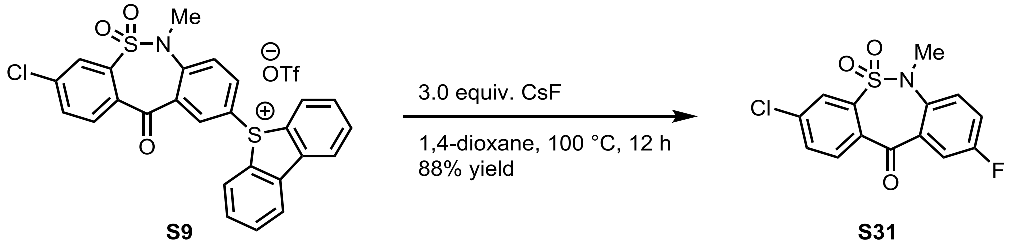

S9 **S31**

In a nitrogen-filled glovebox, a 20-mL borosilicate vial equipped with a magnetic stir bar was charged with 3-chloro-6-methyldibenzo[*c*,*f*][1,2]thiazepin-11(6*H*)-one 5,5-dioxide-derived dibenzothiophenium salt **S9** (128 mg, 0.200 mmol, 1.00 equiv.) and CsF (91.1 mg, 0.600 mmol, 3.00 equiv.). 1,4-Dioxane (4.0 mL, c = 0.05 M) was then added into the vial. The vial was sealed with a Teflon cap and taken out of the glovebox. The vial was placed in a preheated metal heating block (100 °C). After stirring at 100 °C for 12 h, the reaction mixture was allowed to cool to 25 °C in air. The reaction mixture was diluted with ethyl acetate (10 mL) and filtered through a short pad of Celite® using ethyl acetate (10 mL) as eluent. The filtrate was collected and concentrated by rotary evaporation. The residue was purified by chromatography on silica gel eluting with hexanes/ethyl acetate (10:1, v/v) to afford 3-chloro-9-fluoro-6-methyldibenzo[*c*,*f*][1,2]thiazepin-11(6*H*)-one 5,5-dioxide **S31** (57.6 mg, 177 µmol, 88%) as a colorless solid.

R*f* = 0.19 (hexanes/ethyl acetate, 9:1, v/v).

NMR Spectroscopy:

^{1}**H NMR** (500 MHz, CD_2Cl_2, 298 K, δ): 7.98–7.94 (m, 2H), 7.92 (d, *J* = 8.3 Hz, 1H), 7.72 (dd, *J* = 8.3, 2.1

Hz, 1H), 7.42–7.37 (m, 2H), 3.30 (s, 3H) ppm.

^{13}C NMR (126 MHz, CD_2Cl_2, 298 K, δ): 188.7(d, *J* = 1.2 Hz), 160.7 (d, *J* = 246.9 Hz), 139.4, 138.5, 134.0, 133.9 (d, *J* = 4.1 Hz), 133.0 (d, *J* = 6.6 Hz), 127.6 (d, *J* = 8.1 Hz), 126.1, 122.5 (d, *J* = 23.7 Hz), 118.0 (d, *J* = 25.0 Hz), 39.6 ppm.

^{19}F NMR (471 MHz, CD_2Cl_2, 298 K, δ): –115.5 (m) ppm.

HRMS-ESI (m/z) calculated for $C_{14}H_9O_3FClSNa^+$ $[M+Na]^+$, 347.9868; found, 347.9866; deviation: 0.6 ppm.

Fluorodicamba methylester (S32)

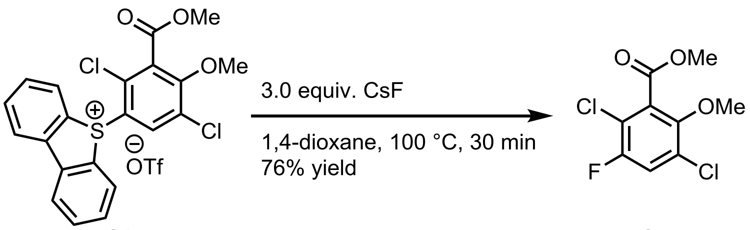

S17 **S32**

In a nitrogen-filled glovebox, a 20-mL borosilicate vial equipped with a magnetic stir bar was charged with dicamba methylester-derived dibenzothiophenium salt **S17** (113 mg, 0.200 mmol, 1.00 equiv.) and CsF (91.1 mg, 0.600 mmol, 3.00 equiv.). 1,4-Dioxane (4.0 mL, c = 0.05 M) was then added into the vial. The vial was sealed with a Teflon cap and taken out of the glovebox. The vial was placed in a preheated metal heating block (100 °C). After stirring at 100 °C for 30 min, the reaction mixture was allowed to cool to 25 °C in air. The reaction mixture was diluted with ethyl acetate (10 mL) and filtered through a short pad of Celite® using ethyl acetate (10 mL) as eluent. The filtrate was collected and concentrated by rotary evaporation. The residue was purified by chromatography on silica gel eluting with hexanes/ethyl acetate (25:1, v/v) to afford fluorodicamba methylester (**S32**) (38.4 mg, 152 µmol, 76%) as a colorless oil.

R*f* = 0.28 (hexanes/ethyl acetate, 25:1, v/v).

NMR Spectroscopy:

^{1}H NMR (500 MHz, $CDCl_3$, 298 K, δ): 7.26 (d, *J* = 8.3 Hz, 1H), 3.97 (s, 3H), 3.87 (s, 3H) ppm.

^{13}C NMR (126 MHz, $CDCl_3$, 298 K, δ): 164.1 (d, *J* = 3.4 Hz), 154.3 (d, *J* = 249.9 Hz), 150.1 (d, *J* = 4.0 Hz), 131.3, 127.4 (d, *J* = 8.9 Hz), 119.0 (d, *J* = 25.1 Hz), 117.4 (d, *J* = 20.8 Hz), 62.6, 53.0 ppm.

^{19}F NMR (471 MHz, $CDCl_3$, 298 K, δ): –116.8 (d, *J* = 8.2 Hz).ppm.

HRMS-ESI (m/z) calculated for $C_9H_7O_3Cl_2FNa^+$ $[M+Na]^+$, 274.9648; found, 274.9646; deviation: 0.8 ppm.

Fluorobifonazole (S33)

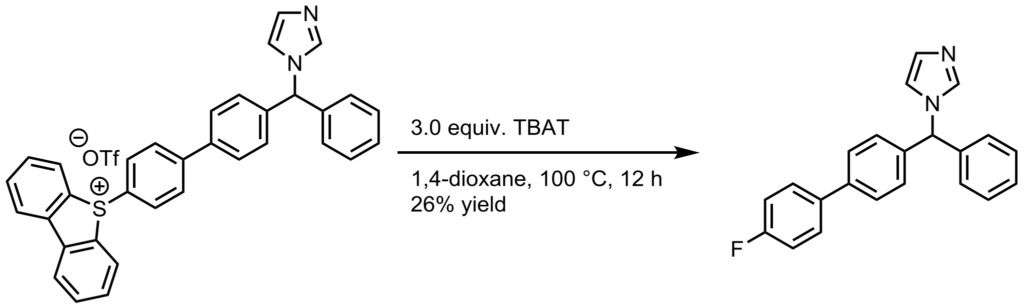

S19 **S33**

In a nitrogen-filled glovebox, a 4-mL borosilicate vial equipped with a magnetic stir bar was charged with bifonazole-derived dibenzothiophenium salt **S19** (64.3 mg, 0.100 mmol, 1.00 equiv.) and TBAT (162 mg, 0.300 mmol, 3.00 equiv.). 1,4-Dioxane (2.0 mL, c = 0.05 M) was then added into the vial. The vial was sealed with a Teflon cap and taken out of the glovebox. The vial was placed in a preheated metal heating block (100 °C). After stirring at 100 °C for 12 h, the reaction mixture was allowed to cool to 25 °C in air. The reaction mixture was diluted with ethyl acetate (1.0 mL) and filtered through a short pad of Celite® using ethyl acetate (10 mL) as eluent. The filtrate was collected and concentrated by rotary evaporation. The residue was purified by chromatography on silica gel eluting with DCM/MeOH (100:1, v/v) to afford fluorobifonazole with impurities. The mixture was further purified by HPLC (YMC-Actus Triart C18 (30×150 mm: 5 µm), 40:60 MeCN/TFA in water (5/1000, v/v), flow rate = 42.5 mL/min, 35 ºC) to afford fluorobifonazole·TFA salt. The salt was then dissolved in 10 mL DCM, and 2.0 mL saturated $NaHCO_3$ was added. The mixture was then poured into a separatory funnel, and the layers were separated. The DCM layer was collected, and the aqueous layer was extracted with DCM (2 × ca. 10 mL). The combined DCM layer was dried over Na_2SO_4, filtered, and the solvent was removed under reduced pressure to afford fluorobifonazole (**S33**) (8.4 mg, 25.6 µmol, 26%) as a colorless solid.

R*f* = 0.39 (DCM/ethyl acetate, 1:1, v/v).

NMR Spectroscopy:

^{1}H NMR (500 MHz, CD_2Cl_2, 298 K, δ): 7.62–7.53 (m, 4H), 7.46–7.33 (m, 4H), 7.24–7.11 (m, 6H), 7.06 (s, 1H), 6.92 (s, 1H), 6.58 (s, 1H) ppm.

^{13}C NMR (126 MHz, CD_2Cl_2, 298 K, δ): 163.0 (d, *J* = 246.5 Hz), 140.5, 139.7, 138.8, 137.7 (brs), 136.9 (d, *J* = 3.2 Hz), 129.5, 129.3, 129.1 (d, *J* = 8.2 Hz), 129.0, 128.8, 128.4, 127.7, 119.7 (brs), 116.0 (d, *J* = 21.5 Hz), 65.1 ppm.

^{19}F NMR (471 MHz, CD_2Cl_2, 298 K, δ): –116.0 (m) ppm.

HRMS-ESI (m/z) calculated for $C_{22}H_{18}N_2F^+$ [M+H]$^+$, 329.1449; found, 329.1444; deviation: 0.9 ppm.

Fluorofenofibrate (S37)

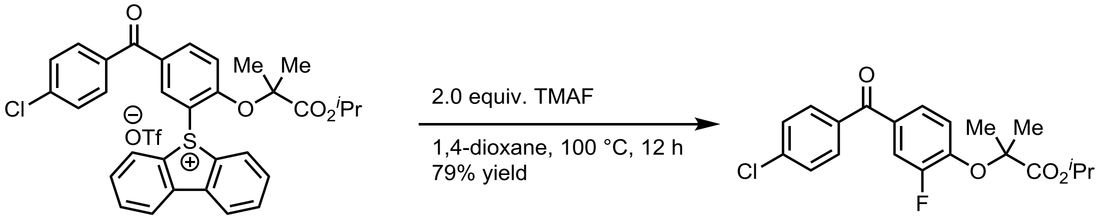

S10 **S37**

In a nitrogen-filled glovebox, a 20-mL borosilicate vial equipped with a magnetic stir bar was charged with fenofibrate-derived dibenzothiophenium salt **S10** (139 mg, 0.200 mmol, 1.00 equiv.) and TMAF (37.3 mg, 0.400 mmol, 2.00 equiv.). 1,4-Dioxane (4.0 mL, c = 0.05 M) was then added into the vial. The vial was sealed with a Teflon cap and taken out of the glovebox. The vial was placed in a preheated metal heating block (100 °C). After stirring at 100 °C for 12 h, the reaction mixture was allowed to cool to 25 °C in air. The reaction mixture was diluted with ethyl acetate (10 mL) and filtered through a short pad of Celite® using ethyl acetate (10 mL) as eluent. The filtrate was collected and concentrated by rotary evaporation. The residue was purified by chromatography on silica gel eluting with hexanes/ethyl acetate (20:1 to 8:1, v/v). The product was collected and dried *in vacuo* to afford fluorofenofibrate (**S37**) (59.5 mg, 157 µmol, 79%) as a colorless solid.

R*f* = 0.36 (hexanes/ethyl acetate, 8:1, v/v).

NMR Spectroscopy:

^{1}H NMR (500 MHz, $CDCl_3$, 298 K, δ): 7.72–7.64 (m, 2H), 7.56 (dd, *J* = 11.4, 2.2 Hz, 1H), 7.50–7.39 (m, 3H), 6.92 (t, *J* = 8.2 Hz, 1H), 5.12–5.05 (m, 1H), 1.65 (s, 6H), 1.22 (d, *J* = 6.3 Hz, 6H) ppm.

^{13}C NMR (126 MHz, $CDCl_3$, 298 K, δ): 193.3 (d, *J* = 1.6 Hz), 172.7, 153.6 (d, *J* = 249.1 Hz), 147.9 (d, *J* = 10.8 Hz), 138.9, 135.9, 131.4 (d, *J* = 5.4 Hz), 131.3, 128.8, 126.6 (d, *J* = 3.3 Hz), 119.2, 118.3 (d, *J* = 20.5 Hz), 81.1, 69.5, 25.3, 21.7 ppm.

^{19}F NMR (471 MHz, $CDCl_3$, 298 K, δ): –129.4 (m) ppm.

HRMS-ESI (m/z) calculated for $C_{20}H_{20}O_4ClFNa^+$ $[M+Na]^+$, 401.0926; found, 401.0926; deviation: 0.2 ppm.

Comparison of fluorination of aryl dibenzothiophenium salts derived from DBTO and B-DBTO

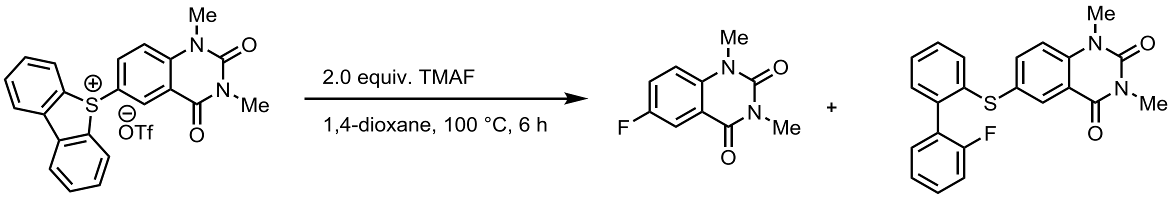

S25-DBT **S28** **S28a**

55% ^{19}F NMR yield 42% ^{19}F NMR yield

In a nitrogen-filled glovebox, a 20-mL borosilicate vial equipped with a magnetic stir bar was charged with

1,3-dimethylquinazoline-2,4(1*H*,3*H*)-dione-derived dibenzothiophenium salt **S25-DBT** (52.3 mg, 0.100 mmol, 1.00 equiv.) and TMAF (18.6 mg, 0.200 mmol, 2.00 equiv.). 1,4-Dioxane (2.0 mL, c = 0.05 M) was then added into the vial. The vial was sealed with a Teflon cap and taken out of the glovebox. The vial was placed in a preheated metal heating block (100 °C). After stirring at 100 °C for 6 h, the reaction mixture was allowed to cool to 25 °C in air. 1,4-Difluorobenzene (11.4 mg, 0.100 mmol, 1.0 equiv) was added as an internal standard. The yield (55%) of **S28** and the yield (42%) of **S28a** were determined by ^{19}F NMR integration relative to the internal standard.

Figure S01. Mass spectrum of product **S28** peak from GCMS

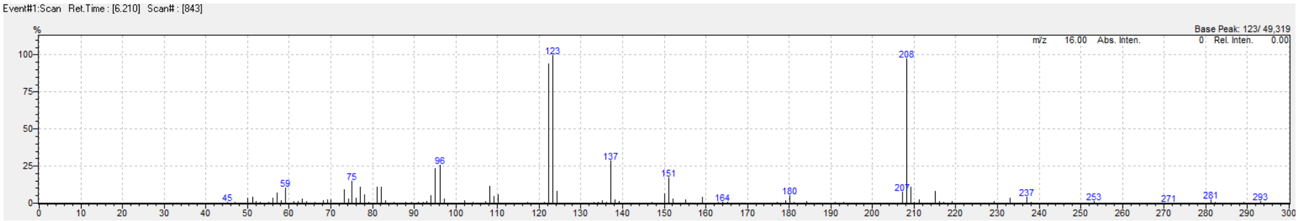

Figure S02. Mass spectrum of product **S28a** peak from LCMS

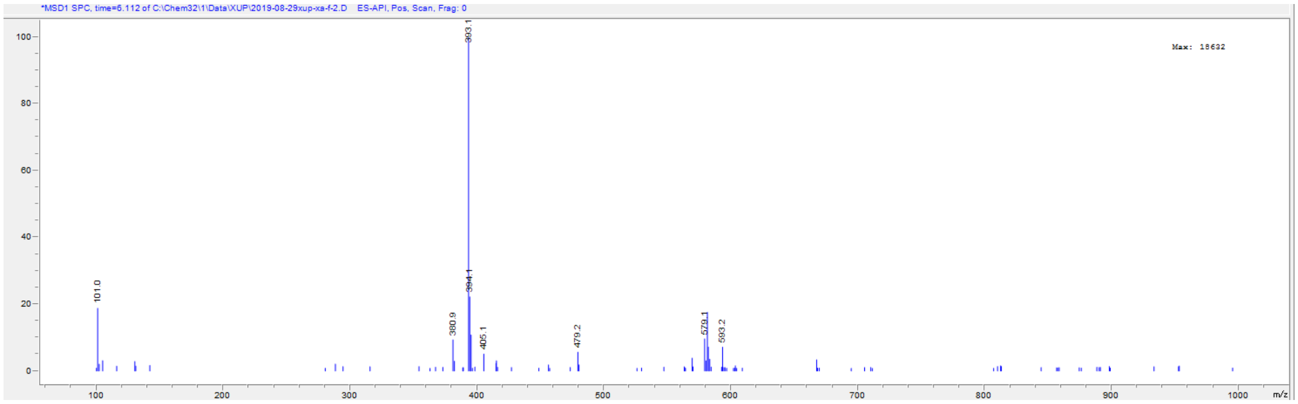

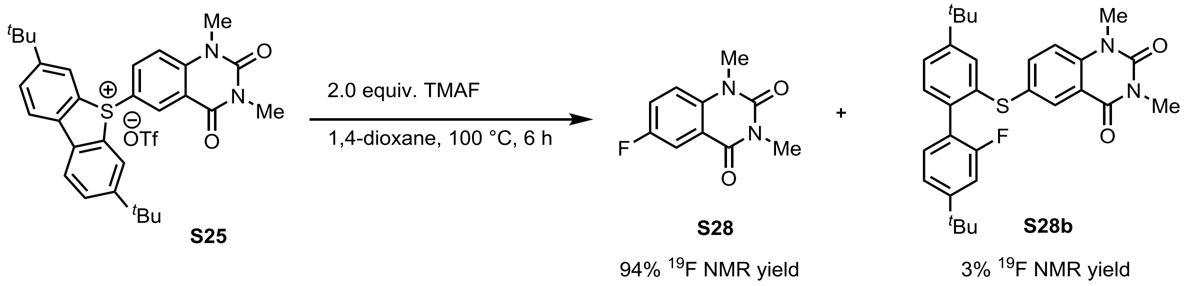

In a nitrogen-filled glovebox, a 20-mL borosilicate vial equipped with a magnetic stir bar was charged with 1,3-dimethylquinazoline-2,4(1*H*,3*H*)-dione-derived 3,7-di-*tert*-butyldibenzothiophenium salt **S25** (63.5 mg, 0.100 mmol, 1.00 equiv.) and TMAF (18.6 mg, 0.200 mmol, 2.00 equiv.). 1,4-Dioxane (2.0 mL, c = 0.05 M) was then added into the vial. The vial was sealed with a Teflon cap and taken out of the glovebox. The vial was placed in a preheated metal heating block (100 °C). After stirring at 100 °C for 6 h, the reaction mixture

was allowed to cool to 25 °C in air. 1,4-Difluorobenzene (11.4 mg, 0.100 mmol, 1.0 equiv) was added as an internal standard. The yield (94%) of **S28** and the yield (3%) of **S28b** were determined by ^{19}F NMR integration relative to the internal standard.

Figure S03. Mass spectrum of product **S28** peak from GCMS

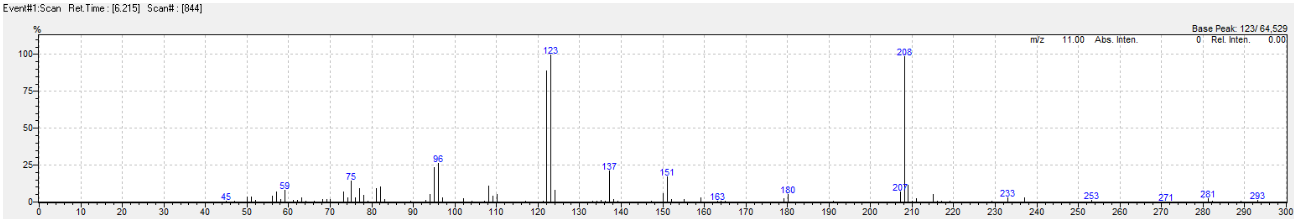

Figure S04. Mass spectrum of product **S28b** peak from LCMS

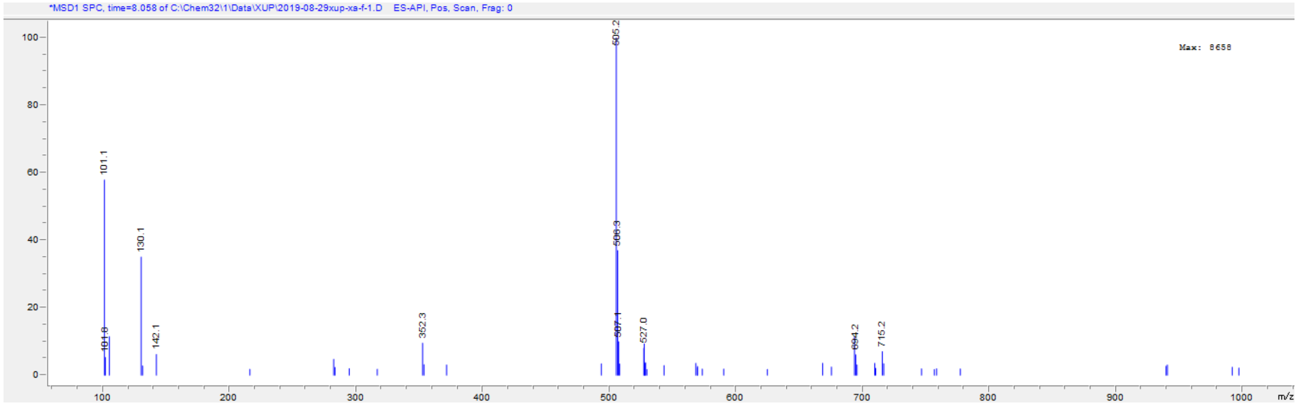

Radiochemistry

General information

No-carrier-added [^{18}F]fluoride was purchased from Advanced Accelerator Applications SA. Liquid chromatographic (LC) analysis was performed with Thermo Scientific Dionex UltiMate 3000 dual channel HPLC system connected to LabLogic NaI/PMT-radiodetectors with Flow-Ram output. A Thermo Scientific™ Accucore™ XL C18, 4 µm, 3×150 mm HPLC column was used for analytic HPLC. Thin-layer chromatography was performed on Macherey-Nagel POLYGRAM SIL G/UV254 TLC plates. Radio-TLC analysis was performed on a LabLogic ScanRAM equipped with plastic/PMT detector.

Solvents for radiochemical experiments: Anhydrous acetonitrile was obtained from Phoenix Solvent Drying Systems. Methanol (absolute, >99.8%), DMSO (ACS reagent >99.9%) were purchased from SigmaAldrich® and used as received. Water was obtained from a Millipore Milli-Q Integral Water Purification System.

HPLC Gradient [Analytical HPLC used the following mobile phases: 0.1% TFA in acetonitrile (A), 0.1% TFA in water (B)]:

0-2 mins (5% A, 95% B) isocratic

2-10 mins (5% A to 95% A) linear increase

10-15 mins (95% A, 5% B) isocratic

15-20 mins (95% A to 5% A) linear decrease

20-22 mins (5% A, 95% B) isocratic

All ^{18}F-labeled molecules were characterized by comparing the HPLC radio-trace of the isolated compound to the HPLC UV-trace of an authentic reference sample. Radioactivity was measured in a Veenstra Instruments, VIK-203 ionization-chamber.

General procedure for pre-conditioning of QMA cartridges

Unless otherwise stated, Chromafix PS-HCO_3 ^{18}F separation cartridge (45 mg) (Product No. 731876 from ABX) was pre-conditioned by sequentially pushing aqueous potassium oxalate solution (3.0 mL, 10mg/mL) and water (2.0 mL) through the cartridge.

General procedure for ^{18}F-fluorination

Aqueous [^{18}F]fluoride solution (20–30 MBq) was loaded with a syringe onto a QMA anion-exchange cartridge that was pre-conditioned according to the general procedure, and then the cartridge was washed with anhydrous MeCN (1.0 mL) and air-dried (2.0 mL). The [^{18}F]fluoride was eluted from the cartridge with a methanol solution (500 µL) of precursor into a 5 mL borosilicate vial (with a stir bar), followed by anhydrous MeCN (500 µL) and air-dried (1.0 mL). The V-vial was heated to 90 °C under a stream of nitrogen until all of the solvent was evaporated. The evaporation process was then repeated twice after addition of anhydrous MeCN (1.0 mL) each time. Approximate azeotropically drying time is 4 min in total. Anhydrous MeCN (500 µL) was added to the V-vial. The V-vial was sealed with a Teflon-lined cap and stirred at a preheated metal heating block (110 °C) for 20 min. The vial was removed from the hot plate before the addition of water and MeCN (500 µL, 1:1, v/v). An aliquot was removed and used for radio-TLC and radio-HPLC analysis. The product identity and purity was determined by comparison of the HPLC radio-trace with the HPLC UV-trace of the authentic reference sample, which was either synthesized as a new or known compound or purchased from a commercial source.

Elution efficiency (EE) was calculated as following:

$$EE = \frac{\text{total activity of the reaction mixture after elution of [}^{18}\text{F]-fluoride into a vial}}{\text{activity of cartridge after loading of aqueous [}^{18}\text{F]-fluoride solution onto the cartridge}}$$

Radiochemical conversion (RCC) was determined by dividing the integrated area under the [^{18}F]fluoroarene peak on radio-TLC trace by the total integrated area of all peaks on the radio-TLC trace.

The **radiochemical purity** was determined by dividing the integrated area under the [^{18}F]fluoroarene peak on radio-HPLC chromatogram by the total integrated area of all non-[^{18}F]-flouride peaks on the radio-HPLC chromatogram.

The analytical **radiochemical yield** (RCY) was calculated as following:

RCY = [elution efficiency]×[radiochemical conversion]×[radiochemical purity]×100%

Reaction optimization

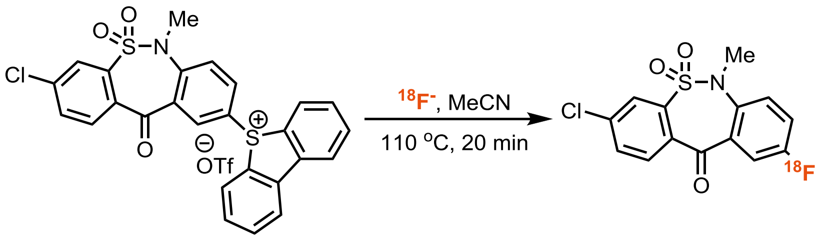

S9 **31**

Table S01. Reaction optimization.

Change of reaction conditions	EE (%)	RCY (%)
none	85	48
elution with pure MeOH	0	0
elution with Kryptofix 222 & K_2CO_3	78	37
2.3 mg of **S9** instead of 5.5 mg	77	40
1,4-dioxane as solvent	83	1
90 °C	82	43

6-[^{18}F]Fluoro-1,3-dimethylquinazoline-2,4(1*H*,3*H*)-dione (28)

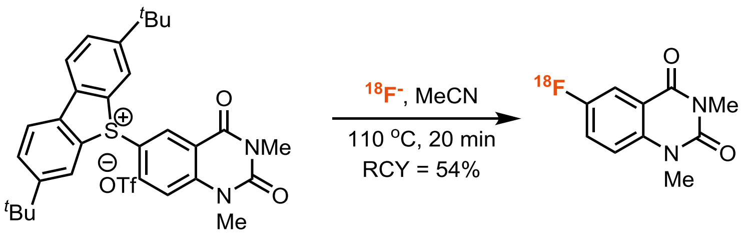

S25 **28**

Aqueous [^{18}F]fluoride solution (20–30 MBq) was loaded with a syringe onto a QMA anion-exchange cartridge that was pre-conditioned according to the general procedure, and then the cartridge was washed with anhydrous MeCN (1.0 mL) and air-dried (2.0 mL). The [^{18}F]fluoride was eluted from the cartridge with a methanol solution (500 µL) of 1,3-dimethylquinazoline-2,4(1*H*,3*H*)-dione-derived 3,7-di-*tert*-butyldibenzothiophenium salt **S25** (5.8 mg, 9.0 µmol) into a 5 mL borosilicate vial (with a stir bar), followed by anhydrous MeCN (500 µL) and air-dried (1.0 mL). The V-vial was heated to 90 °C under a stream of nitrogen until all of the solvent was evaporated. The evaporation process was then repeated twice with the addition of anhydrous MeCN (1.0 mL) each time. After evaporation, anhydrous MeCN (500 µL) was added to the V-vial. The V-vial was sealed with a Teflon-lined cap and stirred at a preheated metal heating block (110 °C) for 20 min. The vial was removed from the hot plate before the addition of water and MeCN (500 µL, 1:1, v/v). An

aliquot was removed and used for radio-TLC and radio-HPLC analysis. The product identity and purity was determined by comparison of the HPLC radio-trace with the HPLC UV-trace of the authentic reference sample 6-fluoro-1,3-dimethylquinazoline-2,4(1*H*,3*H*)-dione (**S28**)

Figure S05. Radio-HPLC trace of the ^{18}F-fluorination reaction yielding **28**.

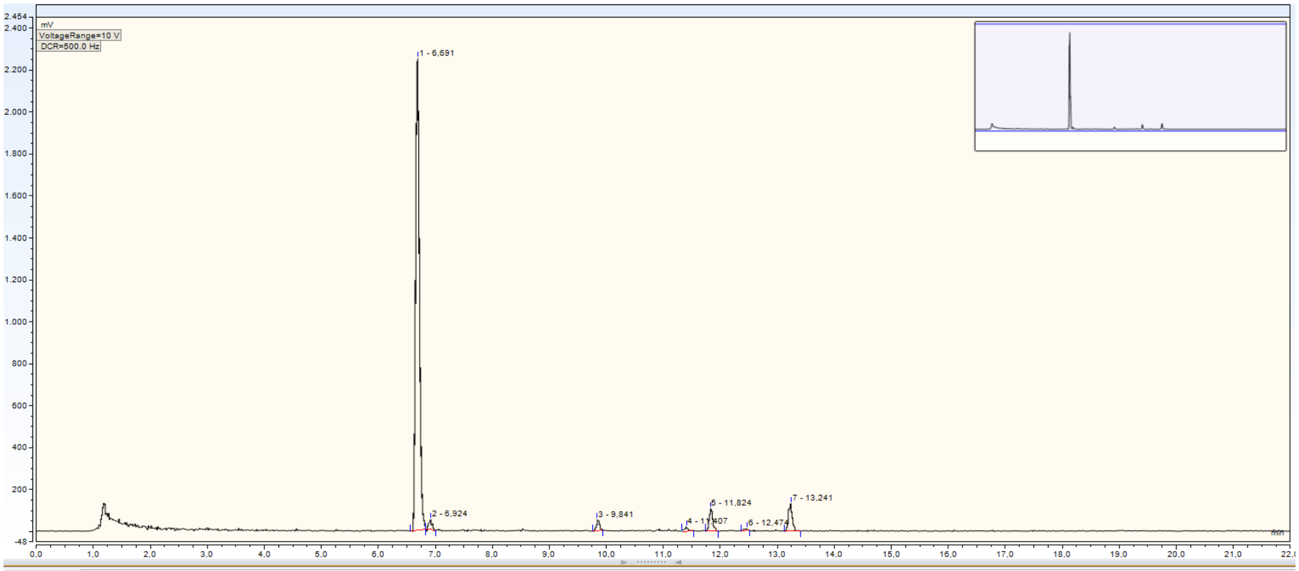

D15

	A	B	C	D	E	F	G	H	I
1	No.	Peakname	Ret.Time	Area	Amount	Type	Height	Rel.Area	Resolution
2			min	mV*min	n.a.		mV	%	
4	1		6,691	175,7685	n.a.	BMB*	2248,880	87,20	2,36
5	2		6,924	2,8219	n.a.	BMB*	47,808	1,40	29,52
6	3		9,841	3,0859	n.a.	BMB*	52,254	1,53	12,55
7	4		11,407	1,6681	n.a.	MB*	21,349	0,83	3,35
8	5		11,824	7,3891	n.a.	BMB*	105,267	3,67	5,11
9	6		12,474	0,6242	n.a.	BMB*	7,444	0,31	6,00
10	7		13,241	10,2209	n.a.	BMB*	131,603	5,07	n.a.
11	Total:			201,5785	0,0000		2614,604	100,00	

Figure S06. HPLC UV-trace of **S28** as the reference.

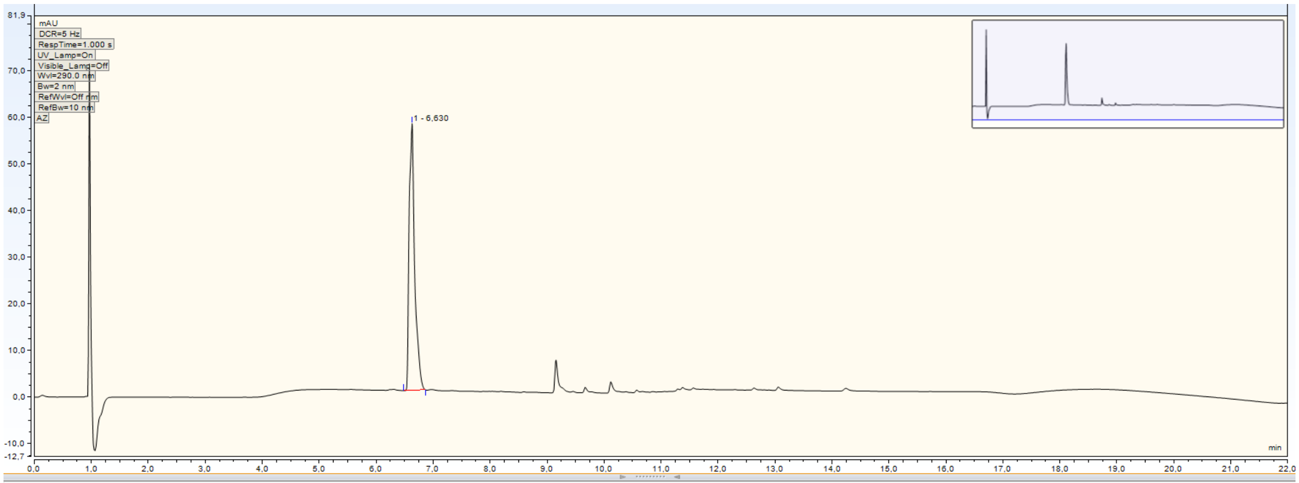

G18

	A	B	C	D	E	F	G	H	I
1	No.	Peakname	Ret.Time	Area	Amount	Type	Height	Rel.Area	Resolution
2			min	mAU*min	n.a.		mAU	%	
4	1		6,630	6,4376	n.a.	BMB*	57,207	100,00	n.a.
6	Total:			6,4376	0,0000		57,207	100,00	

Table S02. Radiochemical yield of **28**.

Reaction	EE	RCC (Radio-TLC)	Radio-HPLC	RCY
1	88%	73%	90%	58%
2	89%	65%	87%	50%
3	86%	69%	92%	55%
average RCY				54 ± 4%

[^{18}F]Fluorodiflunisal derivative 29

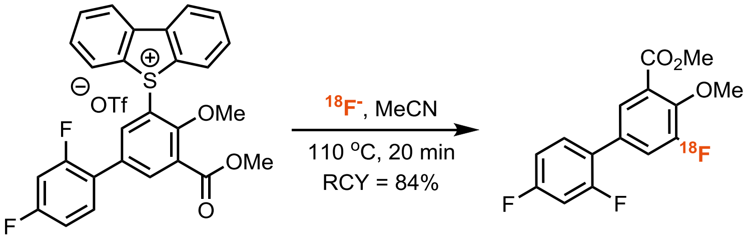

Aqueous [^{18}F]fluoride solution (20–30 MBq) was loaded with a syringe onto a QMA anion-exchange cartridge that was pre-conditioned according to the general procedure, and then the cartridge was washed with anhydrous MeCN (1.0 mL) and air-dried (2.0 mL). The [^{18}F]fluoride was eluted from the cartridge with a methanol solution (500 µL) of diflunisal derivative-derived dibenzothiophenium salt **S4** (5.5 mg, 9.0 µmol) into a 5 mL borosilicate vial (with a stir bar), followed by anhydrous MeCN (500 µL) and air-dried (1.0 mL). The V-vial was heated to 90 °C under a stream of nitrogen until all of the solvent was evaporated. The evaporation process was then repeated twice with the addition of anhydrous MeCN (1.0 mL) each time. After evaporation, anhydrous MeCN (500 µL) was added to the V-vial. The V-vial was sealed with a Teflon-lined cap and stirred at a preheated metal heating block (110 °C) for 20 min. The vial was removed from the hot plate before the addition of water and MeCN (500 µL, 1:1, v/v). An aliquot was removed and used for radio-TLC and radio-HPLC analysis. The product identity and purity was determined by comparison of the HPLC radio-trace with the HPLC UV-trace of the authentic reference sample fluorodiflunisal derivative **S29**.

Figure S07. Radio-HPLC trace of the ^{18}F-fluorination reaction yielding **29**.

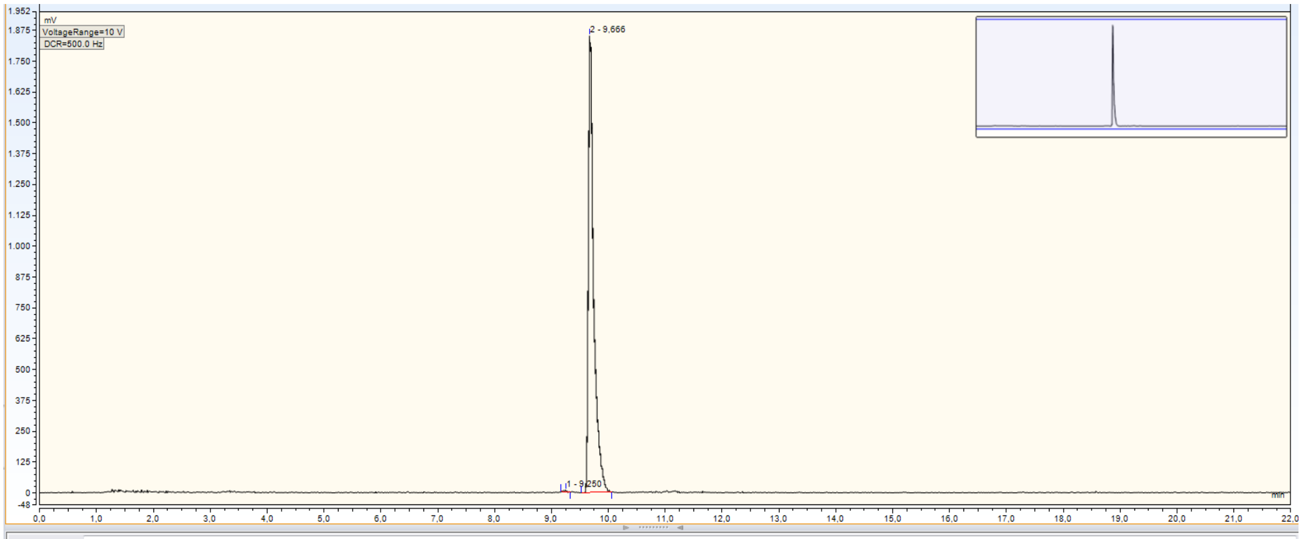

No.	Peakname	Ret.Time min	Area mV*min	Amount n.a.	Type	Height mV	Rel.Area %	Resolution
1		9,250	0,3363	n.a.	BMB*	7,437	0,17	4,88
2		9,666	202,3658	n.a.	BMB*	1850,969	99,83	n.a.
Total:			202,7021	0,0000		1858,406	100,00	

Figure S08. HPLC UV-trace of **S29** as the reference.

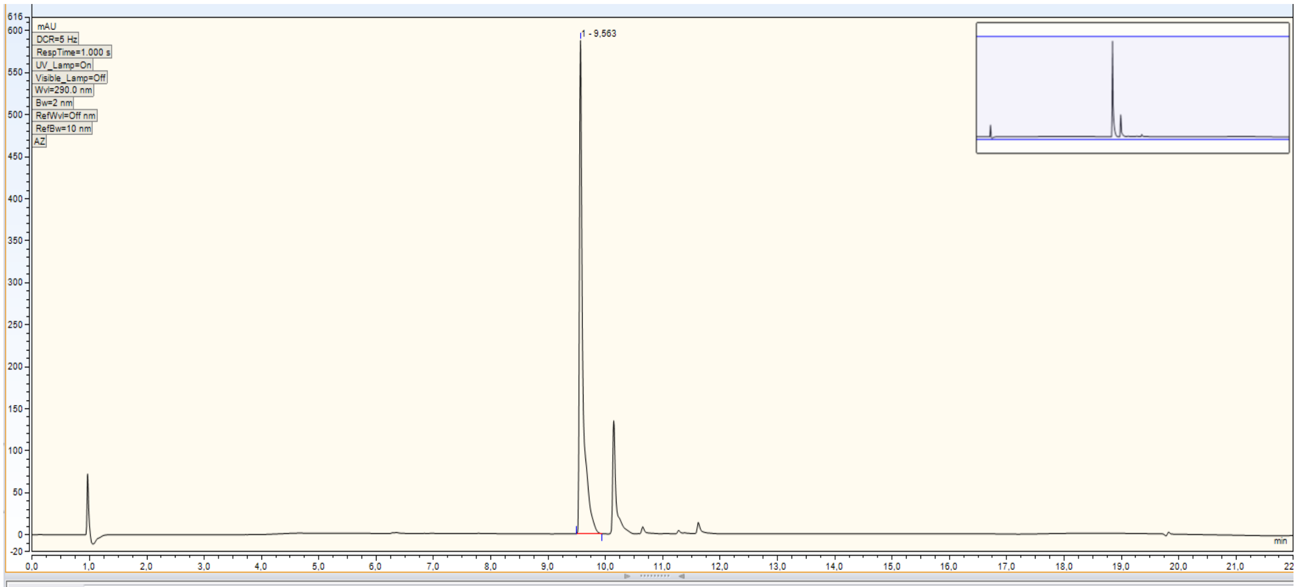

No.	Peakname	Ret.Time min	Area mAU*min	Amount n.a.	Type	Height mAU	Rel.Area %	Resolution
1		9,563	40,9779	n.a.	BMB*	587,353	100,00	n.a.
Total:			40,9779	0,0000		587,353	100,00	

Table S03. Radiochemical yield of **29**.

Reaction	EE	RCC (Radio-TLC)	Radio-HPLC	RCY
1	92%	92%	94%	80%
2	95%	93%	100%	88%

3	91%	92%	93%	78%
average RCY				82 ± 6%

[^{18}F]Fluoroclofibrate (30)

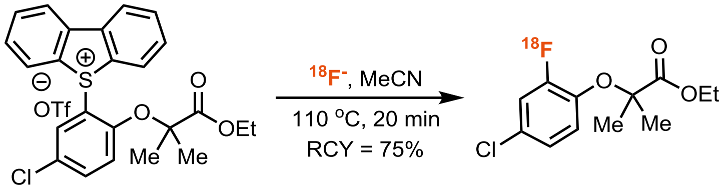

Aqueous [^{18}F]fluoride solution (20–30 MBq) was loaded with a syringe onto a QMA anion-exchange cartridge that was pre-conditioned according to the general procedure, and then the cartridge was washed with anhydrous MeCN (1.0 mL) and air-dried (2.0 mL). The [^{18}F]fluoride was eluted from the cartridge with a methanol solution (500 µL) of clofibrate-derived dibenzothiophenium salt **S18** (5.3 mg, 9.0 µmol) into a 5 mL borosilicate vial (with a stir bar), followed by anhydrous MeCN (500 µL) and air-dried (1.0 mL). The V-vial was heated to 90 °C under a stream of nitrogen until all of the solvent was evaporated. The evaporation process was then repeated twice with the addition of anhydrous MeCN (1.0 mL) each time. After evaporation, anhydrous MeCN (500 µL) was added to the V-vial. The V-vial was sealed with a Teflon-lined cap and stirred at a preheated metal heating block (110 °C) for 20 min. The vial was removed from the hot plate before the addition of water and MeCN (500 µL, 1:1, v/v). An aliquot was removed and used for radio-TLC and radio-HPLC analysis. The product identity and purity was determined by comparison of the HPLC radio-trace with the HPLC UV-trace of the authentic reference sample fluoroclofibrate (**S30**).

Figure S09. Radio-HPLC trace of the ^{18}F-fluorination reaction yielding **30**.

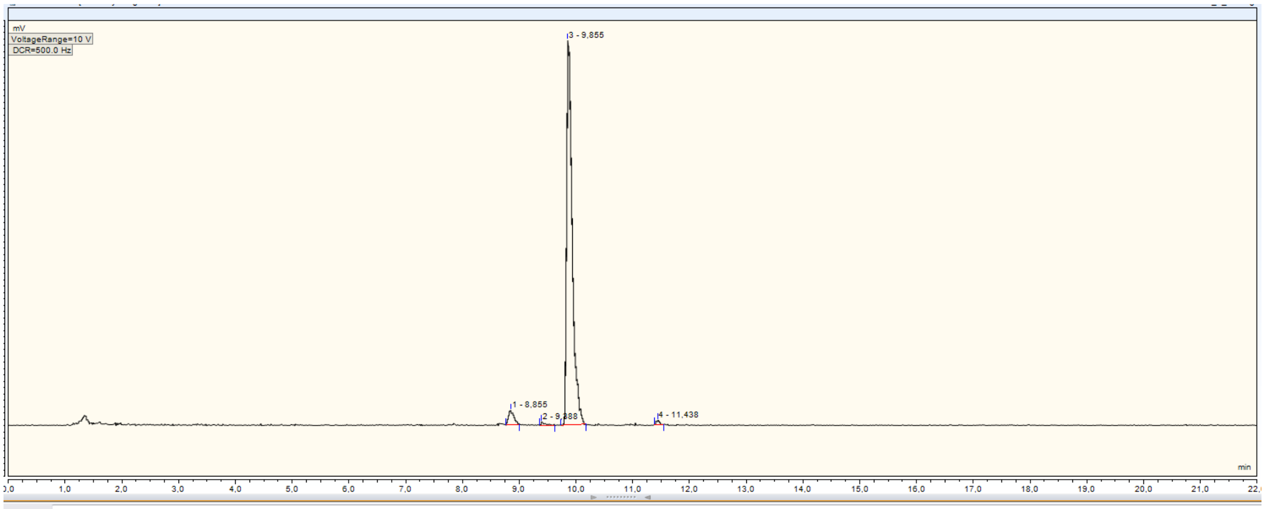

No.	Peakname	Ret.Time min	Area mV*min	Amount n.a.	Type	Height mV	Rel.Area %	Resolution
1		8,855	6,3772	n.a.	BMB*	56,769	3,54	4,72
2		9,388	1,2423	n.a.	BMB*	13,937	0,69	4,12
3		9,855	171,6661	n.a.	BMB*	1517,029	95,26	11,17
4		11,438	0,9166	n.a.	BMB*	16,290	0,51	n.a.
Total:			180,2022	0,0000		1604,025	100,00	

Figure S10. HPLC UV-trace of **S30** as the reference.

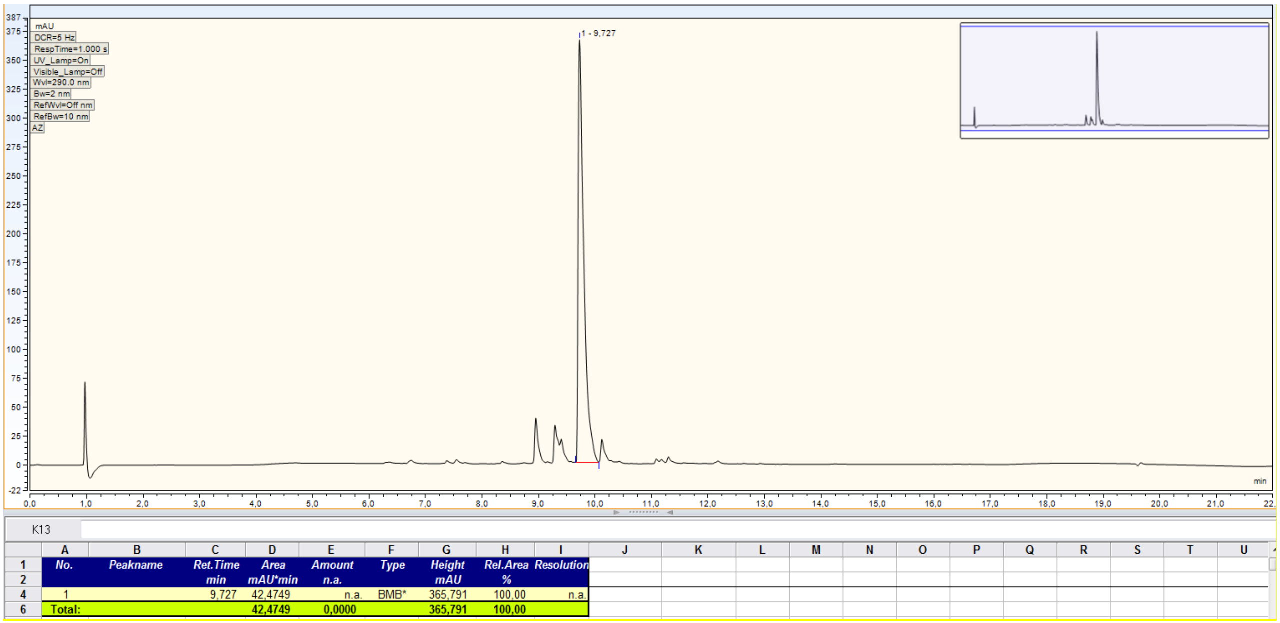

No.	Peakname	Ret.Time min	Area mAU*min	Amount n.a.	Type	Height mAU	Rel.Area %	Resolution
1		9,727	42,4749	n.a.	BMB*	365,791	100,00	n.a.
Total:			42,4749	0,0000		365,791	100,00	

Table S04. Radiochemical yield of **30**.

Reaction	EE	RCC (Radio-TLC)	Radio-HPLC	RCY
1	75%	95%	95%	68%
2	92%	91%	95%	80%
3	92%	87%	96%	77%
average RCY				75 ± 7%

3-Chloro-9-[^{18}F]fluoro-6-methyldibenzo[*c*,*f*][1,2]thiazepin-11(6*H*)-one 5,5-dioxide (31)

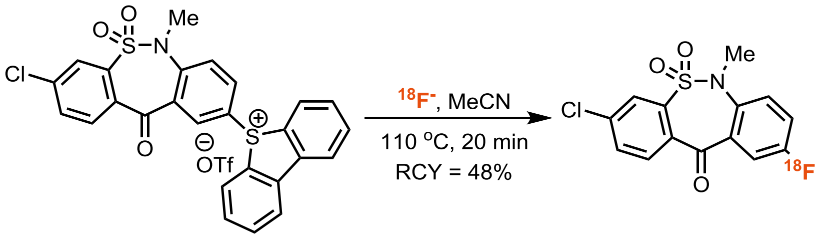

S9 **31**

Aqueous [^{18}F]fluoride solution (20–30 MBq) was loaded with a syringe onto a QMA anion-exchange cartridge that was pre-conditioned according to the general procedure, and then the cartridge was washed with anhydrous MeCN (1.0 mL) and air-dried (2.0 mL). The [^{18}F]fluoride was eluted from the cartridge with a methanol solution (500 µL) of 3-chloro-6-methyldibenzo[*c*,*f*][1,2]thiazepin-11(6*H*)-one 5,5-dioxide-derived dibenzothiophenium salt **S9** (5.5 mg, 9.0 µmol) into a 5 mL borosilicate vial (with a stir bar), followed by anhydrous MeCN (500 µL) and air-dried (1.0 mL). The V-vial was heated to 90 °C under a stream of nitrogen

until all of the solvent was evaporated. The evaporation process was then repeated twice with the addition of anhydrous MeCN (1.0 mL) each time. After evaporation, anhydrous MeCN (500 µL) was added to the V-vial. The V-vial was sealed with a Teflon-lined cap and stirred at a preheated metal heating block (110 °C) for 20 min. The vial was removed from the hot plate before the addition of water and MeCN (500 µL, 1:1, v/v). An aliquot was removed and used for radio-TLC and radio-HPLC analysis. The product identity and purity was determined by comparison of the HPLC radio-trace with the HPLC UV-trace of the authentic reference sample chloro-9-fluoro-6-methyldibenzo[*c*,*f*][1,2]thiazepin-11(6*H*)-one 5,5-dioxide **S31**.

Figure S11. Radio-HPLC trace of the ^{18}F-fluorination reaction yielding **31**.

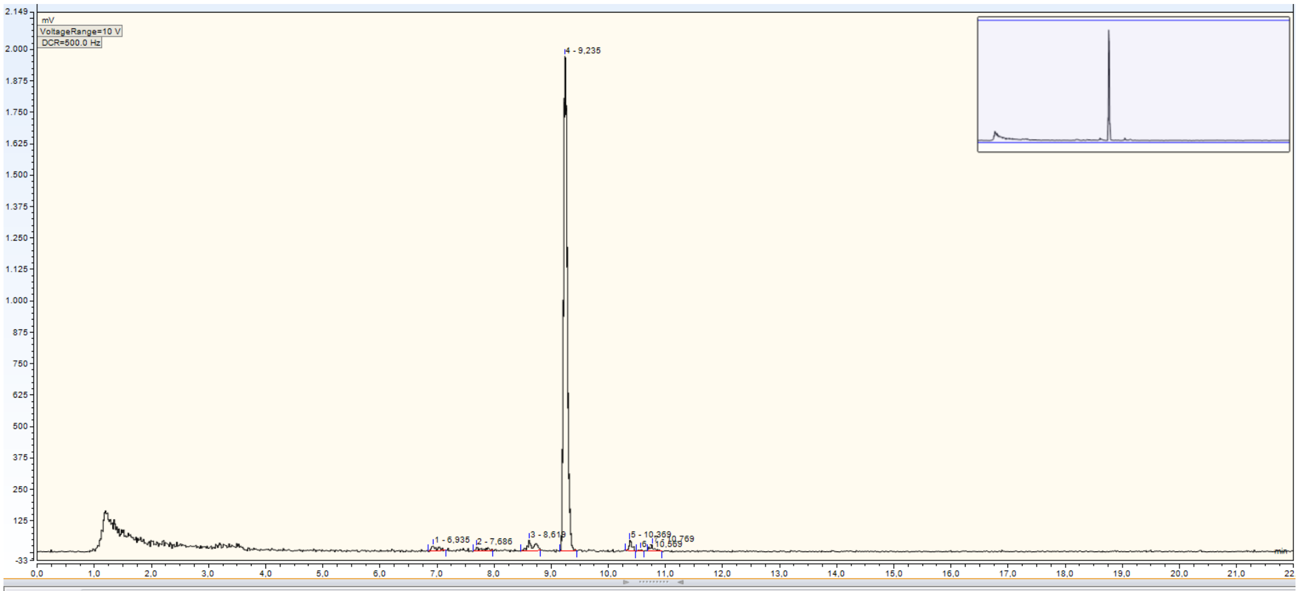

No.	Peakname	Ret.Time min	Area mV*min	Amount n.a.	Type	Height mV	Rel.Area %	Resolution
1		6,935	2,4163	n.a.	BMB*	20,825	1,43	10,59
2		7,686	2,2180	n.a.	BMB*	16,723	1,31	13,22
3		8,619	5,5084	n.a.	BMB*	43,130	3,26	6,24
4		9,235	153,5170	n.a.	BMB*	1968,471	90,97	13,33
5		10,369	2,5506	n.a.	BMB*	41,879	1,51	2,82
6		10,569	0,3824	n.a.	BMB*	6,087	0,23	3,54
7		10,769	2,1634	n.a.	BMB*	24,712	1,28	n.a.
Total:			168,7562	0,0000		2121,826	100,00	

Figure S12. HPLC UV-trace of **S31** as the reference.

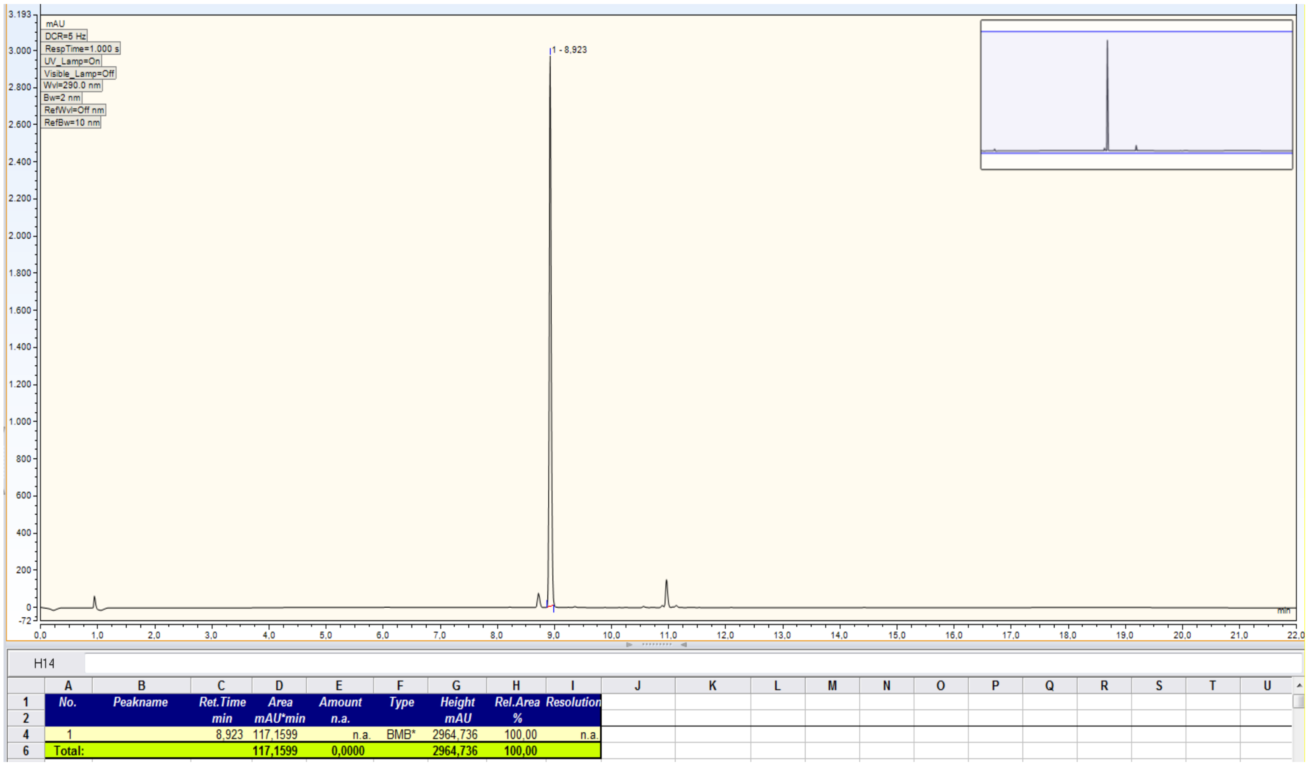

No.	Peakname	Ret.Time min	Area mAU*min	Amount n.a.	Type	Height mAU	Rel.Area %	Resolution
1		8,923	117,1599	n.a.	BMB*	2964,736	100,00	n.a.
Total:			117,1599	0,0000		2964,736	100,00	

Table S05. Radiochemical yield of **31**.

Reaction	EE	RCC (Radio-TLC)	Radio-HPLC	RCY
1	85%	56%	97%	46%
2	91%	60%	91%	50%
3	88%	51%	92%	41%
average RCY				46 ± 5%

[^{18}F]Fluorodicamba methylester (32)

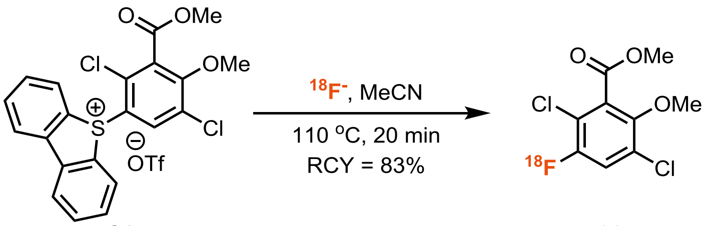

S17 **32**

Aqueous [^{18}F]fluoride solution (20–30 MBq) was loaded with a syringe onto a QMA anion-exchange cartridge that was pre-conditioned according to the general procedure, and then the cartridge was washed with anhydrous MeCN (1.0 mL) and air-dried (2.0 mL). The [^{18}F]fluoride was eluted from the cartridge with a methanol solution (500 µL) of dicamba methylester-derived dibenzothiophenium salt **S17** (5.1 mg, 9.0 µmol) into a 5 mL borosilicate vial (with a stir bar), followed by anhydrous MeCN (500 µL) and air-dried (1.0 mL).

The V-vial was heated to 90 °C under a stream of nitrogen until all of the solvent was evaporated. The evaporation process was then repeated twice with the addition of anhydrous MeCN (1.0 mL) each time. After evaporation, anhydrous MeCN (500 µL) was added to the V-vial. The V-vial was sealed with a Teflon-lined cap and stirred at a preheated metal heating block (110 °C) for 20 min. The vial was removed from the hot plate before the addition of water and MeCN (500 µL, 1:1, v/v). An aliquot was removed and used for radio-TLC and radio-HPLC analysis. The product identity and purity was determined by comparison of the HPLC radio-trace with the HPLC UV-trace of the authentic reference sample fluorodicamba methylester (**S32**).

Figure S13. Radio-HPLC trace of the ^{18}F-fluorination reaction yielding **32**.

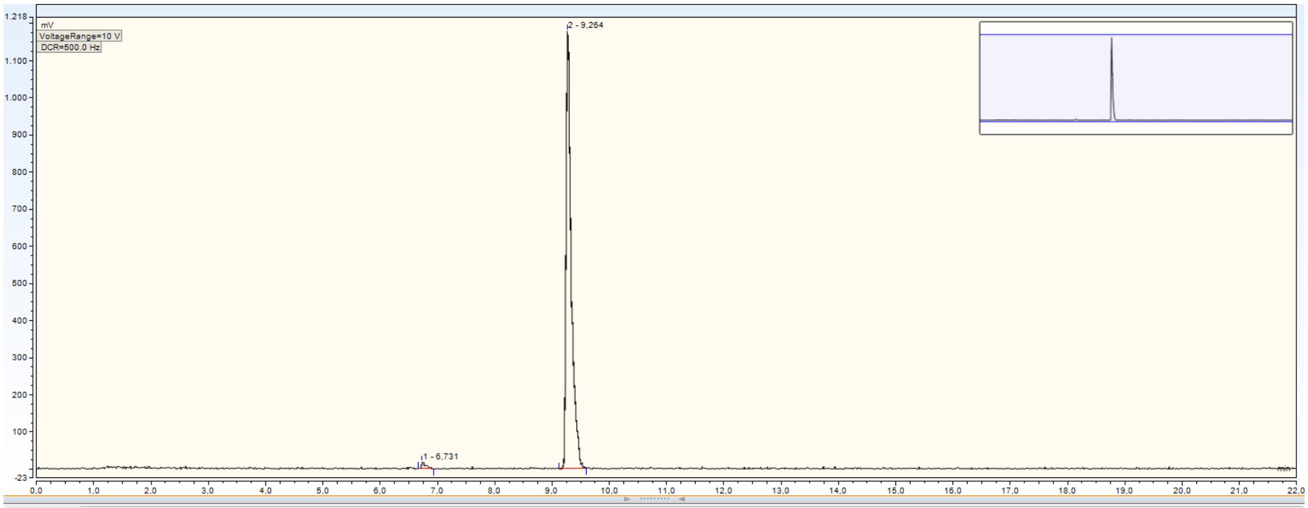

N19

	A	B	C	D	E	F	G	H	I
1	No.	Peakname	Ret.Time	Area	Amount	Type	Height	Rel.Area	Resolution
2			min	mV*min	n.a.		mV	%	
4	1		6,731	1,4298	n.a.	BMB*	16,833	1,13	22,48
5	2		9,264	124,8809	n.a.	BMB*	1179,128	98,87	n.a.
6	Total:			126,3107	0,0000		1195,961	100,00	

Figure S14. HPLC UV-trace of **S32** as the reference.

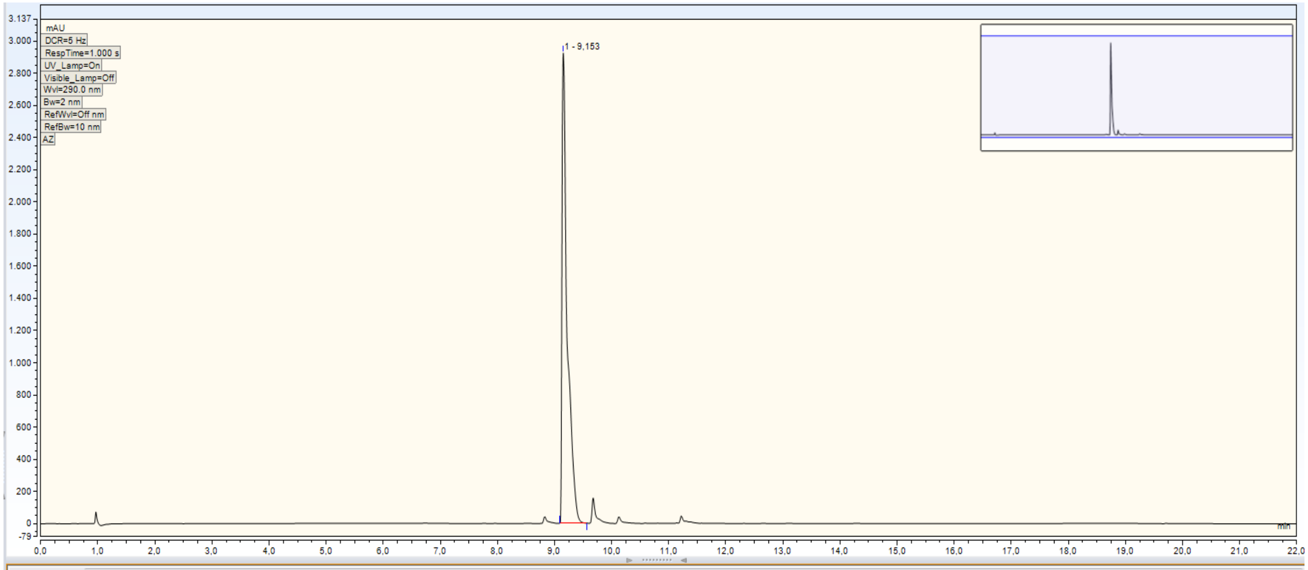

N19

	A	B	C	D	E	F	G	H	I
1	No.	Peakname	Ret.Time	Area	Amount	Type	Height	Rel.Area	Resolution
2			min	mAU*min	n.a.		mAU	%	
4	1		9,153	311,0340	n.a.	BMB*	2923,619	100,00	n.a.
6	Total:			311,0340	0,0000		2923,619	100,00	

Table S06. Radiochemical yield of **32**.

Reaction	EE	RCC (Radio-TLC)	Radio-HPLC	RCY
1	88%	90%	98%	78%
2	93%	95%	99%	87%
3	92%	91%	93%	78%
average RCY				81 ± 6%

[^{18}F]Fluorobifonazole (33)

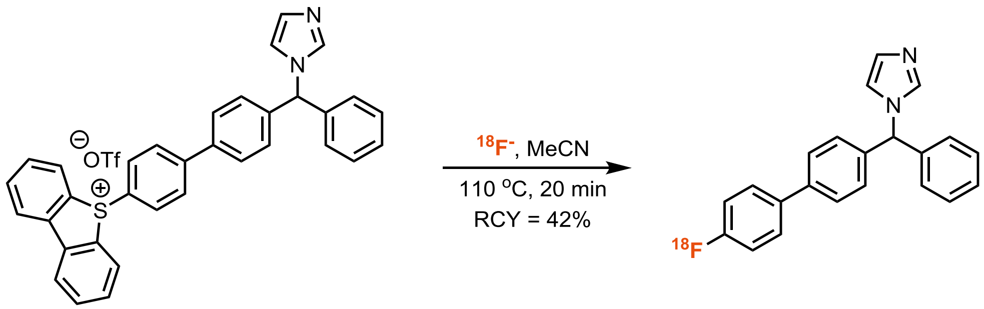

S19 **33**

Aqueous [^{18}F]fluoride solution (20–30 MBq) was loaded with a syringe onto a QMA anion-exchange cartridge that was pre-conditioned according to the general procedure, and then the cartridge was washed with anhydrous MeCN (1.0 mL) and air-dried (2.0 mL). The [^{18}F]fluoride was eluted from the cartridge with a methanol solution (500 µL) of bifonazole-derived dibenzothiophenium salt **S19** (5.8 mg, 9.0 µmol) into a 5 mL borosilicate vial (with a stir bar), followed by anhydrous MeCN (500 µL) and air-dried (1.0 mL). The V-vial was heated to 90 °C under a stream of nitrogen until all of the solvent was evaporated. The evaporation process was then repeated twice with the addition of anhydrous MeCN (1.0 mL) each time. After evaporation, anhydrous MeCN (500 µL) was added to the V-vial. The V-vial was sealed with a Teflon-lined cap and stirred at a preheated metal heating block (110 °C) for 20 min. The vial was removed from the hot plate before the addition of water and MeCN (500 µL, 1:1, v/v). An aliquot was removed and used for radio-TLC and radio-HPLC analysis. The product identity and purity was determined by comparison of the HPLC radio-trace with the HPLC UV-trace of the authentic reference sample **S33**.

Figure S15. Radio-HPLC trace of the ^{18}F-fluorination reaction yielding **33**.

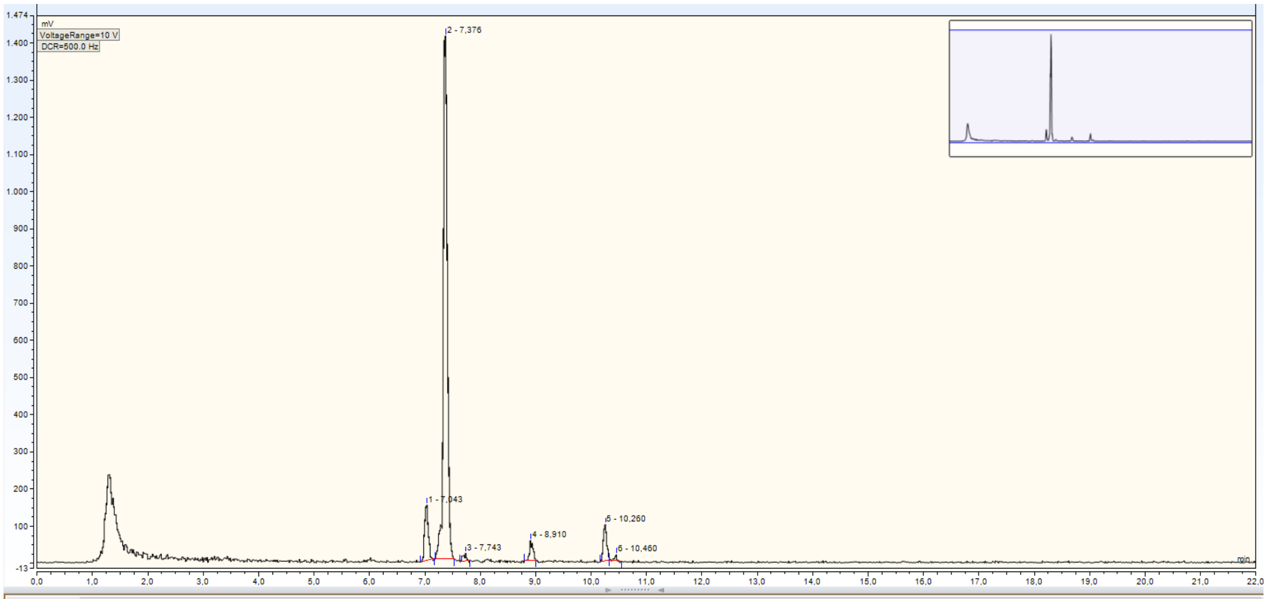

H14

No.	Peakname	Ret.Time min	Area mV*min	Amount n.a.	Type	Height mV	Rel.Area %	Resolution
1		7,043	11,5286	n.a.	BMB*	148,998	7,95	2,62
2		7,376	120,7997	n.a.	BMB*	1407,047	83,26	4,31
3		7,743	1,3495	n.a.	BMB*	20,548	0,93	16,51
4		8,910	3,8345	n.a.	BMB*	54,025	2,64	11,94
5		10,260	6,9262	n.a.	BMB*	97,750	4,77	2,36
6		10,460	0,6510	n.a.	BMB*	16,675	0,45	n.a.
Total:			145,0896	0,0000		1745,042	100,00	

Figure S16. HPLC UV-trace of **S33** as the reference.

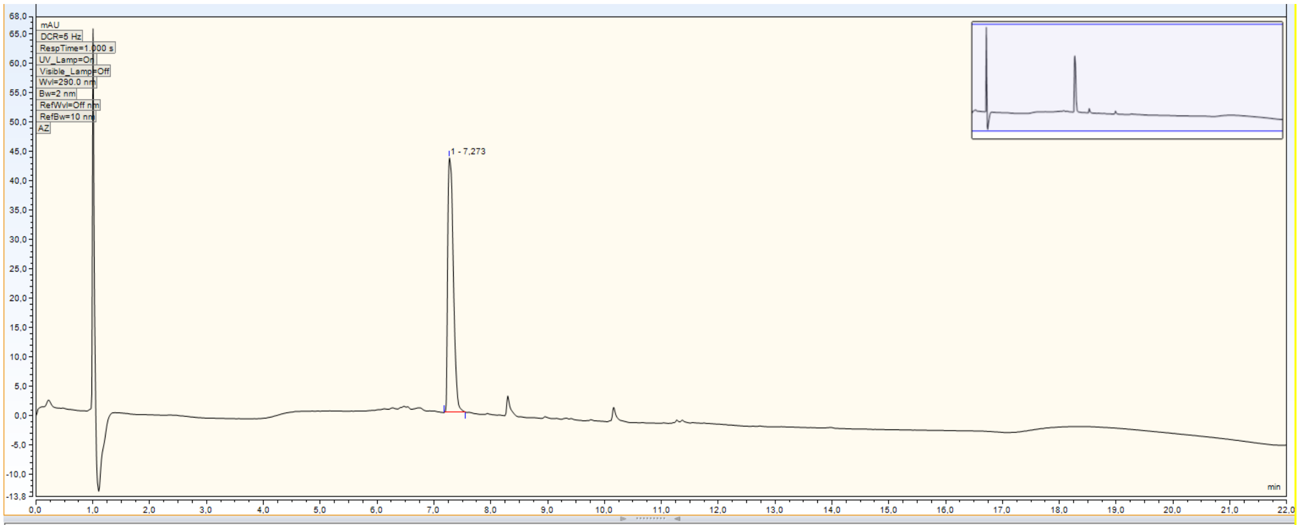

G17

No.	Peakname	Ret.Time min	Area mAU*min	Amount n.a.	Type	Height mAU	Rel.Area %	Resolution
1		7,273	4,9871	n.a.	BMB*	43,372	100,00	n.a.
Total:			4,9871	0,0000		43,372	100,00	

Table S07. Radiochemical yield of **33**.

Reaction	EE	RCC (Radio-TLC)	Radio-HPLC	RCY
1	91%	54%	83%	41%
2	93%	58%	83%	45%

3	90%	56%	81%	40%
average RCY				42 ± 3%

4-[^{18}F]Fluoroiodobenzene (34)

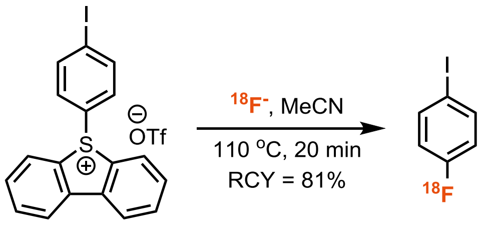

S23 **34**

Aqueous [^{18}F]fluoride solution (20–30 MBq) was loaded with a syringe onto a QMA anion-exchange cartridge that was pre-conditioned according to the general procedure, and then the cartridge was washed with anhydrous MeCN (1.0 mL) and air-dried (2.0 mL). The [^{18}F]fluoride was eluted from the cartridge with a methanol solution (500 µL) of iodobenzene-derived dibenzothiophenium salt **S23** (4.8 mg, 9.0 µmol) into a 5 mL borosilicate vial (with a stir bar), followed by anhydrous MeCN (500 µL) and air-dried (1.0 mL). The V-vial was heated to 90 °C under a stream of nitrogen until all of the solvent was evaporated. The evaporation process was then repeated twice with the addition of anhydrous MeCN (1.0 mL) each time. After evaporation, anhydrous MeCN (500 µL) was added to the V-vial. The V-vial was sealed with a Teflon-lined cap and stirred at a preheated metal heating block (110 °C) for 20 min. The vial was removed from the hot plate before the addition of water and MeCN (500 µL, 1:1, v/v). An aliquot was removed and used for radio-TLC and radio-HPLC analysis. The product identity and purity was determined by comparison of the HPLC radio-trace with the HPLC UV-trace of the authentic reference sample 4-fluoroiodobenzene **S34**.

Figure S17. Radio-HPLC trace of the ^{18}F-fluorination reaction yielding **34**.

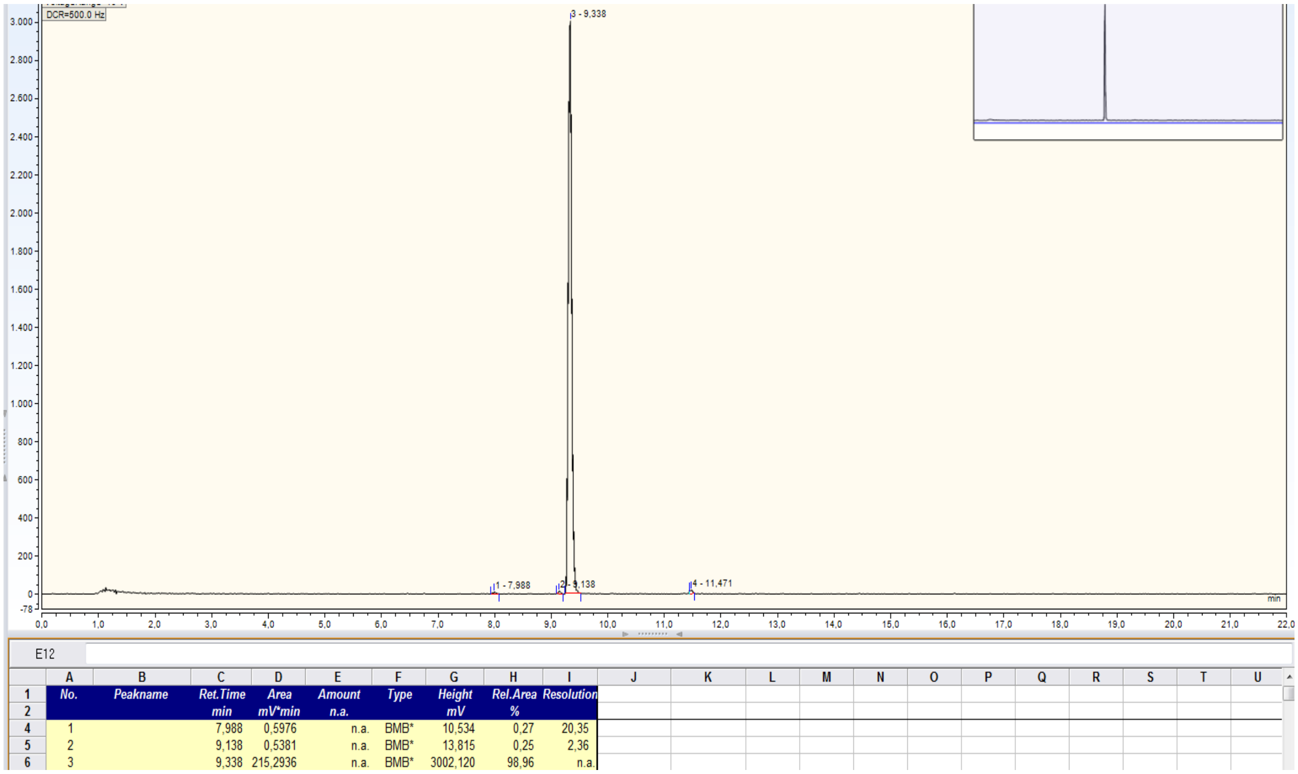

	A	B	C	D	E	F	G	H	I
1	No.	Peakname	Ret.Time	Area	Amount	Type	Height	Rel.Area	Resolution
2			min	mV*min	n.a.		mV	%	
4	1		7,988	0,5976	n.a.	BMB*	10,534	0,27	20,35
5	2		9,138	0,5381	n.a.	BMB*	13,815	0,25	2,36
6	3		9,338	215,2936	n.a.	BMB*	3002,120	98,96	n.a.

Figure S18. HPLC UV-trace of **S34** as the reference.

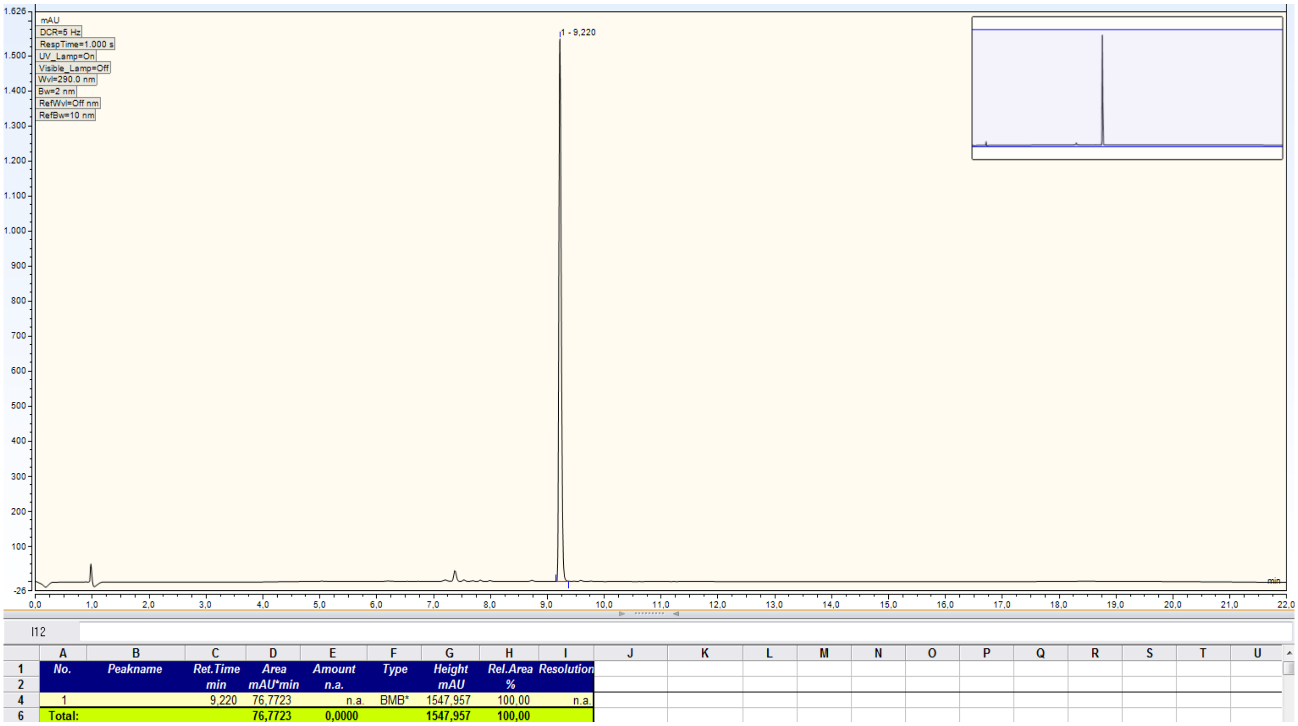

	A	B	C	D	E	F	G	H	I
1	No.	Peakname	Ret.Time	Area	Amount	Type	Height	Rel.Area	Resolution
2			min	mAU*min	n.a.		mAU	%	
4	1		9,220	76,7723	n.a.	BMB*	1547,957	100,00	n.a.
6	Total:			76,7723	0,0000		1547,957	100,00	

Table S08. Radiochemical yield of **34**.

Reaction	EE	RCC (Radio-TLC)	Radio-HPLC	RCY

1	90%	91%	99%	81%
2	87%	82%	100%	71%
3	92%	85%	98%	77%
average RCY				76 ± 5%

[^{18}F]Fluoroflurbiprofen methylester (35)

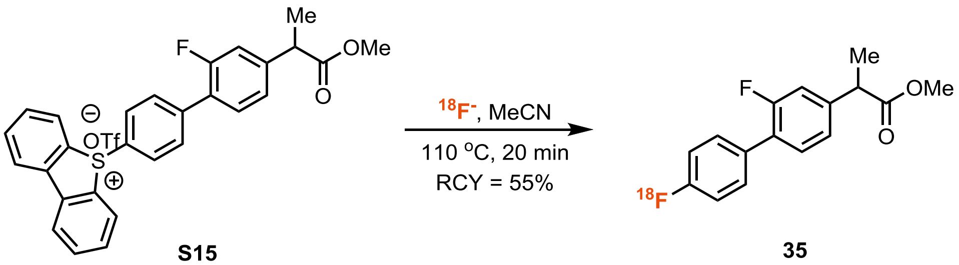

Aqueous [^{18}F]fluoride solution (20–30 MBq) was loaded with a syringe onto a QMA anion-exchange cartridge that was pre-conditioned according to the general procedure, and then the cartridge was washed with anhydrous MeCN (1.0 mL) and air-dried (2.0 mL). The [^{18}F]fluoride was eluted from the cartridge with a methanol solution (500 µL) of flurbiprofen methylester-derived dibenzothiophenium salt **S15** (5.3 mg, 9.0 µmol) into a 5 mL borosilicate vial (with a stir bar), followed by anhydrous MeCN (500 µL) and air-dried (1.0 mL). The V-vial was heated to 90 °C under a stream of nitrogen until all of the solvent was evaporated. The evaporation process was then repeated twice with the addition of anhydrous MeCN (1.0 mL) each time. After evaporation, anhydrous MeCN (500 µL) was added to the V-vial. The V-vial was sealed with a Teflon-lined cap and stirred at a preheated metal heating block (110 °C) for 20 min. The vial was removed from the hot plate before the addition of water and MeCN (500 µL, 1:1, v/v). An aliquot was removed and used for radio-TLC and radio-HPLC analysis. The product identity and purity was determined by comparison of the HPLC radio-trace with the HPLC UV-trace of the authentic reference sample **S35**.

Figure S19. Radio-HPLC trace of the ^{18}F-fluorination reaction yielding **35**.

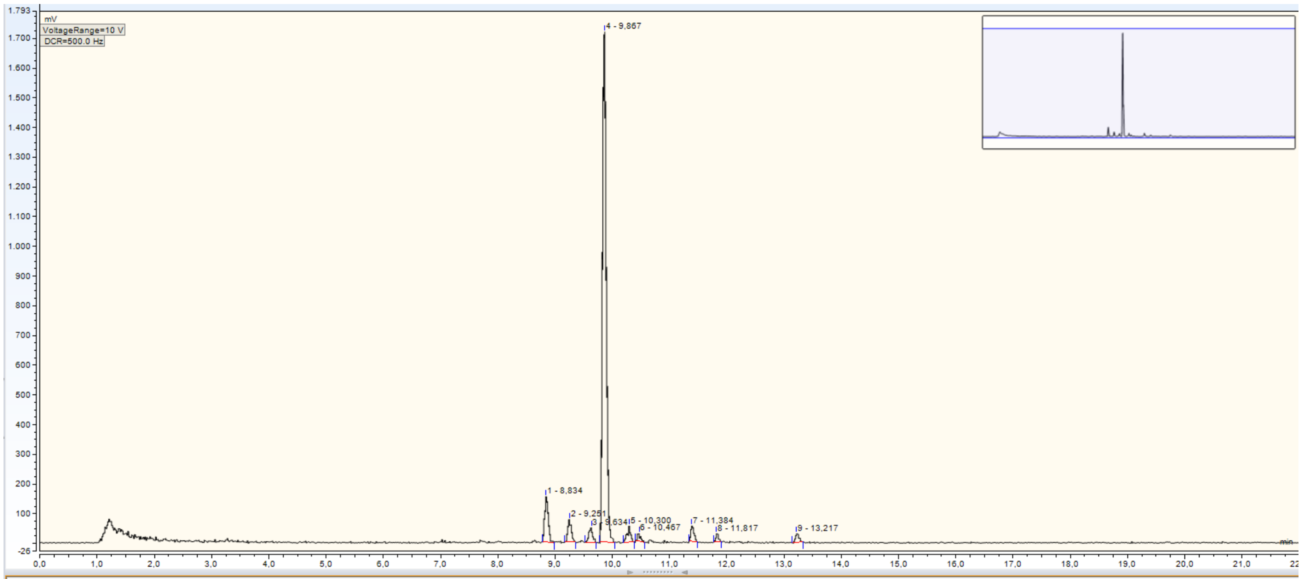

H17

No.	Peakname	Ret.Time min	Area mV*min	Amount n.a.	Type	Height mV	Rel.Area %	Resolution
1		8,834	11,2586	n.a.	BMB*	152,777	6,96	3,69
2		9,251	5,3542	n.a.	BMB*	75,485	3,31	3,40
3		9,634	3,7478	n.a.	BMB*	50,136	2,32	1,84
4		9,867	129,2090	n.a.	BMB*	1717,121	79,92	4,39
5		10,300	3,6401	n.a.	BMB*	54,243	2,25	2,95
6		10,467	1,5596	n.a.	BMB*	25,898	0,96	10,81
7		11,384	3,3941	n.a.	BMB*	51,653	2,10	5,13
8		11,817	1,4102	n.a.	BMB*	27,449	0,87	19,74
9		13,217	2,0920	n.a.	BMB*	29,130	1,29	n.a.
Total:			161,6656	0,0000		2183,893	100,00	

Figure S20. HPLC UV-trace of **S35** as the reference.

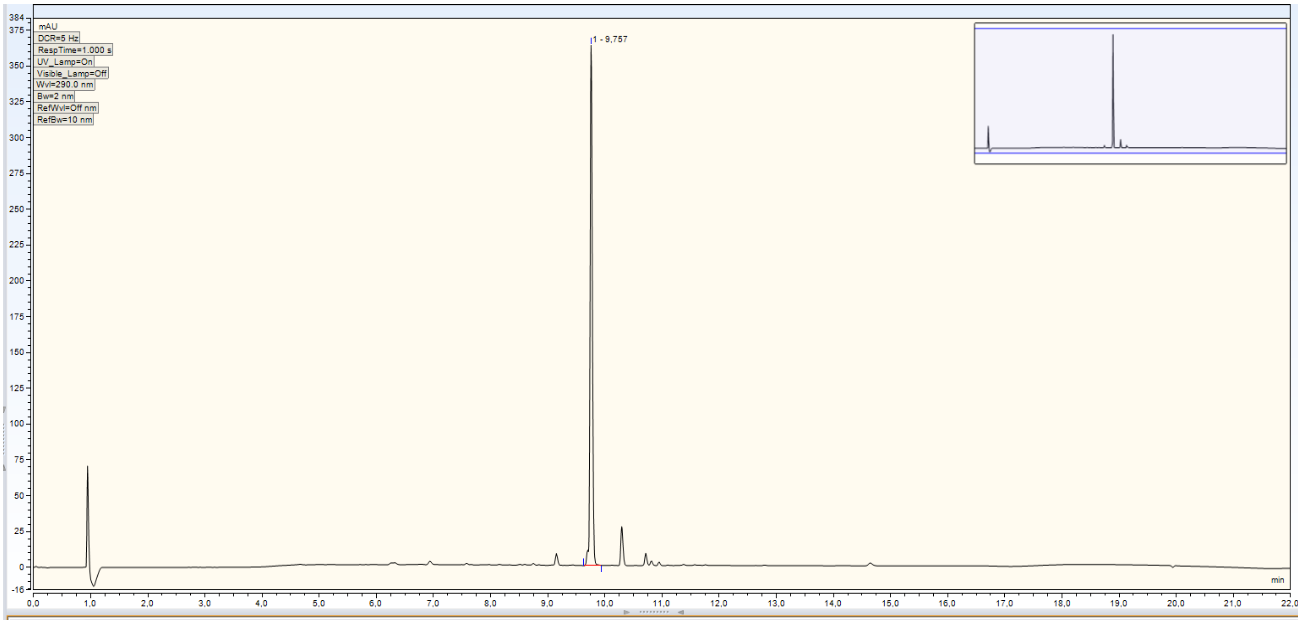

I15

No.	Peakname	Ret.Time min	Area mAU*min	Amount n.a.	Type	Height mAU	Rel.Area %	Resolution
1		9,757	16,9460	n.a.	BMB*	363,275	100,00	n.a.
Total:			16,9460	0,0000		363,275	100,00	

Table S09. Radiochemical yield of **35**.

Reaction	EE	RCC (Radio-TLC)	Radio-HPLC	RCY

1	90%	74%	78%	52%
2	94%	80%	80%	60%
3	92%	77%	75%	53%
average RCY				55 ± 5%

[^{18}F]Fluorosalicin pentaacetate (36)

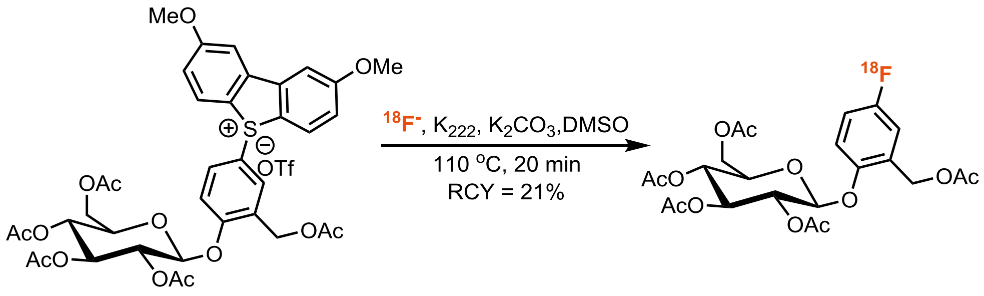

S27 **36**

Chromafix PS-HCO3 ^{18}F separation cartridge (45 mg) (Product No. 731876 from ABX) was pre-conditioned by sequentially pushing aqueous sodium hydroxide solution (5.0 mL, 1.0 M), water (10.0 mL), aqueous potassium carbonate solution (1.0 mL, 1.0 M), water (10.0 mL),and air (10 mL) through the cartridge. Then aqueous [^{18}F]fluoride solution (20–30 MBq) was loaded with a syringe to the pre-conditioned QMA anion-exchange cartridge, and then the cartridge was washed with anhydrous MeCN (1.0 mL) and air-dried (2.0 mL). The [^{18}F]fluoride was eluted from the cartridge with a solution of Kryptofix 222 (5.5 mg, 0.013 mmol) and potassium bicarbonate (1.6 mg, 0.011 mmol) dissolved in 0.5 mL acetonitrile/water (85:15, v:v). The V-vial was heated to 90 °C under a stream of nitrogen until all of the solvent was evaporated. The evaporation process was then repeated twice with the addition of anhydrous MeCN (1.0 mL) each time. Then anhydrous DMSO solution (500 µL) of salicin pentaacetate-derived 2,8-dimethoxydibenzothiophenium salt **S27** (8.0 mg, 9.0 µmol) was added to the V-vial. The V-vial was sealed with a Teflon-lined cap and stirred at a preheated metal heating block (110 °C) for 20 min. The vial was removed from the hot plate before the addition of water and MeCN (500 µL, 1:1, v/v). An aliquot was removed and used for radio-TLC and radio-HPLC analysis. The product identity and purity was determined by comparison of the HPLC radio-trace with the HPLC UV-trace of the authentic reference sample **S36**.

Figure S21. Radio-HPLC trace of the ^{18}F-fluorination reaction yielding **36**.

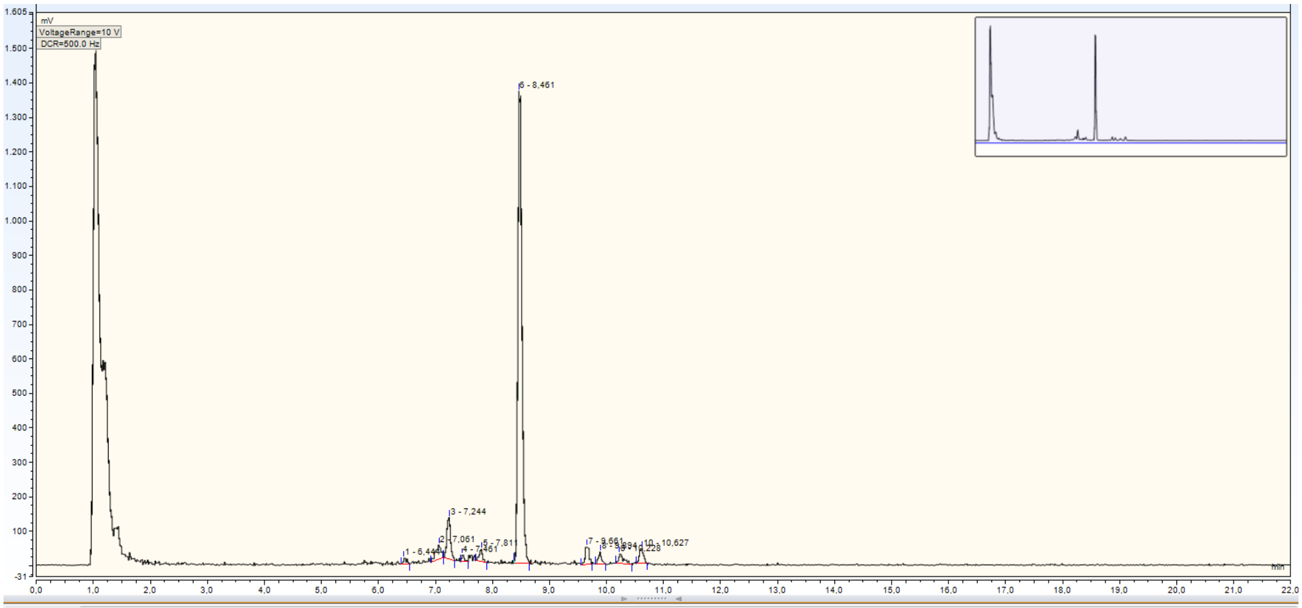

No.	Peakname	Ret.Time min	Area mV*min	Amount n.a.	Type	Height mV	Rel.Area %	Resolution
1		6,444	1,1785	n.a.	BMB*	17,219	0,81	4,84
2		7,061	4,6956	n.a.	BMB*	41,182	3,24	1,18
3		7,244	9,3255	n.a.	BMB*	121,559	6,43	2,18
4		7,461	1,0939	n.a.	BMB*	19,197	0,75	4,13
5		7,811	2,4495	n.a.	BMB*	36,085	1,69	6,57
6		8,461	113,2347	n.a.	MB*	1369,414	78,04	10,59
7		9,661	4,2085	n.a.	BMB*	51,308	2,90	2,36
8		9,894	2,1966	n.a.	BMB*	35,452	1,51	3,38
9		10,228	2,9510	n.a.	BMB*	27,539	2,03	3,15
10		10,627	3,7565	n.a.	BMB*	43,335	2,59	n.a.
Total:			145,0904	0,0000		1762,289	100,00	

Figure S22. HPLC UV-trace of **S36** as the reference.

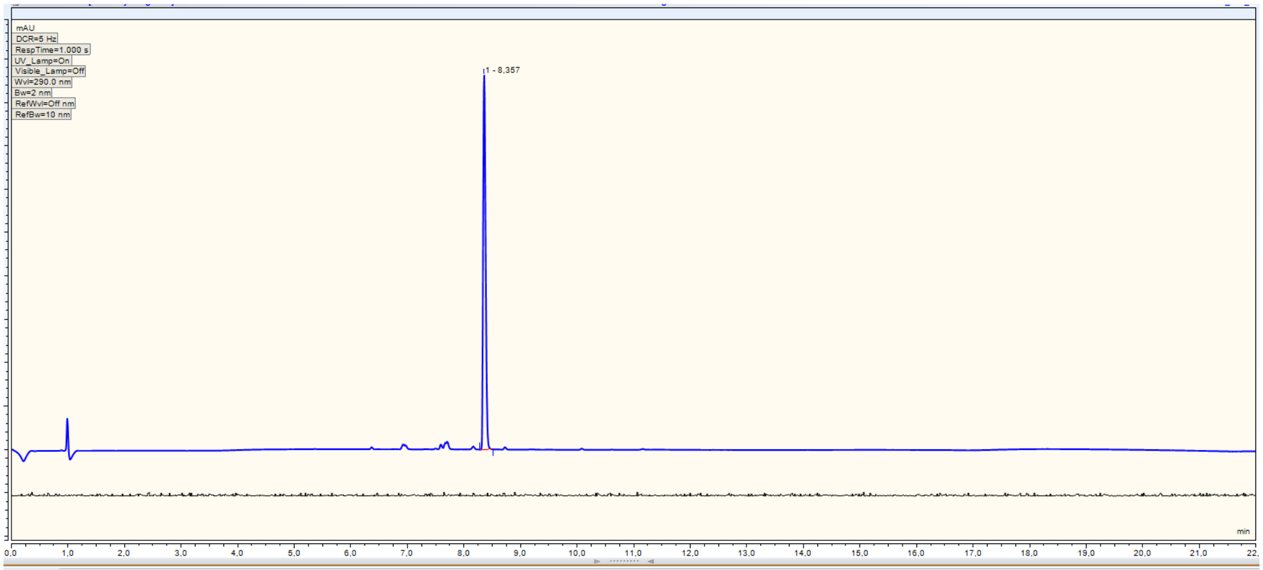

No.	Peakname	Ret.Time min	Area mAU*min	Amount n.a.	Type	Height mAU	Rel.Area %	Resolution
1		8,357	22,1136	n.a.	BMB*	431,086	100,00	n.a.
Total:			22,1136	0,0000		431,086	100,00	

Table S10. Radiochemical yield of **36**.

Reaction	EE	RCC (Radio-TLC)	Radio-HPLC	RCY

1	88%	34%	84%	25%
2	83%	26%	78%	17%
3	81%	40%	68%	22%
average RCY				21 ± 4%

[^{18}F]Fluorofenofibrate (37)

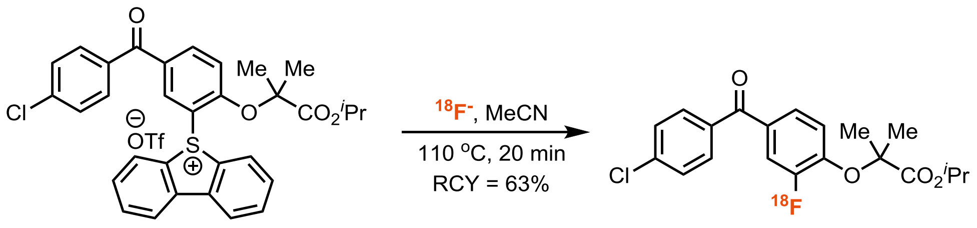

S10 **37**

Aqueous [^{18}F]fluoride solution (20–30 MBq) was loaded with a syringe onto a QMA anion-exchange cartridge that was pre-conditioned according to the general procedure, and then the cartridge was washed with anhydrous MeCN (1.0 mL) and air-dried (2.0 mL). The [^{18}F]fluoride was eluted from the cartridge with a methanol solution (500 µL) of fenofibrate-derived dibenzothiophenium salt **S10** (6.2 mg, 9.0 µmol) into a 5 mL borosilicate vial (with a stir bar), followed by anhydrous MeCN (500 µL) and air-dried (1.0 mL). The V-vial was heated to 90 °C under a stream of nitrogen until all of the solvent was evaporated. The evaporation process was then repeated twice with the addition of anhydrous MeCN (1.0 mL) each time. After evaporation, anhydrous MeCN (500 µL) was added to the V-vial. The V-vial was sealed with a Teflon-lined cap and stirred at a preheated metal heating block (110 °C) for 20 min. The vial was removed from the hot plate before the addition of water and MeCN (500 µL, 1:1, v/v). An aliquot was removed and used for radio-TLC and radio-HPLC analysis. The product identity and purity was determined by comparison of the HPLC radio-trace with the HPLC UV-trace of the authentic reference sample fluorofenofibrate (**S37**).

Figure S23. Radio-HPLC trace of the ^{18}F-fluorination reaction yielding **37**.

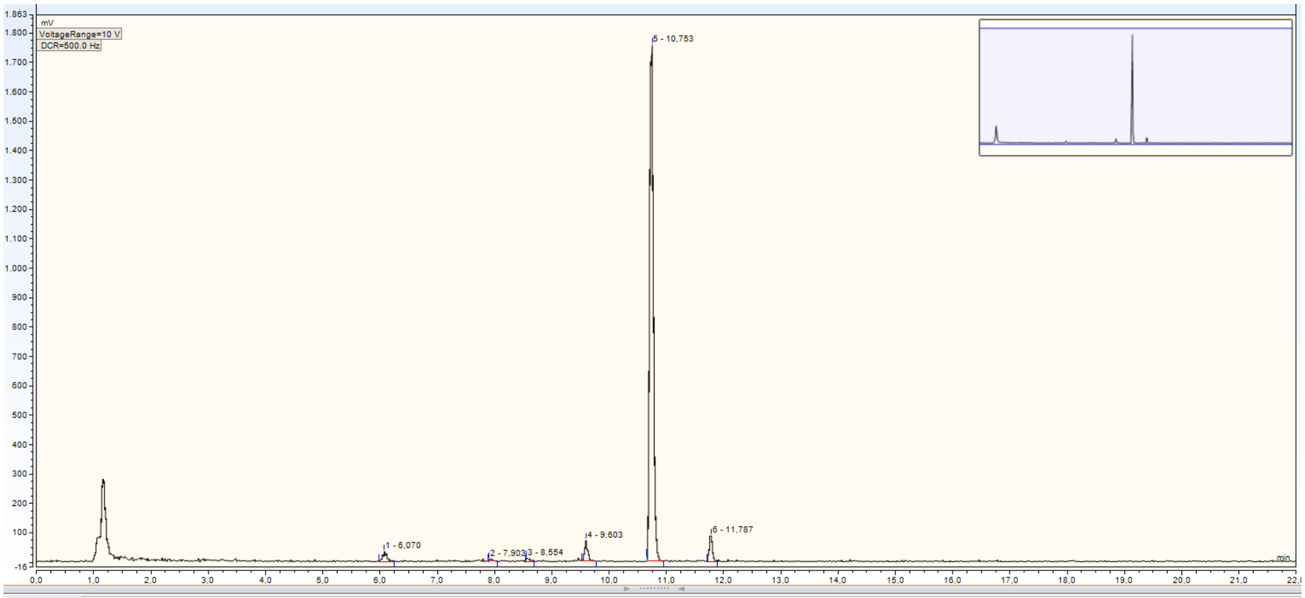

No.	Peakname	Ret.Time min	Area mV*min	Amount n.a.	Type	Height mV	Rel.Area %	Resolution
1		6,070	2,9827	n.a.	BMB*	34,585	1,97	16,18
2		7,903	0,3972	n.a.	BMB*	8,047	0,26	7,67
3		8,554	0,8302	n.a.	BMB*	11,884	0,55	12,39
4		9,603	4,2240	n.a.	BMB*	69,417	2,80	11,60
5		10,753	136,8574	n.a.	MB*	1757,421	90,57	10,43
6		11,787	5,8141	n.a.	BMB*	88,225	3,85	n.a.
Total:			151,1054	0,0000		1969,579	100,00	

Figure S24. HPLC UV-trace of **S37** as the reference.

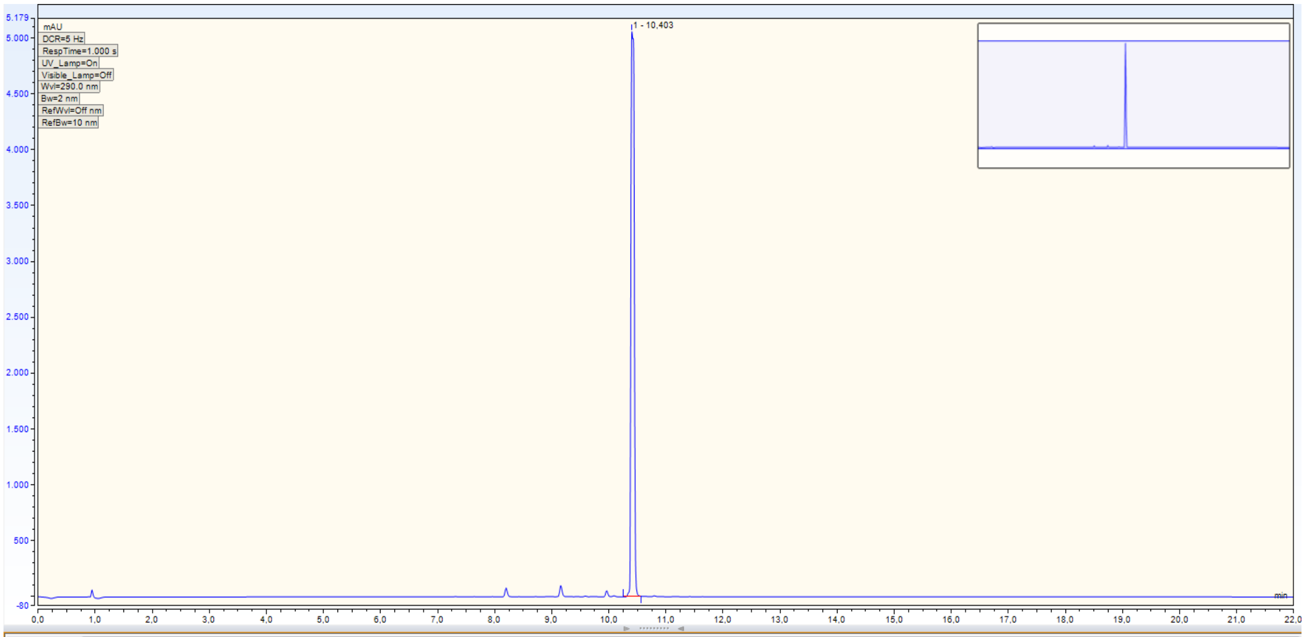

No.	Peakname	Ret.Time min	Area mAU*min	Amount n.a.	Type	Height mAU	Rel.Area %	Resolution
1		10,403	342,8623	n.a.	BMB*	5056,317	100,00	n.a.
Total:			342,8623	0,0000		5056,317	100,00	

Table S11. Radiochemical yield of **37**.

Reaction	EE	RCC (Radio-TLC)	Radio-HPLC	RCY

1	89%	75%	91%	61%
2	91%	77%	93%	65%
3	85%	86%	93%	68%
average RCY				65 ± 4%

[^{18}F]Fluoropyriproxyfen (38)

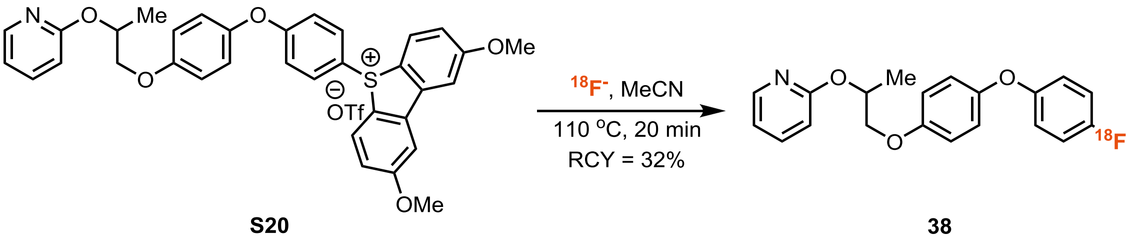

S20 **38**

Aqueous [^{18}F]fluoride solution (20–30 MBq) was loaded with a syringe onto a QMA anion-exchange cartridge that was pre-conditioned according to the general procedure, and then the cartridge was washed with anhydrous MeCN (1.0 mL) and air-dried (2.0 mL). The [^{18}F]fluoride was eluted from the cartridge with a methanol solution (500 µL) of pyriproxyfen-derived 2,8-dimethoxydibenzothiophenium salt **S20** (6.4 mg, 9.0 µmol) into a 5 mL borosilicate vial (with a stir bar), followed by anhydrous MeCN (500 µL) and air-dried (1.0 mL). The V-vial was heated to 90 °C under a stream of nitrogen until all of the solvent was evaporated. The evaporation process was then repeated twice with the addition of anhydrous MeCN (1.0 mL) each time. After evaporation, anhydrous MeCN (500 µL) was added to the V-vial. The V-vial was sealed with a Teflon-lined cap and stirred at a preheated metal heating block (110 °C) for 20 min. The vial was removed from the hot plate before the addition of water and MeCN (500 µL, 1:1, v/v). An aliquot was removed and used for radio-TLC and radio-HPLC analysis. The product identity and purity was determined by comparison of the HPLC radio-trace with the HPLC UV-trace of the authentic reference sample fluoropyriproxyfen (**S38**).

Figure S25. Radio-HPLC trace of the ^{18}F-fluorination reaction yielding **38**.

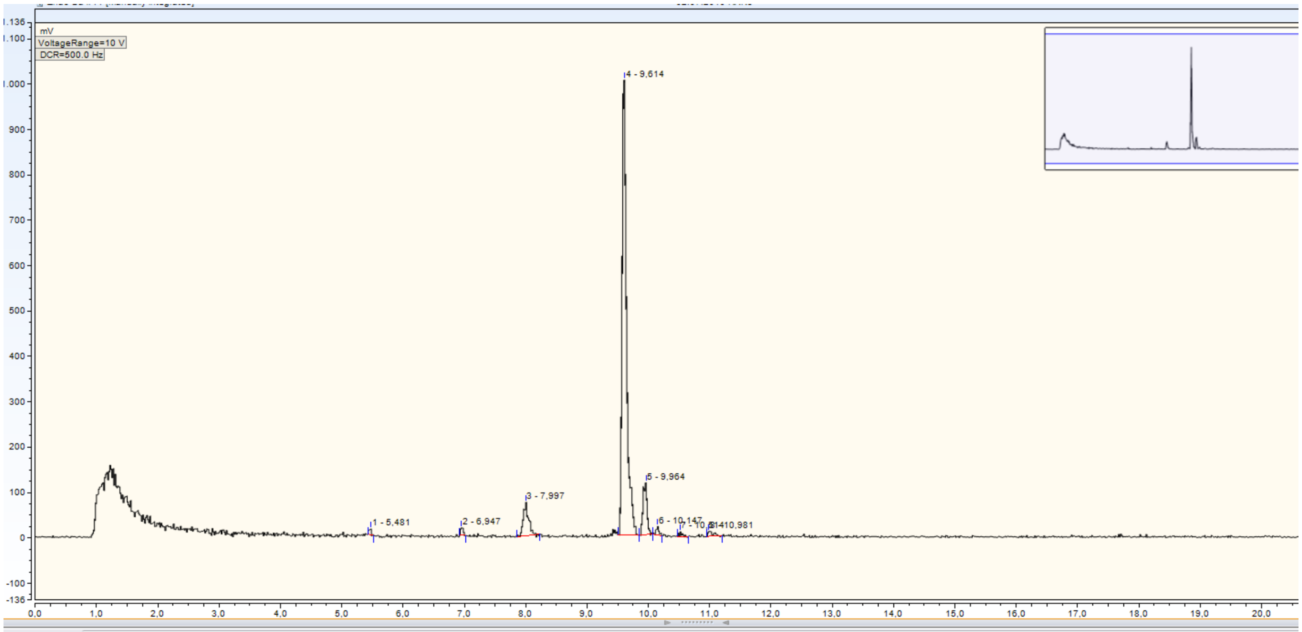

No.	Peakname	Ret.Time min	Area mV*min	Amount n.a.	Type	Height mV	Rel.Area %	Resolution
1		5,481	0,5444	n.a.	BMB*	13,594	0,52	17,26
2		6,947	0,8553	n.a.	BMB*	16,429	0,82	9,31
3		7,997	7,0912	n.a.	BMB*	74,151	6,77	12,74
4		9,614	84,8807	n.a.	BMB*	1002,607	81,09	3,10
5		9,964	8,6112	n.a.	BMB*	114,614	8,23	1,85
6		10,147	1,1302	n.a.	BMB*	19,451	1,08	6,46
7		10,514	0,4625	n.a.	BMB*	10,731	0,44	6,63
8		10,981	1,1011	n.a.	BMB*	10,994	1,05	n.a.
Total:			104,6766	0,0000		1262,572	100,00	

Figure S26. HPLC UV-trace of **S38** as the reference.

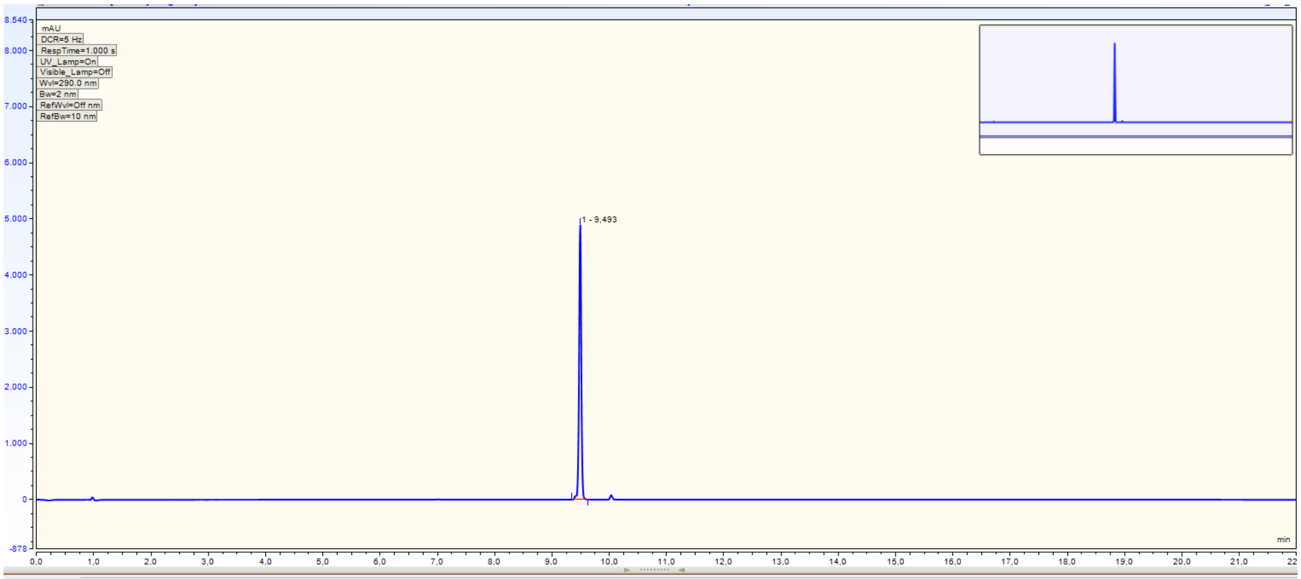

No.	Peakname	Ret.Time min	Area mAU*min	Amount n.a.	Type	Height mAU	Rel.Area %	Resolution
1		9,493	218,1585	n.a.	BMB*	4887,720	100,00	n.a.
Total:			218,1585	0,0000		4887,720	100,00	

Table S12. Radiochemical yield of **38**.

Reaction	EE	RCC (Radio-TLC)	Radio-HPLC	RCY

1	90%	42%	83%	31%
2	86%	43%	91%	31%
3	88%	46%	81%	33%
4	91%	44%	82%	33%
5	84%	38%	90%	29%
6	88%	41%	84%	30%
average RCY				31 ± 2%

Automated synthesis and determination of specific activity of [^{18}F]fluorodicamba methylester 32

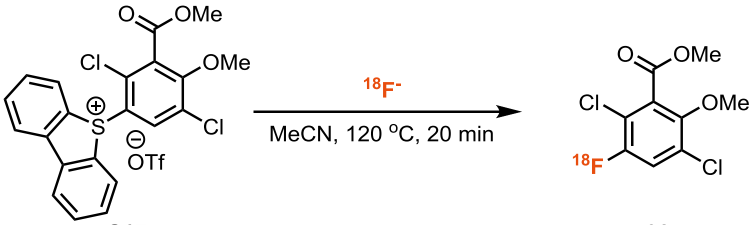

Reactions were performed on an automated cassette-based radiochemical synthesizer ELIXYS FLEX/CHEM connected to a PURE/FORM purification and formulation unit (Sofie Biosciences). Aqueous [^{18}F]fluoride (3.59 GBq, (97.0 mCi), t = 0) was trapped onto a waters QMA light anion-exchange cartridge that was pre-conditioned according to the general procedure, the cartridge was then dried under an argon flow. The [^{18}F]fluoride was eluted from the cartridge with a solution of dicamba methylester-derived dibenzothiophenium salt **S17** (5.1 mg, 9.0 µmol) in methanol (500 µL), into a 5 mL V-vial. The cartridge was eluted a second time with acetonitrile (1 mL) into the same vial. The solvent was evaporated at 135 °C under reduced pressure for 4 minutes. To the V-vial was added acetonitrile (1 mL) and the solvent was evaporated at 120 °C under reduced pressure for 3 minutes. To the V-vial was added acetonitrile (0.7 mL) and the V-vial was sealed against a Teflon-liner and was stirred at elevated temperature (set-point at 120 °C) for 20 minutes and was subsequently cooled to 50 °C. To the V-vial was added sequentially acetonitrile (0.7 mL) and water (2 mL) and then the reaction mixture was transferred from the ELIXYS FLEX/CHEM system to the PURE/FORM, and was purified by HPLC with a Hypersil Gold (250×10 mm, 5 µm, flow rate = 4 mL·min^{-1}) column with an isocratic mixture of 20:80 (MeCN:water, 0.1% TFA, v:v) for 2 minutes, followed by a linear gradient to 90:10 (MeCN:water, 0.1% TFA, v:v) within 25 minutes. The product was collected from 22 min to 23 min. The activity of the product containing fraction was diluted with water (40 mL) and loaded onto a C-18 light SepPak cartridge. The C-18 cartridge was washed with water (3 mL) and the product was sequentially eluted with ethanol (3 mL).

Specific activity of [^{18}F]fluorodicamba methylester **32** was determined by dividing the radioactivity of a sample of **32** by the amount of [^{18}F]fluorodicamba methylester **32** and fluorodicamba methylester **S32** in the sample.

The moles amount of **32** and **S32** in an isolated sample was determined by measuring the UV signal at 210 nm, and converting the UV signal intensity to number of moles according to a standard curve. For 23.4 MBq of purified compound **32** a UV absorbance (at 210 nm) of 1.73 was measured, corresponding to 0.444 nmol for a specific activity of 52.7 GBq·µmol^{-1} (1.42 Ci·µmol^{-1}) at time of injection (TOI). Integration of the UV absorbance signal (210 nm) of five known amounts of **S32** generated the standard curve (see **Table S13** and **Figure S27** for more details.)

Table S13. Data for the standard curve of UV absorbance vs. amount of **S32**.

Amount of **S32** (nmol)	UV absorbance (210 nm)
6.21	24.4
3.10	12.4
0.62	2.32
0.31	1.20
0.062	0.222

Figure S27. Standard curve of UV absorbance vs amount of **S32**.

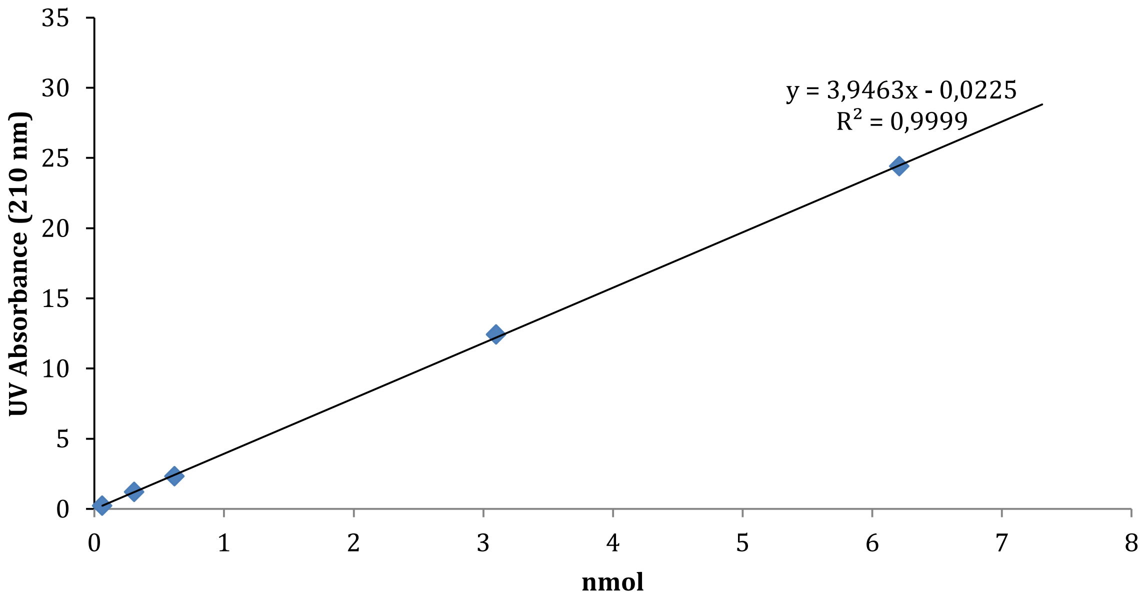

Hammett Analysis

Note: Due to the poor solubility of para-substituted phenyl 2,8-dimethoxydibenzothiophenium trifluoromethanesulfonate salt in 1,4-dioxane, TfO$^-$ counter-ion was replaced with Tf_2N^- to generate much

more soluble para-substituted phenyl 2,8-dimethoxydibenzothiophenium bis(trifluoromethanesulfonyl)amide salt. All the para-substituted phenyl 2,8-dimethoxydibenzothiophenium salts used here were synthesized by reaction with Grignard reagents. One reason is that electron-rich M-DBTO cannot efficiently react with electron-deficient or electron-neutral arenes using the methodology described in this paper: Another reason is that compared with TfO^- counter-ion, Br^- counter-ion used in this procedure was much easier to be exchanged with Tf_2N^-.

General procedure for competition experiments:

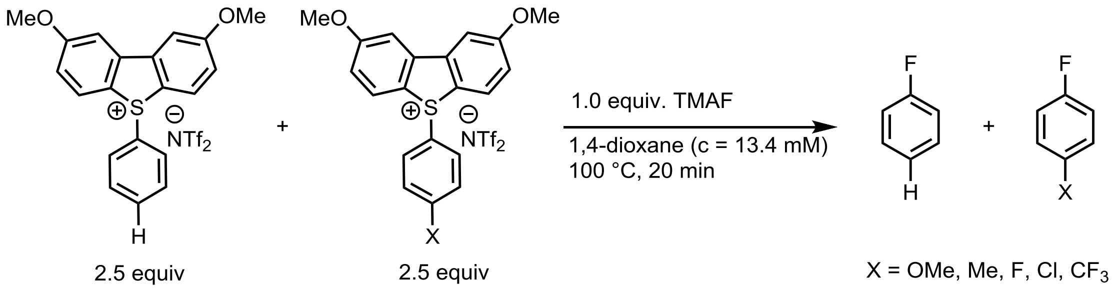

In a nitrogen-filled glovebox, a flame-dried J. Young NMR tube was charged with phenyl 2,8-dimethoxydibenzothiophenium salt (8.1 mg, 13.4 µmol, 2.50 equiv.), *para*-substituted phenyl 2,8-dimethoxydibenzothiophenium salt (13.4 µmol, 2.50 equiv.), and 1,4-dioxane (0.4 mL, c = 13.4 mM). The NMR tube was sealed, transferred out of the glovebox and sonicated for 5 min to afford a clear solution. The NMR tube was transferred back to the glovebox and stored in the fridge (-30 °C) for 10 min before addition of TMAF (0.5 mg, 5.37 µmol, 1.00 equiv.). Then the tube was sealed and transferred out from the glovebox and immediately placed in a preheated oil bath (100 °C). After 20 min, the NMR tube was taken out of the oil bath and placed in an acetone/dry ice bath (-78 °C) to quench the reaction. After warming to 25 °C, the reaction mixture was directly analyzed by ^{19}F NMR spectroscopy. The resulting ^{19}F resonance of the aryl fluoride (4-F-C_6H_4-X) was integrated relatively to the ^{19}F resonance of fluorobenzene (δ = –112.96 ppm) to calculate the ratio of the products. The values of $\log(k_X/k_H)$ were plotted versus the σ_p and σ_p^- value and a better Hammett correlation (Hammett-slope ρ = 3.3827) was obtained with σ_p values (**Figures S28**-**29**).

Table S14. Data for Hammett-Plot.

Substituent R	σ_p	σ_p^-	k_X/k_H	$\log(k_X/k_H)$
CF_3	0.54	0.65	17.61	1.25
Cl	0.23	0.19	3.48	0.54
F	0.06	–0.03	0.37	–0.43
H	0.00	0.00	1.00	0.00
Me	–0.17	–0.17	0.082	–1.08
OMe	–0.27	–0.27	0.033	–1.49

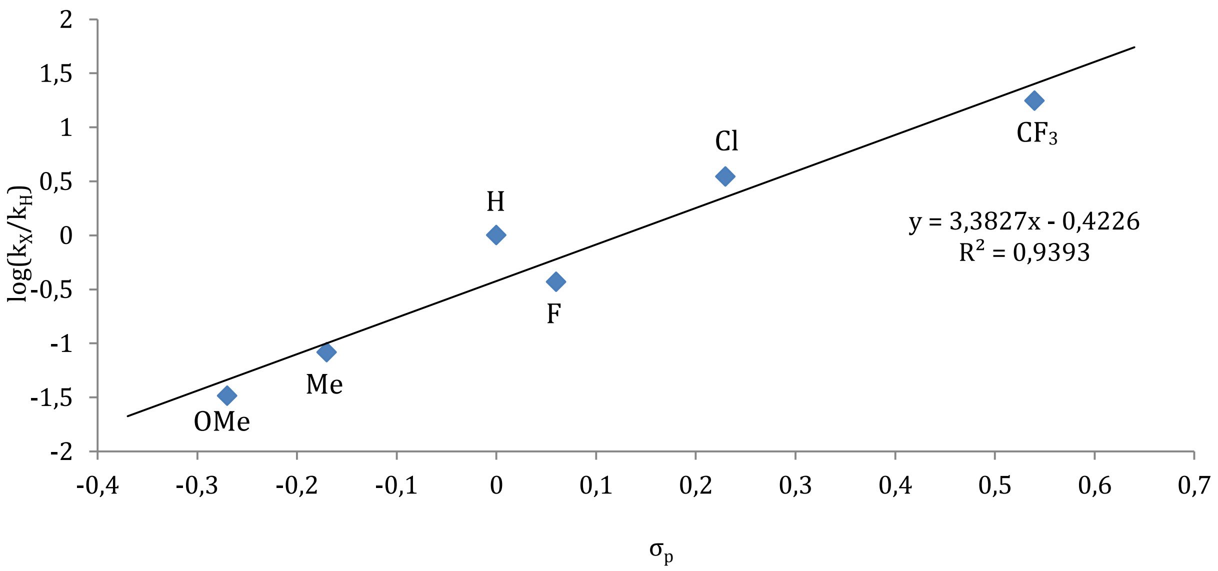

Figure S28. Hammett correlation using the σ-value of the *para*-substituent.

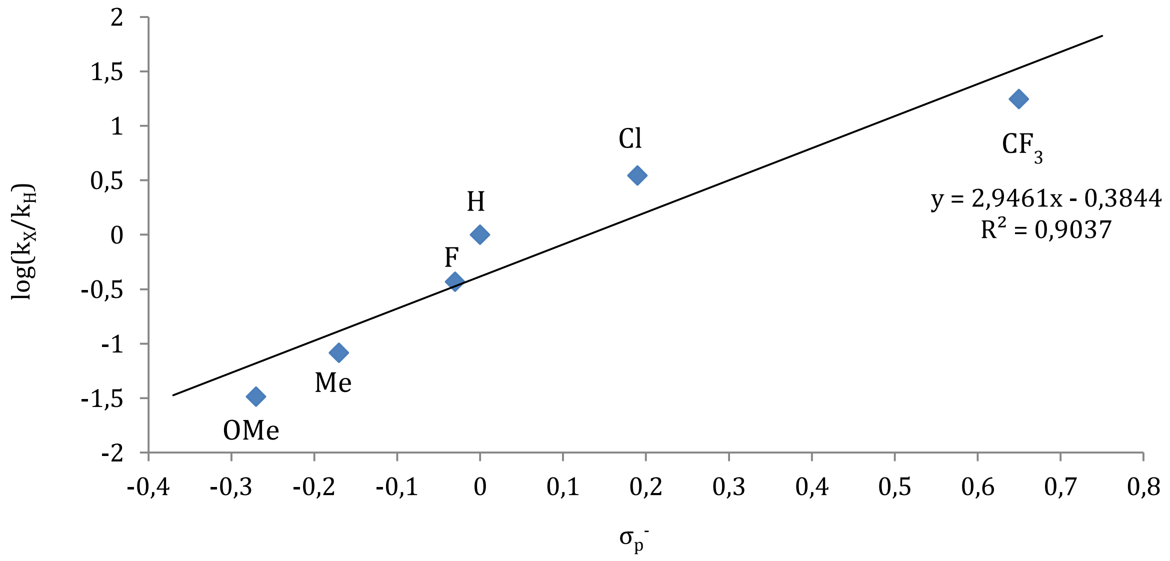

Figure S29. Hammett correlation using the σ_p^--value of the *para*-substituent.

General procedure for *in situ* preparation of aryl Grignard reagents

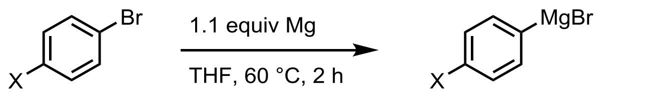

X = OMe, Me, H, F, Cl, CF_3
S39a-S44a

A flame-dried, 10 mL nitrogen-filled *Schlenk*-tube equipped with a magnetic stir bar was charged with aryl

bromide (3.00 mmol, 1.00 equiv.), magnesium turnings (80.2 mg, 3.30 mmol, 1.10 equiv.), and dry THF (3.0 mL, c = 1.0 M) at 25 °C. The reaction mixture was heated to 60 °C and stirred at the same temperature for 2 h. After cooled to 25 °C, the freshly prepared Grignard reagent (**S39a**-**S40a**) was directly used for the next step, and the concentration was treated as 1.0 M.

Anisole-derived 2,8-dimethoxydibenzothiophenium salt S39

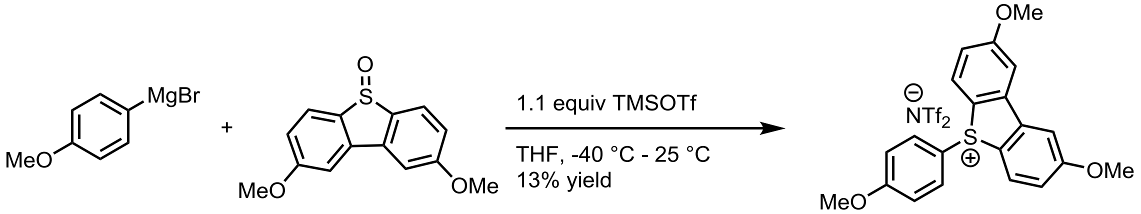

S39a **M-DBTO** **S39**

A flame-dried, 4 mL nitrogen-filled borosilicate vial equipped with a magnetic stir bar was charged with 2,8-dimethoxydibenzothiophene *S*-oxide (**M-DBTO**) (130 mg, 0.500 mmol, 1.00 equiv.) and dry THF (1.0 mL) at 25 °C. After cooling to –40 °C (acetonitrile/dry ice bath), TMSOTf (100 µL, 122 mg, 0.550 mmol, 1.10 equiv.) was added to the reaction mixture dropwise. After stirring for 15 min at –40 °C, freshly prepared 4-methoxyphenyl magnesium bromide (**S39a**) (1.0 M THF solution, 0.500 mL, 0.500 mmol, 1.00 equiv.) was added dropwise into the reaction mixture. The reaction mixture was then allowed to slowly warm to 25 °C over 1 h and stirred at the same temperature for another 20 min. Then the reaction mixture was cooled to –78 °C (acetone/dry ice bath) and quenched by dropwise addition of aqueous HBr (0.5 M, 1.0 mL). After addition, the reaction mixture was warmed to 25 °C in air, and additional 1.0 mL 0.5 M aqueous HBr was added. The mixture was poured into a separatory funnel, and 5 mL DCM was added. The DCM layer was collected, and the aqueous layer was further extracted with DCM (4 × ca. 10 mL). The combined DCM layer was dried over Na_2SO_4, filtered, and the solvent was removed under reduced pressure. The residue was purified by chromatography on silica gel eluting with DCM/MeOH (100:0 to 20:1, v/v) to afford an off white solid. The solid was re-dissolved in 10 mL DCM, and the resulting DCM solution was sequentially washed with aqueous $LiNTf_2$ solution (2 × ca. 5 mL, 10 % w/w) and water (5 mL). The DCM layer was dried over Na_2SO_4, filtered, and the solvent was removed under reduced pressure to afford anisole-derived 2,8-dimethoxydibenzothiophenium salt **S39** (40.0 mg, 63.3 µmol, 13 %) as a colorless solid.

R*f* = 0.51 (DCM/MeOH, 9:1, v/v).

NMR Spectroscopy:

^{1}H NMR (500 MHz, $CDCl_3$, 298 K, δ): 7.78 (d, *J* = 8.9 Hz, 2H), 7.62 (d, *J* = 2.6 Hz, 2H), 7.46 (d, *J* = 9.1 Hz, 2H), 7.11 (dd, *J* = 8.9, 2.5 Hz, 2H), 7.00 (d, *J* = 9.1 Hz, 2H), 4.00 (s, 6H), 3.84 (s, 3H) ppm.

^{13}C NMR (126 MHz, $CDCl_3$, 298 K, δ): 165.2, 165.0, 140.9, 132.6, 129.1, 123.0, 120.1 (q, *J* = 321.9 Hz, CF_3), 118.6, 117.4, 115.8, 109.2, 56.6, 56.2 ppm.

^{19}F NMR (471 MHz, $CDCl_3$, 298 K, δ): –78.7 (s) ppm.

HRMS-ESI (m/z) calculated for $C_{21}H_{19}O_3S^+$ [M-NTf$_2$]$^+$, 351.1049; found, 351.1046; deviation: 1.0 ppm.

Toluene-derived 2,8-dimethoxydibenzothiophenium salt S40

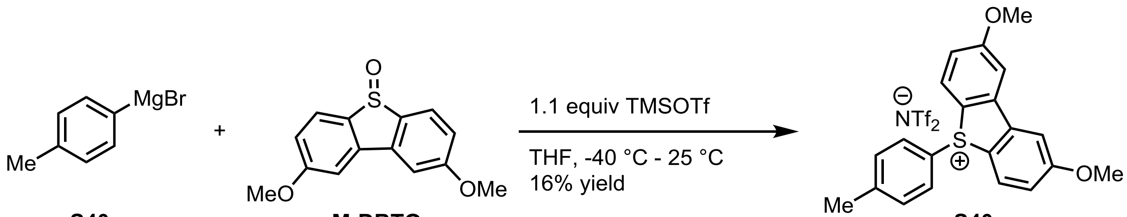

S40a **M-DBTO** **S40**

A flame-dried, 4 mL nitrogen-filled borosilicate vial equipped with a magnetic stir bar was charged with 2,8-dimethoxydibenzothiophene *S*-oxide (**M-DBTO**) (130 mg, 0.500 mmol, 1.00 equiv.) and dry THF (1.0 mL) at 25 °C. After cooling to –40 °C (acetonitrile/dry ice bath), TMSOTf (100 µL, 122 mg, 0.550 mmol, 1.10 equiv.) was added to the reaction mixture dropwise. After stirring for 15 min at –40 °C, freshly prepared 4-methylphenyl magnesium bromide (**S40a**) (1.0 M THF solution, 0.500 mL, 0.500 mmol, 1.00 equiv.) was added dropwise into the reaction mixture. The reaction mixture was then allowed to slowly warm to 25 °C over 1 h and stirred at the same temperature for another 20 min. Then the reaction mixture was cooled to –78 °C (acetone/dry ice bath) and quenched by dropwise addition of aqueous HBr (0.5 M, 1.0 mL). After addition, the reaction mixture was warmed to 25 °C in air, and additional 1.0 mL 0.5 M aqueous HBr was added. The mixture was poured into a separatory funnel, and 5 mL DCM was added. The DCM layer was collected, and the aqueous layer was further extracted with DCM (4 × ca. 10 mL). The combined DCM layer was dried over Na_2SO_4, filtered, and the solvent was removed under reduced pressure. The residue was purified by chromatography on silica gel eluting with DCM/MeOH (100:0 to 20:1, v/v) to afford an off white solid. The solid was re-dissolved in 10 mL DCM, and the resulting DCM solution was sequentially washed with aqueous $LiNTf_2$ solution (2 × ca. 5 mL, 10 % w/w) and water (5 mL). The DCM layer was dried over Na_2SO_4, filtered, and the solvent was removed under reduced pressure to afford toluene-derived 2,8-dimethoxydibenzothiophenium salt **S40** (50.0 mg, 81.2 µmol, 16 %) as a colorless solid.

R*f* = 0.37 (DCM/MeOH, 9:1, v/v).

NMR Spectroscopy:

^{1}**H NMR** (500 MHz, CD_3CN, 298 K, δ): 7.87 (d, *J* = 8.9 Hz, 2H), 7.84 (d, *J* = 2.6 Hz, 2H), 7.45 – 7.36 (m, 4H), 7.21 (dd, *J* = 8.9, 2.6 Hz, 2H), 3.99 (s, 6H), 2.38 (s, 3H) ppm.

^{13}**C NMR** (126 MHz, CD_3CN, 298 K, δ): 165.8, 147.7, 142.5, 133.0, 130.9, 129.9, 125.5, 123.9, 120.9 (q, *J* = 321.0 Hz, CF_3), 119.3, 110.4, 57.3, 21.6 ppm.

^{19}**F NMR** (471 MHz, CD_3CN, 298 K, δ): –80.2 (s) ppm.

HRMS-ESI (m/z) calculated for $C_{21}H_{19}O_2S^+$ [M-NTf$_2$]$^+$, 335.1100; found, 335.1096; deviation: 1.4 ppm.

Benzene-derived 2,8-dimethoxydibenzothiophenium salt S41

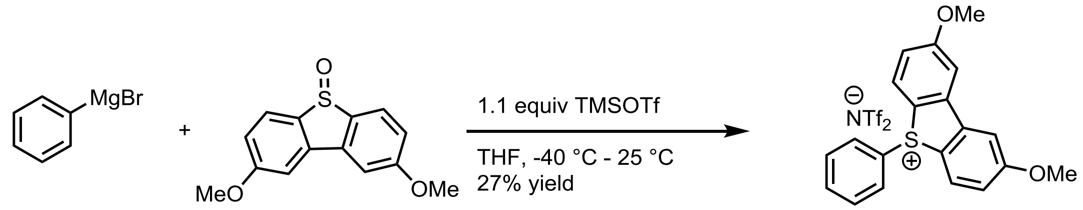

A flame-dried, 4 mL nitrogen-filled borosilicate vial equipped with a magnetic stir bar was charged with 2,8-dimethoxydibenzothiophene *S*-oxide (**M-DBTO**) (130 mg, 0.500 mmol, 1.00 equiv.) and dry THF (1.0 mL) at 25 °C. After cooling to –40 °C (acetonitrile/dry ice bath), (100 µL, 122 mg, 0.550 mmol, 1.10 equiv.) was added to the reaction mixture dropwise. After stirring for 15 min at –40 °C, freshly prepared phenyl magnesium bromide (**S41a**) (1.0 M THF solution, 0.500 mL, 0.500 mmol, 1.00 equiv.) was added dropwise into the reaction mixture. The reaction mixture was then allowed to slowly warm to 25 °C over 1 h and stirred at the same temperature for another 20 min. Then the reaction mixture was cooled to –78 °C (acetone/dry ice bath) and quenched by dropwise addition of aqueous HBr (0.5 M, 0.5 mL). After addition, the mixture was warmed to 25 °C in air, and additional 5.0 mL 0.5 M aqueous HBr was added. The mixture was poured into a separatory funnel, and 5 mL DCM was added. The DCM layer was collected, and the aqueous layer was further extracted with DCM (4 × ca. 10 mL). The combined DCM layer was dried over Na_2SO_4, filtered, and the solvent was removed under reduced pressure. The residue was purified by chromatography on silica gel eluting with DCM/MeOH (100:0 to 30:1, v/v) to afford an off white solid. The solid was re-dissolved in 10 mL DCM, and the resulting DCM solution was sequentially washed with aqueous $LiNTf_2$ solution (2 × ca. 5 mL, 10 % w/w) and water (5 mL). The DCM layer was dried over Na_2SO_4, filtered, and the solvent was removed under reduced pressure to afford benzene-derived 2,8-dimethoxydibenzothiophenium salt **S41** (82.0 mg, 136 µmol, 27 %) as a colorless solid.

R*f* = 0.38 (DCM/MeOH, 9:1, v/v).

NMR Spectroscopy:

^{1}H NMR (500 MHz, $CDCl_3$, 298 K, δ): 7.85 (d, *J* = 8.9 Hz, 2H), 7.68–7.61 (m, 3H), 7.58–7.50 (m, 4H), 7.13 (dd, *J* = 8.9, 2.5 Hz, 2H), 4.01 (s, 6H) ppm.

^{13}C NMR (126 MHz, $CDCl_3$, 298 K, δ): 165.1, 141.3, 135.1, 131.8, 130.1, 129.4, 127.8, 122.0, 120.1 (q, *J* = 321.4 Hz, CF_3), 118.6, 109.4, 56.7 ppm.

^{19}F NMR (471 MHz, $CDCl_3$, 298 K, δ): –78.7 (s) ppm.

HRMS-ESI (m/z) calculated for $C_{20}H_{17}O_2S^+$ [M-NTf_2]$^+$, 321.0944; found, 321.0939; deviation: 1.4 ppm.

Fluorobenzene-derived 2,8-dimethoxydibenzothiophenium salt S42

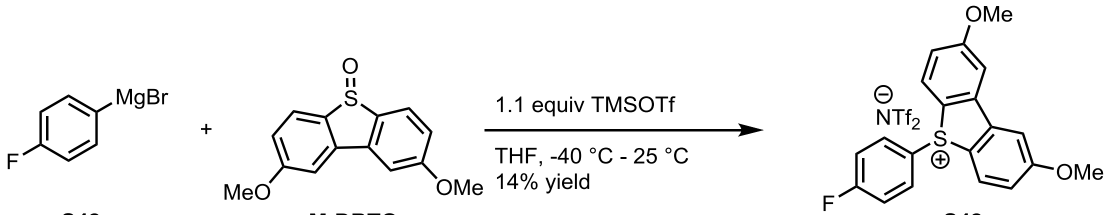

S42a M-DBTO S42

A flame-dried, 4 mL nitrogen-filled borosilicate vial equipped with a magnetic stir bar was charged with 2,8-dimethoxydibenzothiophene *S*-oxide (**M-DBTO**) (130 mg, 0.500 mmol, 1.00 equiv.) and dry THF (1.0 mL) at 25 °C. After cooling to –40 °C (acetonitrile/dry ice bath), TMSOTf (100 µL, 122 mg, 0.550 mmol, 1.10 equiv.) was added to the reaction mixture dropwise. After stirring for 15 min at –40 °C, freshly prepared 4-fluorophenyl magnesium bromide (**S42a**) (1.0 M THF solution, 0.500 mL, 0.500 mmol, 1.00 equiv.) was added dropwise into the reaction mixture. The reaction mixture was then allowed to slowly warm to 25 °C over 1 h and stirred at the same temperature for another 20 min. Then the reaction mixture was cooled to –78 °C (acetone/dry ice bath) and quenched by dropwise addition of aqueous HBr (0.5 M, 0.5 mL). After addition, the mixture was warmed to 25 °C in air, and additional 5.0 mL 0.5 M aqueous HBr was added. The mixture was poured into a separatory funnel, and 5 mL DCM was added. The DCM layer was collected, and the aqueous layer was further extracted with DCM (4 × ca. 10 mL). The combined DCM layer was dried over Na_2SO_4, filtered, and the solvent was removed under reduced pressure. The residue was purified by chromatography on silica gel eluting with DCM/MeOH (100:0 to 30:1, v/v) to afford a solid. The solid was re-dissolved in 10 mL DCM, and the resulting DCM solution was sequentially washed with aqueous $LiNTf_2$ solution (2 × ca. 5 mL, 10 % w/w) and water (5 mL). The DCM layer was dried over Na_2SO_4, filtered, and the solvent was removed under reduced pressure to afford fluorobenzene-derived 2,8-dimethoxydibenzothiophenium salt **S42** (44.0 mg, 71.0 µmol, 14 %) as a yellowish solid.

R*f* = 0.38 (DCM/MeOH, 9:1, v/v).

NMR Spectroscopy:

^{1}H NMR (500 MHz, CD_2Cl_2, 298 K, δ): 7.84 (d, *J* = 8.9 Hz, 2H), 7.67 (d, *J* = 2.6 Hz, 2H), 7.61–7.56 (m, 2H), 7.28 (dd, *J* = 9.1, 7.9 Hz, 2H), 7.20 (dd, *J* = 8.9, 2.5 Hz, 2H), 4.02 (s, 6H) ppm.

^{13}C NMR (126 MHz, CD_2Cl_2, 298 K, δ): 167.0 (d, *J* = 259.6 Hz), 165.6, 141.5, 133.3 (d, *J* = 10.1 Hz), 129.5, 122.9 (d, *J* = 3.4 Hz), 122.1, 120.3 (q, *J* = 322.0 Hz, CF_3), 119.7 (d, *J* = 23.7 Hz), 119.0, 109.8, 57.0 ppm.

^{19}F NMR (471 MHz, CD_2Cl_2, 298 K, δ): –79.4 (s), –100.5 (m) ppm.

HRMS-ESI (m/z) calculated for $C_{20}H_{16}O_2SF^+$ $[M-NTf_2]^+$, 339.0850; found, 339.0846; deviation: 1.1 ppm.

Chlorobenzene-derived 2,8-dimethoxydibenzothiophenium salt S43

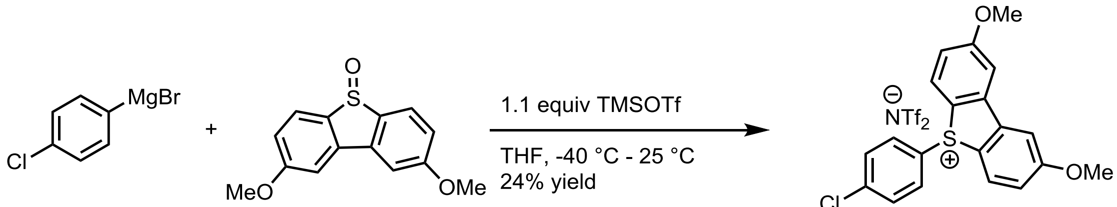

S43a **M-DBTO** **S43**

A flame-dried, 4 mL nitrogen-filled borosilicate vial equipped with a magnetic stir bar was charged with 2,8-dimethoxydibenzothiophene *S*-oxide (**M-DBTO**) (130 mg, 0.500 mmol, 1.00 equiv.) and dry THF (1.0 mL) at 25 °C. After cooling to –40 °C (acetonitrile/dry ice bath), TMSOTf (100 µL, 122 mg, 0.550 mmol, 1.10 equiv.) was added to the reaction mixture dropwise. After stirring for 15 min at –40 °C, freshly prepared 4-chlorophenyl magnesium bromide (**S43a**) (1.0 M THF solution, 0.500 mL, 0.500 mmol, 1.00 equiv.) was added dropwise into the reaction mixture. The reaction mixture was then allowed to slowly warm to 25 °C over 1 h and stirred at the same temperature for another 20 min. Then the reaction mixture was cooled to –78 °C (acetone/dry ice bath) and quenched by dropwise addition of aqueous HBr (0.5 M, 0.5 mL). After addition, the mixture was warmed to 25 °C in air, and additional 5.0 mL 0.5 M aqueous HBr was added. The mixture was poured into a separatory funnel, and 5 mL DCM was added. The DCM layer was collected, and the aqueous layer was further extracted with DCM (4 × ca. 10 mL). The combined DCM layer was dried over Na_2SO_4, filtered, and the solvent was removed under reduced pressure. The residue was purified by chromatography on silica gel eluting with DCM/MeOH (100:0 to 30:1, v/v) to afford a solid. The solid was re-dissolved in 10 mL DCM, and the resulting DCM solution was sequentially washed with aqueous $LiNTf_2$ solution (2 × ca. 5 mL, 10 % w/w) and water (5 mL). The DCM layer was dried over Na_2SO_4, filtered, and the solvent was removed under reduced pressure to afford chlorobenzene-derived 2,8-dimethoxydibenzothiophenium salt **S43** (76.0 mg, 119 µmol, 24 %) as a colorless solid.

R*f* = 0.41 (DCM/MeOH, 9:1, v/v).

NMR Spectroscopy:

^{1}H NMR (500 MHz, CD_2Cl_2, 298 K, δ): 7.85 (d, *J* = 8.9 Hz, 2H), 7.68 (d, *J* = 2.5 Hz, 2H), 7.57–7.53 (m, 2H), 7.53–7.47 (m, 2H), 7.19 (dd, *J* = 8.9, 2.5 Hz, 2H), 4.02 (s, 6H) ppm.

^{13}C NMR (126 MHz, CD_2Cl_2, 298 K, δ): 165.7, 142.5, 141.7, 132.3, 131.7, 129.5, 126.2, 121.8, 120.3 (q, *J* = 322.0 Hz, CF_3), 119.0, 109.8, 57.0 ppm.

^{19}F NMR (471 MHz, CD_2Cl_2, 298 K, δ): –79.4 (s) ppm.

HRMS-ESI (m/z) calculated for $C_{20}H_{16}O_2SCl^+$ $[M-NTf_2]^+$, 355.0554; found, 355.0549; deviation: 1.5 ppm.

Benzotrifluoride-derived 2,8-dimethoxydibenzothiophenium salt S44

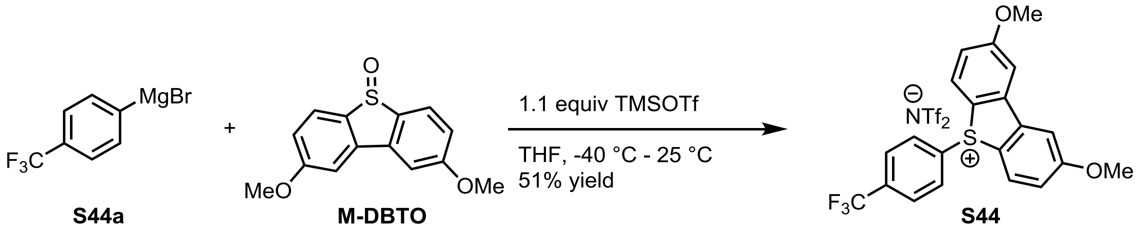

A flame-dried, 4 mL nitrogen-filled borosilicate vial equipped with a magnetic stir bar was charged with 2,8-dimethoxydibenzothiophene *S*-oxide (**M-DBTO**) (130 mg, 0.500 mmol, 1.00 equiv.) and dry THF (1.0 mL) at 25 °C. After cooling to –40 °C (acetonitrile/dry ice bath), TMSOTf (100 µL, 122 mg, 0.550 mmol, 1.10 equiv.) was added to the reaction mixture dropwise. After stirring for 15 min at –40 °C, freshly prepared 4-trifluoromethylphenyl magnesium bromide (**S44a**) (1.0 M THF solution, 0.500 mL, 0.500 mmol, 1.00 equiv.) was added dropwise into the reaction mixture. The reaction mixture was then allowed to slowly warm to 25 °C over 1 h and stirred at the same temperature for another 20 min. Then the reaction mixture was cooled to –78 °C (acetone/dry ice bath) and quenched by dropwise addition of aqueous HBr (0.5 M, 1.0 mL). After addition, the mixture was warmed to 25 °C in air, and additional 1.0 mL 0.5 M aqueous HBr was added. The mixture was poured into a separatory funnel, and 5 mL DCM was added. The DCM layer was collected, and the aqueous layer was further extracted with DCM (4 × ca. 10 mL). The combined DCM layer was dried over Na_2SO_4, filtered, and the solvent was removed under reduced pressure. The residue was purified by chromatography on silica gel eluting with DCM/MeOH (100:0 to 20:1, v/v) to afford an off white solid. The solid was re-dissolved in 10 mL DCM, and the resulting DCM solution was sequentially washed with aqueous $LiNTf_2$ solution (2 × ca. 5 mL, 10 % w/w) and water (5 mL). The DCM layer was dried over Na_2SO_4, filtered, and the solvent was removed under reduced pressure to afford benzotrifluoride-derived 2,8-dimethoxydibenzothiophenium salt **S44** (170 mg, 254 µmol, 51 %) as a colorless solid.

R*f* = 0.37 (DCM/MeOH, 9:1, v/v).

NMR Spectroscopy:

^{1}**H NMR** (500 MHz, CD_2Cl_2, 298 K, δ): 7.85 (d, *J* = 8.9 Hz, 2H), 7.68 (d, *J* = 2.5 Hz, 2H), 7.57–7.53 (m, 2H), 7.53–7.47 (m, 2H), 7.19 (dd, *J* = 8.9, 2.5 Hz, 2H), 4.02 (s, 6H) ppm.

^{13}**C NMR** (126 MHz, CD_2Cl_2, 298 K, δ): 165.8, 141.9, 136.7 (q, *J* = 33.2 Hz), 132.9, 130.9, 129.7, 128.9 (q, *J* = 3.7 Hz), 123.1 (q, *J* = 273.8 Hz, CF_3), 121.0, 120.3 (q, *J* = 321.8 Hz, CF_3), 119.1, 110.0, 57.0 ppm.

^{19}**F NMR** (471 MHz, CD_2Cl_2, 298 K, δ): –63.9 (s), –79.4 (s) ppm.

HRMS-ESI (m/z) calculated for $C_{21}H_{16}O_2SF_3^+$ $[M\text{-}NTf_2]^+$, 389.0818; found, 389.0814; deviation: 1.0 ppm.

Determination of Selectivity

Selectivity of dibenzothiophenylation of ethylbenzene was determined through independent synthesis of all

three isomers for accurate comparison.

Ethylbenzene-derived *para*-dibenzothiophenium salt S45

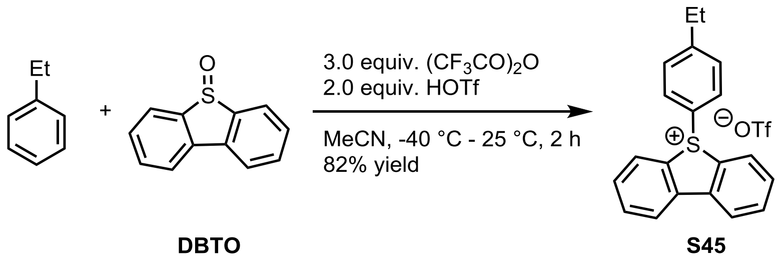

A flame-dried, 10 mL nitrogen-filled *Schlenk*-tube equipped with a magnetic stir bar was charged with ethylbenzene (53.1 mg, 0.500 mmol, 1.00 equiv.) and dry MeCN (2.0 mL, c = 0.25 M) at 25 °C. After cooling to –40 °C (acetonitrile/dry ice bath), trifluoromethanesulfonic acid (88.5 µL, 150 mg, 1.00 mmol, 2.00 equiv.) and trifluoroacetic anhydride (209 µL, 315 mg, 1.50 mmol, 3.00 equiv.) were added to the stirred reaction mixture. Subsequently, dibenzothiophene *S*-oxide (**DBTO**) (150 mg, 0.750 mmol, 1.50 equiv.) was added to the stirred reaction mixture in small portions over 1 min. After addition, the reaction mixture was stirred at –40 °C for 1 h. Subsequently, the *Schlenk*-tube was taken out of the cold bath and warmed to 25 °C in air. After stirring at 25 °C for another 1 h, the reaction mixture was diluted with DCM (10 mL) and poured onto saturated aqueous $NaHCO_3$ (10 mL). The mixture was concentrated under reduced pressure to remove most of the MeCN solvent, and the residue was diluted with 20 mL DCM and 10 mL water. The mixture was poured into a separatory funnel, and the layers were separated. The DCM layer was collected, and the aqueous layer was further extracted with DCM (4 × ca. 30 mL). The combined DCM layer was dried over Na_2SO_4, filtered, and the solvent was removed under reduced pressure. The residue was purified by chromatography on silica gel eluting with DCM/MeOH (50:1 to 30:1, v/v). The product was collected and dried *in vacuo* to afford ethylbenzene-derived *para*-dibenzothiophenium salt **S45** (180 mg, 411 µmol, 82%) as an off white solid.

R*f* = 0.34 (DCM/MeOH, 9:1, v/v).

NMR Spectroscopy:

^{1}H NMR (500 MHz, CD_3CN, 298 K, δ): 8.38–8.32 (m, 2H), 7.93 (td, *J* = 7.7, 1.1 Hz, 2H), 7.76–7.68 (m, 2H), 7.52–7.47 (m, 2H), 7.43 (d, *J* = 8.5 Hz, 2H), 2.68 (q, *J* = 7.6 Hz, 2H), 1.16 (t, *J* = 7.6 Hz, 3H) ppm.

^{13}C NMR (126 MHz, CD_3CN, 298 K, δ): 153.9, 140.3, 135.4, 133.2, 132.6, 132.1, 131.6, 128.8, 125.5, 124.2, 122.1 (q, *J* = 321.2 Hz, CF_3), 29.3, 15.3 ppm.

^{19}F NMR (471 MHz, CD_3CN, 298 K, δ): –79.2 (s) ppm.

HRMS-ESI (m/z) calculated for $C_{20}H_{17}S^+$ $[M-OTf]^+$, 289.1045; found, 289.1042; deviation: 1.1 ppm.

Ethylbenzene-derived *ortho*-dibenzothiophenium salt S46

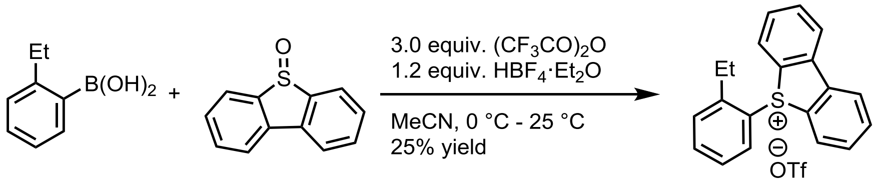

DBTO **S46**

A 4-mL borosilicate vial equipped with a magnetic stir bar was charged with 2-ethylphenylboronic acid (30.0 mg, 0.200 mmol, 1.00 equiv.), dibenzothiophene *S*-oxide (**DBTO**) (40.0 mg, 0.200 mmol, 1.00 equiv.), and dry MeCN (800 µL, c = 0.25 M) at 25 °C under air. After cooling to 0 °C (water/ice bath), trifluoroacetic anhydride (84.6 µL, 126 mg, 0.600 mmol, 3.00 equiv.) and $HBF_4 \cdot OEt_2$ (34.8 µL, 0.240 mmol, 1.20 equiv.) were added in sequence to the stirred reaction mixture. After addition, the reaction mixture was stirred at 0 °C for 20 min. Subsequently, the *Schlenk*-tube was taken out of the cold bath and warmed to 25 °C in air. After stirring at 25 °C for another 2 h, the reaction mixture was evaporated to dryness, and the residue was dissolved in DCM (10 mL) and poured onto 1.0 M aqueous Na_2CO_3 solution (ca. 2 mL). The mixture was poured into a separatory funnel, and the DCM layer was separated and washed with water (ca. 2 mL). The DCM layer was dried over Na_2SO_4, filtered, and the solvent was removed under reduced pressure. The residue was purified by chromatography on silica gel eluting with DCM/MeOH (100:0 to 30:1, v/v) to afford a solid. The solid re-dissolved in 10 mL DCM was washed with aqueous LiOTf solution (2 × ca. 5 mL, 10 % w/w) and water (5 mL). The DCM layer was dried over Na_2SO_4, filtered, and the solvent was removed under reduced pressure to afford ethylbenzene-derived *ortho*-dibenzothiophenium salt **S46** (22.0 mg, 50.2 µmol, 25%) as a colorless solid.

R*f* = 0.38 (DCM/MeOH, 9:1, v/v).

NMR Spectroscopy:

^{1}H NMR (500 MHz, $CDCl_3$, 298 K, δ): 8.21 (d, *J* = 7.8 Hz, 2H), 8.10 (d, *J* = 8.0 Hz, 2H), 7.87 (t, *J* = 7.7 Hz, 2H), 7.68 (t, *J* = 7.8 Hz, 2H), 7.60–7.54 (m, 2H), 7.17 (t, *J* = 7.3 Hz, 1H), 6.59 (d, *J* = 8.2 Hz, 1H), 3.48 (q, *J* = 8.0 Hz, 2H), 1.55 (t, *J* = 7.6 Hz, 3H) ppm.

^{13}C NMR (126 MHz, $CDCl_3$, 298 K, δ): 149.5, 139.2, 135.3, 134.6, 132.0, 131.9, 131.8, 129.6, 128.4, 128.4, 128.1, 128.1, 122.1 (q, *J* = 321.2 Hz, CF_3), 26.9, 16.4 ppm.

^{19}F NMR (471 MHz, $CDCl_3$, 298 K, δ): –78.2 (s) ppm.

HRMS-ESI (m/z) calculated for $C_{20}H_{17}S^+$ [M-OTf]$^+$, 289.1045; found, 289.1043; deviation: 1.0 ppm.

Ethylbenzene-derived *meta*-dibenzothiophenium salt S47

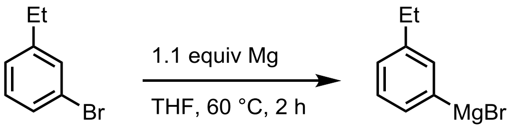

S47a

Grignard reagent **S47a** was freshly prepared by the following procedure: A flame-dried, 10 mL nitrogen-filled *Schlenk*-tube equipped with a magnetic stir bar was charged with 3-ethylbromobenzene (555 mg, 3.00 mmol, 1.00 equiv.), magnesium power (80.2 mg, 3.30 mmol, 1.10 equiv.), and dry THF (3.0 mL, c = 1.0 M) at 25 °C. The reaction mixture was heated to 60 °C and stirred at the same temperature for 2 h. The freshly prepared Grignard reagent **S47a** was used for next step without any further treatment, and the concentration was treated as 1.0 M.

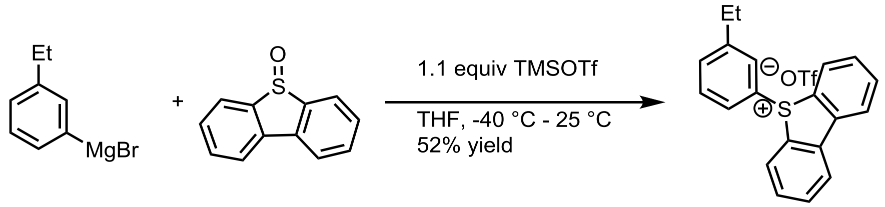

S47a **DBTO** **S47**

A flame-dried, 4 mL nitrogen-filled borosilicate vial equipped with a magnetic stir bar was charged with dibenzothiophene *S*-oxide (**DBTO**) (100 mg, 0.500 mmol, 1.00 equiv.) and dry THF (1.0 mL) at 25 °C. After cooling to –40 °C (acetonitrile/dry ice bath), TMSOTf (100 µL, 0.550 mmol, 1.10 equiv.) was added to the reaction mixture dropwise. After stirring for 15 min at –40 °C, freshly prepared 4-ethylphenyl magnesium bromide (**S47a**) (1M THF solution, 0.500 mL, 0.500 mmol, 1.00 equiv.) was added dropwise into the reaction mixture. The reaction mixture was then allowed to slowly warm to 25 °C over 1 h and stirred at the same temperature for another 20 min. The reaction mixture was cooled to –78 °C (acetone/dry ice bath) and quenched by dropwise addition of aqueous HBr (0.5 M, 1.0 mL). After addition, the mixture was warmed to 25 °C in air, and additional 1.0 mL 0.5 M aqueous HBr was added. The mixture was poured into a separatory funnel, and 5 mL DCM was added. The DCM layer was collected, and the aqueous layer was further extracted with DCM (4 × ca. 10 mL). The combined DCM layer was dried over Na_2SO_4, filtered, and the solvent was removed under reduced pressure. The residue was purified by chromatography on silica gel eluting with DCM/MeOH (100:0 to 20:1, v/v) to afford an off white solid. The solid re-dissolved in 10 mL DCM was washed with aqueous LiOTf solution (2 × ca. 5 mL, 10 % w/w) and water (5 mL). The DCM layer was dried over Na_2SO_4, filtered, and the solvent was removed under reduced pressure to afford ethylbenzene-derived *meta*-dibenzothiophenium salt **S47** (113 mg, 258 µmol, 52 %) as an off white solid.

R*f* = 0.37 (DCM/MeOH, 9:1, v/v).

NMR Spectroscopy:

^{1}H NMR (500 MHz, $CDCl_3$, 298 K, δ): 8.24 (d, *J* = 7.8 Hz, 2H), 8.08 (d, *J* = 8.0 Hz, 2H), 7.84 (t, *J* = 7.7 Hz, 2H), 7.64–7.56 (m, 3H), 7.45 (d, *J* = 7.7 Hz, 1H), 7.37 (t, *J* = 7.9 Hz, 1H), 7.22 (d, *J* = 8.1 Hz, 1H), 2.64 (q, *J* = 7.6 Hz, 2H), 1.16 (t, *J* = 7.6 Hz, 3H) ppm.

^{13}C NMR (126 MHz, $CDCl_3$, 298 K, δ): 148.7, 139.0, 134.8, 134.6, 131.8, 131.7, 131.6, 130.5, 128.5, 127.2, 126.1, 124.5, 121.0 (q, *J* = 321.2 Hz, CF_3), 29.3, 15.3 ppm.

^{19}F NMR (471 MHz, $CDCl_3$, 298 K, δ): –78.1 (s) ppm.

HRMS-ESI (m/z) calculated for $C_{20}H_{17}S^+$ [M-OTf]$^+$, 289.1045; found, 289.1042; deviation: 1.1 ppm.

Selectivity of dibenzothiophenylation of ethylbenzene

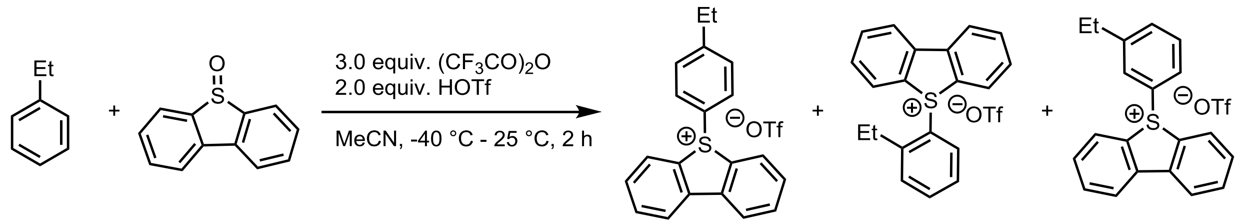

DBTO **S45** **S46** **S47**

The dibenzothiophenylation of ethylbenzene was performed on the standard reaction procedure.

A flame-dried, 10 mL nitrogen-filled *Schlenk*-tube equipped with a magnetic stir bar was charged with ethylbenzene (53.1 mg, 0.500 mmol, 1.00 equiv.) and dry MeCN (2.0 mL, c = 0.25 M) at 25 °C. After cooling to –40 °C (acetonitrile/dry ice bath), trifluoromethanesulfonic acid (88.5 μL, 150 mg, 1.00 mmol, 2.00 equiv.) and trifluoroacetic anhydride (209 μL, 315 mg, 1.50 mmol, 3.00 equiv.) were added to the stirred reaction mixture. Subsequently, dibenzothiophene *S*-oxide (**DBTO**) (150 mg, 0.750 mmol, 1.50 equiv.) was added to the stirred reaction mixture in small portions over 1 min. After addition, the reaction mixture was stirred at –40 °C for 1 h. Subsequently, the *Schlenk*-tube was taken out of the cold bath and warmed to 25 °C in air. After stirring at 25 °C for another 1 h, the reaction mixture was diluted with DCM (10 mL) and poured onto saturated aqueous $NaHCO_3$ (10 mL). The mixture was concentrated under reduced pressure to remove most of the MeCN solvent, and the residue was diluted with 20 mL DCM and 10 mL water. The mixture was poured into a separatory funnel, and the layers were separated. The DCM layer was collected, and the aqueous layer was further extracted with DCM (4 × ca. 30 mL). The combined DCM layer was dried over Na_2SO_4, filtered, and the solvent was removed under reduced pressure. The residue was analyzed by ^1H NMR spectroscopy and the obtained spectrum was compared with the spectra of the other two isomers. The ratio of isomers was determined by ^1H NMR spectroscopy.

Table S15. Selectivity determination of the dibenzothiophenylation of ethylbenzene.

	ratio = 0.5 × relative intensity of *para*-signal / relative intensity of *ortho*- or *meta*-signal
para/*ortho*	0.5 × 204 / 2.03 = 50 / 1
para/*meta*	0.5 × 204 / 1.00 = 102 / 1

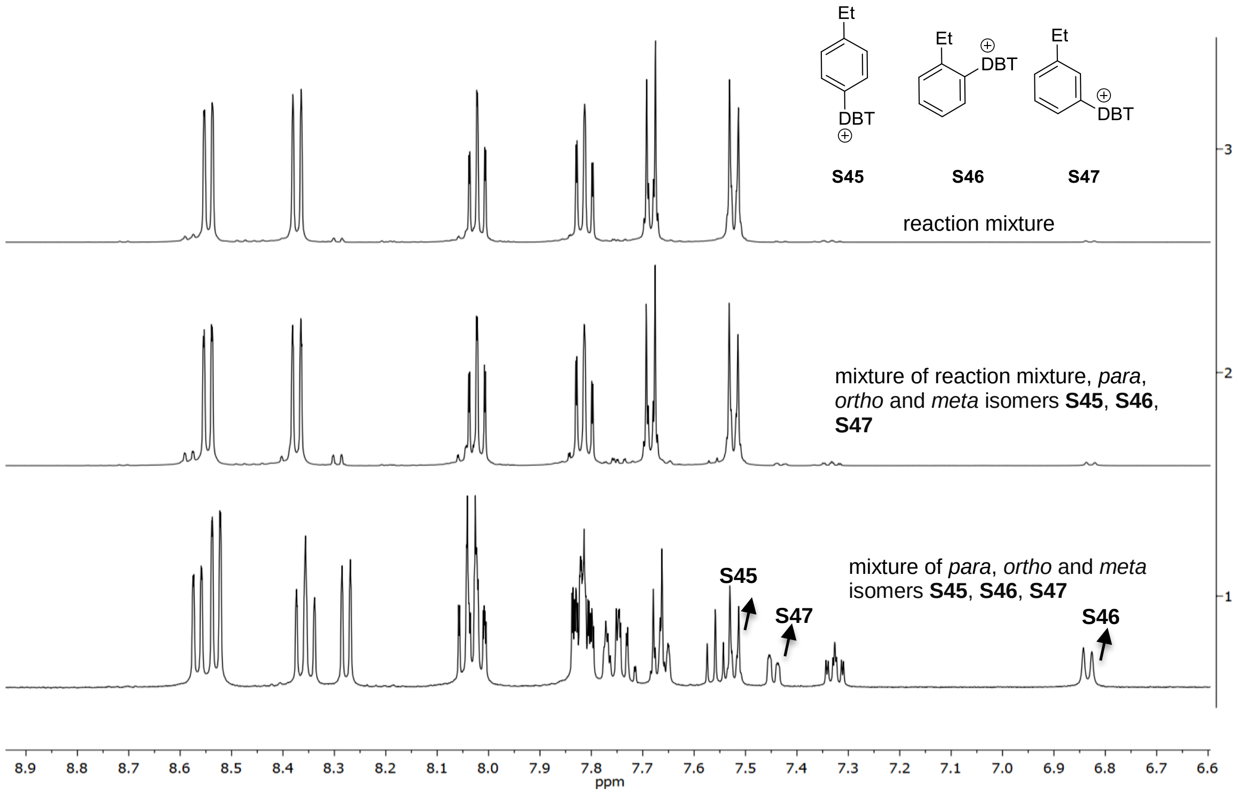

Figure S30: ^{1}H NMR of reaction mixture, in comparison to a mixture of *para*, *ortho* and *meta* isomers **S45**, **S46**, **S47**, acetone-d_6, 500 MHz, 298 K.

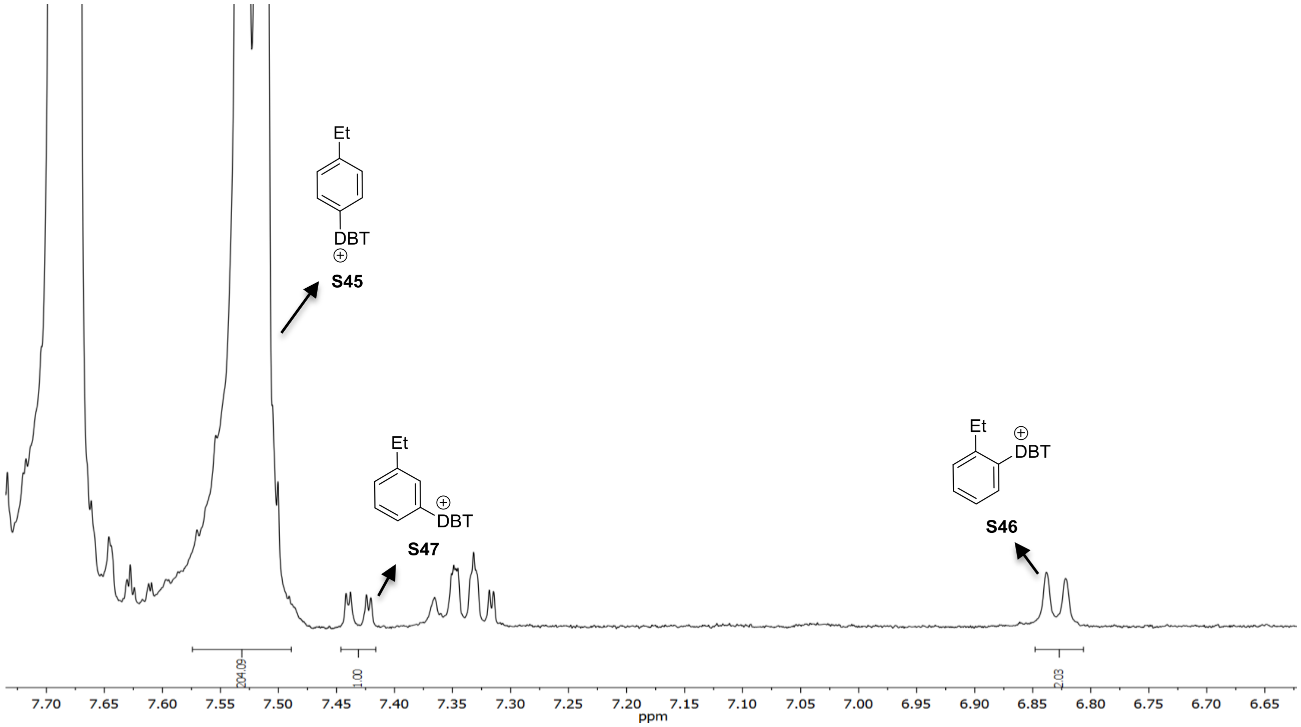

Figure S31: ^{1}H NMR of reaction mixture of ethylbenzene-derived dibenzothiophenium salt, acetone-d_6, 500 MHz, 298 K.

Example of dibenzothiophenylation of arene with moderate selectivity

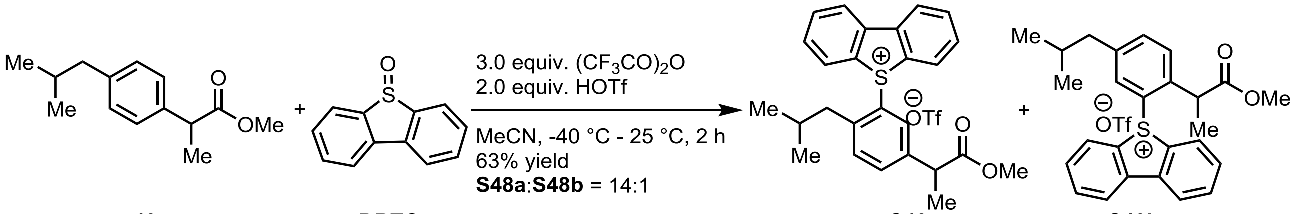

A flame-dried, 10 mL nitrogen-filled *Schlenk*-tube equipped with a magnetic stir bar was charged with ibuprofen methylester (**48**) (110 mg, 0.500 mmol, 1.00 equiv.) and dry MeCN (2.0 mL, c = 0.25 M) at 25 °C. After cooling to –40 °C (acetonitrile/dry ice bath), trifluoromethanesulfonic acid (88.5 µL, 150 mg, 1.00 mmol, 2.00 equiv.) and trifluoroacetic anhydride (209 µL, 315 mg, 1.50 mmol, 3.00 equiv.) were added to the stirred reaction mixture. Subsequently, dibenzothiophene *S*-oxide (**DBTO**) (150 mg, 0.750 mmol, 1.50 equiv.) was added to the stirred reaction mixture in small portions over 1 min. After addition, the reaction mixture was stirred at –40 °C for 1 h. Subsequently, the *Schlenk*-tube was taken out of the cold bath and warmed to 25 °C in air. After stirring at 25 °C for another 1 h, the reaction mixture was diluted with DCM (10 mL) and poured onto saturated aqueous $NaHCO_3$ (10 mL). The mixture was concentrated under reduced pressure to remove most of the MeCN solvent, and the residue was diluted with 20 mL DCM and 10 mL water. The mixture was poured into a separatory funnel, and the layers were separated. The DCM layer was collected, and the aqueous layer was further extracted with DCM (4 × ca. 30 mL). The combined DCM layer was dried over Na_2SO_4, filtered, and the solvent was removed under reduced pressure. The residue was purified by chromatography on silica gel eluting with DCM/MeOH (50:1 to 30:1, v/v). The product was collected and dried *in vacuo* to afford a mixture of ibuprofen methylester-derived dibenzothiophenium salt **S48a** and **S48b** (175 mg, 316 µmol, 63%) as a colorless solid. The structure of main product **S48a** and minor product **S48b** was determined by ^{1}H NMR, ^{13}C NMR, and NOESY spectrum, and the ratio (**S48a**:**S48b** = 14:1) was determined by ^{1}H NMR spectroscopy.

The following data were obtained for the mixture of **S48a** and **S48b**:

R*f* = 0.42 (DCM/MeOH, 9:1, v/v).

NMR Spectroscopy:

^{1}H NMR (500 MHz, $CDCl_3$, 298 K, δ): 8.31–8.25 (m, 2.17H), 8.15 (d, *J* = 7.9 Hz, 0.07H), 7.92 (dd, *J* = 8.0, 3.7 Hz, 2.00H),7.89–7.85 (m, 1.97H), 7.84–7.79 (m, 0.22H), 7.67–7.63 (m, 2.01H), 7.61–7.56 (m, 0.18H), 7.50 (dd, *J* = 8.1, 1.7 Hz, 1.00H), 7.42 (d, *J* = 8.1 Hz, 1.00H), 7.33 (dd, *J* = 8.1, 1.6 Hz, 0.07H), 6.41 (brs, 0.88H), 6.12 (brs, 0.06H), 3.82 (s, 0.19H), 3.45–3.40 (m, 4.09H), 3.19 (brs, 1.91H), 2.11–2.00 (m, 1.09H), 1.85 (d, *J* = 6.7 Hz, 0.20H), 1.20–1.08 (m, 9.09H) ppm.

^{13}C NMR (126 MHz, $CDCl_3$, 298 K, δ): 174.2, 173.1, 145.8, 144.6, 142.4, 142.2, 139.0 (d, *J* = 4.3 Hz), 138.9, 135.9, 134.7, 134.4 (d, *J* = 6.0 Hz), 134.1, 133.2, 132.4, 131.9 (d, *J* = 4.8 Hz), 131.7, 131.6 (d, *J* = 3.4 Hz), 129.5, 129.1, 128.0, 127.8 (d, *J* = 4.8 Hz), 127.0 (brs), 126.4 (brs), 124.9, 124.8 (d, *J* = 2.8 Hz),

124.7 (d, J = 1.6 Hz), 120.8 (q, J = 321.1 Hz, CF_3), 53.3, 52.1, 44.2, 43.9, 41.8, 41.7, 31.7, 29.3, 22.2 (d, J = 2.5 Hz), 21.5 (d, J = 9.1 Hz), 18.0, 16.2 ppm.

^{19}F NMR (471 MHz, $CDCl_3$, 298 K, δ): –78.1 (s) ppm.

HRMS-ESI (m/z) calculated for $C_{26}H_{27}O_2S^+$ [M-OTf]$^+$, 403.1726; found, 403.1725; deviation: 0.3 ppm.

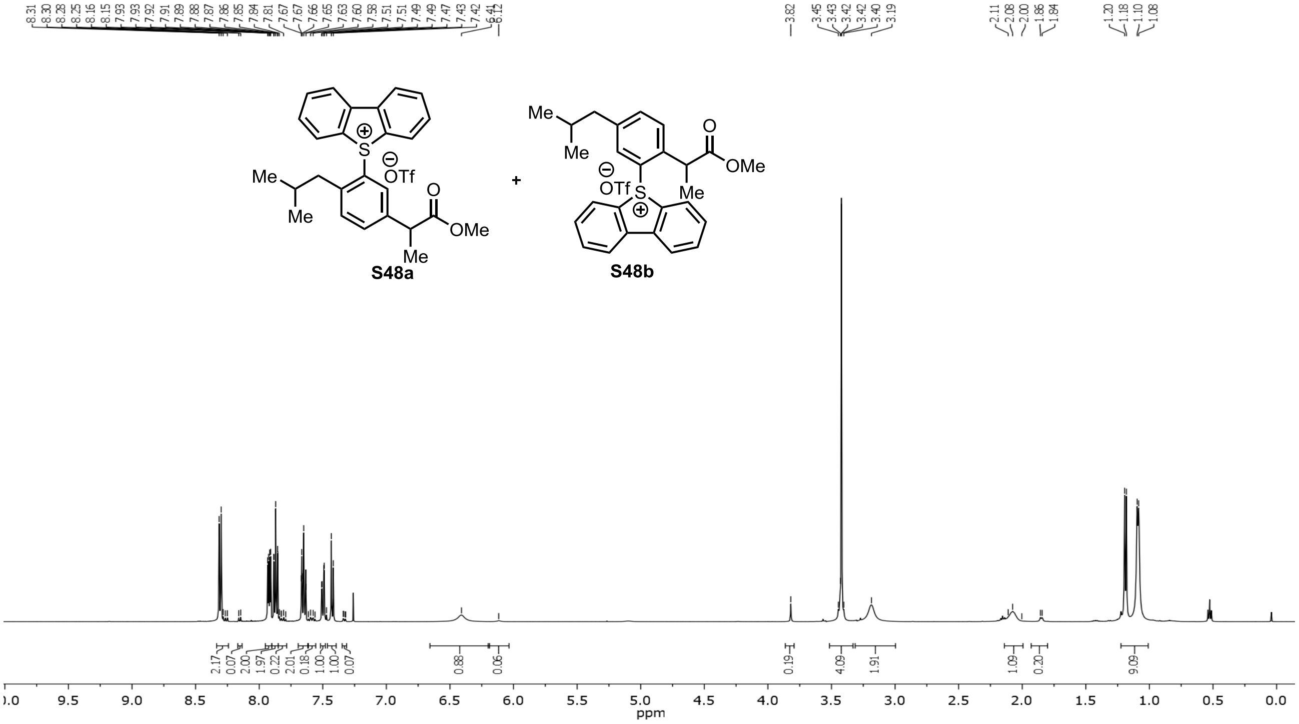

Figure S32: ^{1}H NMR of the mixture of **S48a** and **S48b**, $CDCl_3$, 500 MHz, 298 K.

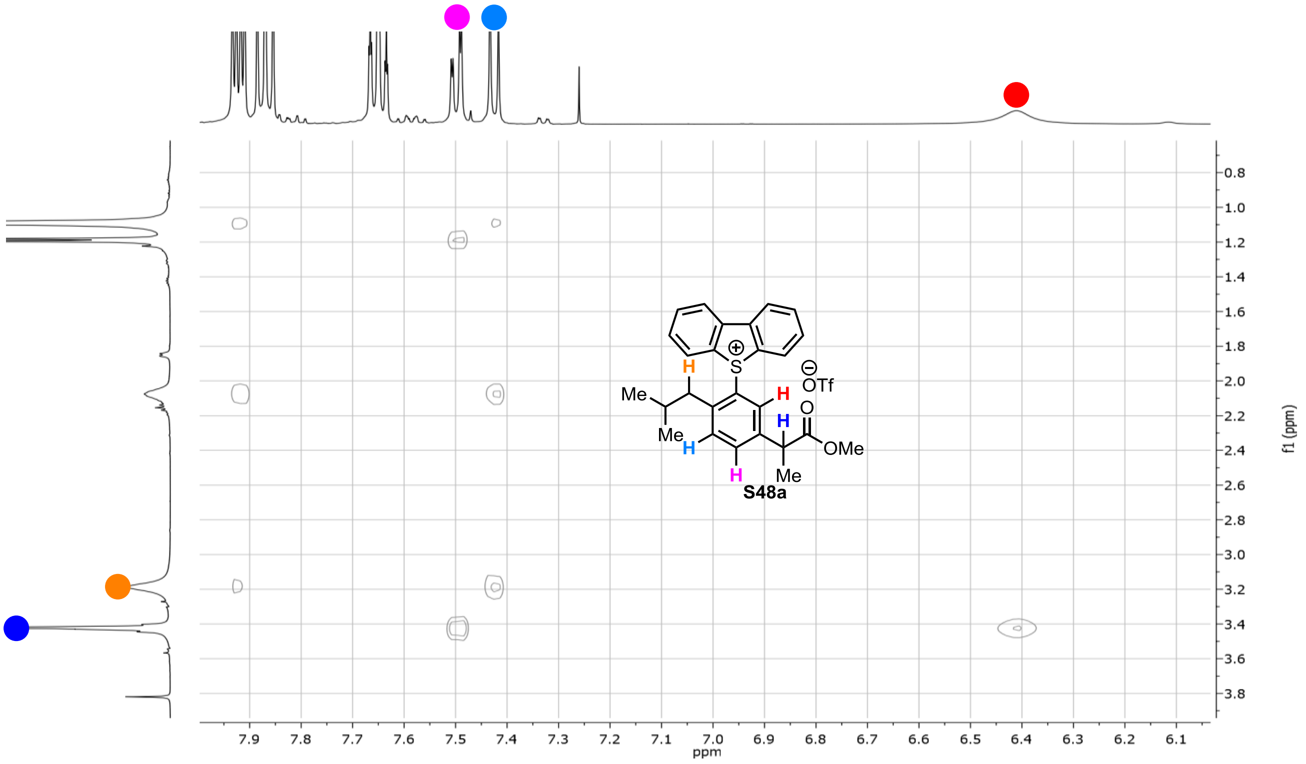

Figure S33: NOESY spectrum of the mixture of **S48a** and **S48b**, $CDCl_3$, 500 MHz, 298 K.

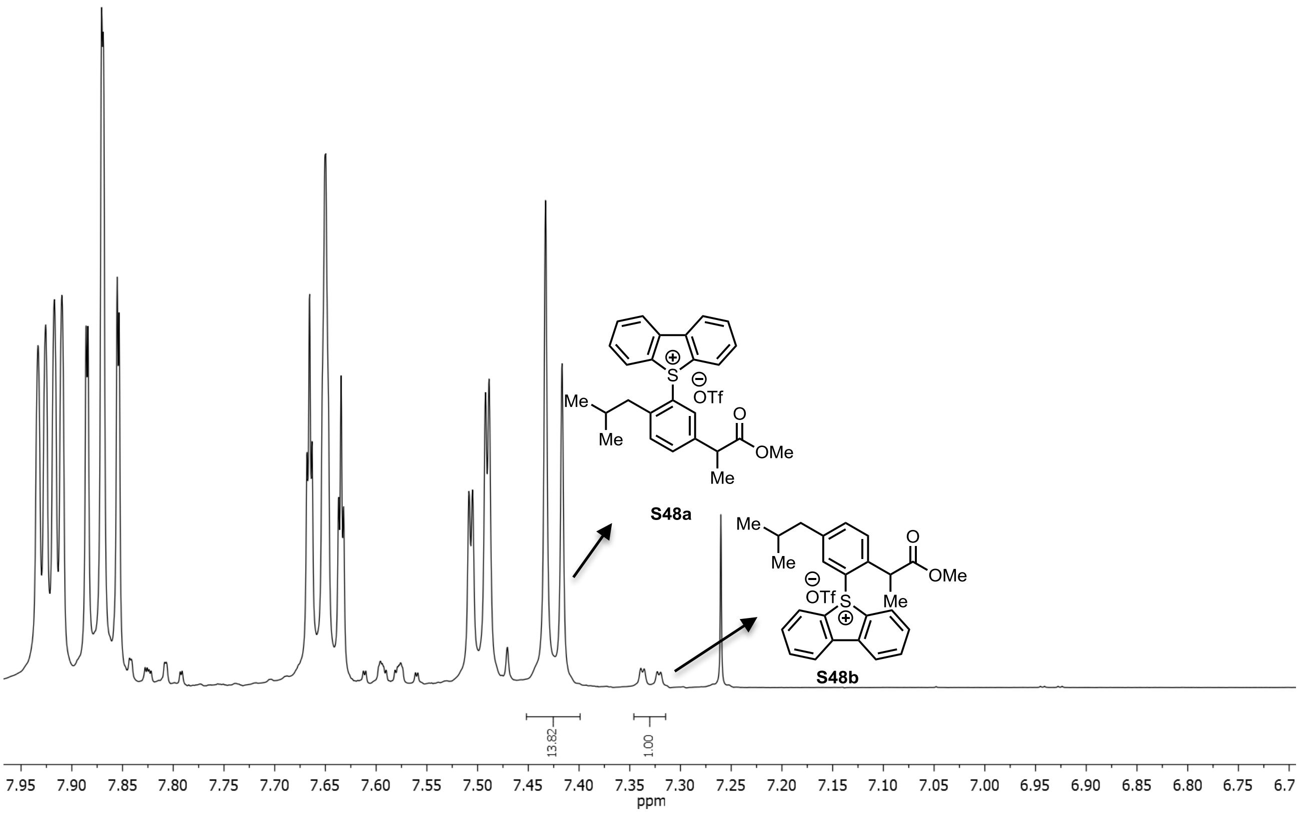

Figure S34: Ratio determined by ^{1}H NMR of the mixture of **S48a** and **S48b**, $CDCl_3$, 500 MHz, 298 K

REFERENCES

1. Fulmer, G. R.; Miller, A. J. M.; Sherden, N. H.; Gottlieb, H. E.; Nudelman, A.; Stoltz, B. M.; Bercaw, J. E.; Goldberg, K. I. NMR Chemical Shifts of Trace Impurities: Common Laboratory Solvents, Organics, and Gases in Deuterated Solvents Relevant to the Organometallic Chemist. *Organometallics* **2010**, *29*, 2176–2179.
2. Kawano, S.; Hamazaki, T.; Suzuki, A.; Kurahashi, K.; Tanaka, K. Metal-Ion-Induced Switch of Liquid-Crystalline Orientation of Metallomacrocycles. *Chem*. - *Eur*. *J*. **2016**, *22*, 15674–15683.
3. Wang, P.; Verma, P.; Xia, G.; Shi, J.; Qiao, J. X.; Tao, S.; Cheng, P. T. W.; Poss, M. A.; Farmer, M. E.; Yeung, K.-S.; Yu, J.-Q. Ligand-Accelerated non-Directed C–H Functionalization of Arenes. *Nature* **2017**, *551*, 489–493.
4. McCammant, S. M.; Thompson, S.; Brooks, A. F.; Krska, S. W.; Scott, P, J. H.; Sanford. M. S. Cu-Mediated C–H ^{18}F-Fluorination of Electron-Rich (Hetero)arenes. *Org. Lett.* **2017**, *19*, 3939−3942.
5. Li, J.; Chen, J.; Sang, R.; Ham, W.-S.; Plutschack, M. B.; Berger, F.; Chabbra, S.; Schnegg, A.; Genicot, C.; Ritter, T. Photoredox Catalysis with Thianthrenium Salts Enables Site-selective Late-stage

Fluorination. *Nat. Chem.* **2019**, *accepted*.

6. Waldecker, B.; Kraft, F.; Golz, C.; Alcarazo, M. 5-(Alkynyl)dibenzothiophenium Triflates: Sulfur-Based Reagents for Electrophilic Alkynylation. *Angew. Chem. Int. Ed.* **2018**, *57*, 12538−12542.

SPECTROSCOPIC DATA

^{1}H NMR of dibenzothiophene *S*-oxide (DBTO)

$CDCl_3$, 298 K

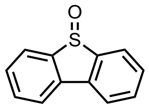

DBTO

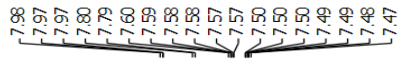
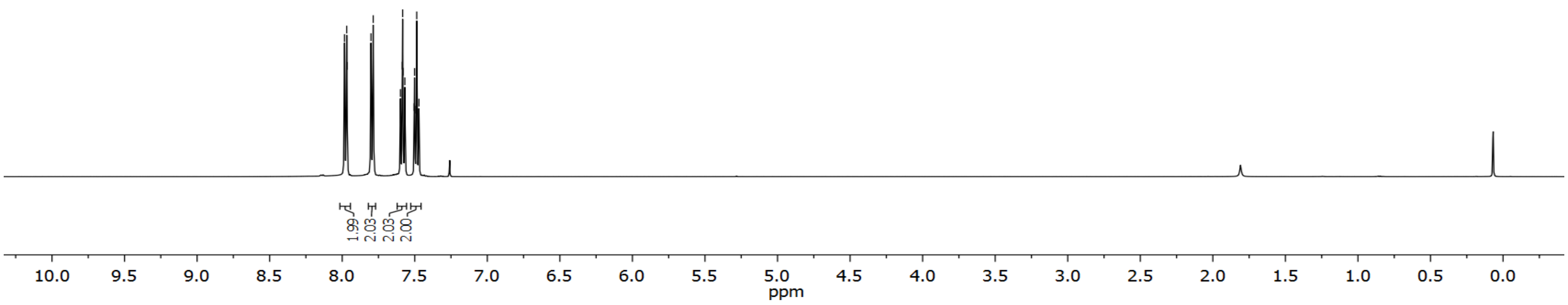

^{13}C NMR of dibenzothiophene *S*-oxide (DBTO)

$CDCl_3$, 298 K

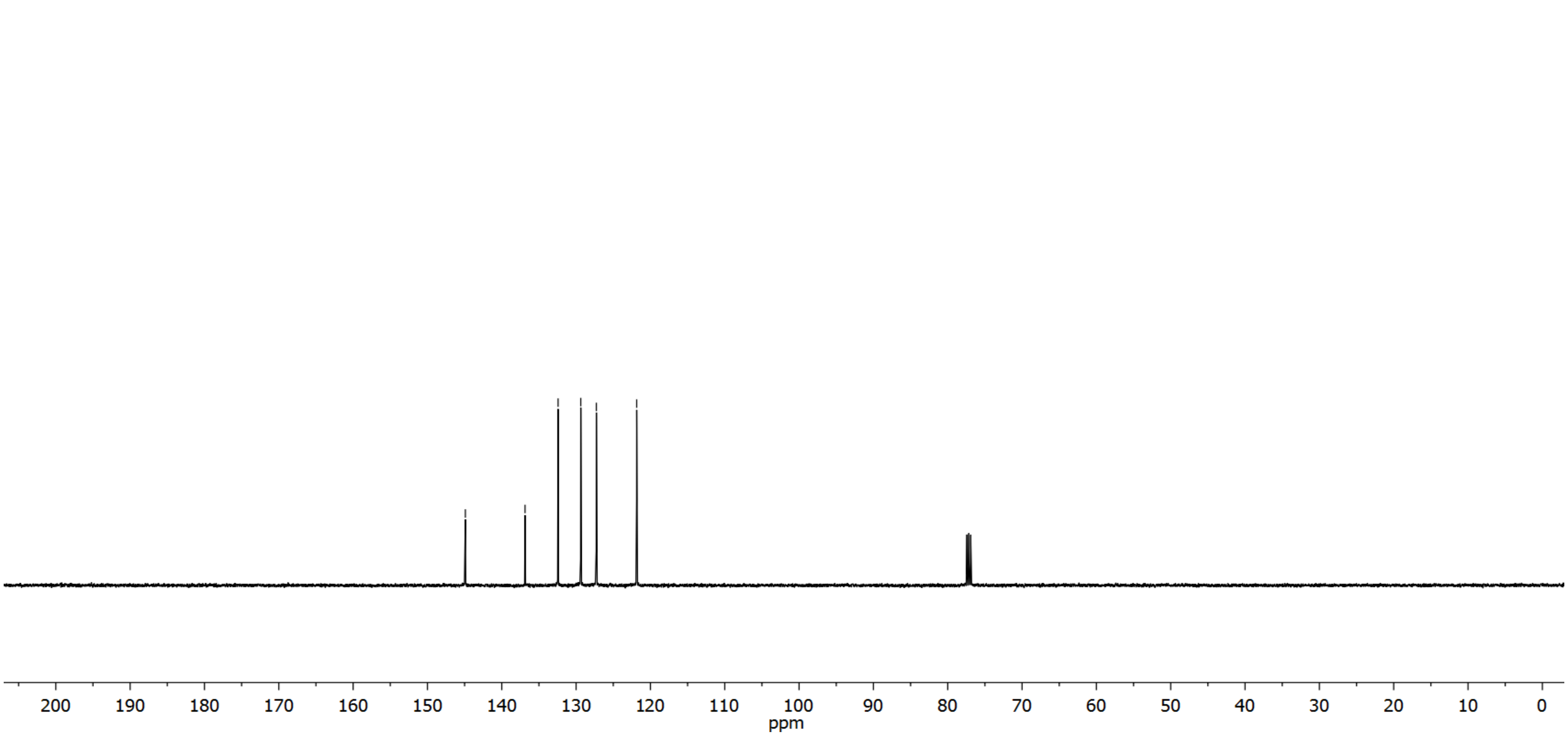

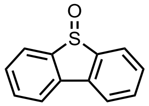

DBTO

^{1}H NMR of 3,7-di-*tert*-butyldibenzothiophene *S*-oxide (B-DBTO)

$CDCl_3$, 298 K

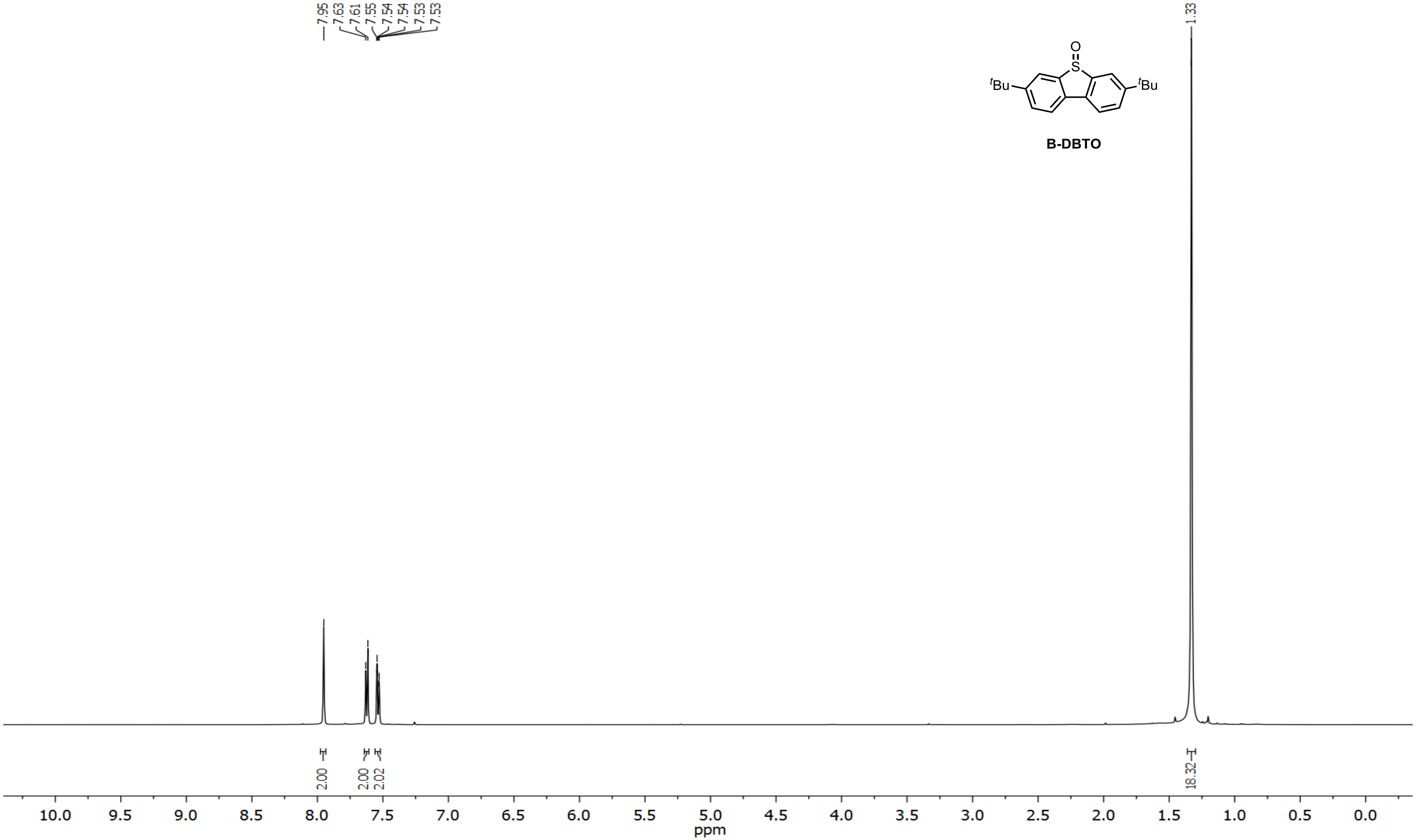

^{13}C NMR of 3,7-di-*tert*-butyldibenzothiophene *S*-oxide (B-DBTO)

$CDCl_3$, 298 K

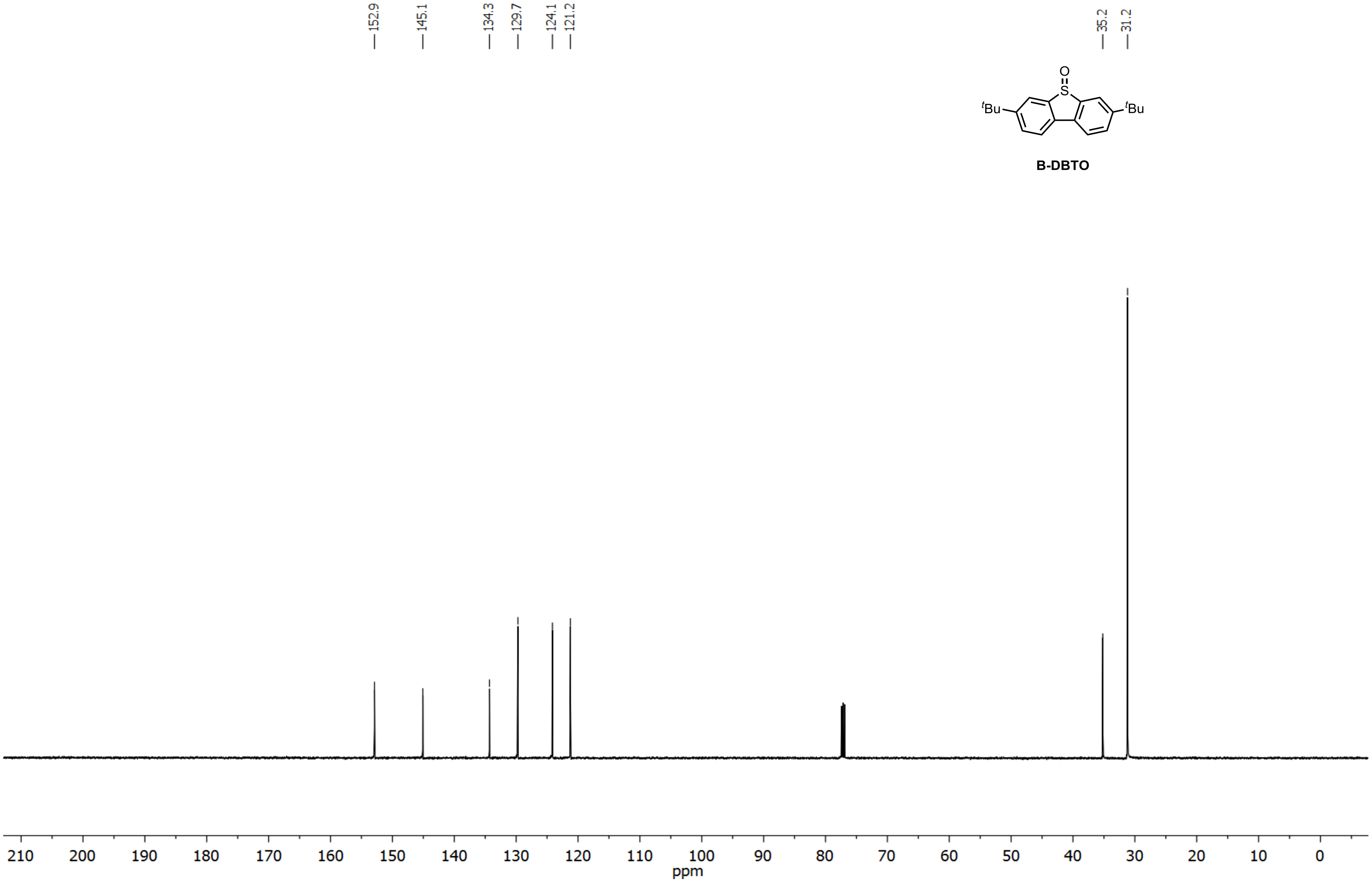

^{1}H NMR of 2,8-dimethoxydibenzothiophene *S*-oxide (M-DBTO)

$CDCl_3$, 298 K

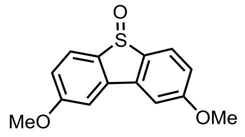

M-DBTO

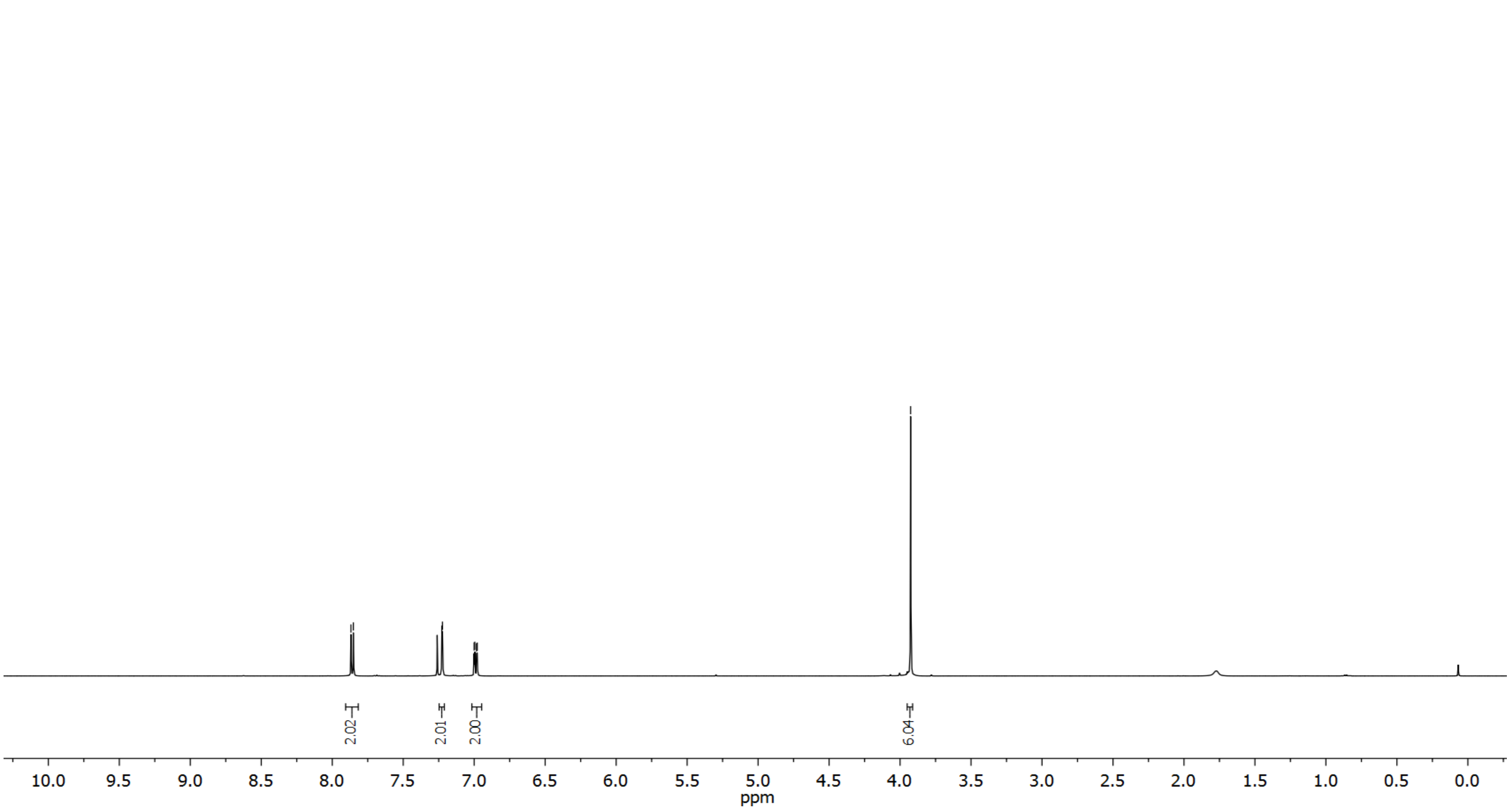

^{13}C NMR of 2,8-dimethoxydibenzothiophene *S*-oxide (M-DBTO)

$CDCl_3$, 298 K

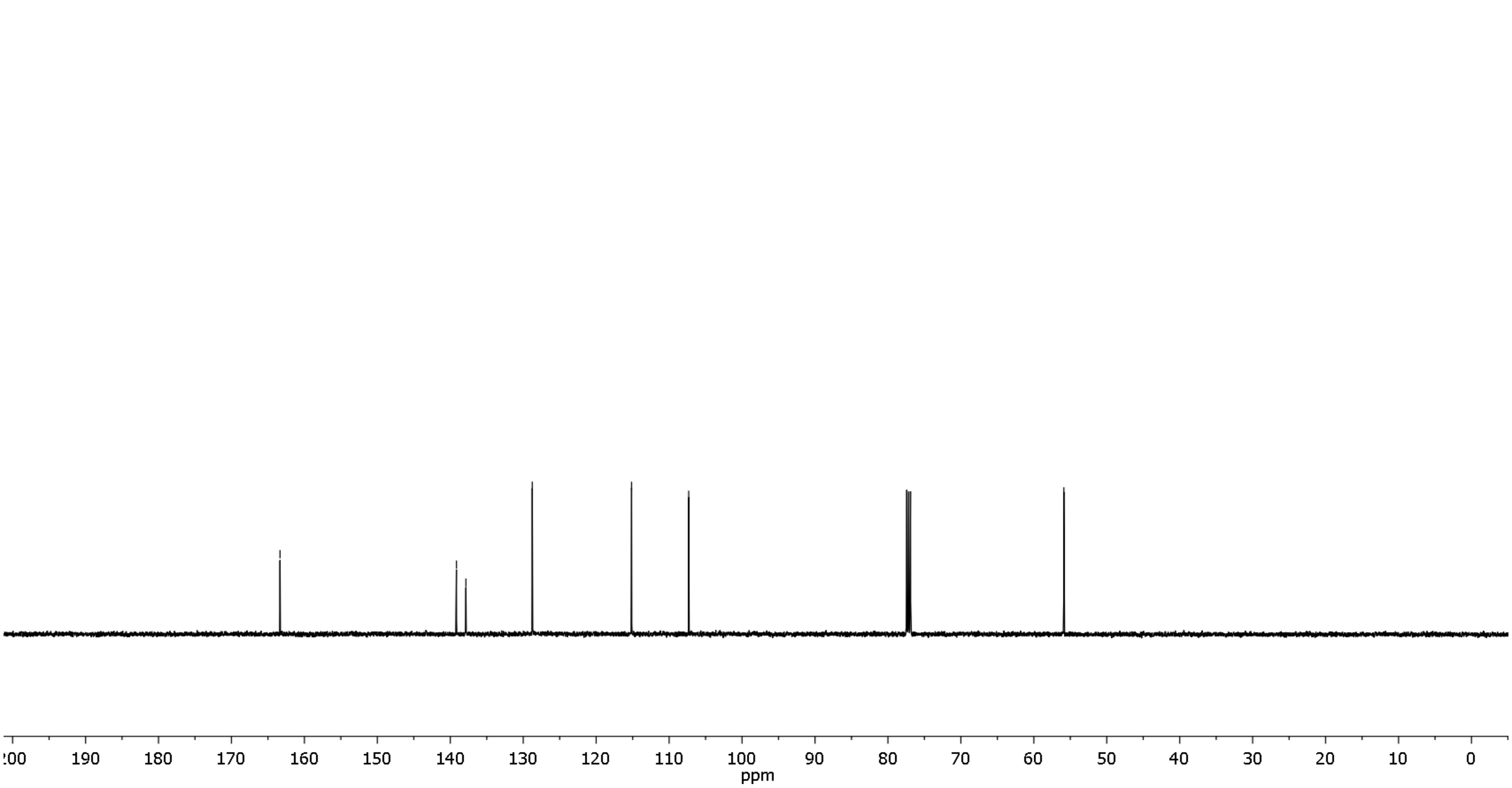

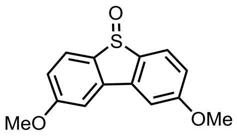

M-DBTO

^{1}H NMR of biphenyl-derived dibenzothiophenium salt S1

CD$_3$CN, 298 K

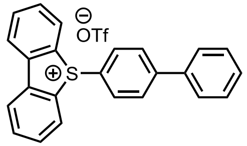

S1

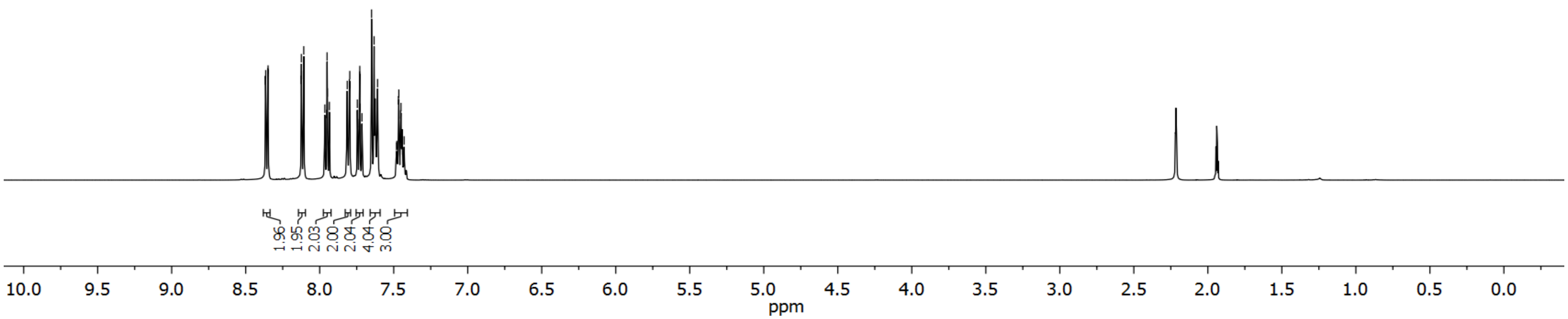

^{13}C NMR of biphenyl-derived dibenzothiophenium salt S1

CD_3CN, 298 K

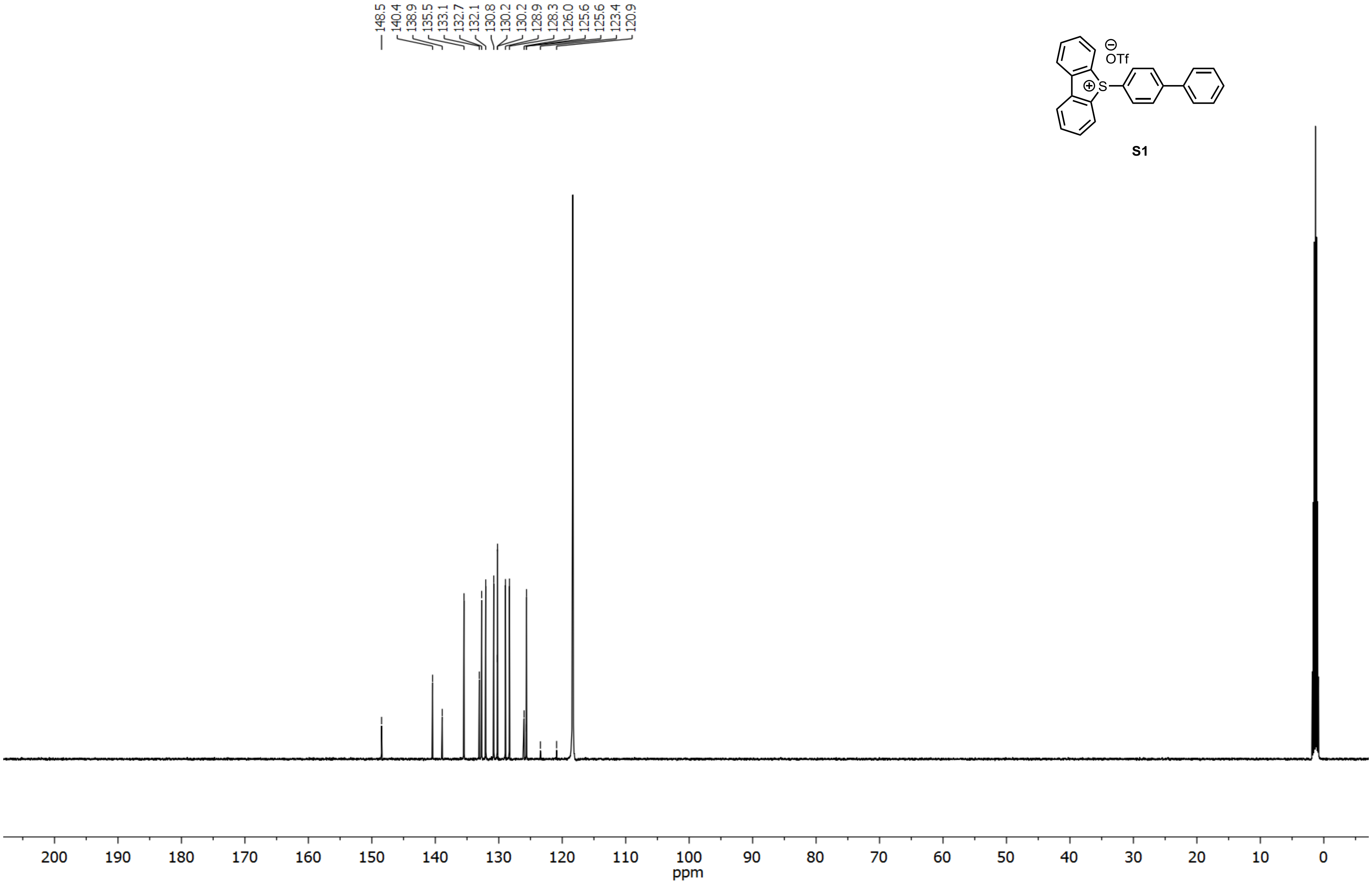

^{19}F NMR of biphenyl-derived dibenzothiophenium salt S1

CD_3CN, 298 K

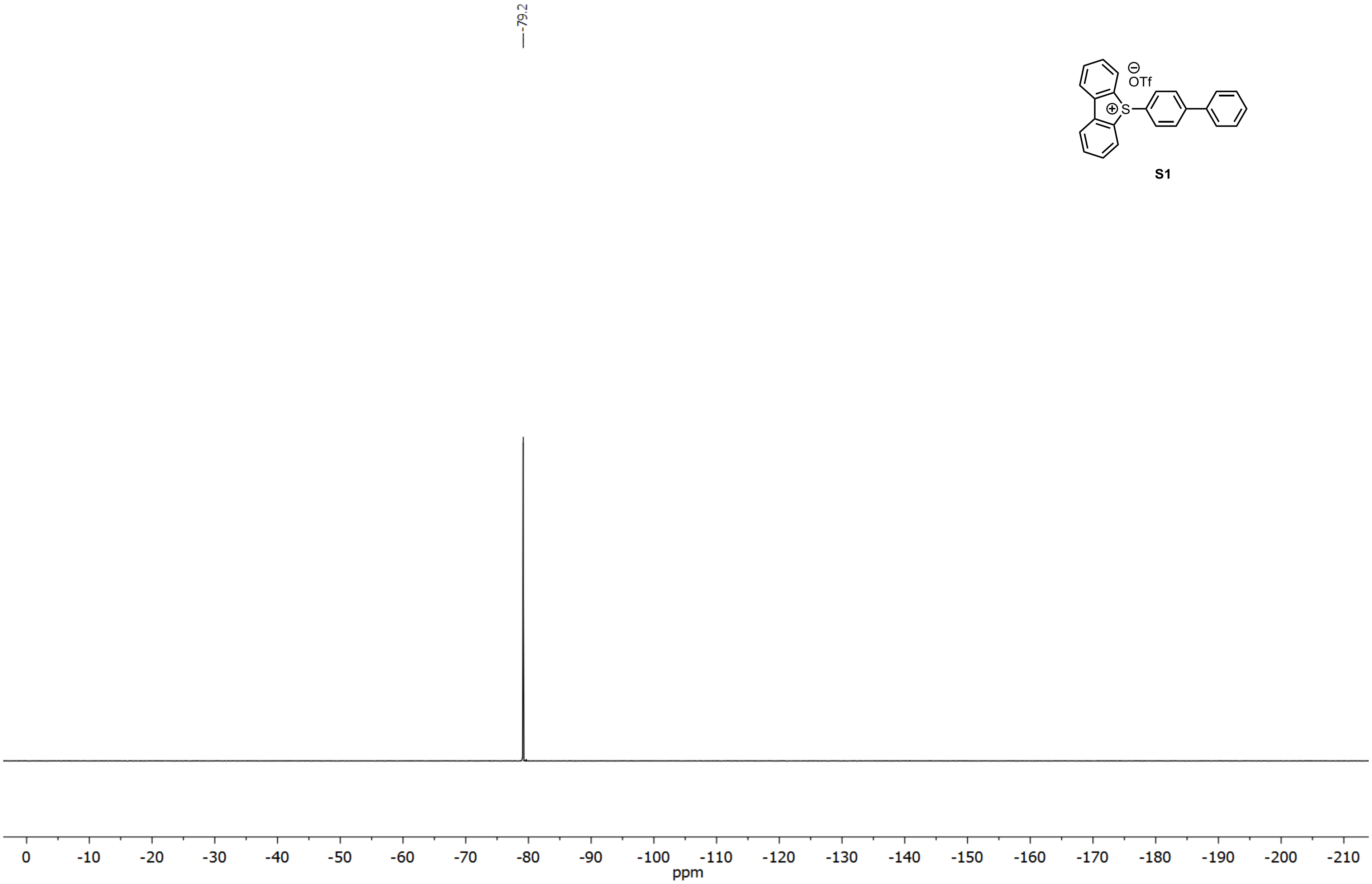

S1

^{1}H NMR of 1,2-dichlorobenzene-derived dibenzothiophenium salt S2

CD_3CN, 298 K

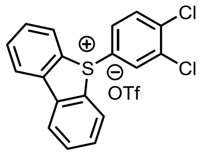

S2

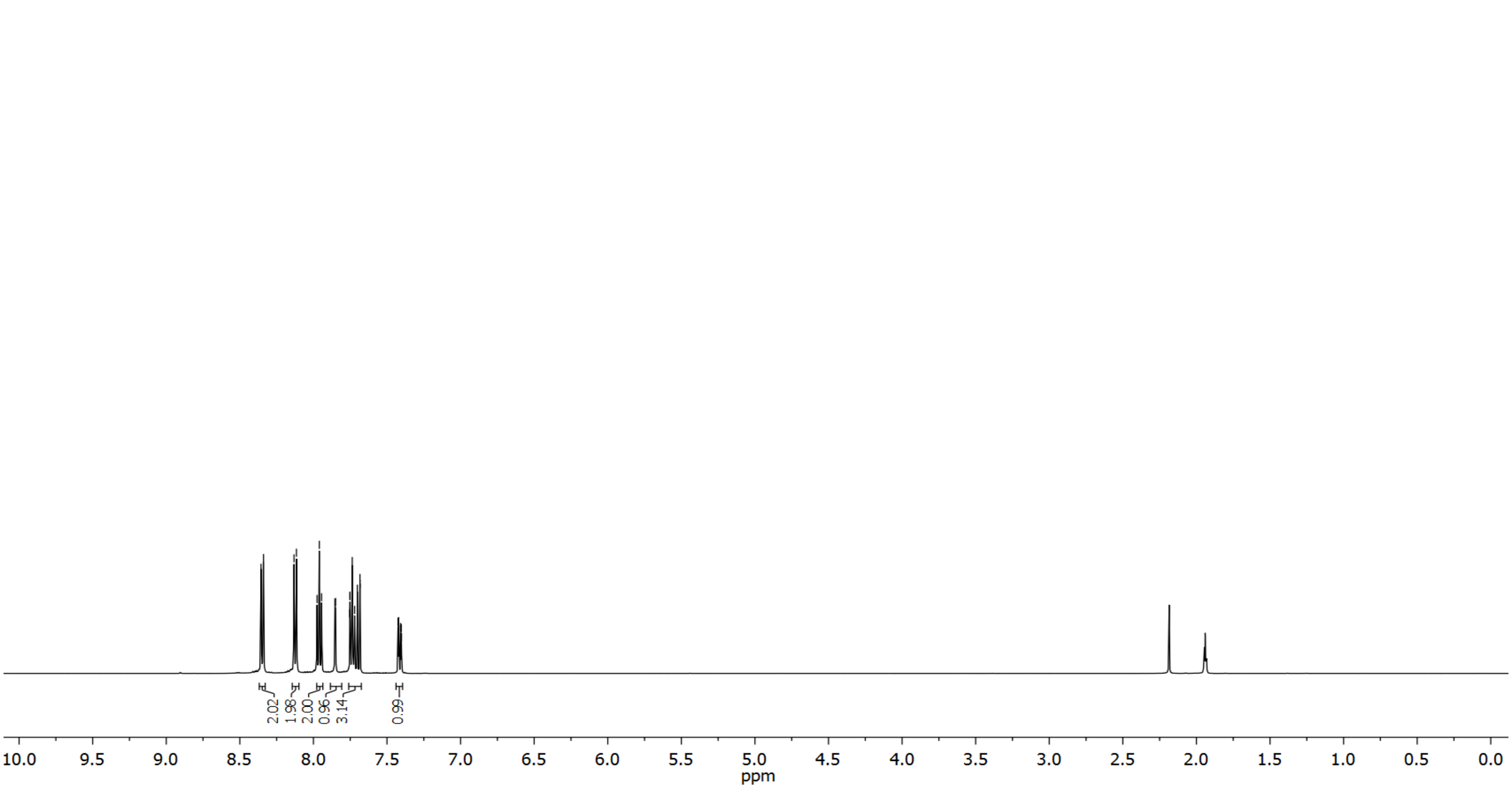

^{13}C NMR of 1,2-dichlorobenzene-derived dibenzothiophenium salt S2

CD_3CN, 298 K

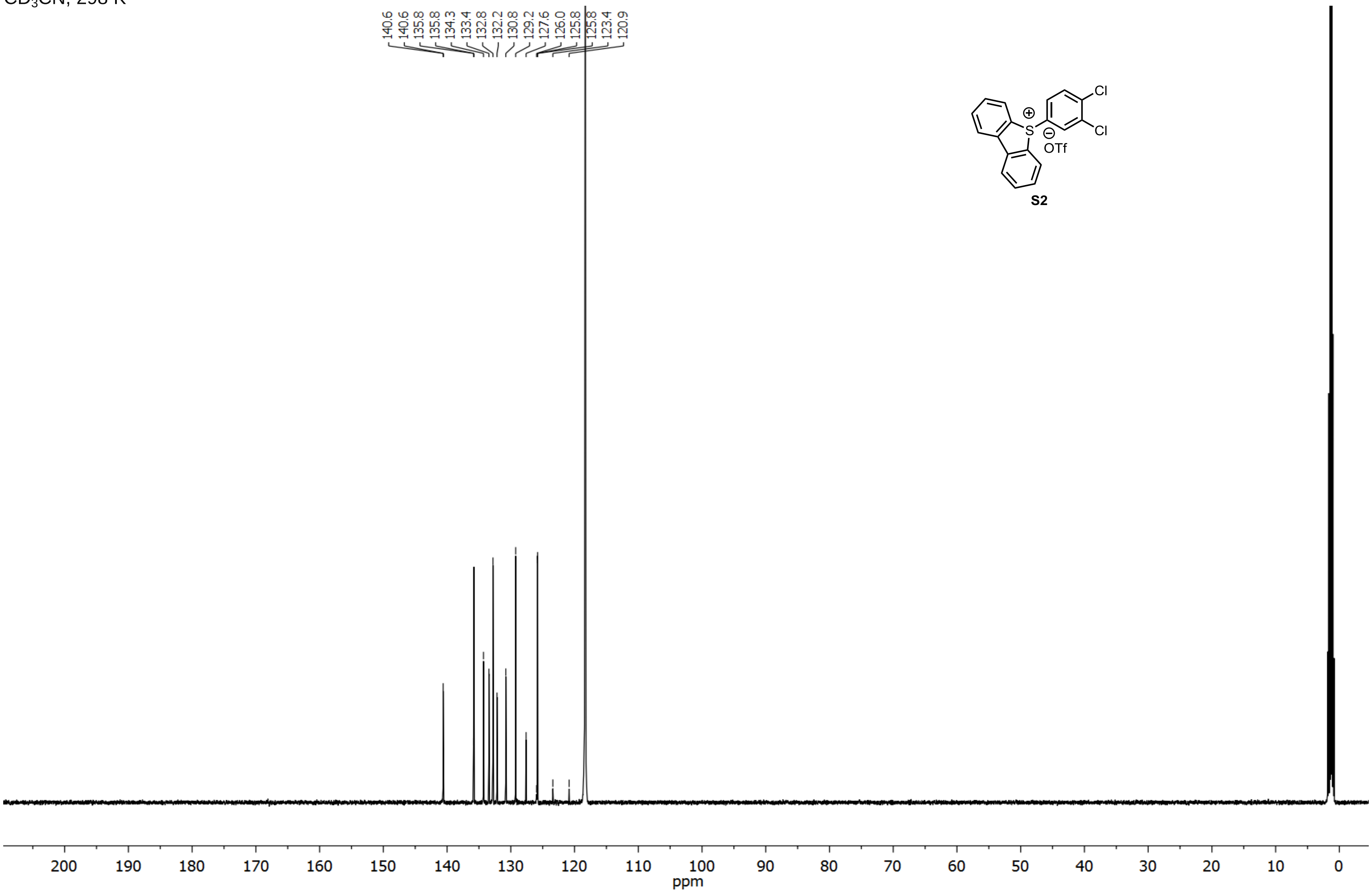

^{19}F NMR of 1,2-dichlorobenzene-derived dibenzothiophenium salt S2

CD_3CN, 298 K

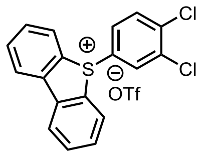

S2

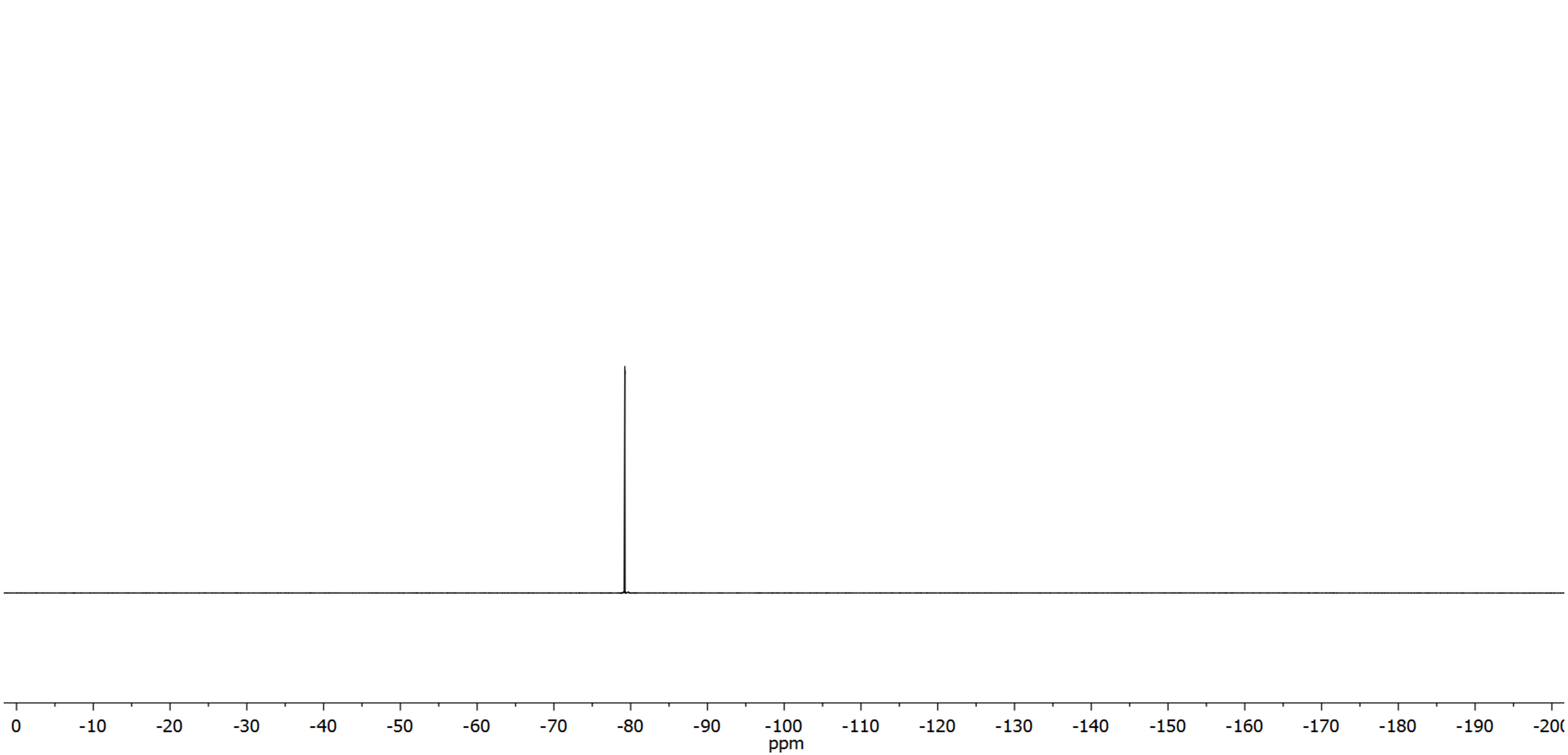

^{1}H NMR of 2-fluoro-6-phenoxybenzonitrile-derived dibenzothiophenium salt S3

CD_3CN, 298 K

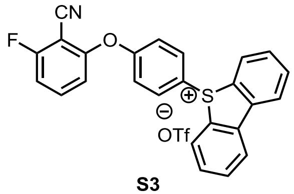

S3

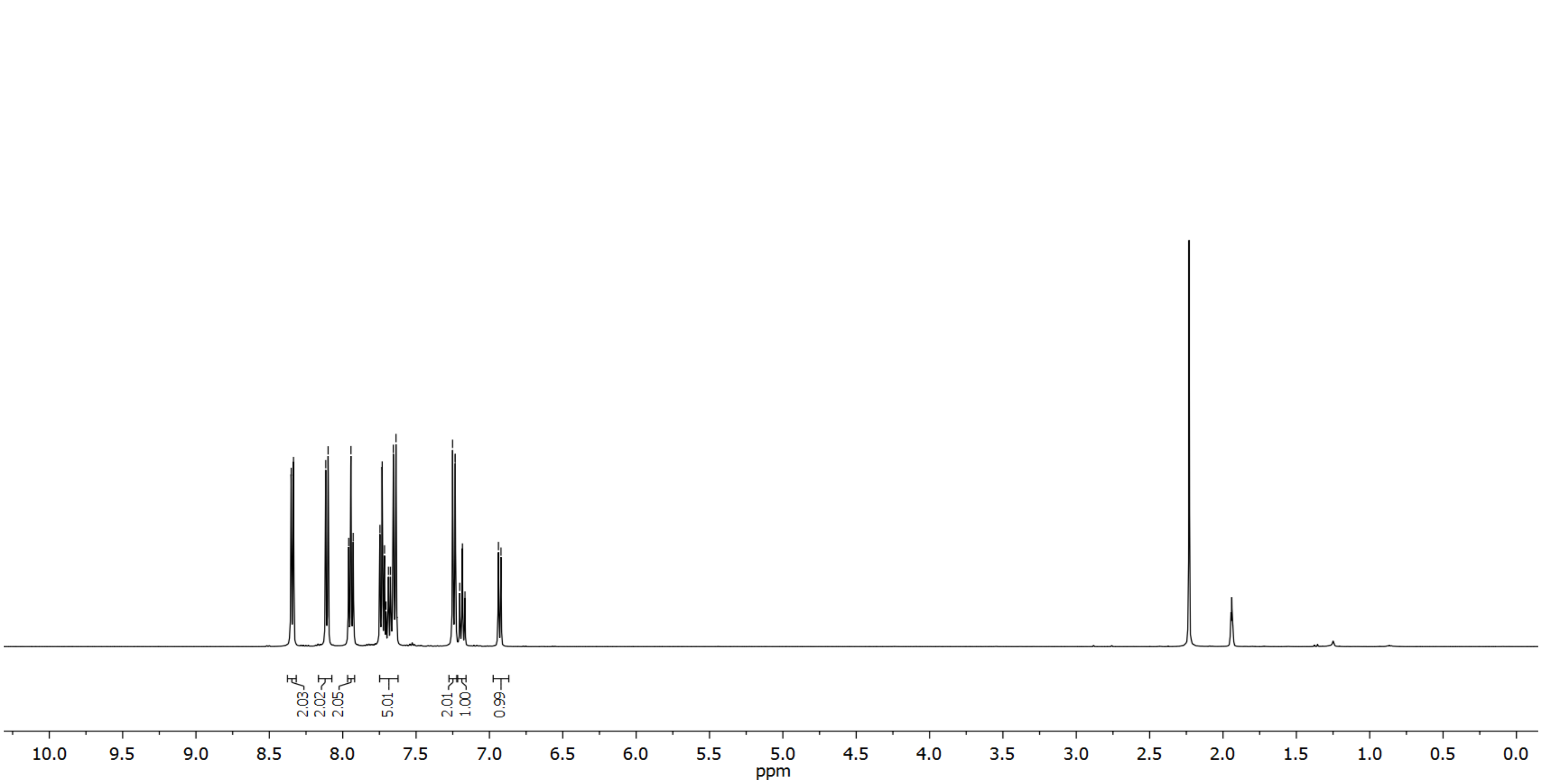

^{13}C NMR of 2-fluoro-6-phenoxybenzonitrile-derived dibenzothiophenium salt S3

CD_3CN, 298 K

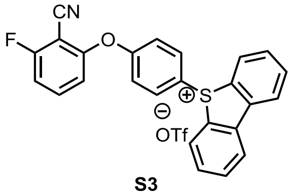

S3

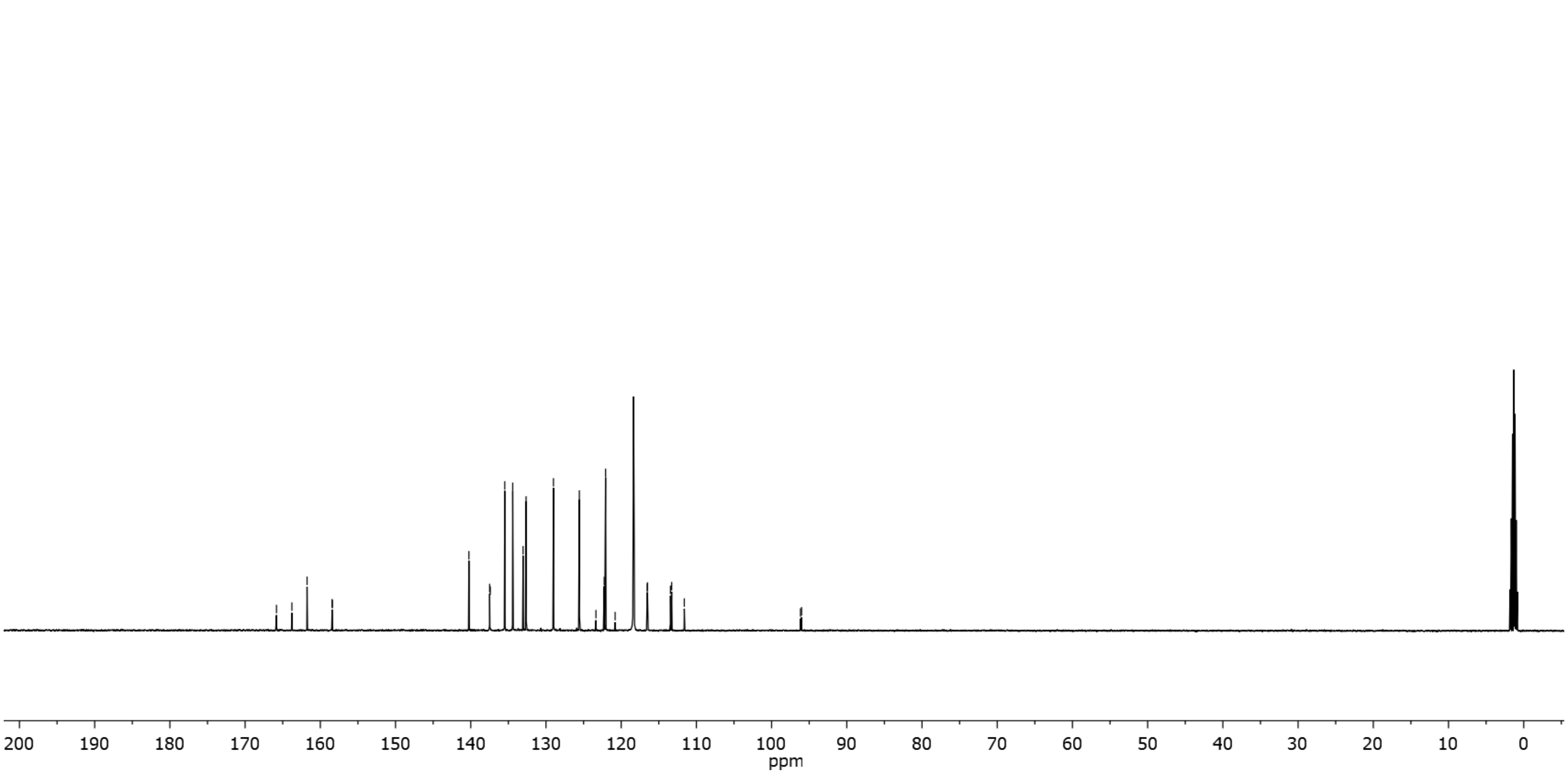

^{19}F NMR of 2-fluoro-6-phenoxybenzonitrile-derived dibenzothiophenium salt S3

CD_3CN, 298 K

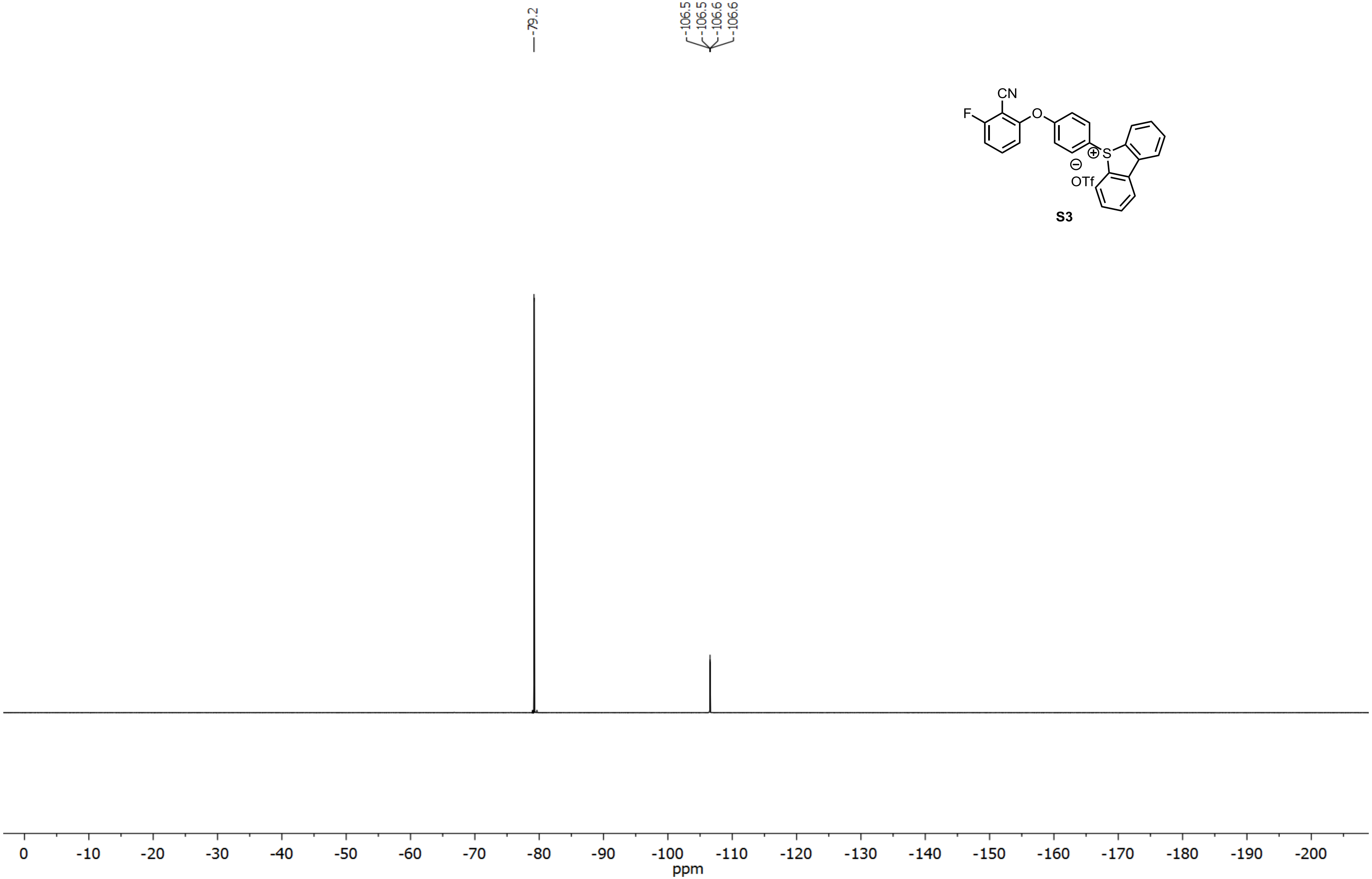

S3

^{1}H NMR of diflunisal derivative-derived dibenzothiophenium salt S4

CD$_3$CN, 298 K

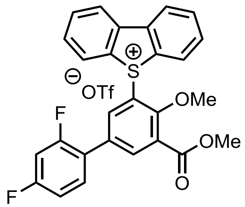

S4

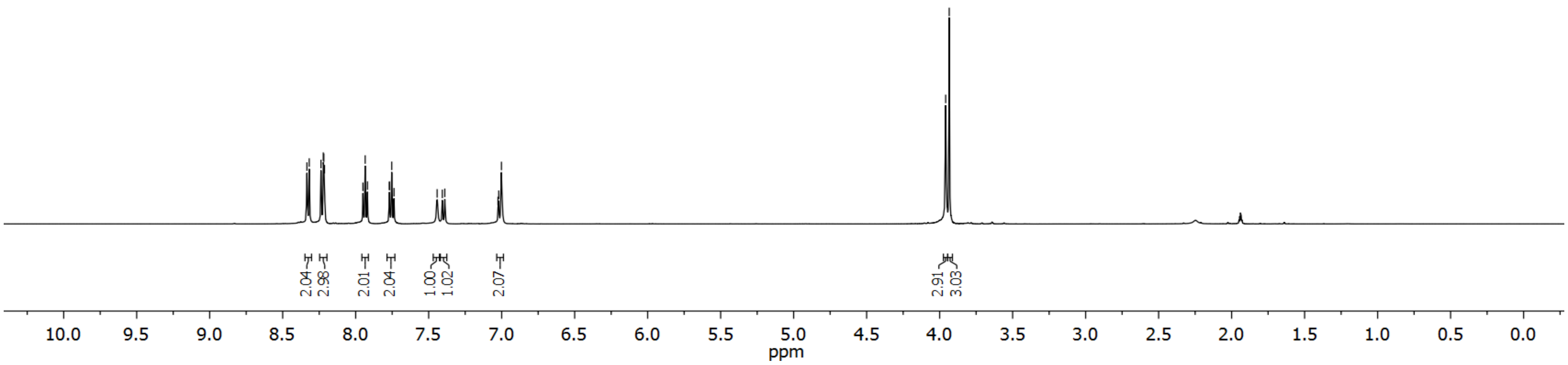

^{13}C NMR of diflunisal derivative-derived dibenzothiophenium salt S4

CD$_3$CN, 298 K

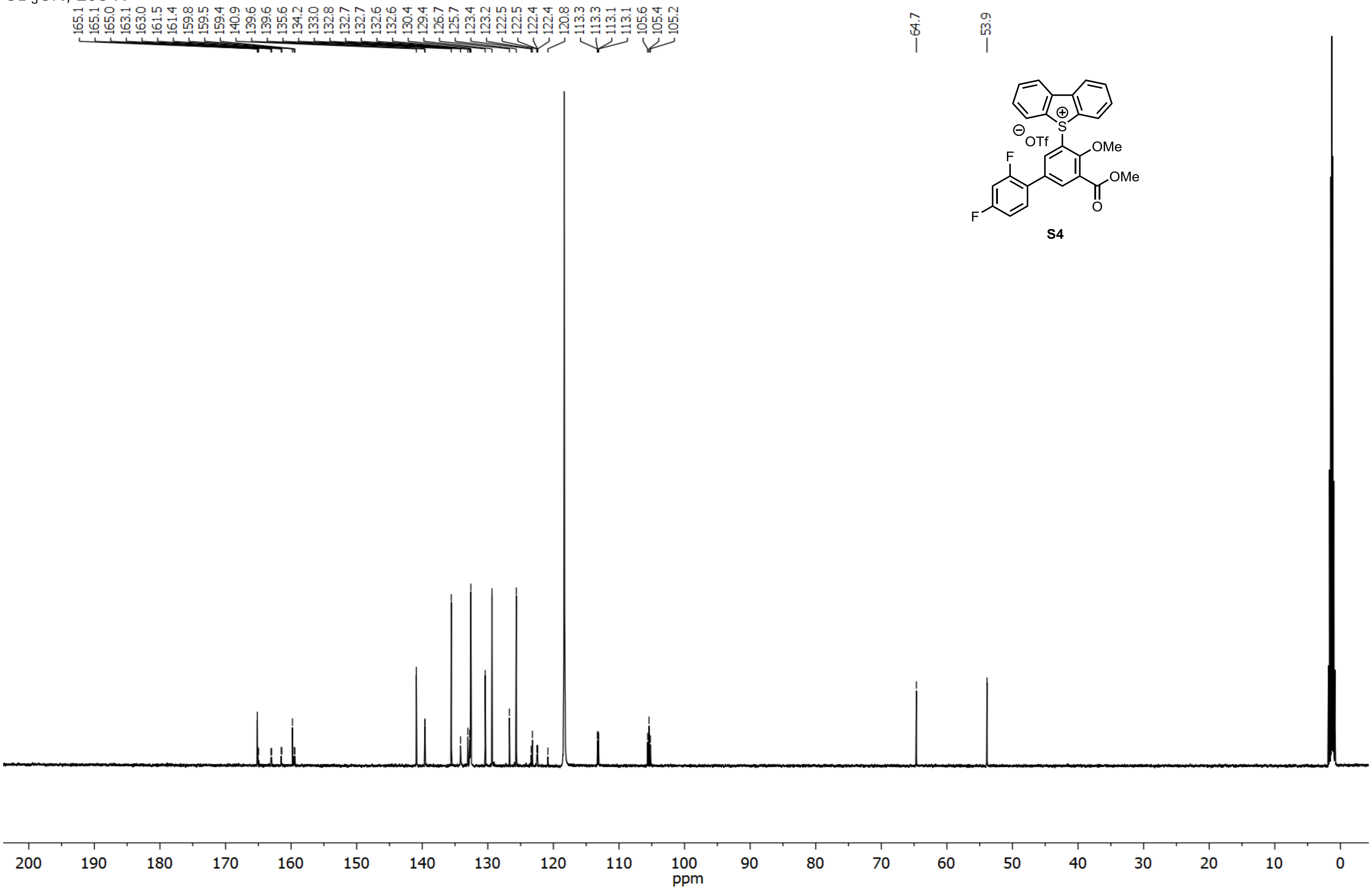

^{19}F NMR of diflunisal derivative-derived dibenzothiophenium salt S4

CD_3CN, 298 K

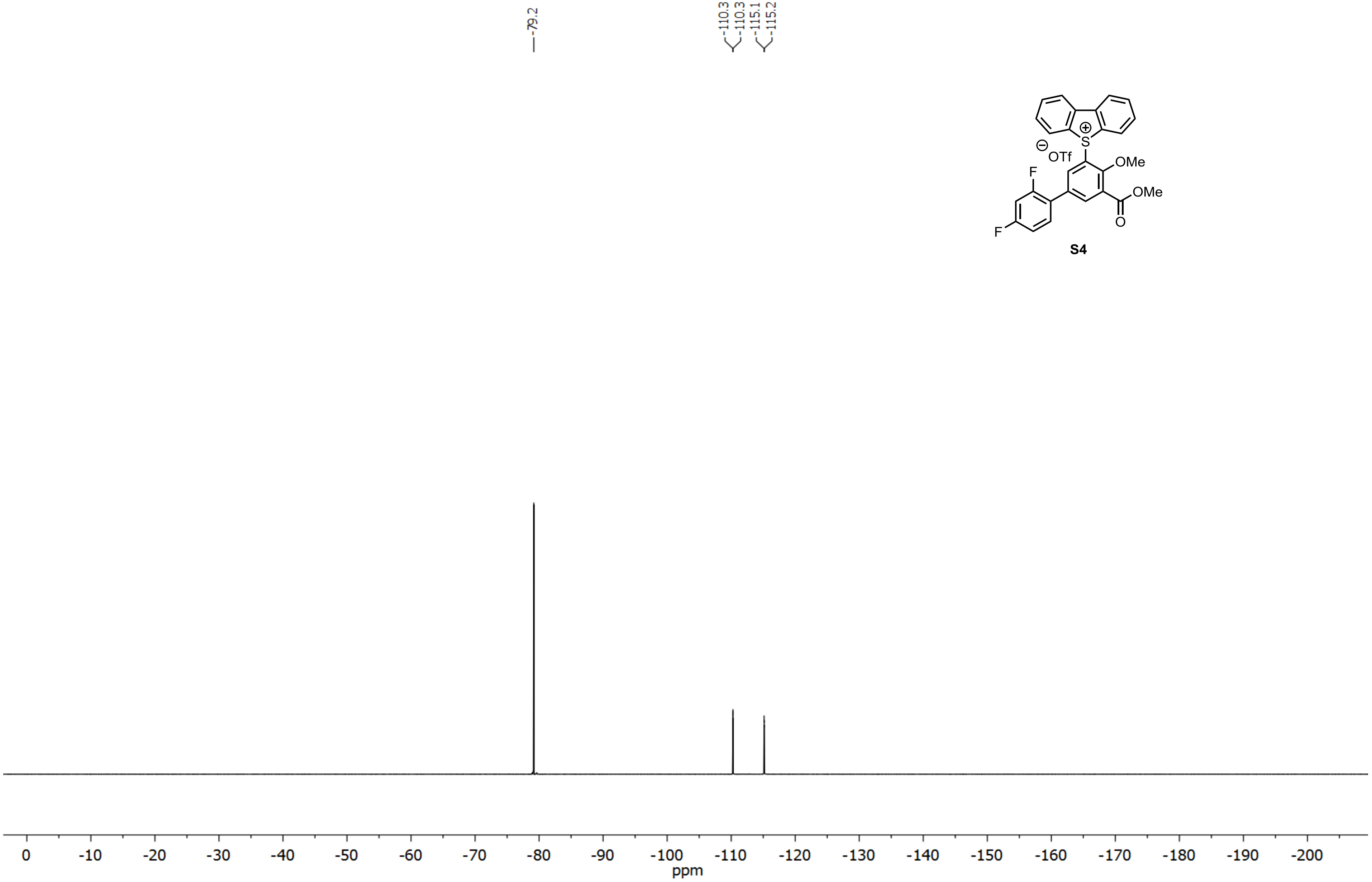

^{1}H NMR of *rac*-propafenone derivative-derived 3,7-di-*tert*-butyldibenzothiophenium salt S5

CD_3CN, 298 K

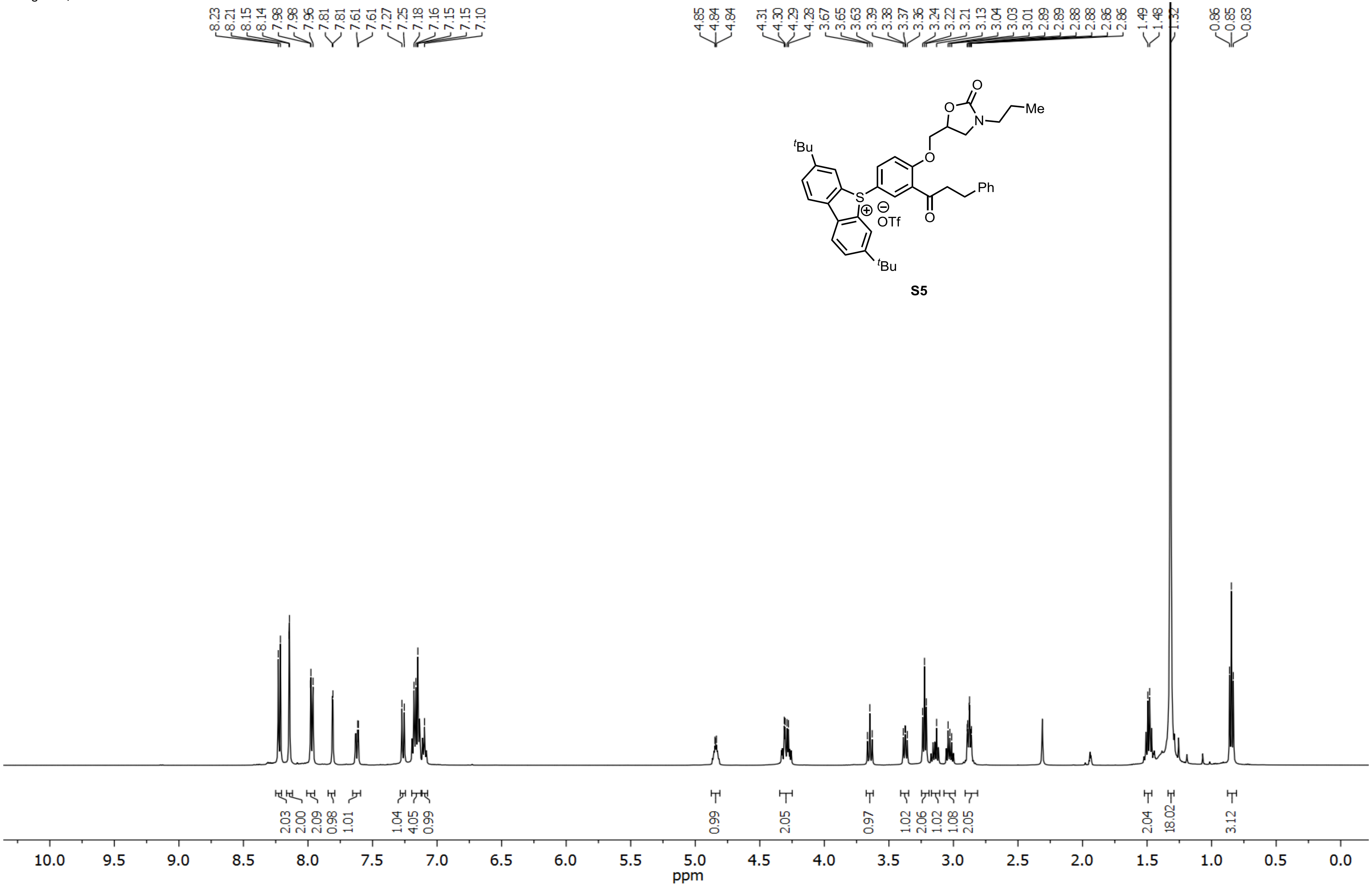

^{13}C NMR of *rac*-propafenone derivative-derived 3,7-di-*tert*-butyldibenzothiophenium salt S5

CD_3CN, 298 K

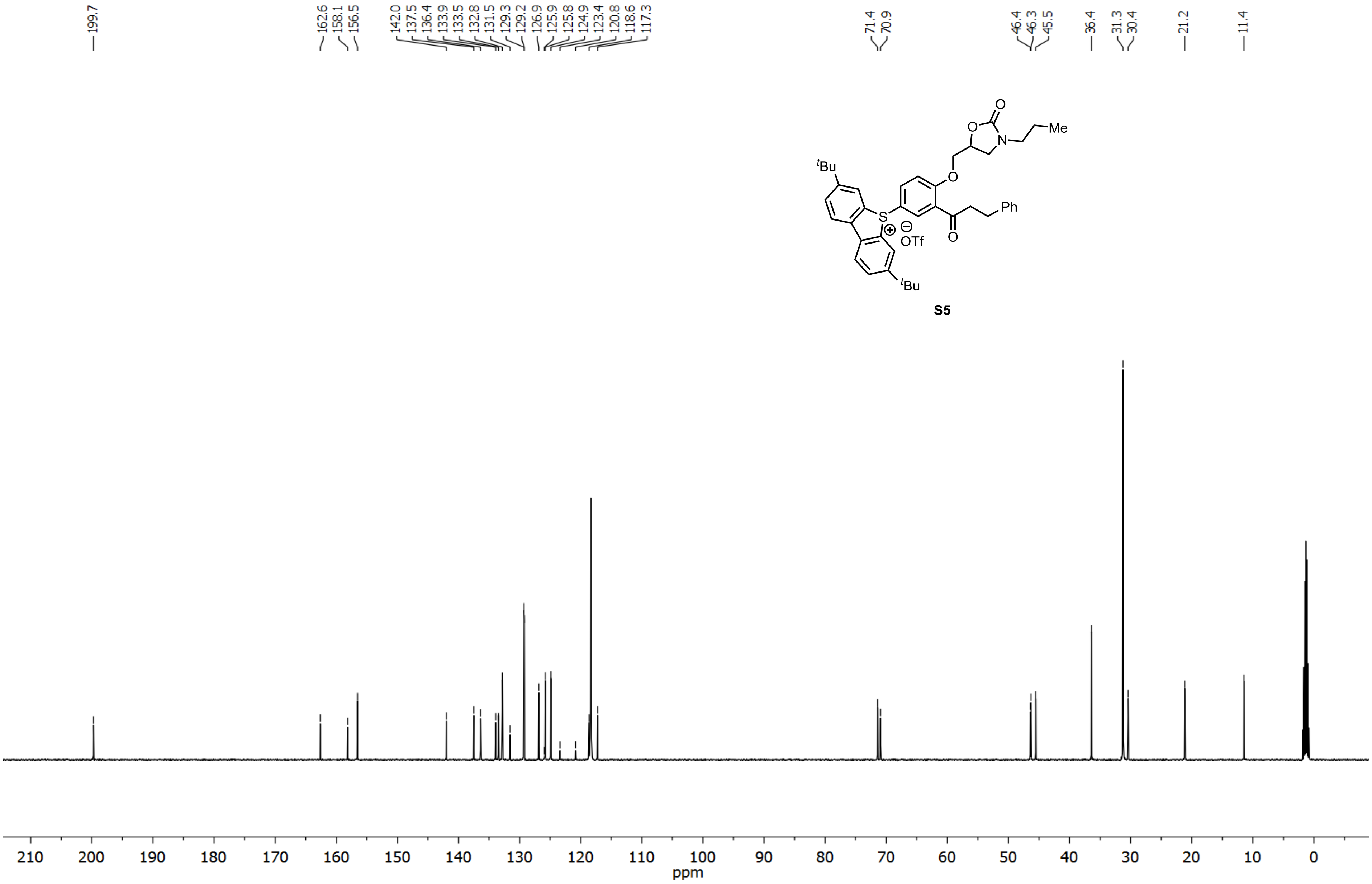

^{19}F NMR of *rac*-propafenone derivative-derived 3,7-di-*tert*-butyldibenzothiophenium salt S5

CD_3CN, 298 K

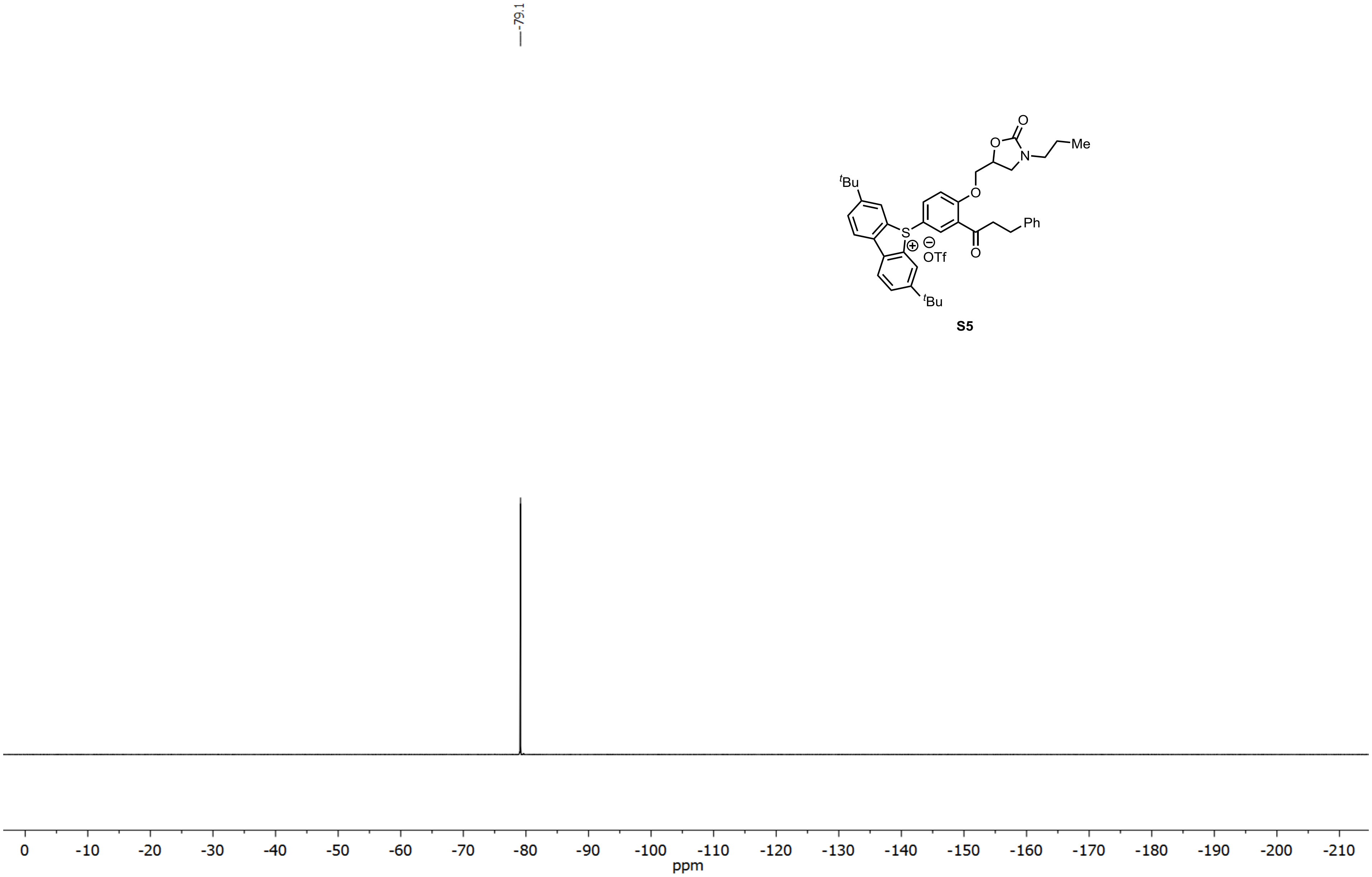

S5

^{1}H NMR of toluene-derived dibenzothiophenium salt S6

CD$_3$CN, 298 K

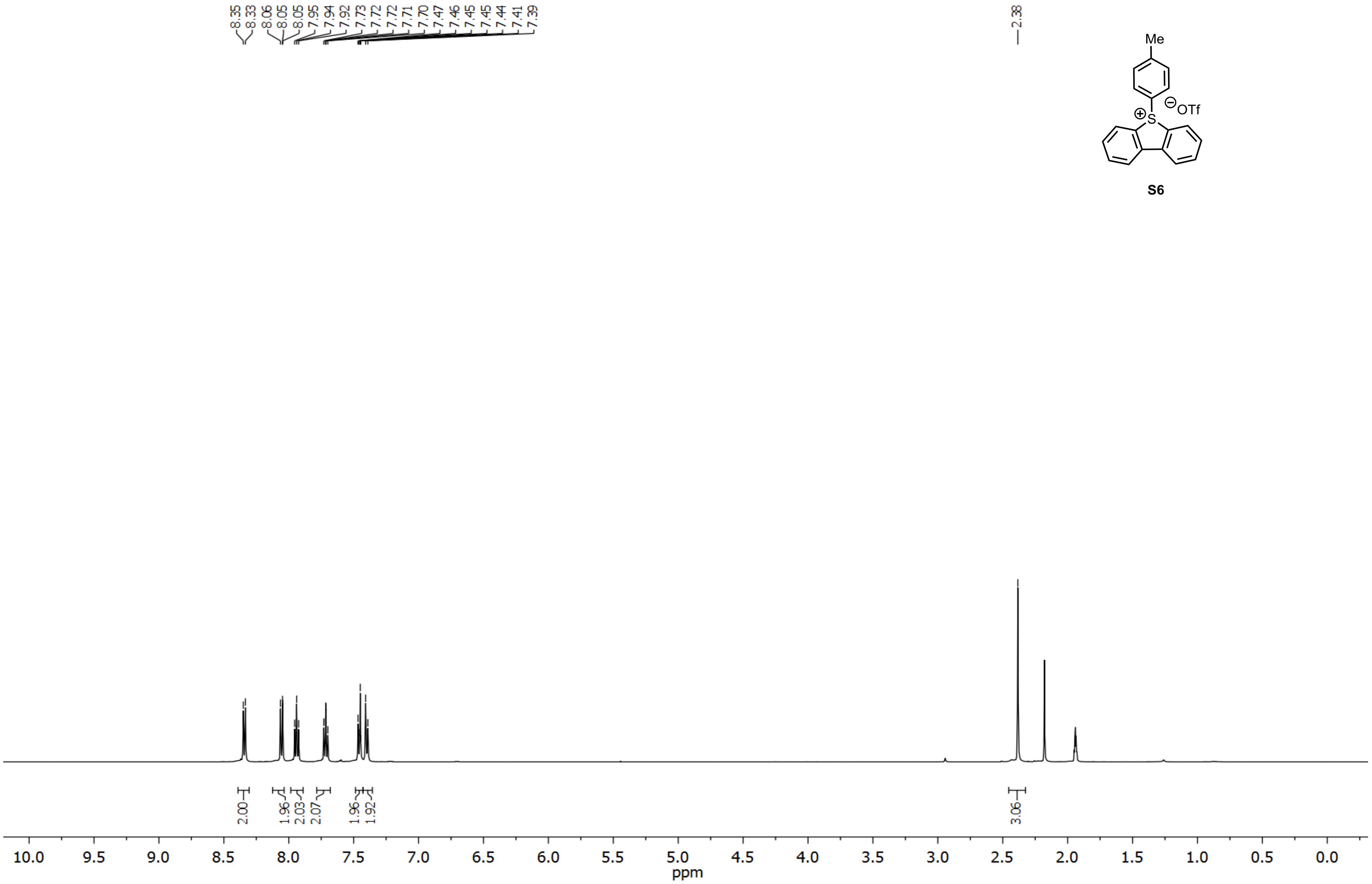

S6

^{13}C NMR of toluene-derived dibenzothiophenium salt S6

CD_3CN, 298 K

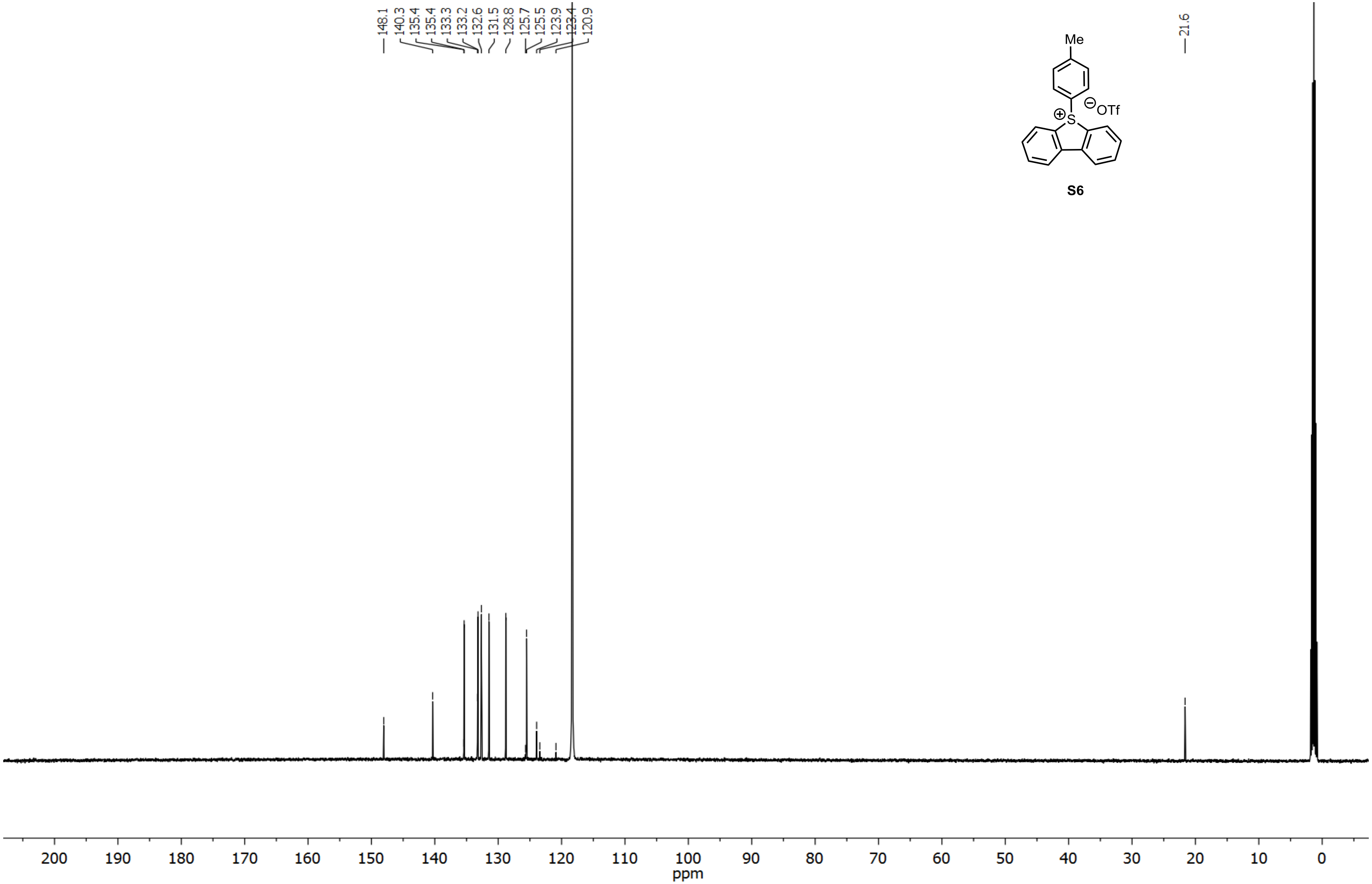

^{19}F NMR of toluene-derived dibenzothiophenium salt S6

CD_3CN, 298 K

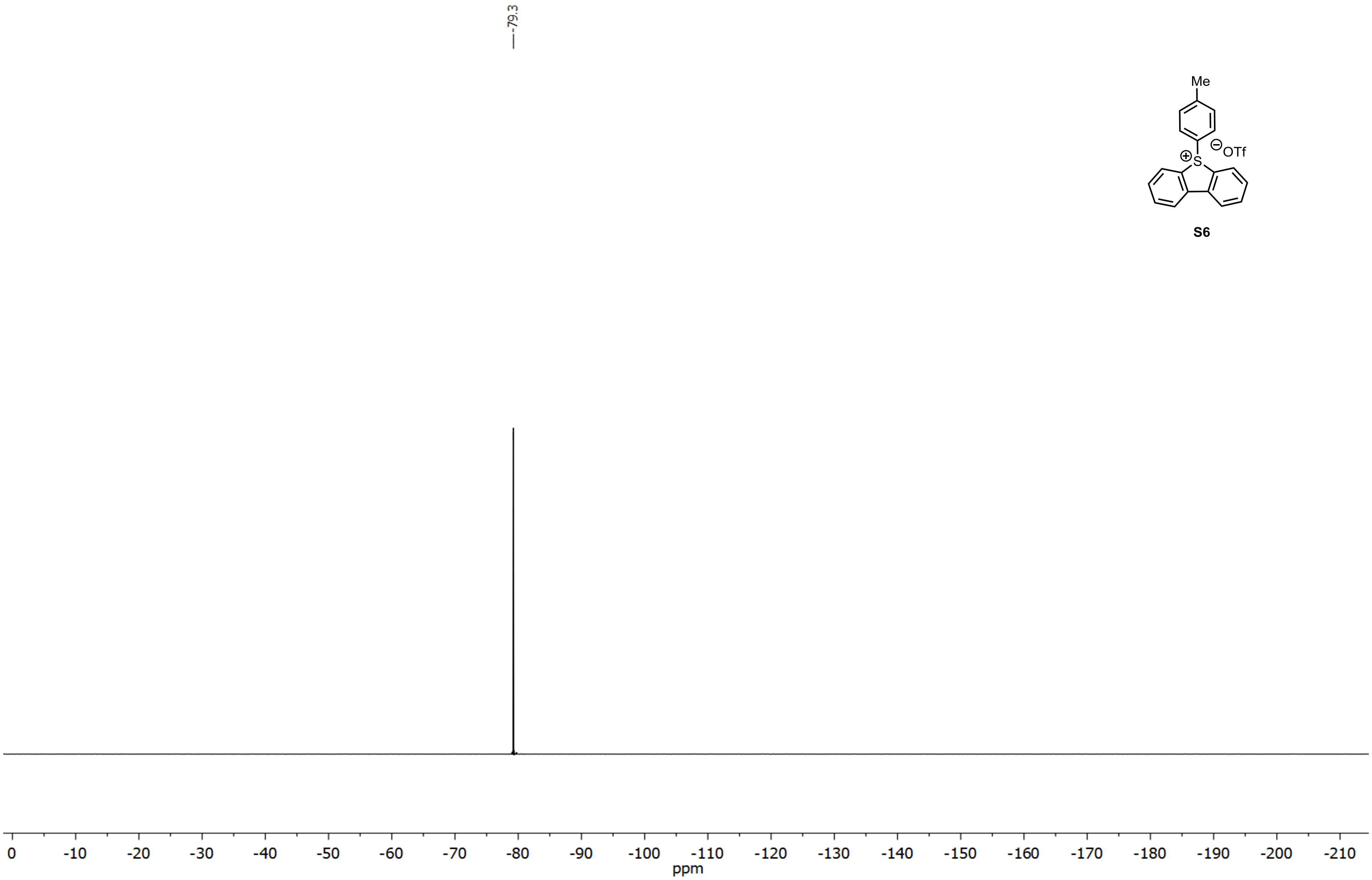

S6

^{1}H NMR of *o*-anisaldehyde-derived 3,7-di-*tert*-butyldibenzothiophenium salt S7

CD_3CN, 298 K

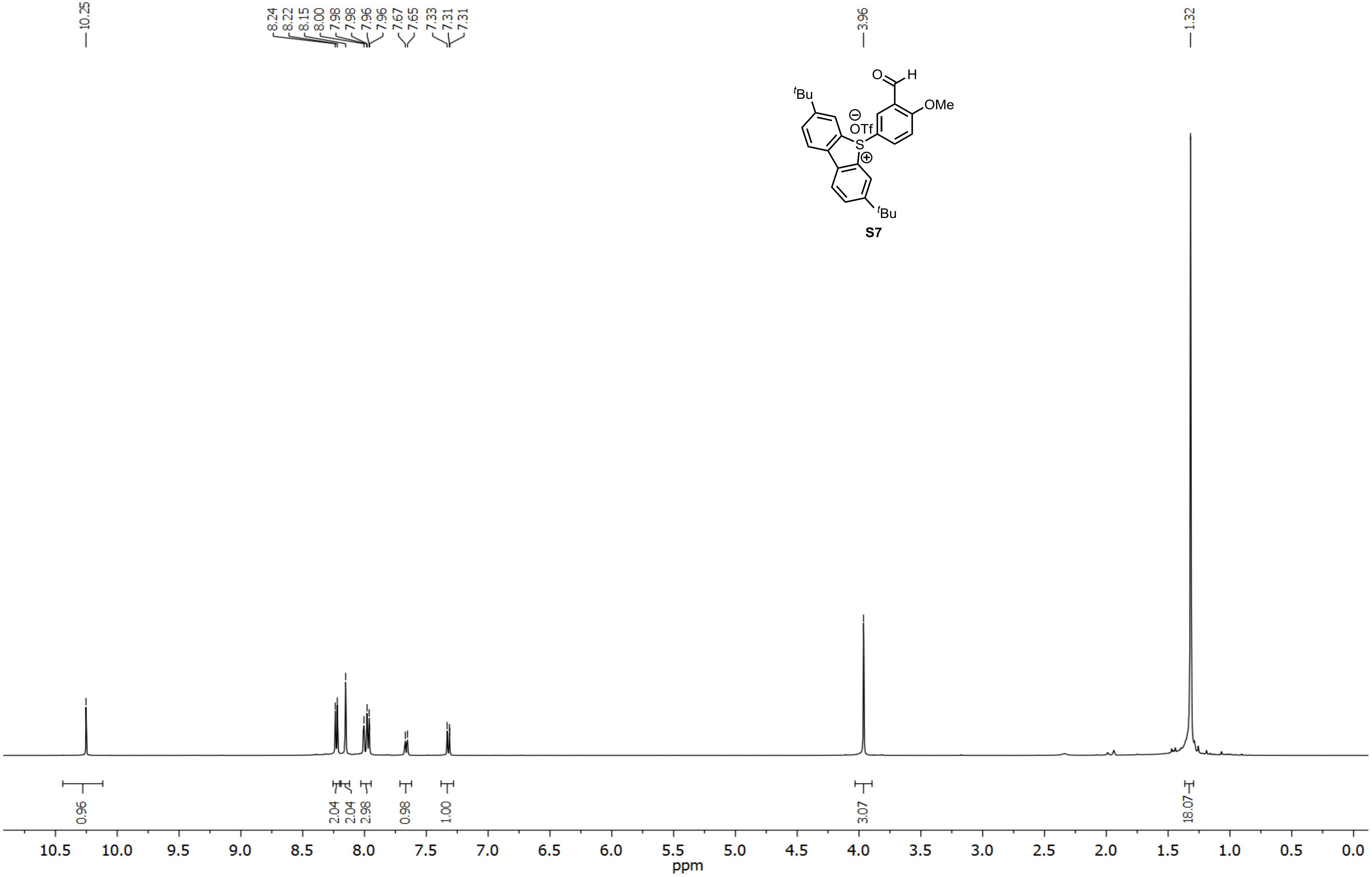

^{13}C NMR of *o*-anisaldehyde-derived 3,7-di-*tert*-butyldibenzothiophenium salt S7

CD_3CN, 298 K

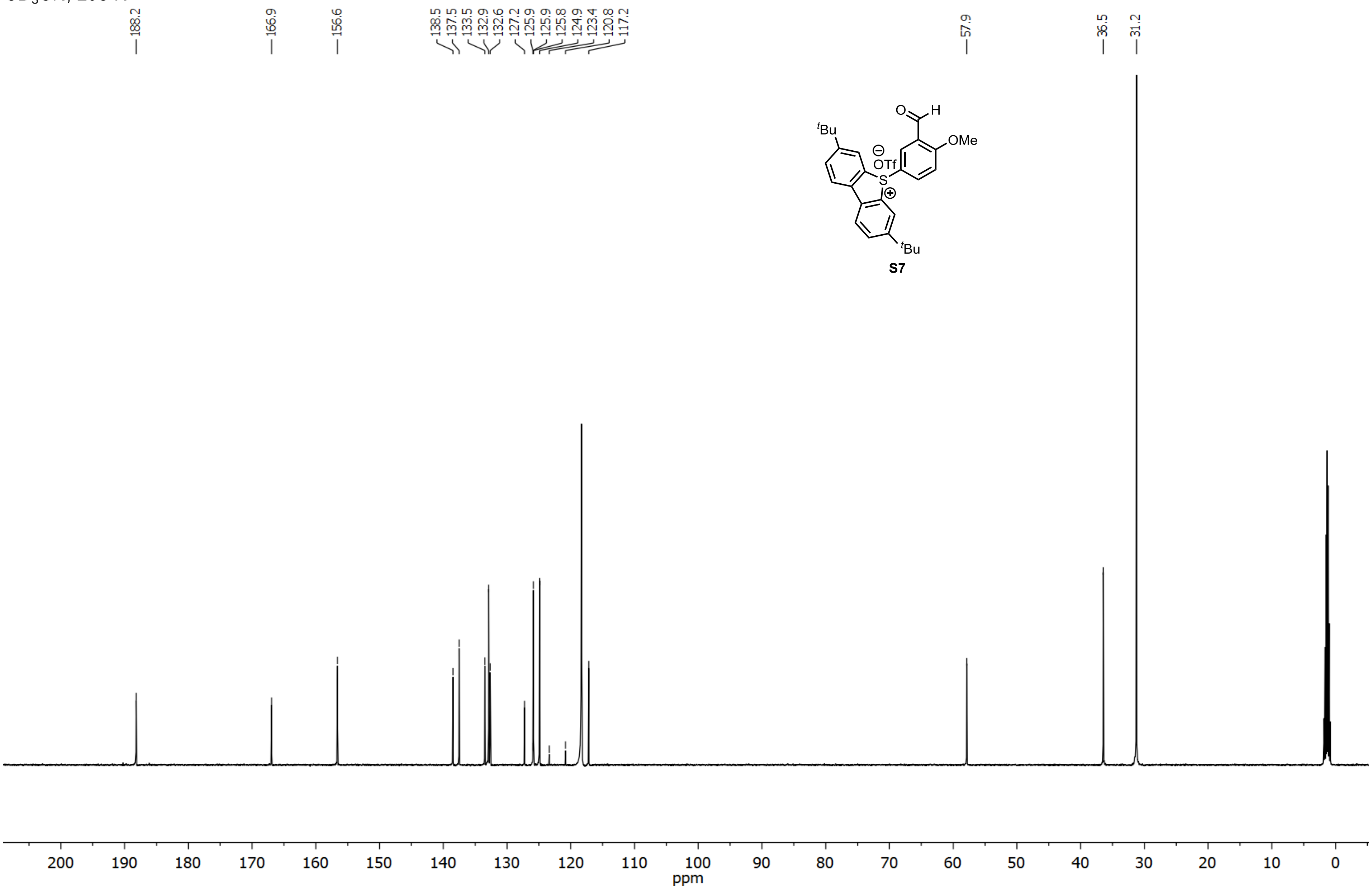

^{19}F NMR of *o*-anisaldehyde-derived 3,7-di-*tert*-butyldibenzothiophenium salt S7

CD_3CN, 298 K

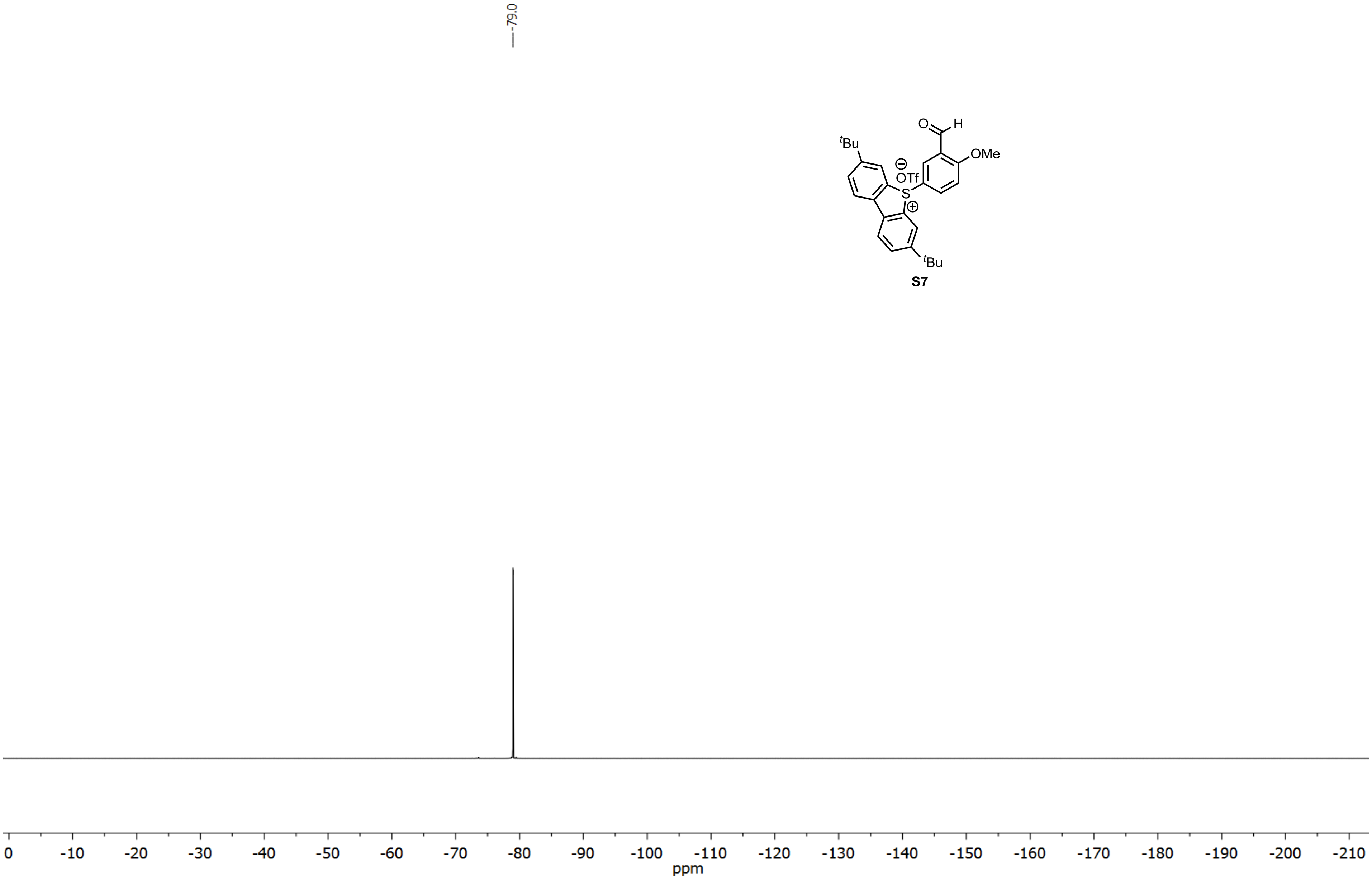

^{1}H NMR of (*R*)-(−)-4-benzyl-3-propionyl-2-oxazolidinone-derived dibenzothiophenium salt S8

CD_3CN, 298 K

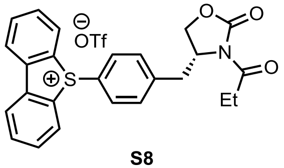

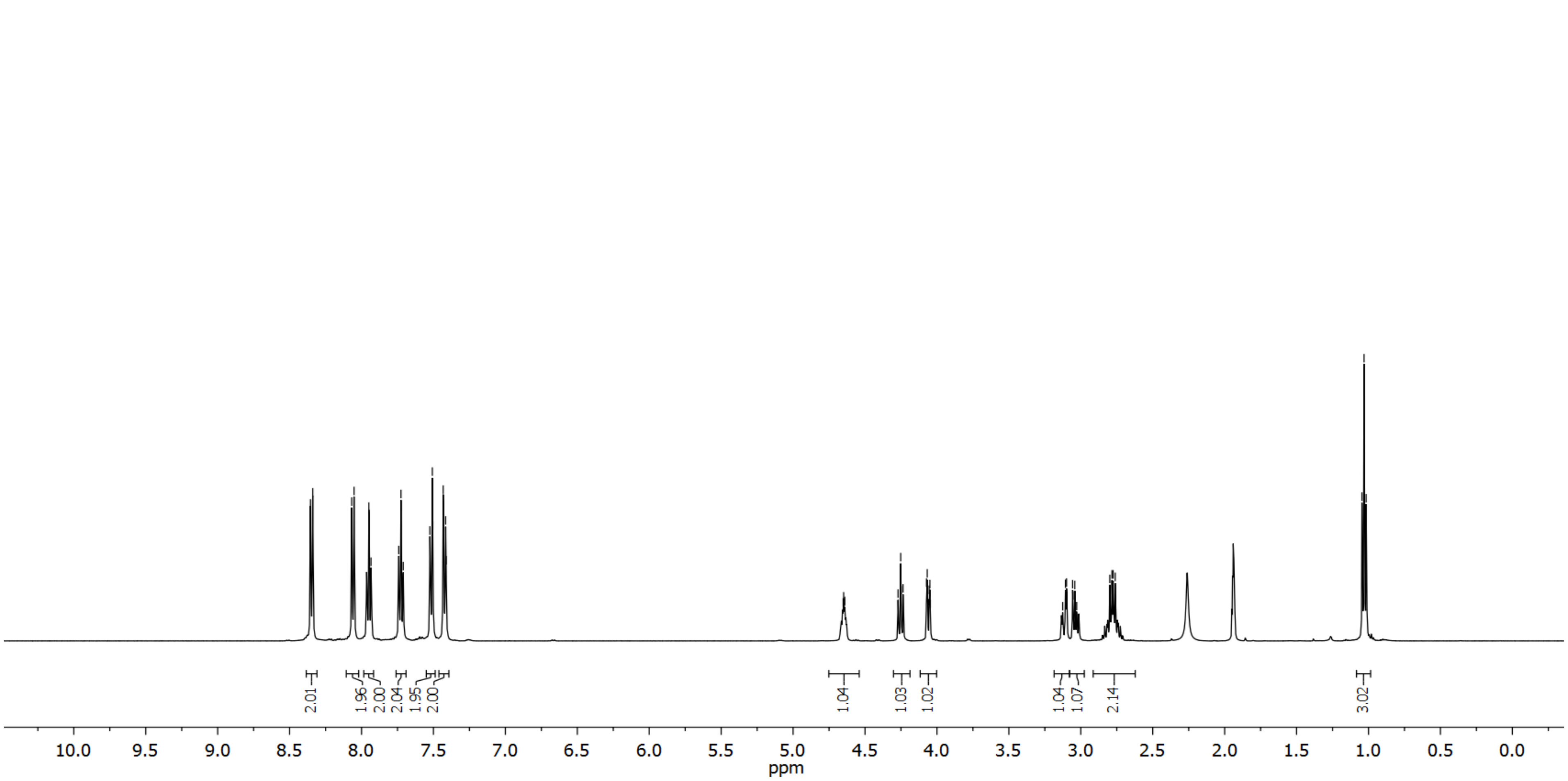

^{13}C NMR of (*R*)-(−)-4-benzyl-3-propionyl-2-oxazolidinone-derived dibenzothiophenium salt S8

CD_3CN, 298 K

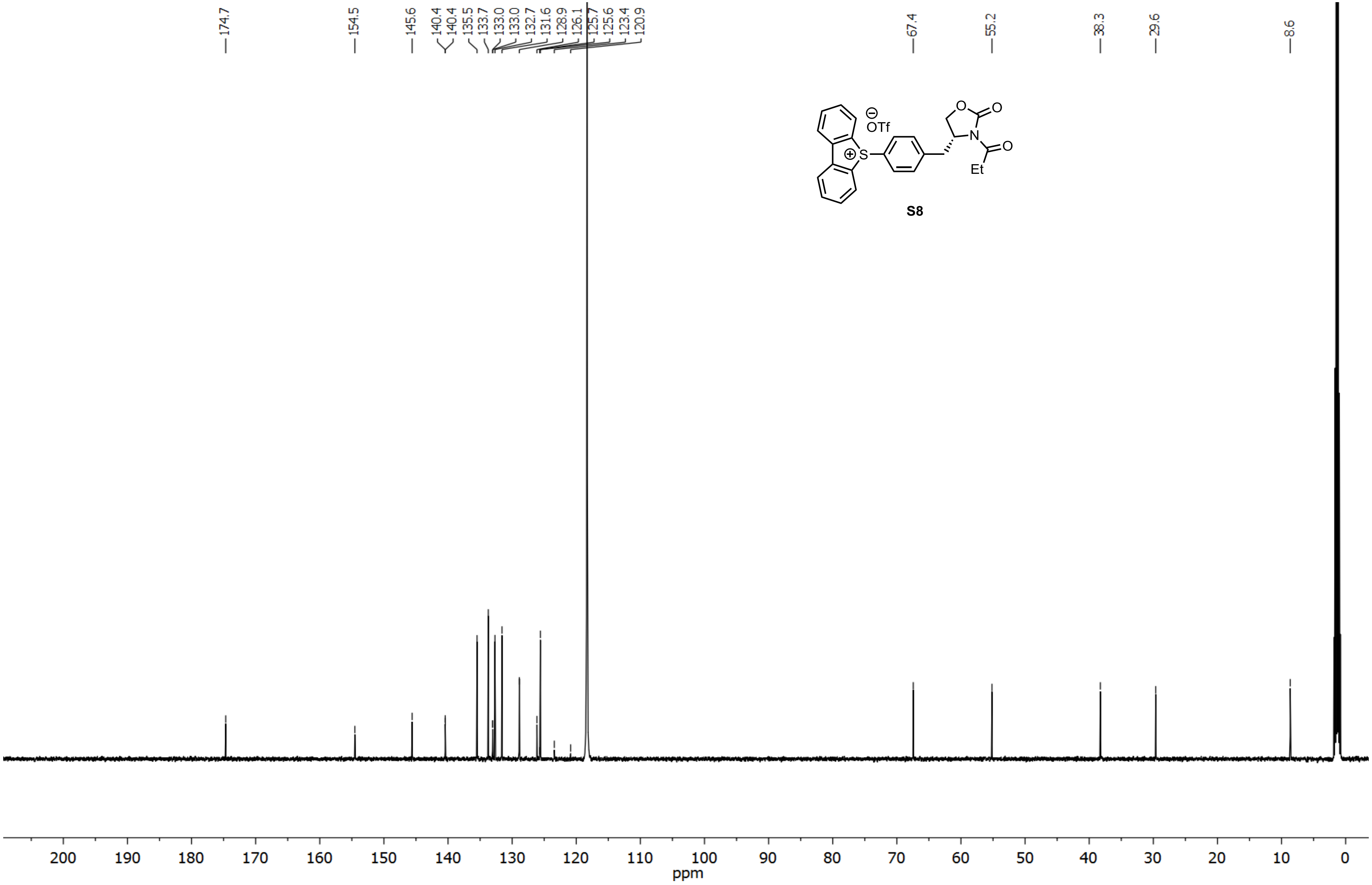

^{19}F NMR of (*R*)-(−)-4-benzyl-3-propionyl-2-oxazolidinone-derived dibenzothiophenium salt S8

CD$_3$CN, 298 K

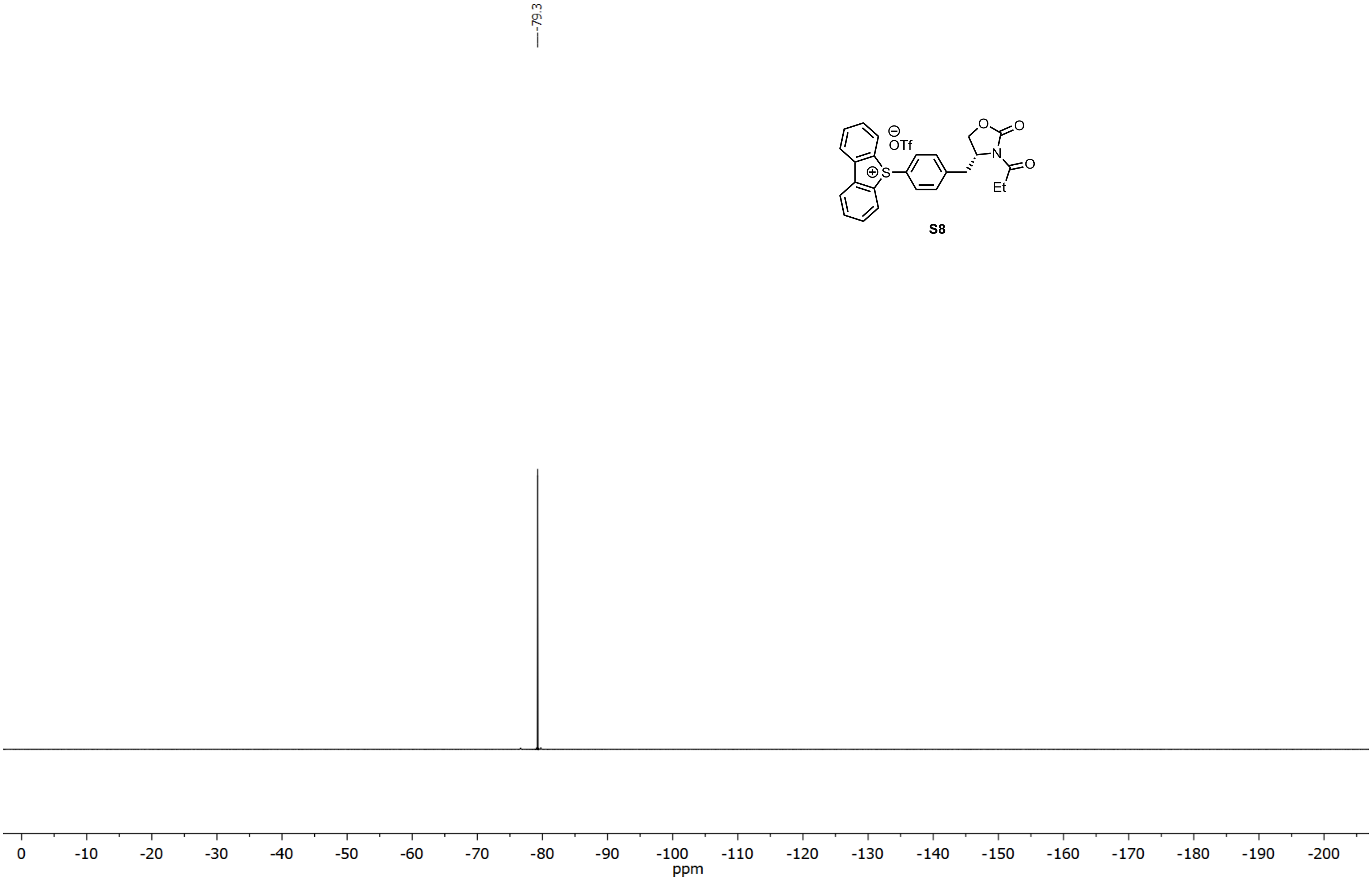

S8

^{1}H NMR of 3-chloro-6-methyldibenzo[*c*,*f*][1,2]thiazepin-11(6*H*)-one 5,5-dioxide-derived dibenzothiophenium salt S9

CD_3CN, 298 K

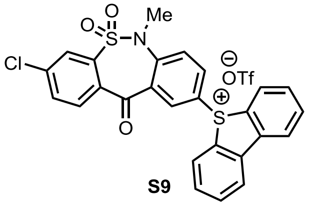

S9

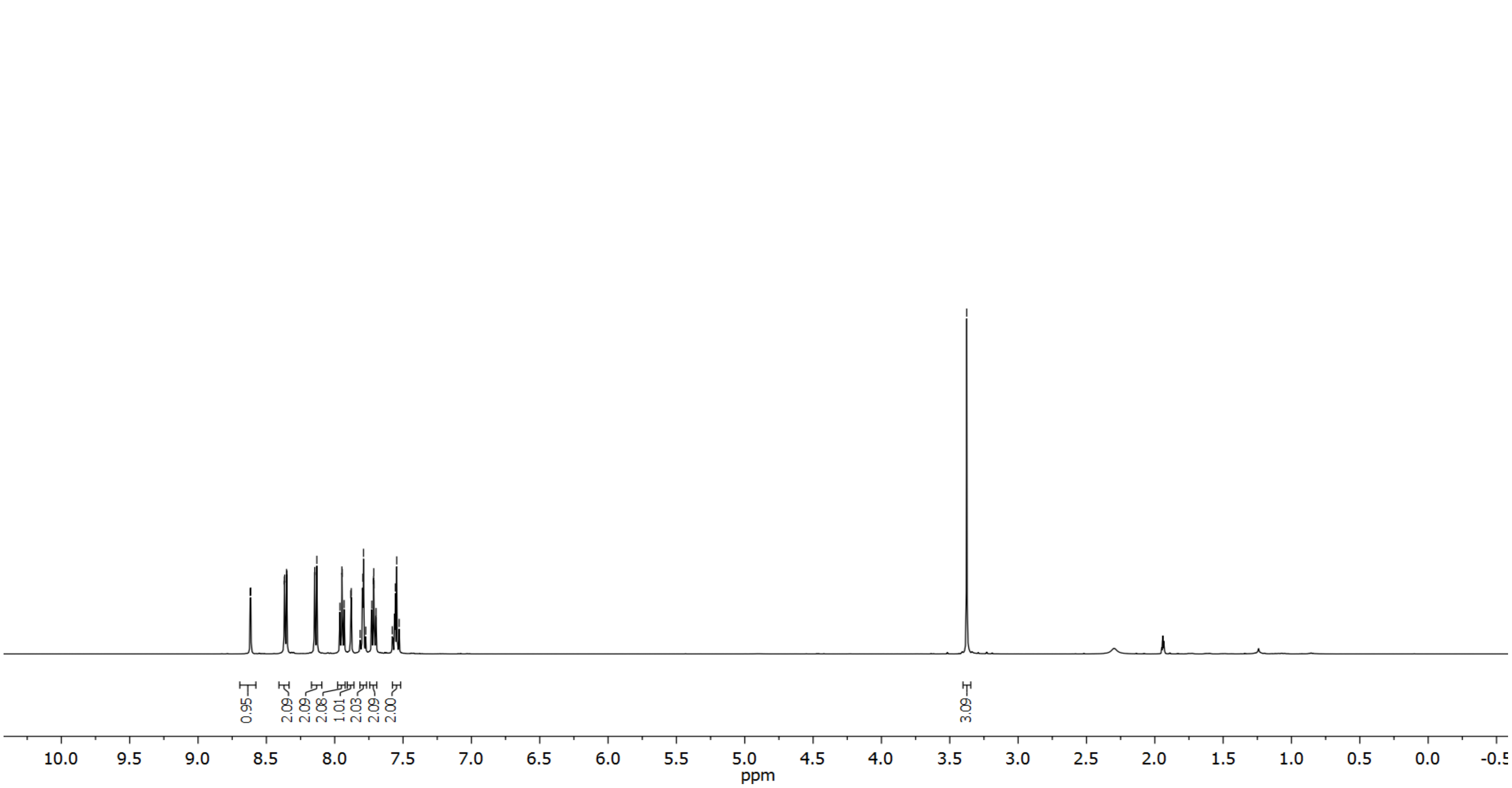

^{13}C NMR of 3-chloro-6-methyldibenzo[*c*,*f*][1,2]thiazepin-11(6*H*)-one 5,5-dioxide-derived dibenzothiophenium salt S9

CD_3CN, 298 K

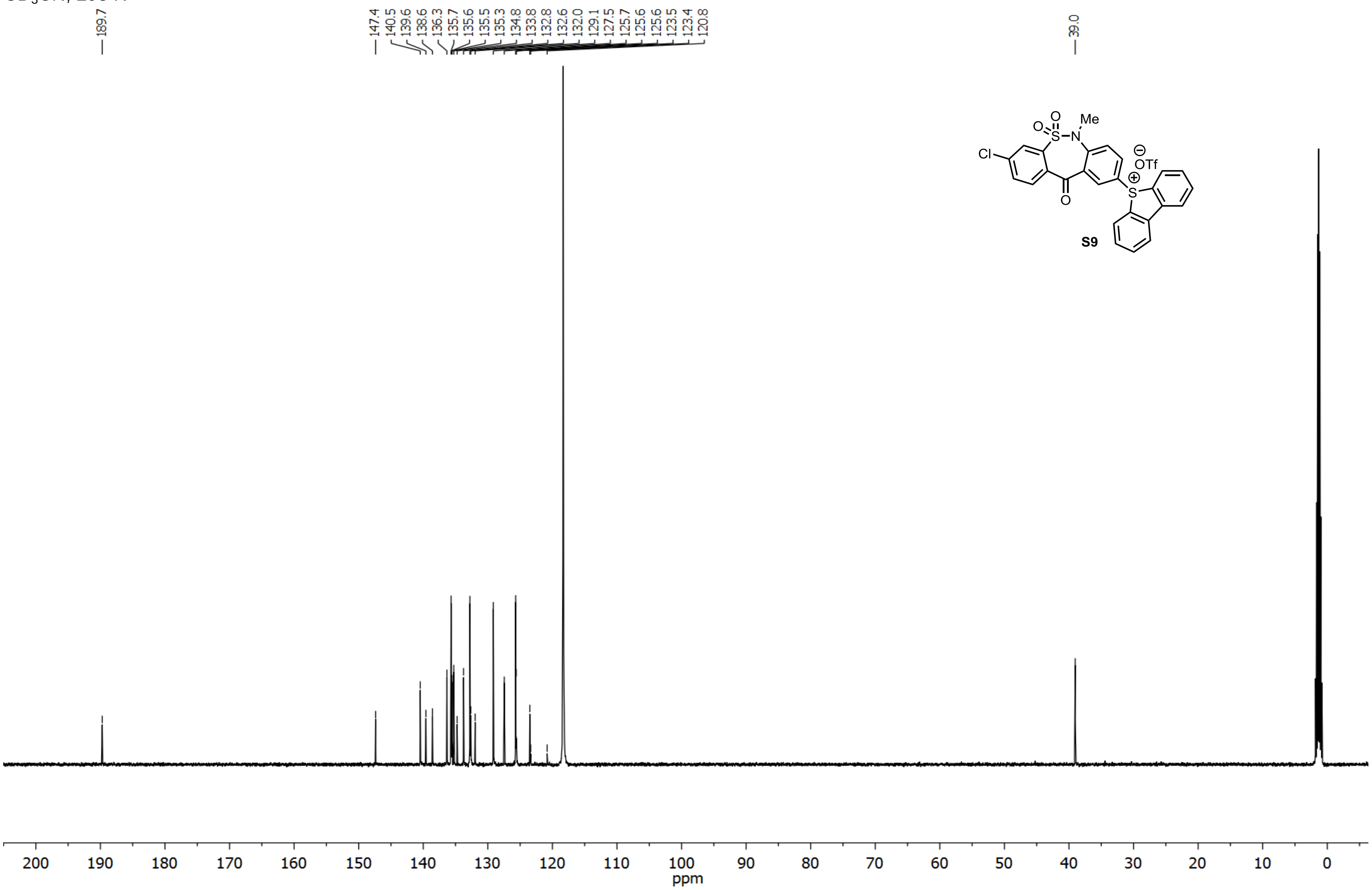

^{19}F NMR of 3-chloro-6-methyldibenzo[*c*,*f*][1,2]thiazepin-11(6*H*)-one 5,5-dioxide-derived dibenzothiophenium salt S9

CD_3CN, 298 K

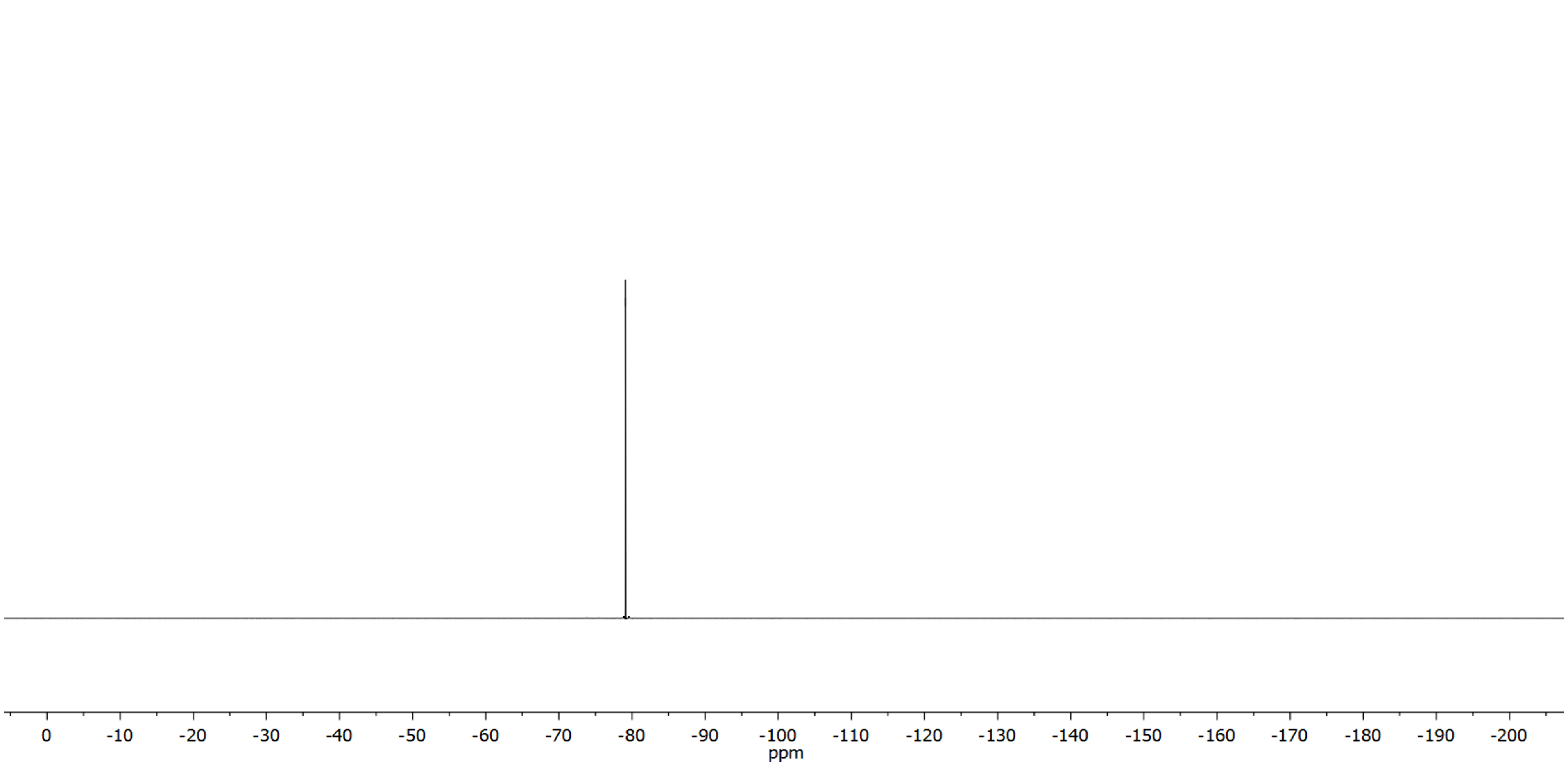

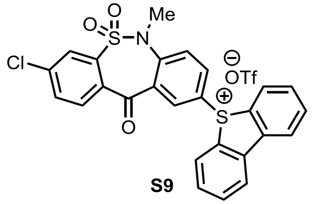

S9

^{1}H NMR of fenofibrate-derived dibenzothiophenium salt S10

CD$_3$CN, 298 K

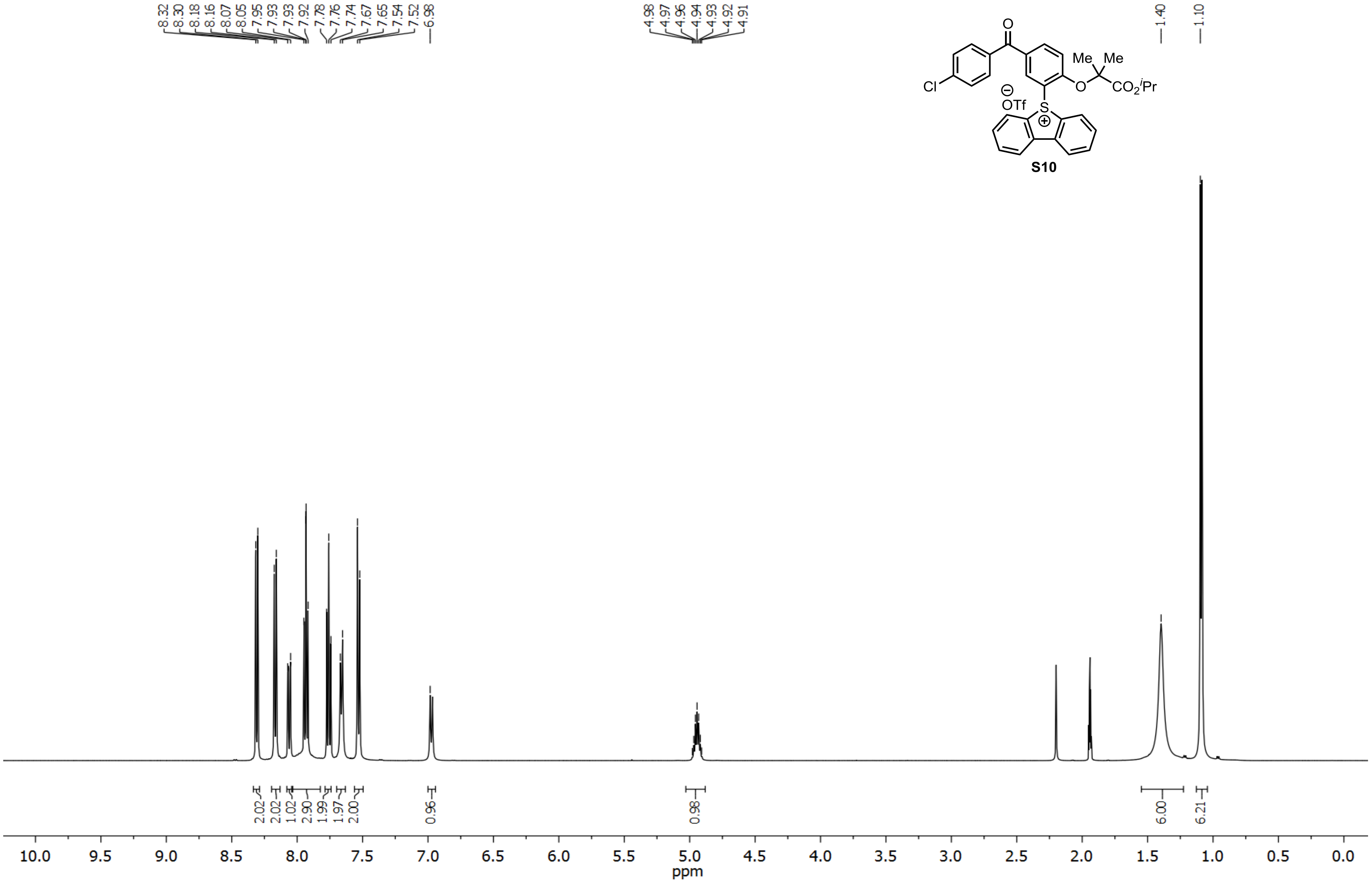

^{13}C NMR of fenofibrate-derived dibenzothiophenium salt S10

CD$_3$CN, 298 K

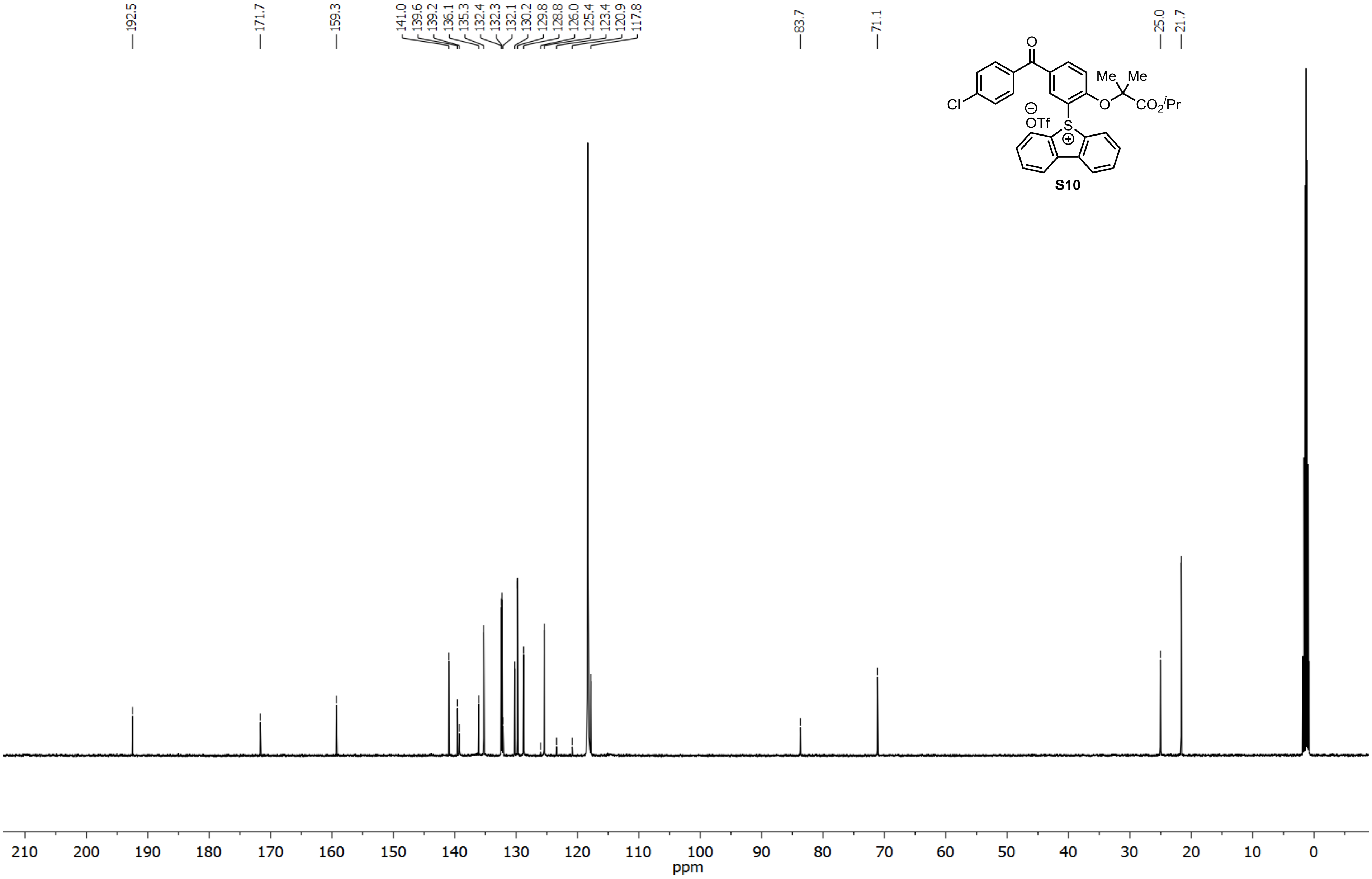

^{19}F NMR of fenofibrate-derived dibenzothiophenium salt S10

CD_3CN, 298 K

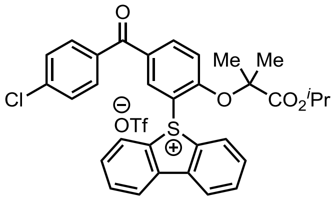

S10

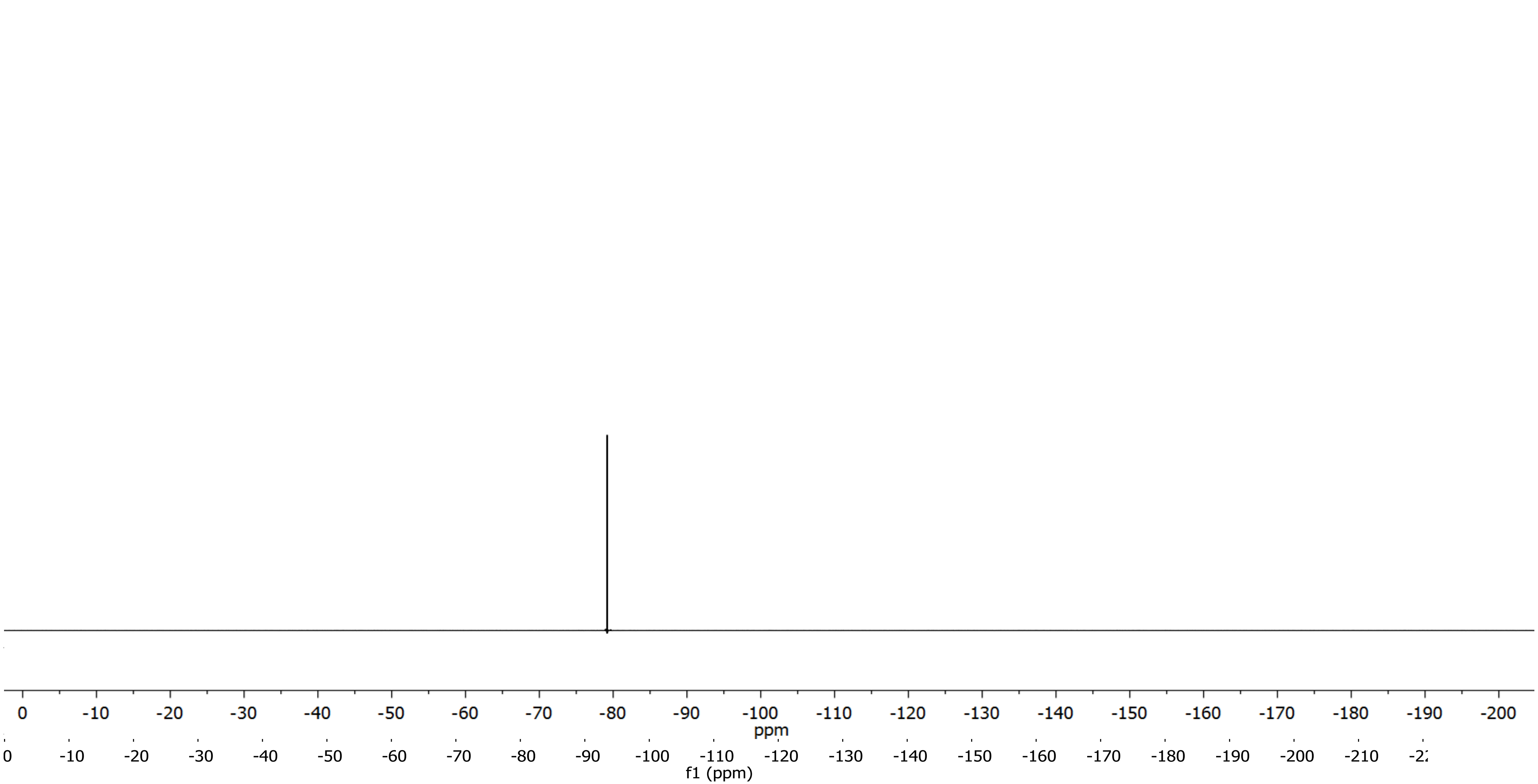

^{1}H NMR of cyclopropylbenzene-derived 3,7-di-*tert*-butyldibenzothiophenium salt S11

CD_3CN, 298 K

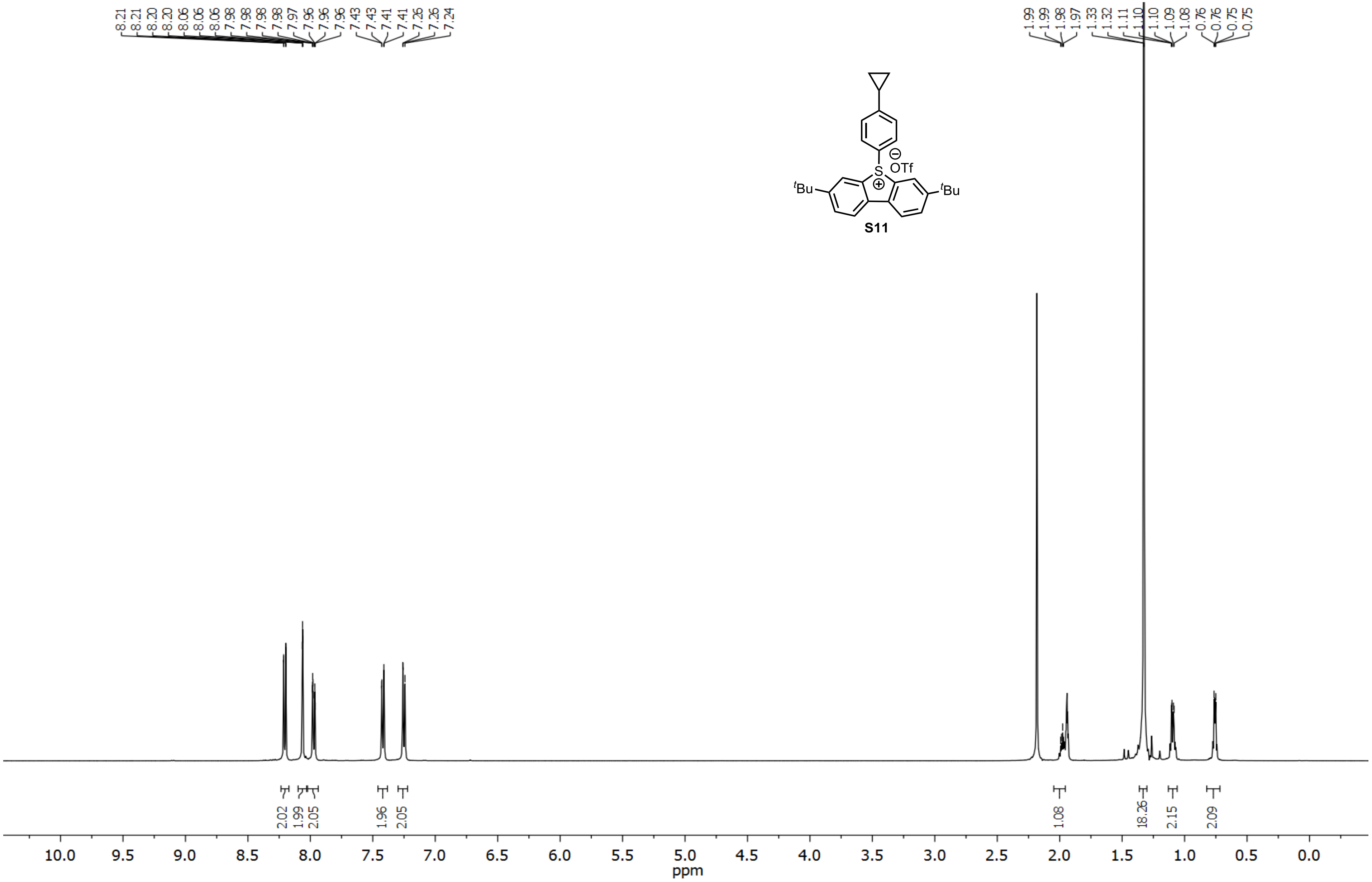

^{13}C NMR of cyclopropylbenzene-derived 3,7-di-*tert*-butyldibenzothiophenium salt S11

CD_3CN, 298 K

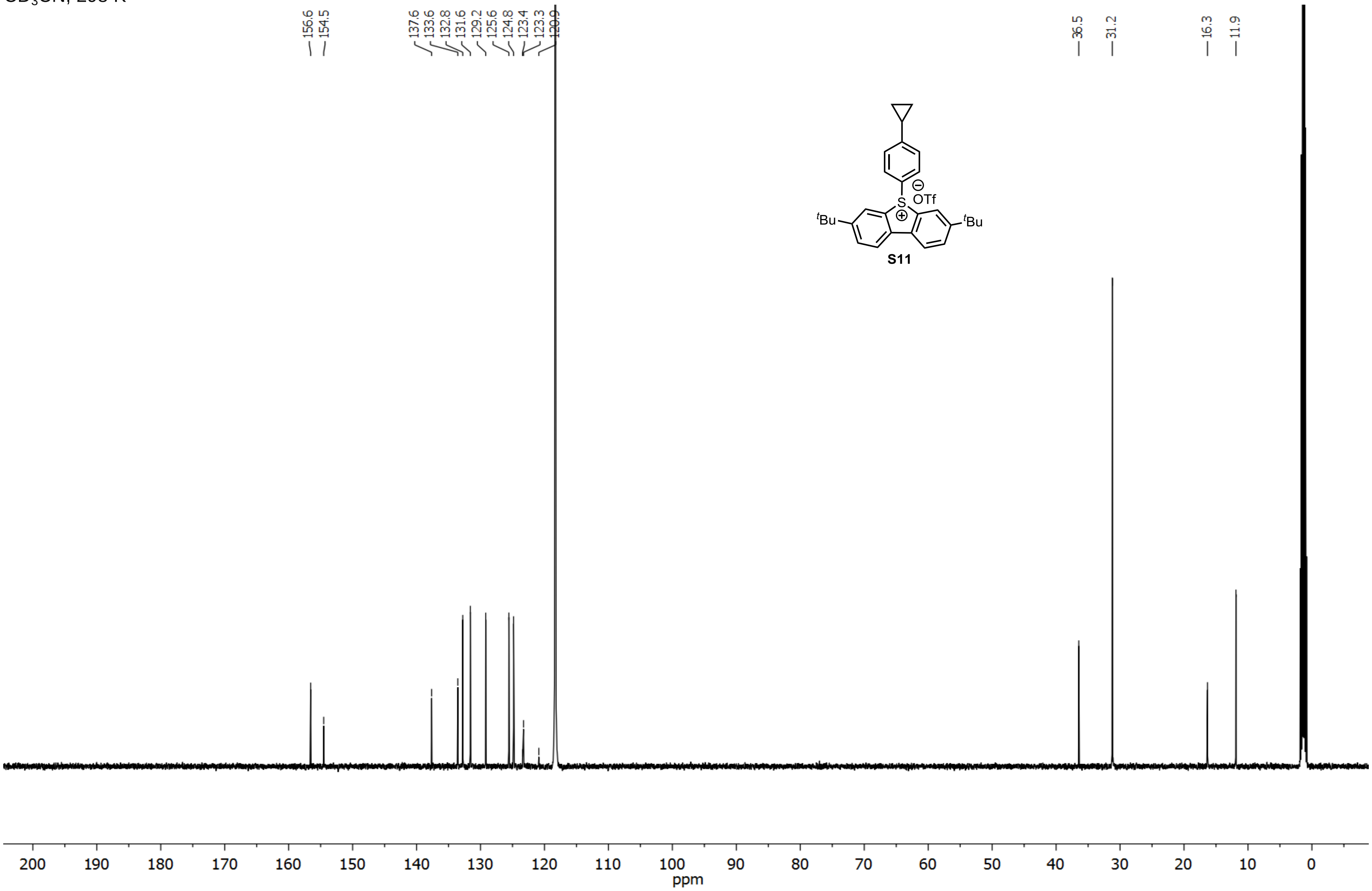

^{19}F NMR of cyclopropylbenzene-derived 3,7-di-*tert*-butyldibenzothiophenium salt S11

CD_3CN, 298 K

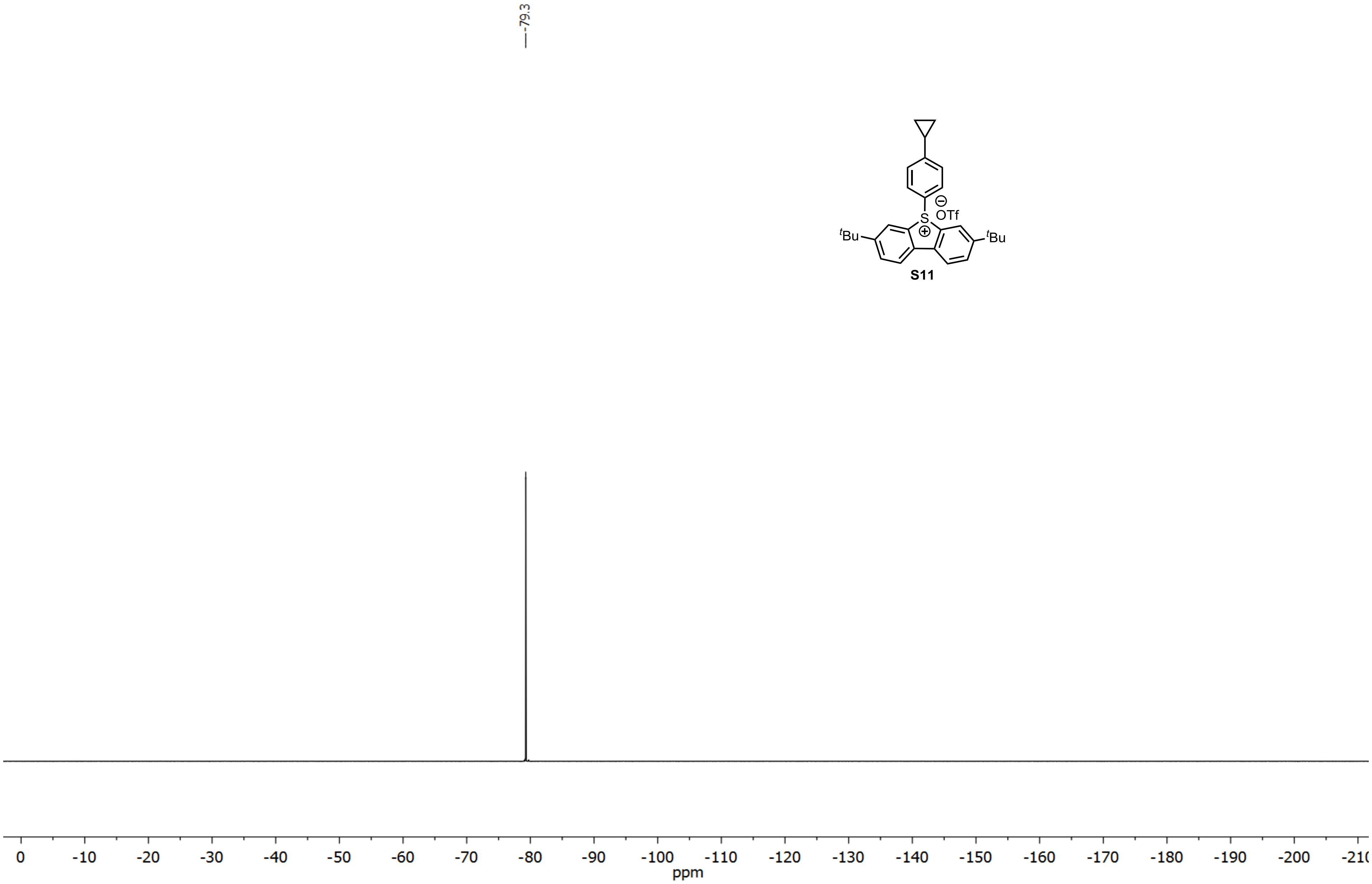

^{1}H NMR of 6-methoxyquinoline-derived dibenzothiophenium salt S12

CD$_3$CN, 298 K

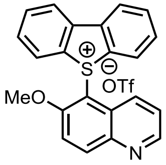

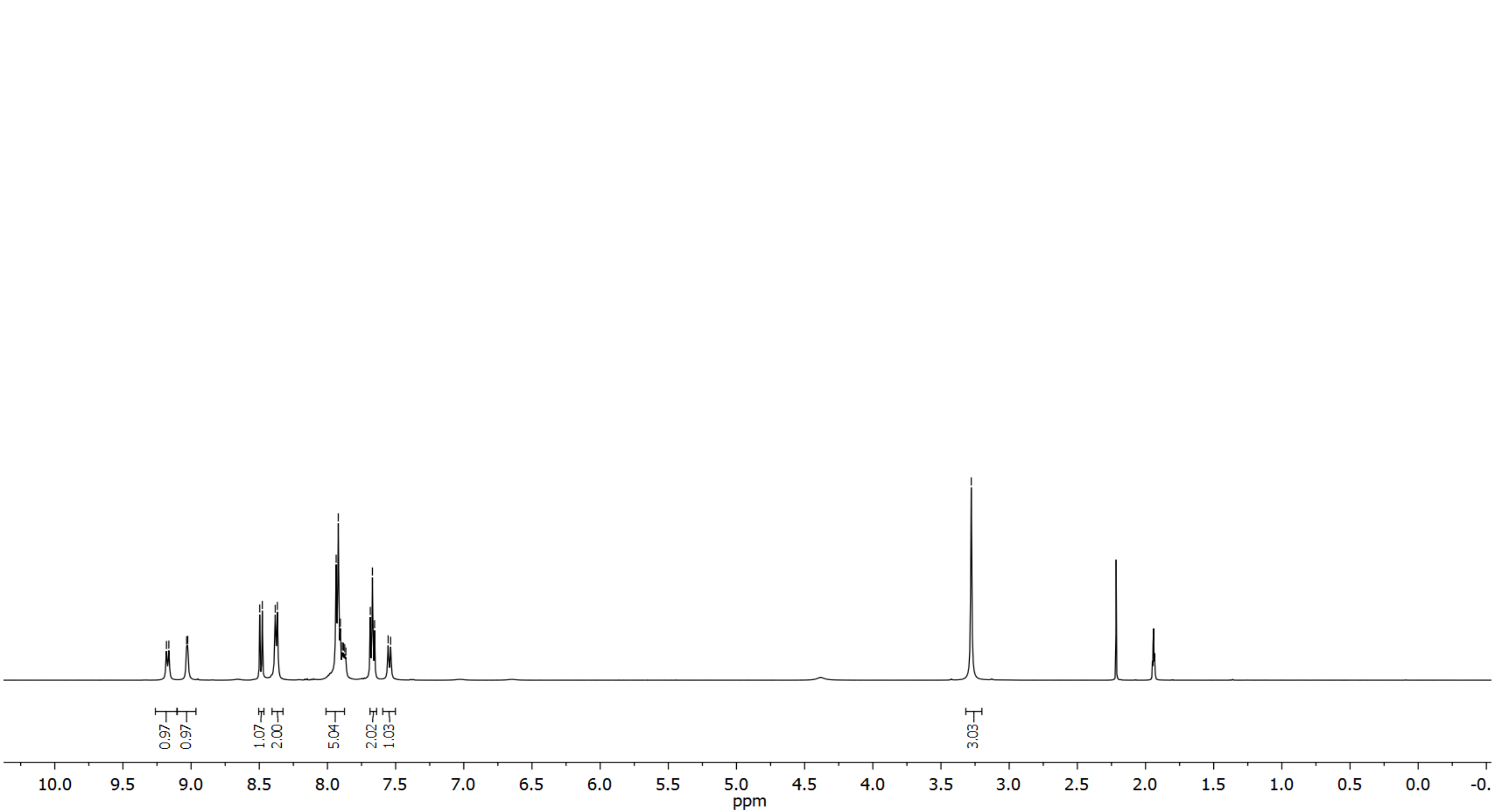

^{13}C NMR of 6-methoxyquinoline-derived dibenzothiophenium salt S12

CD_3CN, 298 K

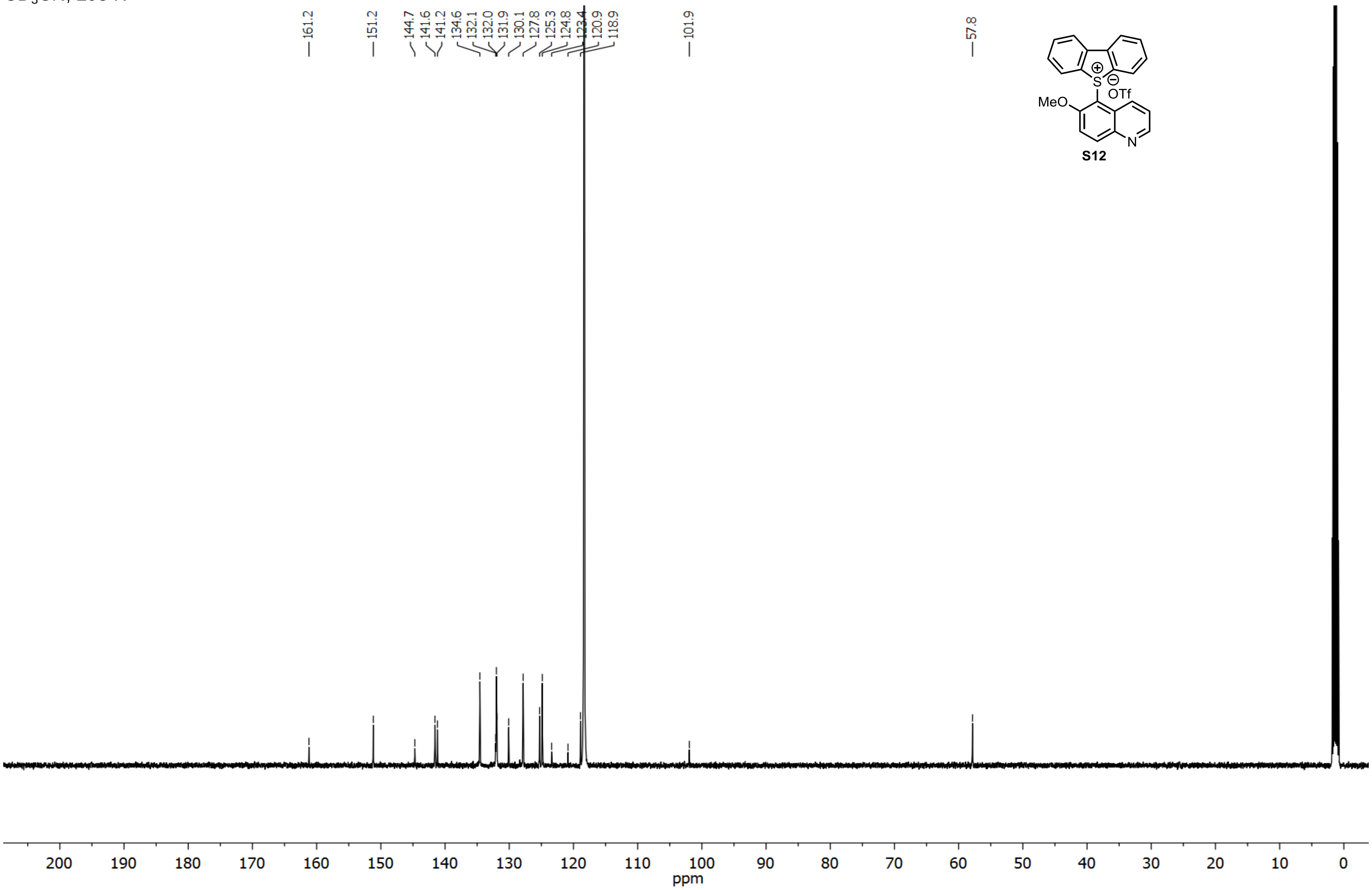

^{19}F NMR of 6-methoxyquinoline-derived dibenzothiophenium salt S12

CD_3CN, 298 K

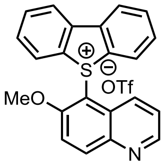

S12

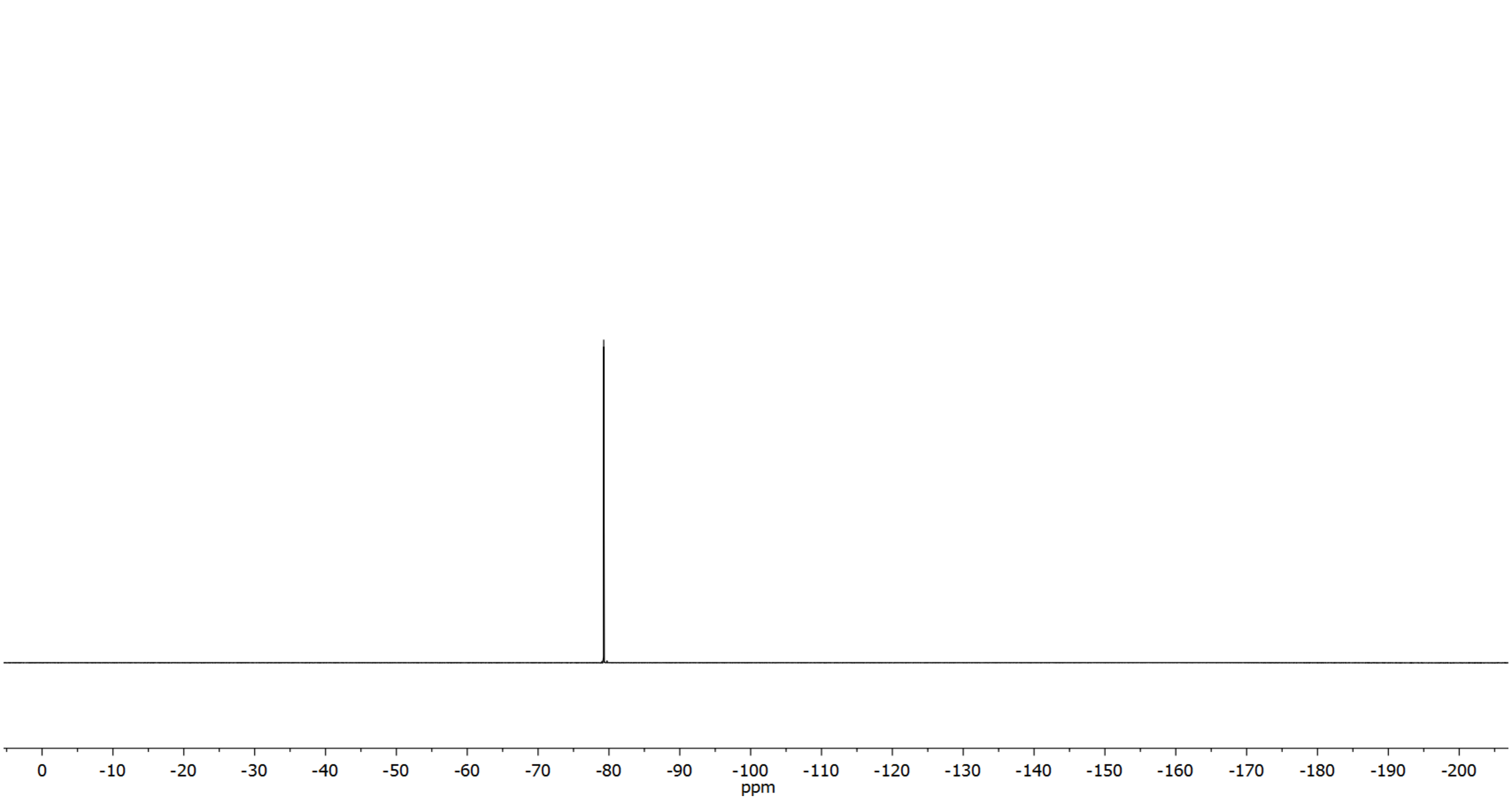

^{1}H NMR of 1-chloro-3-phenylpropane-derived 3,7-di-*tert*-butyldibenzothiophenium salt S13

CD_2Cl_2, 298 K

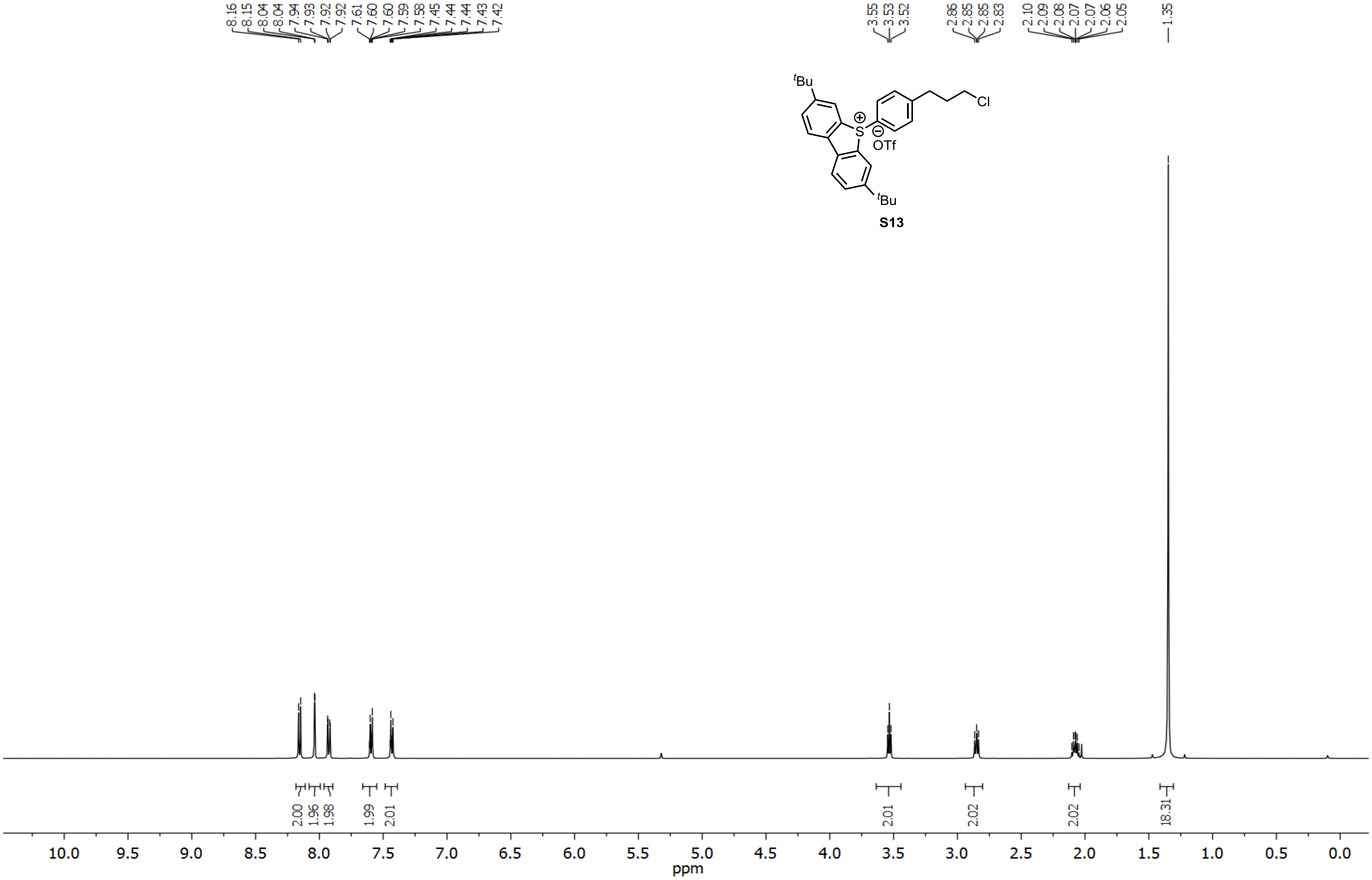

^{13}C NMR of 1-chloro-3-phenylpropane-derived 3,7-di-*tert*-butyldibenzothiophenium salt S13

CD_2Cl_2, 298 K

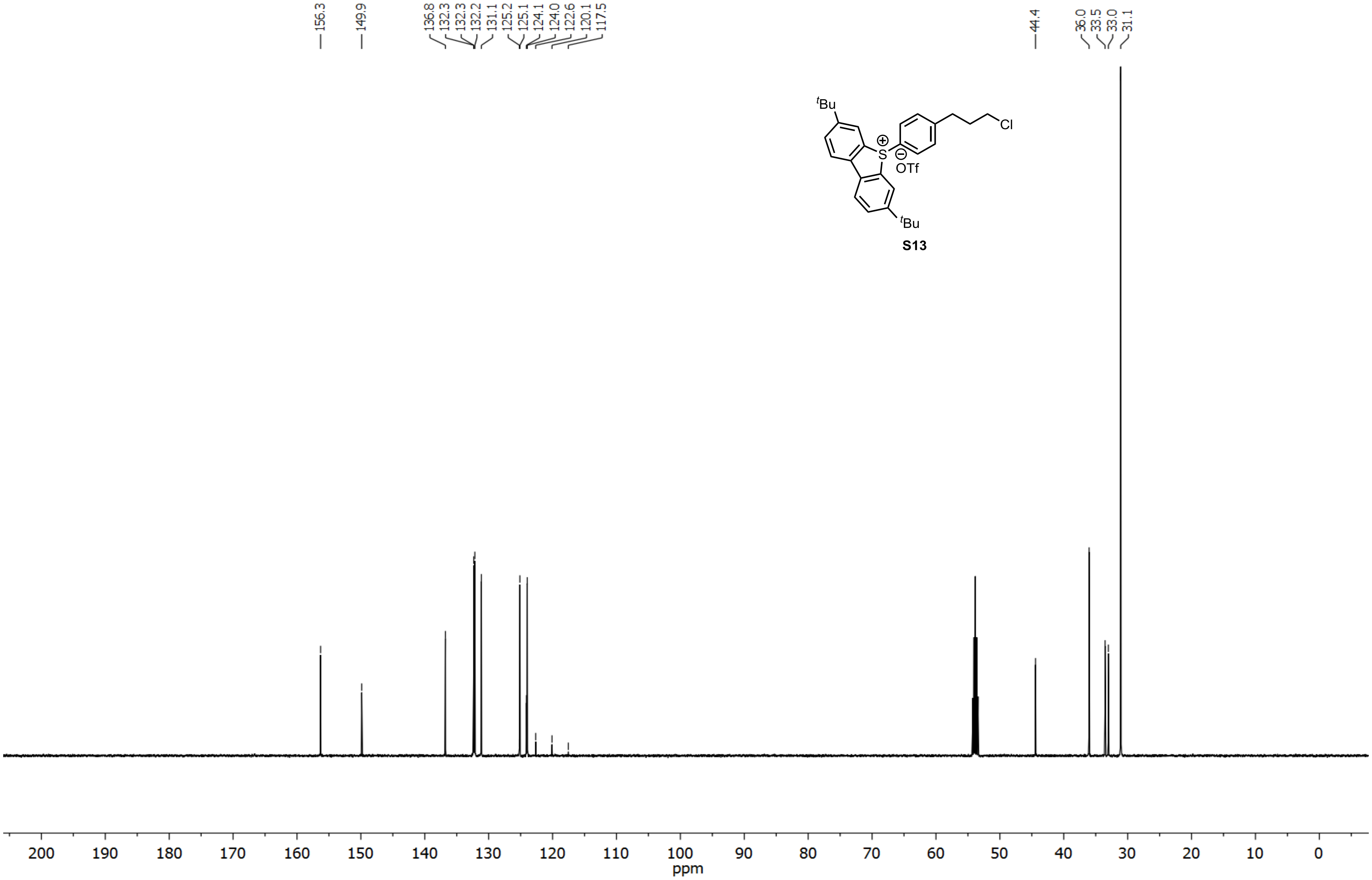

^{19}F NMR of 1-chloro-3-phenylpropane-derived 3,7-di-*tert*-butyldibenzothiophenium salt S13

CD_2Cl_2, 298 K

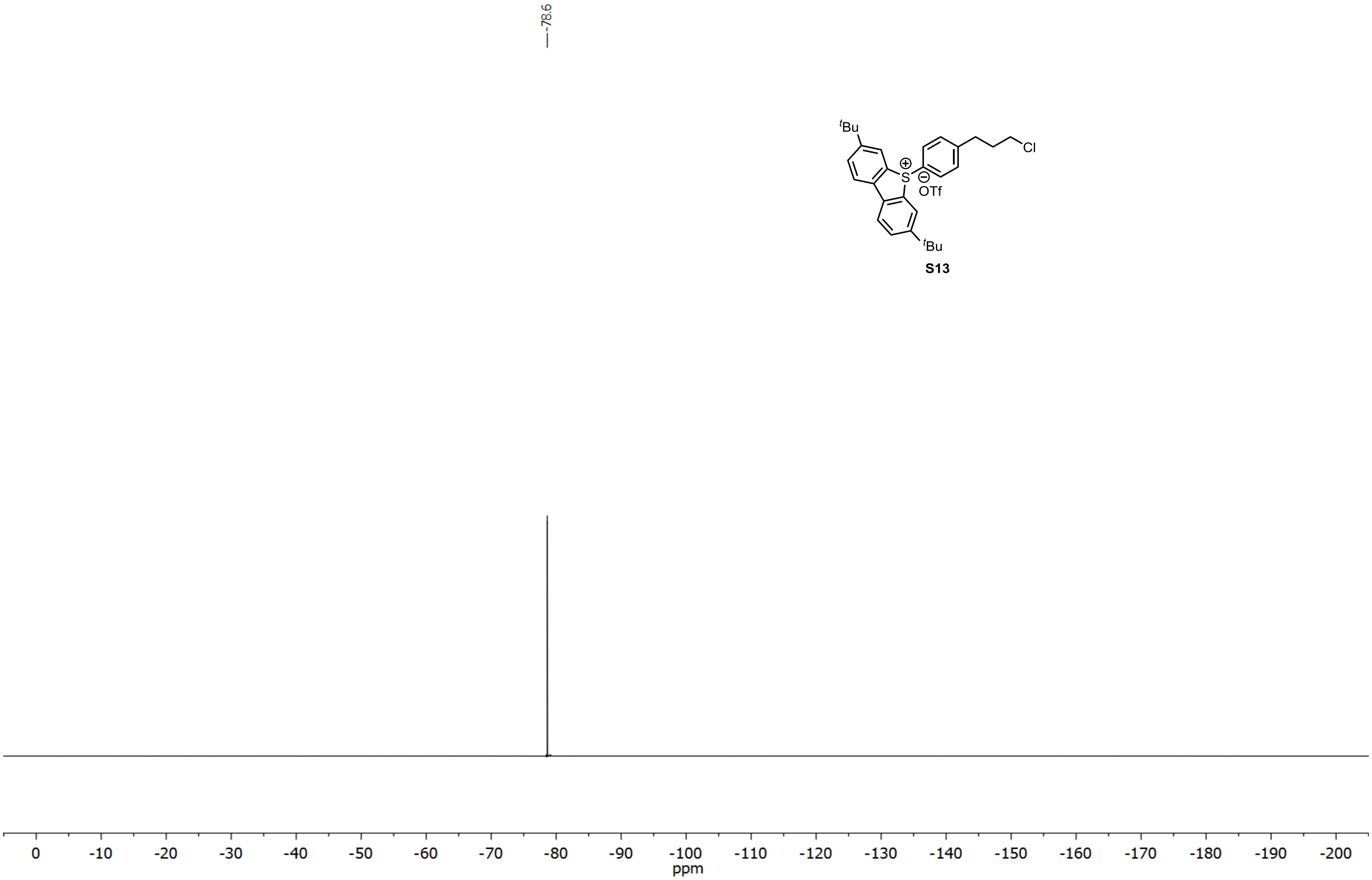

^{1}H NMR of *N*-benzyl-nimesulide-derived dibenzothiophenium salt S14

CD_3CN, 298 K

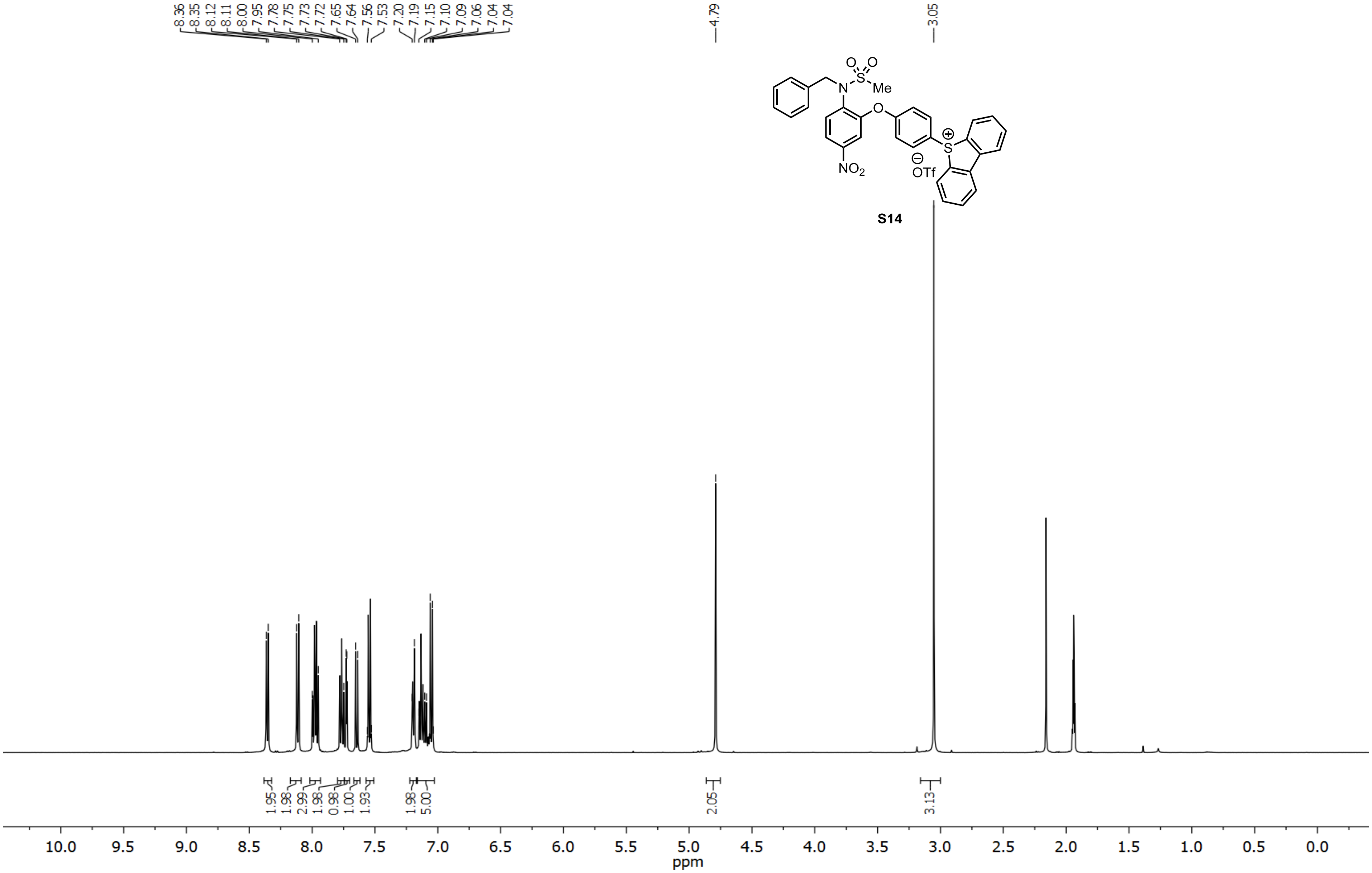

^{13}C NMR of *N*-benzyl-nimesulide-derived dibenzothiophenium salt S14

CD_3CN, 298 K

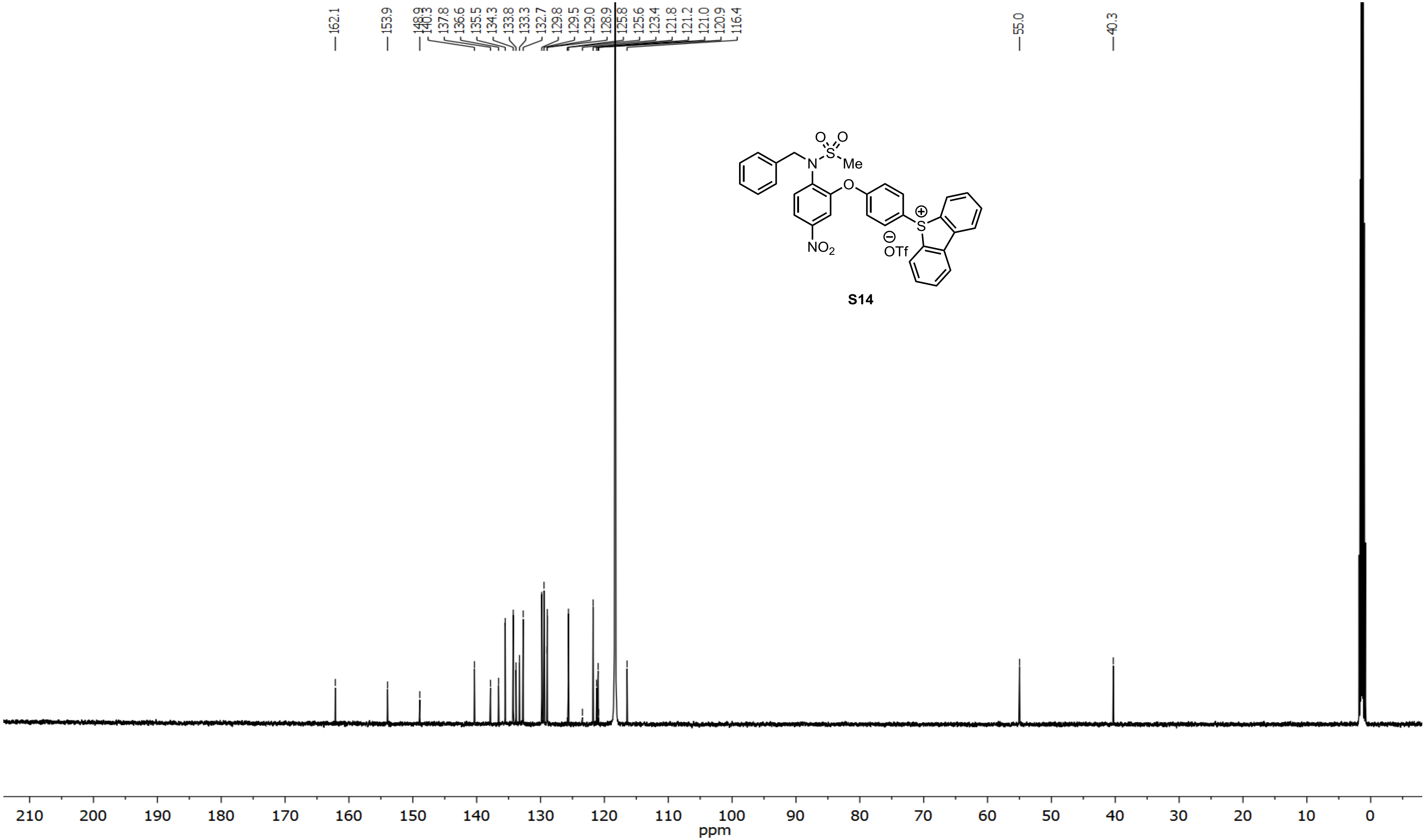

^{19}F NMR of *N*-benzyl-nimesulide-derived dibenzothiophenium salt S14

CD_3CN, 298 K

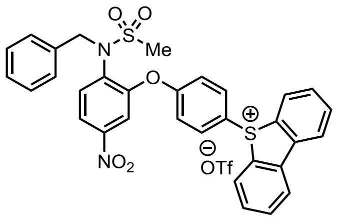

S14

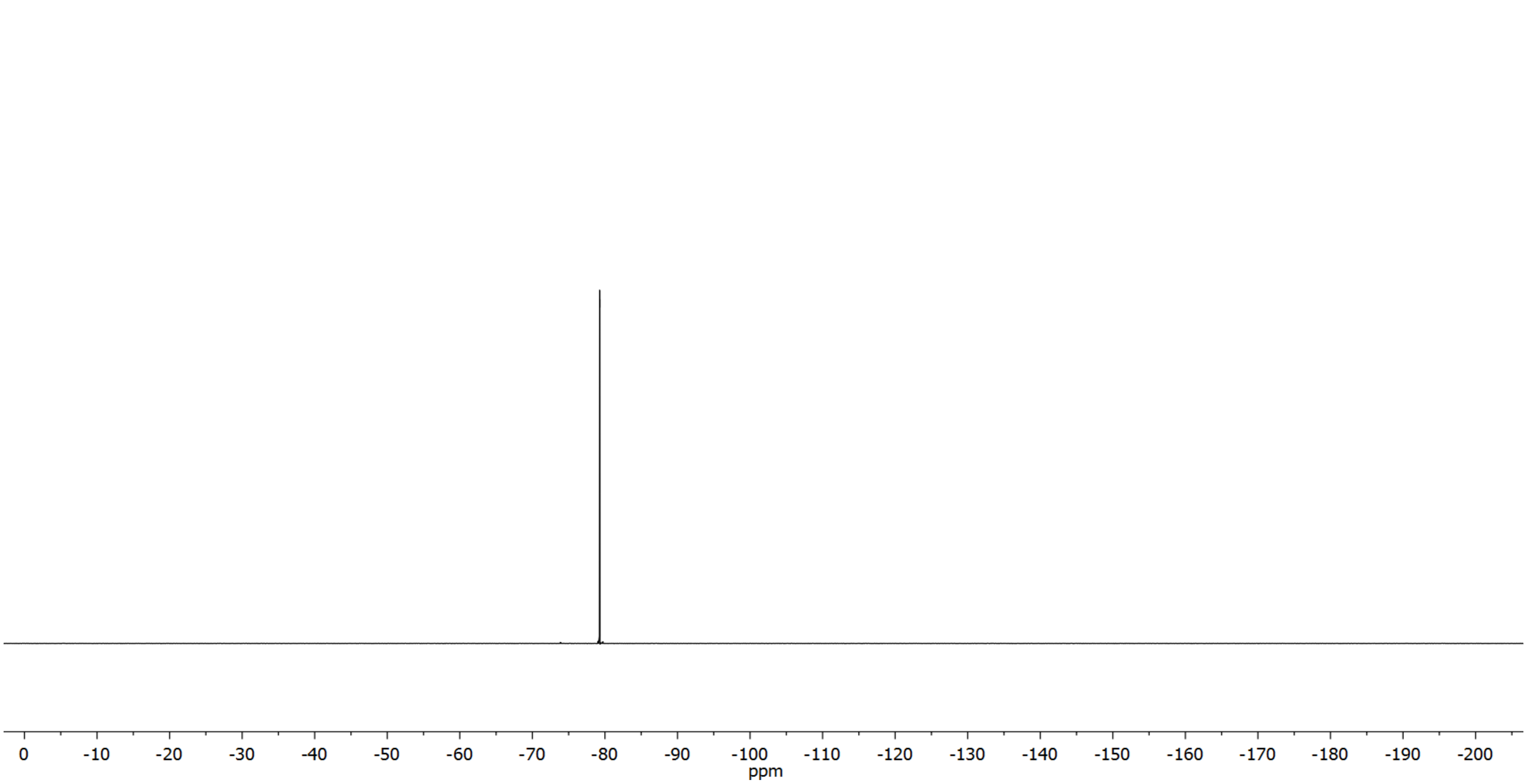

^{1}H NMR of flurbiprofen methylester-derived dibenzothiophenium salt S15

CD_3CN, 298 K

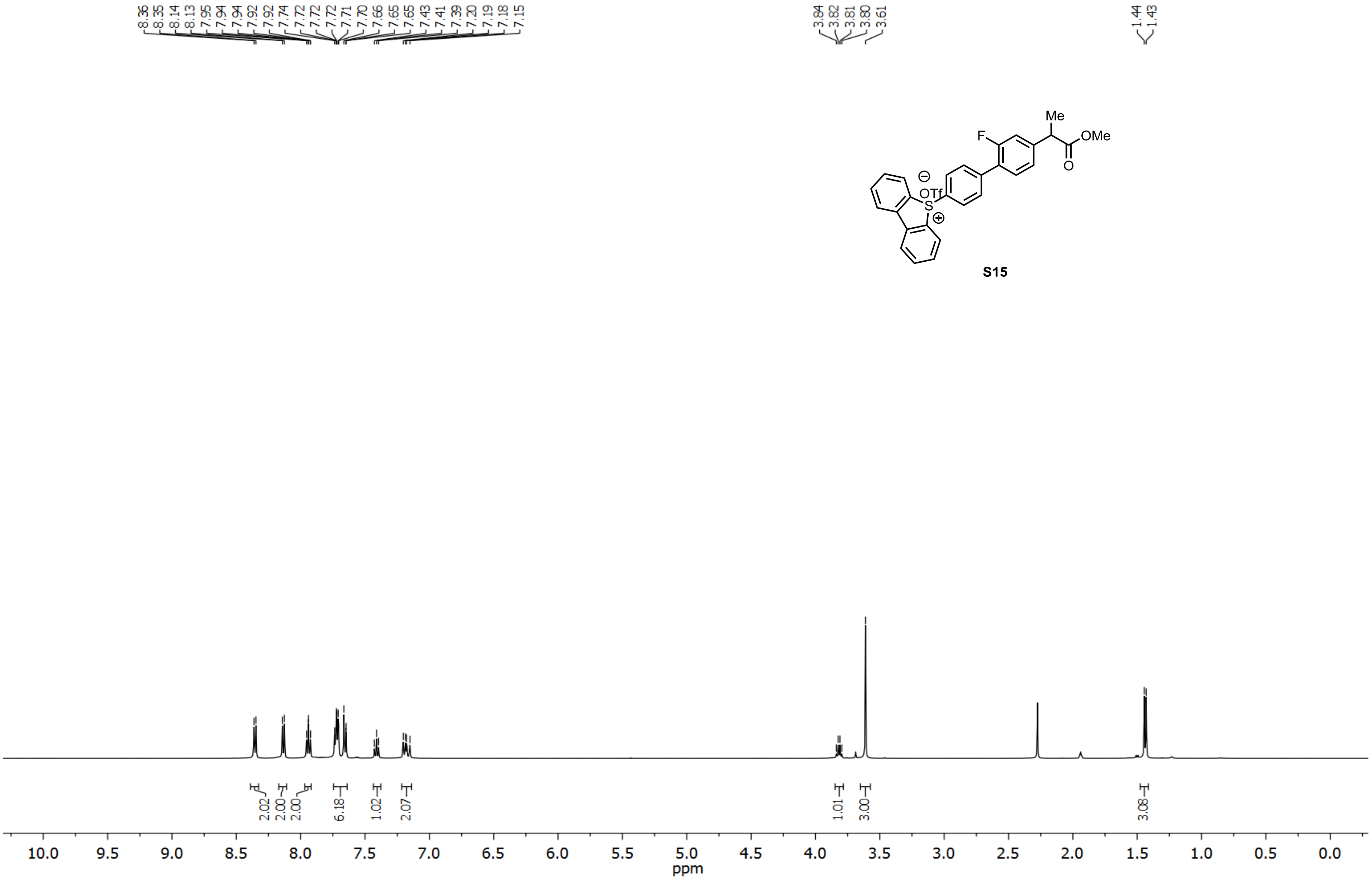

^{13}C NMR of flurbiprofen methylester-derived dibenzothiophenium salt S15

CD$_3$CN, 298 K

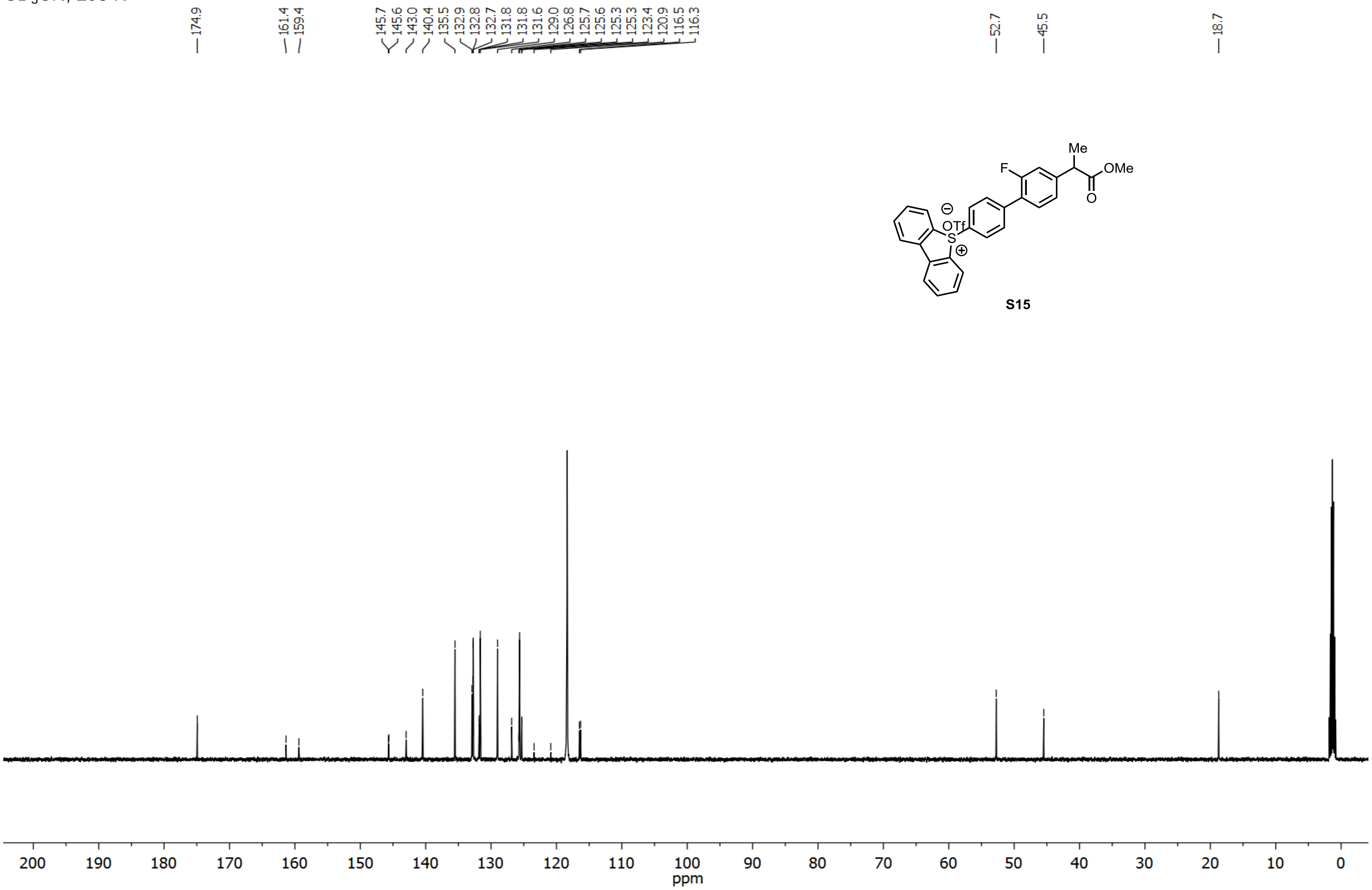

S15

^{19}F NMR of flurbiprofen methylester-derived dibenzothiophenium salt S15

CD_3CN, 298 K

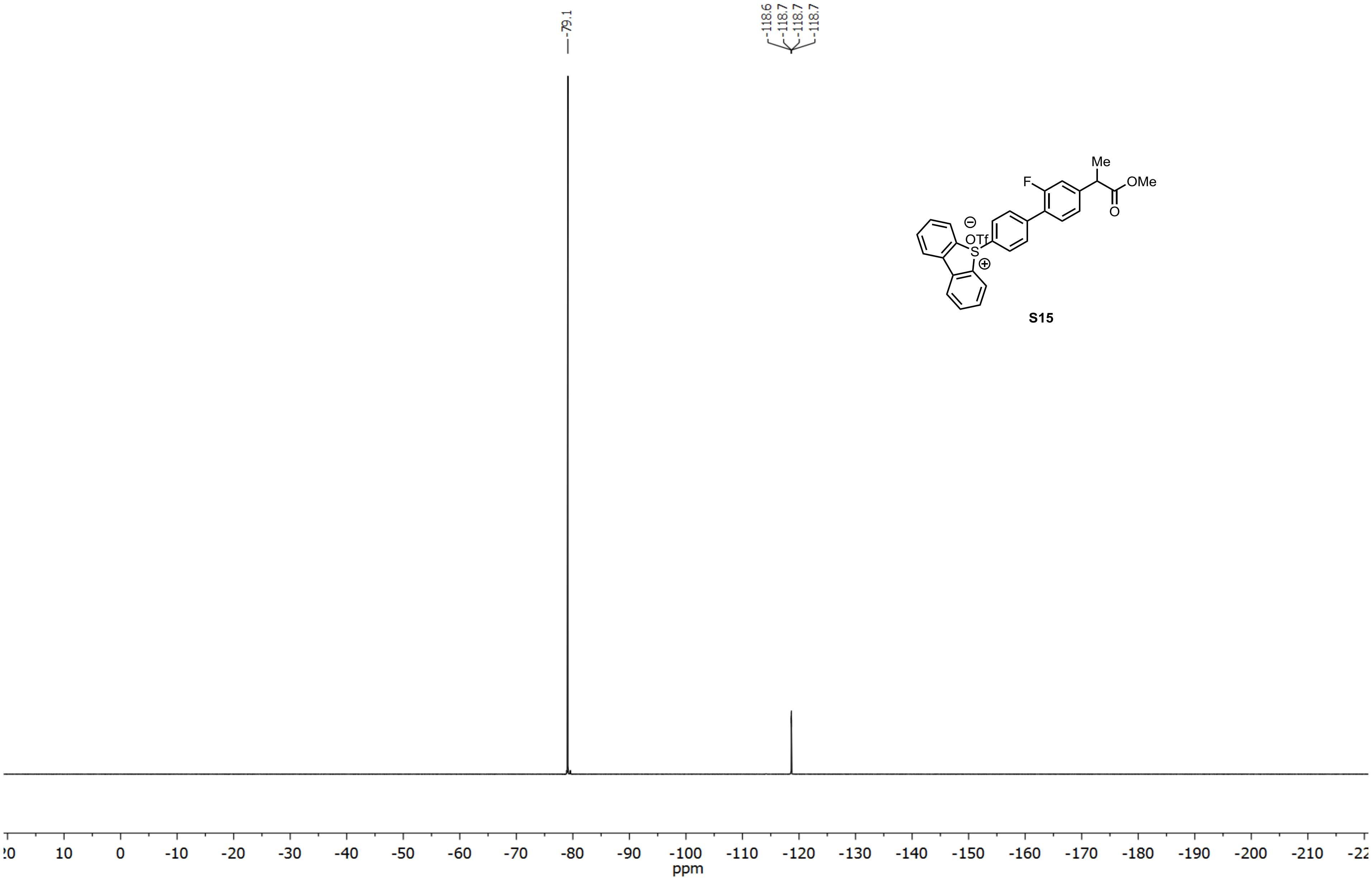

^{1}H NMR of anisole-derived 2,8-dimethoxydibenzothiophenium salt S16

CD_3CN, 298 K

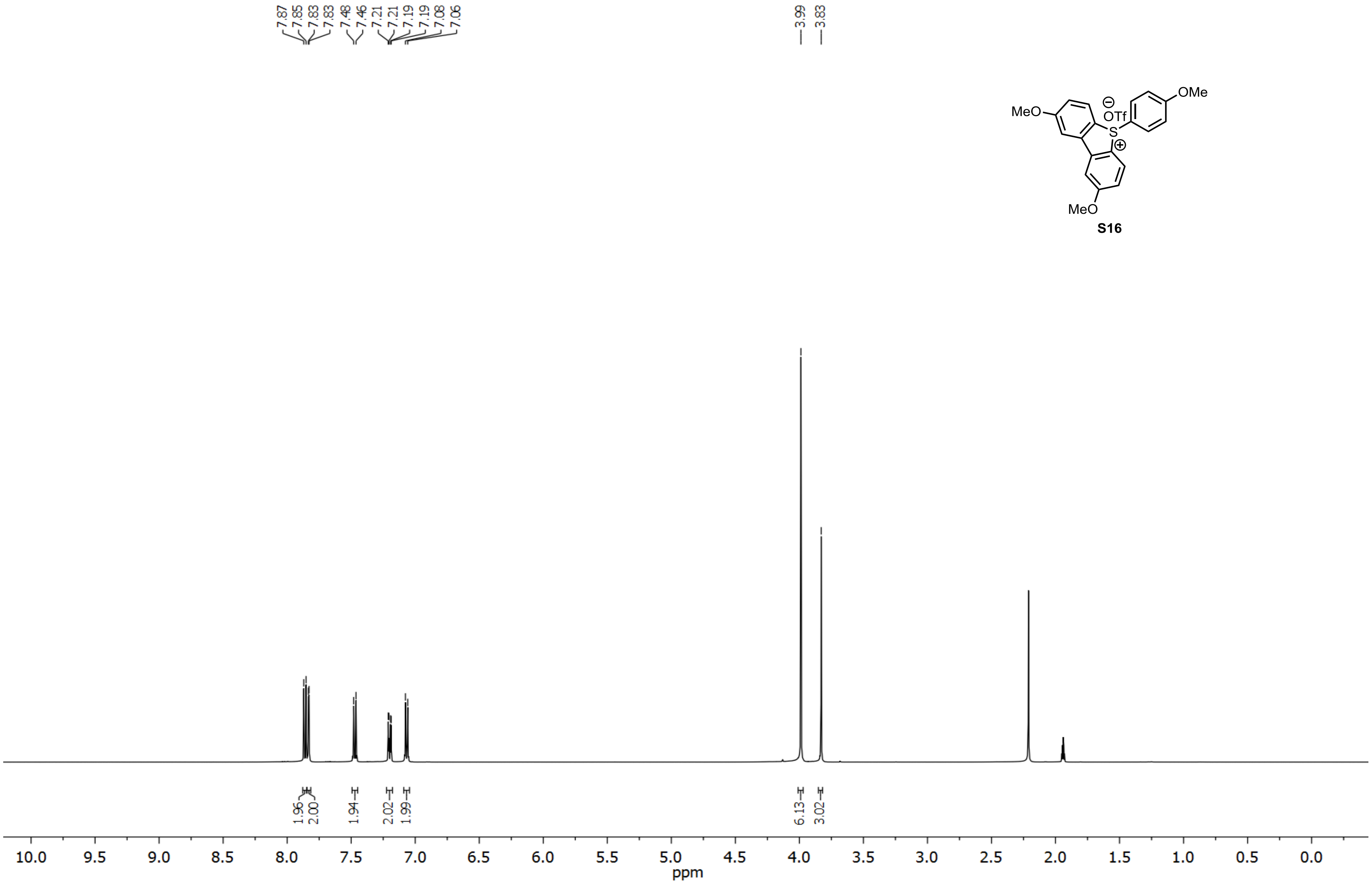

^{13}C NMR of anisole-derived 2,8-dimethoxydibenzothiophenium salt S16

CD_3CN, 298 K

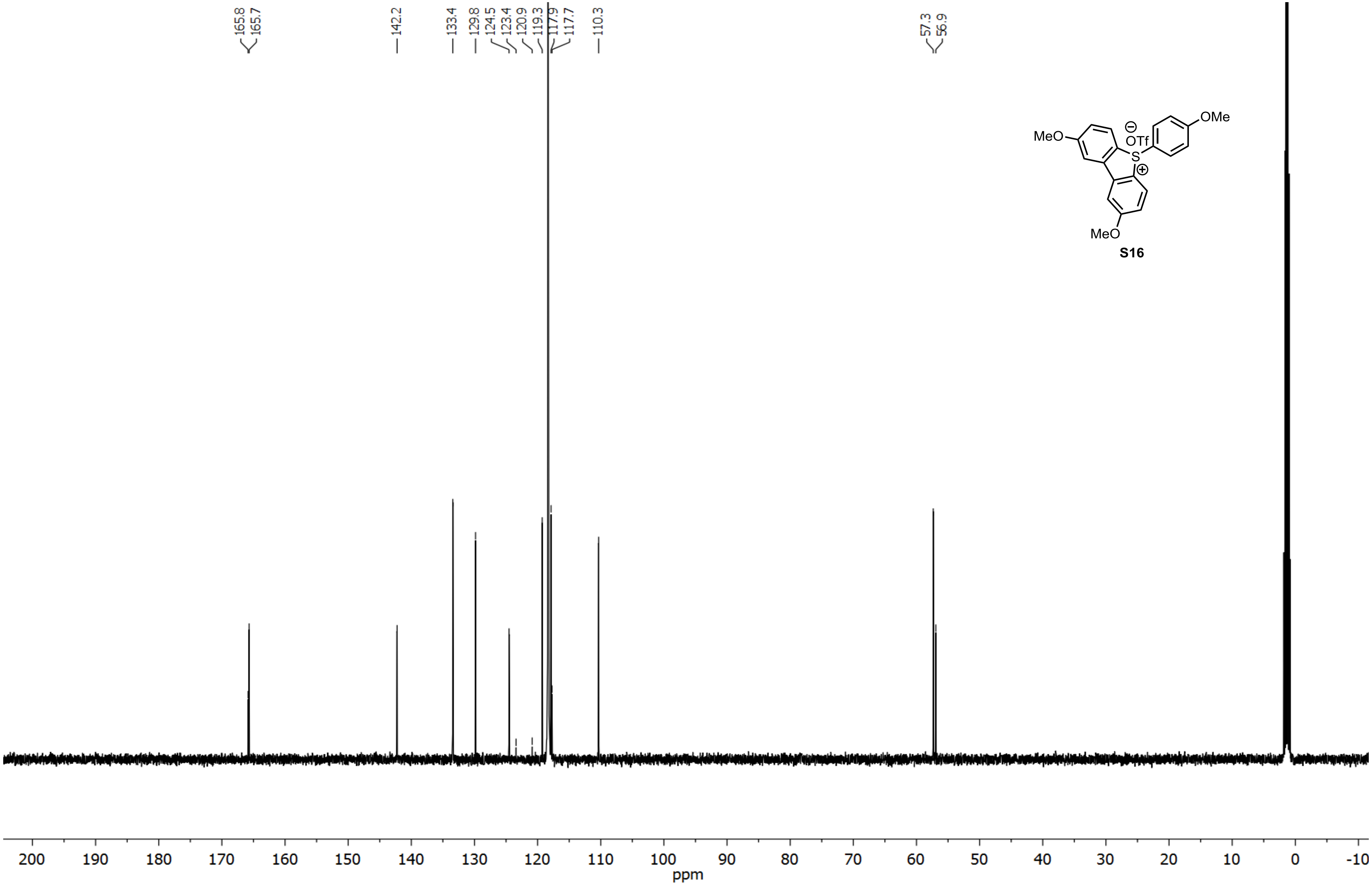

^{19}F NMR of anisole-derived 2,8-dimethoxydibenzothiophenium salt S16

CD_3CN, 298 K

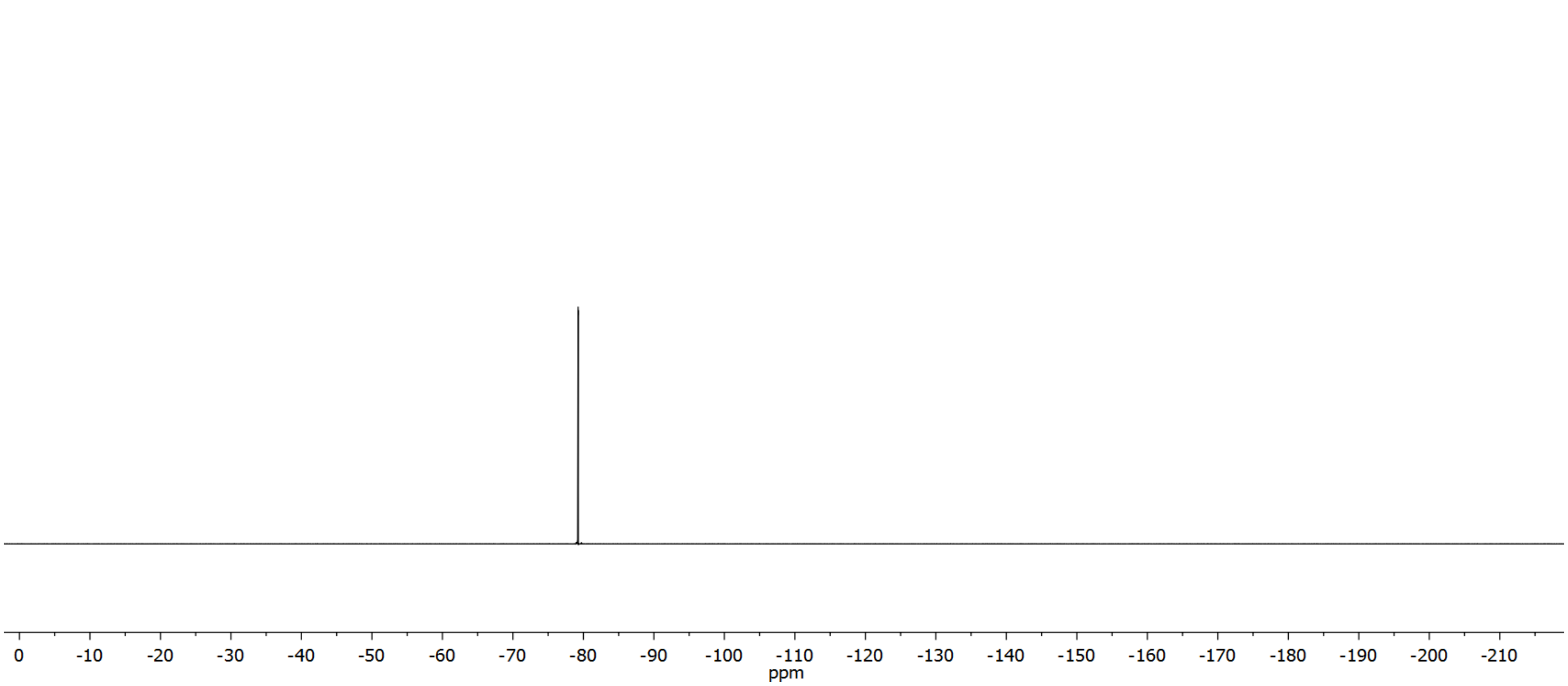

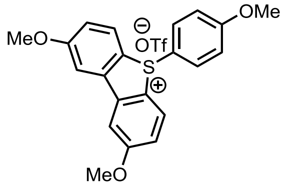

S16

^{1}H NMR of dicamba methylester (17)

$CDCl_3$, 298 K

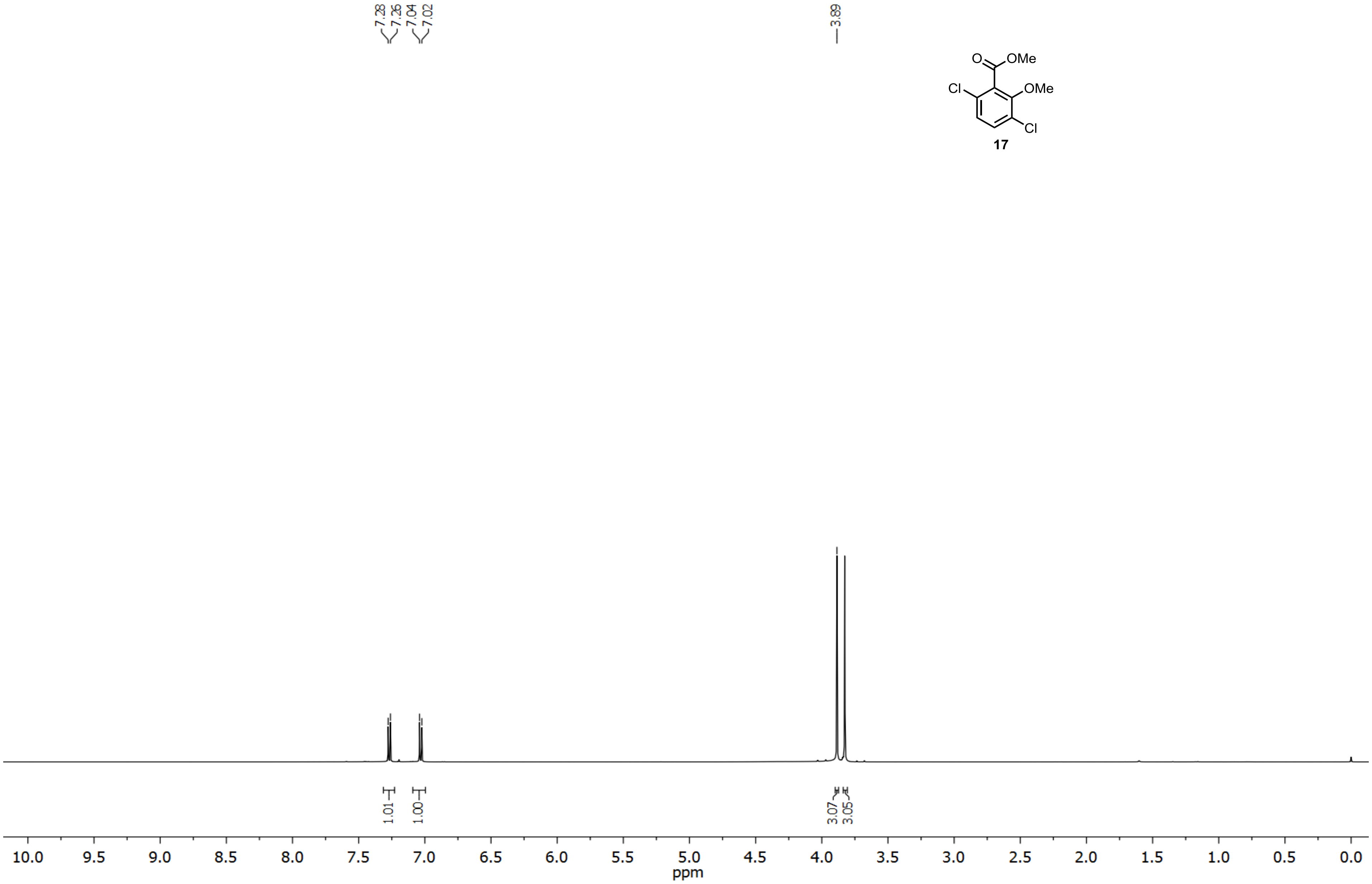

^{13}C NMR of dicamba methylester (17)

$CDCl_3$, 298 K

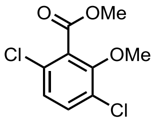

17

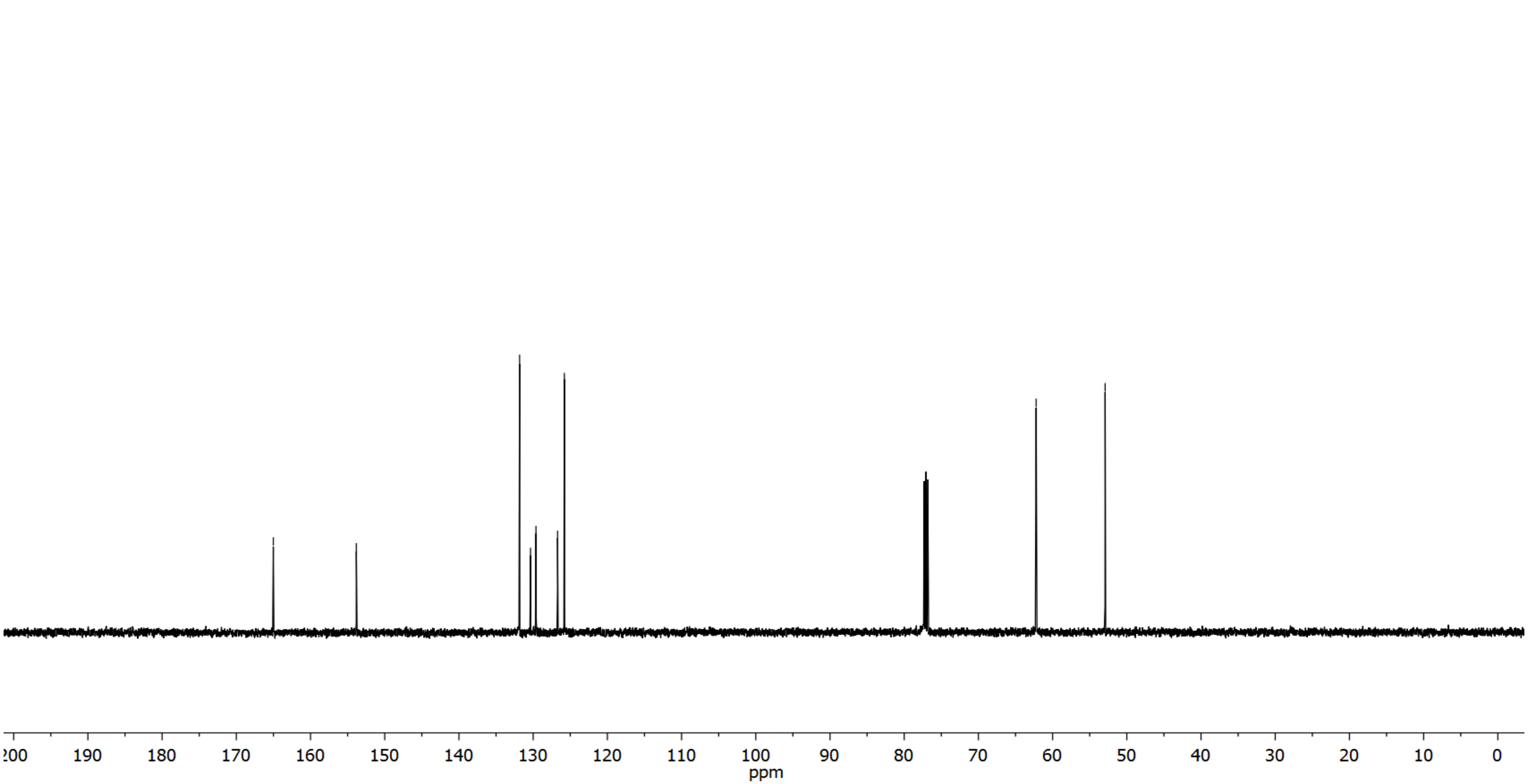

^{1}H NMR of dicamba methylester-derived dibenzothiophenium salt S17

CD_3CN, 298 K

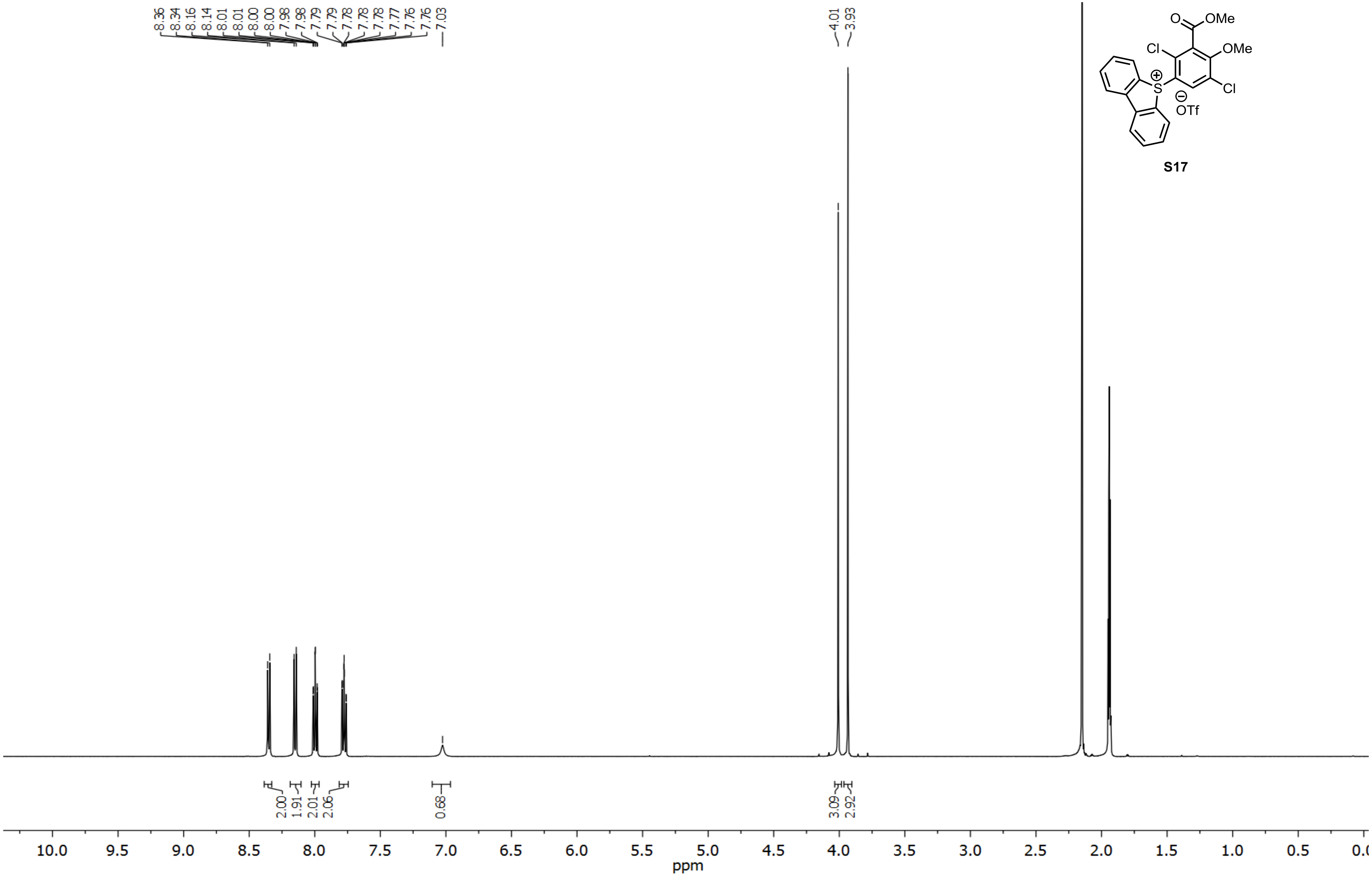

^{13}C NMR of dicamba methylester-derived dibenzothiophenium salt S17

CD_3CN, 298 K

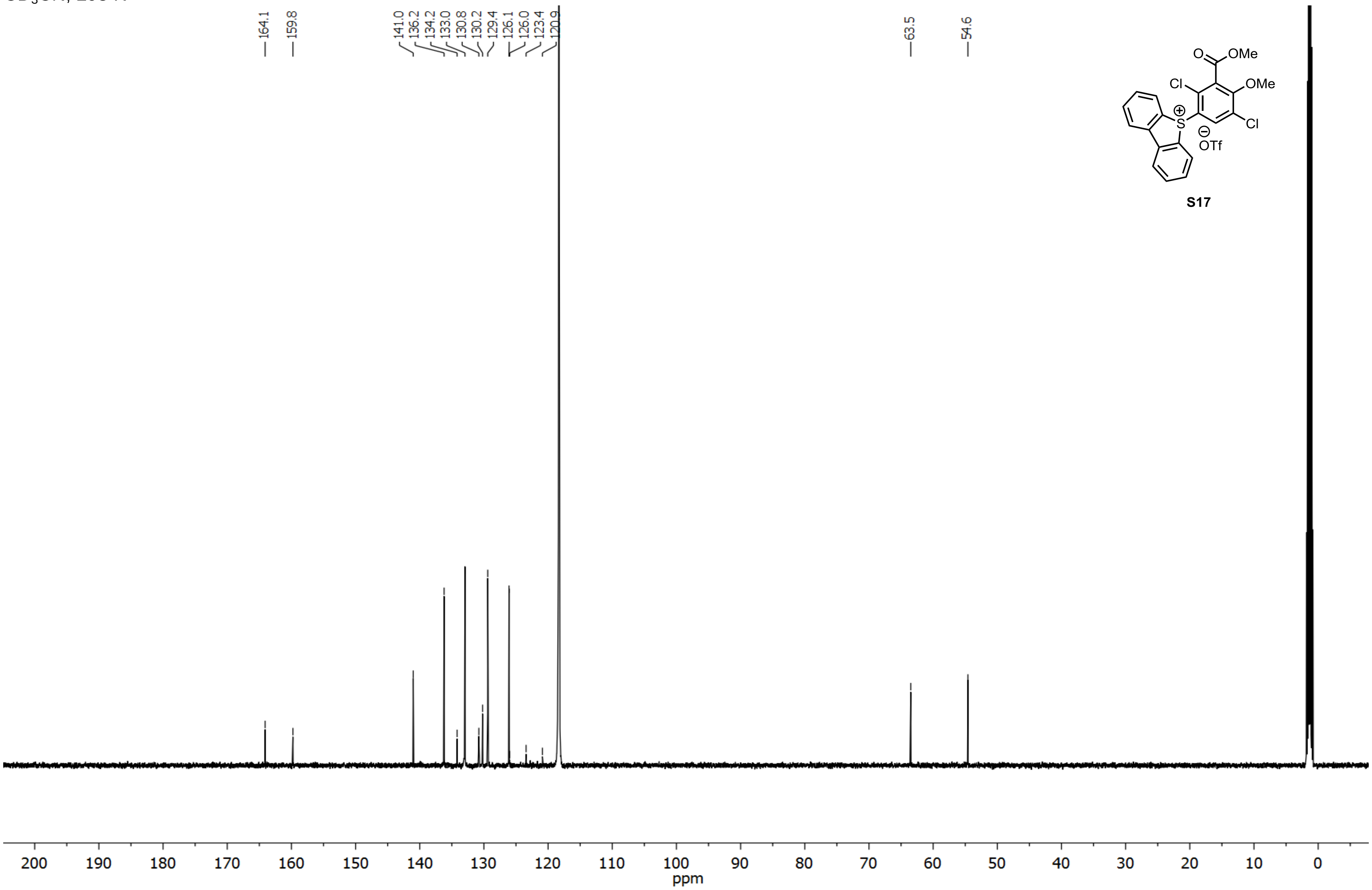

S17

^{19}F NMR of dicamba methylester-derived dibenzothiophenium salt S17

CD_3CN, 298 K

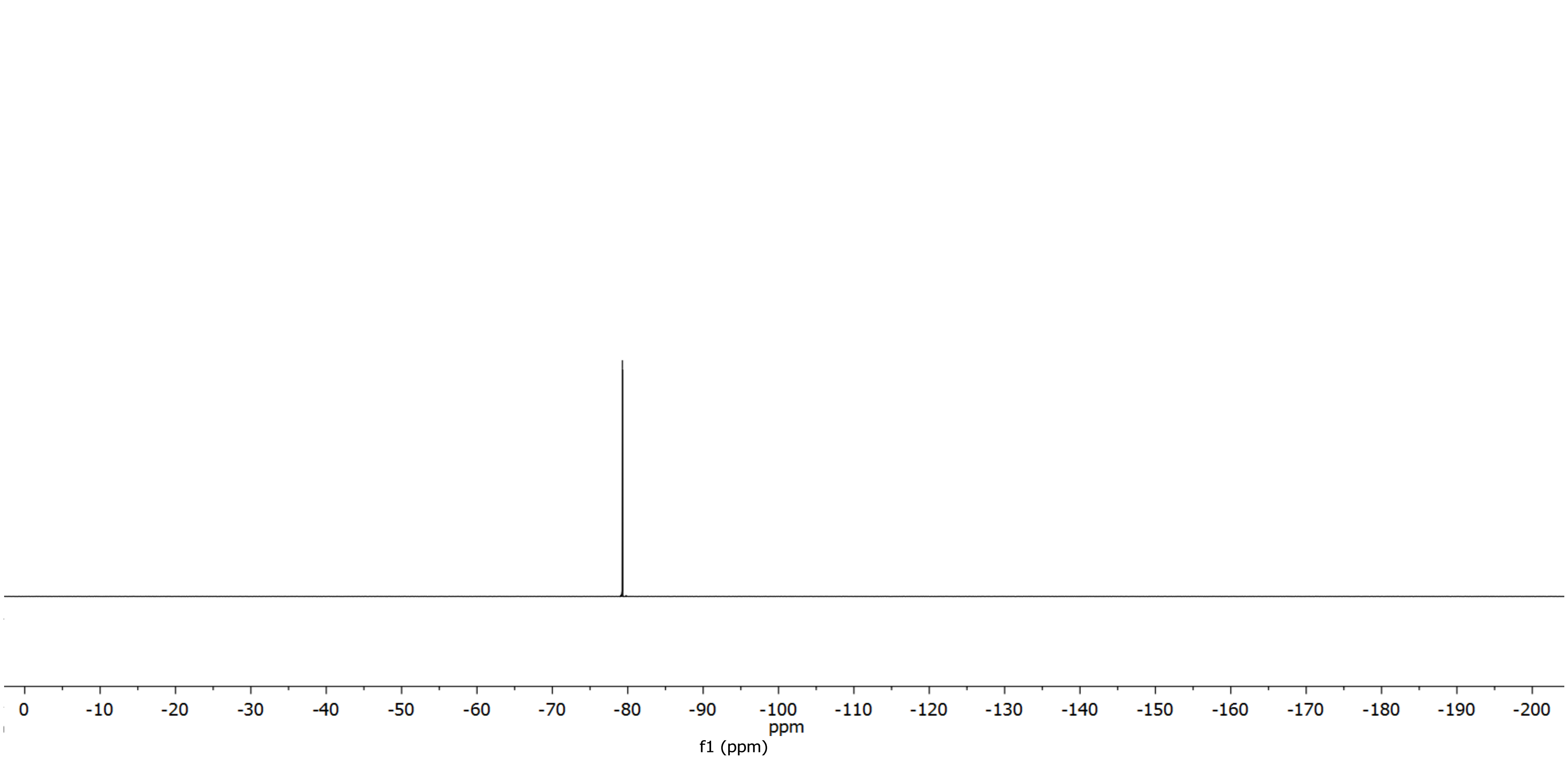

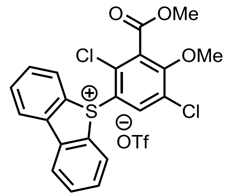

S17

^{1}H NMR of clofibrate-derived dibenzothiophenium salt S18

CD$_3$CN, 298 K

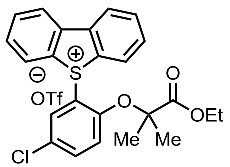

S18

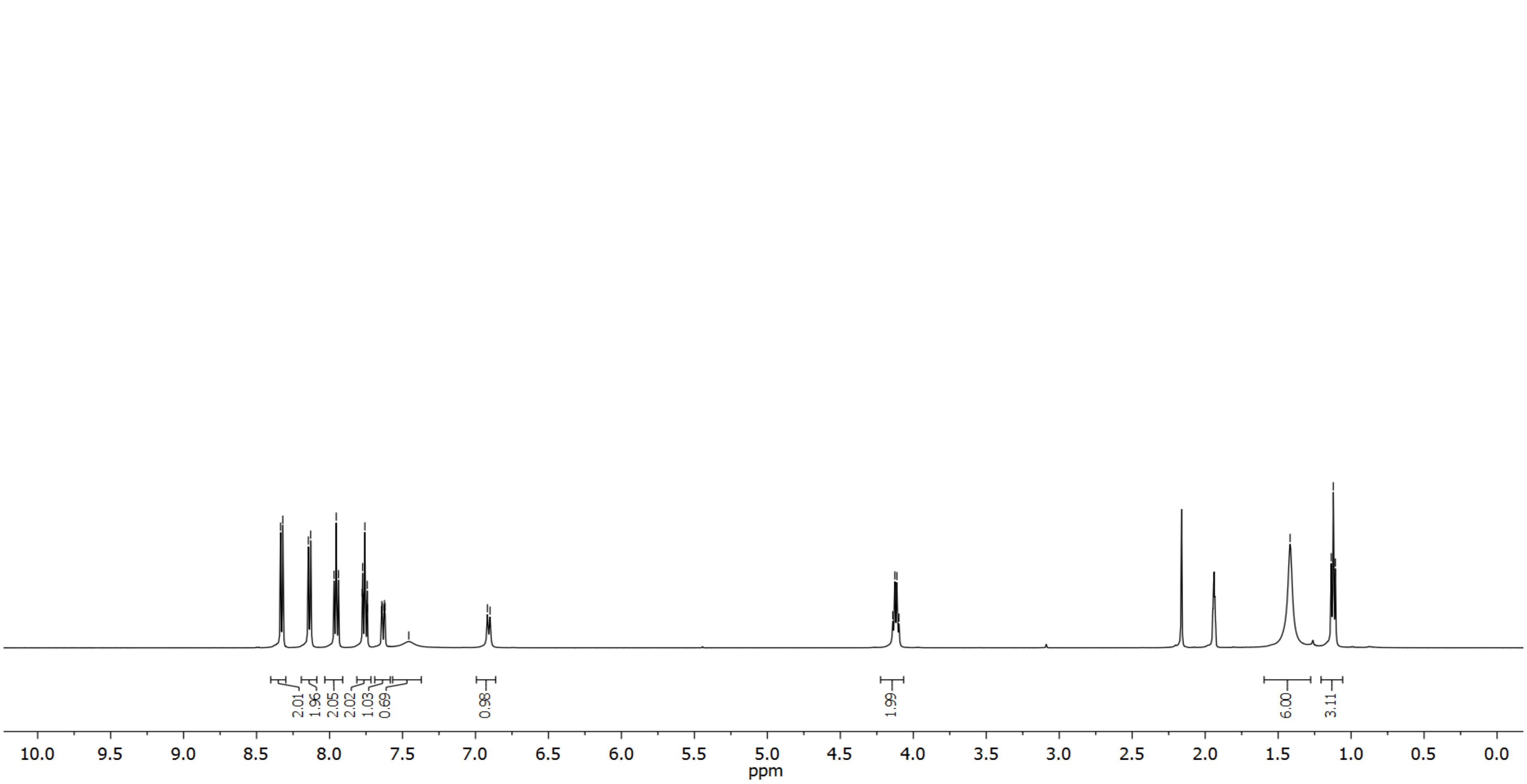

^{13}C NMR of clofibrate-derived dibenzothiophenium salt S18

CD_3CN, 298 K

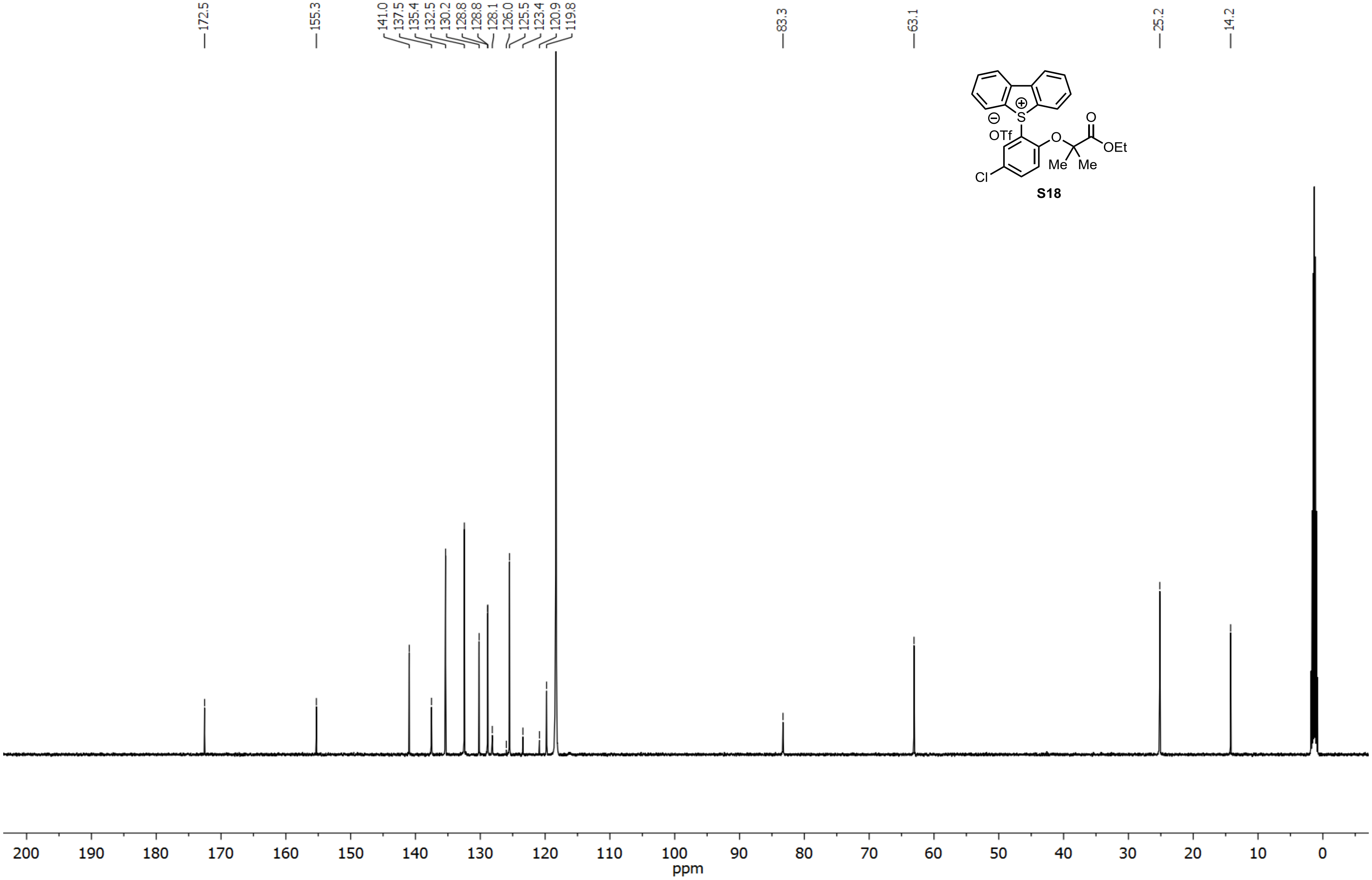

^{19}F NMR of clofibrate-derived dibenzothiophenium salt S18

CD_3CN, 298 K

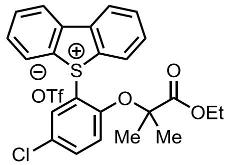

S18

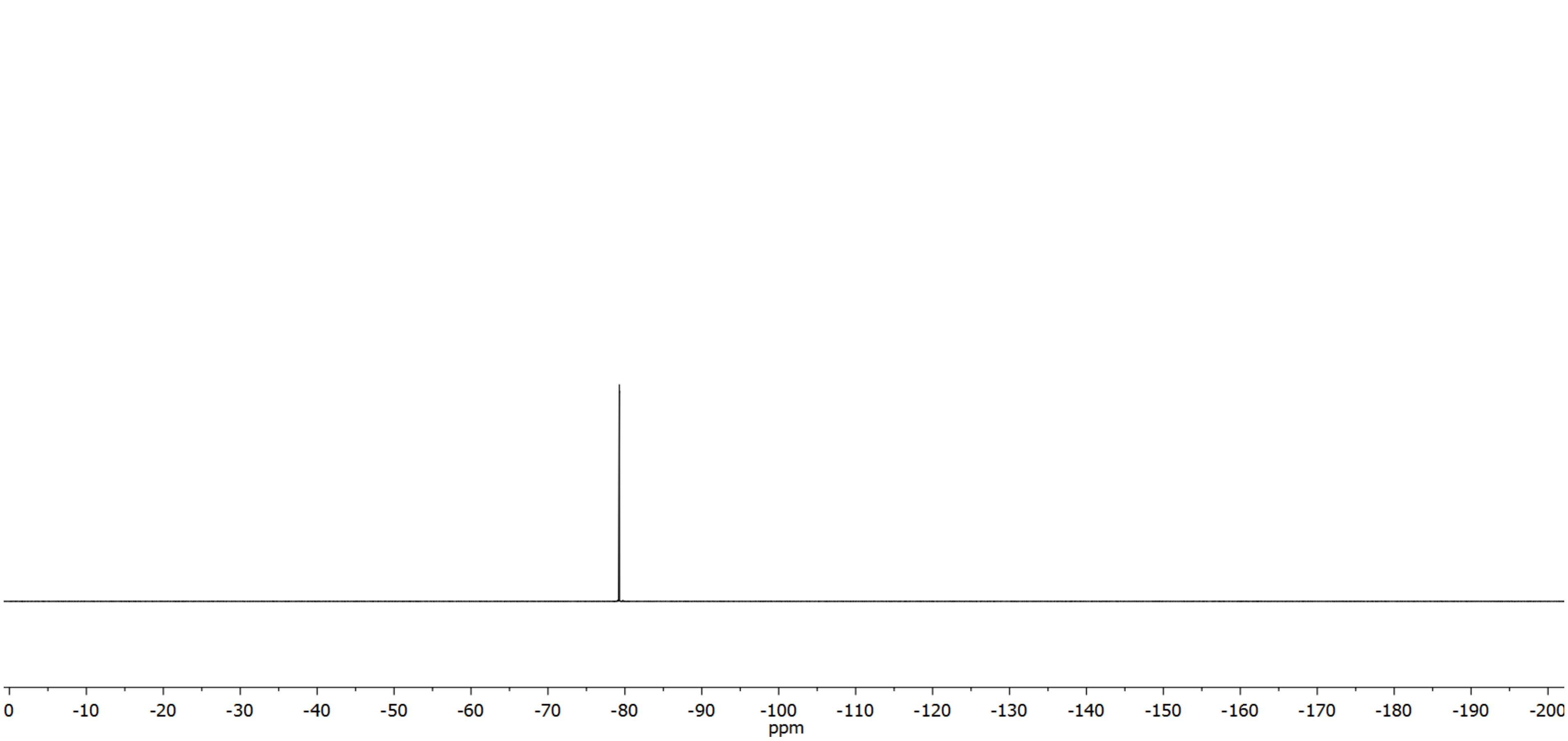

^{1}H NMR of bifonazole-derived dibenzothiophenium salt S19

CD_3CN, 298 K

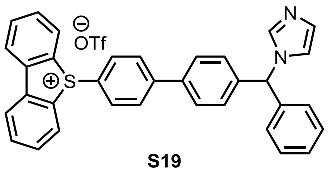

S19

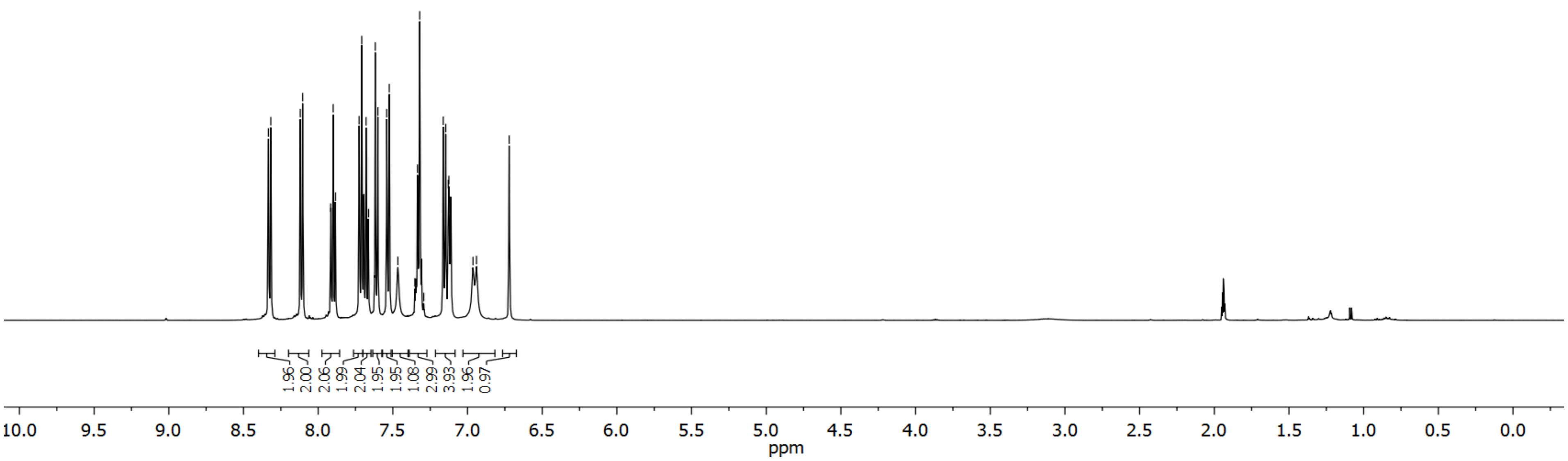

^{13}C NMR of bifonazole-derived dibenzothiophenium salt S19

CD_3CN, 298 K

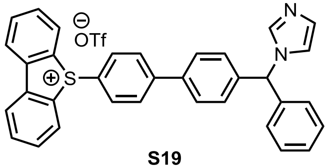

S19

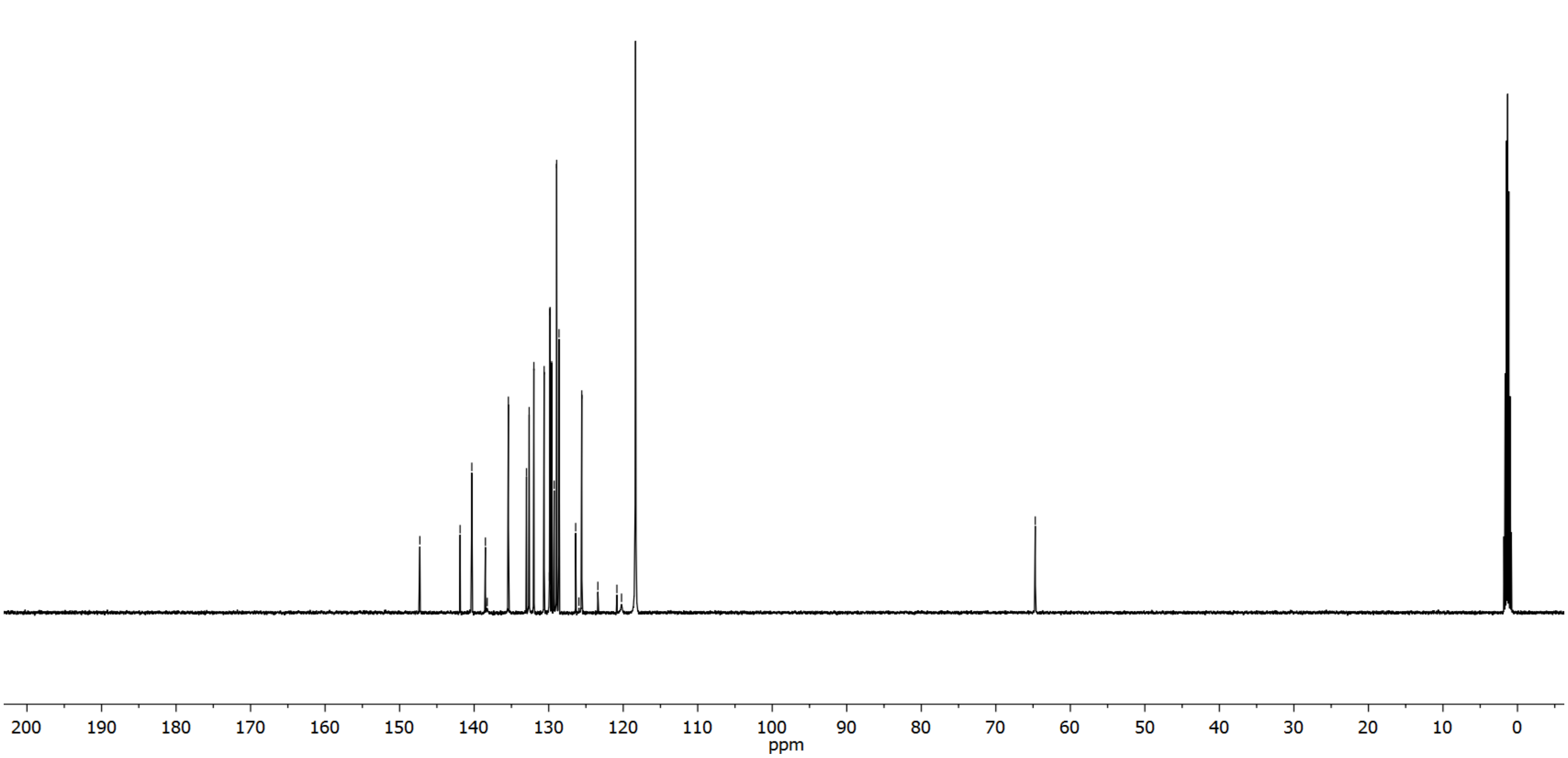

^{19}F NMR of bifonazole-derived dibenzothiophenium salt S19

CD_3CN, 298 K

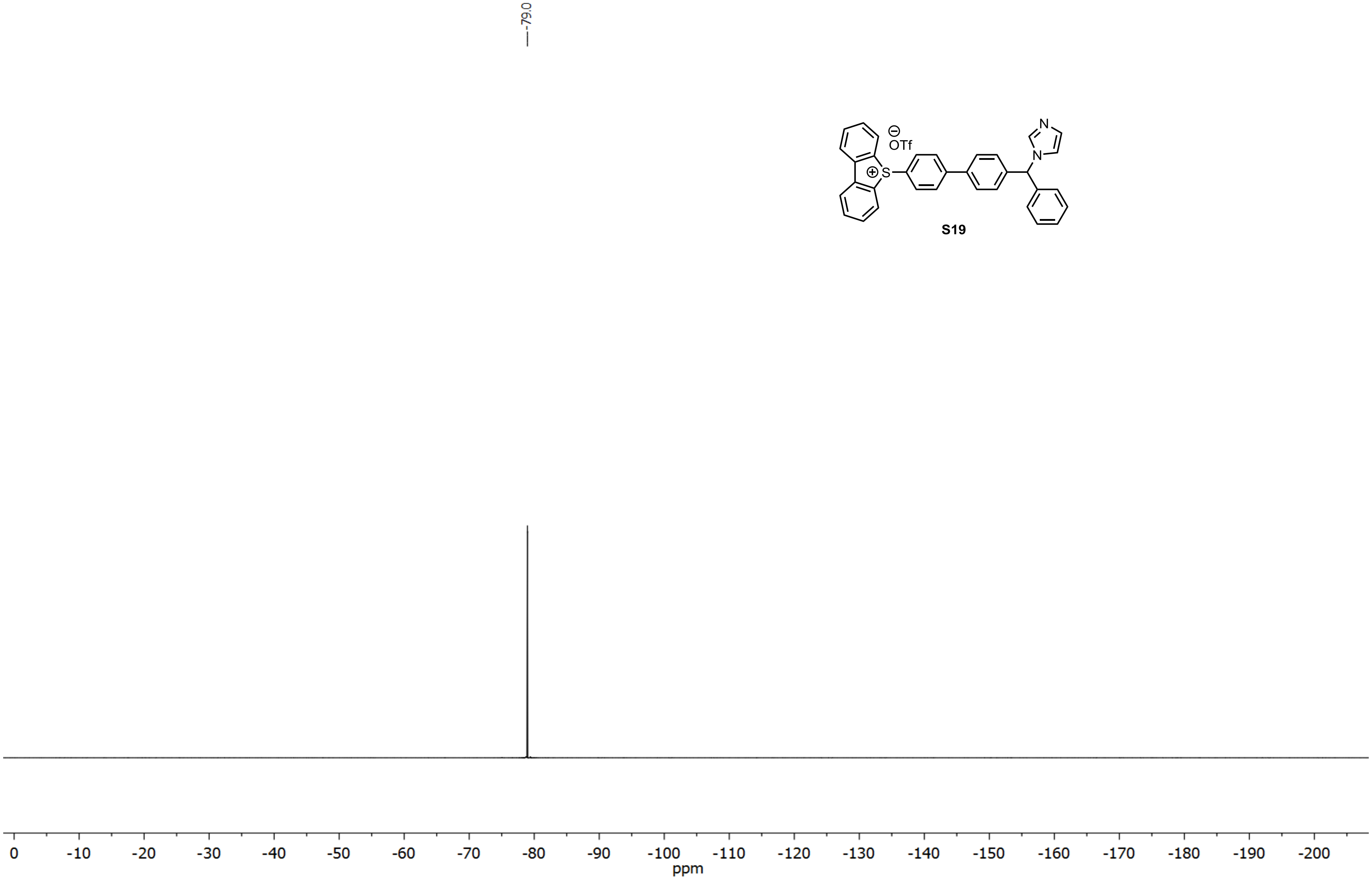

^{1}H NMR of pyriproxyfen-derived 2,8-dimethoxydibenzothiophenium salt S20

CD_3CN, 298 K

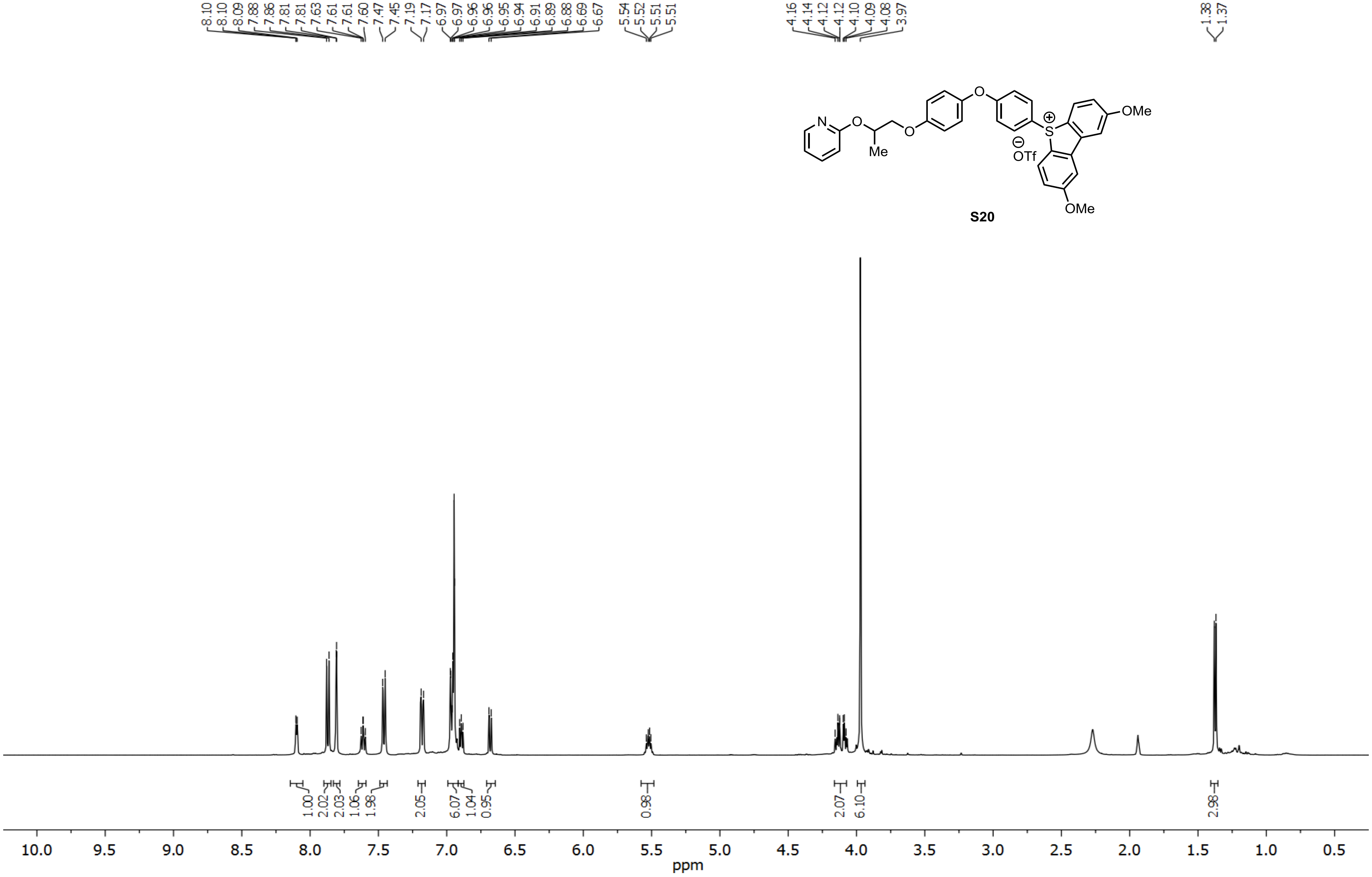

^{13}C NMR of pyriproxyfen-derived 2,8-dimethoxydibenzothiophenium salt S20

CD_3CN, 298 K

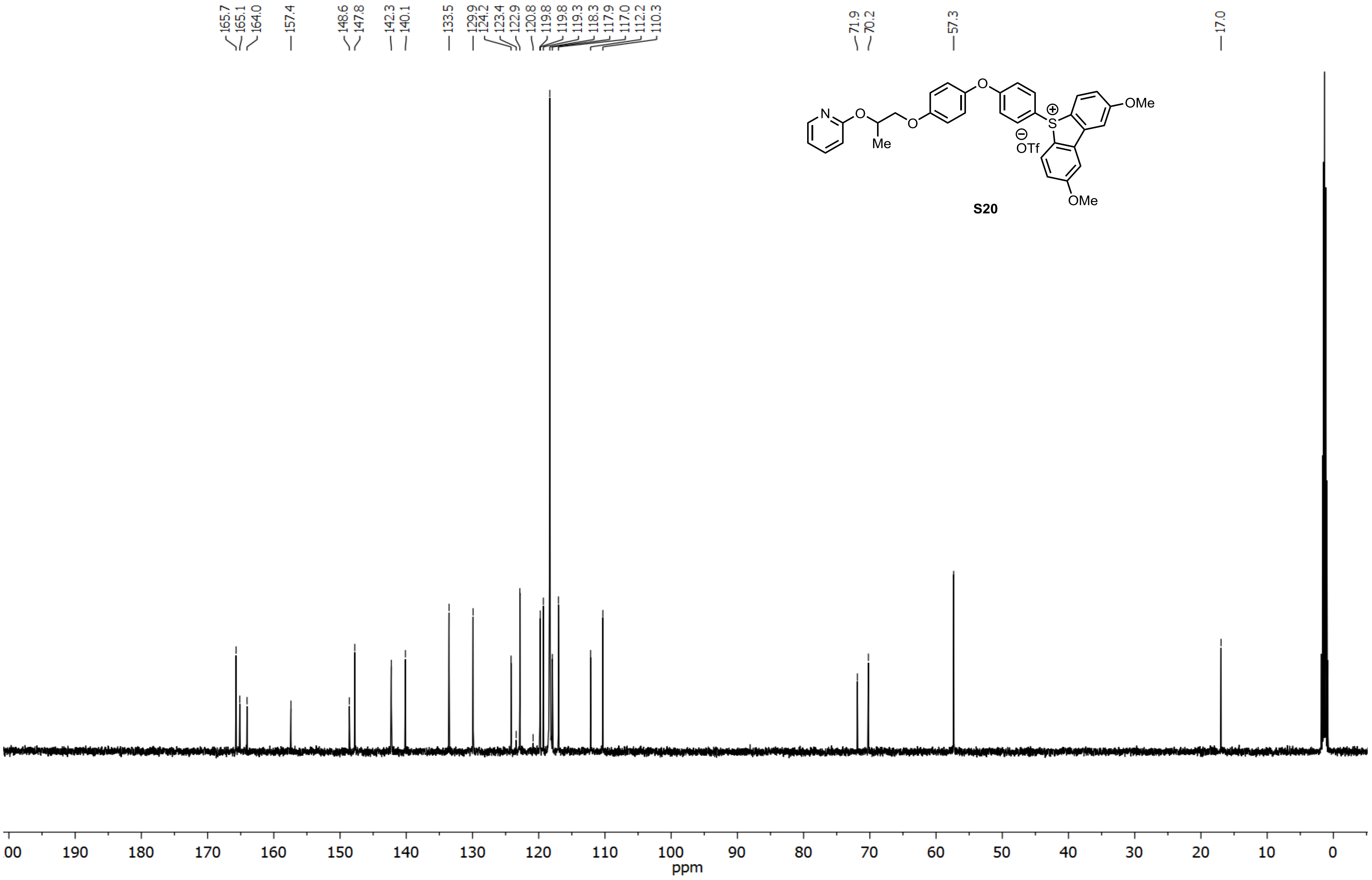

^{19}F NMR of pyriproxyfen-derived 2,8-dimethoxydibenzothiophenium salt S20

CD_3CN, 298 K

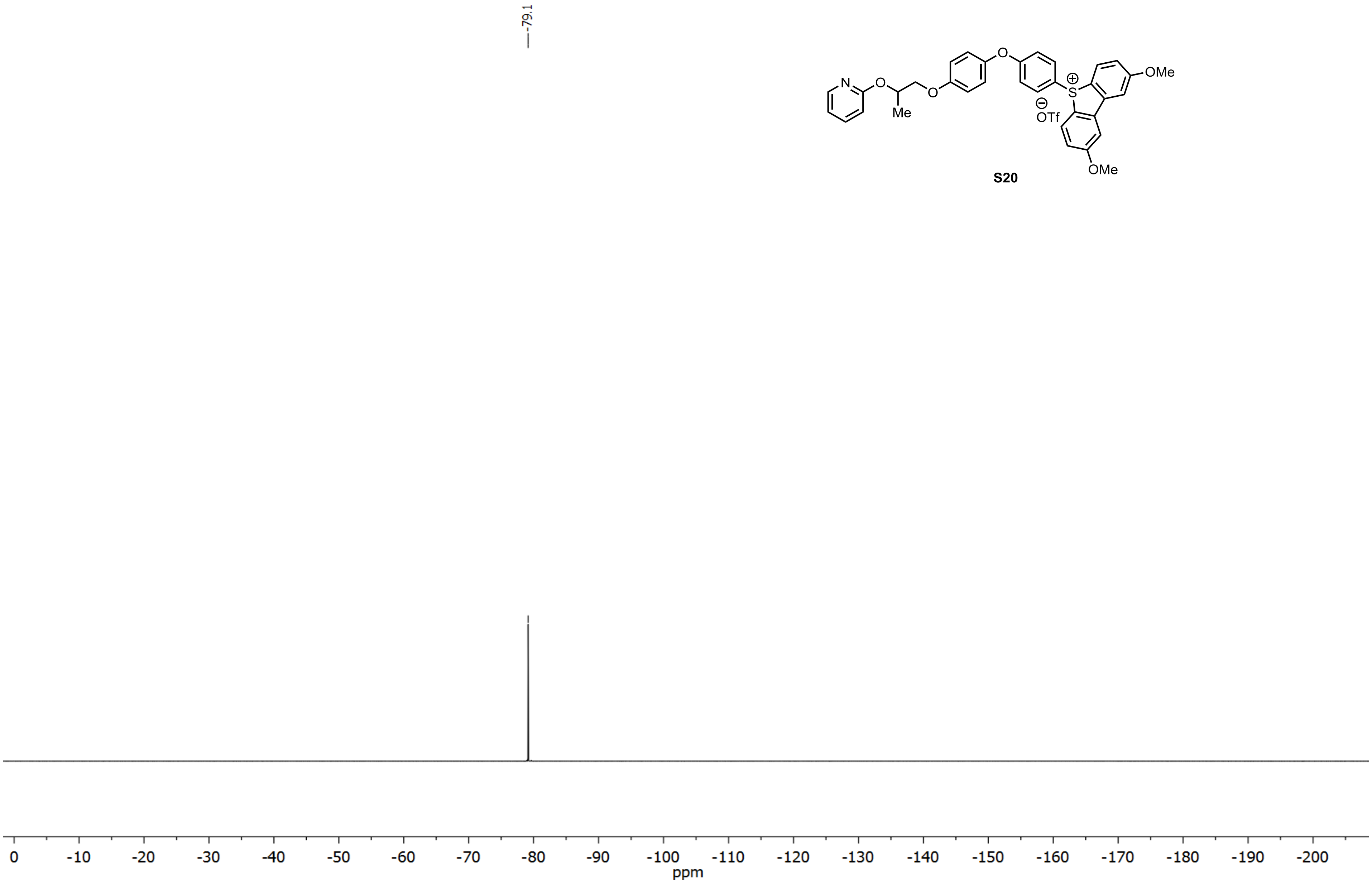

^{1}H NMR of fluorobenzene-derived dibenzothiophenium salt S21

CD$_3$CN, 298 K

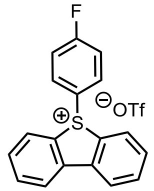

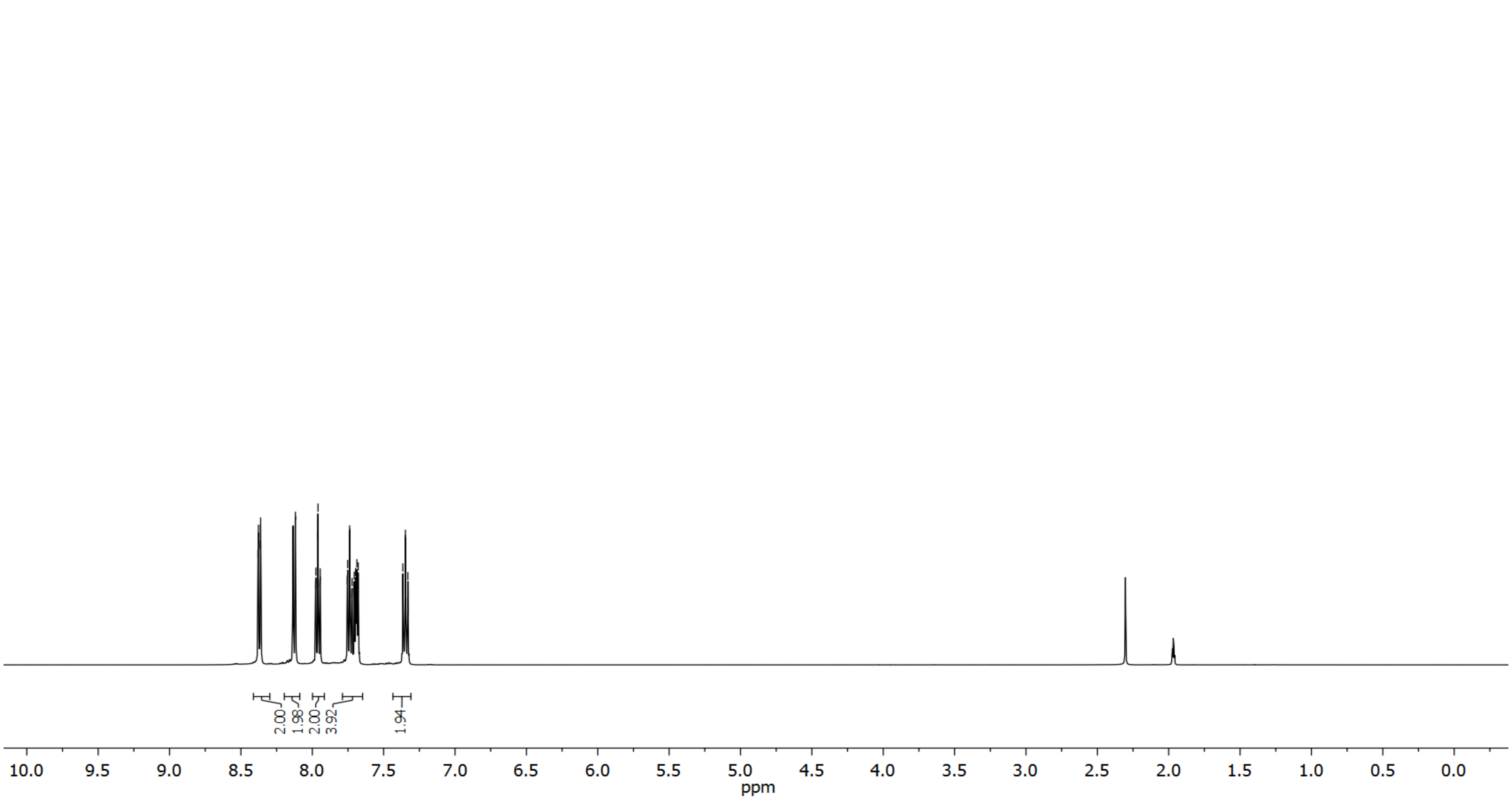

^{13}C NMR of fluorobenzene-derived dibenzothiophenium salt S21

CD_3CN, 298 K

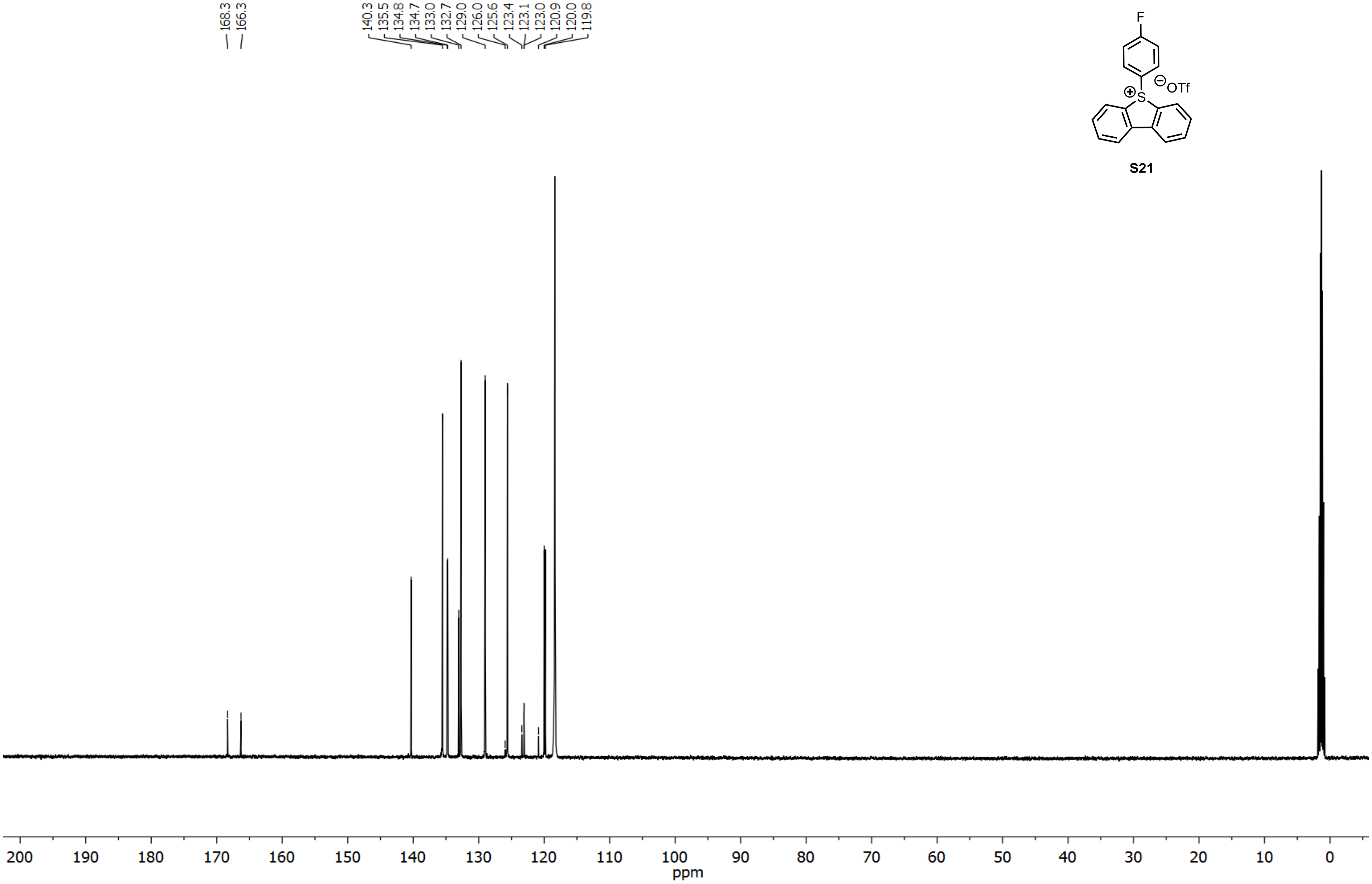

^{19}F NMR of fluorobenzene-derived dibenzothiophenium salt S21

CD_3CN, 298 K

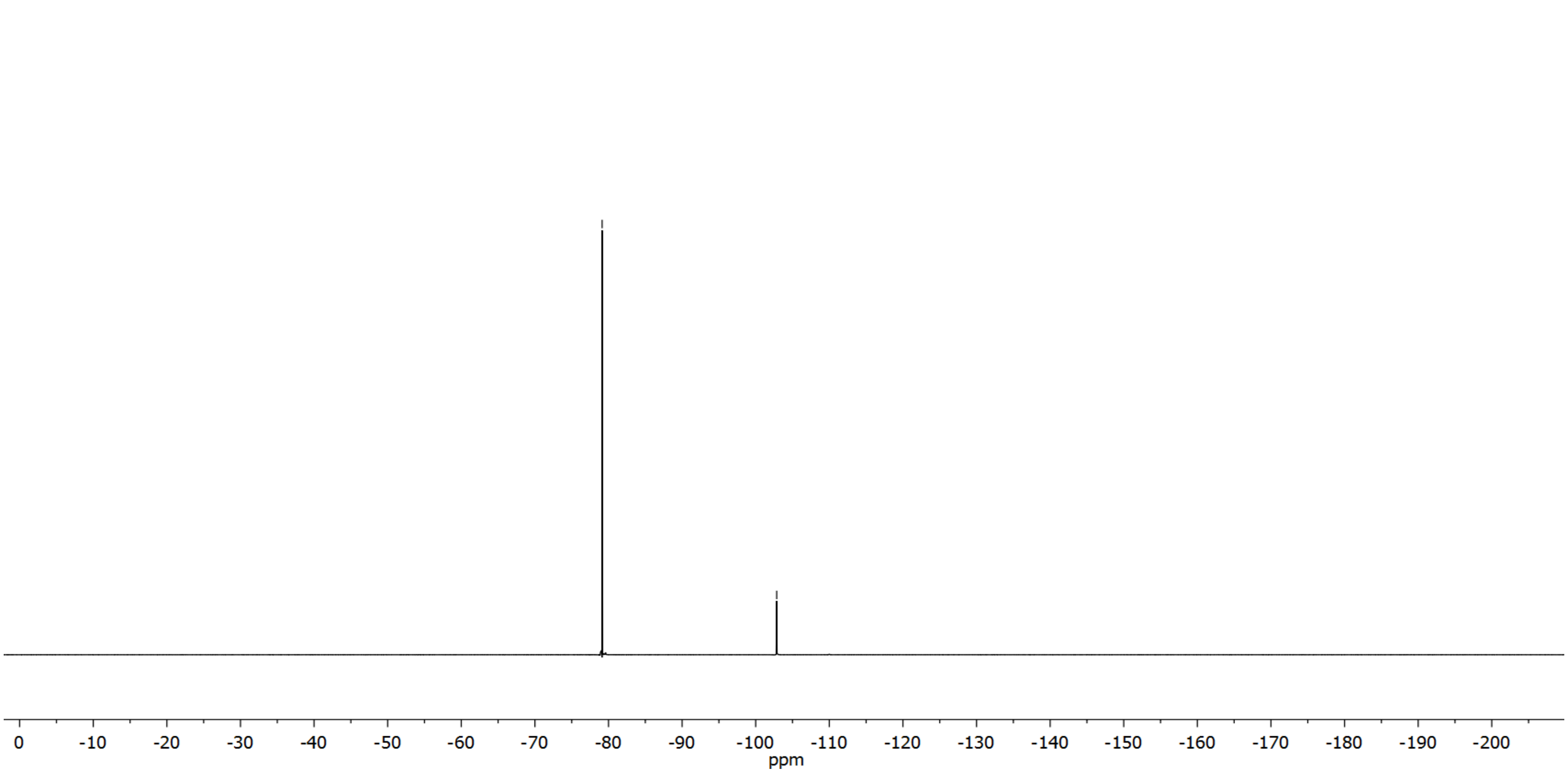

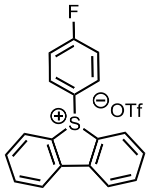

S21

^{1}H NMR of chlorobenzene-derived dibenzothiophenium salt S22

CD$_3$CN, 298 K

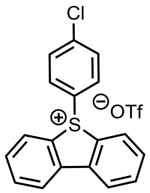

S22

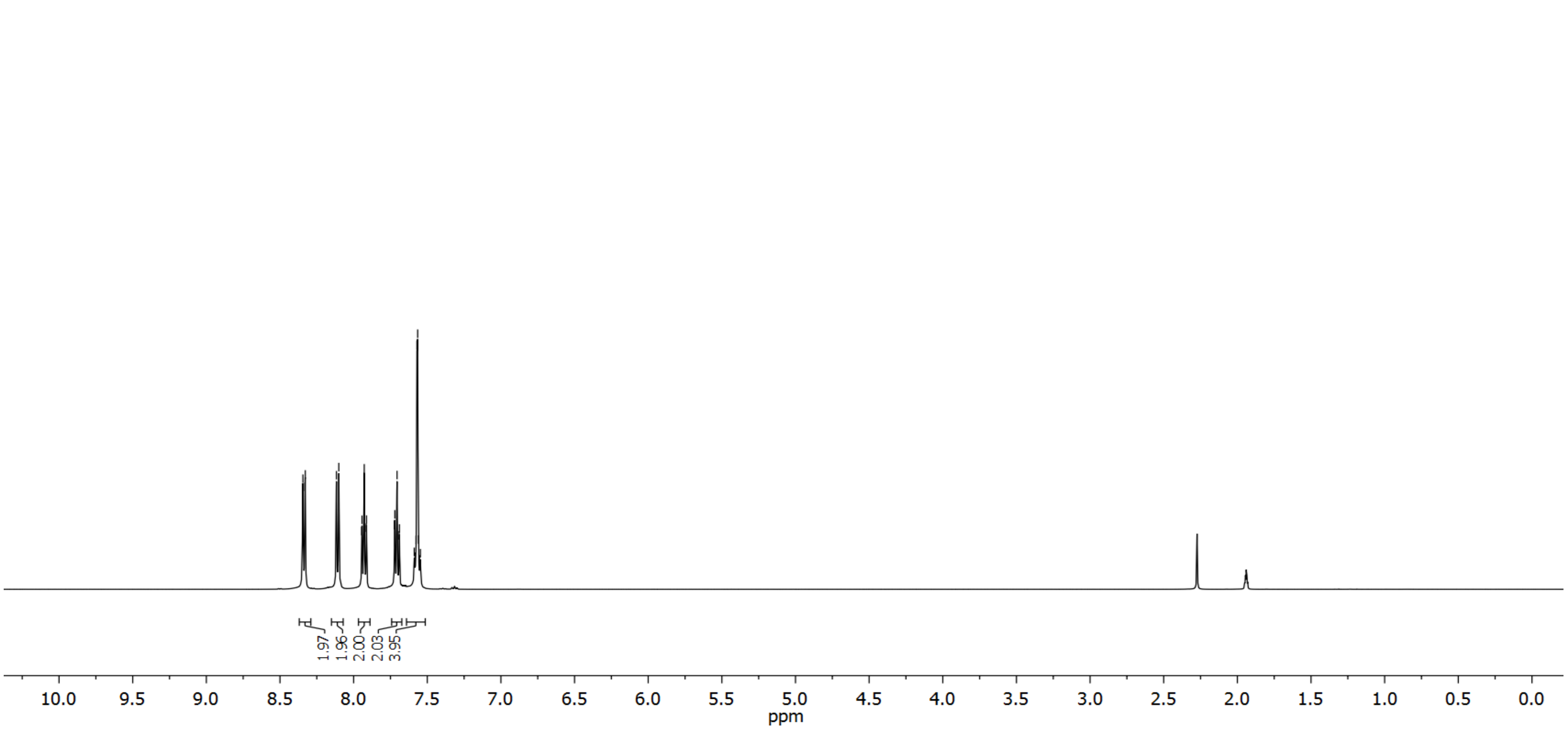

^{13}C NMR of chlorobenzene-derived dibenzothiophenium salt S22

CD_3CN, 298 K

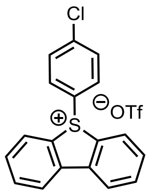

S22

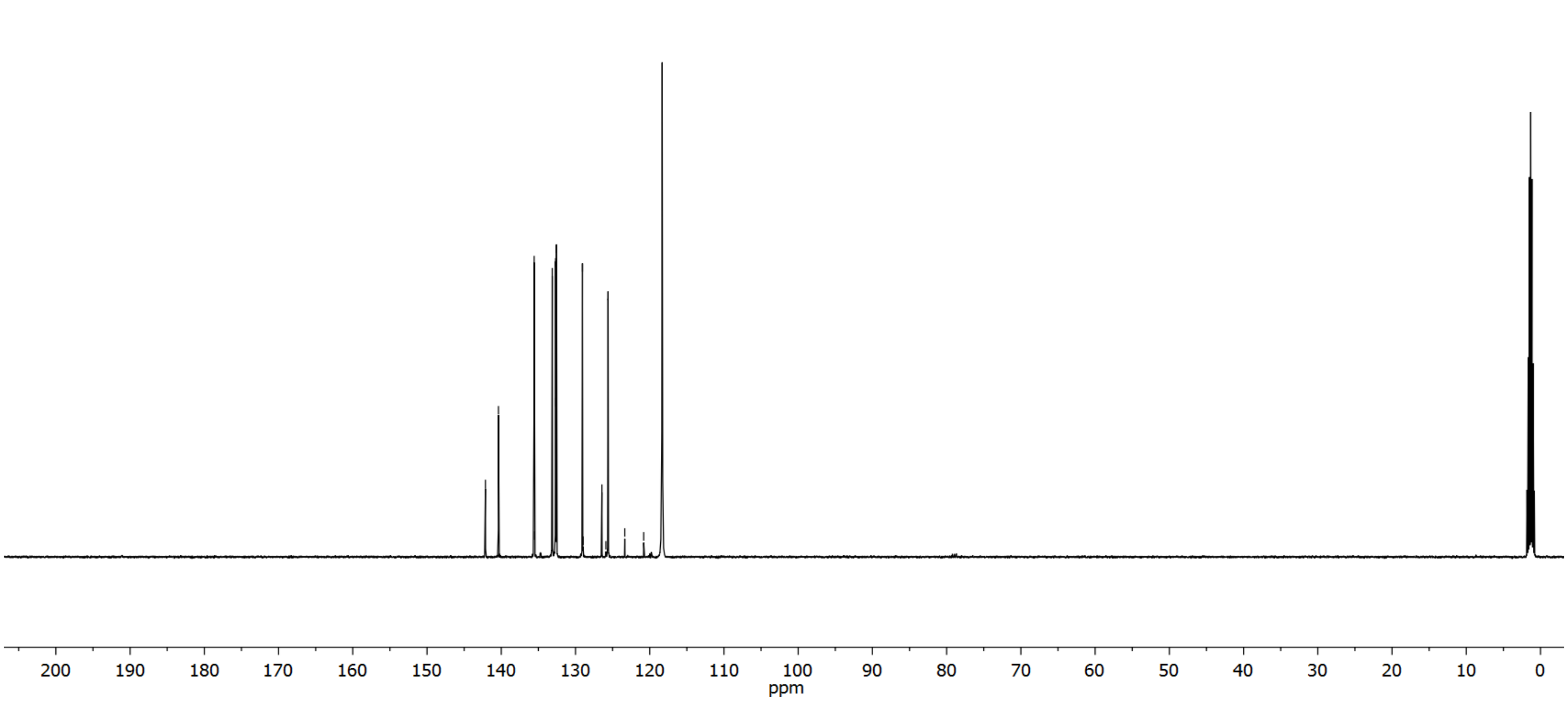

^{19}F NMR of chlorobenzene-derived dibenzothiophenium salt S22

CD_3CN, 298 K

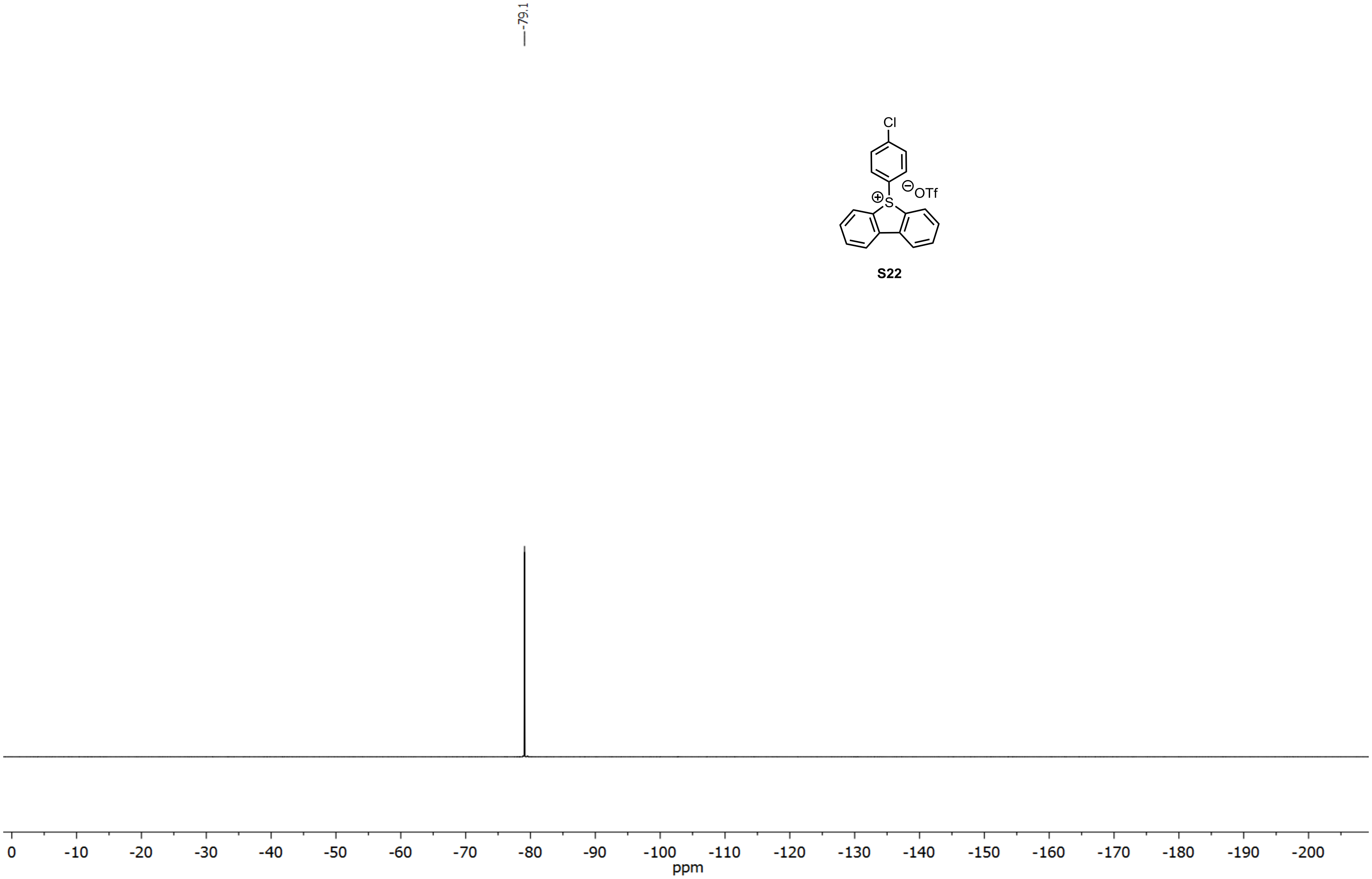

^{1}H NMR of iodobenzene-derived dibenzothiophenium salt S23

DMSO-d_6, 298 K

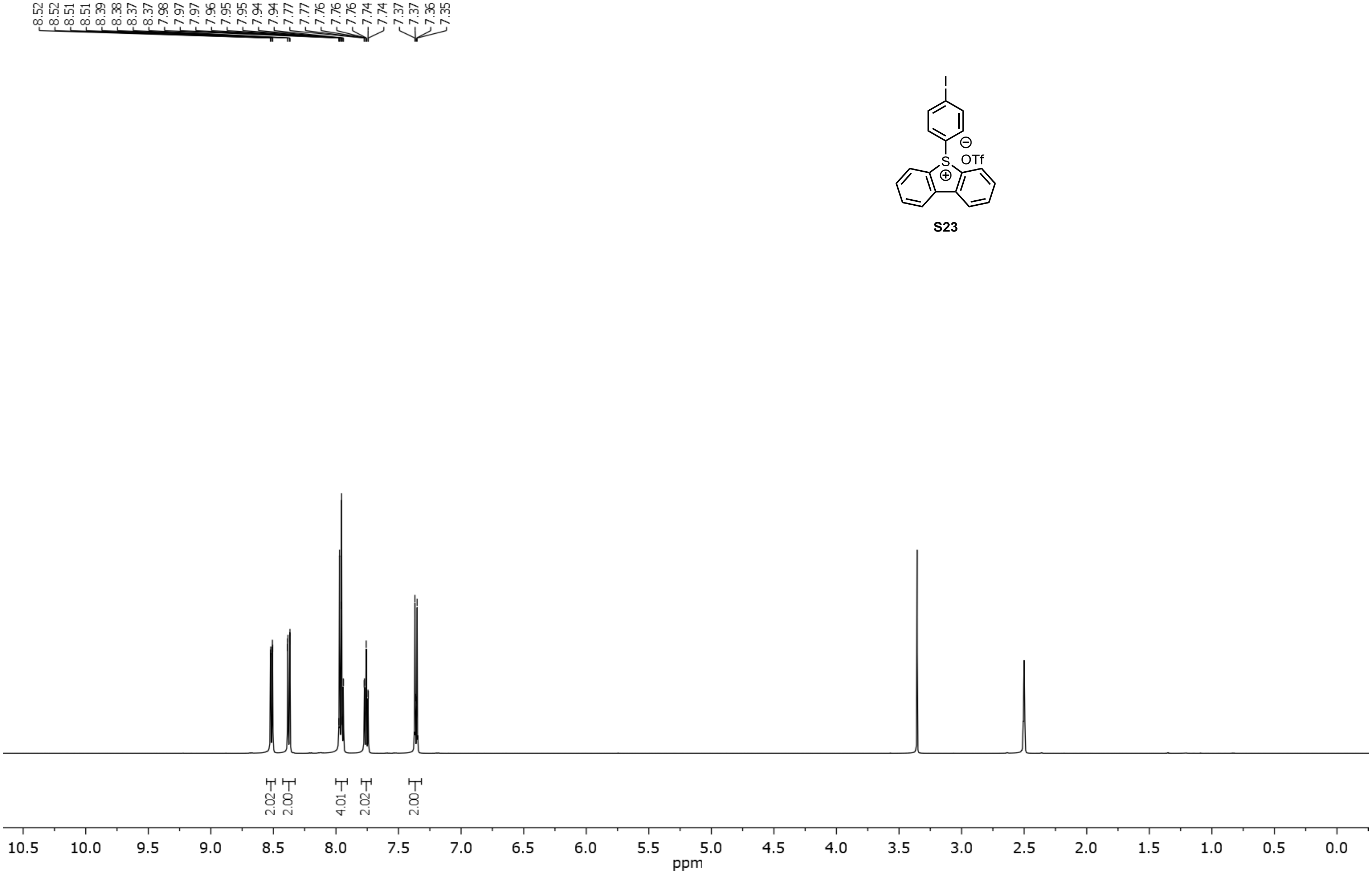

^{13}C NMR of iodobenzene-derived dibenzothiophenium salt S23

DMSO-d$_6$, 298 K

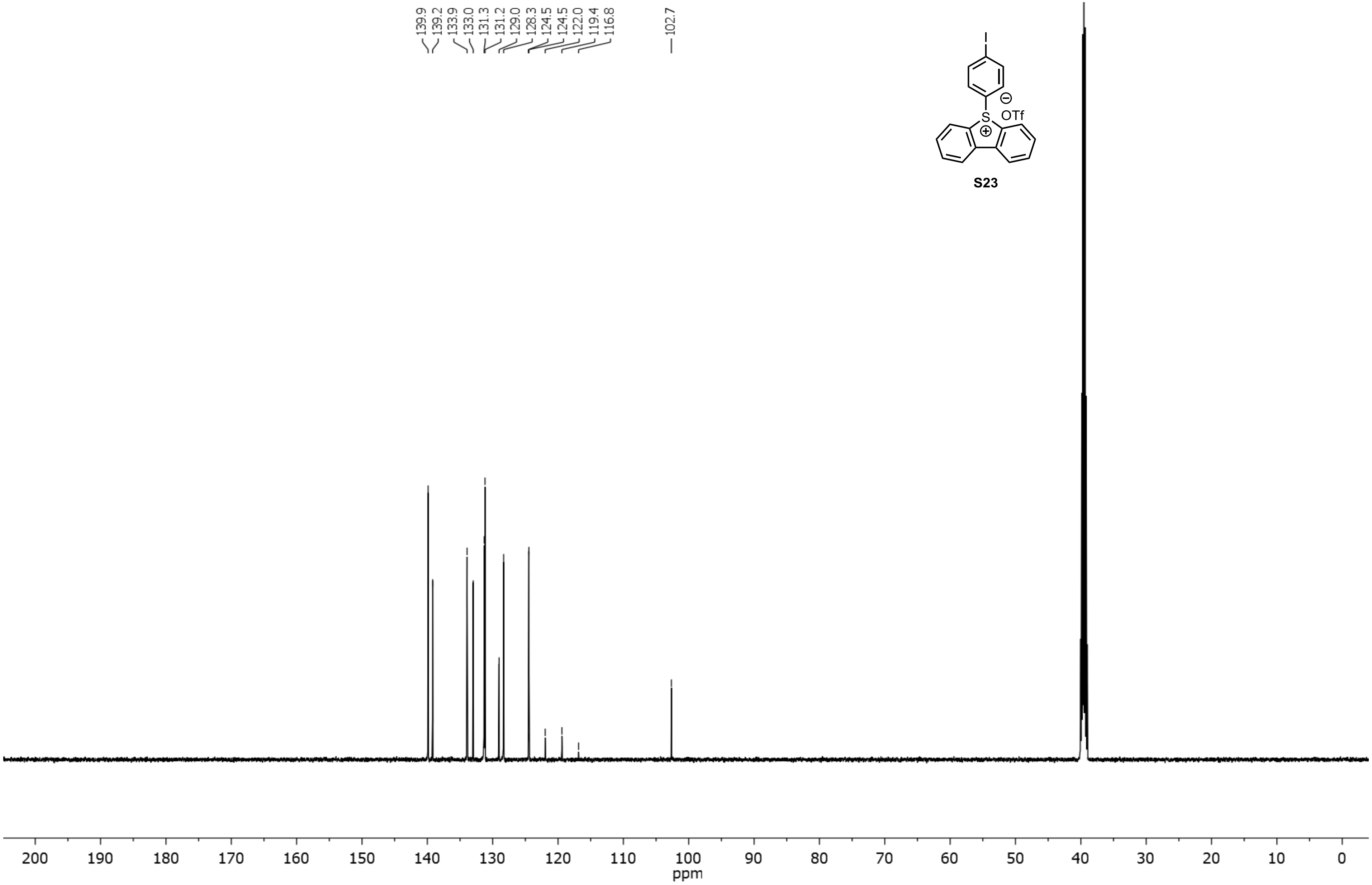

^{19}F NMR of iodobenzene-derived dibenzothiophenium salt S23

DMSO-d_6, 298 K

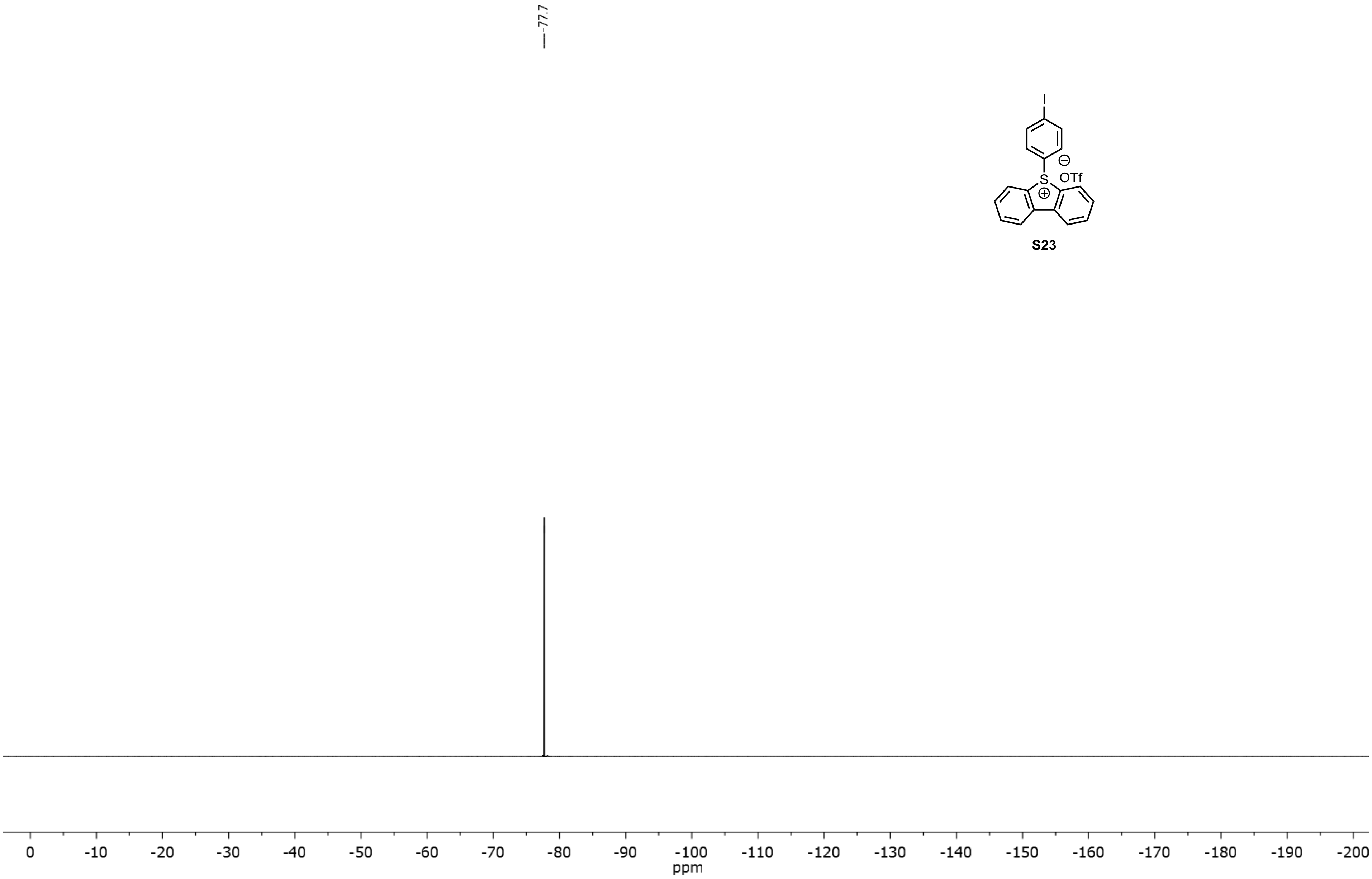

^{1}H NMR of 4-bromodiphenylether-derived 2,8-dimethoxydibenzothiophenium salt S24

CD_3CN, 298 K

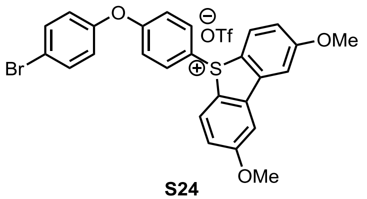

S24

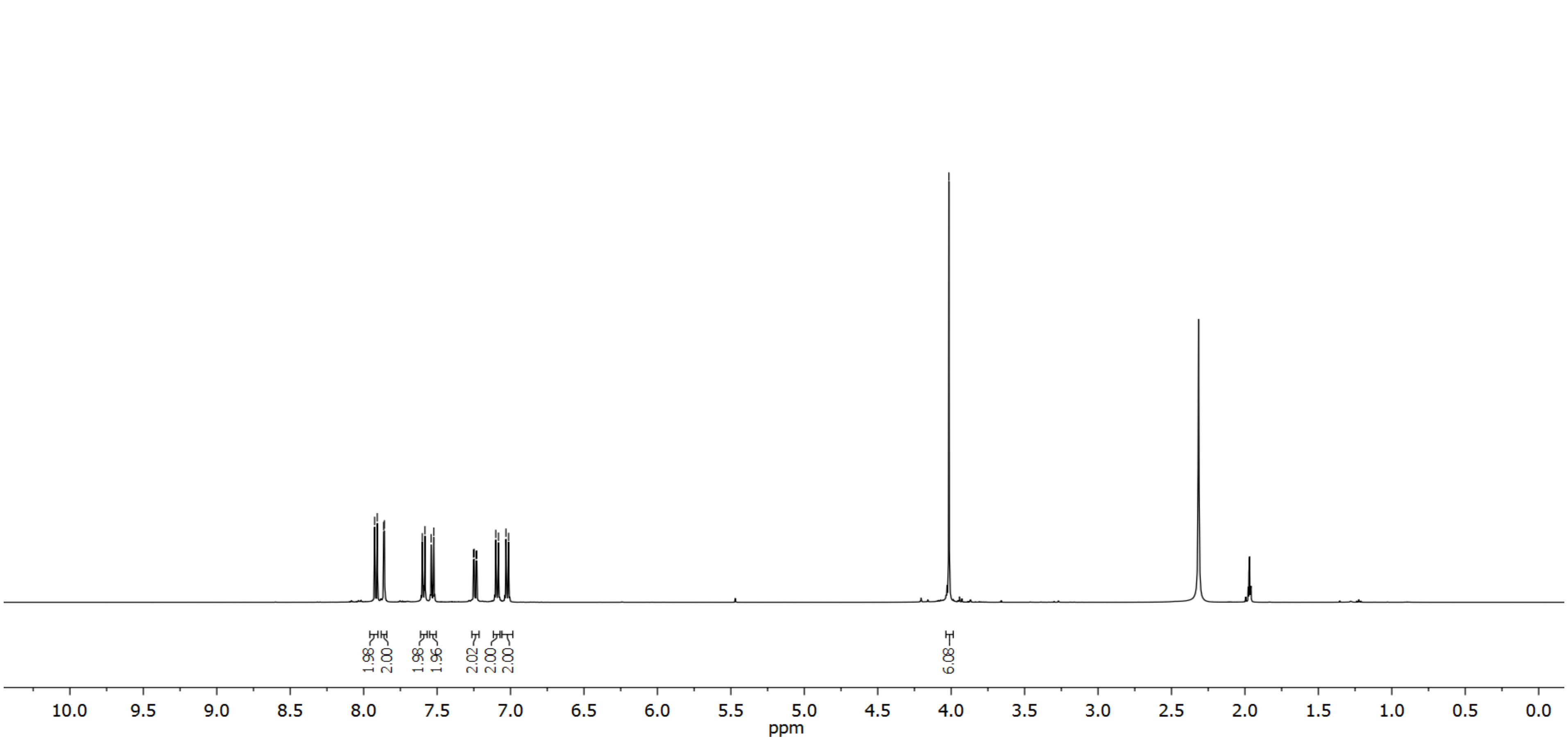

^{13}C NMR of 4-bromodiphenylether-derived 2,8-dimethoxydibenzothiophenium salt S24

CD_3CN, 298 K

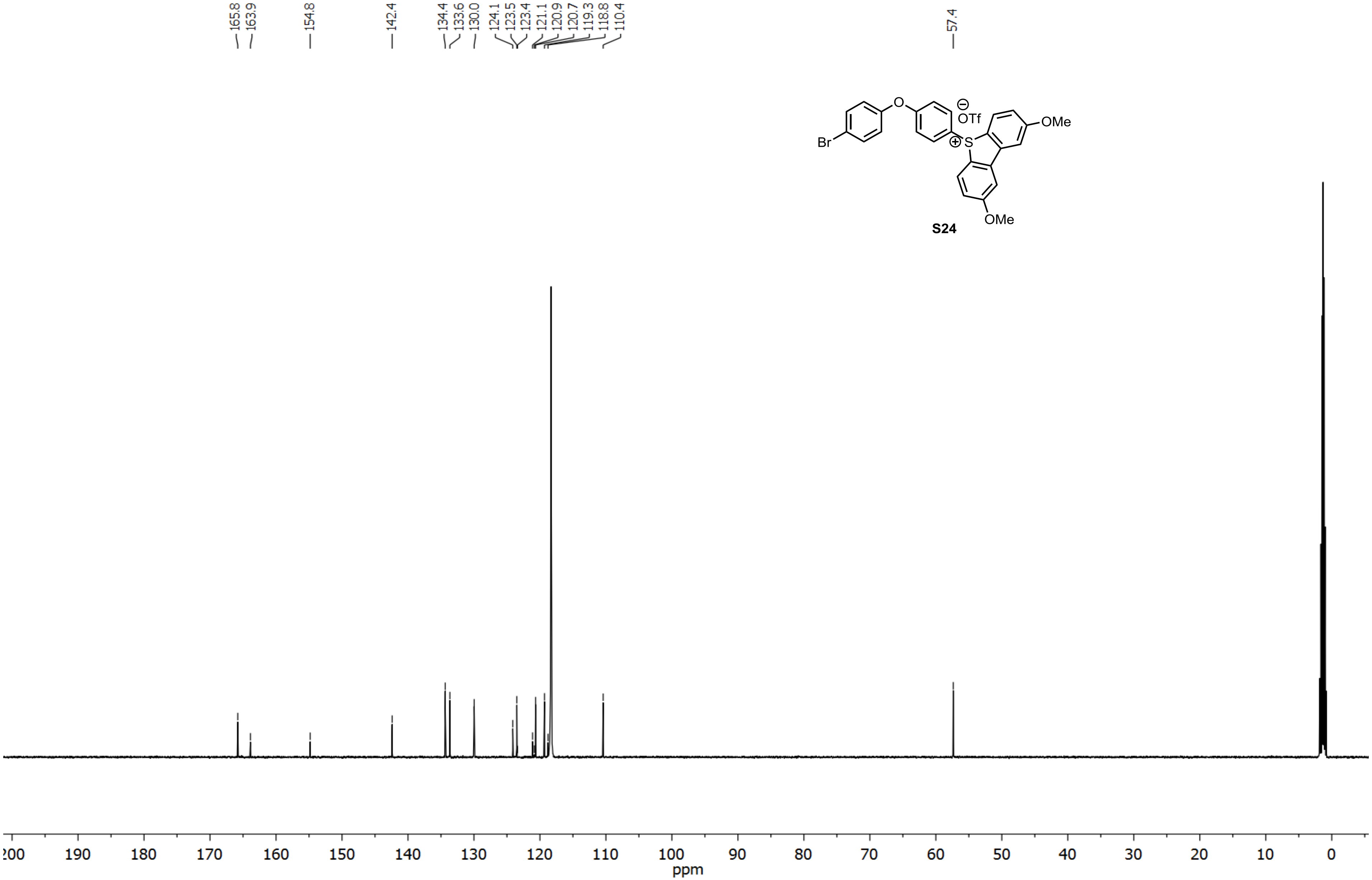

^{19}F NMR of 4-bromodiphenylether-derived 2,8-dimethoxydibenzothiophenium salt S24

CD_3CN, 298 K

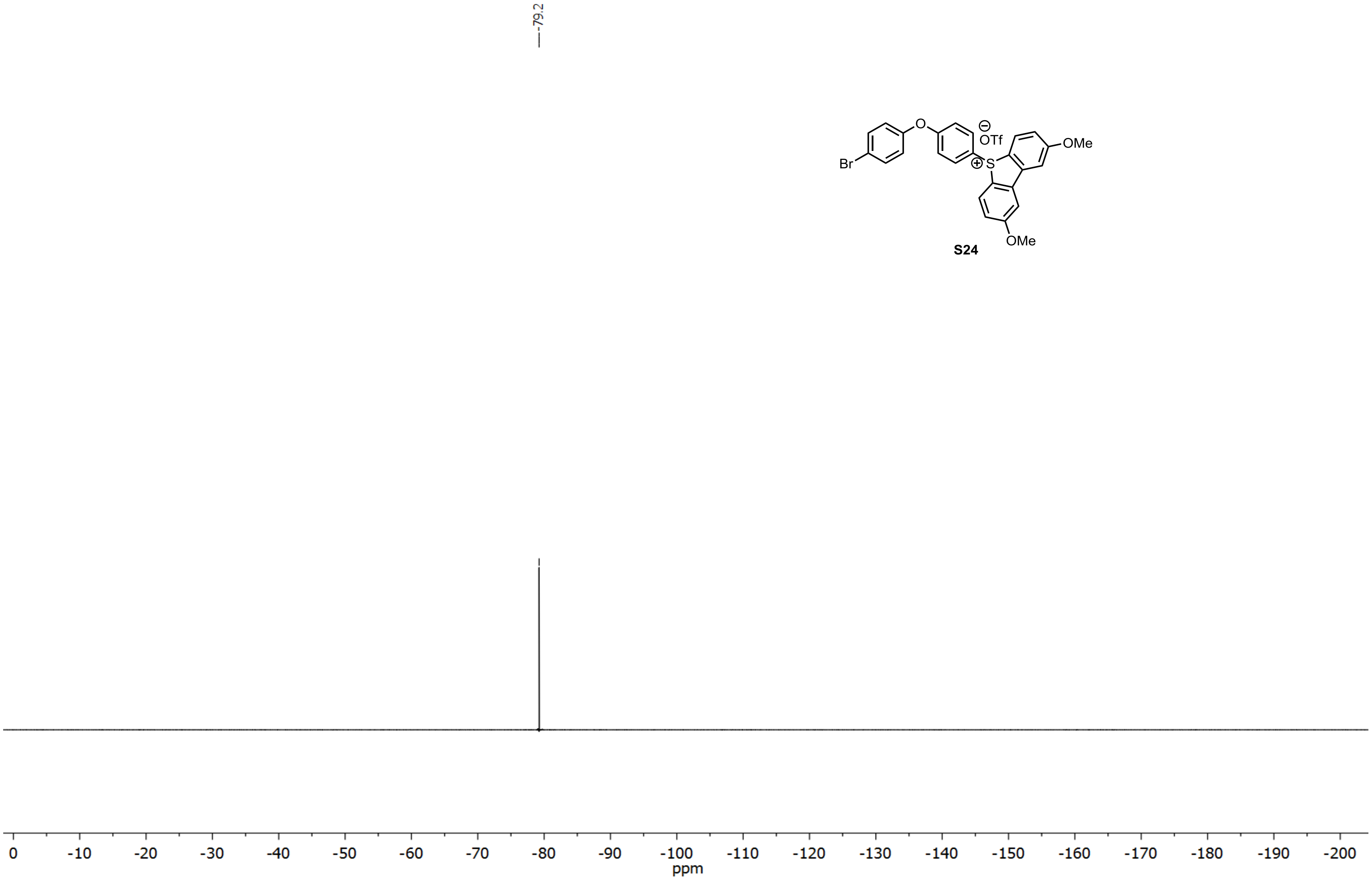

^{1}H NMR of 1,3-dimethylquinazoline-2,4(1*H*,3*H*)-dione-derived 3,7-di-*tert*-butyldibenzothiophenium salt S25

CD_3CN, 298 K

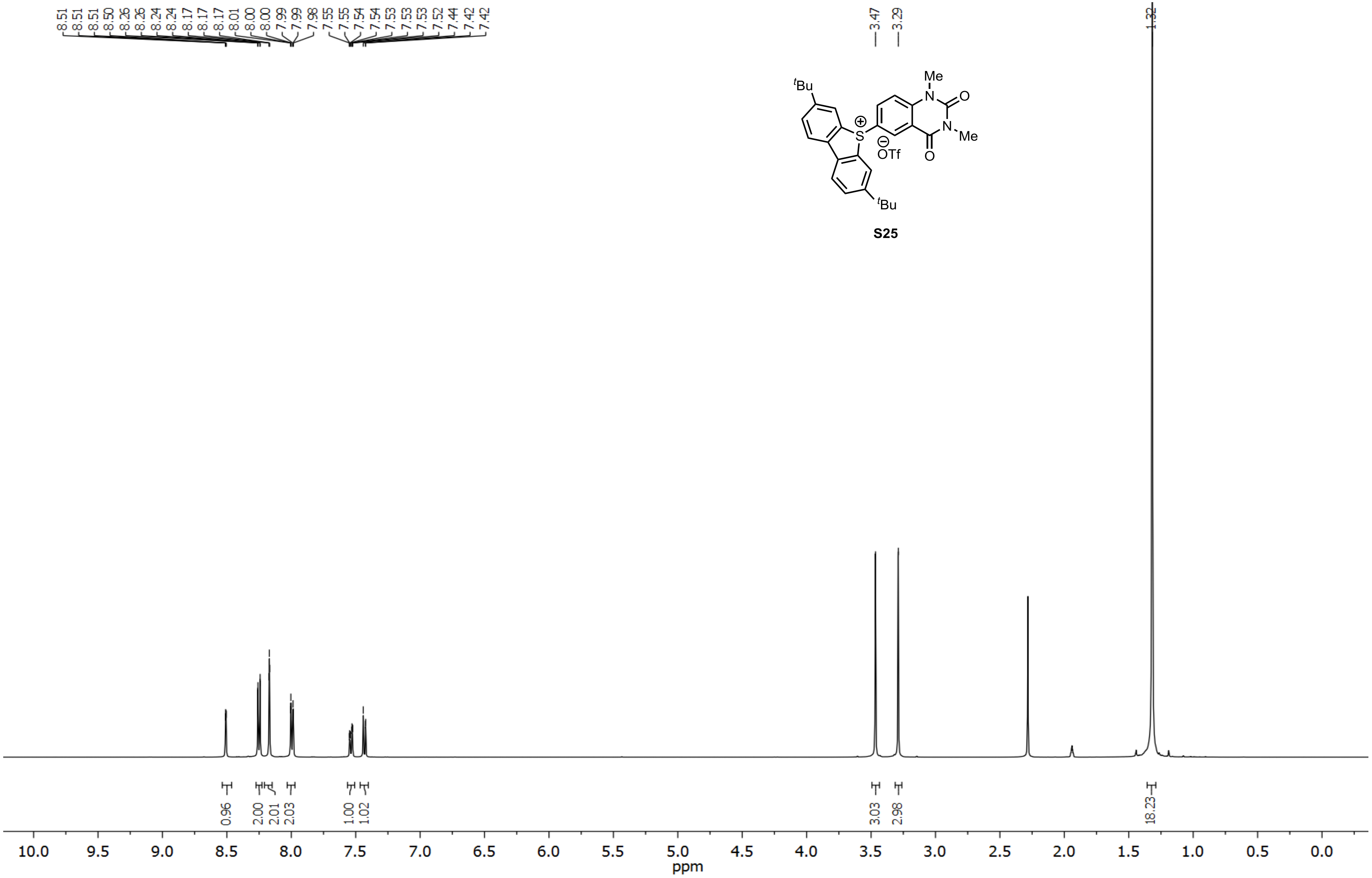

^{13}C NMR of 1,3-dimethylquinazoline-2,4(1*H*,3*H*)-dione-derived 3,7-di-*tert*-butyldibenzothiophenium salt S25

CD_3CN, 298 K

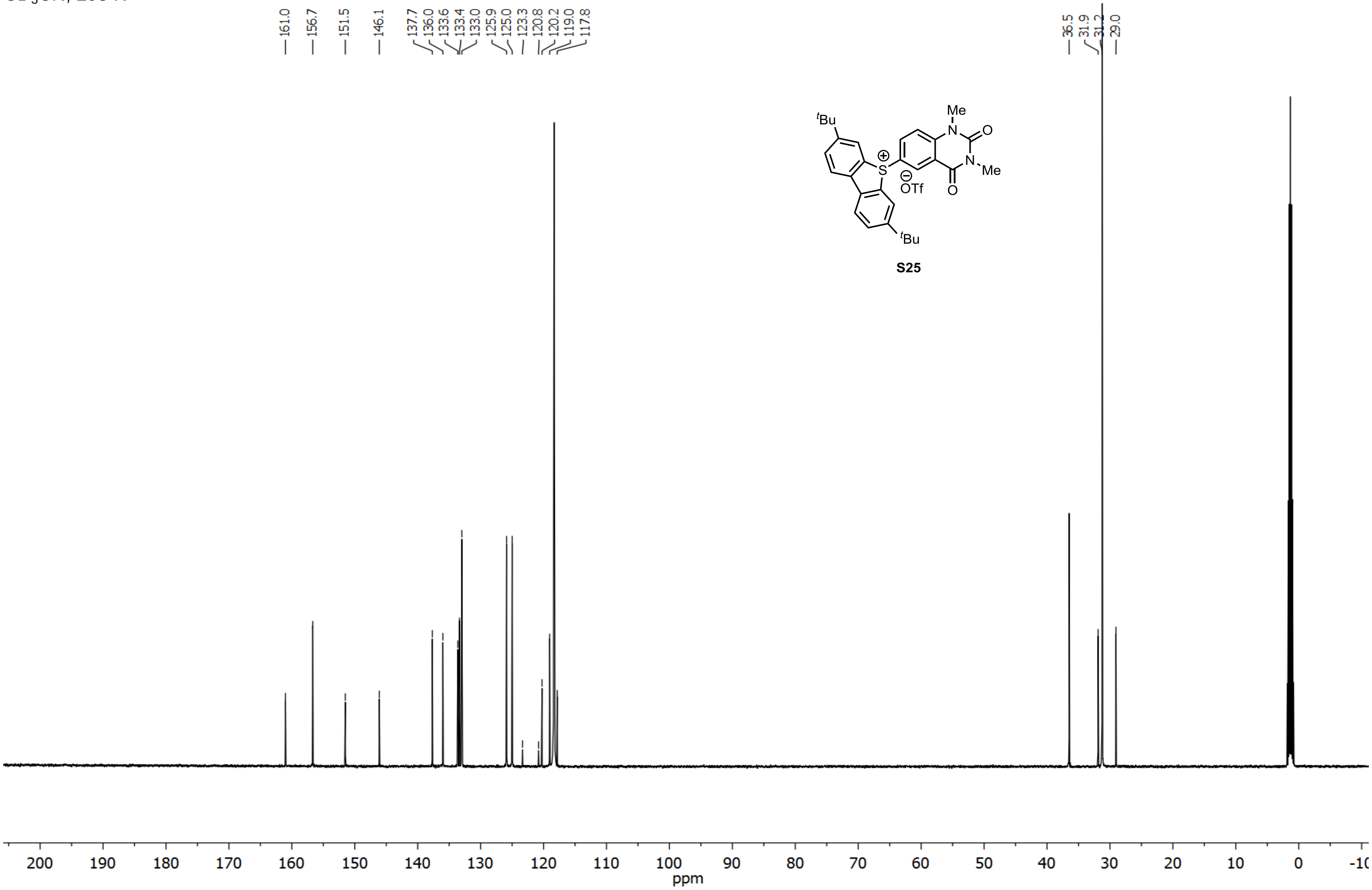

^{19}F NMR of 1,3-dimethylquinazoline-2,4(1*H*,3*H*)-dione-derived 3,7-di-*tert*-butyldibenzothiophenium salt S25

CD_3CN, 298 K

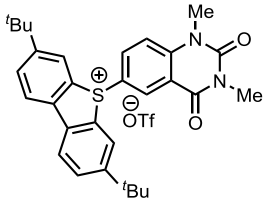

S25

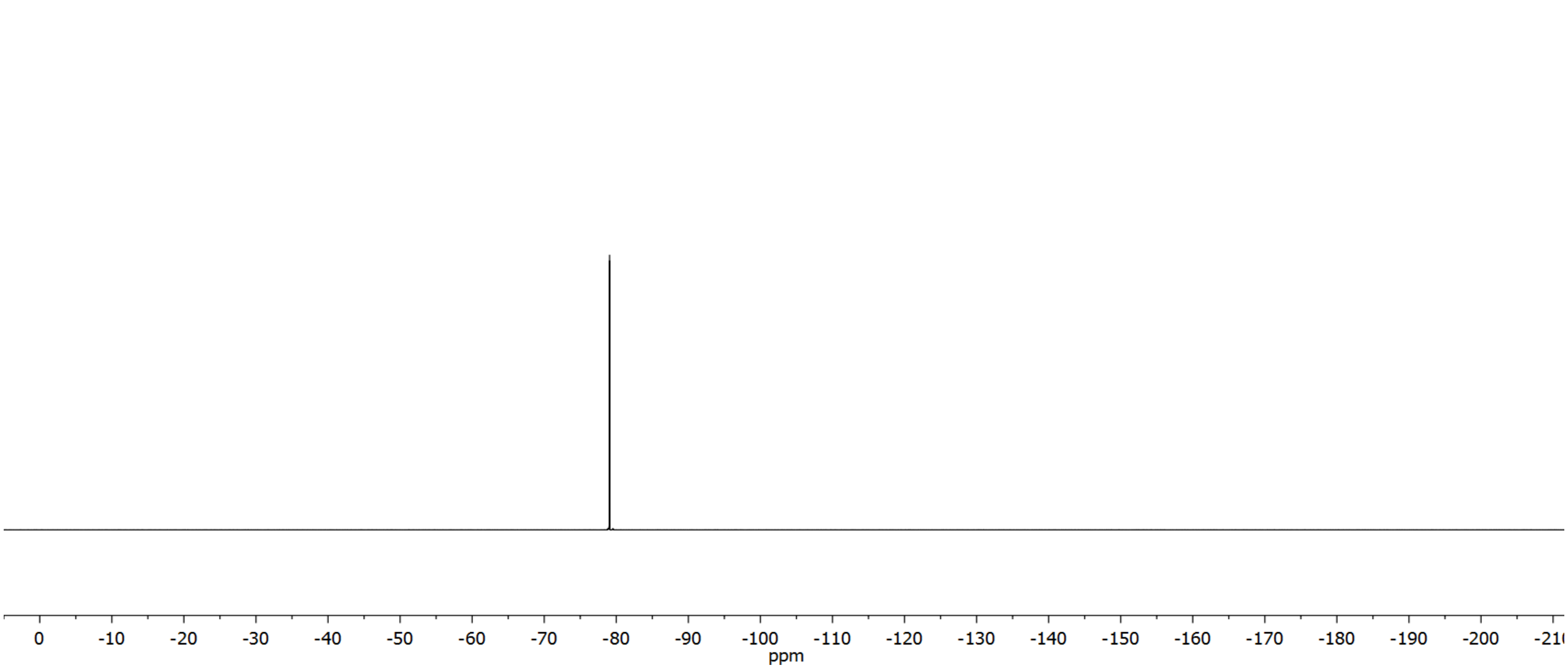

^{1}H NMR of 1,3-dimethylquinazoline-2,4(1*H*,3*H*)-dione-derived dibenzothiophenium salt S25-DBT

CD$_3$CN, 298 K

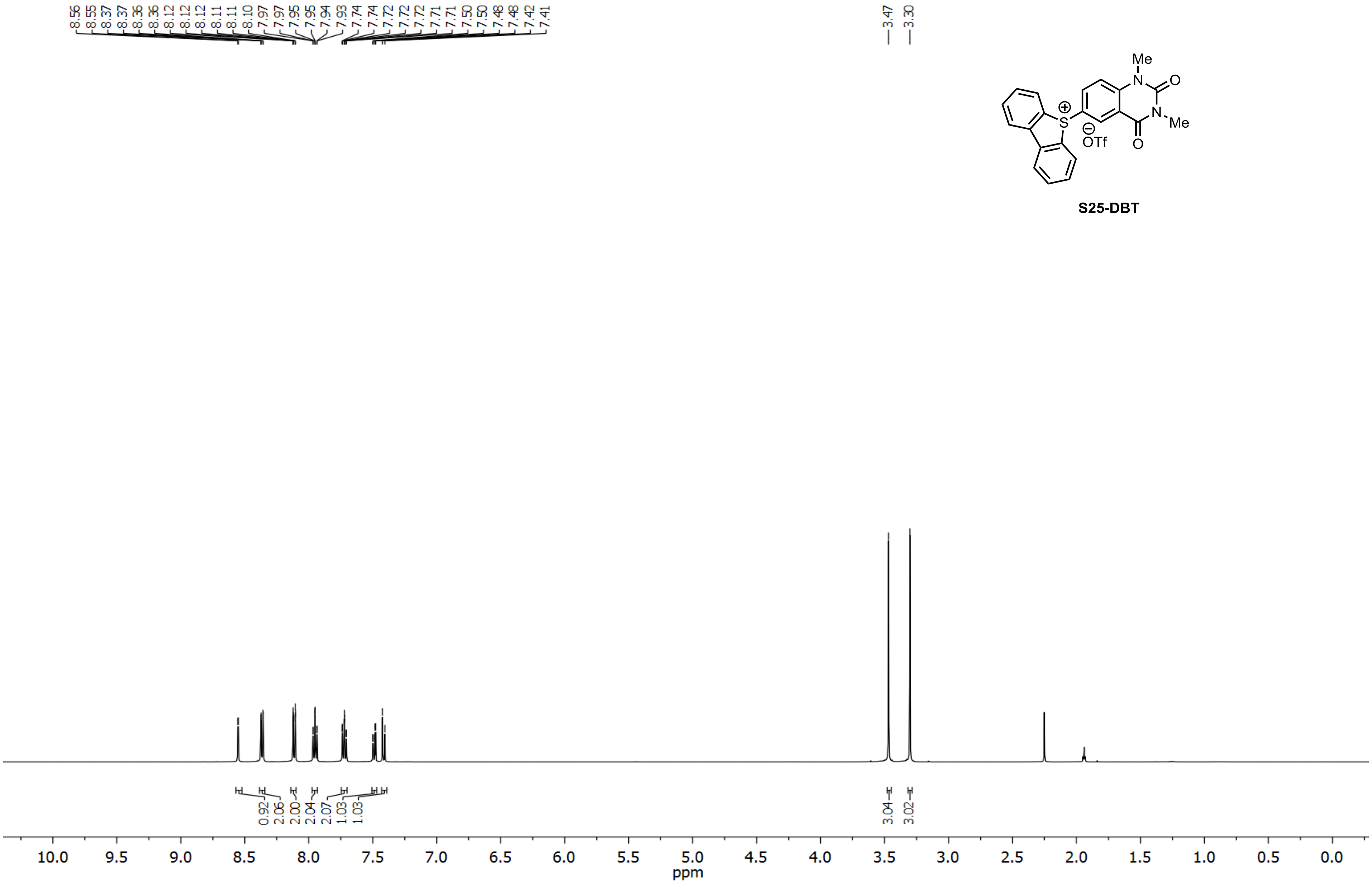

S25-DBT

^{13}C NMR of 1,3-dimethylquinazoline-2,4(1*H*,3*H*)-dione-derived dibenzothiophenium salt S25-DBT

CD$_3$CN, 298 K

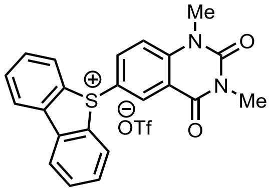

S25-DBT

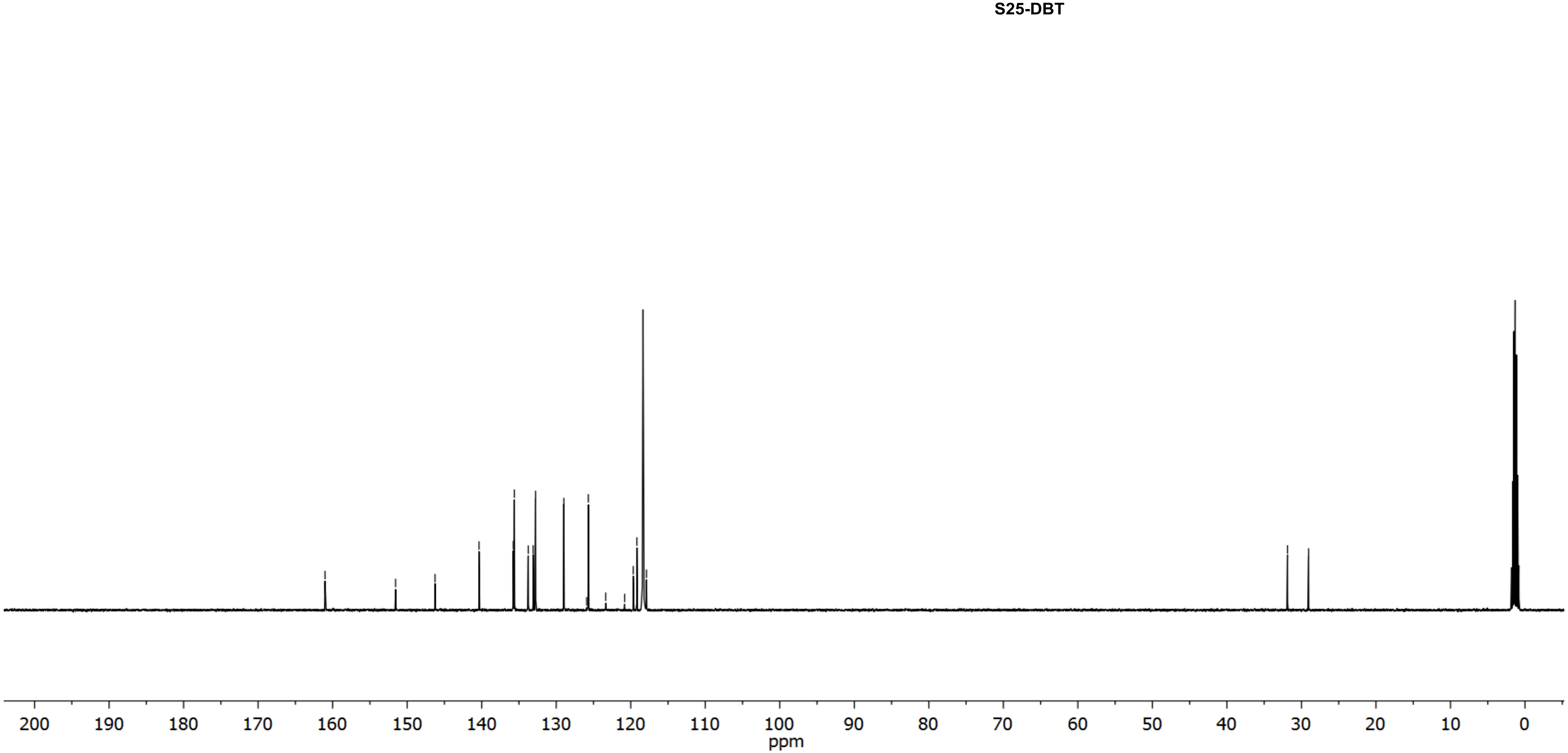

^{19}F NMR of 1,3-dimethylquinazoline-2,4(1*H*,3*H*)-dione-derived dibenzothiophenium salt S25-DBT

CD$_3$CN, 298 K

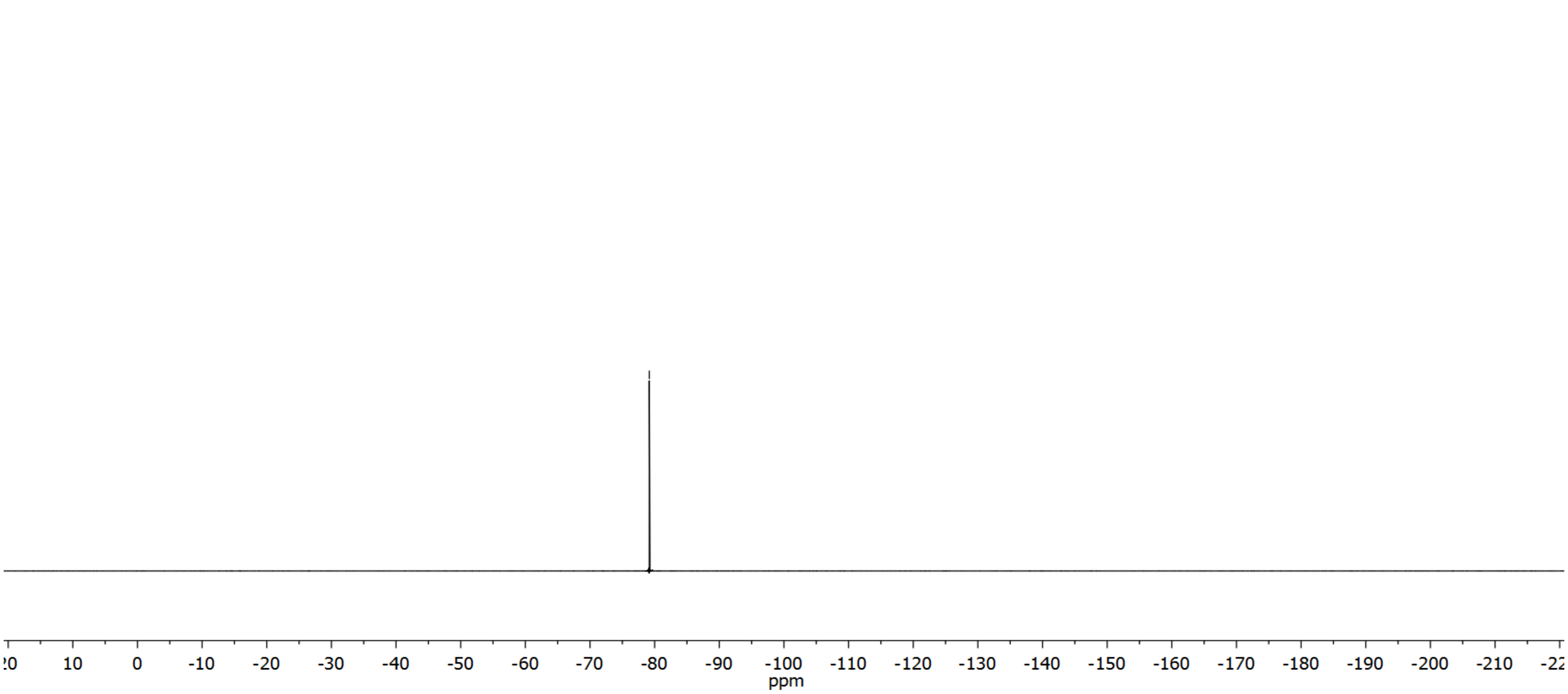

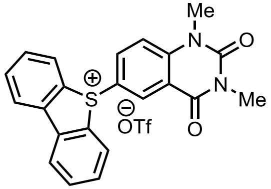

S25-DBT

^{1}H NMR of airacetam-derived 3,7-di-*tert*-butyldibenzothiophenium salt S26

CD$_3$CN, 298 K

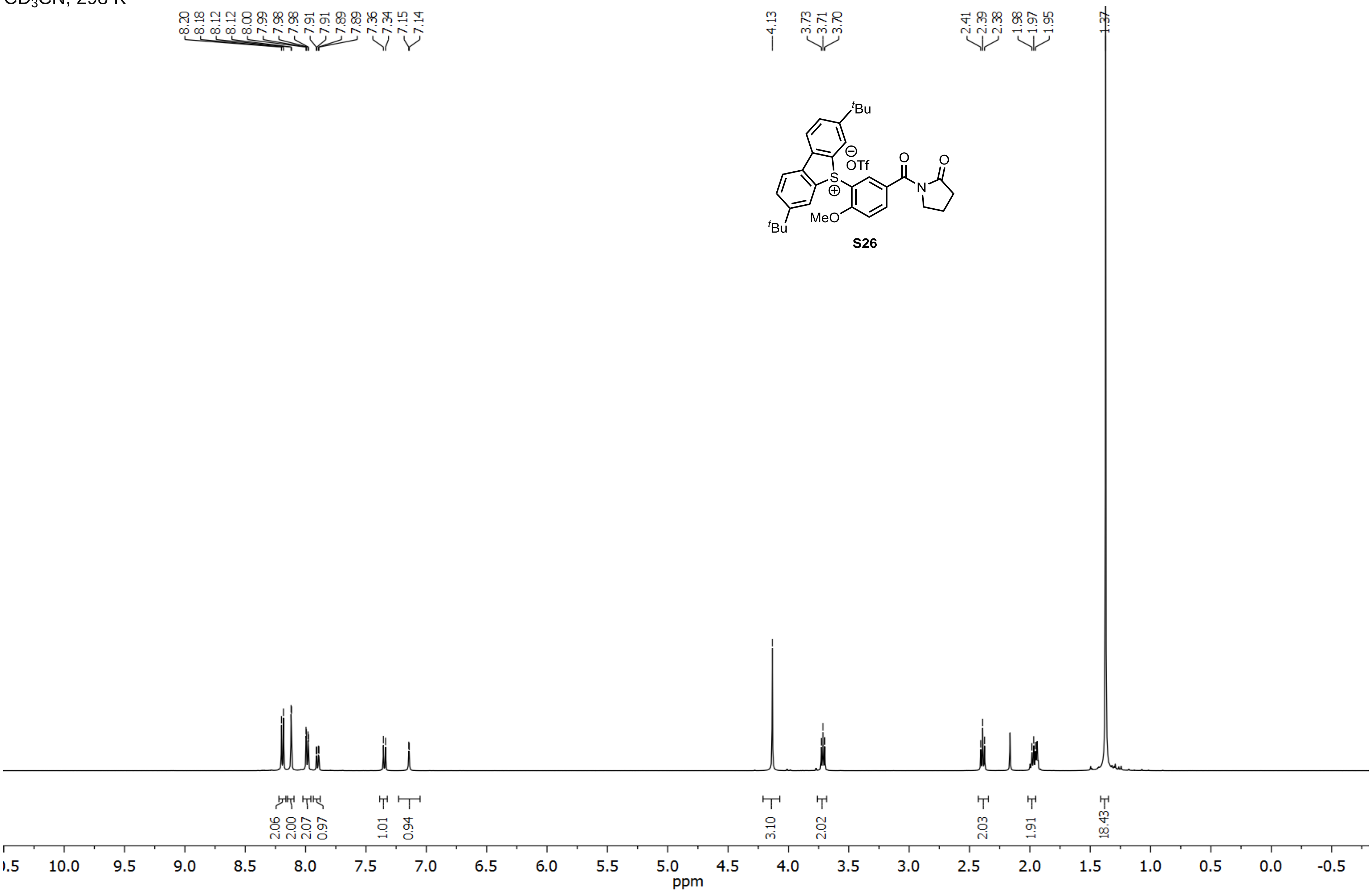

^{13}C NMR of airacetam-derived 3,7-di-*tert*-butyldibenzothiophenium salt S26

CD_3CN, 298 K

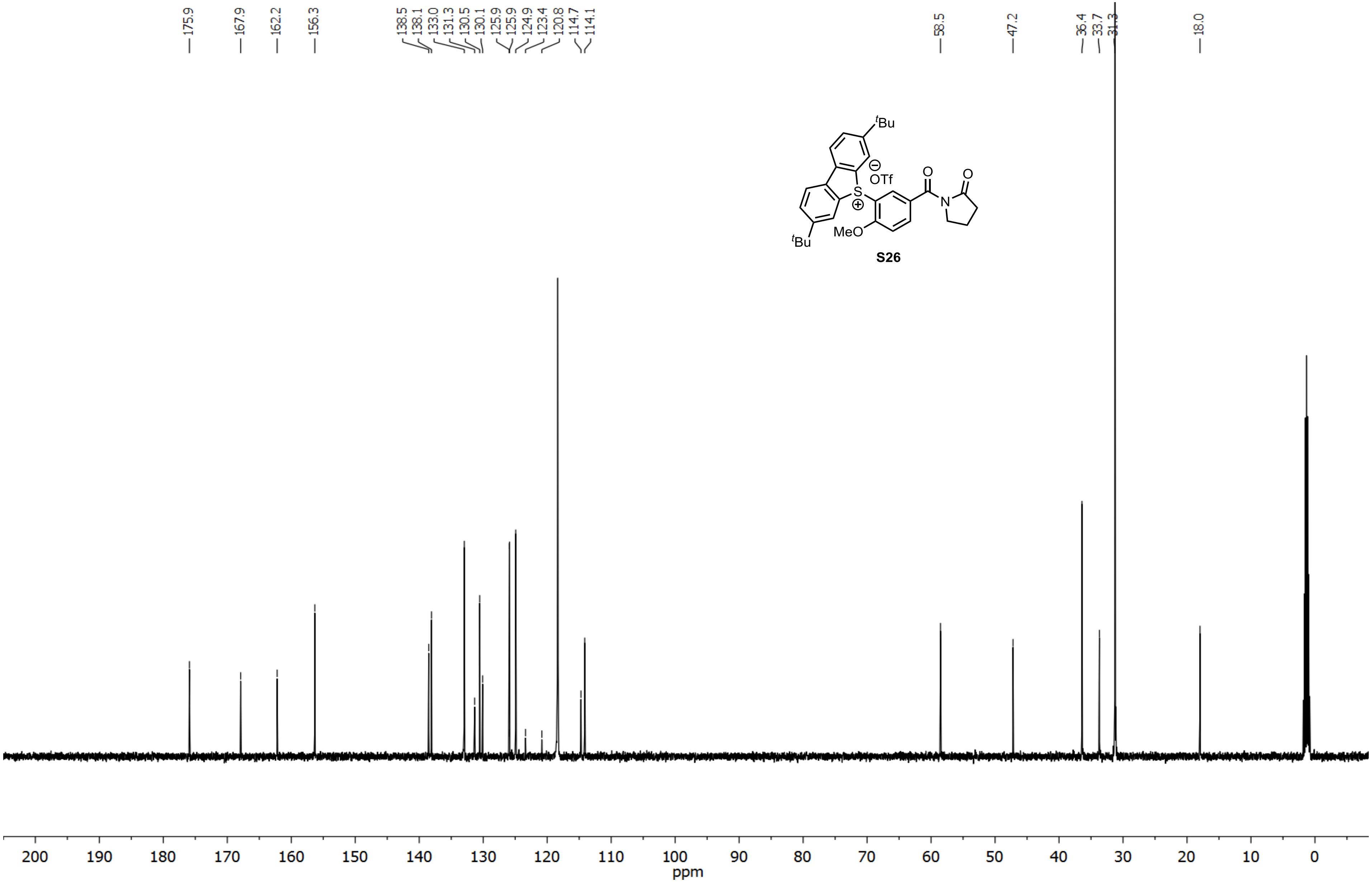

^{19}F NMR of airacetam-derived 3,7-di-*tert*-butyldibenzothiophenium salt S26

CD_3CN, 298 K

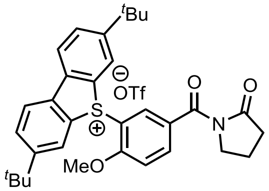

S26

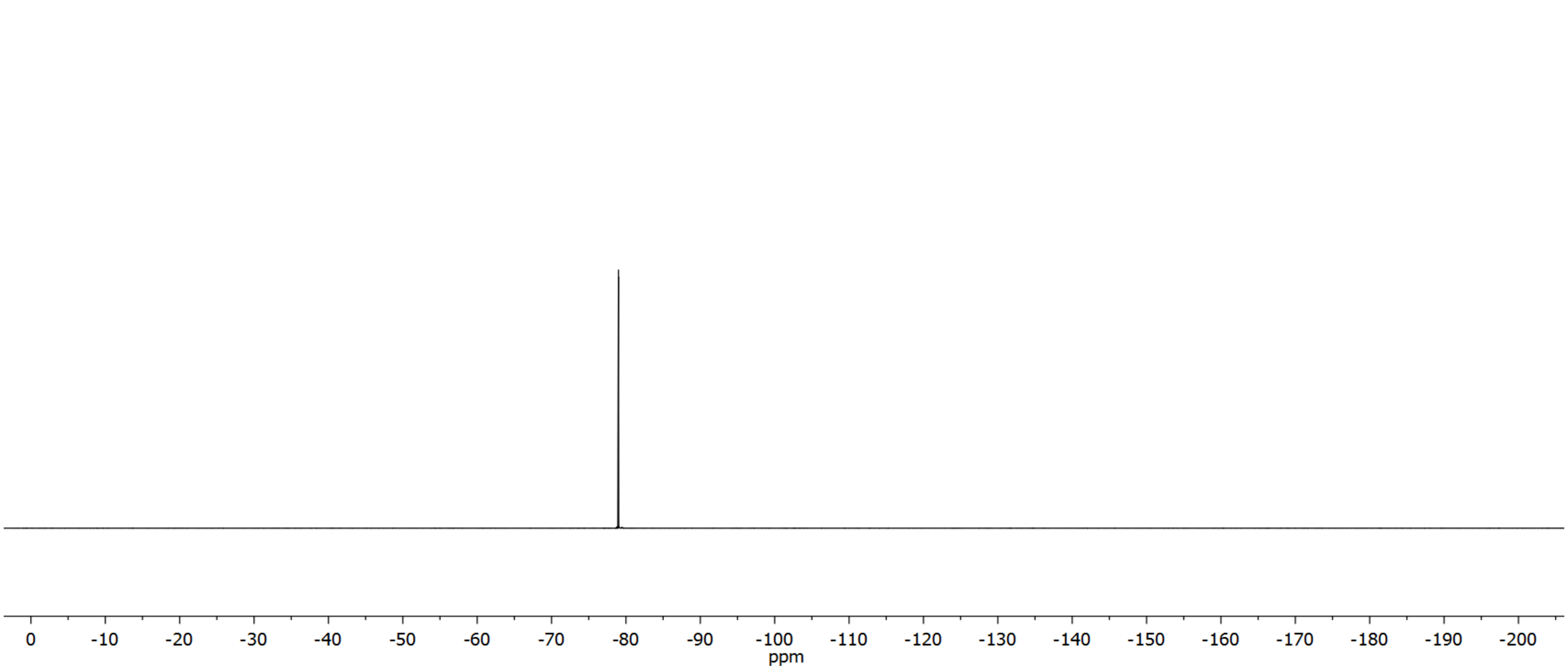

^{1}H NMR of salicin pentaacetate-derived 2,8-dimethoxydibenzothiophenium salt S27

CD_3CN, 298 K

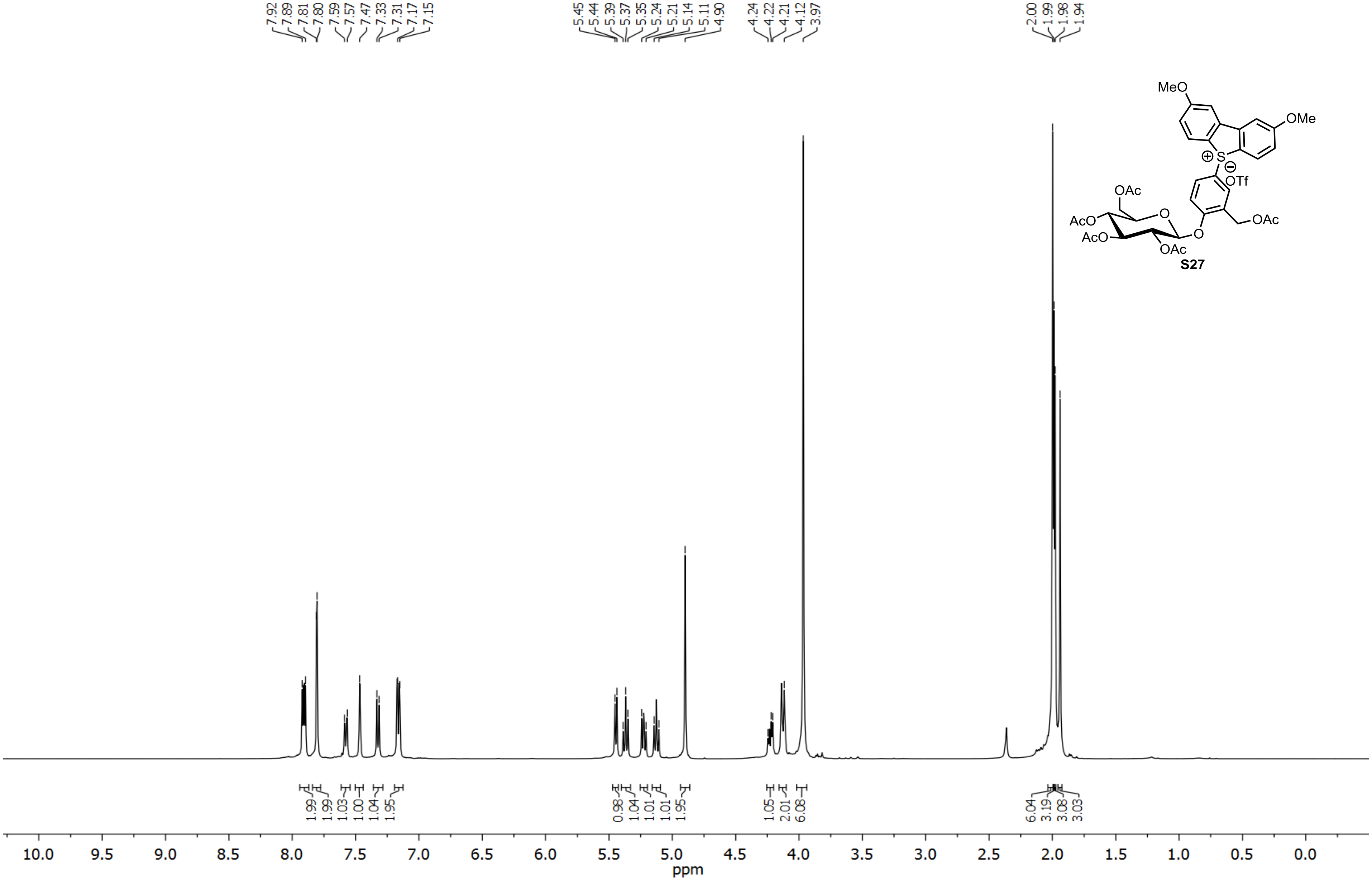

^{13}C NMR of salicin pentaacetate-derived 2,8-dimethoxydibenzothiophenium salt S27

CD_3CN, 298 K

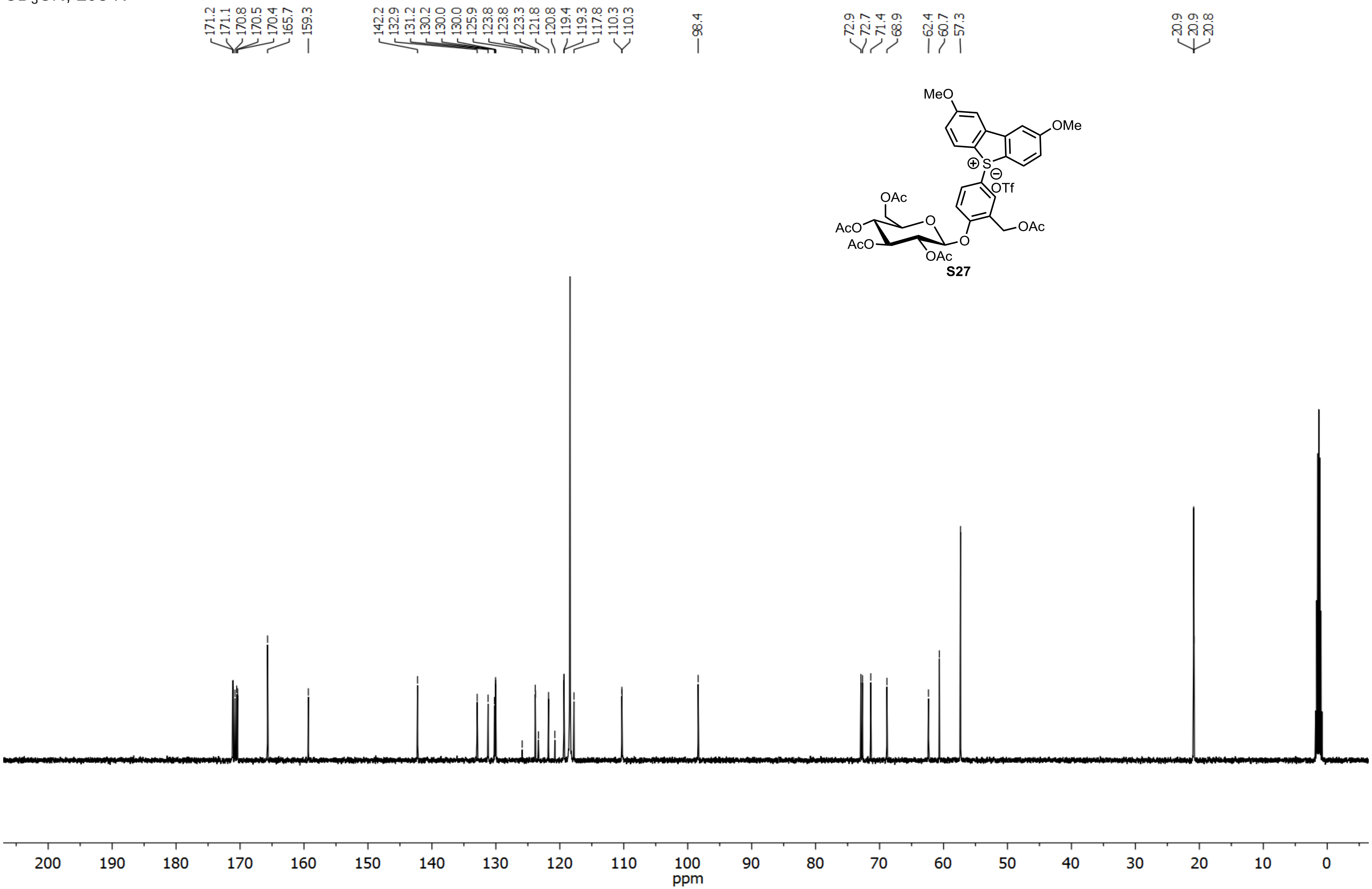

^{19}F NMR of salicin pentaacetate-derived 2,8-dimethoxydibenzothiophenium salt S27

CD_3CN, 298 K

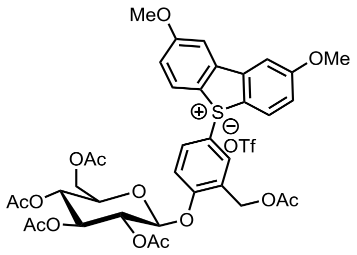

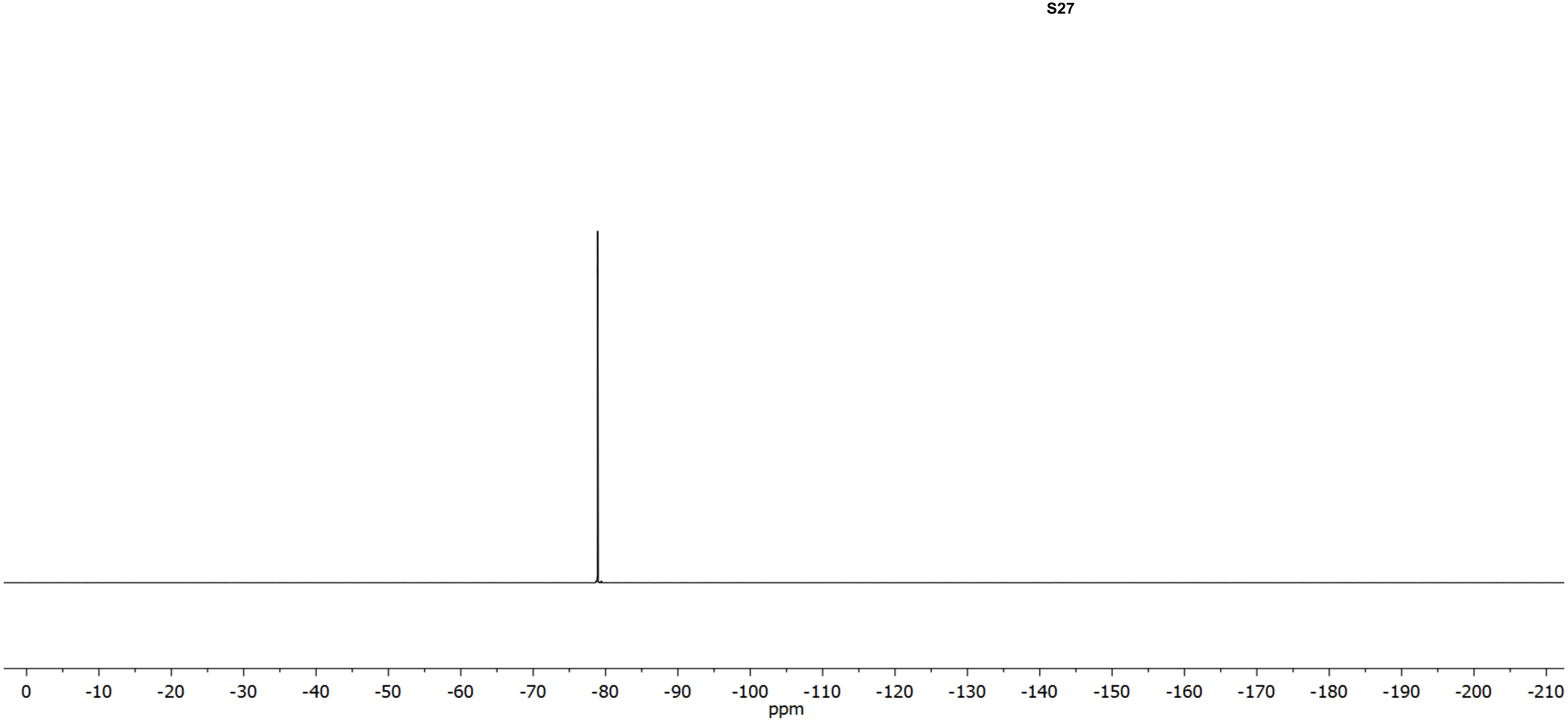

^{1}H NMR of biphenyl-derived thianthrenium salt 1-TT

CD_3CN, 298 K

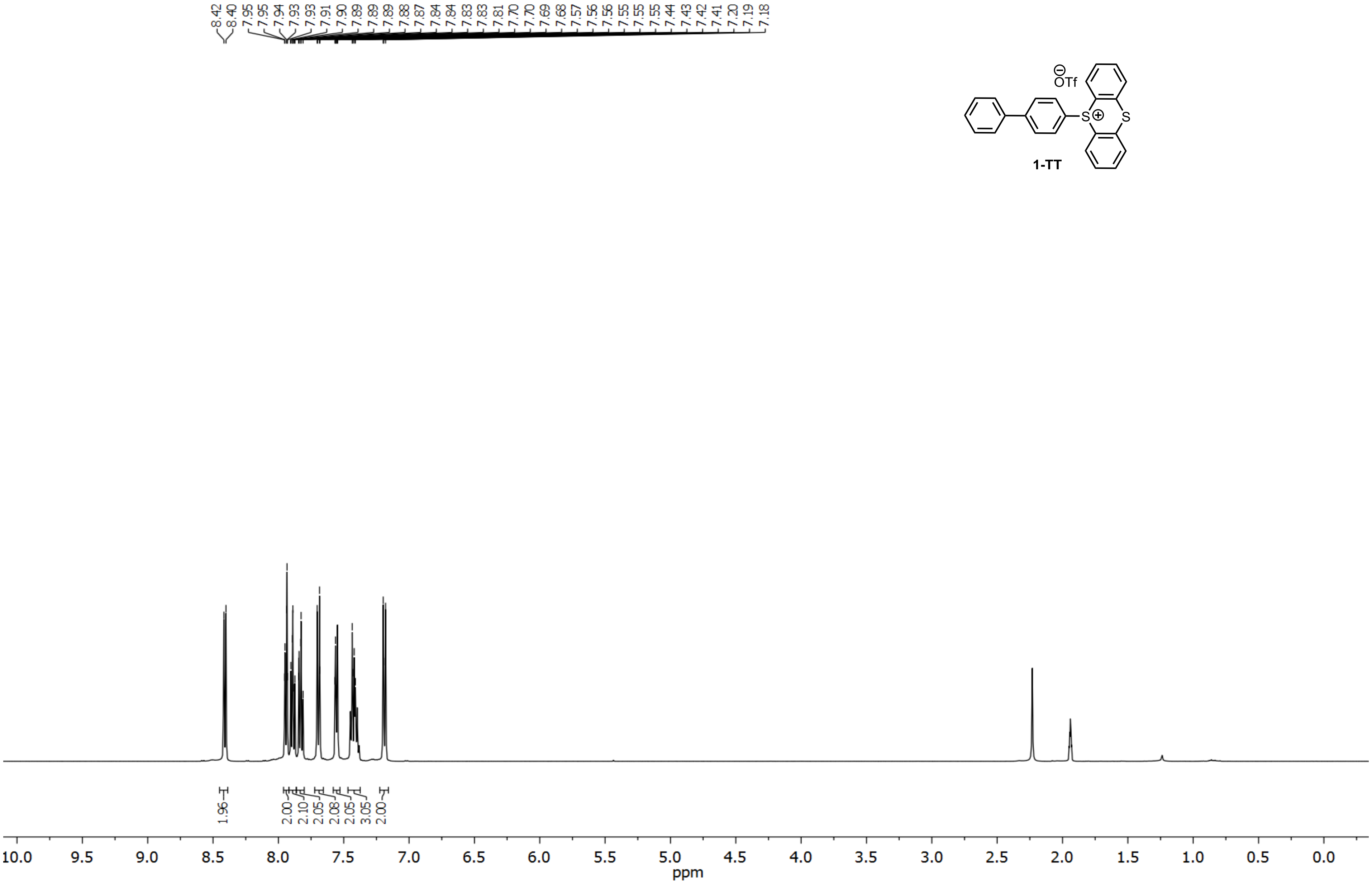

^{13}C NMR of biphenyl-derived thianthrenium salt 1-TT

CD_3CN, 298 K

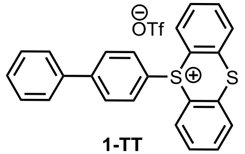

1-TT

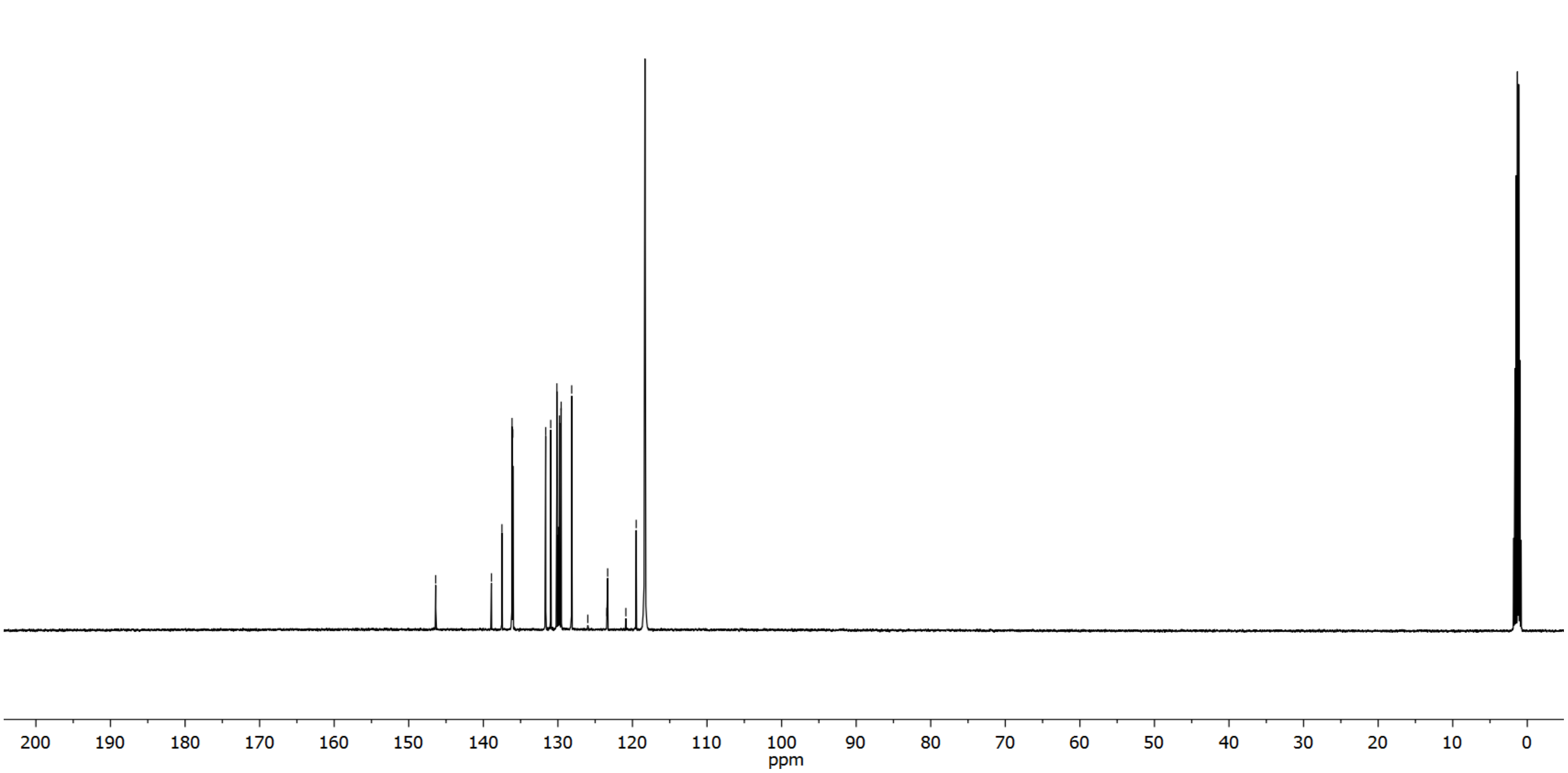

^{19}F NMR of biphenyl-derived thianthrenium salt 1-TT

CD_3CN, 298 K

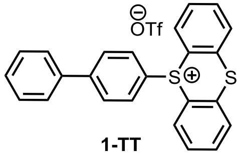

1-TT

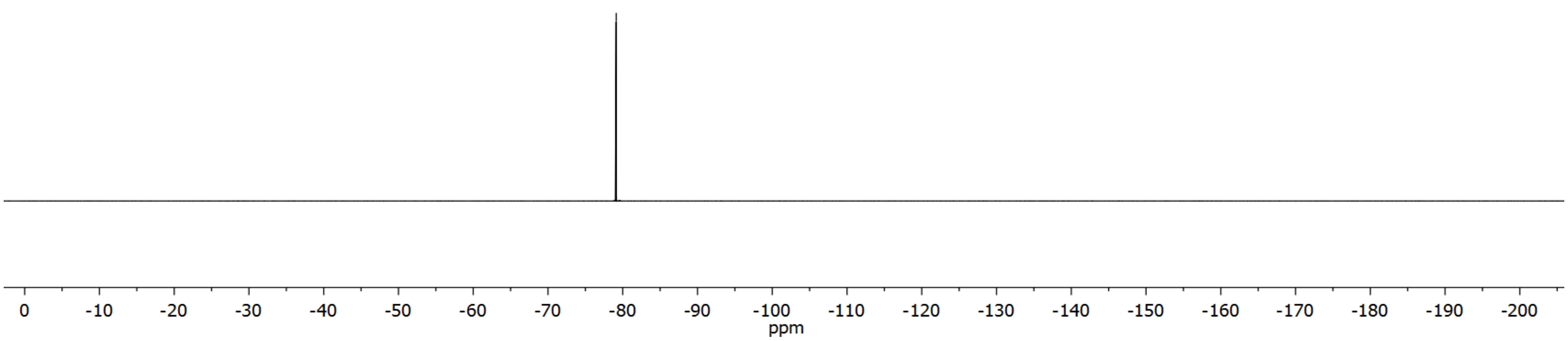

^{1}H NMR of 4-fluorobiphenyl (1a)

$CDCl_3$, 298 K

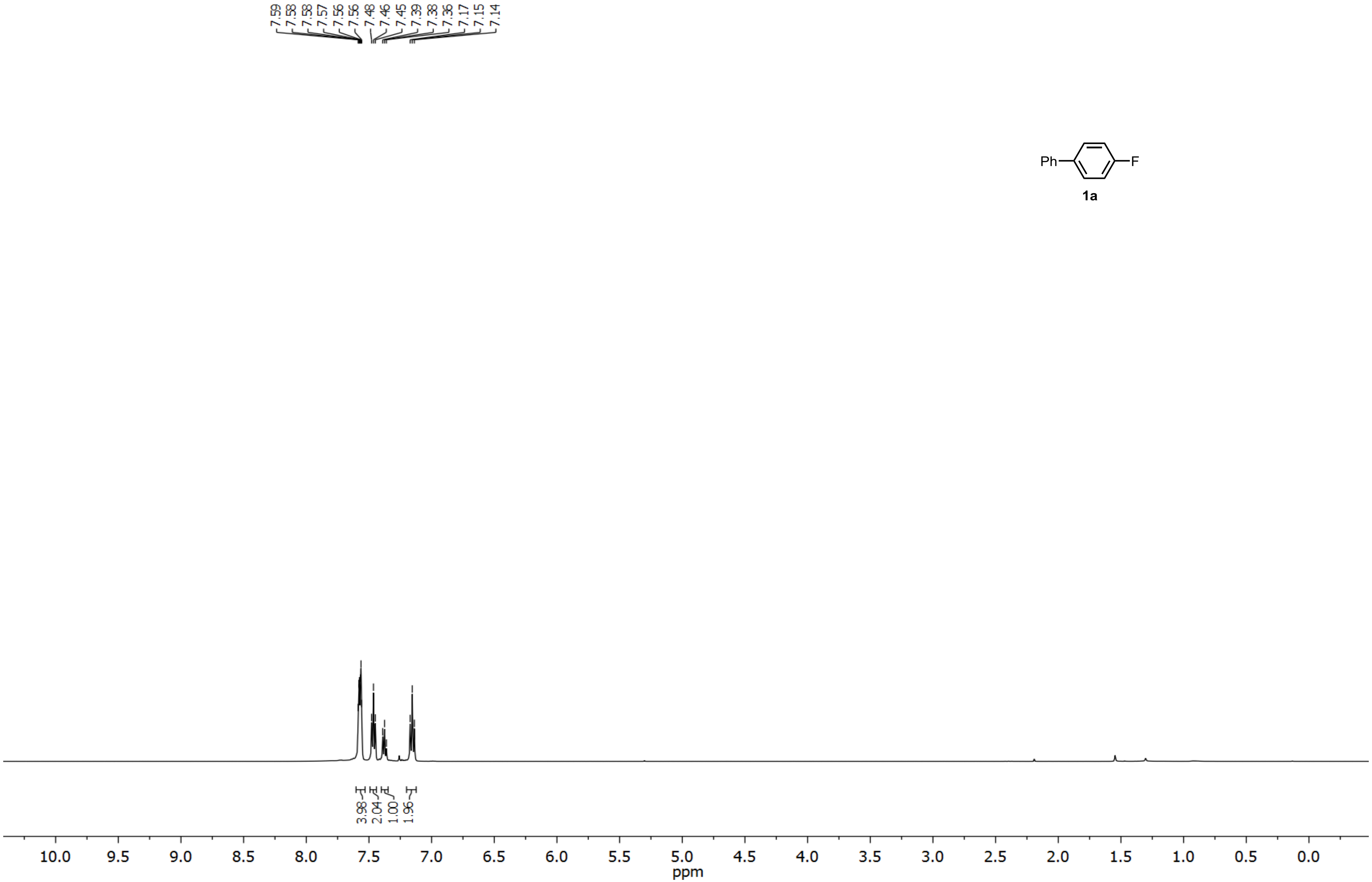

1a

^{13}C NMR of 4-fluorobiphenyl (1a)

$CDCl_3$, 298 K

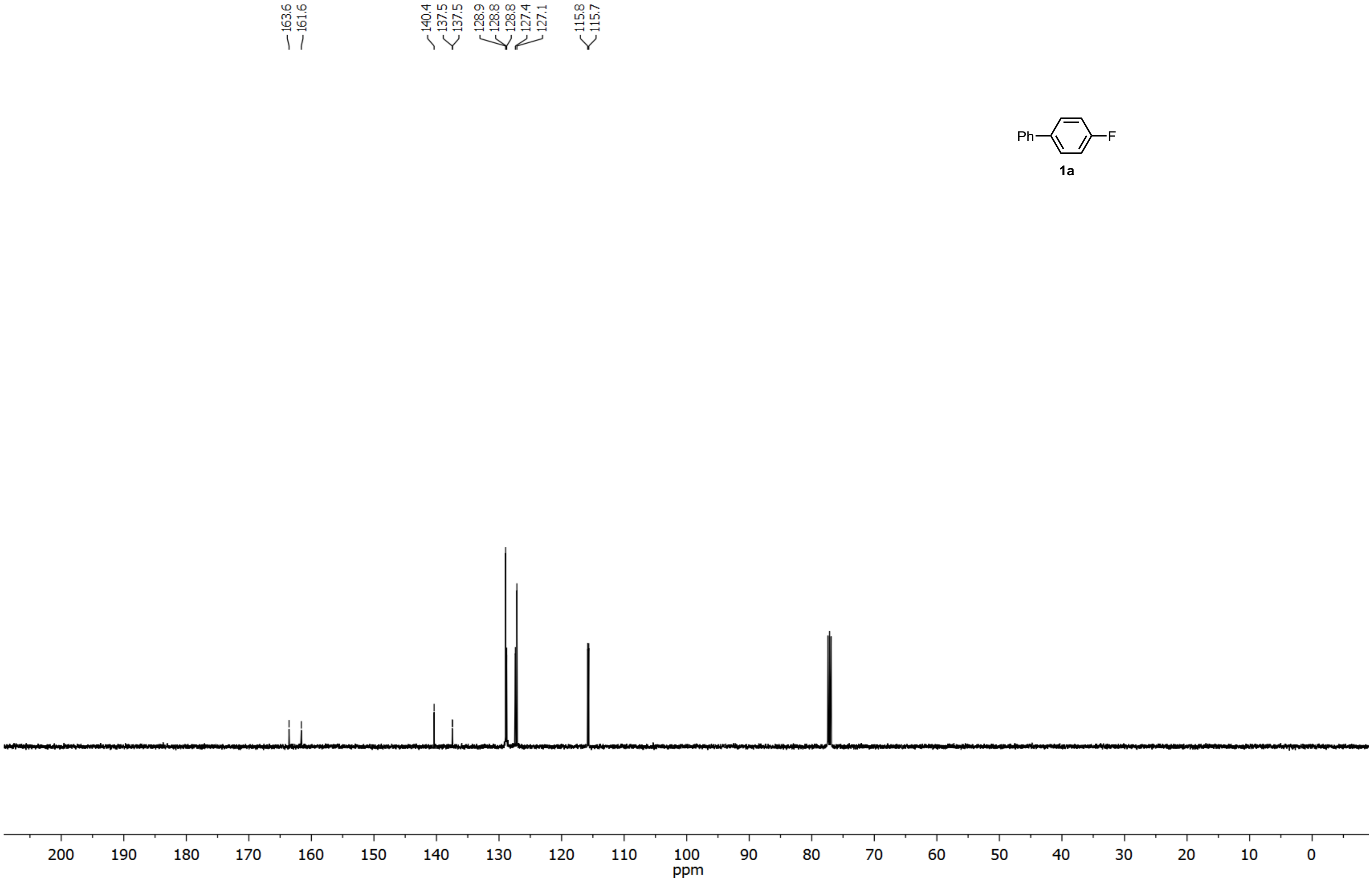

^{19}F NMR of 4-fluorobiphenyl (1a)

$CDCl_3$, 298 K

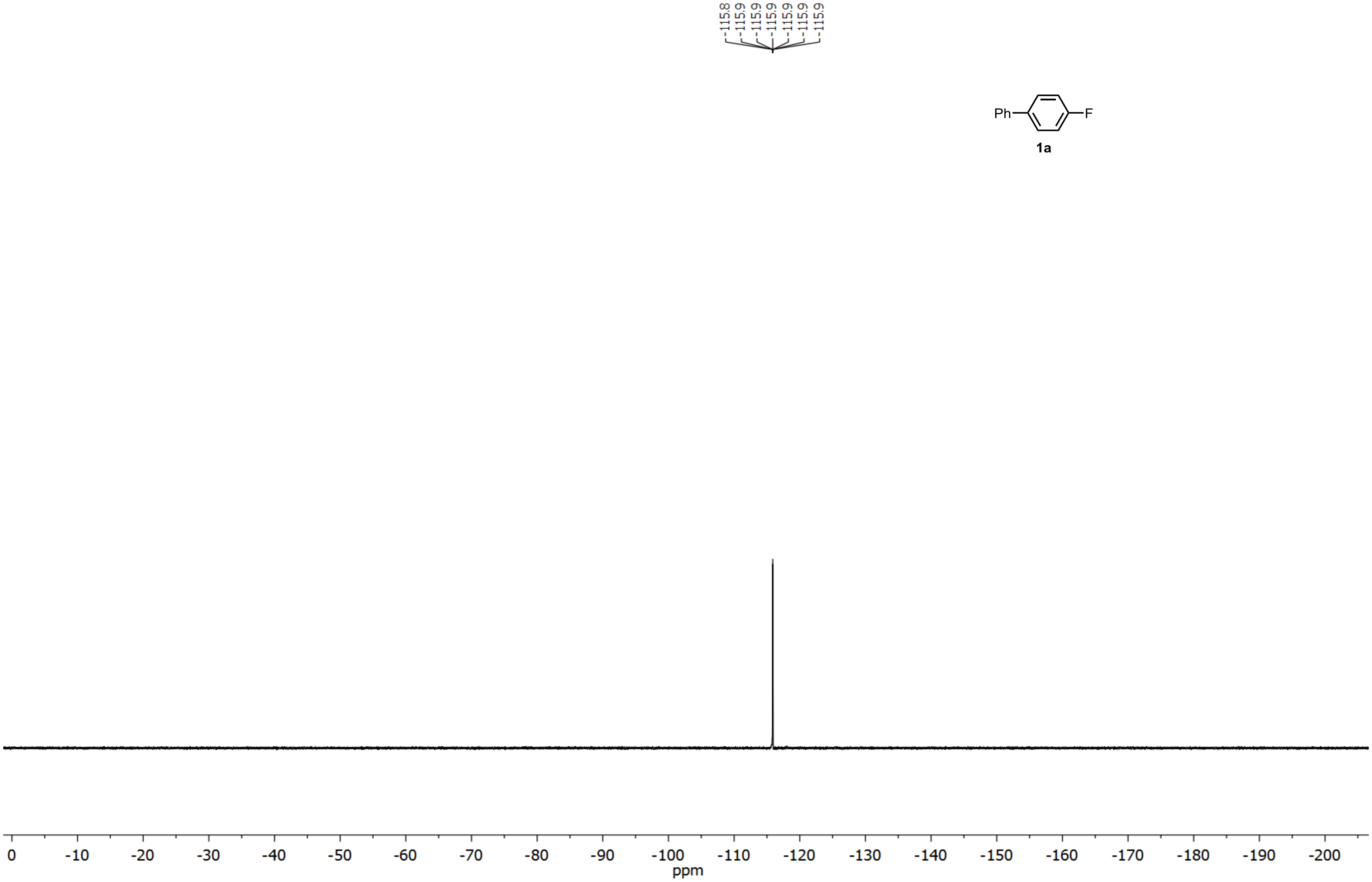

^{1}H NMR of [1,1'-biphenyl]-4-yl(2-((2-fluorophenyl)thio)phenyl)sulfane (1b)

$CDCl_3$, 298 K

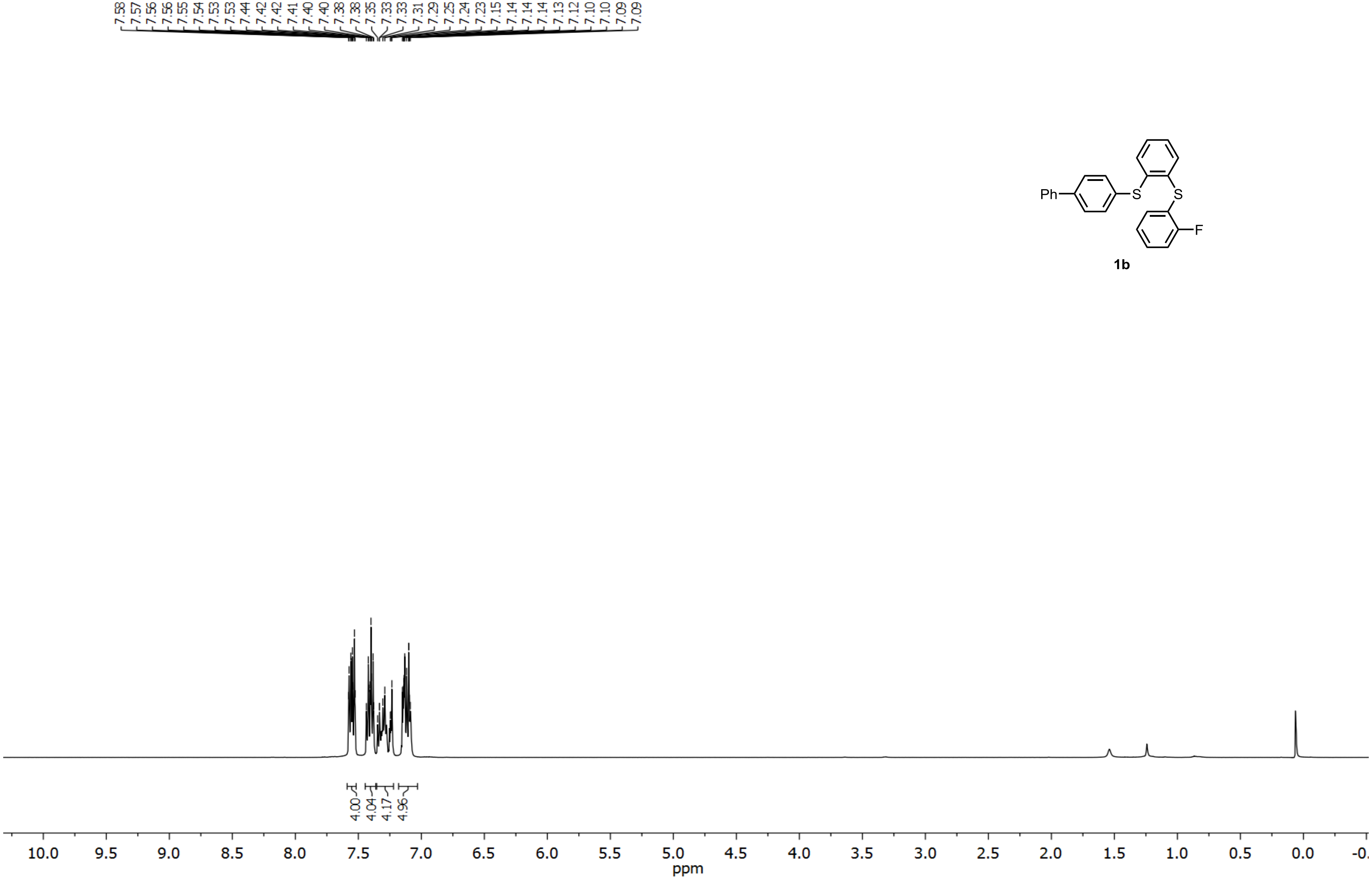

^{13}C NMR of [1,1'-biphenyl]-4-yl(2-((2-fluorophenyl)thio)phenyl)sulfane (1b)

CDCl$_3$, 298 K

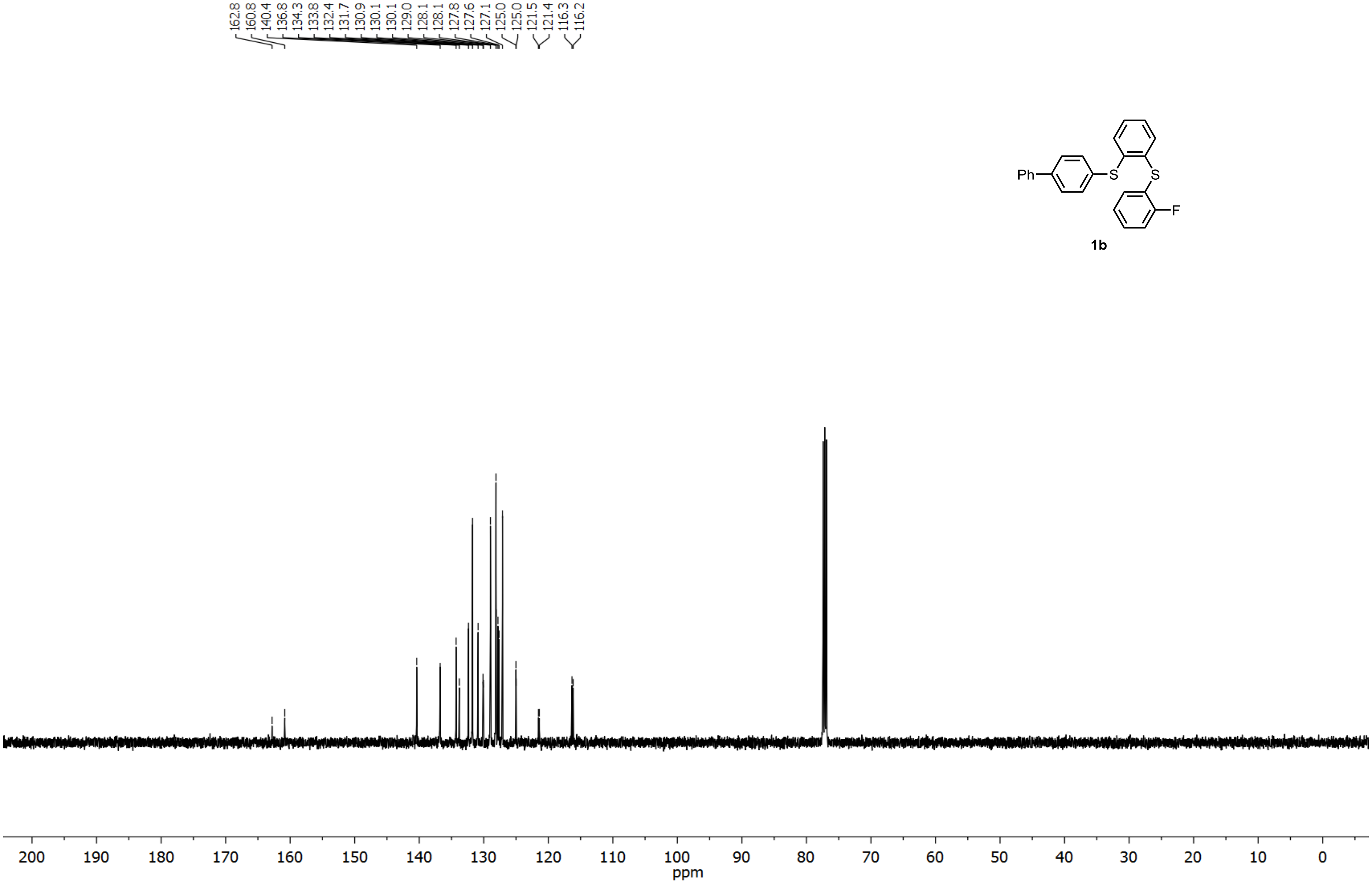

1b

^{19}F NMR of [1,1'-biphenyl]-4-yl(2-((2-fluorophenyl)thio)phenyl)sulfane (1b)

$CDCl_3$, 298 K

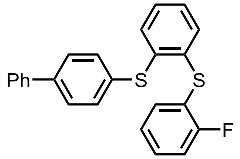

1b

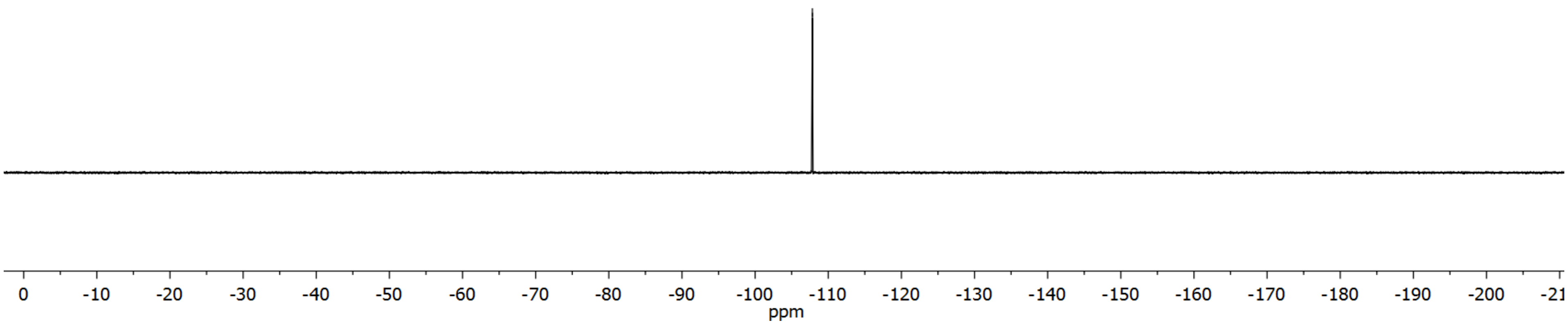

^{1}H NMR of 6-fluoro-1,3-dimethylquinazoline-2,4(1*H*,3*H*)-dione (S28)

CDCl$_3$, 298 K

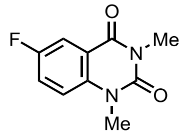

S28

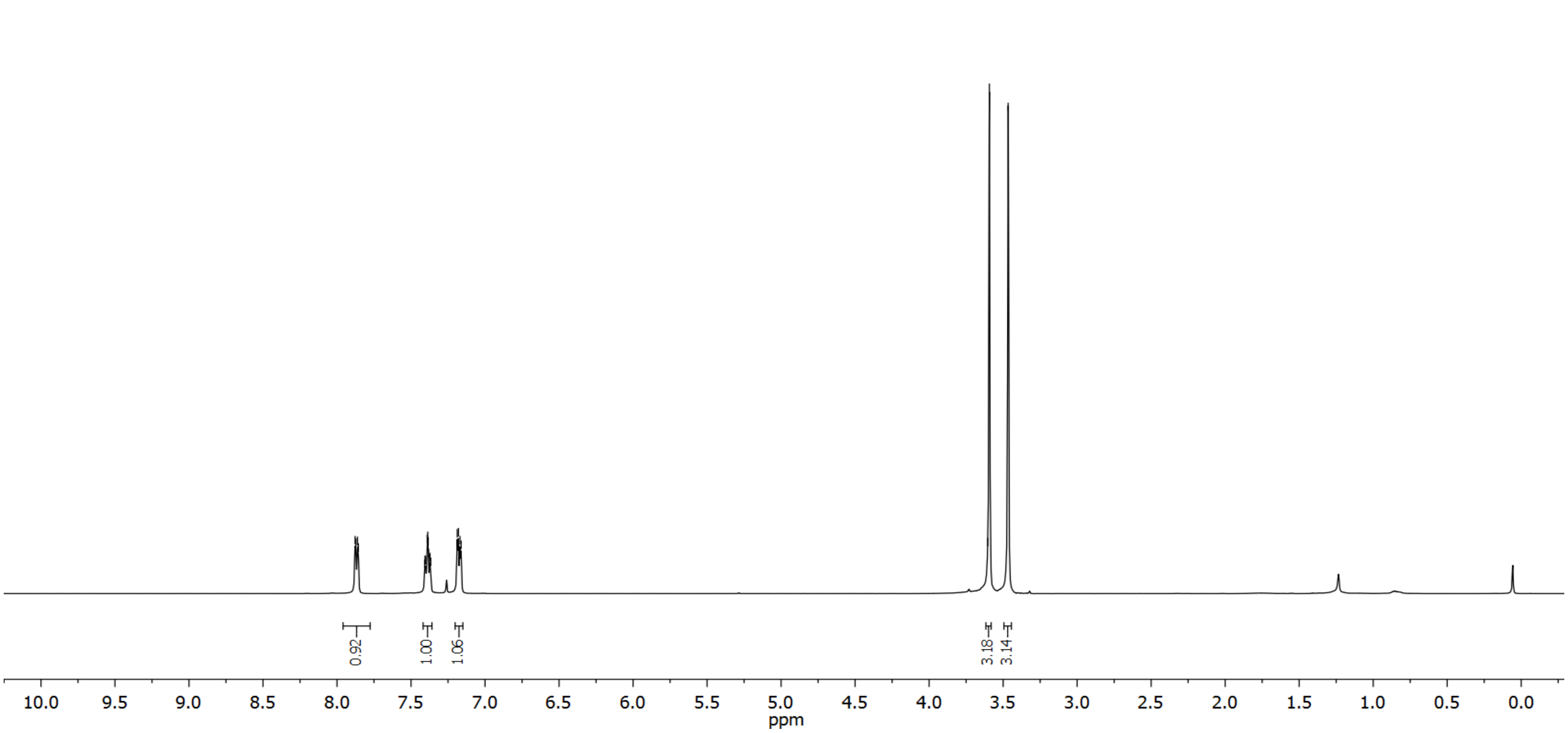

^{13}C NMR of 6-fluoro-1,3-dimethylquinazoline-2,4(1*H*,3*H*)-dione (S28)

CDCl$_3$, 298 K

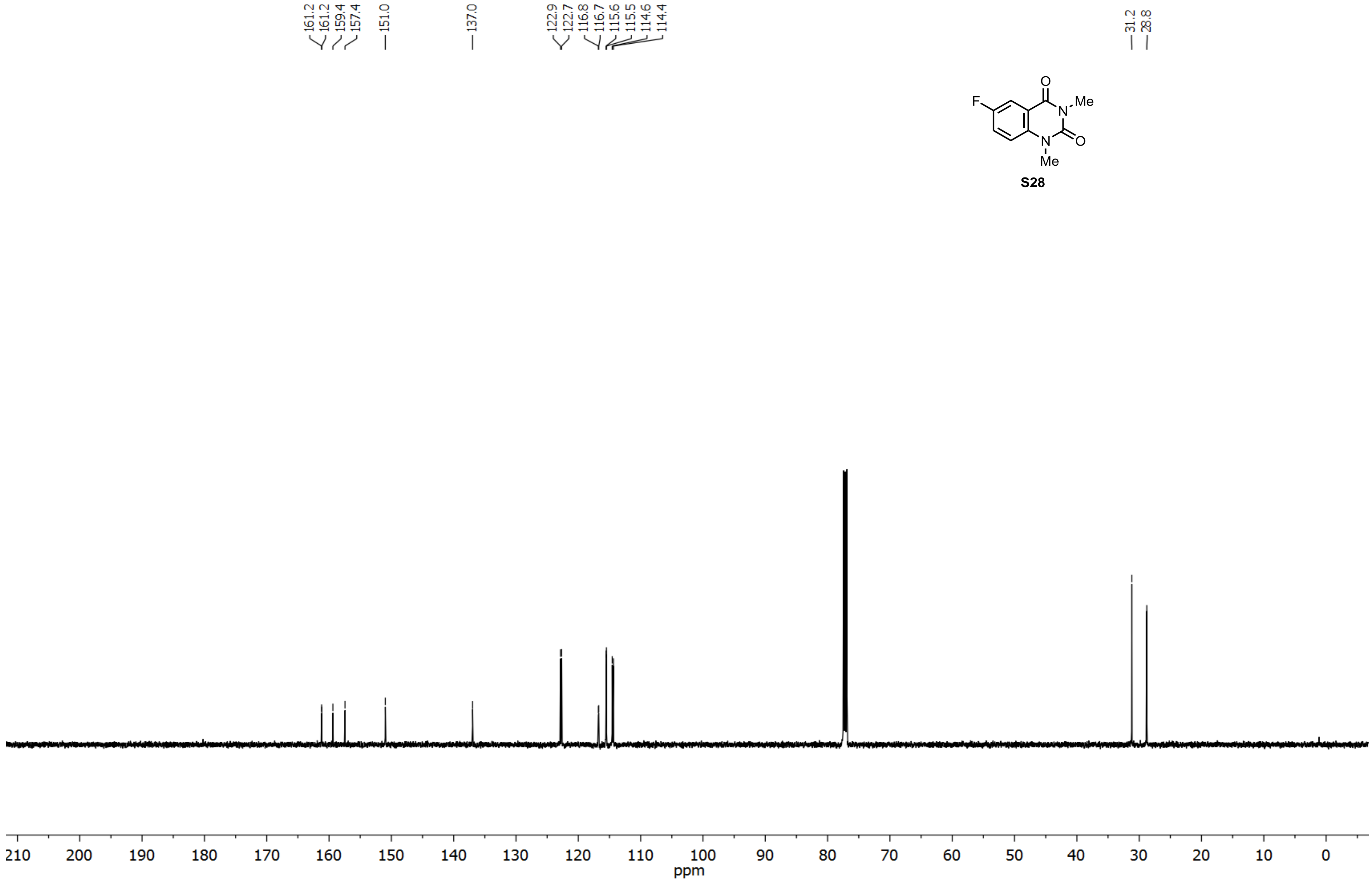

S28

^{19}F NMR of 6-fluoro-1,3-dimethylquinazoline-2,4(1*H*,3*H*)-dione (S28)

$CDCl_3$, 298 K

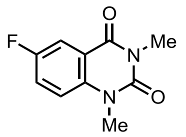

S28

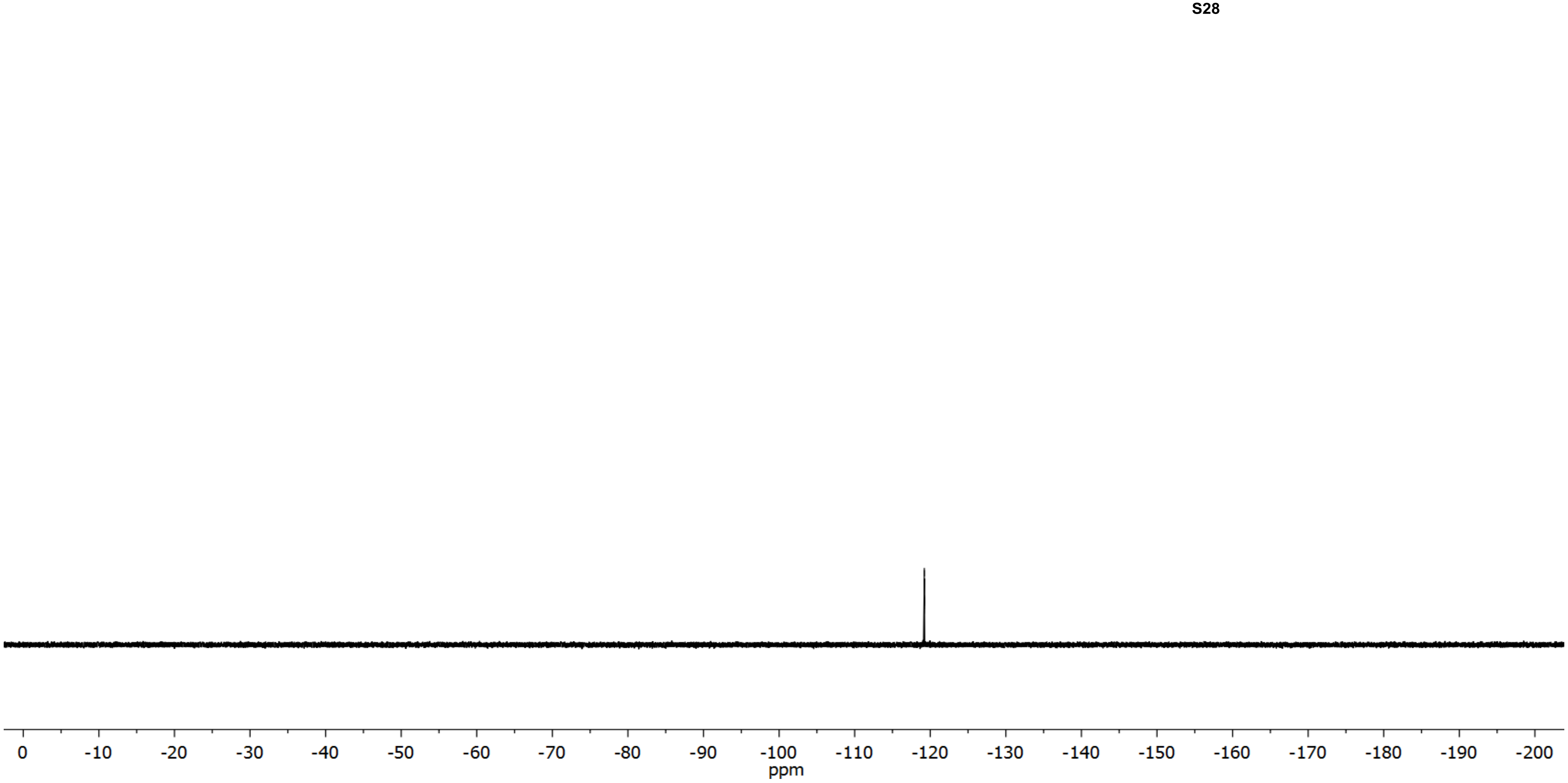

^{1}H NMR of fluorodiflunsial derivative S29

$CDCl_3$, 298 K

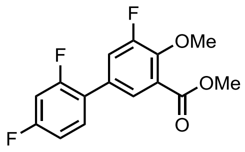

S29

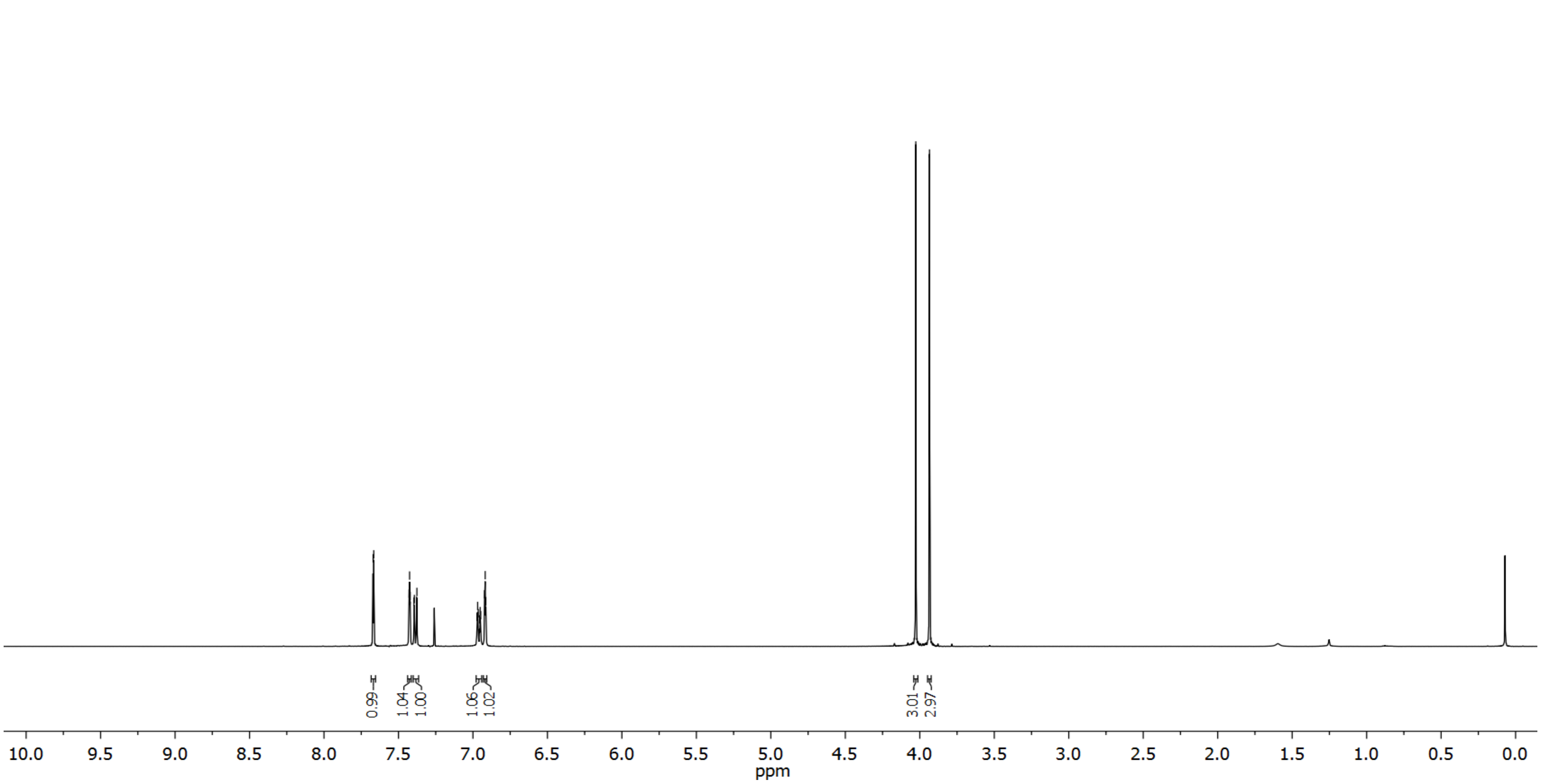

^{13}C NMR of fluorodiflunsial derivative S29

$CDCl_3$, 298 K

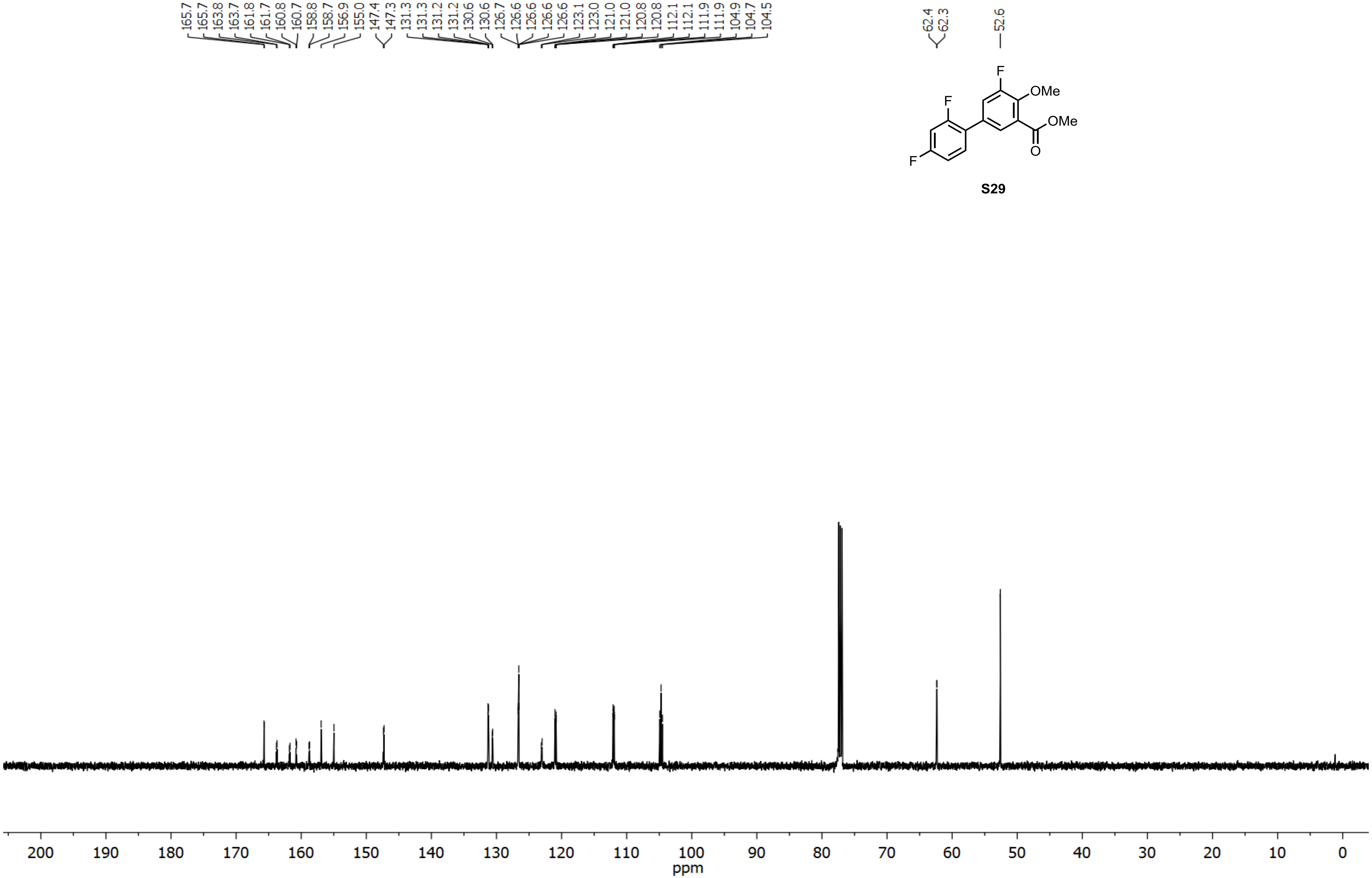

^{19}F NMR of fluorodiflunsial derivative S29

$CDCl_3$, 298 K

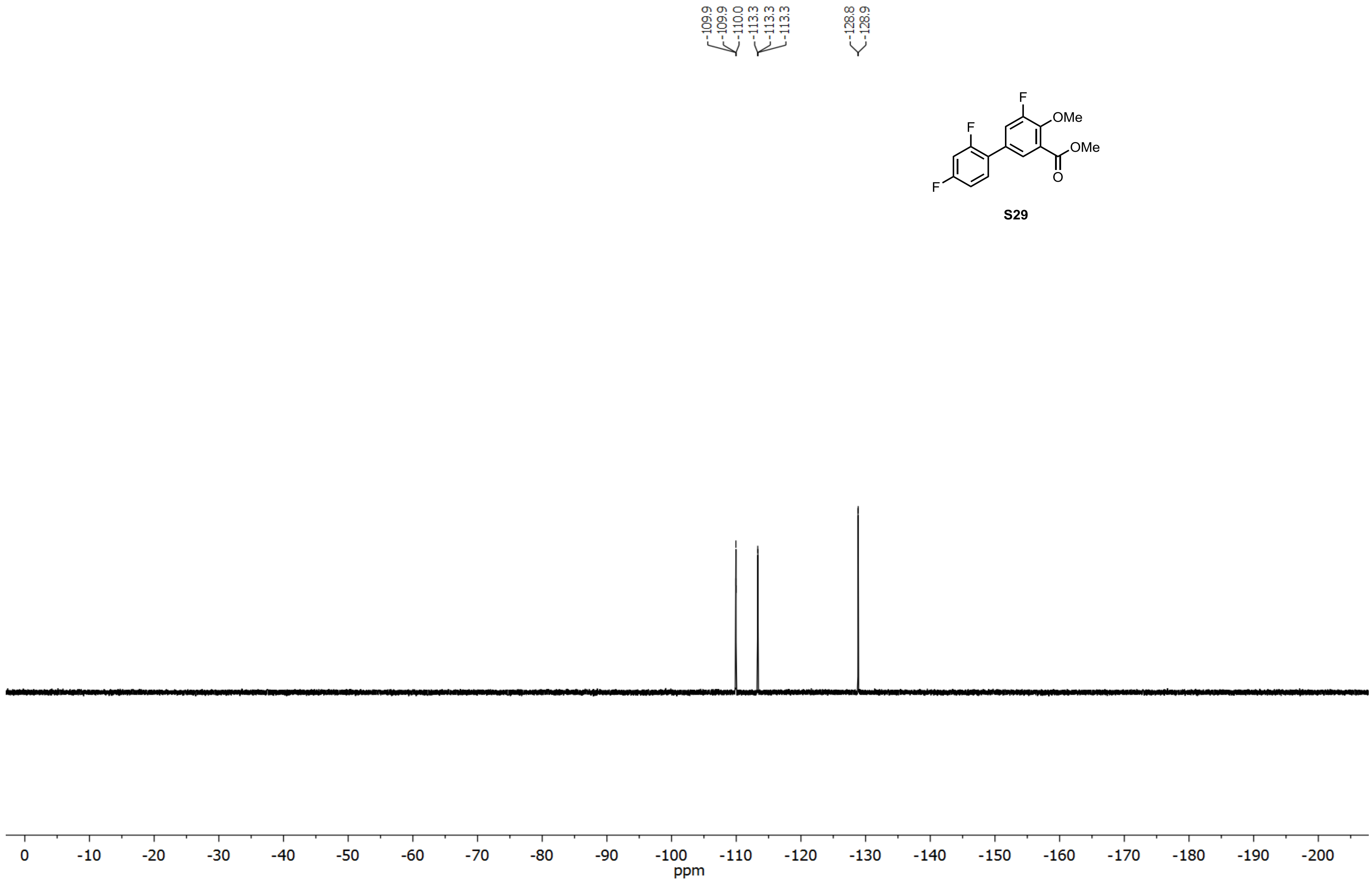

^{1}H NMR of fluoroclofibrate (S30)

$CDCl_3$, 298 K

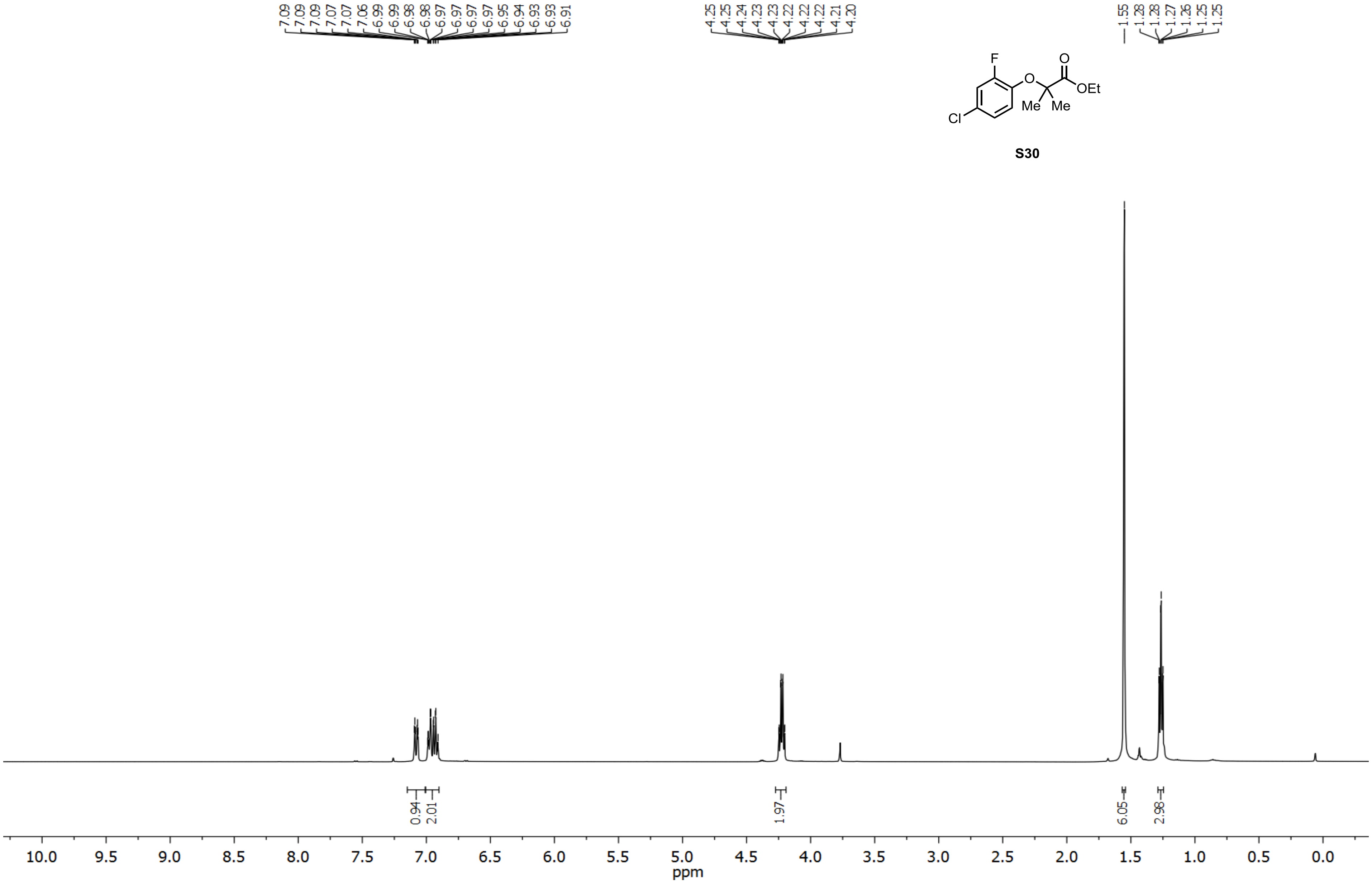

S30

^{13}C NMR of fluoroclofibrate (S30)

$CDCl_3$, 298 K

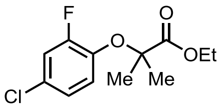

S30

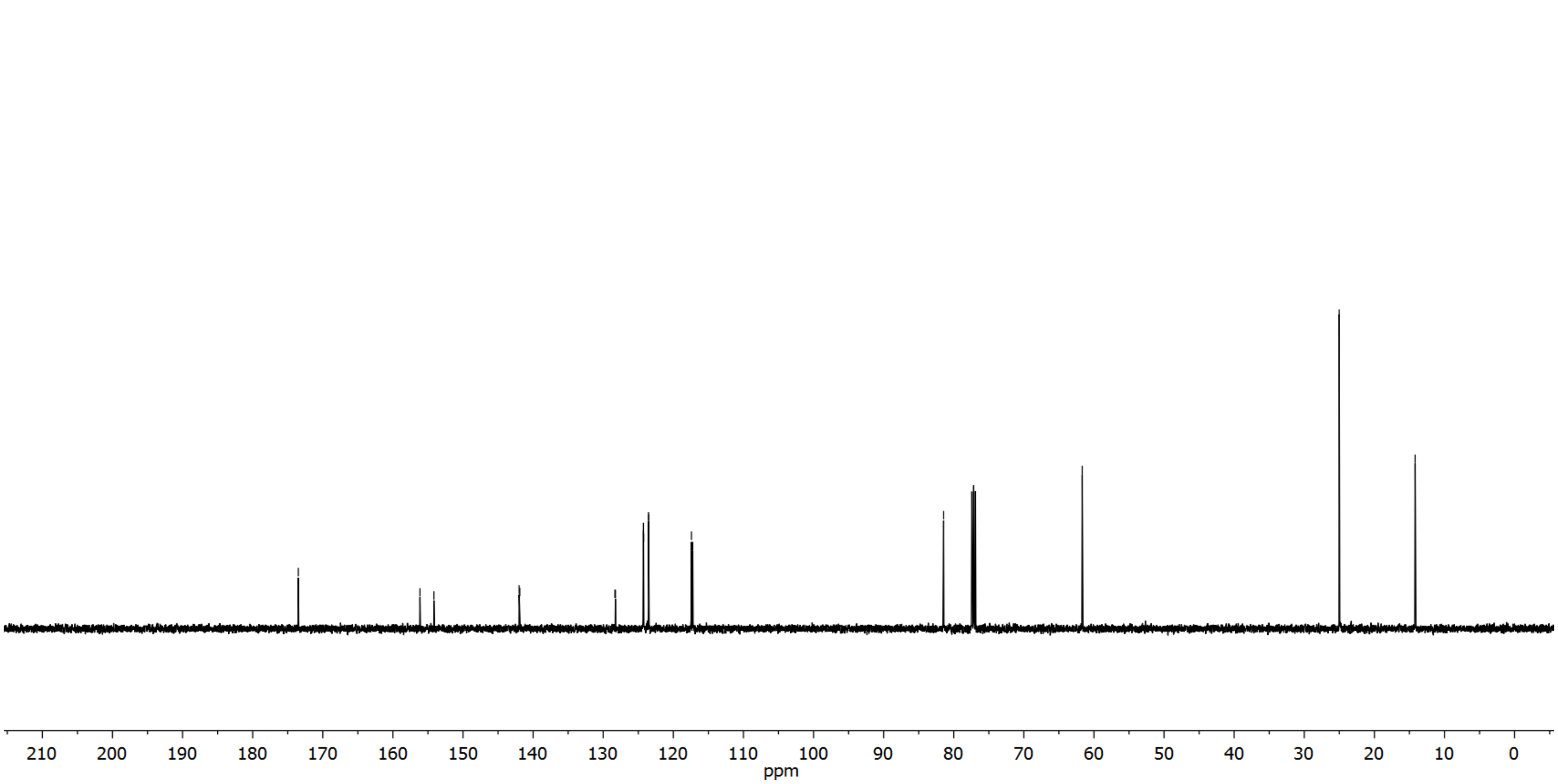

^{19}F NMR of fluoroclofibrate (S30)

$CDCl_3$, 298 K

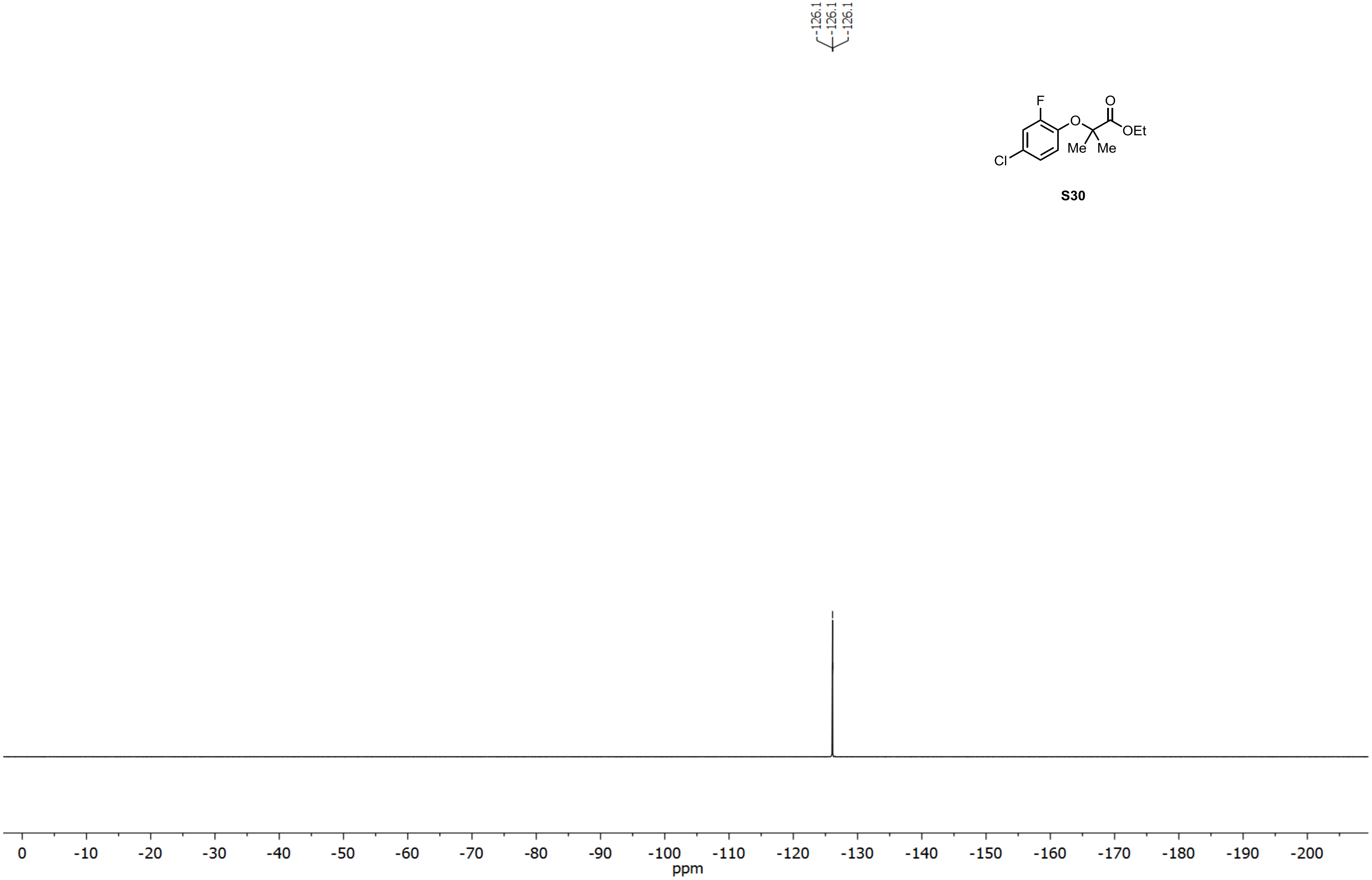

S30

^{1}H NMR of 3-chloro-9-fluoro-6-methyldibenzo[*c*,*f*][1,2]thiazepin-11(6*H*)-one 5,5-dioxide (S31)

CD_2Cl_2, 298 K

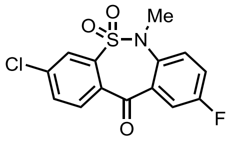

S31

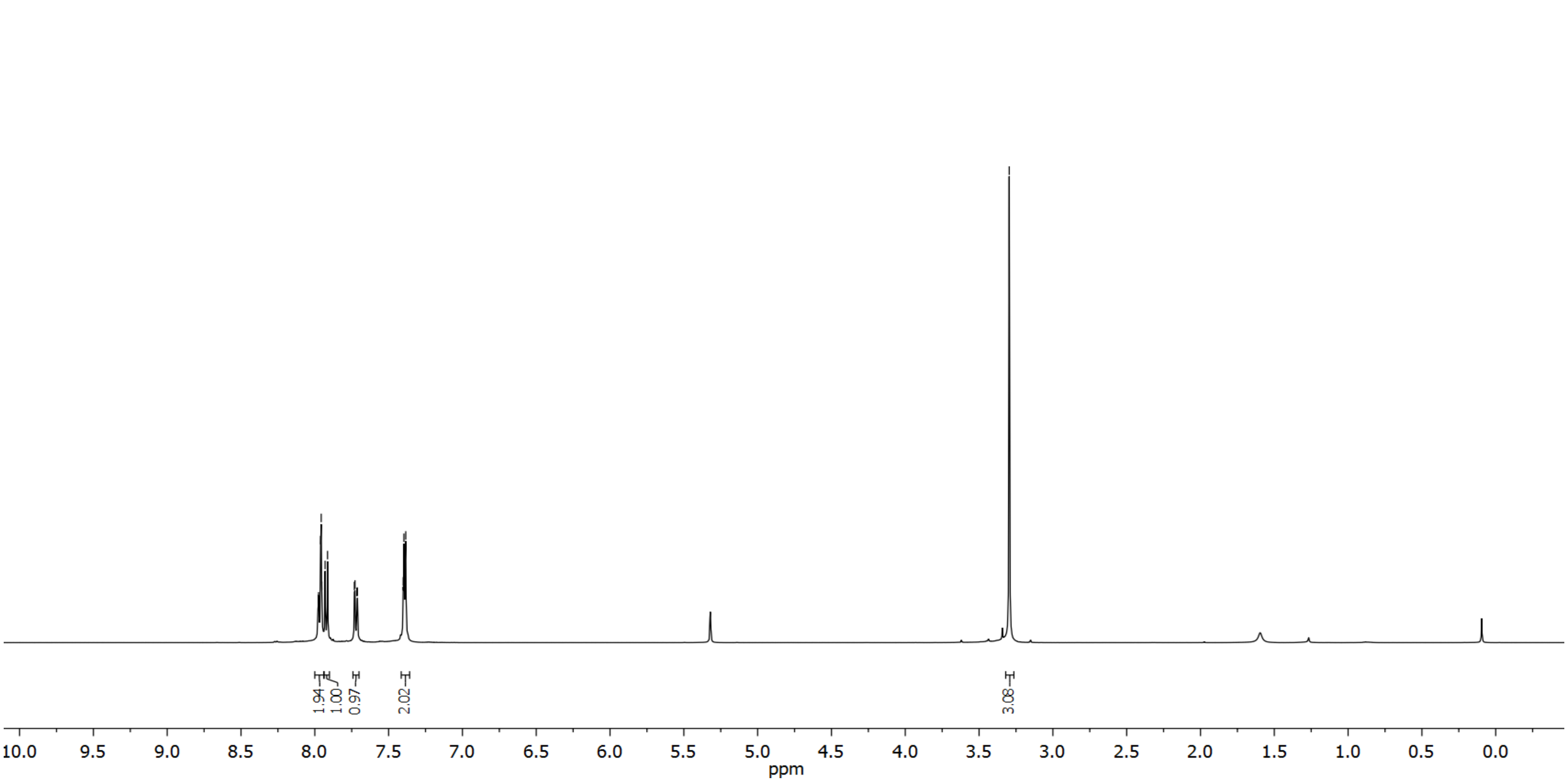

^{13}C NMR of 3-chloro-9-fluoro-6-methyldibenzo[*c*,*f*][1,2]thiazepin-11(6*H*)-one 5,5-dioxide (S31)

CD_2Cl_2, 298 K

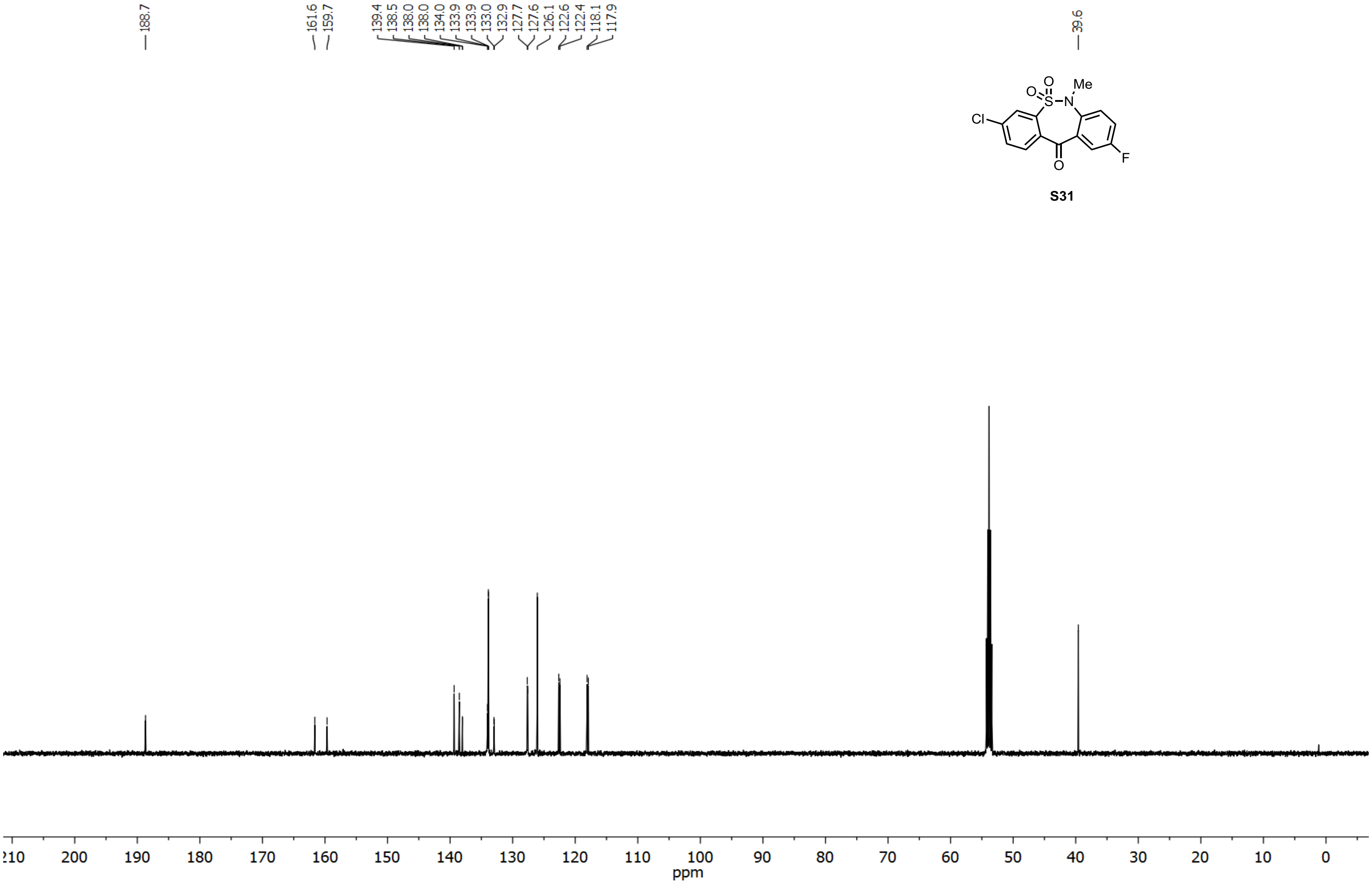

^{19}F NMR of 3-chloro-9-fluoro-6-methyldibenzo[*c*,*f*][1,2]thiazepin-11(6*H*)-one 5,5-dioxide (S31)

CD_2Cl_2, 298 K

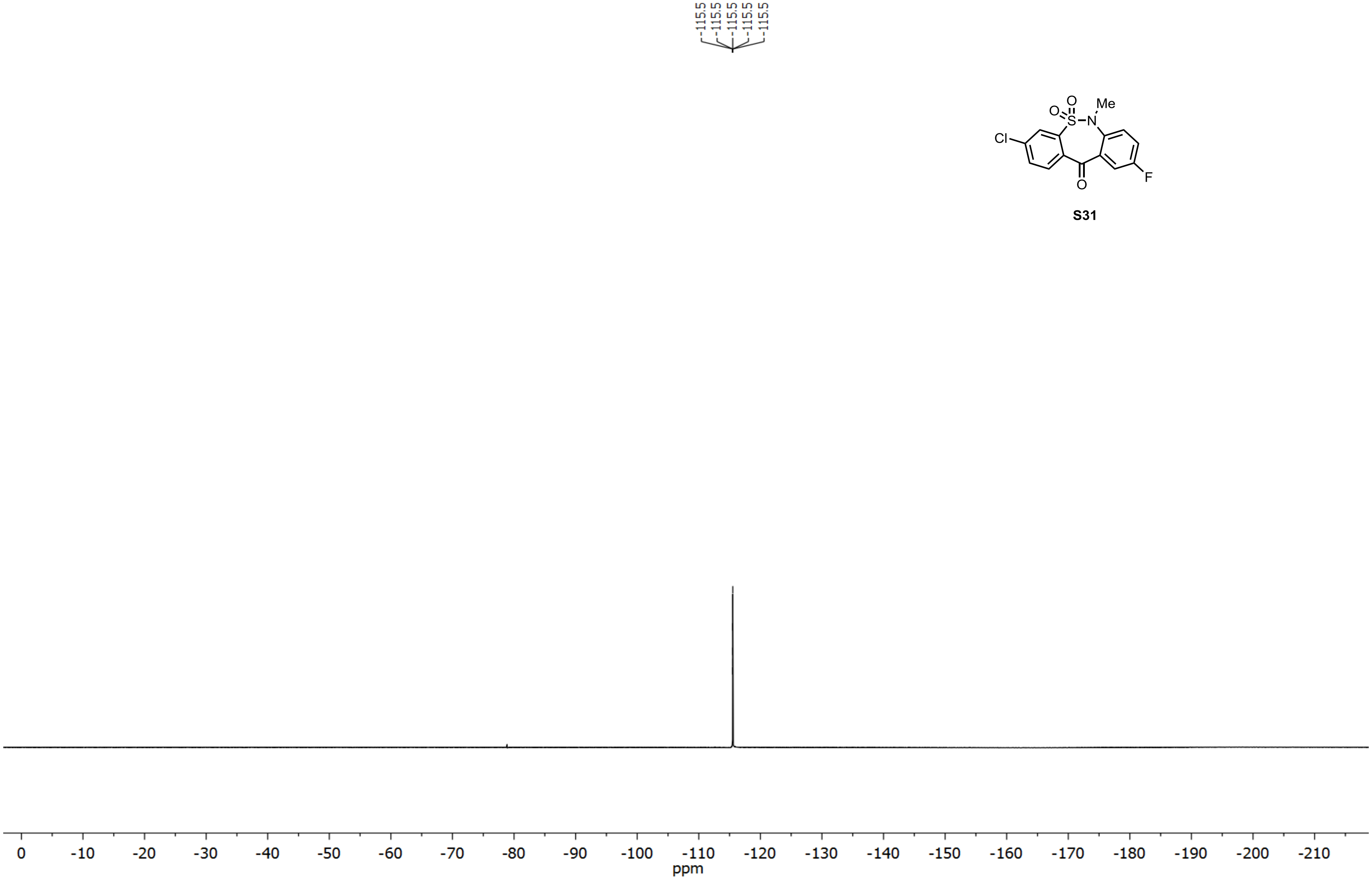

S31

^{1}H NMR of fluorodicamba methylester (S32)

$CDCl_3$, 298 K

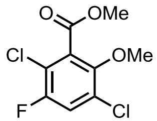

S32

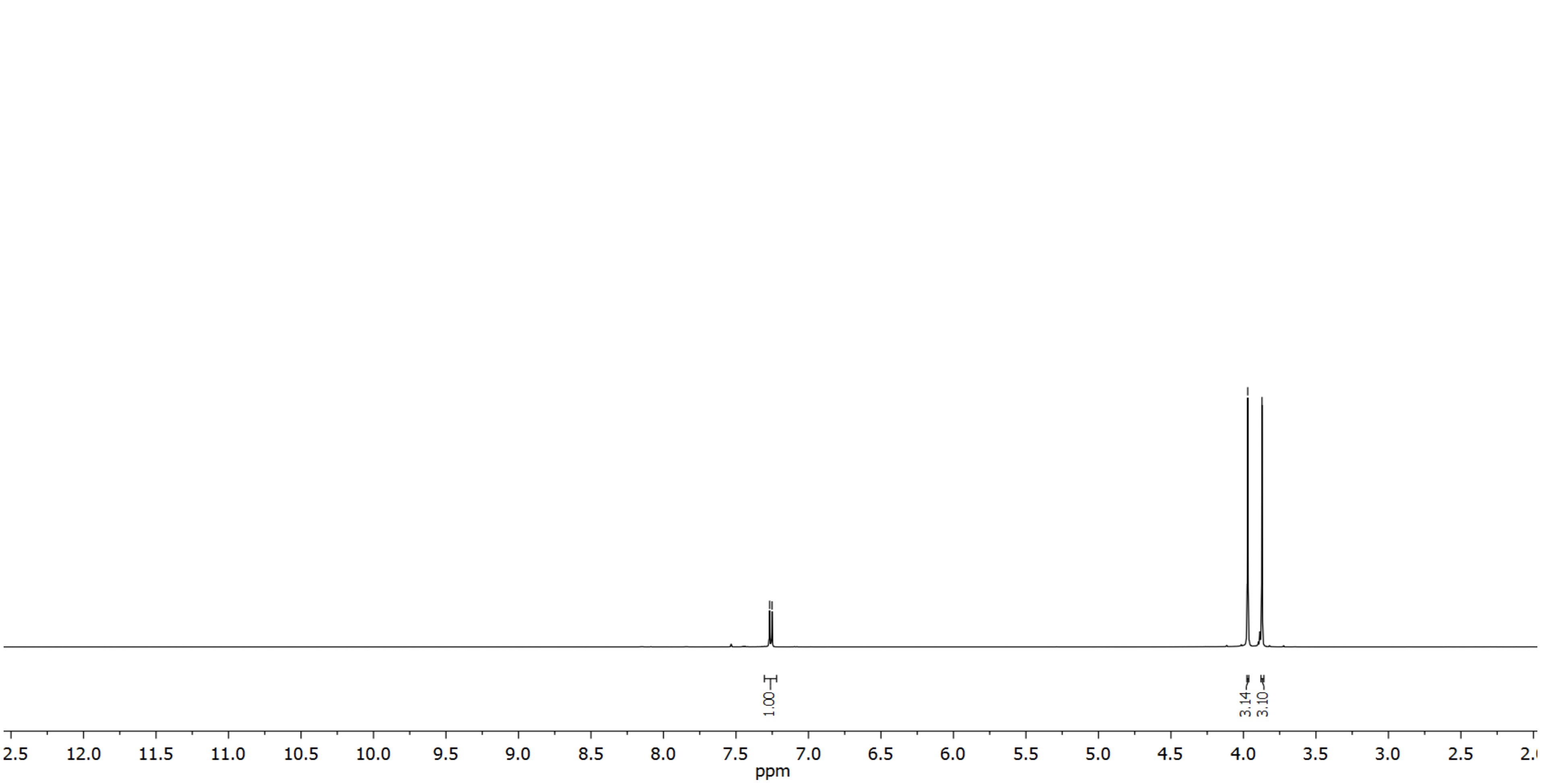

^{13}C NMR of fluorodicamba methylester (S32)

$CDCl_3$, 298 K

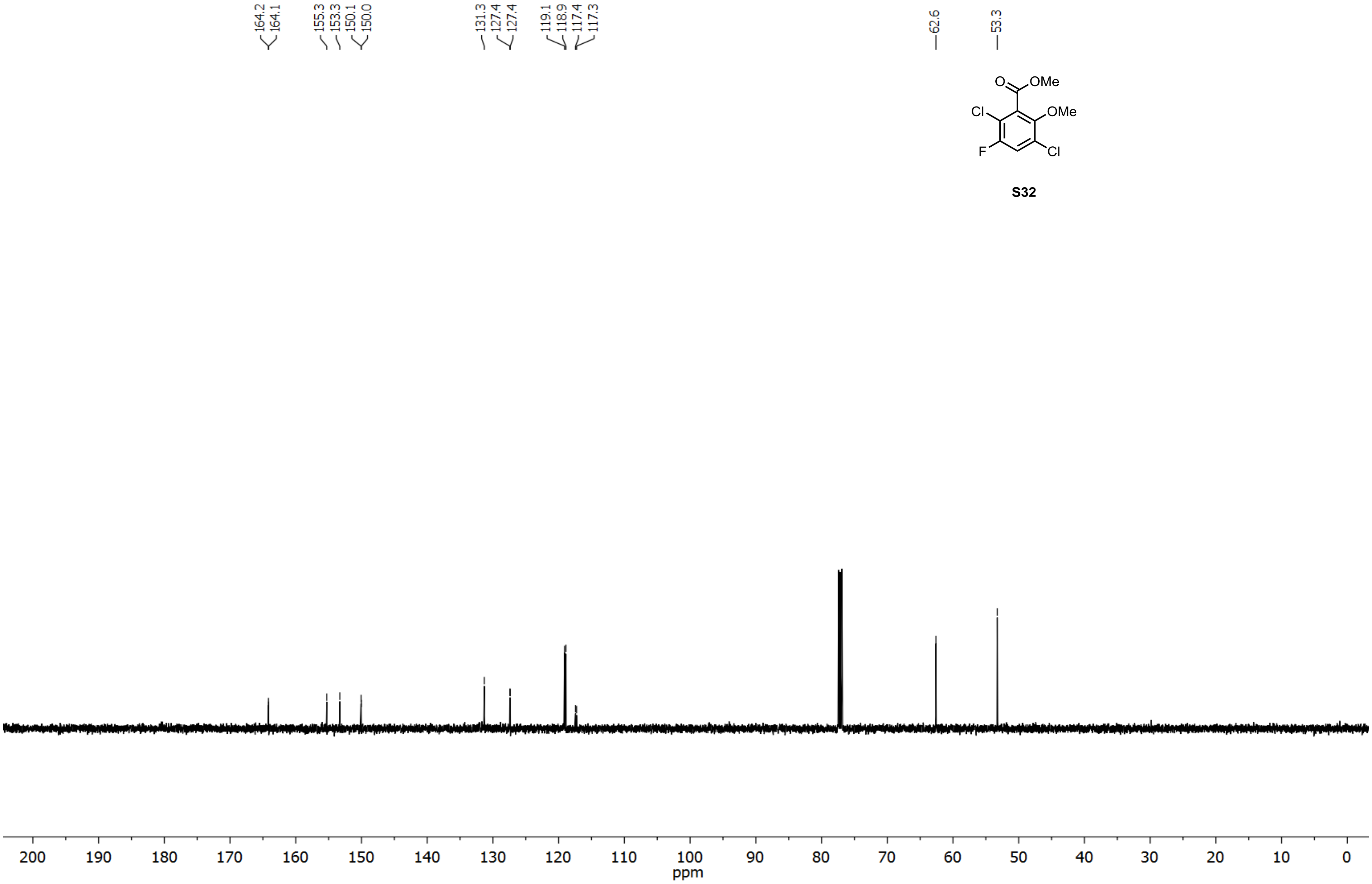

^{19}F NMR of fluorodicamba methylester (S32)

$CDCl_3$, 298 K

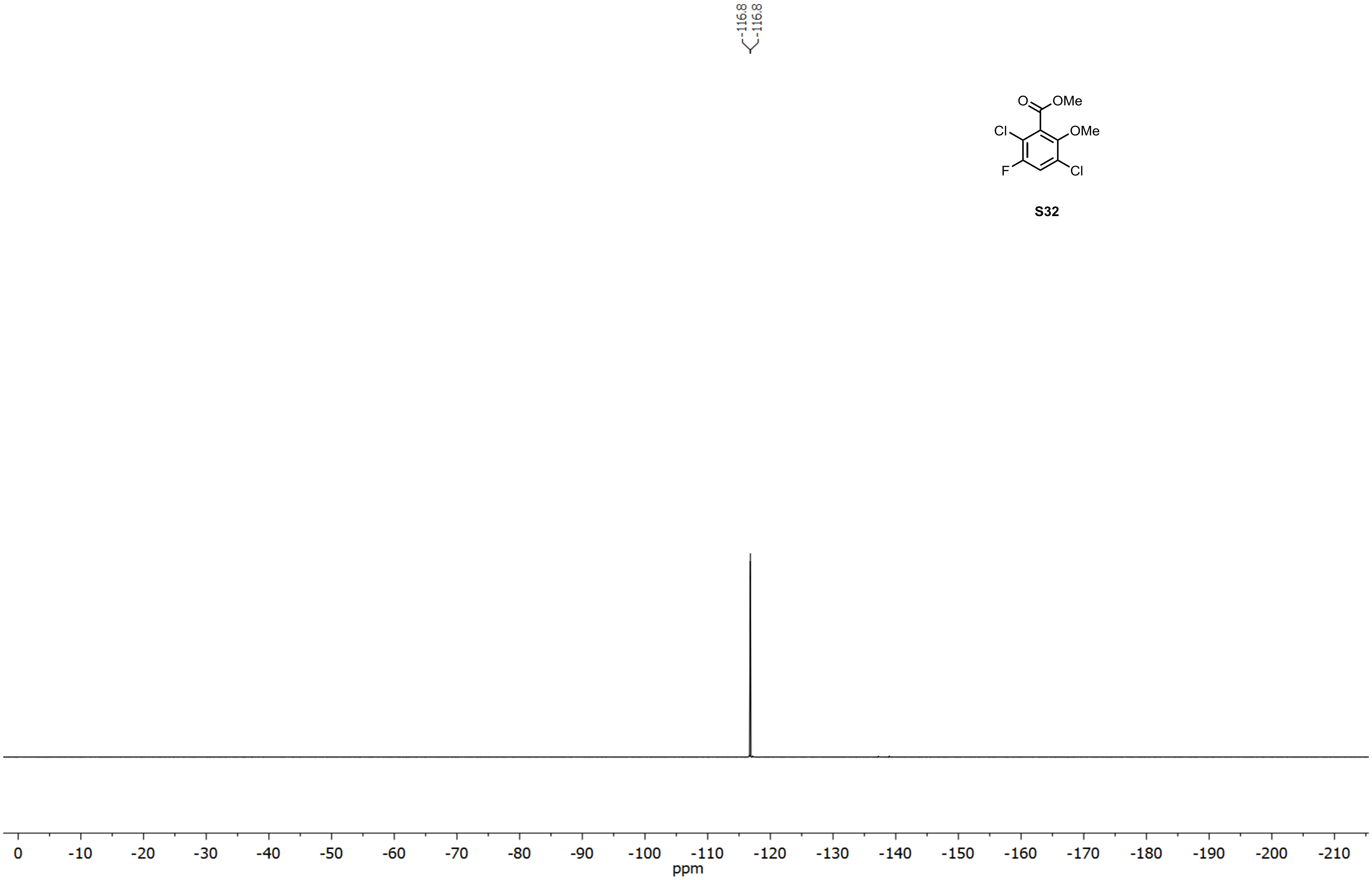

S32

^{1}H NMR of fluorobifonazole (S33)

CD_2Cl_2, 298 K

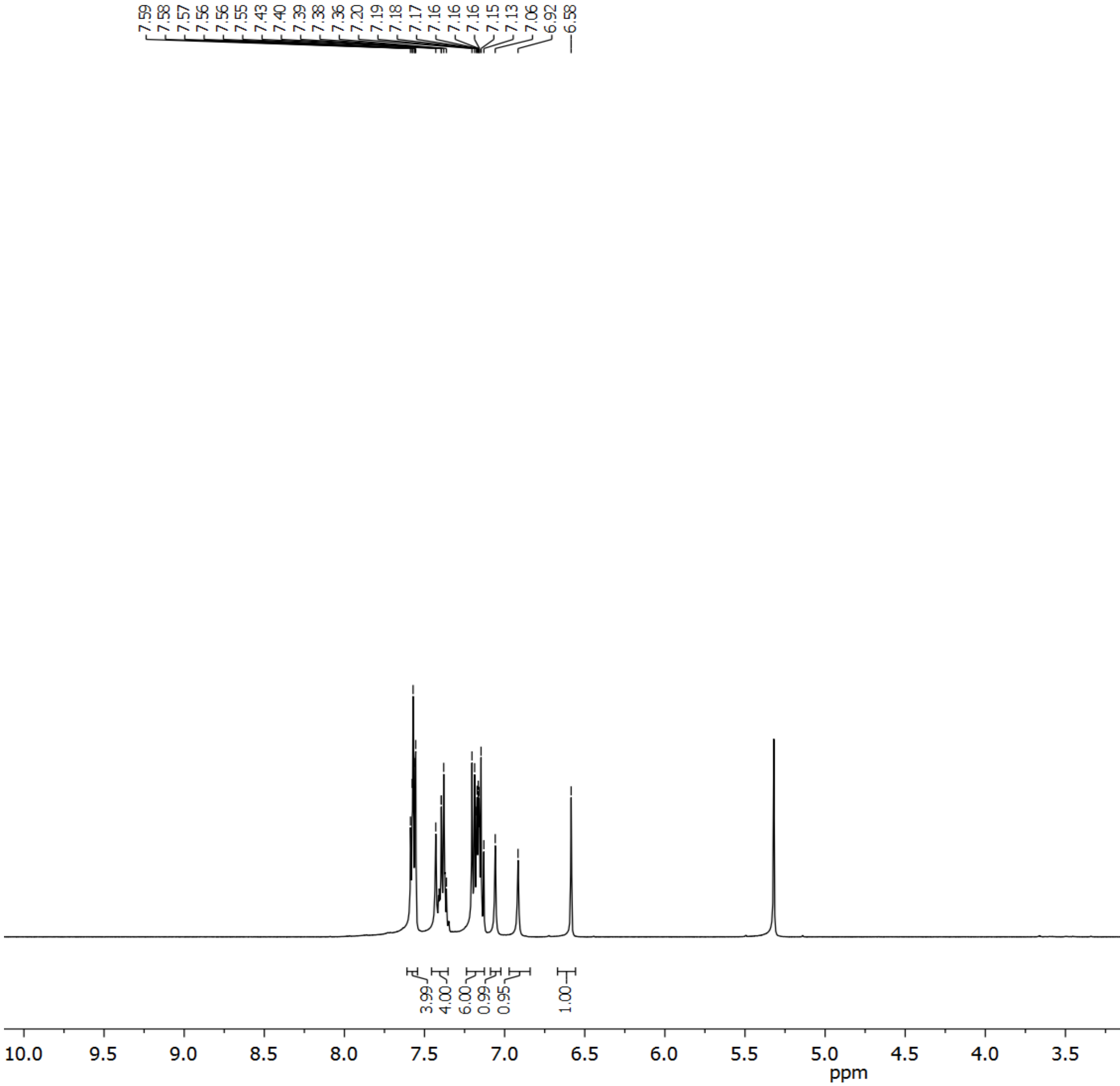

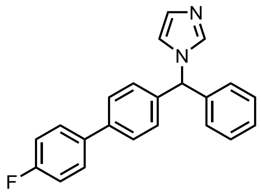

S33

^{13}C NMR of fluorobifonazole (S33)

CD_2Cl_2, 298 K

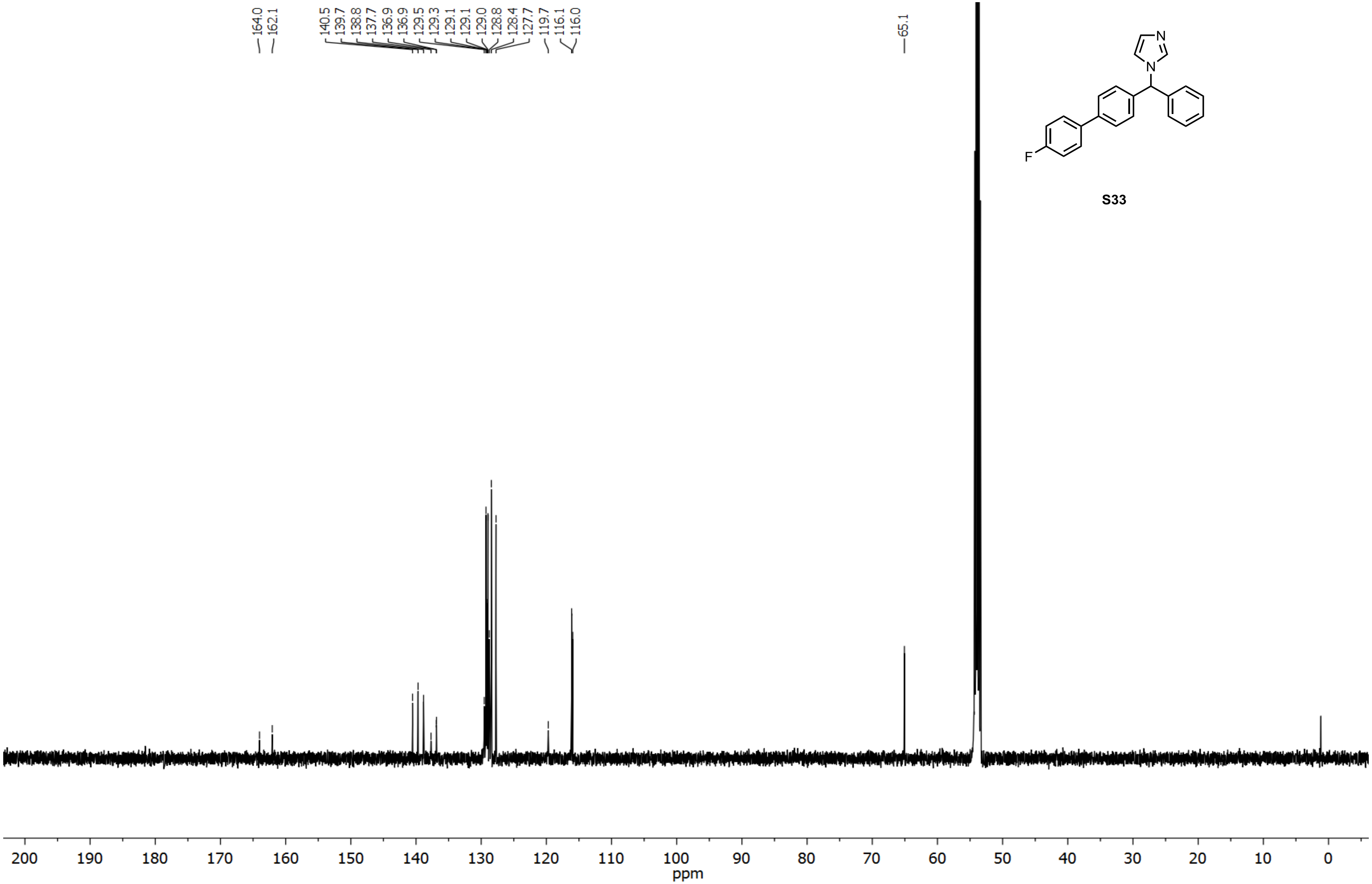

S33

^{19}F NMR of fluorobifonazole (S33)

CD_2Cl_2, 298 K

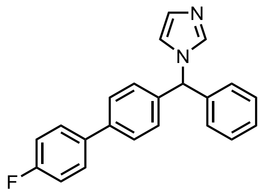

S33

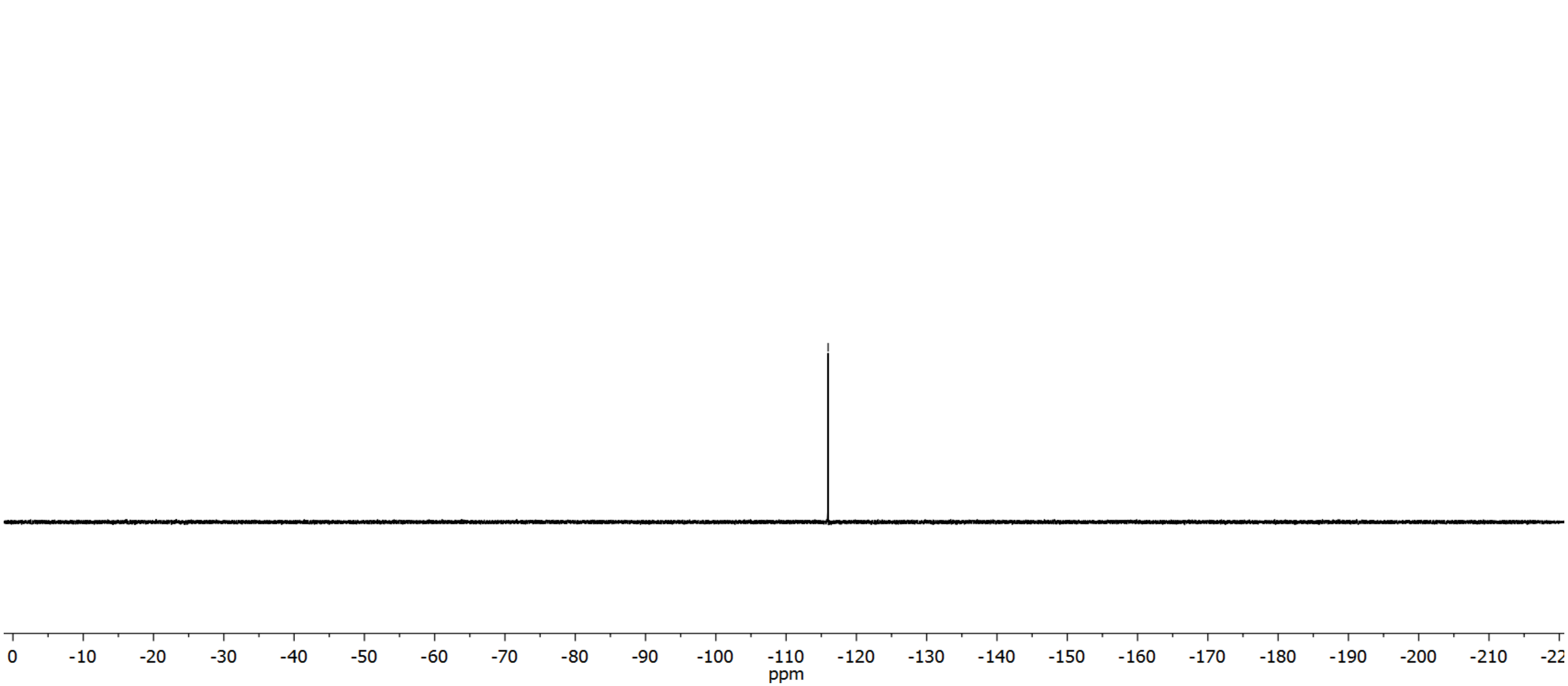

^{1}H NMR of fluorofenofibrate (S37)

$CDCl_3$, 298 K

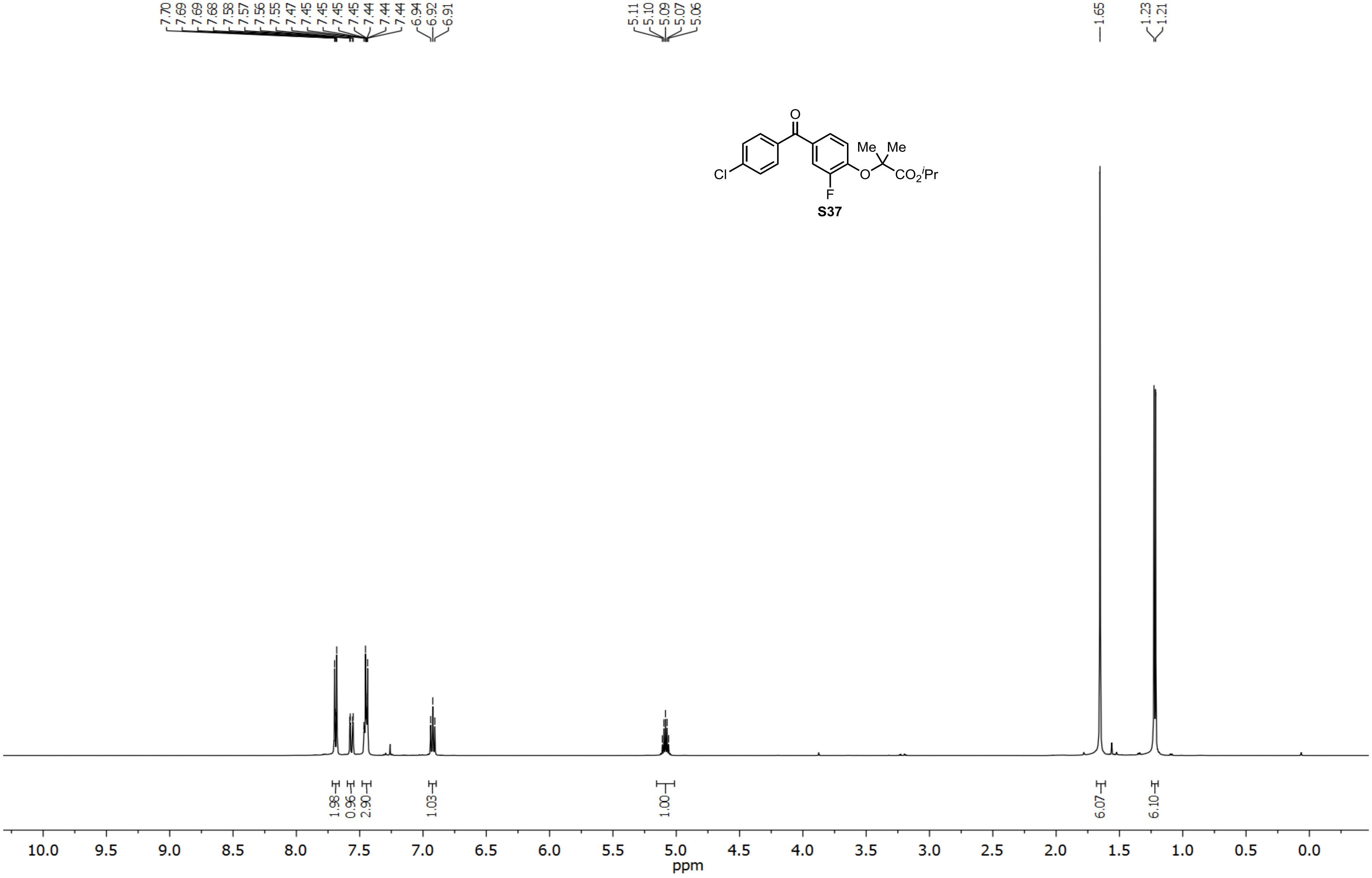

^{13}C NMR of fluorofenofibrate (S37)

$CDCl_3$, 298 K

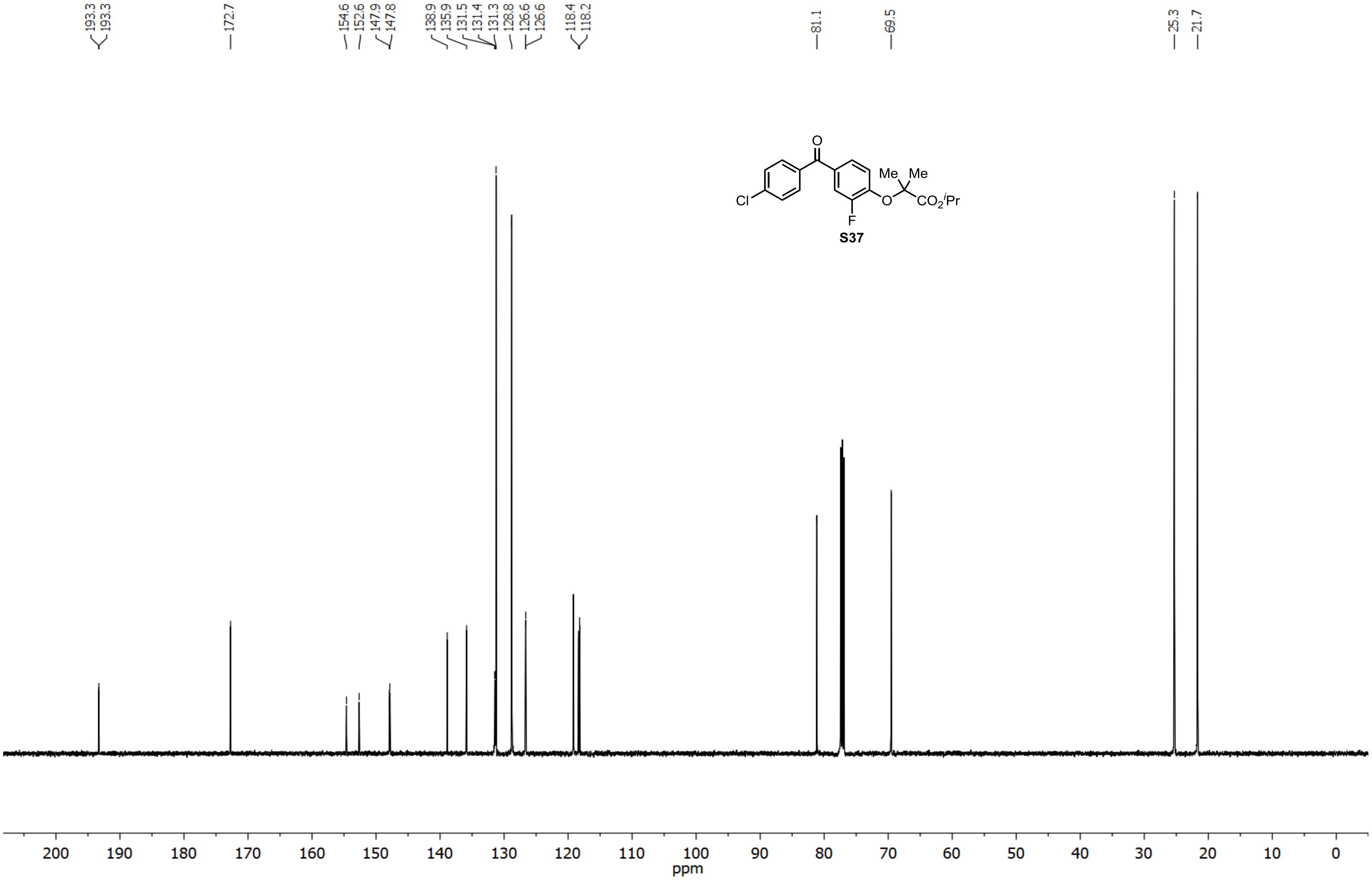

^{19}F NMR of fluorofenofibrate (S37)

$CDCl_3$, 298 K

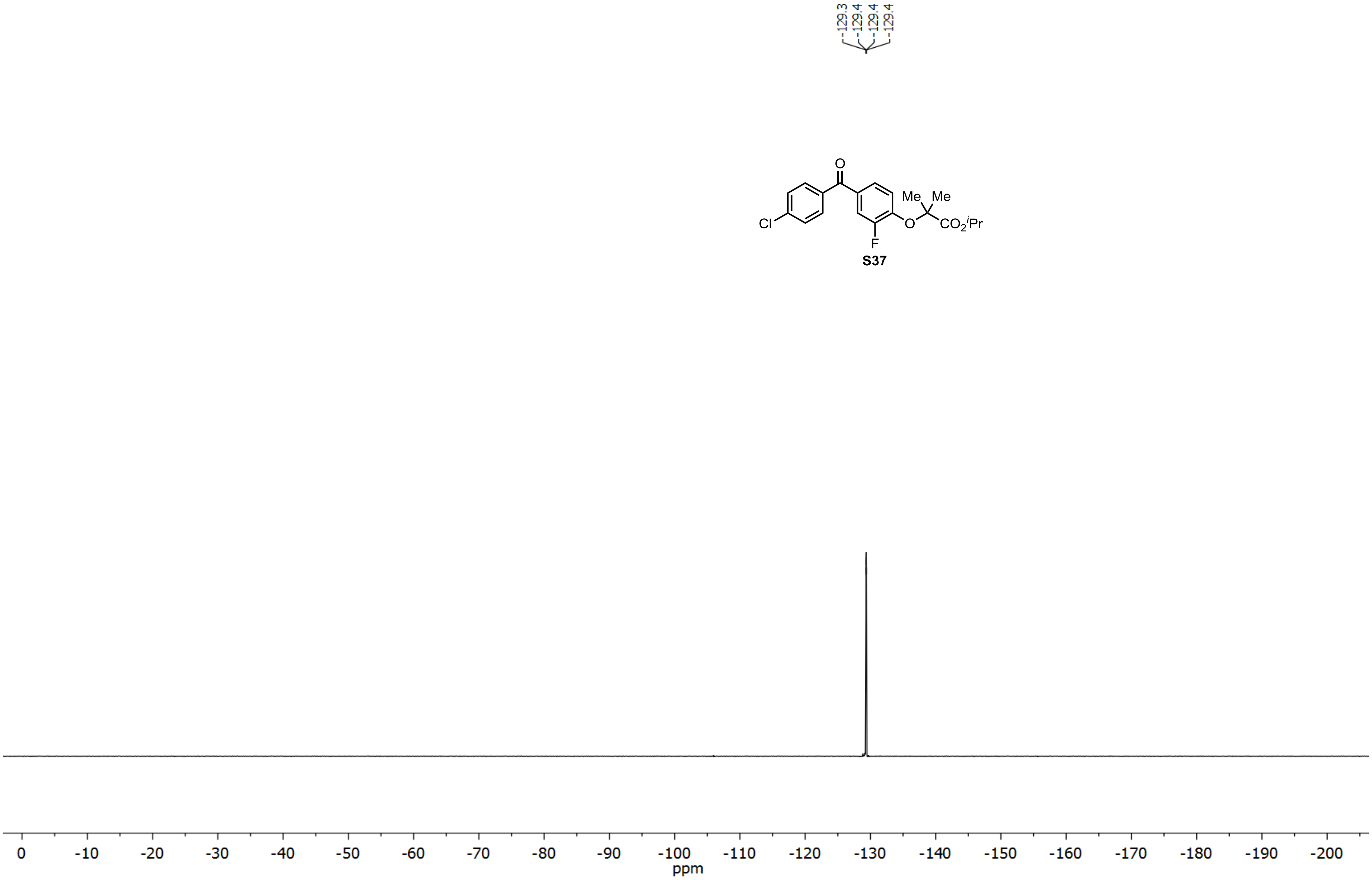

^{1}H NMR of anisole-derived 2,8-dimethoxydibenzothiophenium salt S39

$CDCl_3$, 298 K

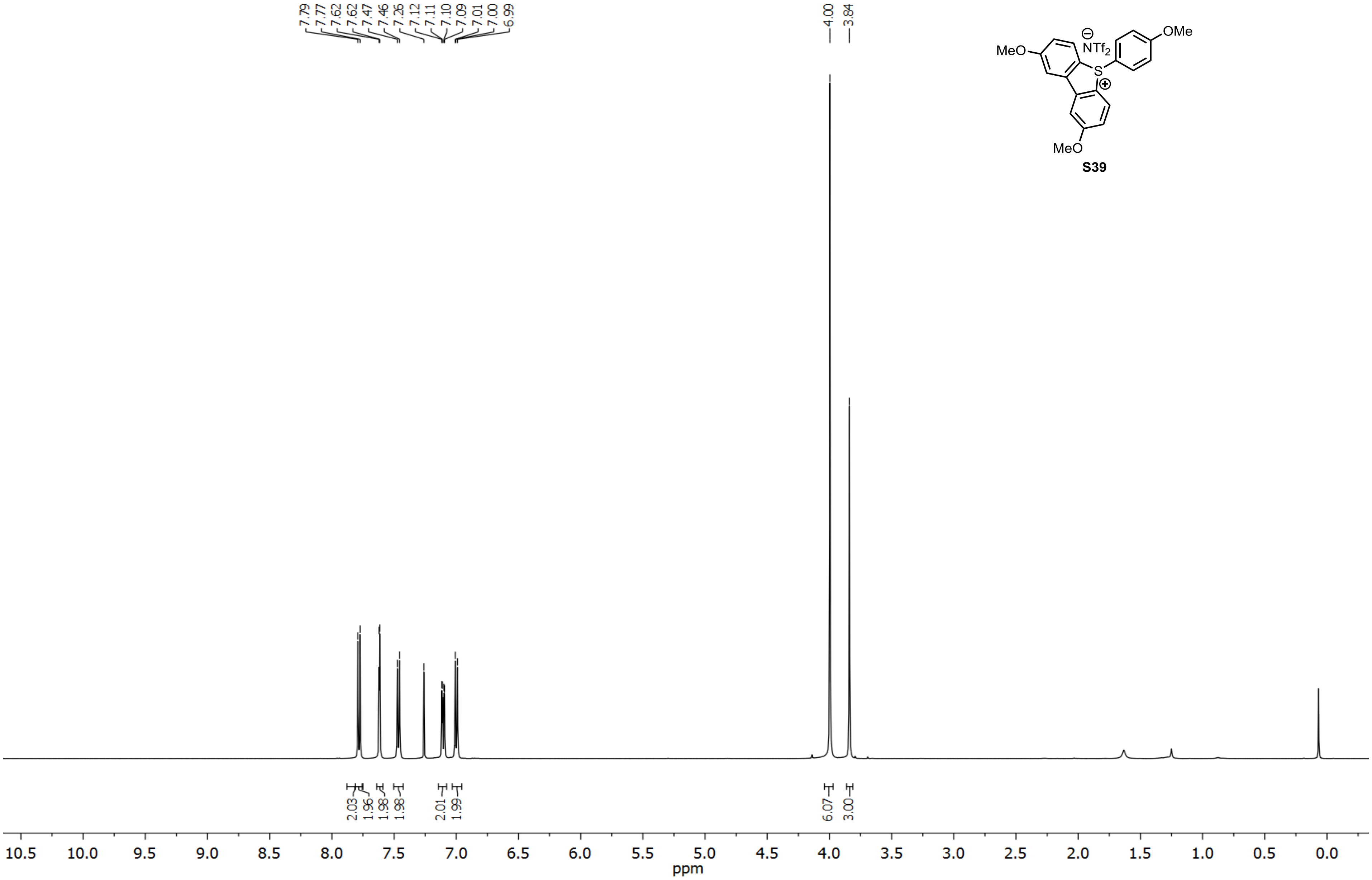

^{13}C NMR of anisole-derived 2,8-dimethoxydibenzothiophenium salt S39

CDCl$_3$, 298 K

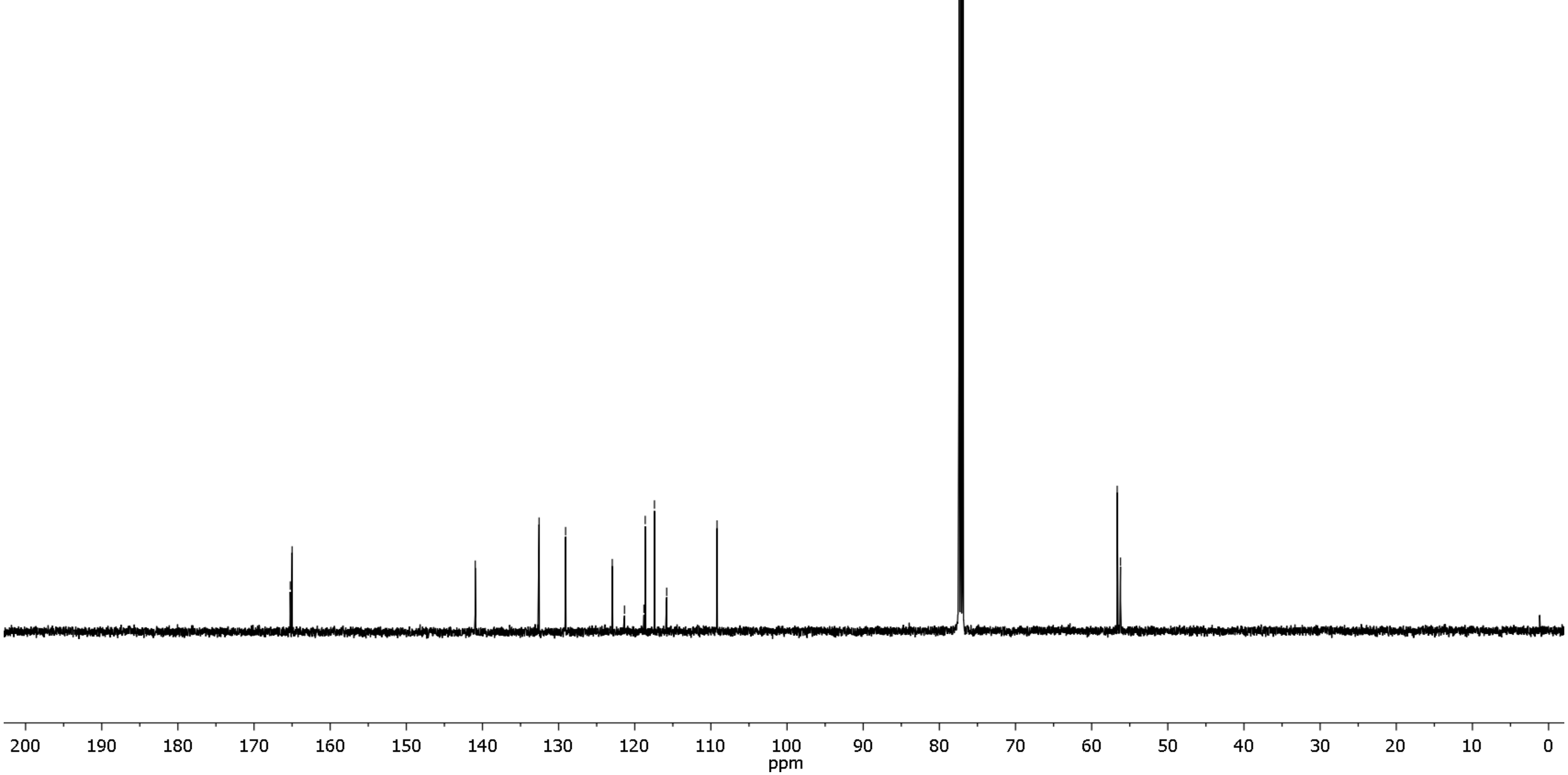

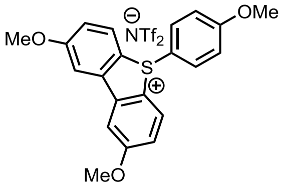

S39

^{19}F NMR of anisole-derived 2,8-dimethoxydibenzothiophenium salt S39

$CDCl_3$, 298 K

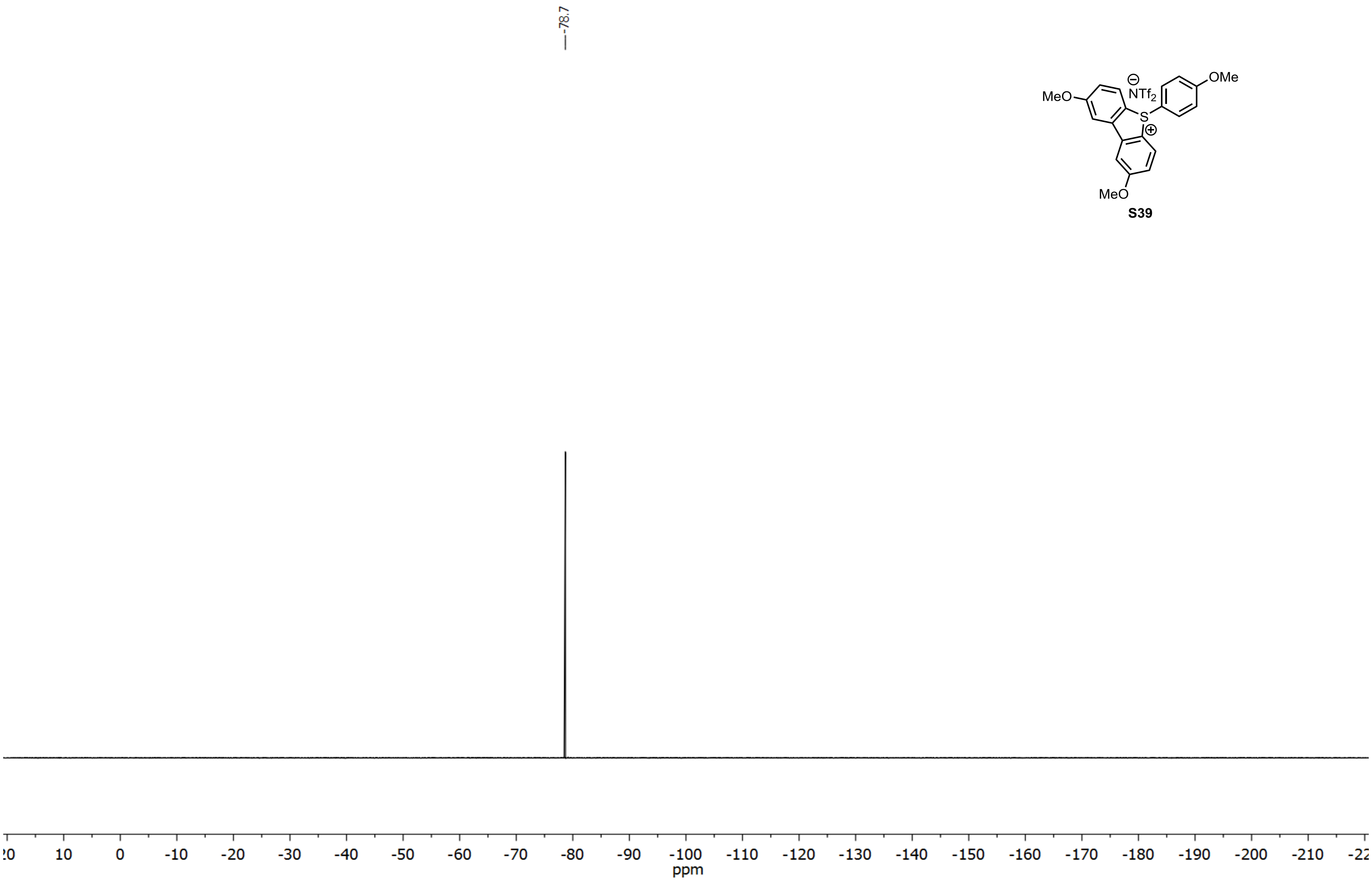

^{1}H NMR of toluene-derived 2,8-dimethoxydibenzothiophenium salt S40

CD$_3$CN, 298 K

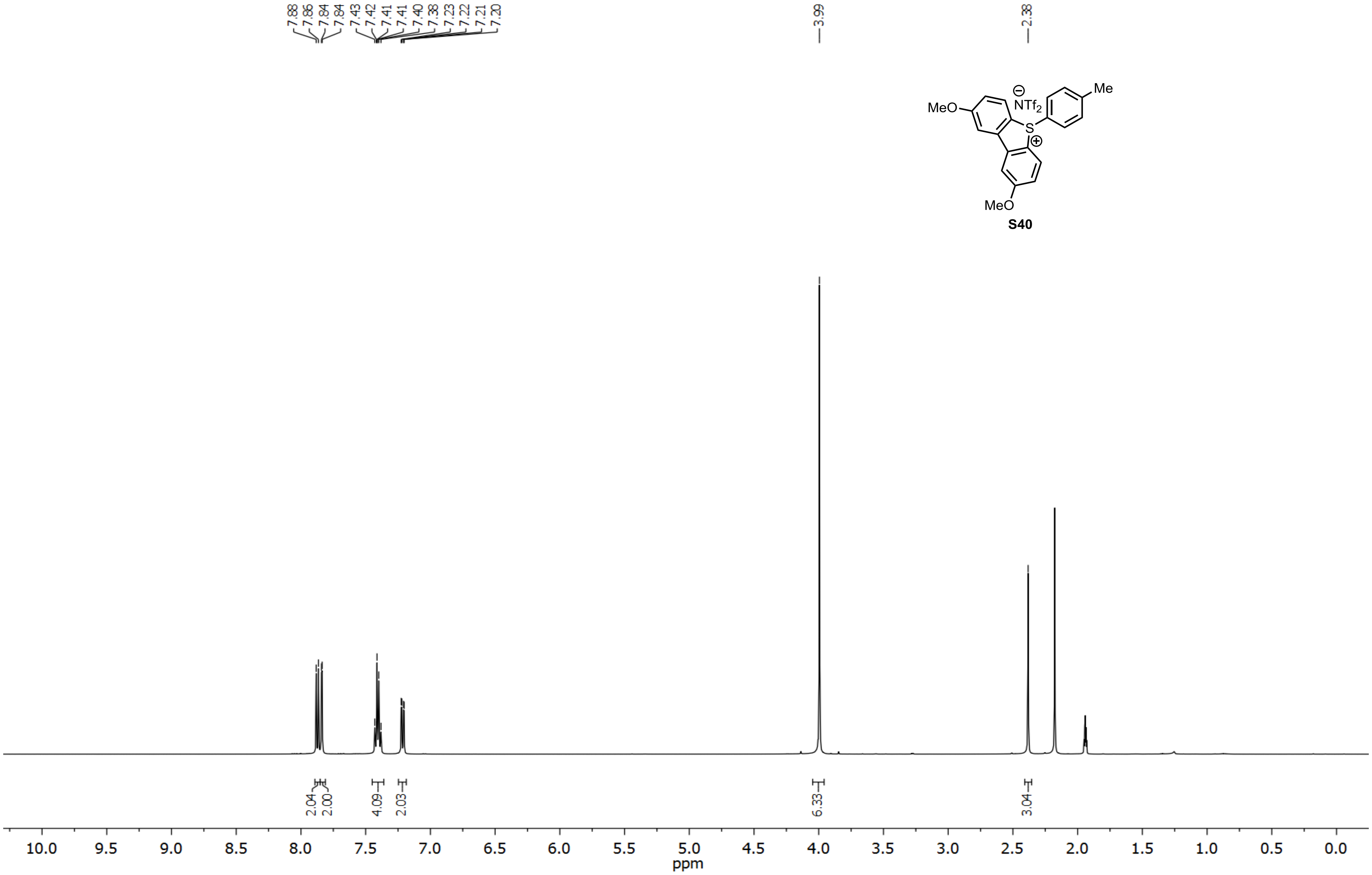

^{13}C NMR of toluene-derived 2,8-dimethoxydibenzothiophenium salt S40

CD_3CN, 298 K

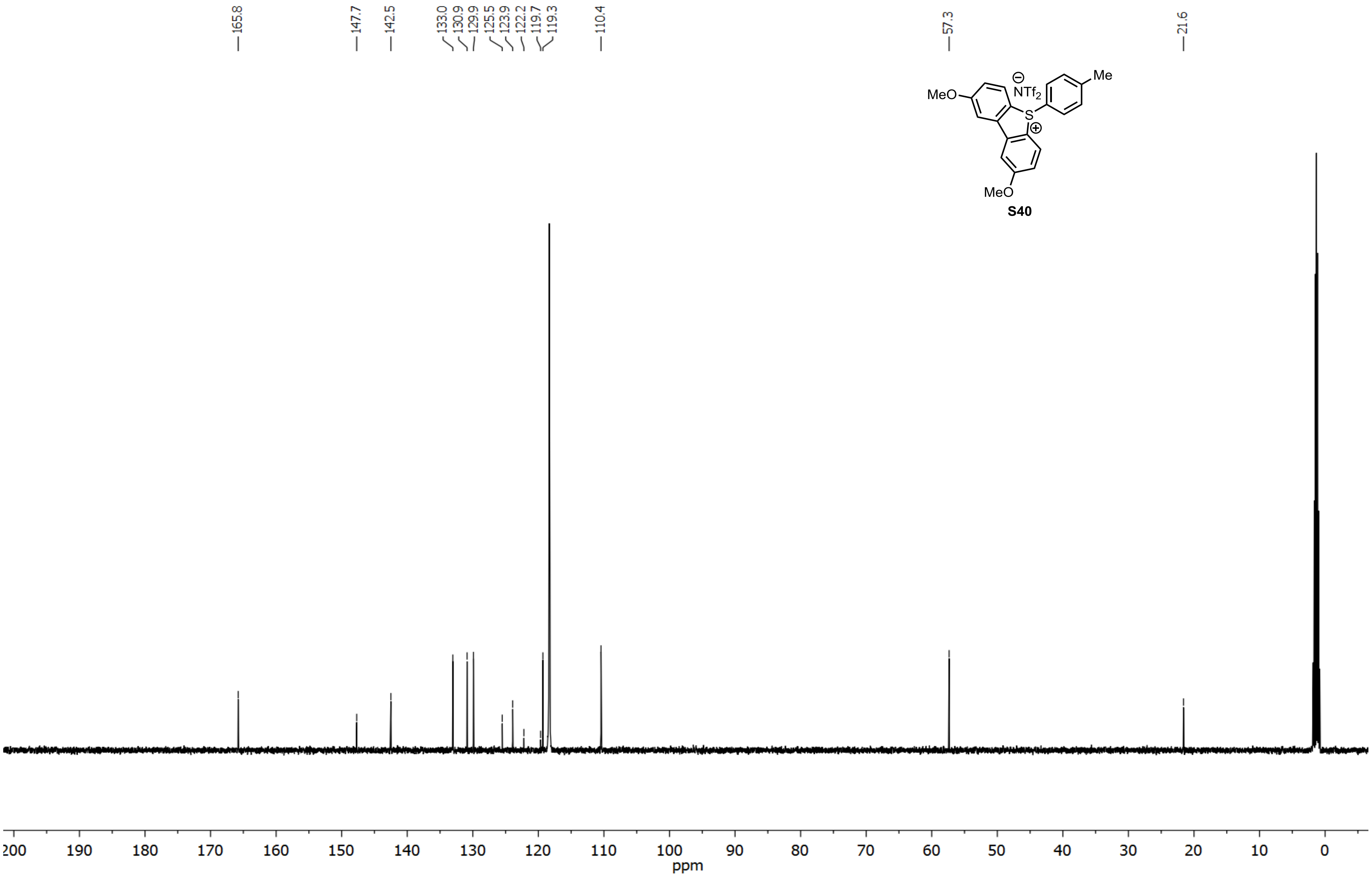

^{19}F NMR of toluene-derived 2,8-dimethoxydibenzothiophenium salt S40

CD_3CN, 298 K

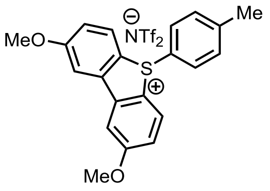

S40

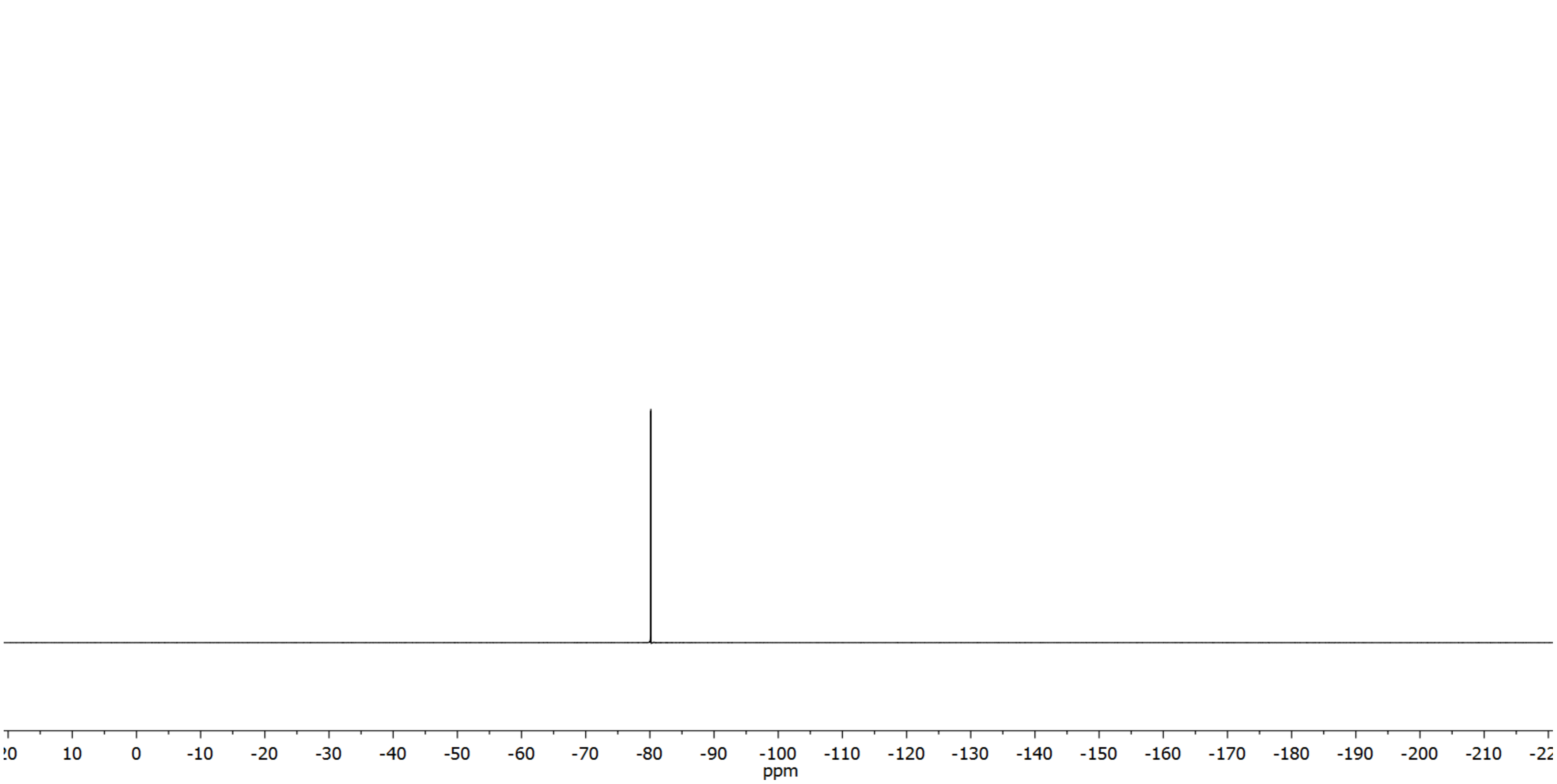

^{1}H NMR of benzene-derived 2,8-dimethoxydibenzothiophenium salt S41

$CDCl_3$, 298 K

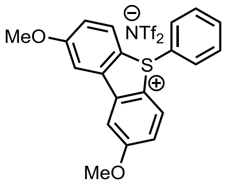

S41

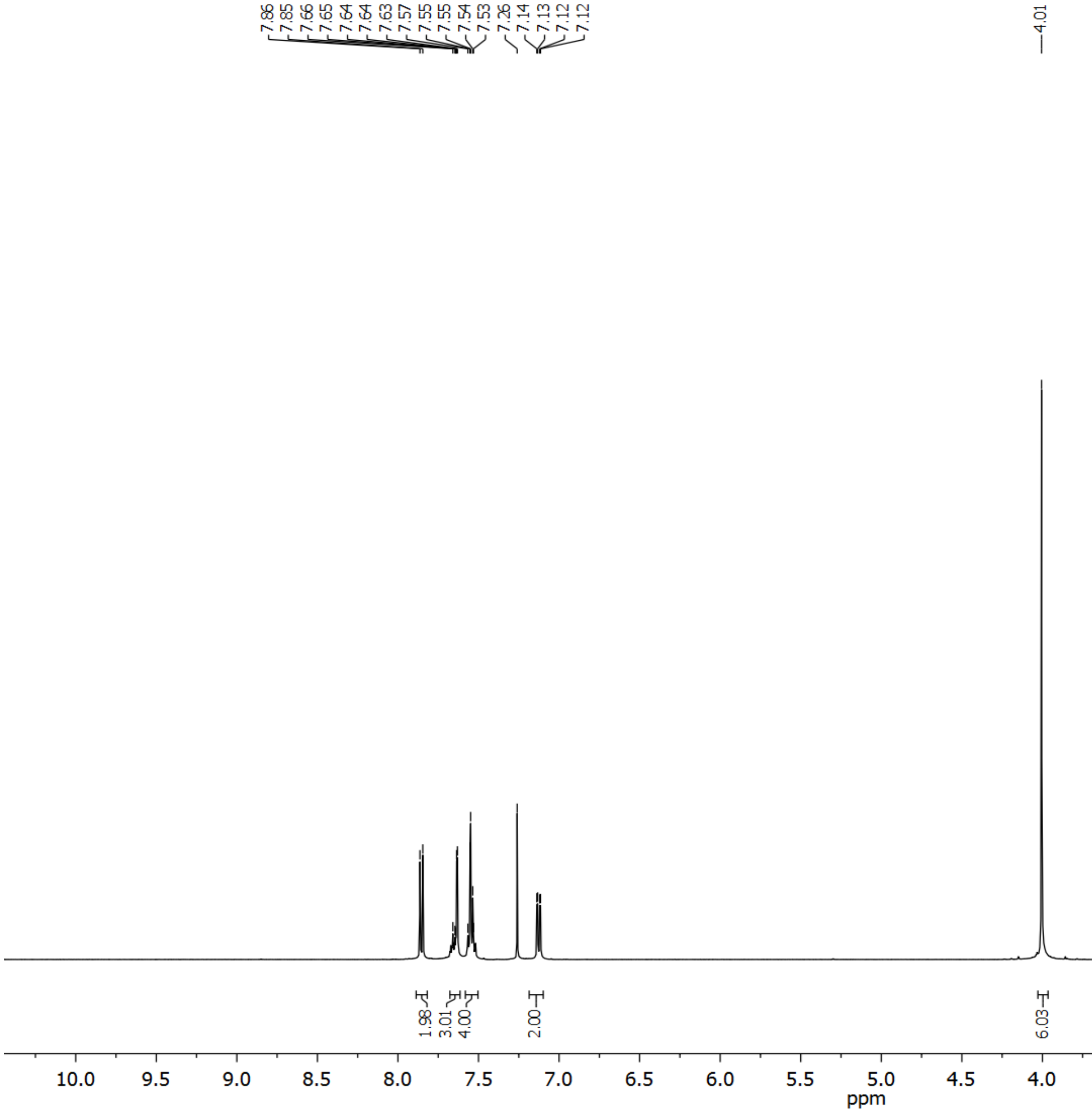

^{13}C NMR of benzene-derived 2,8-dimethoxydibenzothiophenium salt S41

CDCl$_3$, 298 K

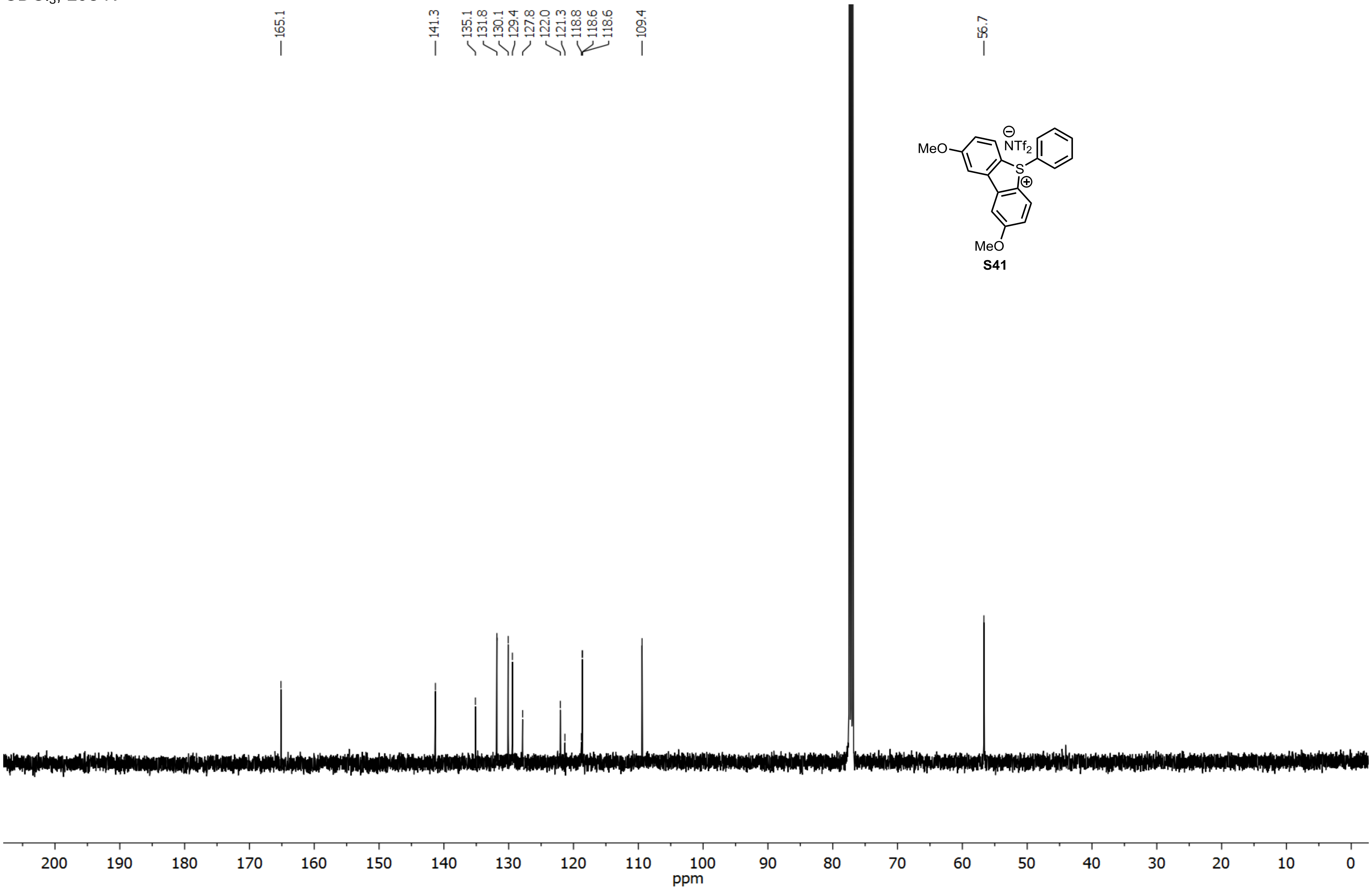

^{19}F NMR of benzene-derived 2,8-dimethoxydibenzothiophenium salt S41

$CDCl_3$, 298 K

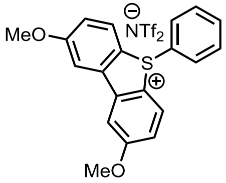

S41

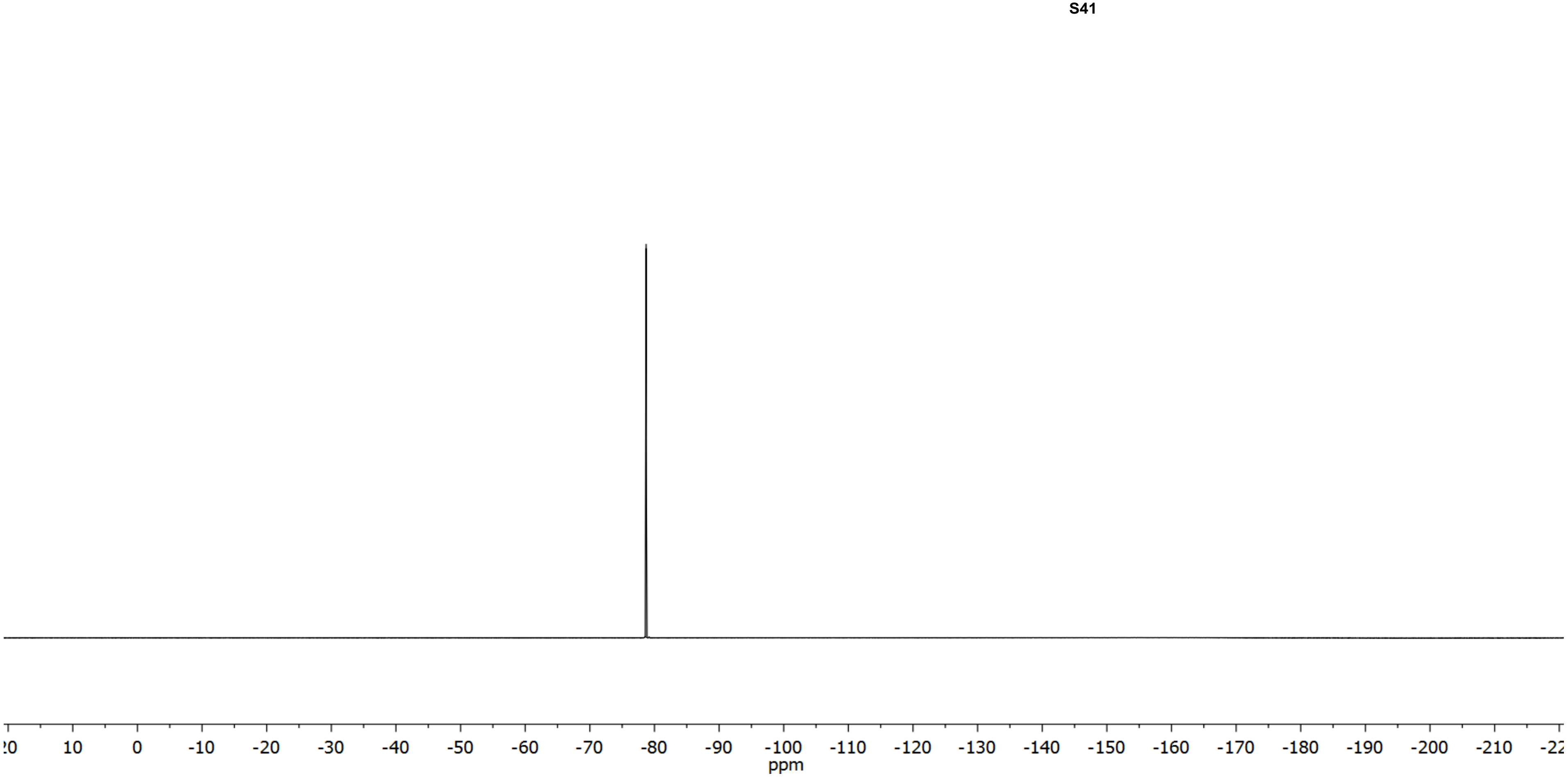

^{1}H NMR of fluorobenzene-derived 2,8-dimethoxydibenzothiophenium salt S42

CD_2Cl_2, 298 K

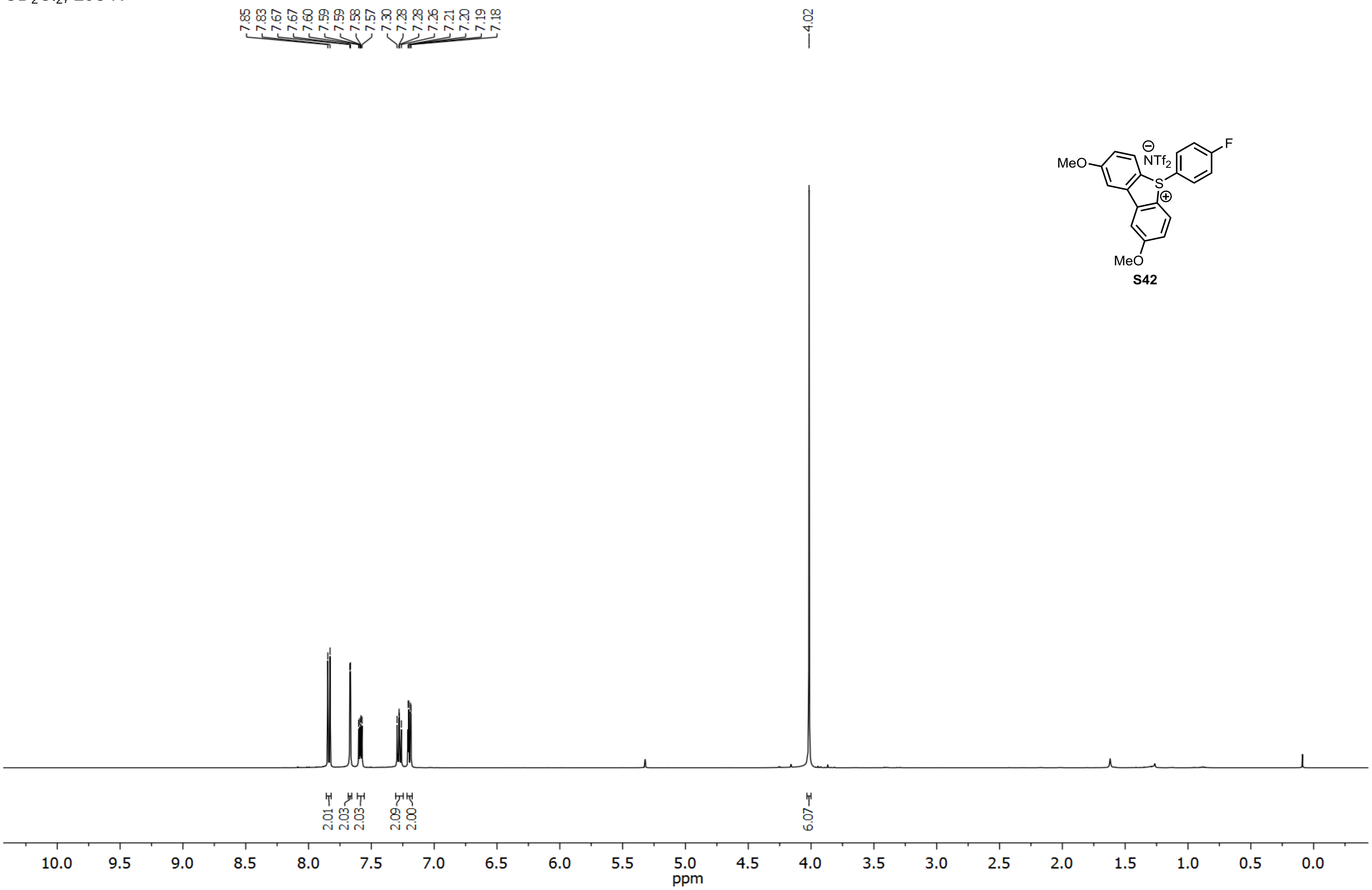

^{13}C NMR of fluorobenzene-derived 2,8-dimethoxydibenzothiophenium salt S42

CD_2Cl_2, 298 K

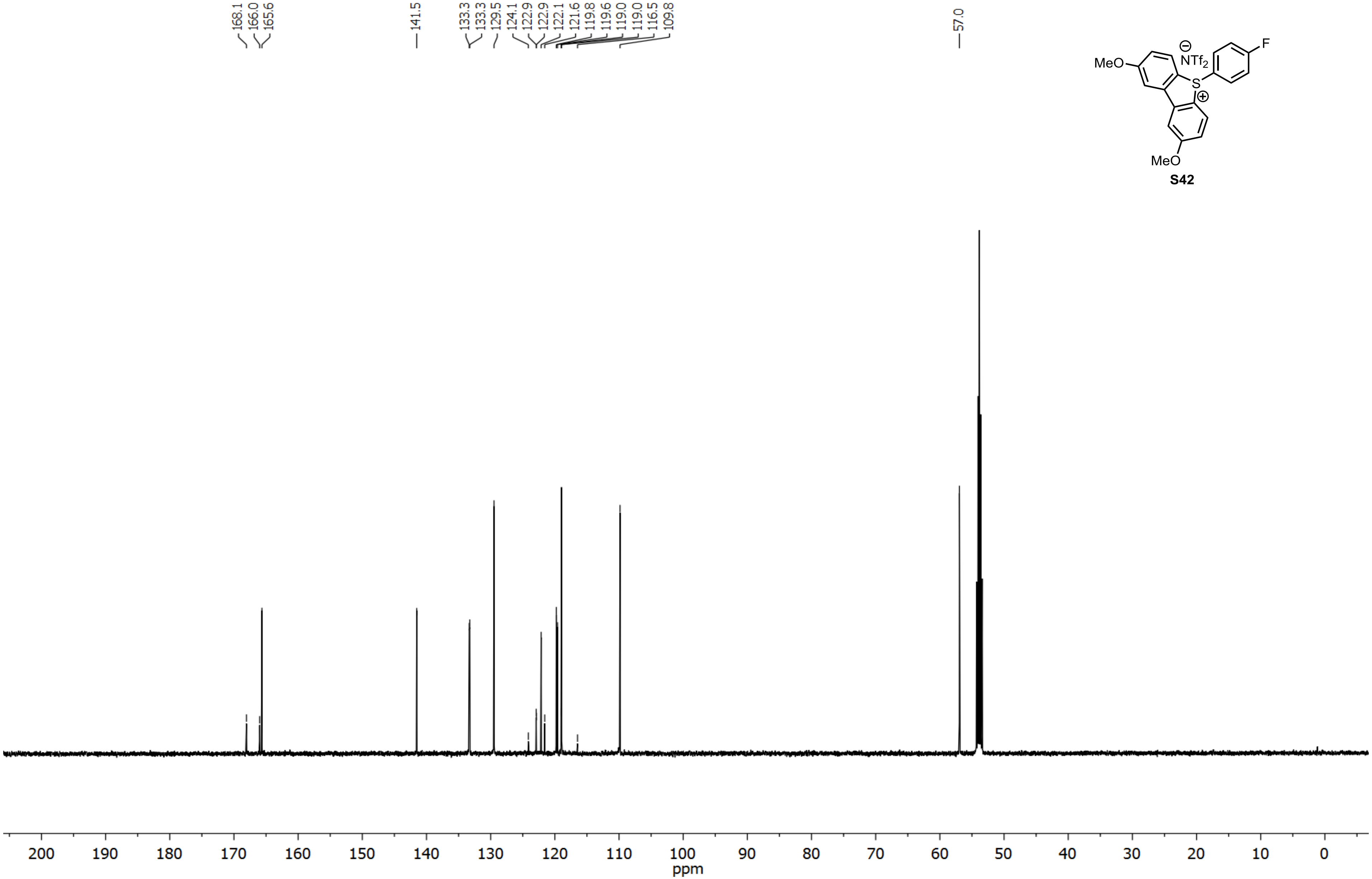

^{19}F NMR of fluorobenzene-derived 2,8-dimethoxydibenzothiophenium salt S42

CD_2Cl_2, 298 K

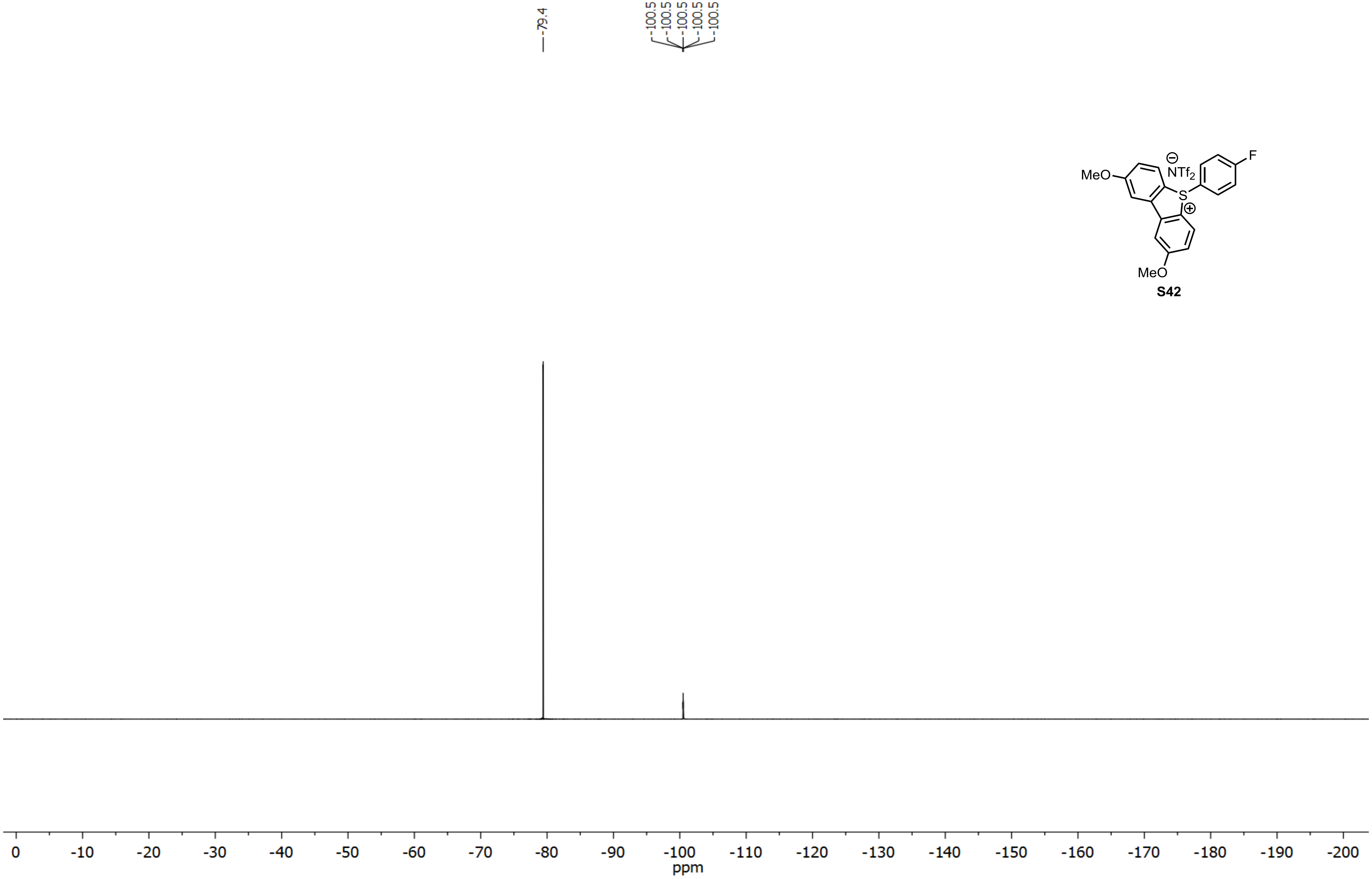

S42

^{1}H NMR of chlorobenzene-derived 2,8-dimethoxydibenzothiophenium salt S43

CD_2Cl_2, 298 K

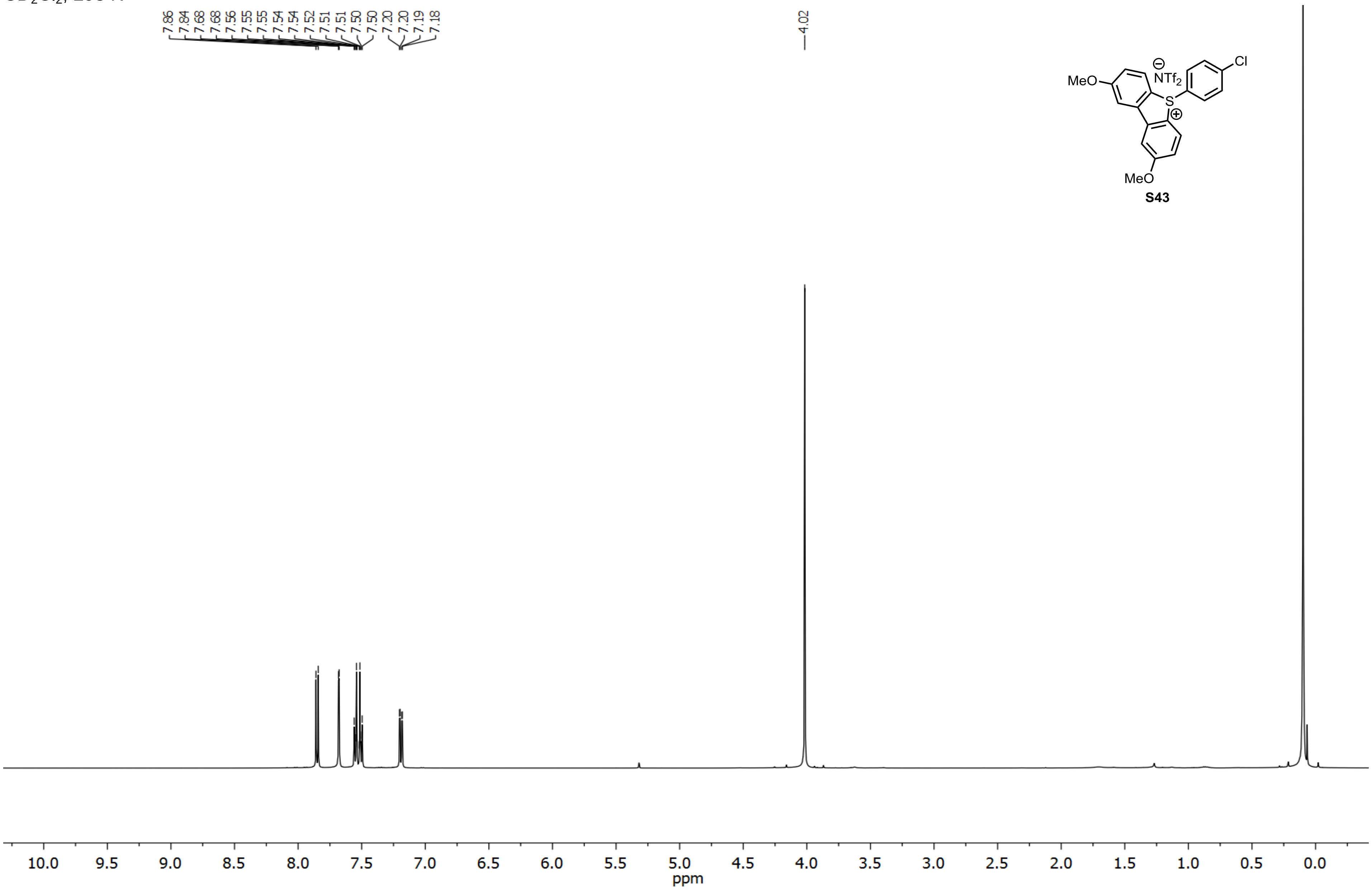

^{13}C NMR of chlorobenzene-derived 2,8-dimethoxydibenzothiophenium salt S43

CD_2Cl_2, 298 K

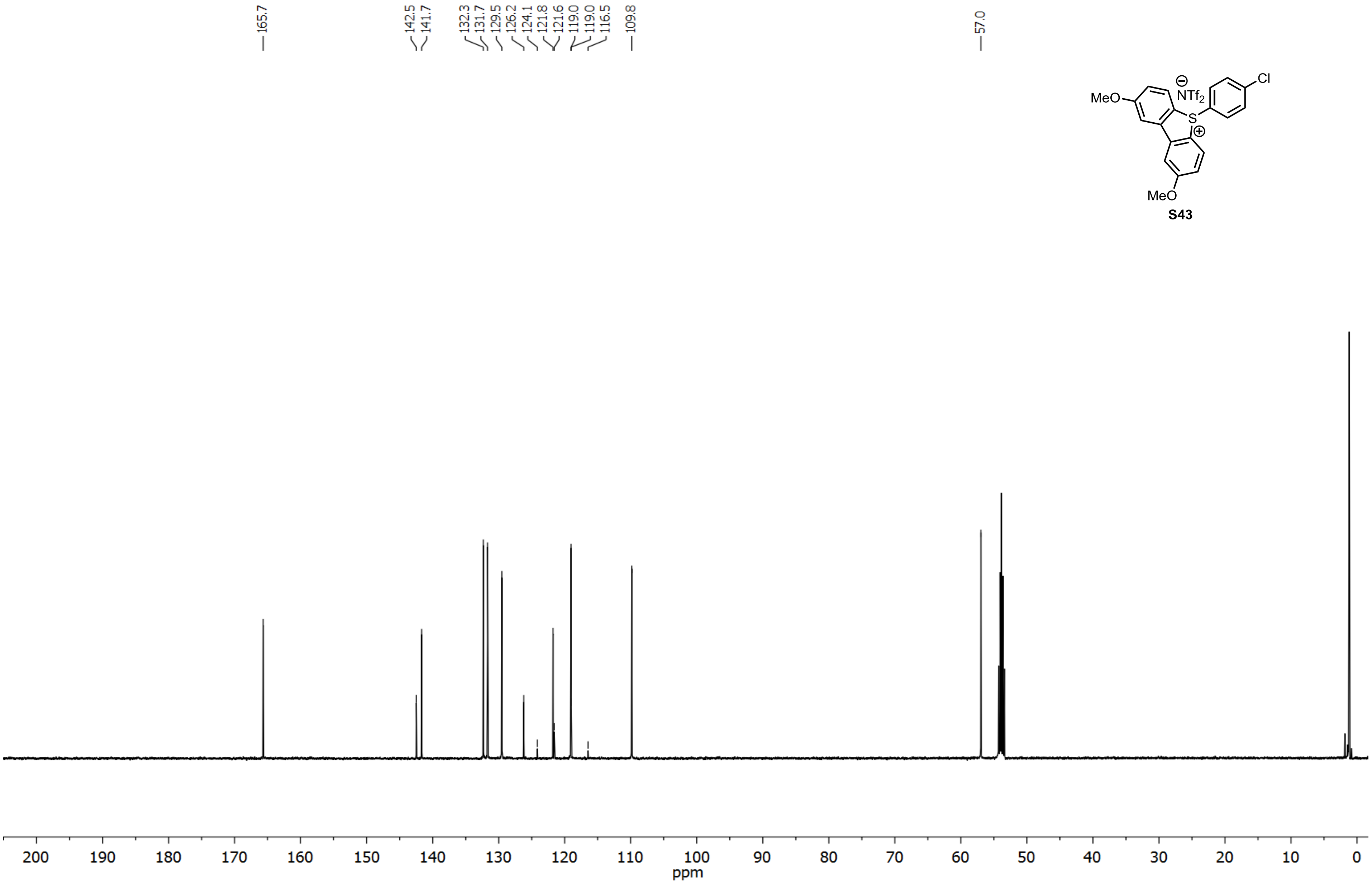

S43

^{19}F NMR of chlorobenzene-derived 2,8-dimethoxydibenzothiophenium salt S43

CD_2Cl_2, 298 K

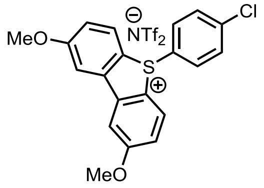

S43

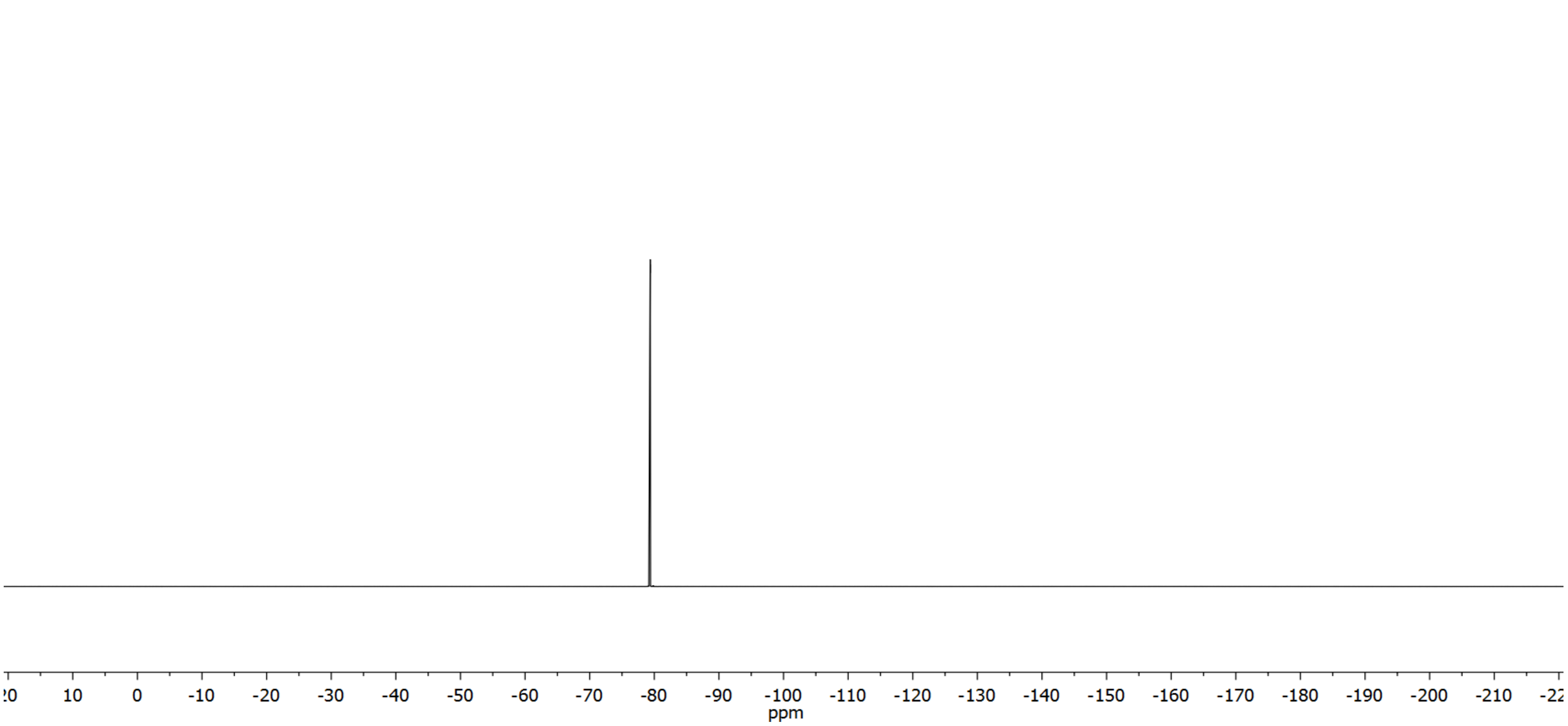

^{1}H NMR of benzotrifluoride-derived 2,8-dimethoxydibenzothiophenium salt S44

CD_2Cl_2, 298 K

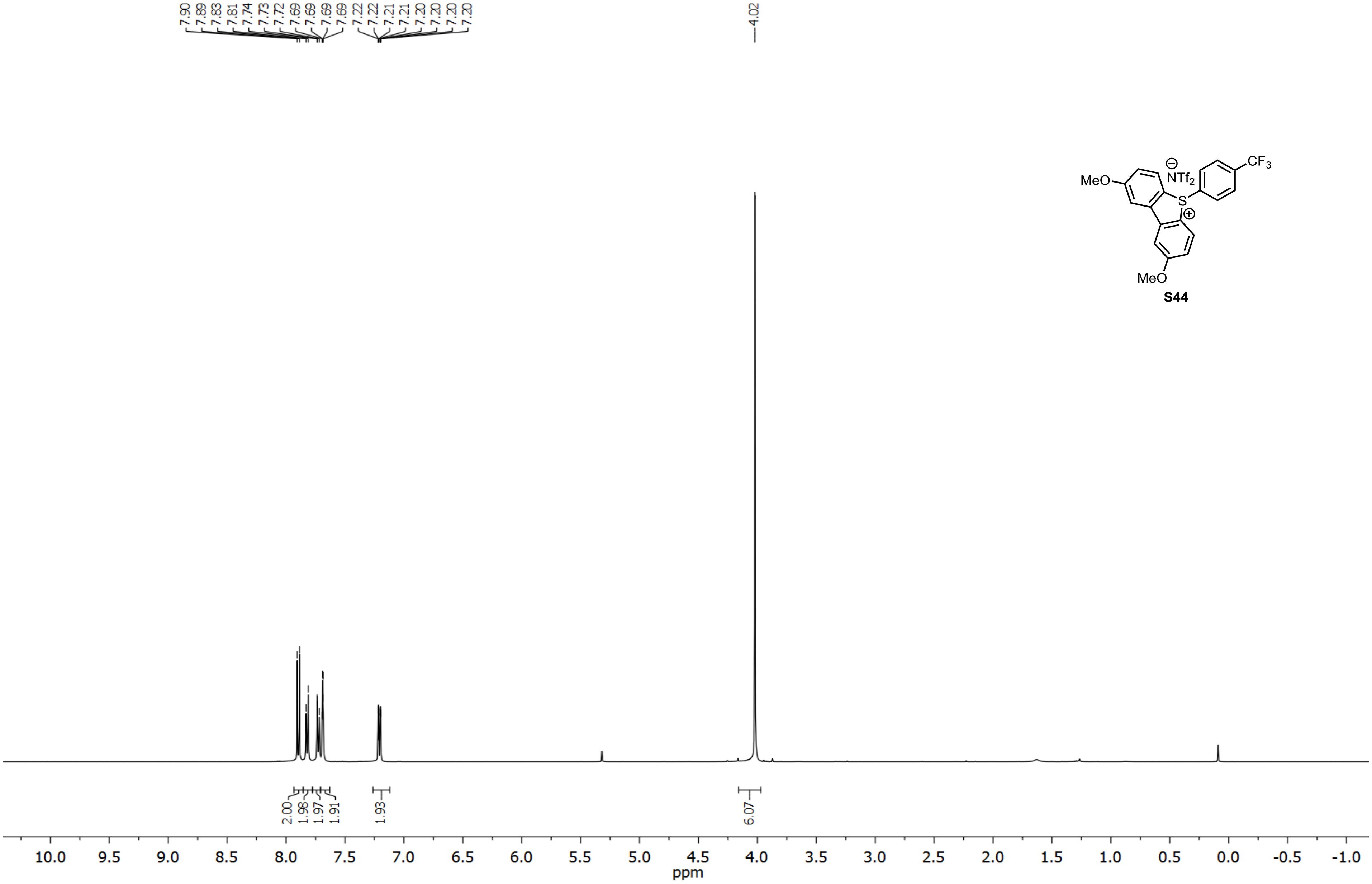

^{13}C NMR of benzotrifluoride-derived 2,8-dimethoxydibenzothiophenium salt S44

CD_2Cl_2, 298 K

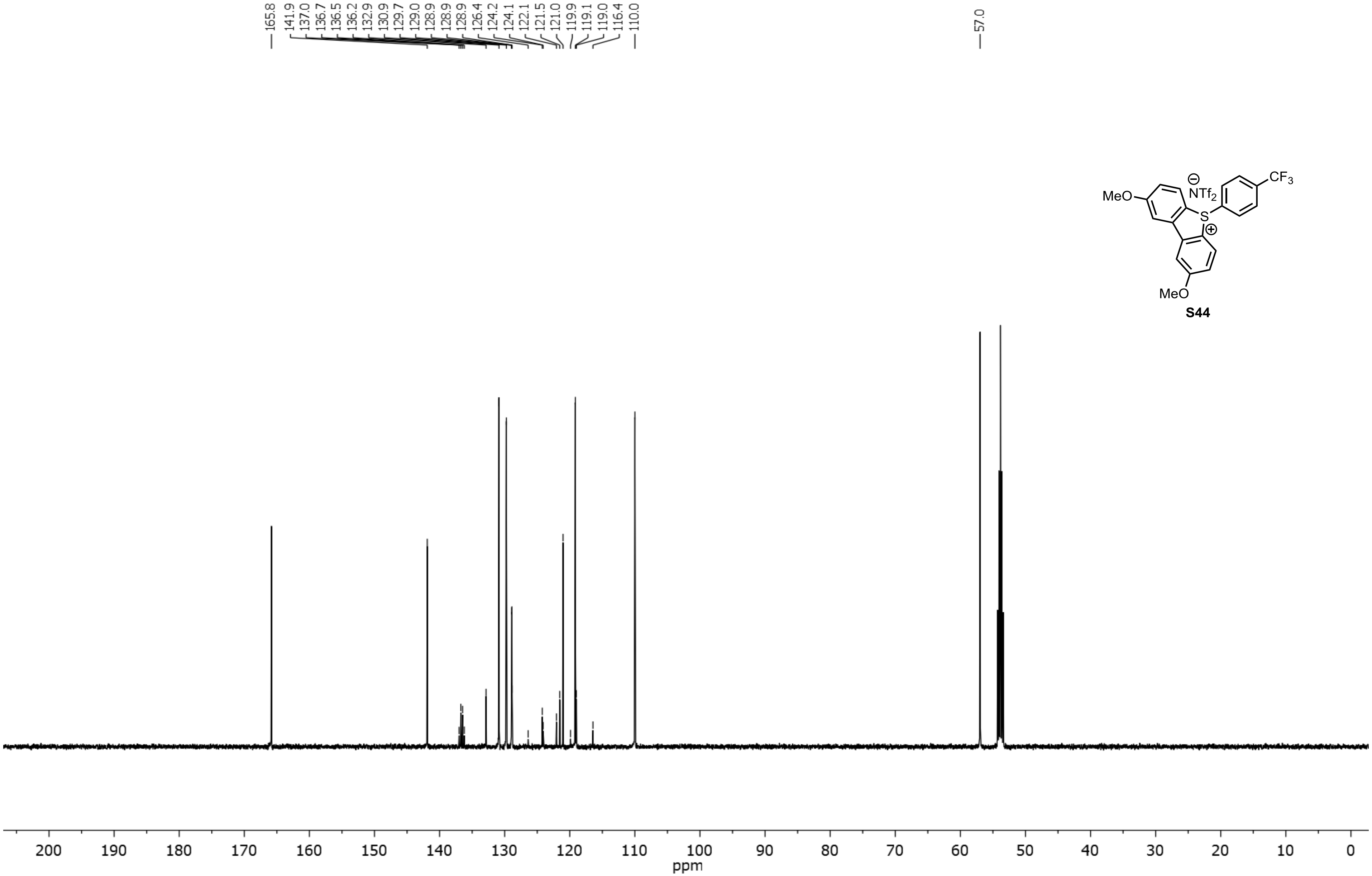

S44

^{19}F NMR of benzotrifluoride-derived 2,8-dimethoxydibenzothiophenium salt S44

CD_2Cl_2, 298 K

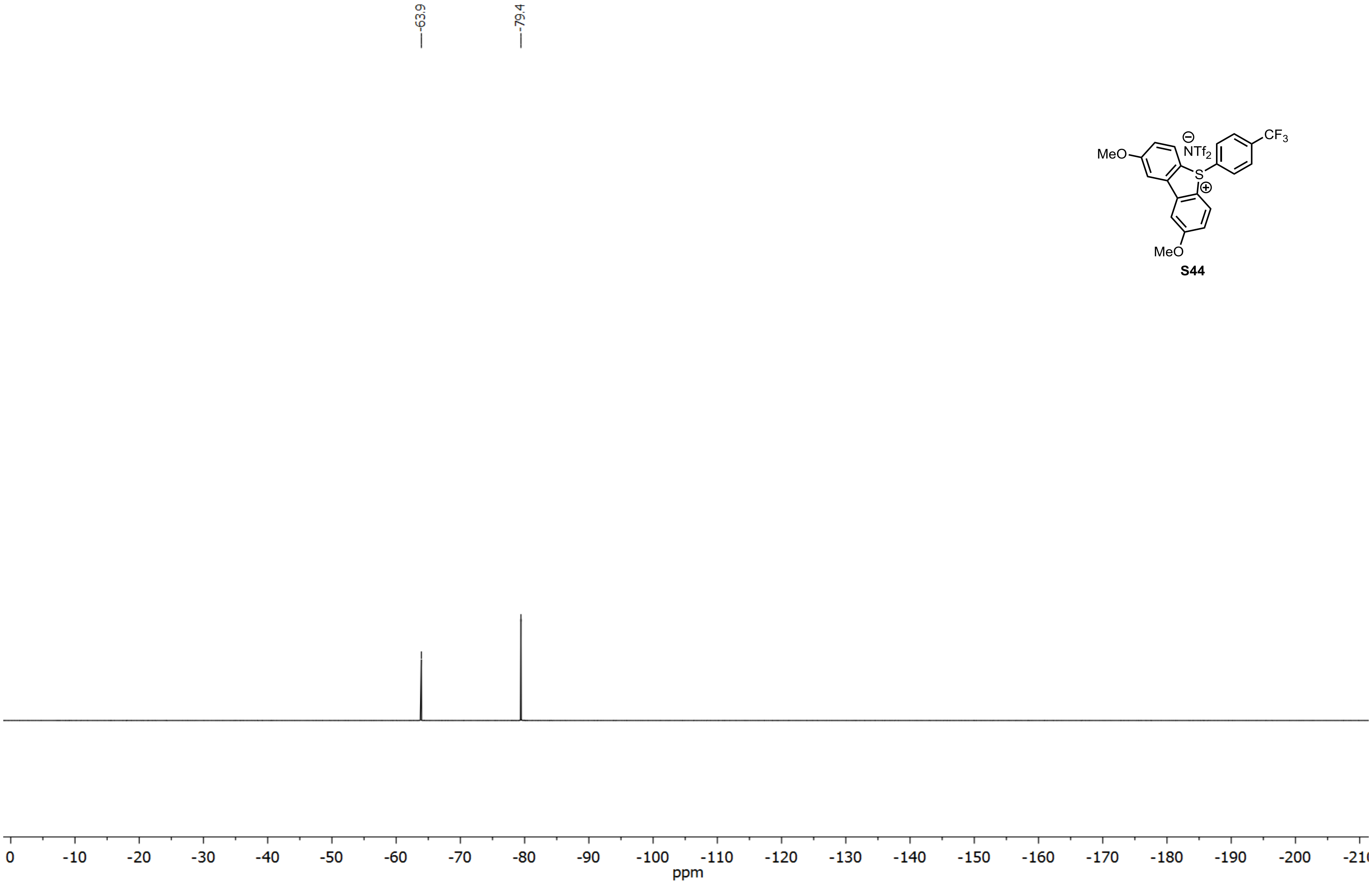

S44

^{1}H NMR of ethylbenzene-derived *para*-dibenzothiophenium salt S45

CD_3CN, 298 K

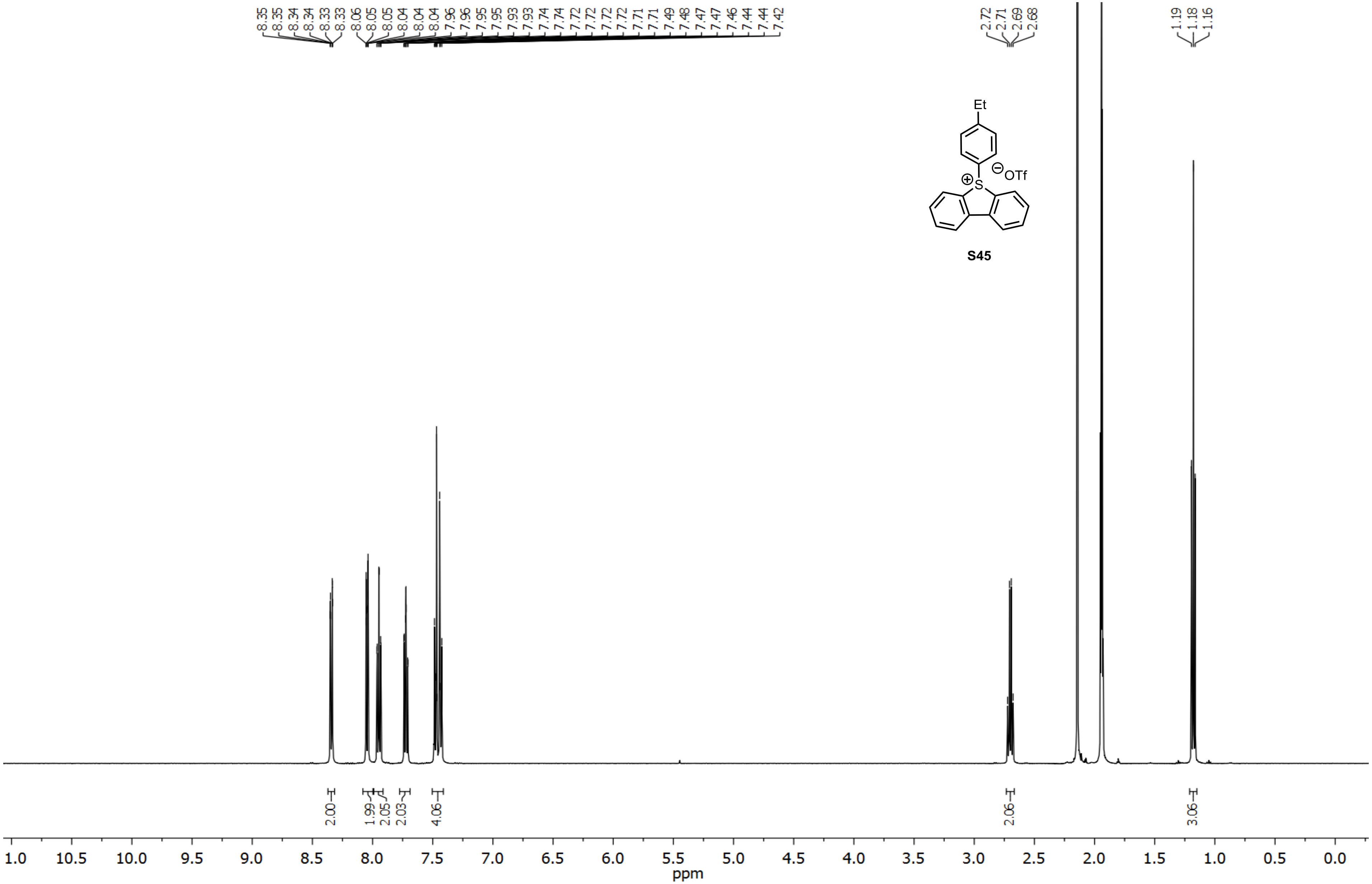

^{13}C NMR of ethylbenzene-derived *para*-dibenzothiophenium salt S45

CD_3CN, 298 K

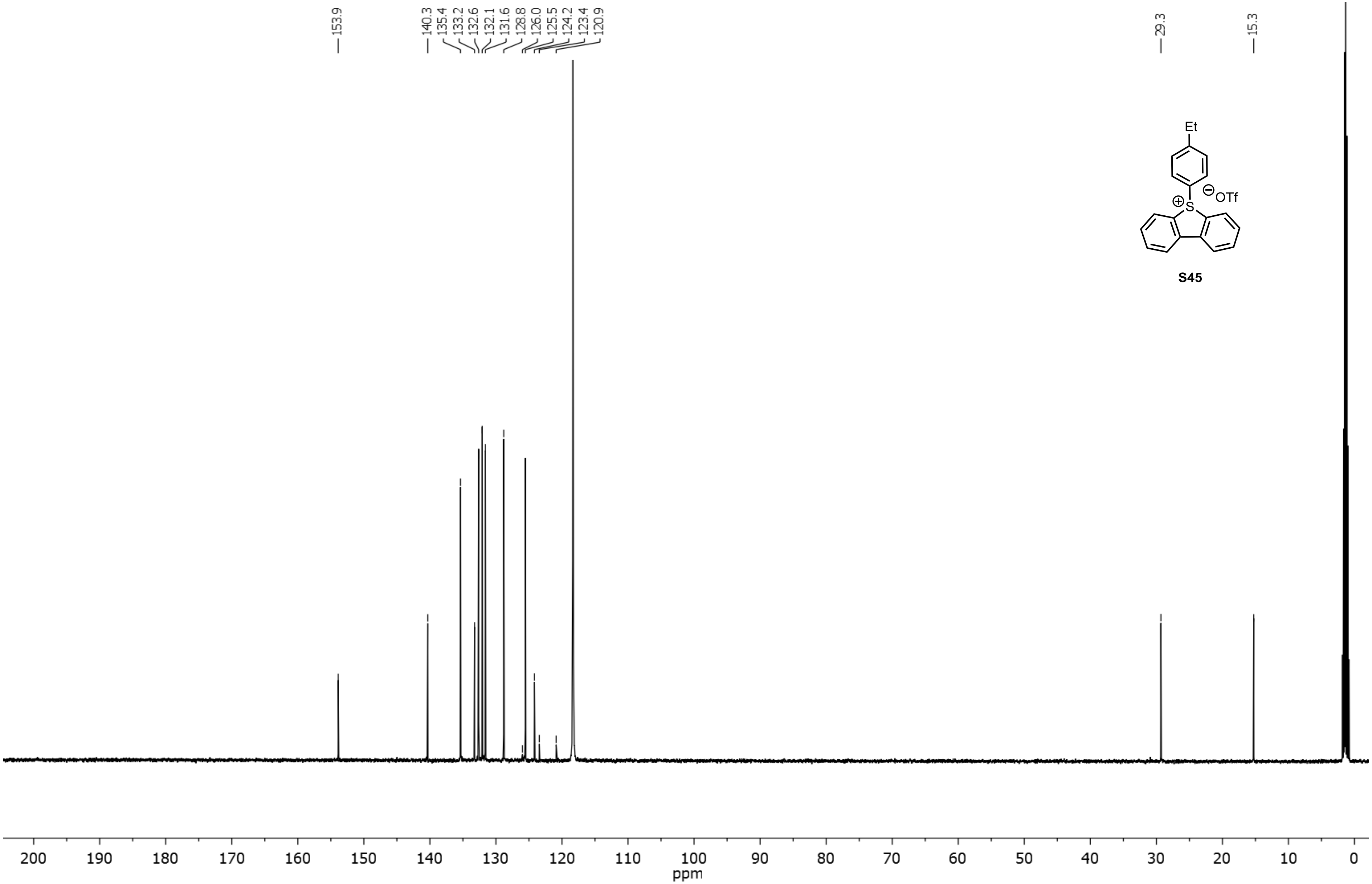

^{19}F NMR of ethylbenzene-derived *para*-dibenzothiophenium salt S45

CD$_3$CN, 298 K

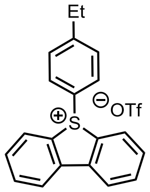

S45

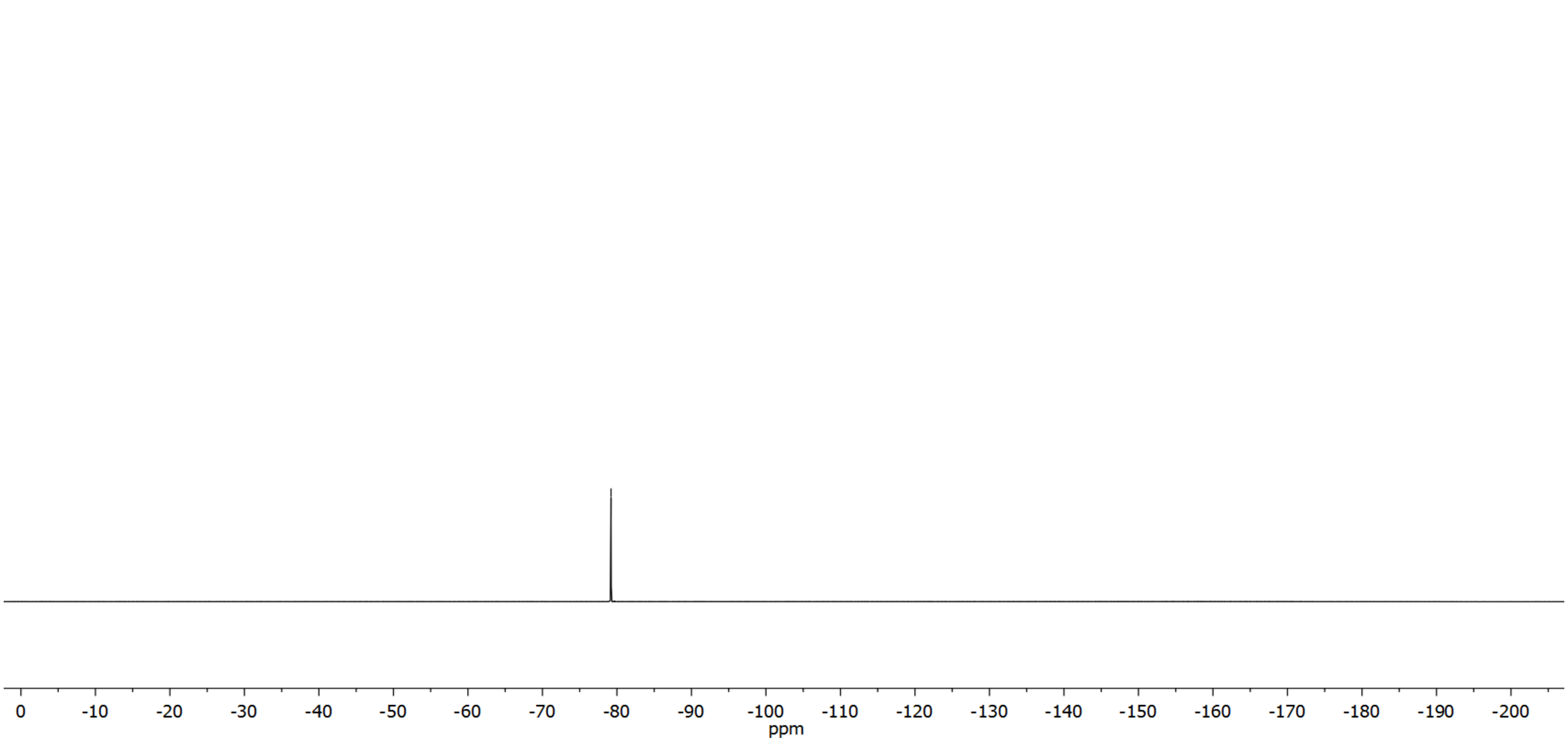

^{1}H NMR of ethylbenzene-derived *ortho*-dibenzothiophenium salt S46

$CDCl_3$, 298 K

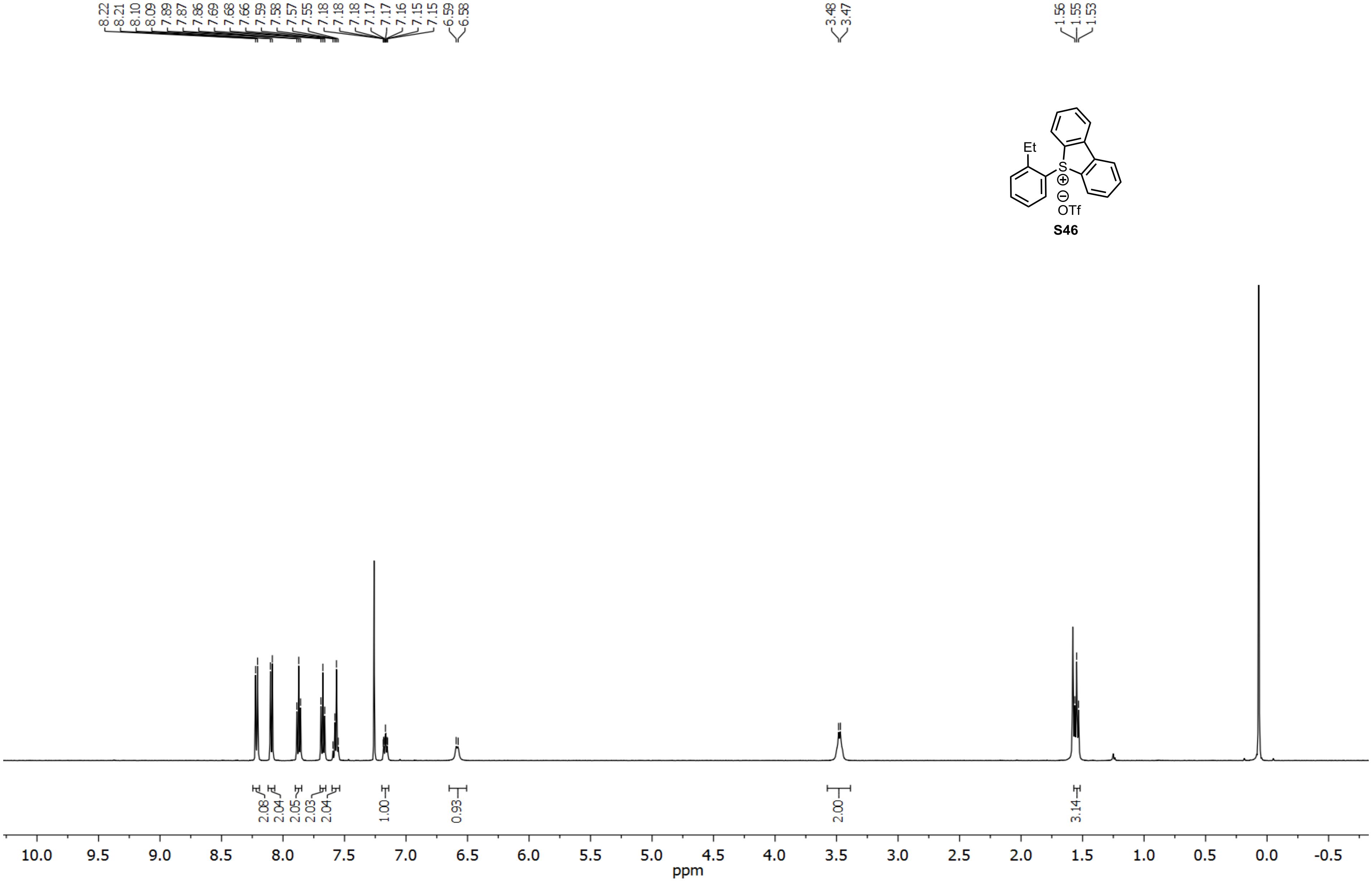

^{13}C NMR of ethylbenzene-derived *ortho*-dibenzothiophenium salt S46

CDCl$_3$, 298 K

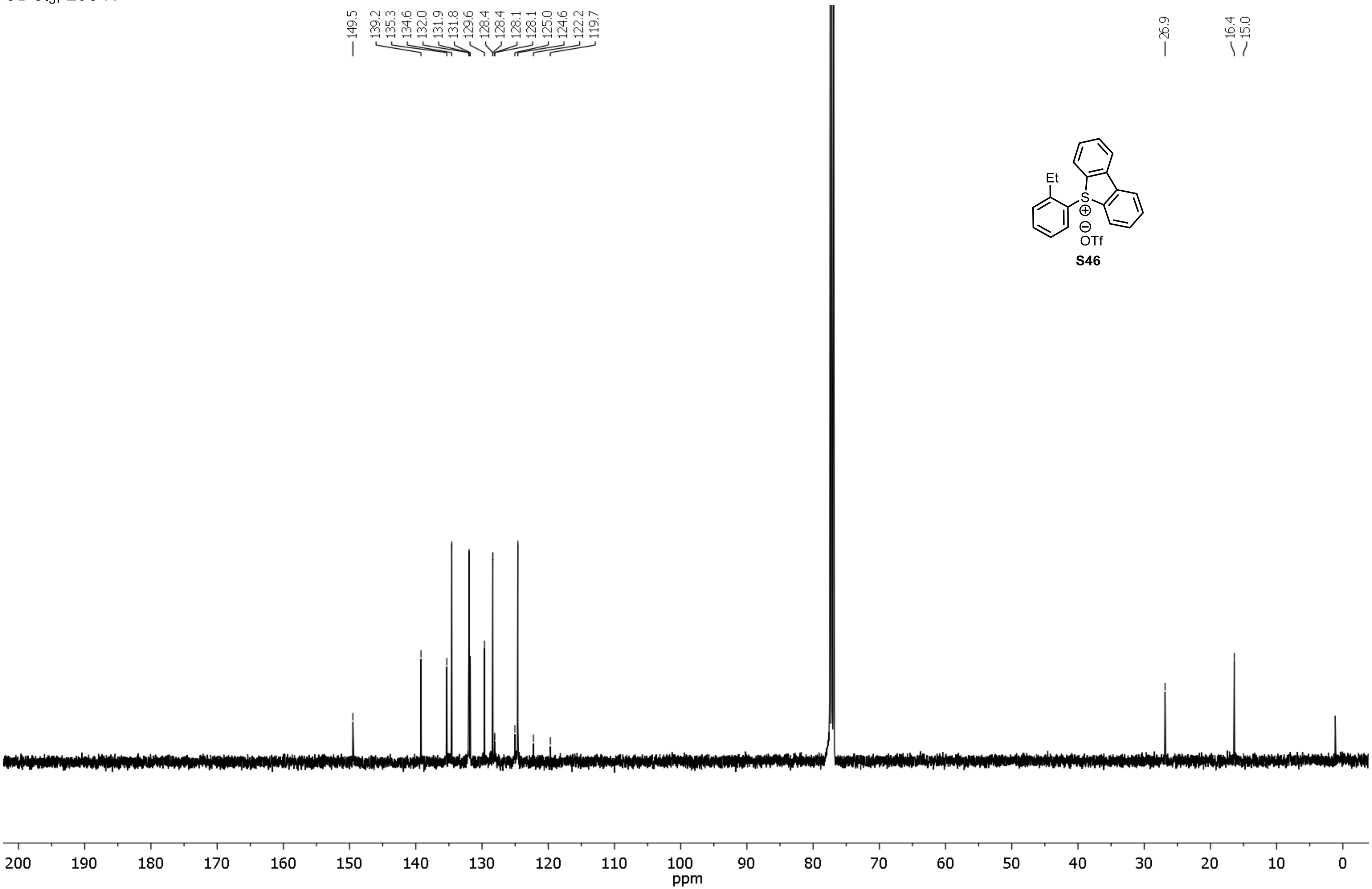

^{19}F NMR of ethylbenzene-derived *ortho*-dibenzothiophenium salt S46

$CDCl_3$, 298 K

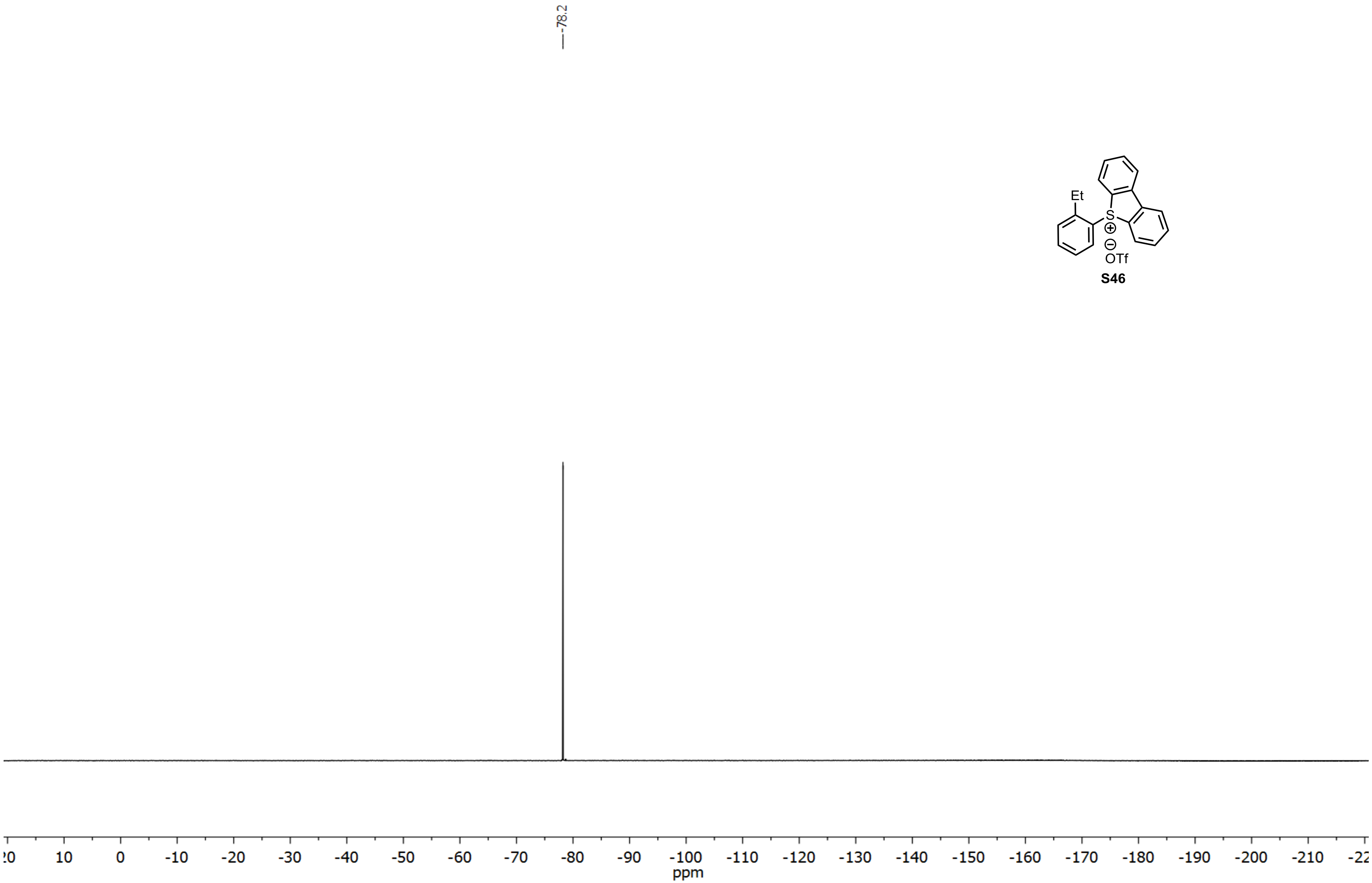

^{1}H NMR of ethylbenzene-derived *meta*-dibenzothiophenium salt S47

$CDCl_3$, 298 K

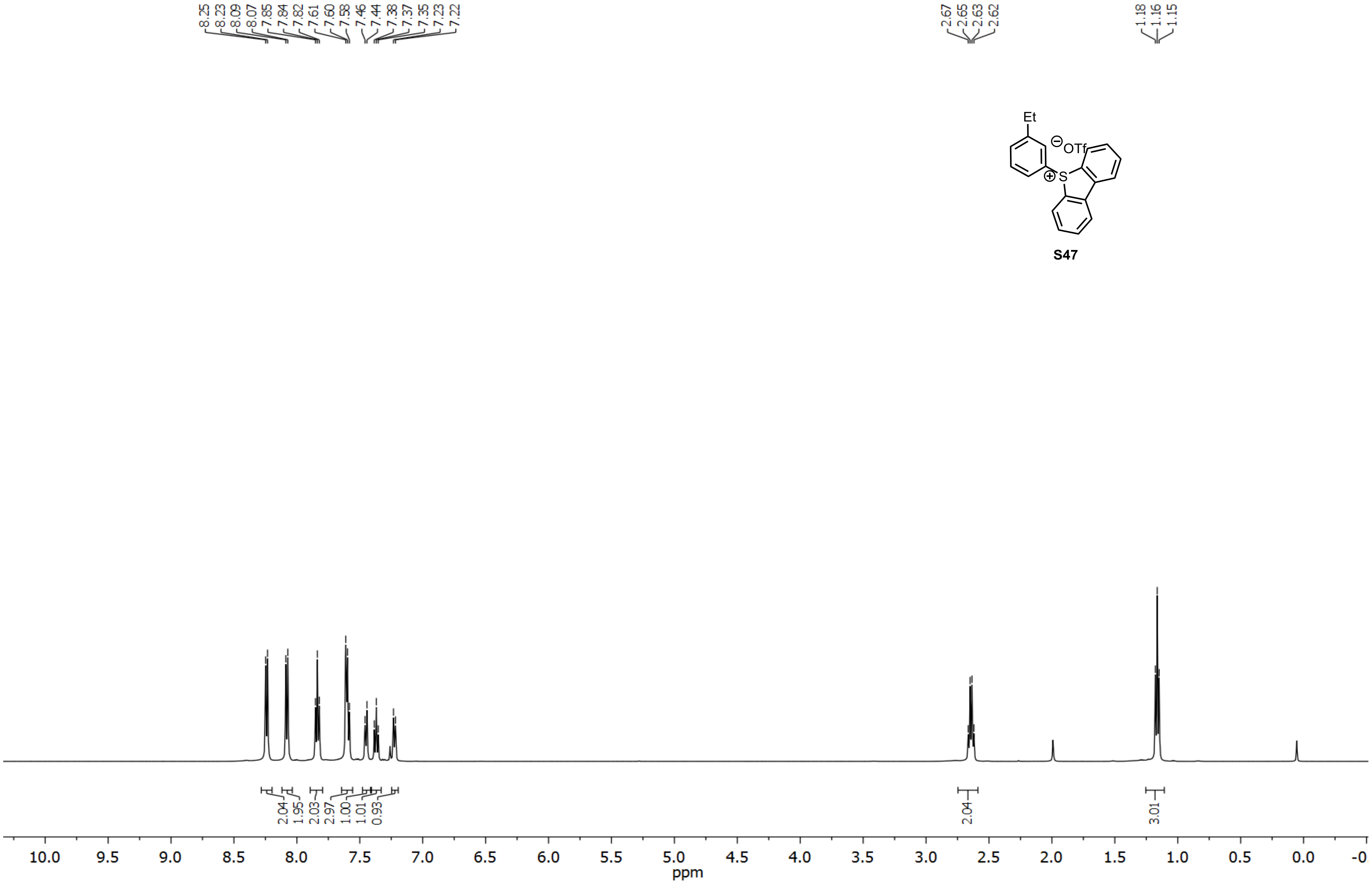

^{13}C NMR of ethylbenzene-derived *meta*-dibenzothiophenium salt S47

CDCl$_3$, 298 K

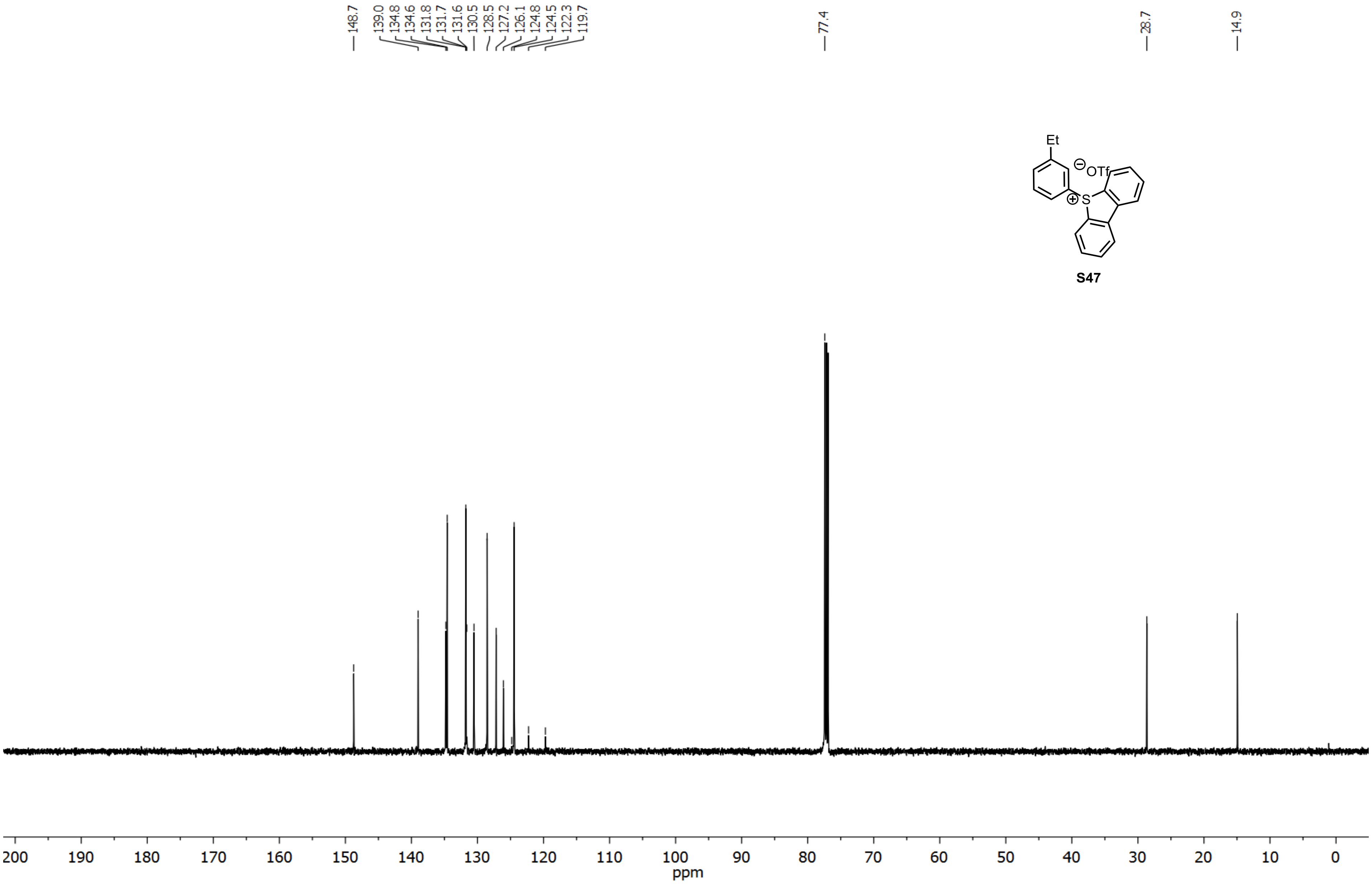

^{19}F NMR of ethylbenzene-derived *meta*-dibenzothiophenium salt S47

CDCl$_3$, 298 K

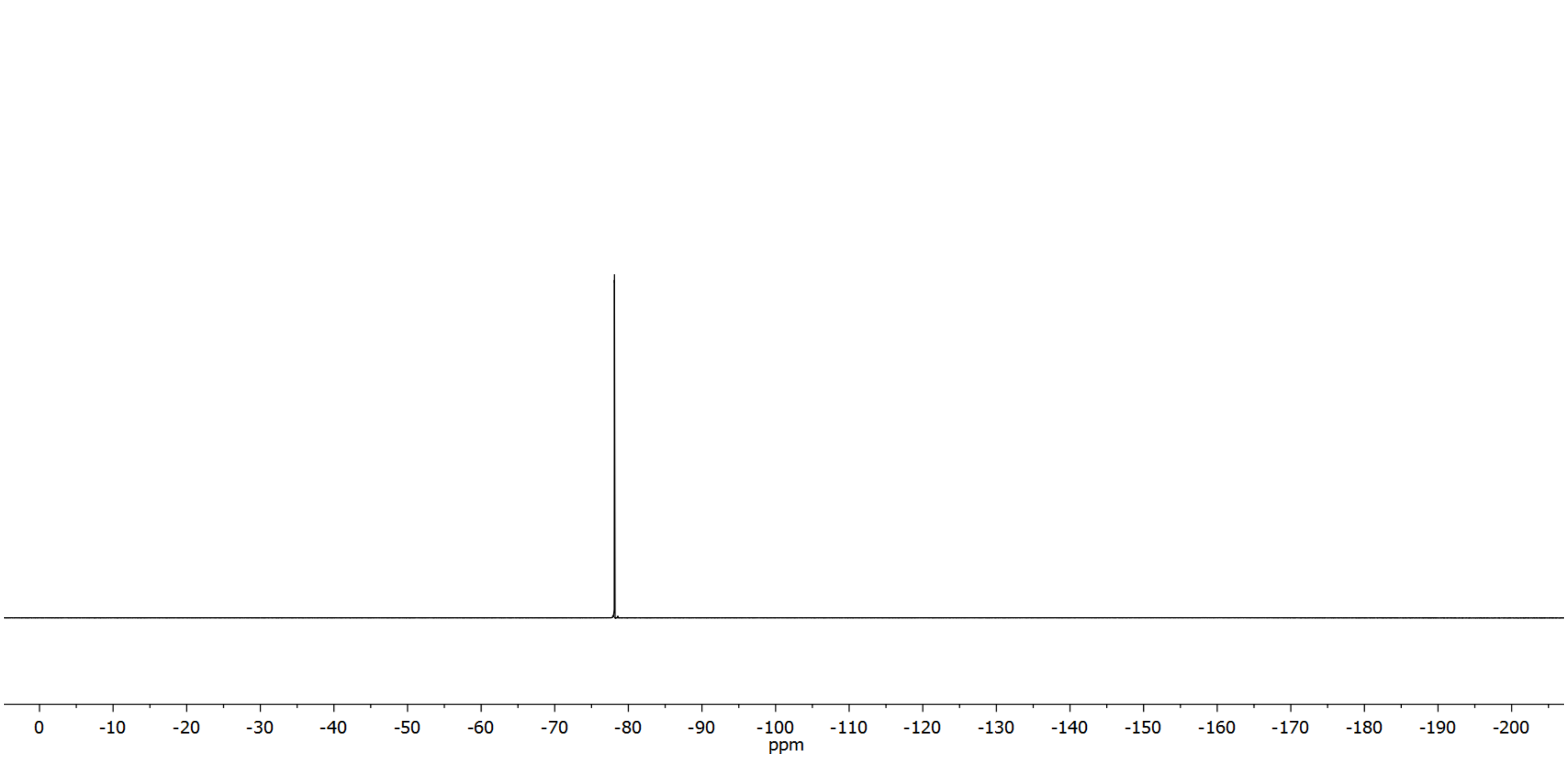

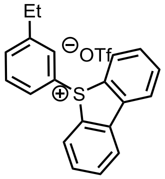

S47

^{1}H NMR of ibuprofen methylester-derived dibenzothiophenium salts S48a and S48b

$CDCl_3$, 298 K

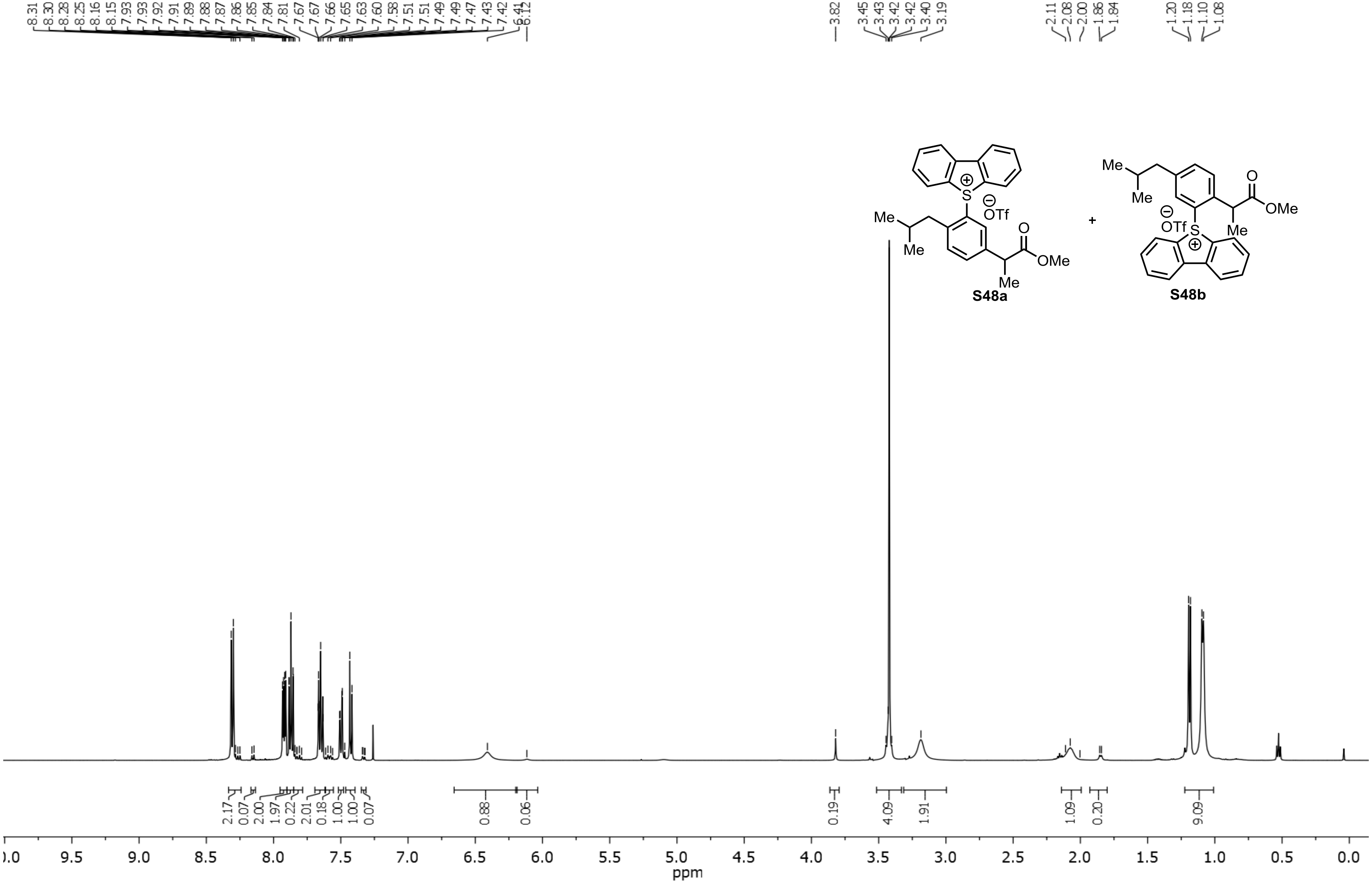

^{13}C NMR of ibuprofen methylester-derived dibenzothiophenium salts S48a and S48b

$CDCl_3$, 298 K

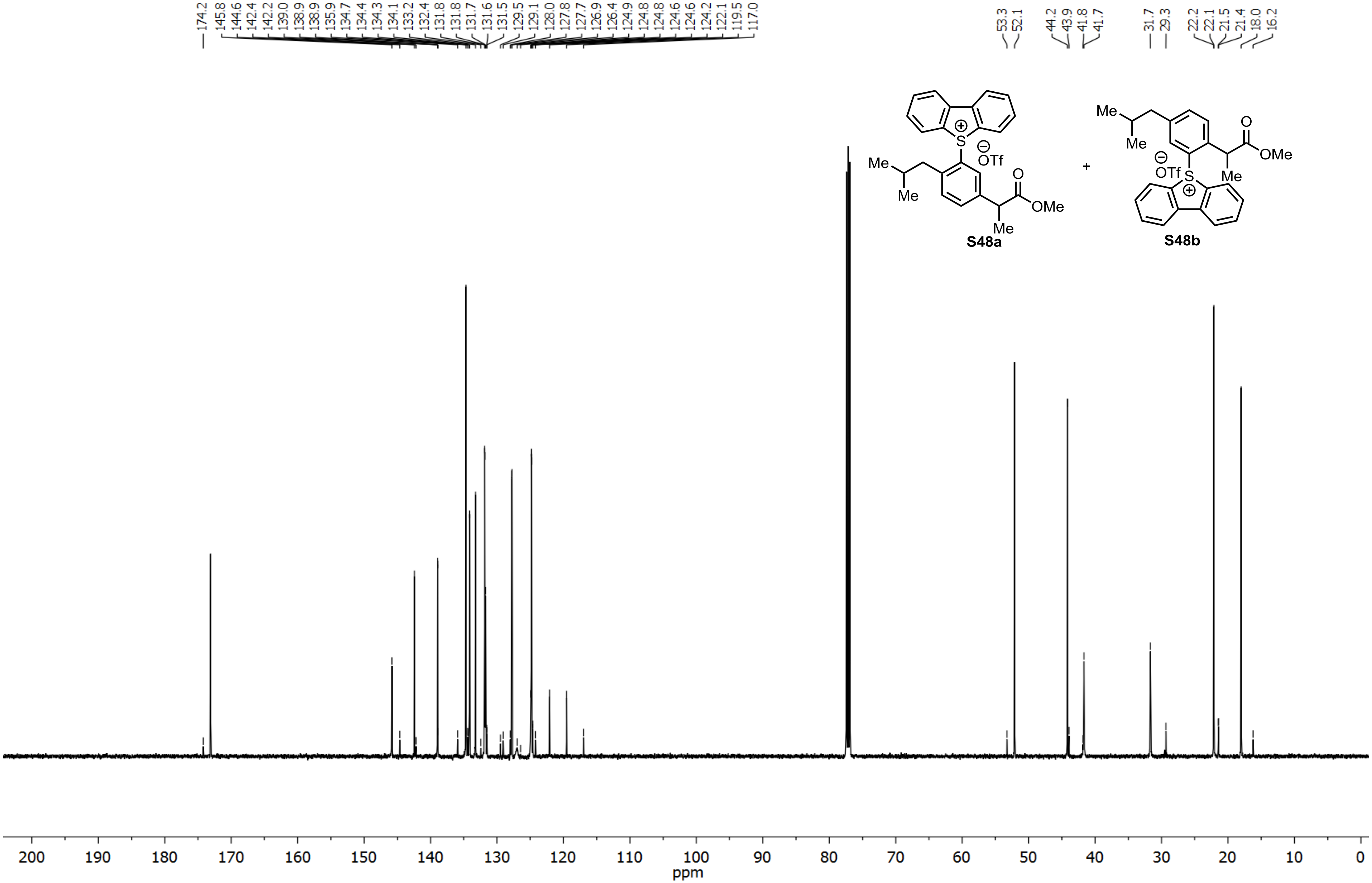

^{19}F NMR of ibuprofen methylester-derived dibenzothiophenium salts S48a and S48b

$CDCl_3$, 298 K

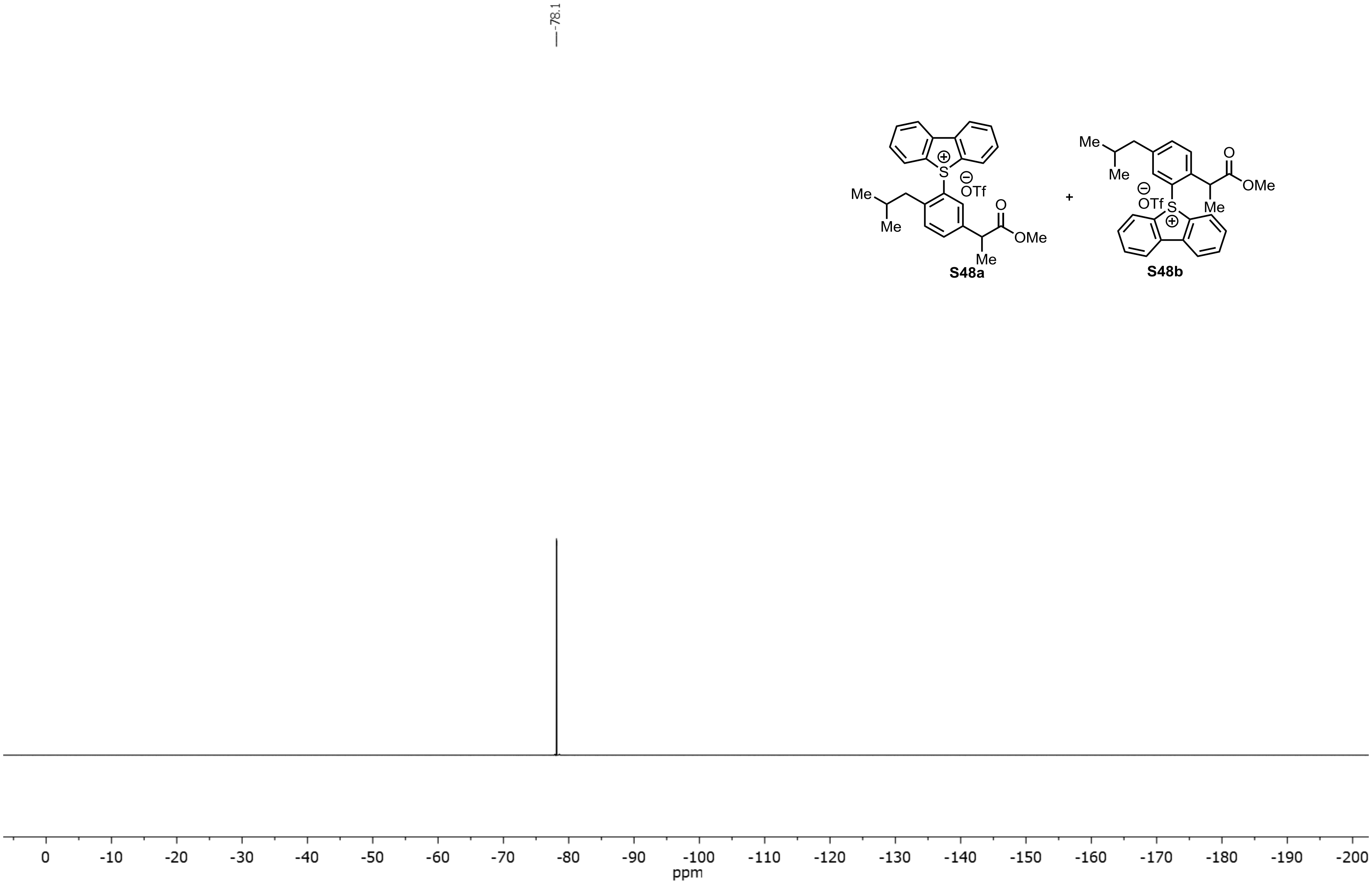

Supplement: Supplementary file 1 — Supplementary [file ANIE-59-1956-s001.pdf]
